# Novel HDAC inhibitors exhibit pre-clinical efficacy in lymphoma models and point to the importance of *CDKN1A* expression levels in mediating their anti-tumor response

**DOI:** 10.18632/oncotarget.3239

**Published:** 2014-12-30

**Authors:** Afua Adjeiwaa Mensah, Ivo Kwee, Eugenio Gaudio, Andrea Rinaldi, Maurilio Ponzoni, Luciano Cascione, Gianluca Fossati, Anastasios Stathis, Emanuele Zucca, Gianluca Caprini, Francesco Bertoni

**Affiliations:** ^1^ Lymphoma & Genomics Research Program, IOR Institute of Oncology Research, Bellinzona, Switzerland; ^2^ Dalle Molle Institute for Artificial Intelligence (IDSIA), Manno, Switzerland; ^3^ Unit of Lymphoid Malignancies, Department of Onco-Hematology, San Raffaele Scientific Institute, Milan, Italy; ^4^ IOSI Oncology Institute of Southern Switzerland, Bellinzona, Switzerland; ^5^ Preclinical R&D Department, Italfarmaco S.p.A., Cinisello Balsamo, Milan, Italy

**Keywords:** histone deacetylase, CDKN1A, MYC, NFKB, STAT3

## Abstract

We investigated the pre-clinical activities of two novel histone deacetylase inhibitors (HDACi), ITF-A and ITF-B, in a large panel of pre-clinical lymphoma models. The two compounds showed a dose-dependent anti-proliferative activity in the majority of cell lines. Gene expression profiling (GEP) of diffuse large B-cell lymphoma (DLBCL) cells treated with the compounds showed a modulation of genes involved in chromatin structure, cell cycle progression, apoptosis, B-cell signaling, and genes encoding metallothioneins. Cell lines showed differences between the concentrations of ITF-A and ITF-B needed to cause anti-proliferative or cytotoxic activity, and cell cycle and apoptosis genes appeared implicated in determining the type of response. In particular, *CDKN1A* expression was higher in DLBCL cells that, to undergo apoptosis, required a much higher amount of drug than that necessary to induce only an anti-proliferative effect.

In conclusion, the two novel HDACi ITF-A and ITF-B demonstrated anti-proliferative activity across different mature B-cell lymphoma cell lines. Basal *CDKN1A* levels appeared to be important in determining the gap between HDACi concentrations causing cell cycle arrest and those that lead to cell death.

## INTRODUCTION

Despite the important advances made in the treatment of B-cell lymphomas, particularly with the addition of the monoclonal antibody Rituximab to standard chemotherapy, significant numbers of these neoplasms are still refractory to currently used therapies [1, 2]. The importance of epigenetic mechanisms in lymphomagenesis is known, with specific histone and chromatin modification enzymes targeted by inactivating mutations and genomic aberrations [3, 4]. These observations suggest that targeting of the epigenome in lymphomas may be therapeutically beneficial.

The family of histone deacetylases (HDACs) is divided into four classes: class I HDACs are predominantly localized in the nucleus and comprise HDAC1, 2, 3 and 8; class II HDACs, subdivided into class IIa (HDAC4, 5, 7, 9) and class IIb (HDAC6 and 10), shuttle between the nucleus and cytoplasm; class III HDACs comprise Sirtuins 1-7 and are mainly localised in the nucleus and mitochondrion. Differently from the zinc-dependent HDACs in the other three classes, Sirtuins require NAD+ to catalyse deacetylation. HDAC class IV only contains HDAC11, a nuclear HDAC [5, 6]. HDACs are aberrantly expressed in various solid and hematological cancers including diffuse large B-cell lymphomas (DLBCLs) and peripheral T-cell lymphomas (PTCLs) [7-9]. Due to their targeting of multiple histone and non-histone proteins, including regulators of proliferation and apoptosis, alterations in their expression can contribute to tumorigenesis [5].

HDAC inhibitors (HDACi) function by interfering in the interaction between HDACs and their substrates [5, 6, 10-13]. Several structurally distinct classes of HDACi exist and, of these, the hydroxamic acid derivatives are the most potent [6, 11]. HDACi have shown pre-clinical and clinical efficacy in T-cell lymphomas leading to the approval of vorinostat [14], romidepsin [15] and, most recently, belinostat [16] for the treatment of relapsed or refractory cutaneous T-cell lymphomas and PTCLs. HDACi appear to exert their anti-tumor activities primarily by targeting gene transcription [13, 17]. The most common anti-tumor response to treatment with HDACi is cell cycle arrest in G1. Arrest in G1 is associated with transcriptional activation of *CDKN1A*, the gene encoding the cyclin dependent kinase inhibitor, p21^WAF1/CIP1^ [11, 13, 18]. HDACi can also induce apoptosis and in most cases, the induction of G1 arrest or apoptosis is governed by drug concentration, with lower doses inducing cell cycle arrest and higher doses provoking cell death [10, 19, 20]. Cell type can also contribute to the type of proliferation inhibition and related to this, the genetic background of the cell may be an important factor [21]. The expression levels of several genes have been associated with the response to HDACi: overexpression of *BCL2, BCL2L1, STAT1, STAT3* and the antioxidant genes *TXN* (thioredoxin), *SOD2, GSR* (glutathione reductase) has been reported to correlate with resistance to HDACi [22, 23]. Induction of *CDKN1A* expression following HDACi treatment is also associated with resistance to HDACi-mediated cytotoxicity and cells that fail to upregulate CDKN1A/p21^WAF1/CIP1^ following HDACi treatment readily undergo apoptosis [19, 20].

Here, we assessed the preclinical activities and the mechanism of action of two novel HDACi, in over 30 lymphoma cell lines.

## RESULTS

### ITF-A and ITF-B exhibit anti-proliferative activities in lymphoma cell lines

**Figure 1:**
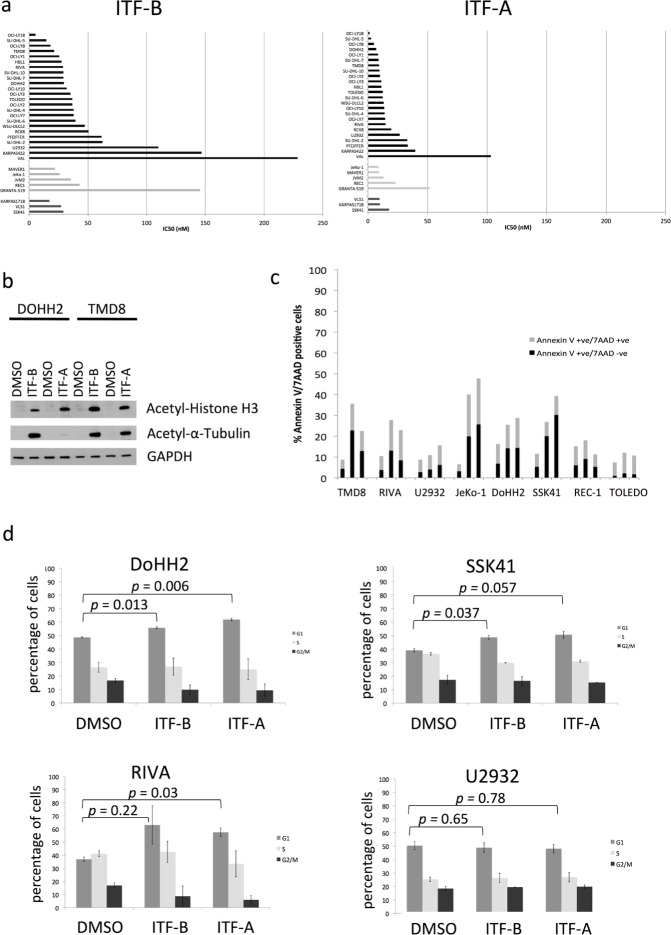
The novel histone deacetylase inhibitors ITF-B and ITF-A exhibit antiproliferative activities in a wide range of lymphoma cell lines (a) Individual IC50 values for ITF-B and ITF-A. The 32 cell lines are grouped and colored according to their lymphoma histology: DLBCL, black bars; MCL, light grey bars; SMZL, dark grey bars. IC50 values are in nM. IC50 values for ITF-B ranged from 5 nM to 228 nM while those for ITF-A ranged from 1.5 nM to 103 nM. (b) Twenty-four hours treatment of DoHH2 (GCB-DLBCL) and TMD8 (ABC-DLBCL) cells with 200nM ITF-B and 100nM ITF-A markedly induced acetylated histone H3 and acetylated α-tubulin proteins. (c) Apoptosis analysis in a panel of lymphoma cell lines following treatment with 50 nM ITF-B or 20 nM ITF-A for 72 hours. Dual staining with Annexin V and 7AAD was used to distinguish early and late apoptotic cells. For each bar in the graph, the lower part in black denotes the percentage of early apoptotic cells (Annexin V +ve/7AAD –ve), while the upper grey part denotes the percentage of late apoptotic/necrotic cells (Annexin V +ve/7AAD +ve). For each cell line, the bars are shown in the following order from left to right: DMSO-treated cells, cells treated with ITF-B, cells treated with ITF-A. (d) Cell cycle analyses of lymphoma cell lines following treatment with 50 nM ITF-B or 20 nM ITF-A for 72 hours. Following treatment, cells were fixed with 70% ethanol before staining with 7AAD for cell cycle analysis. The percentage of cells in each phase of the cell cycle is shown for each treatment type. Error bars represent the S.E. p-values were obtained using a two-tailed Student's t-test.

We assessed the anti-proliferative activities of two new HDACi, here termed ITF-A and ITF-B, using the MTT cell viability assay in 24 diffuse large B-cell lymphoma (DLBCL) cell lines, 5 mantle cell lymphoma (MCL) cell lines and 3 splenic marginal zone lymphoma (SMZL) cell lines. ITF-A and ITF-B presented dose-dependent anti-proliferative activities with median IC50 (50% Inhibitory Concentration) values of 12nM for ITF-A (range, 1.5nM – 103nM) and 34nM for ITF-B (range, 5 – 228nM). The majority of IC50 values were below 20nM and 50nM for ITF-A and ITF-B, respectively (Figure 1a). In addition to IC50 values, we also determined GI50 (50% Growth Inhibition), LC50 (50% Lethal Concentration) and TGI (Total Growth Inhibition) values (see Methods for further explanation). Similarly to the IC50 values, the ranges of GI50, LC50 and TGI were narrower for ITF-A than for ITF-B (Supplementary Figure 1a). The IC50 and GI50 are similar anti-proliferation parameters and comparbly to the IC50 values, most cell lines had GI50 values less than 20nM or 50nM for ITF-A and ITF-B, respectively. We did not observe any association between lymphoma histology and IC50, GI50, LC50 or TGI for either of the two HDACi (Supplementary Figure 1b).

We also assessed the ability of ITF-B and ITF-A to inhibit HDAC activity. The DLBCL cell lines DoHH2 and TMD8 treated with ITF-B or ITF-A showed increases in nuclear acetylated histone H3 and cytoplasmic acetylated α-tubulin levels (Figure 1b). Thus, ITF-B and ITF-A exhibited nuclear and cytoplasmic activities and caused the acetylation of both histone and non-histone proteins.

### The anti-proliferative effects of ITF-A and ITF-B are mainly due to a block in the cell cycle

Following the demonstration of the anti-proliferative effects of ITF-A and ITF-B in lymphoma cell lines, we performed cell cycle and apoptosis analyses. We treated cells with fixed doses of 20 nM ITF-A or 50 nM ITF-B for 72 hours. Only the MCL cell line JeKo-1 exhibited a marked induction of apoptosis, as determined by flow cytometry analysis of Annexin V-stained cells (Figure 1c), also supported by cell cycle analysis, which showed a prominent accumulation of sub-G1 cells (data not shown). Among the eight cell lines, the induction of apoptosis could not be predicted from IC50 or GI50 values: seven out of eight cell lines had IC50 or GI50 values that were lower than the administered doses of HDACi but did not exhibit a marked induction of apoptosis. Cell cycle analysis at the used doses showed that in the absence of apoptosis, arrest in G1 was responsible for the anti-proliferative effects of the two HDACi (Figure 1d, Supplementary Figure 2a). Of the cell lines tested, the DLBCL cell line U2932 was the only one that did not undergo arrest in G1 in response to HDACi treatment, likely because its IC50 and GI50 values were much higher than the doses used for these experiments. These results indicate that at doses representative of the IC50 or GI50 the main anti-proliferative effect of the two HDACi is to block the cell cycle. This observation is in agreement with reported results obtained with other HDACi [10, 24], which usually mediate a block in the cell cycle. Since we also had data indicating concentrations at which each HDACi could be cytotoxic, we treated selected lymphoma cell lines with the respective LC50 doses of each HDACi. At the LC50 doses we observed a marked induction of apoptosis in all cell lines tested, confirming that at these doses, both HDACi were able to induce cell death (Supplementary Figure 2b).

### Treatment of DLBCL cell lines with ITF-A and ITF-B induces diverse transcriptional changes

**Figure 2:**
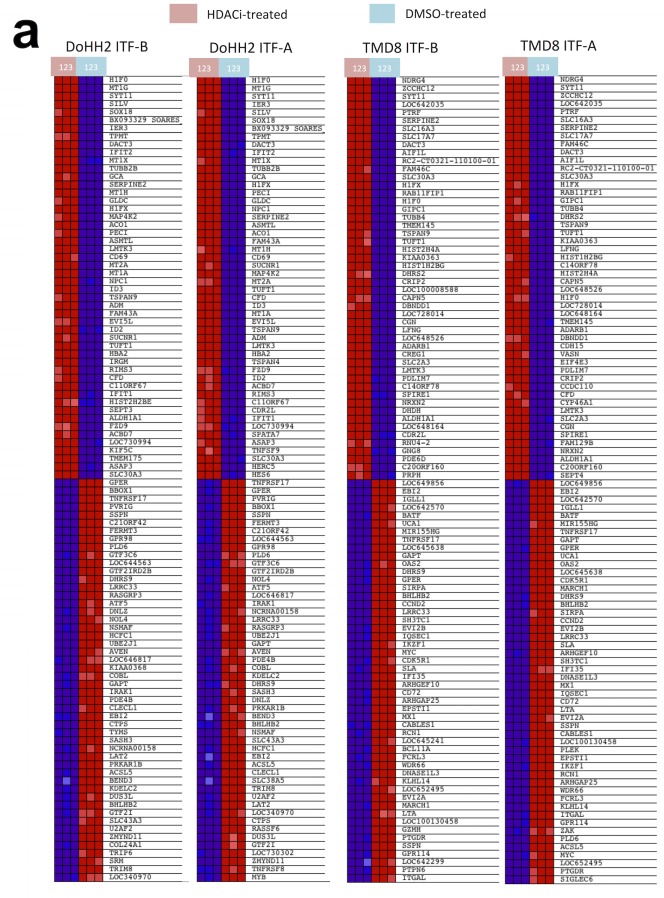

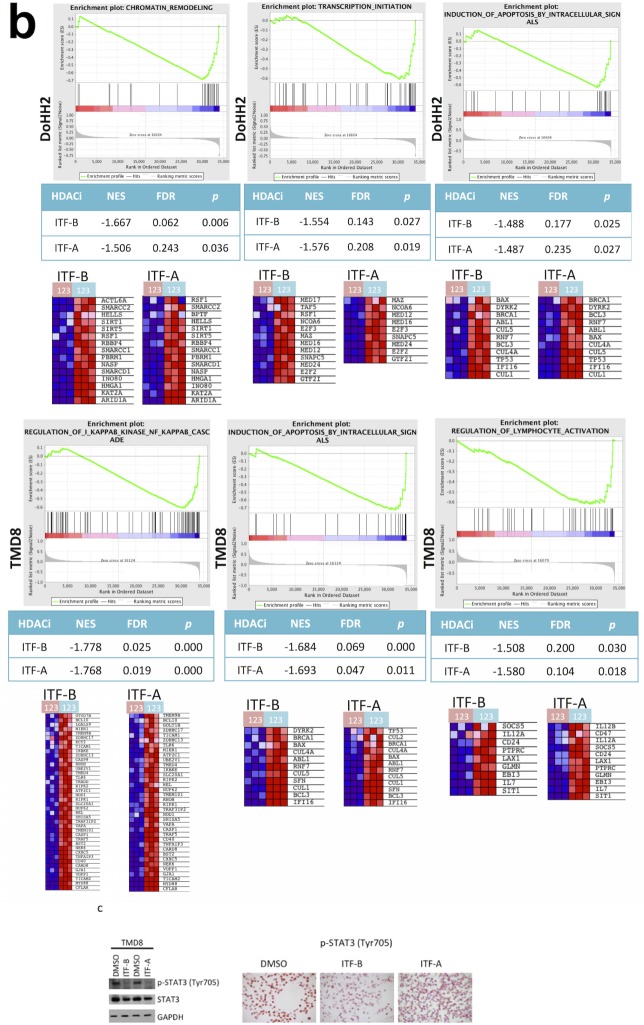
Treatment of DLBCL cell lines with ITF-B and ITF-A similarly modulates the expression of multiple genes DoHH2 (GCB-DLBCL) and TMD8 (ABC-DLBCL) cells were treated for 8 hours with ITF-B (200 nM) or ITF-A (100 nM). Control treated cells to which equivalent concentrations of DMSO were added were included. Total RNA extracted from treated cells was subjected to GEP, followed by Gene Set Enrichment Analysis (GSEA) of the GEP data. The individual heatmaps show the top 50 up-regulated (upper part of heatmap) and top 50 down-regulated (lower part of heatmap) genes in each cell line following treatment with each HDACi. Samples labeled with a pink box are the HDACi-treated cells from three independent experiments while samples labeled with a blue box are the corresponding DMSO-treated cells. Red denotes high expression, blue denotes low expression. For each cell line, many of the top 50 up- and down-regulated genes were similarly modulated by the two HDACi. This was also true when comparing the two cell lines. (b) Enrichment plots for gene sets that are statistically significant in both ITF-B- and ITF-A-treated cells. Enrichment plots in the upper panel are for DoHH2 cells, those in the lower panel are for TMD8 cells. Below each enrichment plot are the corresponding heatmaps for each HDACi showing the core enrichment genes. The pink and blue boxes to the left of each heatmap indicate HDACi and DMSO-treated samples, respectively as described above. NES = normalized enrichment score, FDR = False discovery rate q-value. The enrichment plots shown are for ITF-B and are similar to those obtained for ITF-A. (c) Left panel, immunoblotting demonstrates the up-regulation of phosphorylated STAT3 (Tyr705) and total STAT3 in the ABC-DLBCL cell line TMD8 following 24 hours treatment with 200 nM ITF-B or 100 nM ITF-A. Right panel, Immunohistochemical staining shows that phosphorylated STAT3 is down-regulated and absent from most nuclei in TMD8 cells treated for 24 hours with 200 nM ITF-B or 100 nM ITF-A.

To better understand the transcriptional changes that occur following treatment of cells with ITF-A and ITF-B, we performed gene expression profiling (GEP) experiments on two DLBCL cell lines: DoHH2 (GCB-DLBCL) and TMD8 (ABC-DLBCL). Cells were treated for 8 hours with 100nM of ITF-A, 200nM of ITF-B or DMSO. Treatment with either HDACi affected diverse processes in both cell lines as determined by Gene Set Enrichment Analysis (GSEA). Similar gene sets were enriched in both cell lines, suggesting that the two HDACi similarly modulate specific processes as well as the expression of multiple genes in DLBCL cells (Figures 2a, 2b). Commonly modulated gene sets included those defining signaling processes, those defining the structure and composition of chromatin, gene sets determining the transcription and translation of nucleic acids as well as gene sets comprising genes involved in cell cycle progression and apoptosis (Figure 2b, Supplementary Tables 1-4). For the ABC cell line TMD8, gene sets for the NfκB signaling cascade were also modulated (Supplementary Tables 3 and 4). The number and variety of gene sets obtained following HDACi treatment demonstrated the widespread effects of the two HDACi on diverse cellular processes, a phenomena also observed for other HDACi [17, 25]. When comparing transcriptional changes at the single gene level, genes that were commonly up-regulated for both cell lines following treatment with either HDACi comprised anti-proliferation and pro-apoptotic genes, including several cyclin-dependent kinase inhibitors e.g. *CDKN1A*, *CDKN2D* and *CDKN2C*. Among the top 100 most up-regulated genes, genes encoding histones, tubulin genes and metallothionein genes were frequently represented (Supplementary Tables 5 and 6). Down-regulated genes included pro-survival and pro-proliferation genes such as *MYC*, *CCND2* and *NFKB1*. Among the top 100 most down-regulated genes, genes important for B-cell signaling were also represented: *IRAK1*, *SYK* and *BTK* (Supplementary Tables 5 and 6). Quantitative real-time PCR (qPCR) of selected up- and down-regulated genes (*IRAK1*, *TNFRSF17*, *HISTH2BE*) supported the GEP results (Supplementary Figure 3). Western blot and immunohistochemical analyses of total STAT3 and phospho-STAT3 (Tyr705) showed marked down-regulation of both proteins in TMD8 cells treated with either of the two HDACi (Figure 2c).

### Cell lines transcriptomes determine the response to ITF-A and ITF-B

**Figure 3:**
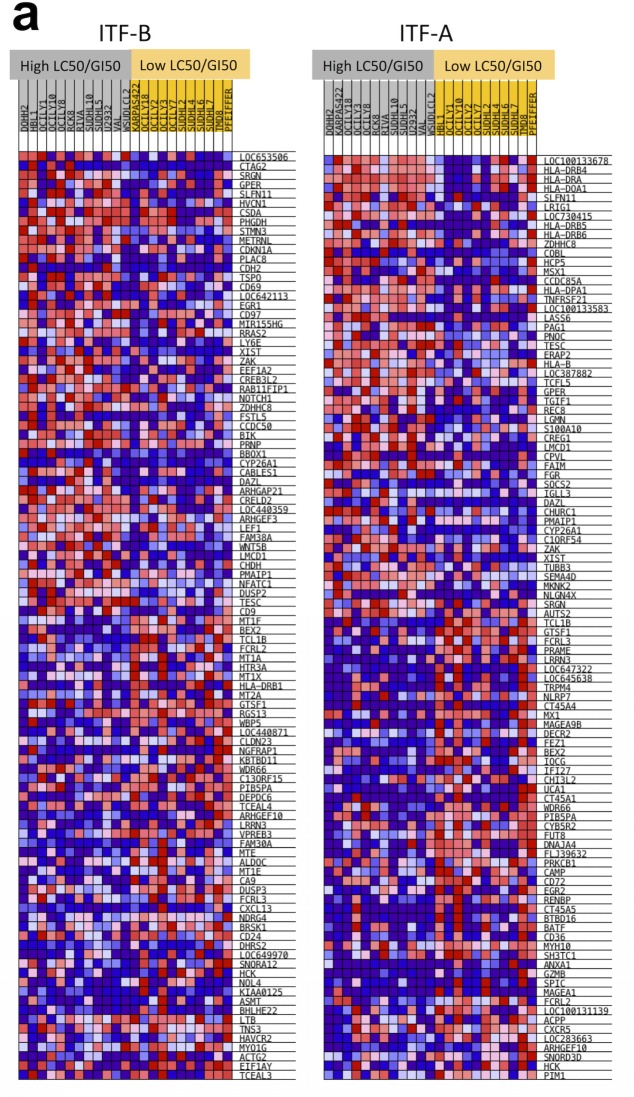

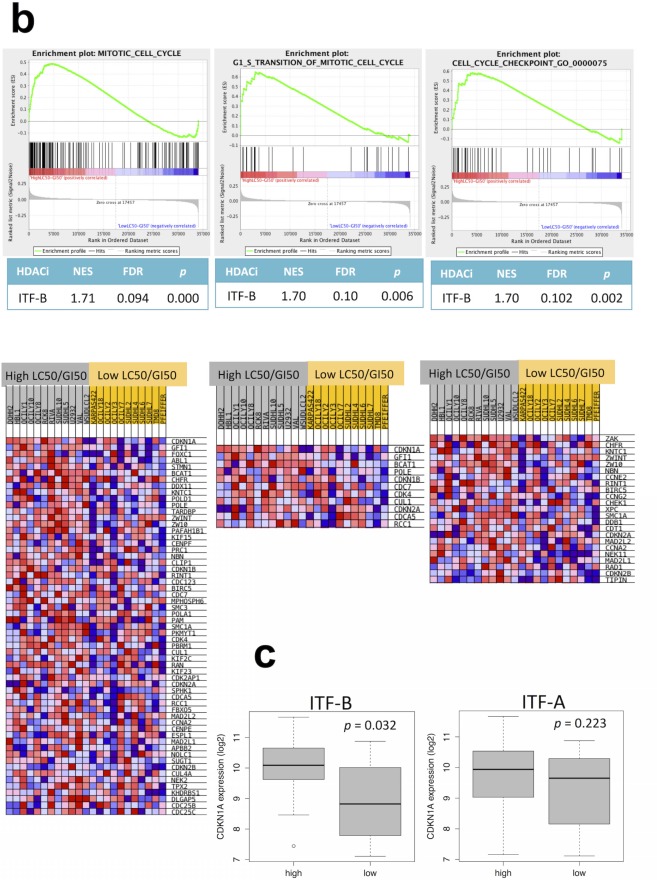
DLBCL cells assigned to low LC50/GI50 and high LC50/GI50 groups exhibit specific genetic signatures (a) The LC50/GI50 value represents the fold difference between LC50 and GI50 values for each cell line. Cell lines with LC50/GI50 values higher than the median are in the high LC50/GI50 group (grey), while those with a lower than the median fold difference are in the low LC50/GI50 group (yellow). LC50/GI50 values were calculated for each HDACi. The heatmap shows the relative expressions of the top 50 up-regulated and the top 50 down-regulated genes. Red denotes high expression, blue denotes low expression. (b) Enrichment plots for cell cycle gene sets that are modulated for ITF-B when comparing high and low LC50/GI50 groups. The heatmaps below the enrichment plots show the core enrichment genes for each gene set. NES = normalized enrichment score, FDR = False discovery rate q-value, *p* = p-value. (c) Higher expression of the *CDKN1A* transcript in the high LC50/GI50 group compared to the low LC50/GI50 group for ITF-B. The thick black line on the boxplot represents the median, while the whiskers represent the interquartile ranges. p-values were obtained using a one-tailed Student's t-test.

As described above, lymphoma cell lines generally underwent G1 cell cycle arrest when treated with HDACi representative of IC50 or GI50 doses. However, some cell lines presented small differences between doses conferring cell cycle arrest (GI50 doses) and those inducing cell death (LC50 doses), while other cell lines presented large differences between these values. We were therefore interested in determining if distinct genetic features characterized these different cell lines. We focused on 23 DLBCL cell lines for which we had drug response data as well as baseline GEP data (e.g., GEP data from cells grown under normal culture conditions, in the absence of HDACi treatment), to prevent histology-specific genetic signatures from masking signatures specifically associated with response. For each of these 23 cell lines, LC50 values were divided by GI50 values to obtain LC50/GI50 values. For each HDACi, the 23 DLBCL cell lines were assigned to one of two groups using the median LC50/GI50 value for each HDACi as a cutoff. For ITF-B, GSEA showed the enrichment of multiple cell cycle associated gene sets in the group of cell lines with high LC50/GI50 values i.e. with LC50/GI50 values greater than the median (Supplementary Table 7 and Figures 3a, 3b). Genes associated with inhibiting cell cycle progression and promoting cell cycle arrest (*CDKN1A, CDKN1B, CDKN2A*) were more highly expressed in the high LC50/GI50 group (Figure 3b). Comparison of CDKN1A levels in high and low LC50/GI50 cell lines showed that CDKN1A was specifically overexpressed in the high LC50/GI50 group (p = 0.032) (Figure 3c). These observations indicate that the high LC50/GI50 and low LC50/GI50 groups comprise cell lines with genetically distinct transcriptomes, which may be important in mediating their response to treatment with HDACi.

We then looked at the association between the anti-proliferation responses of all 32 lymphoma cell lines used in this study and genetic or biological features previously reported as being associated with resistance to HDACi (Supplementary Table 8). The overexpression of anti-apoptotic genes (*BCL2* and *BCL2L1*) has been associated with resistance to HDACi [26]. Expression of *BCL2L1* was positively correlated with IC50 values for ITF-A (r^2^ = 0.490, p-value = 0.006), but not with IC50 values for ITF-B (r^2^ = 0.227, p-value = 0.219). Expression of *BCL2L1* was not associated with LC50 values for either of the two HDACi. *BCL2* expression exhibited no correlation with IC50 or LC50 values. Additionally, when considering only DLBCL cell lines we did not observe any association between *BCL2* translocation status and IC50 values (Supplementary Figure 4). Increased levels of the antioxidant genes *SOD2,* glutathione reductase (*GSR*) and thioredoxin (*TXN*) have also been associated with resistance to HDACi treatment [26]. *SOD2* and *GSR* levels correlated with LC50 values for both HDACi suggesting that they may be specifically associated with the cytotoxic effects of ITF-A and ITF-B. For *SOD2*, the correlation between expression and LC50 values for ITF-B and ITF-A respectively, were r^2^ = 0.569, p-value = 0.001 and r^2^ = 0.542, p-value = 0.002). For *GSR,* the values were r^2^ = 0.444, p-value = 0.013 and r^2^ = 0.419, p-value = 0.020 for ITF-B and ITF-A, respectively (Supplementary Table 8). Overexpression of *STAT1* and *STAT3* transcripts has been reported to reduce sensitivity to some HDACi, but we did not observe an association between dose response values and *STAT1* or *STAT3* expression. Furthermore, as mentioned above, we did not observe any association between dose response values and DLBCL cell of origin, suggesting that the presence of phosphorylated and constitutively activated STAT3 that is exclusively observed in ABC-DLBCL, was unlikely to be associated with response. Inactivating mutations of the histone acetyltransferase genes *EP300* and *CREBBP,* were not associated with response in DLBCL cells (Supplementary Figure 4), which frequently harbor mutations of these genes [3, 4]. At the level of gene expression, *CREBBP* exhibited no correlation with IC50 values for either HDACi, but showed a positive association with LC50 values for ITF-B (r^2^ = 0.431, p-value = 0.016). *EP300* expression negatively correlated with LC50 values for both HDACi (r^2^ = −0.382, p-value = 0.035 for ITF-B and r^2^ = −0.485, p-value = 0.006 for ITF-A).

## DISCUSSION

In this study, we have demonstrated the anti-proliferative activities of two novel HDACi in a panel of 32 lymphoma cell lines. ITF-A and ITF-B are hydroxamic acid HDACi. These novel HDACi target classes I, IIb and IV HDACs. Both HDACi showed the ability to inhibit the proliferation of lymphoma cell lines of different histologies (DLBCL, MCL and SMZL) without any association between potency and lymphoma histology, also when comparing GCB-DLBCL and ABC-DLBCL. For both HDACi, IC50 values were in the nanomolar range with most cell lines displaying IC50 values below the thresholds of 20nM for ITF-A and below 50nM for ITF-B. These values compare favourably with other HDACi currently in the literature [13, 17]. We demonstrated the ability of the two novel HDACi to reverse the deacetylation of histone and non-histone proteins: ITF-A and ITF-B were able to induce the acetylation of histone H3 and α-tubulin in the DLBCL cell lines DoHH2 and TMD8.

In order to better understand the mechanisms of action of the two HDACi, we performed GEP on two DLBCL cell lines that had been treated with each HDACi. We observed the modulation of multiple transcripts and consequently, of numerous signaling pathways and biological processes following the 8 hours treatment. These processes included those regulating cell cycle progression and apoptosis, although these were not the most frequently altered processes. The top 100 up-regulated genes following treatment primarily comprised histone, tubulin and metallothionein genes. Metallothioneins comprised 8/100 (8%) of the top 100 up-regulated genes. Metallothioneins are a class of heavy-metal binding proteins that also scavenge free radicals. HDACi treatment has been shown to up-regulate their expression in certain cell types, and they may be involved in drug resistance [27, 28]. The top 100 down-regulated genes, which included the pro-survival genes *MYC, CCND2* and *NFKB1* likely comprised genes that were targeted for down-regulation following HDACi treatment, probably via mechanisms that specifically reduce the binding of acetylated histones and specific transcription factors to the regulatory regions of these genes [21].

The anti-proliferation response to HDACi can either result from cell cycle arrest or apoptosis [18]. It is not fully clear which factors determine a cytostatic or a cytotoxic response to HDACi although HDACi concentration and cell type are likely to play major roles [18, 29]. Studies investigating the anti-proliferation activities of anti-cancer drugs usually consider the IC50 as a measure of cellular response and drug potency. Determination of additional dose response parameters may provide further information that assists in identifying genetic and/or biological characteristics that influence response [30]. In this study, we also determined GI50, LC50 and TGI values in addition to IC50 values. These measurements enabled us to determine doses at which each HDACi was cytotoxic i.e. the LC50 dose, in each cell line. We observed that at doses representative of the IC50 or GI50, most cell lines tested underwent cell cycle arrest in G1 with little or no apoptosis. Interestingly, cell lines displayed marked differences when comparing their LC50 and GI50 values. Thus, we performed functional annotation analysis comparing cell lines with larger lold differences between their LC50 and GI50 values (high LC50/GI50 group) to those with smaller lold differences between their LC50 and GI50 values (low LC50/GI50 group). We observed that the cell lines belonging to the high LC50/GI50 group were specifically enriched of cell cycle gene sets compared to the low LC50/GI50 group. When looking closer at the single gene level, several genes encoding cyclin dependent kinase inhibitors showed a trend for overexpression in the high LC50/GI50 group and of these *CDKN1A* was more highly expressed in the high LC50/GI50 group than in the low LC50/GI50 group, suggesting that high basal expression of CDKN1A might protect cells from HDACi-mediated cytotoxicity. This observation is important considering the critical role of CDKN1A in the anti-proliferation response to HDACi treatment [11, 13, 18, 31]. The ability of HDACi to increase CDKN1A expression is the main mechanism by which they mediate cell cycle arrest in G1 [11, 13, 18]. However, increased CDKN1A expression can also protect cells from cytotoxicity [19] and indeed, therapies combining HDACi with agents that abrogate HDACi-mediated induction of CDKN1A enhance HDACi-mediated apoptosis [32]. Recently it has been shown that cell lines resistant to another hydroxamic acid derived HDACi, belinostat, exhibit prolonged up-regulation of CDKN1A/p21^WAF1/CIP1^ following treatment with belinostat and do not undergo apoptosis whereas sensitive cell lines only transiently up-regulated CDKN1A/p21^WAF1/CIP1^ and underwent apoptosis [20]. This together with our observations indicates that the baseline level of CDKN1A/p21^WAF1/CIP1^ as well as the duration of its induction following HDACi treatment [20] are important in determining a particular cell's anti-proliferative response to such treatment.

The expression levels of several genes have been shown to impact on the response to HDACi treatment. Among these, overexpression of the anti-apoptotic genes *BCL2* and *BCL2L1* has been reported to confer resistance to HDACi [21, 23, 33]. We did not observe any association between expression levels of *BCL2* and IC50 or LC50 values for the 32 cell lines used in this study. Expression of *BCL2L1* was only positively correlated with IC50 values for ITF-A. Notably, the expression of the antioxidant genes *SOD2* and *GSR* correlated positively with LC50 values for both HDACi. The role of antioxidant genes in conferring resistance to HDACi-induced cytotoxicity has been reported [22, 34] and it is interesting that levels of *SOD2* and *GSR* correlate strongly with LC50 values, suggesting that their higher expression specifically impedes a cytotoxic response.

Recent discoveries of the prevalence of inactivating mutations in the acetyltransferase genes EP300 and CREBBP in lymphomas have highlighted the crucial role of epigenetics in the pathobiology of lymphomas, including especially DLBCL [3, 4]. As HDACi are epigenetic drugs that mediate changes in the acetylation levels of numerous histone and non-histone proteins, it is possible that the presence of mutated EP300 and CREBBP proteins may affect the response to HDACi. However, in DLBCL cells, we observed no correlation between the mutational status of CREBBP or EP300 and the dose response values for ITF-B and ITF-A. We also looked at the expression levels of *CREBBP* and *EP300* transcripts in all 32 cell lines as factors other than mutation or genomic aberrations may reduce the expression of these genes [4]. *CREBBP* expression did not correlate with IC50 values for either HDACi, but positively correlated with LC50 values (i.e., the drug cytotoxic activity) for ITF-B. Conversely, *EP300* levels showed a negative correlation with LC50 values for both HDACi.

In conclusion, we have demonstrated the robust anti-proliferative activity of two novel HDACi, ITF-B and ITF-A. Basal *CDKN1A* levels appear to be important in determining the gap between HDACi concentrations that cause cell cycle arrest and those that cause cell death. The importance of baseline *CDKN1A* levels in mediating cytotoxicity at doses similar to or much higher than the GI50 has not been previously reported. These data may be useful in identifying cells that would benefit from combination approaches using HDACi and agents that overcome cell cycle arrest.

## METHODS

### Cell lines and compounds

Established human cell lines derived from DLBCL, MCL and SMZL were cultured according to the recommended conditions. All media were supplemented with fetal bovine serum (10%), Penicillin-Streptomycin-Neomycin (~5,000 units penicillin, 5 mg streptomycin and 10 mg neomycin/mL, Sigma) and L-glutamine (1%). Two novel HDACi were used: ITF-A and ITF-B (Italfarmaco S.p.A., Milan, Italy).

### Cell proliferation, cell death and cell cycle

Cells were seeded in 96-well plates (non-tissue culture treated) at a density of 10,000 cells for all cell lines except for VAL and JeKo-1, which were seeded at 20,000 cells per well. Each HDACi was dissolved in dimethyl sulphoxide (DMSO). For treatment of cells, compounds were serially diluted in the appropriate tissue culture medium, at a range of 7.8 – 500 nM and added to cells (in five replicates). Cells were incubated for 72 hours at 37ºC, 5% CO_2_. DMSO alone was added to negative control (untreated) cells. Wells containing medium only were included on each plate and served as blanks for absorbance readings. MTT; 3-(4,5-dimethylthiazolyl-2)-2, 5-diphenyltetrazoliumbromide (Sigma-Aldrich Chemie GmbH, Buchs, Switzerland) was prepared as a 5 mg/ml stock in PBS and filter-sterilized. MTT solution (22μL) was added to each well and tissue culture plates were incubated at 37ºC for 4 hours. Cells were then lysed with 25% SDS lysis buffer and absorbance was read at 570 nm using an AD340 plate reader (Beckman Coulter International SA, Switzerland). For each cell line and biological replicate, a “time zero” (t0) plate was seeded with the appropriate number of cells per well and processed immediately to allow a time zero density to be determined. The IC50 (50% Inhibitory Concentration), GI50 (50% Growth Inhibition), LC50 (50% Lethal Concentration: the drug concentration that kills 50% of the cells that were present at the time of drug addition), and TGI (Total Growth Inhibition: the drug concentration that yields no net growth over the course of the assay) were calculated from absorbance values obtained from the treatment plates alone (IC50) or from the treatment plates and time zero plates (GI50, LC50 and TGI). The doses corresponding to the IC50, GI50, LC50 and TGI were estimated by fitting a sigmoidal model through the dose response curve using the R statistical package (www.r-project.org). Cell proliferation, apoptosis and cell cycle were evaluated on cells treated with DMSO or different doses of ITF-B and ITF-A. For cell cycle analyses, the percentages of cells in G1, S and G2/M phases of the cell cycle were determined using the Watson Pragmatic model and the FlowJo software (TreeStar Inc., Ashland, OR, USA).

### Western blotting analysis

Protein extractions, SDS-PAGE and immunoblotting were performed as previously described [35]. The antibodies used were: anti-STAT3 (9139, Cell Signaling), anti-phospho-STAT3 (Tyr705) (9131, Cell Signaling), anti-Acetyl Histone H3 (Lys27) (8173, Cell Signaling), anti-Acetyl--Tubulin (Lys40) (5335, Cell Signaling).

### Immunohistochemistry

Cells were fixed in 2% paraformaldehyde (PFA) and embedded in paraffin. Antigen retrieval was performed with Tris-EDTA at pH 9 for 30 minutes at 98°C. A monoclonal antibody against phospho-STAT3 (Cell Signaling) was applied. Reactions were developed with the Ultravision Quanto Detection System (HRP-Polymer kit (TL-125-QHL, Thermo Scientific).

### Real-time polymerase chain reaction (PCR)

RNA was extracted using TRI-reagent (Sigma-Aldrich Chemie GmbH, Buchs, Switzerland). Quantitative Real-time Polymerase chain reaction (qPCR) was performed as previously described [35] (primer sequences available upon request).

### Gene expression profiling

Gene Expression Profiling (GEP) was performed using the HumanHT-12 v4 Expression BeadChip (Illumina, San Diego, CA, USA), as described [35]. Data processing and statistical analysis were performed using R/Bioconductor [36]. Transcript mapping was based on HG19 using manufacturer-supplied annotation. Data were quantile normalized and subsequently batch corrected using ComBat [37]. Differential expression analysis was performed using LIMMA [38]. Functional annotation was performed using the Gene Set Enrichment Analysis (GSEA) software [39, 40]. Raw GEP data are available on the Gene Expression Omnibus (GEO) database (GSE64821).

## SUPPLEMENTARY MATERIAL FIGURES AND TABLES


















