# Supplementary material for: Novel HDAC inhibitors exhibit pre-clinical efficacy in lymphoma models and point to the importance of CDKN1A expression levels in mediating their anti-tumor response
Source: Oncotarget. 2014 Dec 30;6(7):5059–71. doi: 10.18632/oncotarget.3239 (PMC4467133; doi:10.18632/oncotarget.3239)
Supplement: Supplementary file 1 [file oncotarget-06-5059-s001.pdf]

Novel HDAC inhibitors exhibit pre-clinical efficacy in lymphoma models and point to the importance of *CDKN1A* expression levels in mediating their anti-tumor response

Supplementary Material

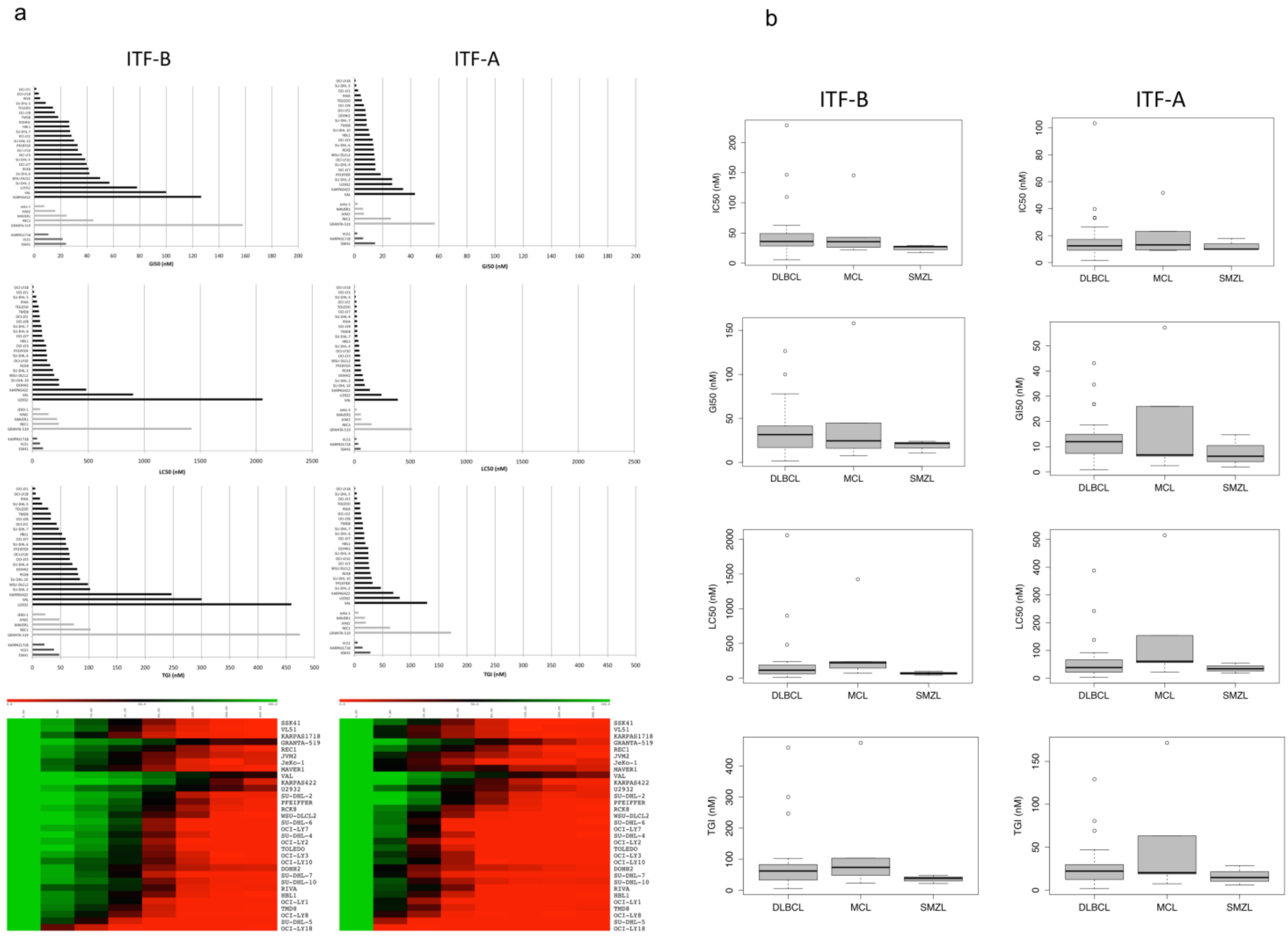

Supplementary Figure 1: The novel histone deacetylase inhibitors ITF-B and ITF-A exhibit antiproliferative activities in a wide range of lymphoma cell lines. (a) Individual GI50, LC50 and TGI values for ITF-B and ITF-A. The 32 cell lines are grouped and coloured according to their lymphoma histology: DLBCL, black bars; MCL, light grey bars; SMZL, dark grey bars. All values are in nM. GI50 values for ITF-B ranged from 1.7 nM to 158.1 nM while those for ITF-A ranged from 0.9 to 57.2 nM. LC50 values range from 10 to 2056.8 nM for ITF-B and from 2.9 to 514.8 nM for ITF-A. TGI values ranged from 5.1 to 474.3 nM for ITF-B, and from 1.6 to 171.6 nM for ITF-A. Bottom panel, color gradient heatmap showing the sensitivity of NHL cell lines to nanomolar doses of either HDACi, as well as the relatively higher sensitivity of cells to ITF-A compared to ITF-B. (green, 100% viable cells; black, 50% viable cells; red, 0% viable cells). (b) Boxplots showing that the dose response parameters (IC50, GI50, LC50 and TGI) are not associated with lymphoma histology. The range and median of values for each dose response parameter are comparable between DLBCL, SMZL and MCL cell lines. The thick black line on each boxplot represents the median, while the whiskers represent the interquartile ranges.

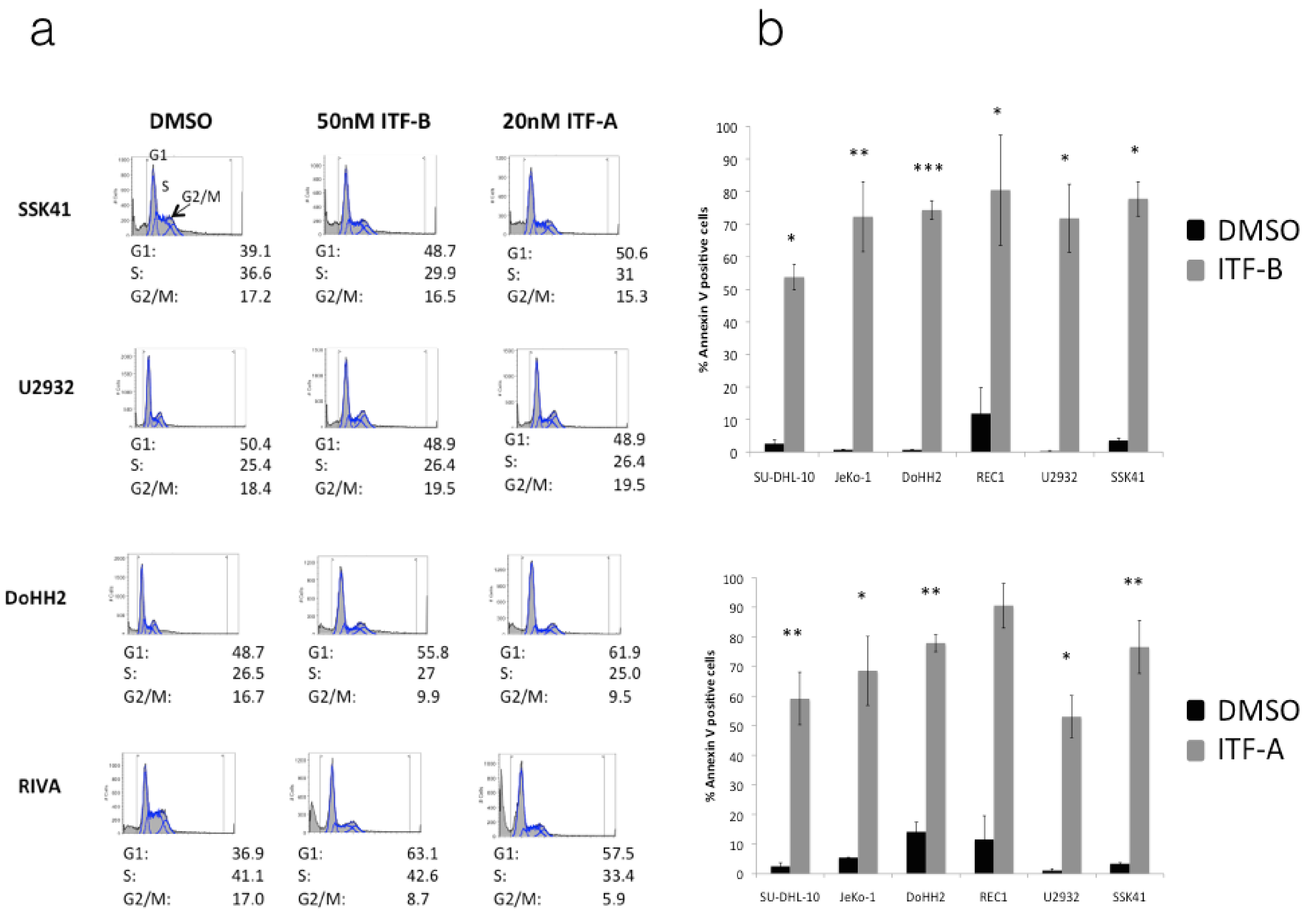

Supplementary Figure 2: Lymphoma cell lines treated with doses representative of the ic50/Gi50, or LC50 doses of ITF-B and ITF-A undergo cell cycle arrest in G1 or apoptosis. (a) Cell cycle analyses of lymphoma cell lines following treatment with 50 nM ITF-B or 20 nM ITF-A for 72 hours. The plots shown are representative of at least two independent experiments. (b) Apoptosis analysis for lymphoma cells treated with LC50 doses of each HDACi. All cell lines undergo pronounced apoptosis. Error bars represent the S.E. (*) denotes $p \leq 0.05$, (**) denotes $p \leq 0.01$, (***) denotes $p \leq 0.001$.

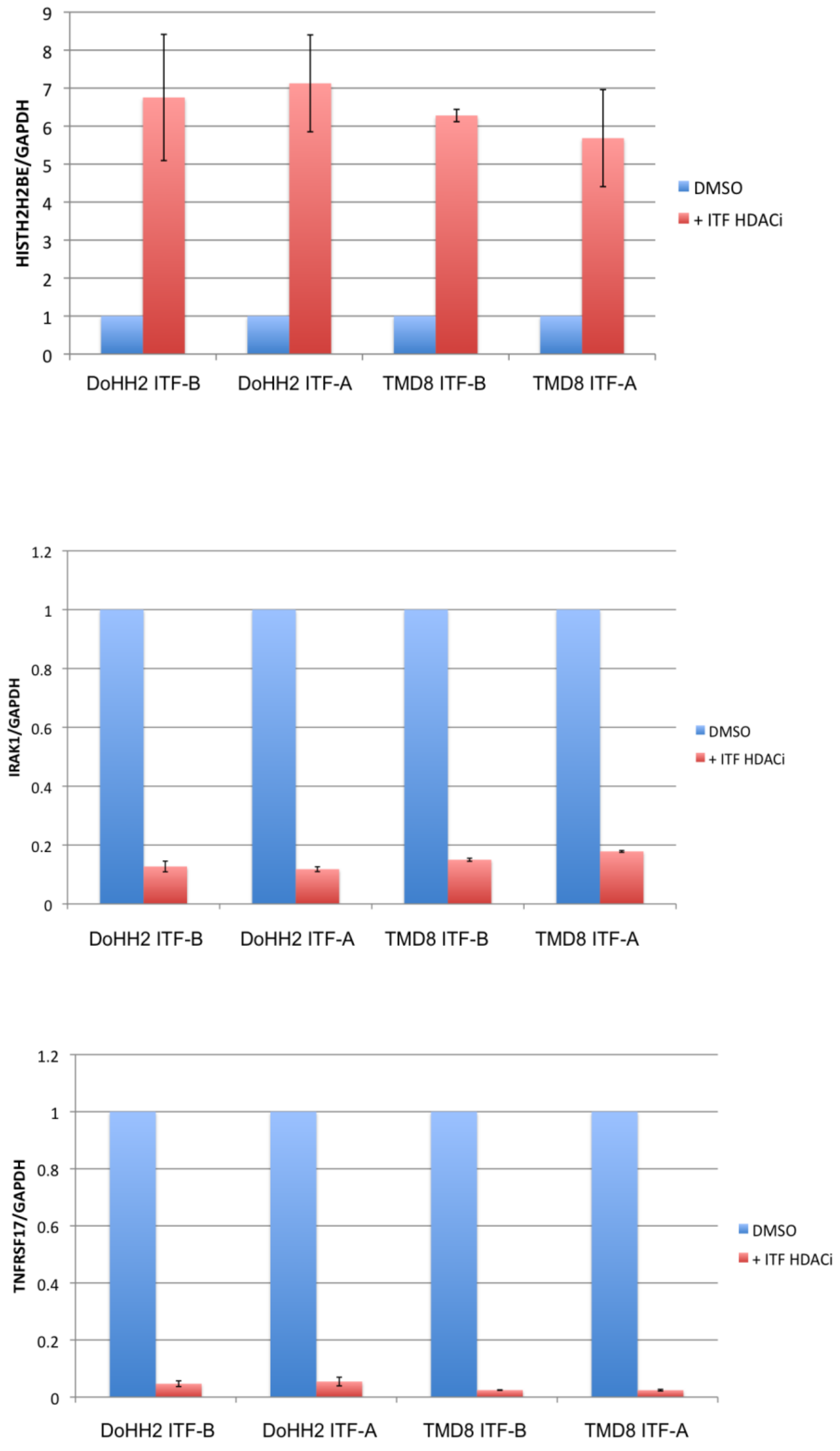

Supplementary Figure 3: Quantitative real-time PCR validation of the modulated expression of *HISTH2BE*, *IRAK1* and *TNFRSF17* transcripts. The expression of each transcript was normalized to *GAPDH*. Error bars represent the S.E.

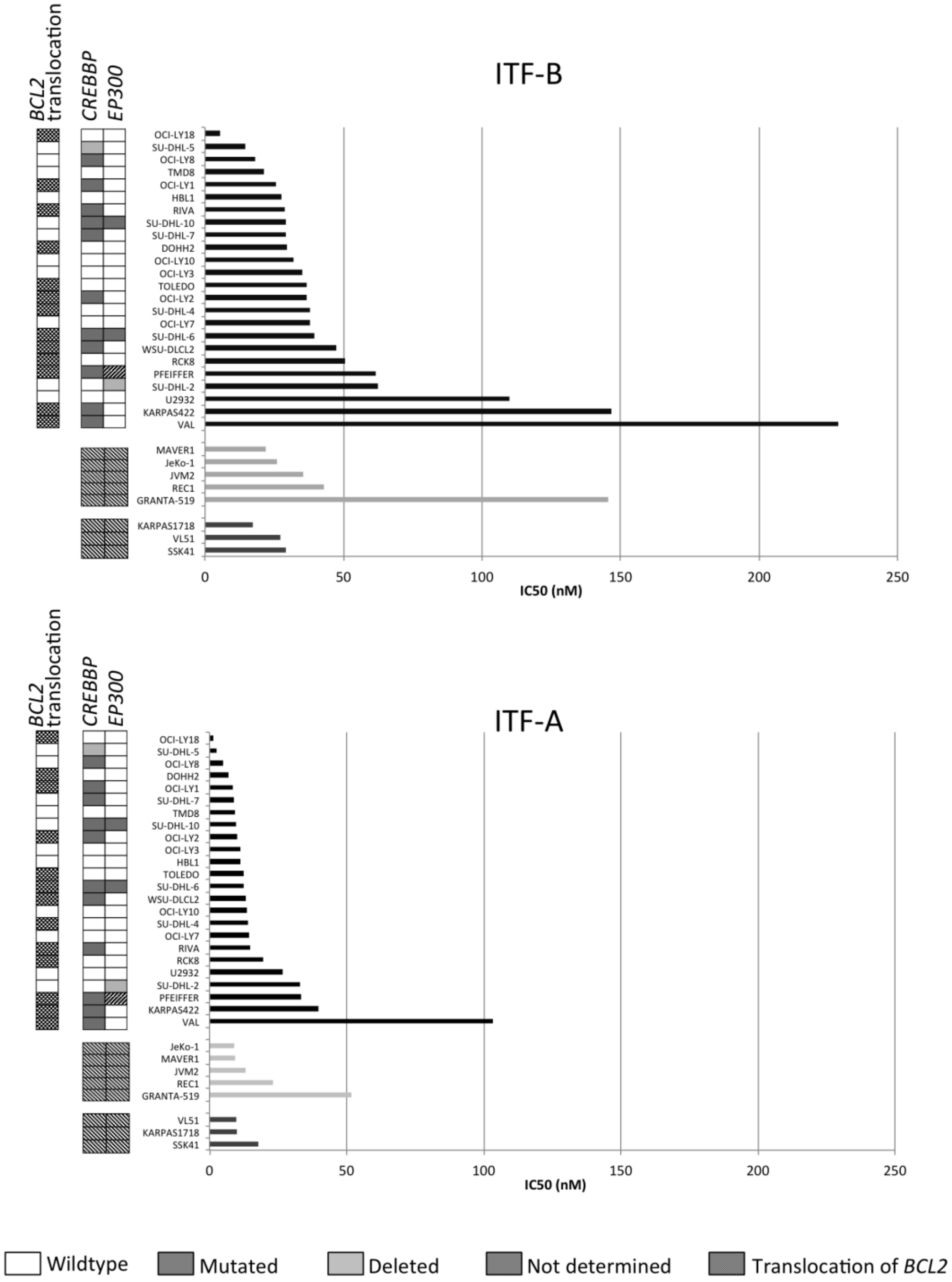

Supplementary Figure 4: BCL2 translocation status and CREBBP/EP300 mutations are not associated with IC50 values. Individual IC50 values for ITF-B (upper panel) and ITF-A (lower panel). The 32 cell lines are grouped and coloured according to their lymphoma histology: DLBCL, black bars; MCL, light grey bars; SMZL, dark grey bars. IC50 values are in nM. To the left of each bar chart are shown the translocation status of BCL2 (for DLBCL cell lines only), the mutational status of CREBBP and the mutational status of EP300. The key for the different genetic aberrations is shown at the bottom. None were associated with IC50 values for the two HDACi.

Supplementary Table 1: Gene ontology (GO) gene sets that were significantly enriched (FDR < 0.25) in DoHH2 (n = 3) cells following 8 hours treatment with 200nM ITF-B and GEP analysis. Column labels: NAME, name of gene set; SIZE, number of members in gene set; NES, normalized enrichment score; ES, enrichment score; NOM p-val, nominal p-value; FDR q-val, false discovery rate q-value.

Supplementary Table 2: Gene ontology (GO) gene sets that were significantly enriched (FDR < 0.25) in DoHH2 (n = 3) cells following 8 hours treatment with 100nM ITF-A and GEP analysis. Column labels: NAME, name of gene set; SIZE, number of members in gene set; NES, normalized enrichment score; ES, enrichment score; NOM p-val, nominal p-value; FDR q-val, false discovery rate q-value.

Supplementary Table 3: Gene ontology (GO) gene sets that were significantly enriched (FDR < 0.25) in TMD8 (n = 3) cells following 8 hours treatment with 200nM ITF-B and GEP analysis. Column labels: NAME, name of gene set; SIZE, number of members in gene set; NES, normalized enrichment score; ES, enrichment score; NOM p-val, nominal p-value; FDR q-val, false discovery rate q-value.

Supplementary Table 4: Gene ontology (GO) gene sets that were significantly enriched (FDR < 0.25) in TMD8 (n = 3) cells following 8 hours treatment with 100nM ITF-A and GEP analysis. Column labels: NAME, name of gene set; SIZE, number of members in gene set; NES, normalized enrichment score; ES, enrichment score; NOM p-val, nominal p-value; FDR q-val, false discovery rate q-value.

Supplementary Table 5: Results of LIMMA analysis of GEP data obtained from DoHH2 (n = 3) and TMD8 (n = 3) cells treated for 8 hours with ITF-B (200 nM). The table lists all probes that were significantly altered between DMSO-treated and HDACi-treated cells. Column labels: PROBE I.D., Illumina probe I.D.; SYMBOL, gene symbol specific for adjacent probe I.D.; log2 ratio, fold change expression between HDACi-treated and DMSO-treated cells. Positive log2 ratios denote increased expression in HDACi-treated cells and negative log2 ratios denote decreased expression in HDACi-treated cells, relative to the DMSO controls. P.Value, p-value; adj.P.Val, p-value adjusted for multiple test correction.

Supplementary Table 6: Results of LIMMA analysis of GEP data obtained from DoHH2 (n = 3) and TMD8 (n = 3) cells treated for 8 hours with ITF-A (100 nM). The table lists all probes that were significantly altered between DMSO-treated and HDACi-treated cells. Column labels: PROBE I.D., Illumina probe I.D.; SYMBOL, gene symbol specific for adjacent probe I.D.; log2 ratio, fold change expression between HDACi-treated and DMSO-treated cells. Positive log2 ratios denote increased expression in HDACi-treated cells and negative log2 ratios denote decreased expression in HDACi-treated cells, relative to the DMSO controls. P.Value, p-value; adj.P.Val, p-value adjusted for multiple test correction.

Supplementary Table 7: Gene ontology (GO) gene sets that were significantly enriched (FDR < 0.25) in high LC50/GI50 and low LC50/GI50 groups for ITF-B. Column labels are as described above for supplementary tables 1-4.

Supplementary Table 8: Spearman correlation coefficients and corresponding p-values for genes whose expression is associated with IC50 or LC50 values for ITF-B or ITF-A.
