# Supplementary material for: Novel HDAC inhibitors exhibit pre-clinical efficacy in lymphoma models and point to the importance of CDKN1A expression levels in mediating their anti-tumor response
Source: Oncotarget. 2014 Dec 30;6(7):5059–71. doi: 10.18632/oncotarget.3239 (PMC4467133; doi:10.18632/oncotarget.3239)
Supplement: Supplementary file 2 [file oncotarget-06-5059-s002.pdf]

Supplementary table 1. DoHH2 DMSO vs DoHH2 ITF-B

NAME	SIZE	ES	NES
GO gene sets with an FDR q-value <0.25 for DoHH2 cells treated with DMSO			
RIBONUCLEOPROTEIN_COMPLEX	142	-0.6683086	-2.11475
NUCLEOLUS	124	-0.63880605	-2.0089557
RNA_PROCESSING	153	-0.61370194	-1.9701163
NUCLEOLAR_PART	18	-0.84737456	-1.8973324
RNA_BINDING	247	-0.54916763	-1.8588868
POSITIVE_REGULATION_OF_TRANSLATION	35	-0.7256097	-1.8058368
RIBOSOME_BIOGENESIS_AND_ASSEMBLY	18	-0.7904628	-1.774277
RNA_SPLICING	74	-0.5884327	-1.7027128
TRANSLATION_FACTOR_ACTIVITY_NUCLEIC_ACID_BINDING	38	-0.6886533	-1.7471045
TRANSLATION_REGULATOR_ACTIVITY	40	-0.6476154	-1.6965946
NUCLEAR_LUMEN	376	-0.48704976	-1.6921141
REGULATION_OF_CYTOKINE_BIOSYNTHETIC_PROCESS	38	-0.6429697	-1.7045639
SPLICEOSOME	50	-0.6349131	-1.7084599
RRNA_PROCESSING	15	-0.7990083	-1.7284105
SMALL_NUCLEAR_RIBONUCLEOPROTEIN_COMPLEX	22	-0.7354578	-1.7111521
INTRACELLULAR_RECEPTOR_MEDIATED_SIGNALING_PATHWAY	20	-0.73194176	-1.6806785
CHROMATIN_REMODELING	25	-0.68813854	-1.6672996
MEMBRANE_ENCLOSED_LUMEN	446	-0.48217404	-1.6838106
TRANSCRIPTION_ACTIVATOR_ACTIVITY	172	-0.5147355	-1.6692865
RIBONUCLEOPROTEIN_COMPLEX_BIOGENESIS_AND_ASSEMBLY	76	-0.6002576	-1.7331848
ORGANELLE_LUMEN	446	-0.48217404	-1.7158227
ESTABLISHMENT_AND_OR_MAINTENANCE_OF_CHROMATIN_ARCHITECTURE	73	-0.57884777	-1.67181
B_CELL_ACTIVATION	20	-0.7344276	-1.6724149
RRNA_METABOLIC_PROCESS	16	-0.78541774	-1.7167335
TRANSLATION_INITIATION_FACTOR_ACTIVITY	24	-0.69705003	-1.6521269
TRANSCRIPTION_COACTIVATOR_ACTIVITY	123	-0.53115624	-1.653349
STEROID_HORMONE_RECEPTOR_SIGNALING_PATHWAY	19	-0.7370688	-1.6411257
CALCIUM_MEDIATED_SIGNALING	16	-0.7638546	-1.6381007
POSITIVE_REGULATION_OF_CYTOKINE_BIOSYNTHETIC_PROCESS	25	-0.68445295	-1.6274549
CHROMATIN_ASSEMBLY_OR_DISASSEMBLY	26	-0.6865081	-1.6280227
CHROMOSOME_ORGANIZATION_AND_BIOGENESIS	118	-0.5288872	-1.613346
CYTOKINE_METABOLIC_PROCESS	42	-0.60400826	-1.6043673
TRNA_METABOLIC_PROCESS	18	-0.7051526	-1.5796515
VITAMIN_METABOLIC_PROCESS	17	-0.7163434	-1.569271
CHROMATIN_MODIFICATION	51	-0.560716	-1.5623714
CYTOKINE_BIOSYNTHETIC_PROCESS	41	-0.606363	-1.5693831
RIBOSOMAL_SUBUNIT	20	-0.678977	-1.5575495
MRNA_PROCESSING_GO_0006397	55	-0.56759304	-1.5627192
BASE_EXCISION_REPAIR	16	-0.72263724	-1.5518343
HELICASE_ACTIVITY	50	-0.56216437	-1.549304
TRANSCRIPTION_INITIATION	35	-0.60801667	-1.5539697
REGULATION_OF_TRANSLATION	93	-0.5057617	-1.5311645
TRANSLATIONAL_INITIATION	39	-0.5916824	-1.5407702
ORGANELLAR_RIBOSOME	22	-0.65810055	-1.5313628
PROTEIN_DNA_COMPLEX_ASSEMBLY	48	-0.5671293	-1.5420848
INSOLUBLE_FRACTION	15	-0.72848374	-1.5360746
REGULATION_OF_TRANSLATIONAL_INITIATION	31	-0.59900224	-1.5313994
HORMONE_RECEPTOR_BINDING	29	-0.6209668	-1.5244161
TRANSLATION	178	-0.47049975	-1.532691
TRANSCRIPTION_INITIATION_FROM_RNA_POLYMERASE_II_PROMOTER	29	-0.6118413	-1.5163132
MITOCHONDRIAL_RIBOSOME	22	-0.65810055	-1.5172762
MACROMOLECULAR_COMPLEX_DISASSEMBLY	15	-0.70423734	-1.5069184
RIBOSOME	39	-0.58313733	-1.5047755
PROTEASOME_COMPLEX	23	-0.6490232	-1.5011836
REGULATION_OF_T_CELL_PROLIFERATION	16	-0.69474155	-1.4848404
T_CELL_PROLIFERATION	19	-0.66152847	-1.4817278
PROTEIN_FOLDING	57	-0.531674	-1.4853814
PROTEIN_RNA_COMPLEX_ASSEMBLY	57	-0.5234774	-1.4863
INDUCTION_OF_APOPTOSIS_BY_INTRACELLULAR_SIGNALS	23	-0.63355714	-1.4875244
DOUBLE_STRANDED_DNA_BINDING	32	-0.59530115	-1.4886168
NUCLEOPLASM	270	-0.43690157	-1.4771794
MACROMOLECULAR_COMPLEX_ASSEMBLY	269	-0.43104842	-1.4939547
NUCLEAR_HORMONE_RECEPTOR_BINDING	28	-0.615708	-1.4919788
STRUCTURE_SPECIFIC_DNA_BINDING	55	-0.537628	-1.4891741
MITOCHONDRION_ORGANIZATION_AND_BIOGENESIS	47	-0.5446293	-1.46452
RNA_DEPENDENT_ATPASE_ACTIVITY	17	-0.6702436	-1.457006
PORE_COMPLEX	36	-0.57562625	-1.4551884
TRANSCRIPTION_COFACTOR_ACTIVITY	223	-0.43784958	-1.4512144
REGULATION_OF_DNA_METABOLIC_PROCESS	44	-0.54886025	-1.4415286
ATP_DEPENDENT_RNA_HELICASE_ACTIVITY	16	-0.649511	-1.4366455
DNA_METABOLIC_PROCESS	245	-0.4278727	-1.4294144
CHROMATIN_BINDING	32	-0.56701773	-1.4312412
RIBONUCLEASE_ACTIVITY	25	-0.60394996	-1.4266405
NUCLEAR_PORE	31	-0.57249576	-1.431324

CHROMOSOMEPERICENTRIC_REGION	31	-0.5654424	-1.4327272
CHROMATIN_REMODELING_COMPLEX	17	-0.64763415	-1.4221178
CELLULAR_COMPONENT_ASSEMBLY	286	-0.41786787	-1.4153849
ATP_DEPENDENT_HELICASE_ACTIVITY	26	-0.5899623	-1.4109006
HUMORAL_IMMUNE_RESPONSE	32	-0.5670373	-1.4109234
GO gene sets with an FDR q-value <0.25 for DoHH2 cells treated with 200nM ITF-B			
MICROTUBULE_ASSOCIATED_COMPLEX	47	0.7265401	1.8540069
ENDOSOME_TRANSPORT	23	0.8006768	1.8357769
VESICLE_MEDIATED_TRANSPORT	193	0.5898236	1.8599873
HYDRO_LYASE_ACTIVITY	27	0.75701255	1.7794979
CYTOSOL	205	0.5452738	1.7316146
CLATHRIN_COATED_VESICLE	36	0.6856709	1.6740823
GOLGI_MEMBRANE	44	0.6540872	1.6774311
MOTOR_ACTIVITY	28	0.7153378	1.6799856
GOLGI_APPARATUS	222	0.5269615	1.6834804
REGULATION_OF_G_PROTEIN_COUPLED_RECEPTOR_PROTEIN_SIGNALING_PATHWAY	23	0.7763036	1.7349573
SECRETION	178	0.54328346	1.6915967
CYTOSKELETAL_PROTEIN_BINDING	158	0.5475612	1.6842606
MICROTUBULE_CYTOSKELETON	145	0.54678327	1.6580878
LIPID_TRANSPORTER_ACTIVITY	28	0.72481203	1.7023879
OXIDOREDUCTASE_ACTIVITY_ACTING_ON_THE_ALDEHYDE_OR_OXO_GROUP_OF_DONORS	22	0.7566588	1.695187
LIPID_TRANSPORT	28	0.7276045	1.7090336
LIPID_HOMEOSTASIS	16	0.7774584	1.6398264
NITROGEN_COMPOUND_CATABOLIC_PROCESS	29	0.69091594	1.6420951
REGULATION_OF_NEUROTRANSMITTER_LEVELS	24	0.7038678	1.6099482
GLUTATHIONE_TRANSFERASE_ACTIVITY	15	0.7443736	1.551416
SECRETION_BY_CELL	116	0.5449789	1.6099759
LYASE_ACTIVITY	69	0.5910774	1.613995
ACTIN_FILAMENT_BASED_PROCESS	115	0.5219311	1.5519682
FATTY_ACID_OXIDATION	18	0.7169639	1.5522623
MONOVALENT_INORGANIC_CATION_TRANSPORT	93	0.5329851	1.5396085
AMINE_CATABOLIC_PROCESS	27	0.6791309	1.5990794
RESPONSE_TO_NUTRIENT_LEVELS	29	0.641945	1.5410624
RESPONSE_TO_NUTRIENT	17	0.7573103	1.5953596
CYTOPLASM_ORGANIZATION_AND_BIOGENESIS	15	0.7588257	1.5553219
PROTEIN_KINASE_ACTIVITY	281	0.47170925	1.5524039
PHOSPHOLIPID_BINDING	47	0.59809124	1.5603452
AMINO_ACID_CATABOLIC_PROCESS	25	0.6722415	1.5422083
MEMBRANE_ORGANIZATION_AND_BIOGENESIS	133	0.51030964	1.5347729
MICROTUBULE_MOTOR_ACTIVITY	16	0.724689	1.5573968
DEVELOPMENTAL_MATURATION	18	0.7089525	1.5321097
PROTEIN_KINASE_REGULATOR_ACTIVITY	39	0.6026566	1.5266964
ENZYME_LINKED_RECEPTOR_PROTEIN_SIGNALING_PATHWAY	140	0.515702	1.5673956
VACUOLE	68	0.5715181	1.564358
NEURON_DEVELOPMENT	61	0.57224005	1.5757174
GOLGI_APPARATUS_PART	98	0.53980386	1.5604517
TIGHT_JUNCTION	31	0.6239602	1.5076591
CYTOSKELETON_ORGANIZATION_AND_BIOGENESIS	207	0.4733538	1.5005836
HYDROLASE_ACTIVITY_HYDROLYZING_O_GLYCOSYL_COMPOUNDS	37	0.64234656	1.5781364
CARBON_CARBON_LYASE_ACTIVITY	18	0.7210119	1.5270947
CARBON_OXYGEN_LYASE_ACTIVITY	31	0.6444252	1.5690886
DETECTION_OF_EXTERNAL_STIMULUS	23	0.6693147	1.5202872
METAL_ION_TRANSPORT	117	0.50993246	1.5087047
APICAL_JUNCTION_COMPLEX	34	0.60649353	1.5139655
SECRETORY_PATHWAY	84	0.5568588	1.5711442
CALCIUM_INDEPENDENT_CELL_CELL_ADHESION	22	0.6712066	1.5009648
CYTOSKELETAL_PART	230	0.47927865	1.5204204
T_CELL_DIFFERENTIATION	15	0.7288376	1.5145875
LIPOPROTEIN_BINDING	17	0.7077341	1.516423
CYTOSKELETON	357	0.45510924	1.5107311
CELL_MATURATION	16	0.7246372	1.5088812
HETEROCYCLE_METABOLIC_PROCESS	27	0.6776636	1.5790309
TRANSMEMBRANE_RECEPTOR_PROTEIN_TYROSINE_KINASE_SIGNALING_PATHWAY	83	0.53472245	1.5013542
CATION_TRANSPORT	146	0.5162964	1.5858904
VESICLE	121	0.49735412	1.4914323
RESPONSE_TO_EXTRACELLULAR_STIMULUS	33	0.6108238	1.5016779
OXIDOREDUCTASE_ACTIVITY_ACTING_ON_THE_ALDEHYDE_OR_OXO_GROUP_OF_DONORSNAD_OR_NADP_AS_ACCEPTOR	16	0.7721069	1.5808773
GTPASE_ACTIVITY	99	0.5181239	1.4939619
PROTEIN_SERINE_THREONINE_KINASE_ACTIVITY	202	0.4713966	1.4919822
GOLGI_VESICLE_TRANSPORT	48	0.57057106	1.4862974
REGULATION_OF_HYDROLASE_ACTIVITY	78	0.51987463	1.4741195
LIPID_BINDING	87	0.5122723	1.4722123
SYNAPSE	27	0.628908	1.4745849
CYTOPLASMIC_VESICLE	116	0.49996084	1.4819608
TUBULIN_BINDING	46	0.57167953	1.4749355
MEMBRANE_BOUND_VESICLE	114	0.49543852	1.4636265
PEROXISOME	45	0.5587444	1.4587991

APICOLATERAL_PLASMA_MEMBRANE	34	0.60649353	1.4775307
ENDOSOME	66	0.54419833	1.4755374
ION_TRANSPORT	184	0.46622548	1.4552613
ACTIN_CYTOSKELETON_ORGANIZATION_AND_BIOGENESIS	105	0.49855614	1.4638475
CELL_PROJECTION_BIOGENESIS	25	0.62143964	1.4591367
SMALL_GTPASE_BINDING	33	0.5939568	1.4533591
PHOSPHOTRANSFERASE_ACTIVITY_ALCOHOL_GROUP_AS_ACCEPTOR	330	0.44699347	1.4782015
ION_BINDING	267	0.44617248	1.4516926
NERVOUS_SYSTEM_DEVELOPMENT	381	0.4361527	1.455871
LAMELLIPODIUM	25	0.618594	1.4499168
OXIDOREDUCTASE_ACTIVITY_ACTING_ON_THE_CH_CH_GROUP_OF_DONORS	23	0.65313095	1.4659601
CARBOHYDRATE_TRANSPORT	19	0.6557965	1.4643732
CELL_PROJECTION	107	0.49649185	1.4594647
MICROBODY	45	0.55874443	1.4421461
CELLULAR_LIPID_CATABOLIC_PROCESS	35	0.5675329	1.4328537
G_PROTEIN_COUPLED_RECEPTOR_BINDING	54	0.5374589	1.4446687
KINASE_ACTIVITY	364	0.43430722	1.4460144
MYOSIN_COMPLEX	16	0.6879892	1.4430586
GTPASE_BINDING	34	0.58149636	1.433647
REGULATION_OF_CATALYTIC_ACTIVITY	273	0.440413	1.4297359
LIPID_CATABOLIC_PROCESS	38	0.55699223	1.3983587
CYTOPLASMIC_MEMBRANE_BOUND_VESICLE	112	0.49197596	1.4358207
REGULATION_OF_CELL_GROWTH	43	0.5611896	1.439537
CARBOHYDRATE_METABOLIC_PROCESS	180	0.45648935	1.430004
REGULATION_OF_ANATOMICAL_STRUCTURE_MORPHOGENESIS	25	0.62081057	1.4378728
SYNAPTIC_VESICLE	15	0.689496	1.4339148
COATED_VESICLE	46	0.55043757	1.398433
CASPASE_ACTIVATION	26	0.60508895	1.3950838
EXTRACELLULAR_STRUCTURE_ORGANIZATION_AND_BIOGENESIS	32	0.57296944	1.3961947
LIPID_METABOLIC_PROCESS	318	0.43726063	1.4363127
