# Supplementary material for: Novel HDAC inhibitors exhibit pre-clinical efficacy in lymphoma models and point to the importance of CDKN1A expression levels in mediating their anti-tumor response
Source: Oncotarget. 2014 Dec 30;6(7):5059–71. doi: 10.18632/oncotarget.3239 (PMC4467133; doi:10.18632/oncotarget.3239)
Supplement: Supplementary file 3 [file oncotarget-06-5059-s003.pdf]

Supplementary table 2. DoHH2 DMSO vs DoHH2 ITF-A

NAME	SIZE	ES	NES	NOM p-val	FDR q-val
GO gene sets with an FDR q-value <0.25 for DoHH2 cells treated with DMSO					
RNA_PROCESSING	153	-0.63482404	-2.0205438	0	9.69E-04
RRNA_METABOLIC_PROCESS	16	-0.8634268	-1.9031278	0	0.004907108
RRNA_PROCESSING	15	-0.8638641	-1.9084433	0	0.00542658
RIBOSOME_BIOGENESIS_AND_ASSEMBLY	18	-0.84540546	-1.8570218	0	0.009537009
RNA_SPLICING	74	-0.61536133	-1.8149229	0	0.015170613
RIBONUCLEOPROTEIN_COMPLEX_BIOGENESIS_AND_ASSEMBLY	76	-0.6141472	-1.793436	0	0.018158648
POSITIVE_REGULATION_OF_TRANSLATION	35	-0.67217606	-1.723561	0.002272727	0.059082158
INTRACELLULAR_RECEPTOR_MEDIATED_SIGNALING_PATHWAY	20	-0.7304811	-1.6436745	0.004264392	0.11697672
MRNA_PROCESSING_GO_0006397	55	-0.5936987	-1.6477762	0	0.12119681
STEROID_HORMONE_RECEPTOR_SIGNALING_PATHWAY	19	-0.7371631	-1.6492529	0.002217295	0.13175921
CALCIUM_MEDIATED_SIGNALING	16	-0.7562959	-1.6566162	0.006479482	0.13612059
POSITIVE_REGULATION_OF_CYTOKINE_BIOSYNTHETIC_PROCESS	25	-0.67000633	-1.5976083	0.01632653	0.17810297
VITAMIN_METABOLIC_PROCESS	17	-0.70854366	-1.5711032	0.010548524	0.20375498
TRANSCRIPTION_INITIATION	35	-0.61133456	-1.5760583	0.019148936	0.20777315
TRNA_METABOLIC_PROCESS	18	-0.691187	-1.5385395	0.02892562	0.20857576
TRANSCRIPTION_INITIATION_FROM_RNA_POLYMERASE_II_PROMOTER	29	-0.6162327	-1.5320098	0.025052192	0.2102346
ESTABLISHMENT_AND_OR_MAINTENANCE_OF_CHROMATIN_ARCHITECTURE	73	-0.53335124	-1.5407854	0	0.21567933
MITOCHONDRION_ORGANIZATION_AND_BIOGENESIS	47	-0.56922704	-1.5437455	0.015521064	0.22175695
REGULATION_OF_CYTOKINE_BIOSYNTHETIC_PROCESS	38	-0.5910318	-1.5189065	0.022123894	0.22666678
TRANSLATION	178	-0.47377467	-1.5518727	0.002590674	0.23100092
CHROMOSOME_ORGANIZATION_AND_BIOGENESIS	118	-0.4969925	-1.5456069	0.002403846	0.2317634
INDUCTION_OF_APOPTOSIS_BY_INTRACELLULAR_SIGNALS	23	-0.63246936	-1.4867721	0.027459955	0.23534188
PROTEIN_DNA_COMPLEX_ASSEMBLY	48	-0.53919613	-1.4622419	0.022522522	0.23539223
VIRAL_INFECTIOUS_CYCLE	32	-0.57502425	-1.4585178	0.03524229	0.23670973
REGULATION_OF_DNA_METABOLIC_PROCESS	44	-0.5525173	-1.4894961	0.035794184	0.23901428
TRANSLATIONAL_INITIATION	39	-0.5780719	-1.5032567	0.019438446	0.23928149
PROTEIN_RNA_COMPLEX_ASSEMBLY	57	-0.53092694	-1.4675852	0.013824885	0.23951559
CHROMATIN_ASSEMBLY_OR_DISASSEMBLY	26	-0.6186254	-1.4635402	0.057017542	0.24056216
CYTOKINE_BIOSYNTHETIC_PROCESS	41	-0.56763	-1.4712147	0.025751073	0.24079245
CHROMATIN_REMODELING	25	-0.629941	-1.5064597	0.035639413	0.24266666
B_CELL_ACTIVATION	20	-0.64798653	-1.4745712	0.044843048	0.24299717
MESODERM_DEVELOPMENT	22	-0.6206894	-1.4465055	0.05172414	0.2453747
DNA_METABOLIC_PROCESS	245	-0.43535963	-1.4762784	0	0.24808535
CYTOKINE_METABOLIC_PROCESS	42	-0.5668394	-1.489854	0.008888889	0.24819064
GO gene sets with an FDR q-value <0.25 for DoHH2 cells treated with 100nM ITF3352					
ENDOSOME_TRANSPORT	23	0.80766124	1.87209	0	0.006132597
REGULATION_OF_G_PROTEIN_COUPLED_RECEPTOR_PROTEIN_SIGNALING_PATHWAY	23	0.81443197	1.8796303	0	0.008186678
VESICLE_MEDIATED_TRANSPORT	193	0.5806581	1.8132256	0	0.022516789
LIPID_TRANSPORT	28	0.7370543	1.766543	0.001862197	0.0468874
CATION_TRANSPORT	146	0.54944146	1.6789579	0	0.1527188
CYTOPLASM_ORGANIZATION_AND_BIOGENESIS	15	0.77432734	1.6360766	0.015065913	0.16468285
SECRETION	178	0.5326729	1.6426193	0	0.16874748
RESPONSE_TO_NUTRIENT	17	0.74473906	1.6228224	0.012727273	0.17595486
ENZYME_LINKED_RECEPTOR_PROTEIN_SIGNALING_PATHWAY	140	0.5489901	1.6451019	0	0.18722029
NITROGEN_COMPOUND_CATABOLIC_PROCESS	29	0.6745041	1.6076156	0.007352941	0.19411509
LIPID_HOMEOSTASIS	16	0.7668896	1.6003537	0.01984127	0.19501562
MONOVALENT_INORGANIC_CATION_TRANSPORT	93	0.57558674	1.6461823	0	0.21602628
CARBOHYDRATE_METABOLIC_PROCESS	180	0.46893248	1.4681245	0.001669449	0.22263394
ACTIN_CYTOSKELETON_ORGANIZATION_AND_BIOGENESIS	105	0.5110678	1.4691807	0.005199307	0.22575782
MAPKKK_CASCADE_GO_0000165	103	0.5078206	1.469908	0.015358361	0.22988807
ACTIN_FILAMENT_BASED_PROCESS	115	0.5274642	1.560652	0.00170068	0.23262605
AMINO_ACID_CATABOLIC_PROCESS	25	0.6393488	1.4709973	0.048780486	0.23335254
CELL_MATRIX_ADHESION	38	0.59950197	1.4757731	0.036900368	0.2340043
DETECTION_OF_STIMULUS	47	0.5618121	1.4787682	0.010928961	0.2344782
CYTOSKELETON_DEPENDENT_INTRACELLULAR_TRANSPORT	26	0.6296058	1.4723301	0.042435423	0.23620903
DEVELOPMENTAL_MATURATION	18	0.68588	1.4900123	0.034951456	0.237324
METAL_ION_TRANSPORT	117	0.52669436	1.5530785	0	0.23738036
TRANSMEMBRANE_RECEPTOR_PROTEIN_TYROSINE_KINASE_SIGNALING_PATHWAY	83	0.5605245	1.5637133	0.005514706	0.2397919
CELL_SUBSTRATE_ADHESION	39	0.5968138	1.4825708	0.030909091	0.24019367
T_CELL_DIFFERENTIATION	15	0.712333	1.4787927	0.04283054	0.24111971
CELL_PROJECTION_BIOGENESIS	25	0.6418874	1.5097239	0.037664782	0.2428778
CALCIUM_INDEPENDENT_CELL_CELL_ADHESION	22	0.643438	1.4838004	0.025316456	0.2445762
PHOSPHOINOSITIDE_METABOLIC_PROCESS	31	0.6229605	1.4900626	0.031481482	0.24481401
SECRETION_BY_CELL	116	0.5285298	1.5678555	0.00661157	0.24584353
CELL_MATURATION	16	0.7074772	1.5122068	0.035714287	0.2465322
NEGATIVE_REGULATION_OF_MAP_KINASE_ACTIVITY	17	0.71107185	1.515123	0.030487806	0.24986666
RESPONSE_TO_NUTRIENT_LEVELS	29	0.63750136	1.5191718	0.026455026	0.24995592
