# Supplementary material for: Novel HDAC inhibitors exhibit pre-clinical efficacy in lymphoma models and point to the importance of CDKN1A expression levels in mediating their anti-tumor response
Source: Oncotarget. 2014 Dec 30;6(7):5059–71. doi: 10.18632/oncotarget.3239 (PMC4467133; doi:10.18632/oncotarget.3239)
Supplement: Supplementary file 4 [file oncotarget-06-5059-s004.pdf]

Supplementary table 3. TMD8 DMSO vs TMD8 ITF-B

NAME	SIZE	ES	NES
GO gene sets with an FDR q-value <0.25 for TMD8 cells treated with DMSO			
POSITIVE_REGULATION_OF_TRANSLATION	35	-0.7988508	-2.028977
HUMORAL_IMMUNE_RESPONSE	32	-0.807858	-1.9685322
REGULATION_OF_CYTOKINE_BIOSYNTHETIC_PROCESS	38	-0.74022186	-1.9022937
MESODERM_DEVELOPMENT	22	-0.8233811	-1.8961663
CYTOKINE_BIOSYNTHETIC_PROCESS	41	-0.7399187	-1.8876904
CYTOKINE_METABOLIC_PROCESS	42	-0.7385127	-1.9096785
RESPONSE_TO_VIRUS	49	-0.69948876	-1.8811599
RESPONSE_TO_OTHER_ORGANISM	82	-0.65528023	-1.9200163
POSITIVE_REGULATION_OF_CYTOKINE_BIOSYNTHETIC_PROCESS	25	-0.78198856	-1.8080004
RIBONUCLEOPROTEIN_COMPLEX	142	-0.57118034	-1.7997347
REGULATION_OF_I_KAPPAB_KINASE_NF_KAPPAB_CASCADE	90	-0.61013377	-1.778009
POSITIVE_REGULATION_OF_I_KAPPAB_KINASE_NF_KAPPAB_CASCADE	84	-0.6014649	-1.751625
POSITIVE_REGULATION_OF_JNK_ACTIVITY	18	-0.7961333	-1.757035
ACTIVATION_OF_JNK_ACTIVITY	16	-0.79802877	-1.6915996
TRANSCRIPTION_ACTIVATOR_ACTIVITY	172	-0.53510994	-1.7064543
I_KAPPAB_KINASE_NF_KAPPAB_CASCADE	111	-0.5692791	-1.6821969
RNA_PROCESSING	153	-0.5348341	-1.6957581
INDUCTION_OF_APOPTOSIS_BY_INTRACELLULAR_SIGNALS	23	-0.7269506	-1.6844964
REGULATION_OF_JNK_ACTIVITY	20	-0.7618196	-1.6923356
IMMUNE_RESPONSE	234	-0.5151259	-1.6983012
IMMUNE_SYSTEM_PROCESS	327	-0.49120587	-1.6619964
STRUCTURE_SPECIFIC_DNA_BINDING	55	-0.6104479	-1.6467421
NUCLEOLUS	124	-0.5423444	-1.6493324
RESPONSE_TO_BIOTIC_STIMULUS	119	-0.5335225	-1.6258438
NUCLEOLAR_PART	18	-0.73399794	-1.6099895
CYTOKINE_AND_CHEMOKINE_MEDIATED_SIGNALING_PATHWAY	22	-0.72647846	-1.6162909
POSITIVE_REGULATION_OF_SIGNAL_TRANSDUCTION	123	-0.52896345	-1.6118376
REGULATION_OF_T_CELL_ACTIVATION	28	-0.6789743	-1.5929914
PROTEIN_AMINO_ACID_DEPHOSPHORYLATION	63	-0.57290566	-1.5816338
POSITIVE_REGULATION_OF_CELLULAR_PROTEIN_METABOLIC_PROCESS	73	-0.5420779	-1.5749317
RIBOSOME_BIOGENESIS_AND_ASSEMBLY	18	-0.7162312	-1.5777783
TRANSCRIPTION_COACTIVATOR_ACTIVITY	123	-0.5184093	-1.5866828
RRNA_PROCESSING	15	-0.748584	-1.5715295
RRNA_METABOLIC_PROCESS	16	-0.7467341	-1.5820082
B_CELL_ACTIVATION	20	-0.6928285	-1.549143
REGULATION_OF_TRANSLATION	93	-0.5228688	-1.5461768
DEPHOSPHORYLATION	70	-0.54358625	-1.5504819
MEMBRANE_ENCLOSED_LUMEN	446	-0.4456989	-1.540395
CYTOKINE_PRODUCTION	73	-0.54875064	-1.5522257
ORGANELLE_LUMEN	446	-0.4456989	-1.5414231
MITOCHONDRIAL_RIBOSOME	22	-0.66162497	-1.5206051
CYTOKINE_ACTIVITY	111	-0.5114468	-1.5313213
REGULATION_OF_LYMPHOCYTE_ACTIVATION	35	-0.6055355	-1.5076615
TRANSCRIPTION_INITIATION_FROM_RNA_POLYMERASE_II_PROMOTER	29	-0.6258204	-1.5208344
DOUBLE_STRANDED_DNA_BINDING	32	-0.61582434	-1.5045879
NUCLEAR_LUMEN	376	-0.44107887	-1.508633
CALCIUM_MEDIATED_SIGNALING	16	-0.7135546	-1.5210056
SINGLE_STRANDED_DNA_BINDING	34	-0.60526276	-1.5090548
RNA_BINDING	247	-0.46208572	-1.5211779
RECEPTOR_SIGNALING_PROTEIN_ACTIVITY	81	-0.52012295	-1.5093406
CHEMOKINE_RECEPTOR_BINDING	43	-0.5857955	-1.4973732
MACROMOLECULAR_COMPLEX_DISASSEMBLY	15	-0.7125339	-1.4932573
VIRAL_GENOME_REPLICATION	21	-0.66401374	-1.5109493
POSITIVE_REGULATION_OF_PROTEIN_METABOLIC_PROCESS	75	-0.53479725	-1.5226486
DEFENSE_RESPONSE	267	-0.44906944	-1.4902235
CHEMOKINE_ACTIVITY	42	-0.5860933	-1.4935734
MOLECULAR_ADAPTOR_ACTIVITY	49	-0.5503586	-1.4847975
ORGANELLAR_RIBOSOME	22	-0.66162497	-1.4746549
SPLICEOSOME	50	-0.53618056	-1.4507927
RNA_SPLICING	74	-0.5152229	-1.4716372
TRANSLATION	178	-0.45839664	-1.4555454
REGULATION_OF_CELLULAR_PROTEIN_METABOLIC_PROCESS	162	-0.4560066	-1.4461854
NEURON_APOPTOSIS	17	-0.6890734	-1.4608828
NEGATIVE_REGULATION_OF_TRANSCRIPTION	181	-0.46049848	-1.4624113
MULTI_ORGANISM_PROCESS	163	-0.46447274	-1.4564203
THYROID_HORMONE_RECEPTOR_BINDING	17	-0.6932494	-1.4681964
SH3_SH2_ADAPTOR_ACTIVITY	43	-0.5572921	-1.4509261
DNA_DAMAGE_RESPONSESIGNAL_TRANSDUCTION	34	-0.5844977	-1.4523119
PORE_COMPLEX	36	-0.57700294	-1.4465114
TRANSLATION_FACTOR_ACTIVITY_NUCLEIC_ACID_BINDING	38	-0.56628996	-1.4424969
RIBOSOMAL_SUBUNIT	20	-0.6606814	-1.4566759
REGULATION_OF_PROTEIN_METABOLIC_PROCESS	173	-0.45744562	-1.462632
RIBONUCLEASE_ACTIVITY	25	-0.62663937	-1.46401
DOUBLE_STRANDED_RNA_BINDING	17	-0.67789066	-1.4392189

GO gene sets with an FDR q-value <0.25 for TMD8 cells treated with 200nM ITF-B

MICROTUBULE_ASSOCIATED_COMPLEX	47	0.72462815	1.8491583
HYDRO_LYASE_ACTIVITY	27	0.8008314	1.8347774
LIPID_TRANSPORT	28	0.82004654	1.8783444
PROTEASE_INHIBITOR_ACTIVITY	41	0.7141151	1.7822123
CYTOSKELETAL_PROTEIN_BINDING	158	0.57884413	1.7302155
OXIDOREDUCTASE_ACTIVITY_ACTING_ON_THE_ALDEHYDE_OR_OXO_GROUP_OF_DONORS	22	0.7671115	1.7220784
ION_TRANSPORT	184	0.55078495	1.6670012
CELL_SUBSTRATE_ADHESION	39	0.6752655	1.668684
VESICLE	121	0.5704933	1.6759623
CYTOPLASMIC_VESICLE	116	0.5766303	1.6587607
TRANSMEMBRANE_RECEPTOR_PROTEIN_TYROSINE_KINASE_SIGNALING_PATHWAY	83	0.5995249	1.6787605
MEMBRANE_BOUND_VESICLE	114	0.5748014	1.6697694
OXIDOREDUCTASE_ACTIVITY_ACTING_ON_THE_ALDEHYDE_OR_OXO_GROUP_OF_DONORSNAD_OR_NADP_AS_ACCEPTOR	16	0.8263915	1.6815355
ENZYME_LINKED_RECEPTOR_PROTEIN_SIGNALING_PATHWAY	140	0.5845588	1.7321614
CELL_MATRIX_ADHESION	38	0.67811024	1.6504881
CARBON_OXYGEN_LYASE_ACTIVITY	31	0.7152309	1.6973898
CYTOPLASMIC_MEMBRANE_BOUND_VESICLE	112	0.57134026	1.6358961
OXIDOREDUCTASE_ACTIVITY_ACTING_ON_THE_CH_CH_GROUP_OF_DONORS	23	0.7526253	1.6902388
CLATHRIN_COATED_VESICLE	36	0.6943509	1.6836087
ENDOSOME_TRANSPORT	23	0.7209724	1.6283348
CATION_TRANSPORT	146	0.54091	1.6297524
SYNAPTIC_VESICLE	15	0.806389	1.6359048
LYASE_ACTIVITY	69	0.6270366	1.7013003
MONOVALENT_INORGANIC_CATION_TRANSPORT	93	0.58024406	1.6372137
METAL_ION_TRANSPORT	117	0.5606015	1.6378144
SERINE_TYPE_ENDOPEPTIDASE_INHIBITOR_ACTIVITY	25	0.6971932	1.6073362
TUBULIN_BINDING	46	0.61997646	1.5974485
ANION_TRANSPORT	31	0.692894	1.5991988
REGULATION_OF_NEUROTRANSMITTER_LEVELS	24	0.6918017	1.5900297
RESPONSE_TO_NUTRIENT_LEVELS	29	0.683074	1.5844029
VESICLE_MEDIATED_TRANSPORT	193	0.5161734	1.577159
LIPID_METABOLIC_PROCESS	318	0.4866567	1.5565604
VOLTAGE_GATED_CHANNEL_ACTIVITY	73	0.57716095	1.5525063
LIPID_HOMEOSTASIS	16	0.7602915	1.5490676
MICROTUBULE_CYTOSKELETON	145	0.5149308	1.5388688
ACTIN_FILAMENT	18	0.71993256	1.5398669
TRANSMEMBRANE_RECEPTOR_PROTEIN_TYROSINE_KINASE_ACTIVITY	43	0.5963804	1.4783607
OXIDOREDUCTASE_ACTIVITY	282	0.46416247	1.4813665
CELLULAR_CARBOHYDRATE_METABOLIC_PROCESS	126	0.50517386	1.4820746
REGULATION_OF_G_PROTEIN_COUPLED_RECEPTOR_PROTEIN_SIGNALING_PATHWAY	23	0.66214263	1.4787116
VOLTAGE_GATED_POTASSIUM_CHANNEL_ACTIVITY	36	0.6231329	1.5319774
SECONDARY_METABOLIC_PROCESS	26	0.651036	1.4828038
POSITIVE_REGULATION_OF_CELLULAR_COMPONENT_ORGANIZATION_AND_BIOGENESIS	36	0.60036856	1.483298
HETEROCYCLE_METABOLIC_PROCESS	27	0.65128094	1.4856887
CYTOSOL	205	0.4830568	1.4836692
CARBOHYDRATE_METABOLIC_PROCESS	180	0.47868147	1.4712026
POTASSIUM_ION_TRANSPORT	58	0.5558958	1.4866707
RESPONSE_TO_EXTRACELLULAR_STIMULUS	33	0.6330094	1.5104522
COATED_VESICLE	46	0.5897706	1.4958532
FEEDING_BEHAVIOR	24	0.6538333	1.4880667
ORGANIC_ACID_METABOLIC_PROCESS	177	0.49341607	1.4996854
DI___TRI_VALENT_INORGANIC_CATION_TRANSMEMBRANE_TRANSPORTER_ACTIVITY	22	0.6809577	1.5052457
CELLULAR_LIPID_CATABOLIC_PROCESS	35	0.6354915	1.5135391
CELLULAR_LIPID_METABOLIC_PROCESS	250	0.4794773	1.511274
HORMONE_METABOLIC_PROCESS	32	0.60543686	1.4675186
INORGANIC_CATION_TRANSMEMBRANE_TRANSPORTER_ACTIVITY	58	0.57274723	1.4906857
MOTOR_ACTIVITY	28	0.64005375	1.5068809
ENZYME_INHIBITOR_ACTIVITY	119	0.5230431	1.5150949
CYTOSKELETON	357	0.4595679	1.4883952
COFACTOR_BINDING	22	0.67867863	1.4959031
ALCOHOL_METABOLIC_PROCESS	87	0.53473794	1.4999487
SUBSTRATE_SPECIFIC_TRANSPORTER_ACTIVITY	392	0.44832945	1.4650539
INORGANIC_ANION_TRANSPORT	18	0.71280366	1.5237353
STEROID_BINDING	18	0.70226437	1.4909915
REGULATION_OF_ANATOMICAL_STRUCTURE_MORPHOGENESIS	25	0.6436024	1.4602364
LIPID_CATABOLIC_PROCESS	38	0.62210876	1.5152202
FATTY_ACID_METABOLIC_PROCESS	61	0.5690057	1.5000827
INTRINSIC_TO_GOLGI_MEMBRANE	15	0.7468192	1.5168229
EPIDERMAL_GROWTH_FACTOR_RECEPTOR_SIGNALING_PATHWAY	22	0.660972	1.4608161
ACTIN_BINDING	76	0.52207065	1.4444488
CARBON_CARBON_LYASE_ACTIVITY	18	0.6884953	1.4457614
MONOCARBOXYLIC_ACID_METABOLIC_PROCESS	86	0.5410509	1.518601
GENERATION_OF_A_SIGNAL_INVOLVED_IN_CELL_CELL_SIGNALING	29	0.61089027	1.4410154
PEROXISOME	45	0.5752274	1.4465324
LIPID_TRANSPORTER_ACTIVITY	28	0.6309211	1.4553784
VOLTAGE_GATED_CATION_CHANNEL_ACTIVITY	66	0.5385512	1.4413351

CARBOHYDRATE_TRANSPORT	19	0.6541888	1.4467908
CATION_TRANSMEMBRANE_TRANSPORTER_ACTIVITY	213	0.46732634	1.4497474
MICROBODY	45	0.5752273	1.4510971
