# Supplementary material for: Novel HDAC inhibitors exhibit pre-clinical efficacy in lymphoma models and point to the importance of CDKN1A expression levels in mediating their anti-tumor response
Source: Oncotarget. 2014 Dec 30;6(7):5059–71. doi: 10.18632/oncotarget.3239 (PMC4467133; doi:10.18632/oncotarget.3239)
Supplement: Supplementary file 5 [file oncotarget-06-5059-s005.pdf]

Supplementary table 4. TMD8 DMSO vs TMD8 ITF-A

NAME	SIZE	ES	NES	NOM p-val
GO gene sets with an FDR q-value <0.25 for TMD8 cells treated with DMSO				
POSITIVE_REGULATION_OF_TRANSLATION	35	-0.8127692	-2.0280066	0
HUMORAL_IMMUNE_RESPONSE	32	-0.8087862	-1.988164	0
CYTOKINE_METABOLIC_PROCESS	42	-0.7336005	-1.9290184	0
RESPONSE_TO_OTHER_ORGANISM	82	-0.63882184	-1.8668617	0
POSITIVE_REGULATION_OF_CYTOKINE_BIOSYNTHETIC_PROCESS	25	-0.8104099	-1.9061518	0
CYTOKINE_BIOSYNTHETIC_PROCESS	41	-0.73549986	-1.8749161	0
MESODERM_DEVELOPMENT	22	-0.8213619	-1.8912076	0
RESPONSE_TO_VIRUS	49	-0.6968748	-1.8858156	0
REGULATION_OF_CYTOKINE_BIOSYNTHETIC_PROCESS	38	-0.73304737	-1.8970499	0
REGULATION_OF_I_KAPPAB_KINASE_NF_KAPPAB_CASCADE	90	-0.6049046	-1.7679448	0
RNA_PROCESSING	153	-0.5567354	-1.7513783	0
I_KAPPAB_KINASE_NF_KAPPAB_CASCADE	111	-0.5665419	-1.7404561	0
IMMUNE_RESPONSE	234	-0.52138704	-1.7275748	0
POSITIVE_REGULATION_OF_I_KAPPAB_KINASE_NF_KAPPAB_CASCADE	84	-0.6018473	-1.730548	0
INDUCTION_OF_APOPTOSIS_BY_INTRACELLULAR_SIGNALS	23	-0.7292159	-1.6930474	0.010504202
REGULATION_OF_JNK_ACTIVITY	20	-0.74586356	-1.6753316	0.004140787
RNA_SPLICING	74	-0.5746094	-1.655525	0.002288329
POSITIVE_REGULATION_OF_JNK_ACTIVITY	18	-0.75577295	-1.6447318	0.004329004
IMMUNE_SYSTEM_PROCESS	327	-0.4909607	-1.640147	0
ACTIVATION_OF_JNK_ACTIVITY	16	-0.74643064	-1.6263955	0.01754386
POSITIVE_REGULATION_OF_CELLULAR_PROTEIN_METABOLIC_PROCESS	73	-0.556632	-1.6067505	0.010025063
REGULATION_OF_T_CELL_ACTIVATION	28	-0.66635144	-1.6179832	0.012765957
B_CELL_ACTIVATION	20	-0.7277915	-1.6077001	0.013100437
RIBOSOME_BIOGENESIS_AND_ASSEMBLY	18	-0.73373324	-1.60824	0.023157895
CALCIUM_MEDIATED_SIGNALING	16	-0.7631841	-1.5923302	0.022540983
VIRAL_INFECTIOUS_CYCLE	32	-0.64705193	-1.6093686	0.006944445
CYTOKINE_AND_CHEMOKINE_MEDIATED_SIGNALING_PATHWAY	22	-0.6924846	-1.5885215	0.018018018
POSITIVE_REGULATION_OF_SIGNAL_TRANSDUCTION	123	-0.5199006	-1.5926328	0
REGULATION_OF_LYMPHOCYTE_ACTIVATION	35	-0.61995506	-1.5802643	0.017857144
RRNA_PROCESSING	15	-0.74019104	-1.5614043	0.023354564
POSITIVE_REGULATION_OF_PROTEIN_METABOLIC_PROCESS	75	-0.5429001	-1.5644244	0.006976744
NEURON_APOPTOSIS	17	-0.71819556	-1.5652916	0.017429193
VIRAL_REPRODUCTIVE_PROCESS	36	-0.6210797	-1.542001	0.009070295
RESPONSE_TO_BIOTIC_STIMULUS	119	-0.5129604	-1.544404	0.004739337
RRNA_METABOLIC_PROCESS	16	-0.7361446	-1.5336318	0.01871102
NEGATIVE_REGULATION_OF_TRANSCRIPTION	181	-0.47806486	-1.534155	0
DNA_DAMAGE_RESPONSESIGNAL_TRANSDUCTION	34	-0.60496205	-1.5179834	0.016806724
TRANSCRIPTION_INITIATION_FROM_RNA_POLYMERASE_II_PROMOTER	29	-0.61351216	-1.5136131	0.027713627
PROTEIN_AMINO_ACID_DEPHOSPHORYLATION	63	-0.54581565	-1.5098037	0.013186813
MACROMOLECULAR_COMPLEX_DISASSEMBLY	15	-0.72192454	-1.4968058	0.04121475
CYTOKINE_PRODUCTION	73	-0.53037214	-1.4905823	0.016091954
REGULATION_OF_TRANSLATION	93	-0.5057539	-1.4802495	0.002409639
NEGATIVE_REGULATION_OF_NUCLEOBASENUCLEOSIDENUCLEOTIDE_AND_NUCLEIC_ACID_METABOLIC_PROCESS	201	-0.4551481	-1.4818486	0.005434783
TRANSLATION	178	-0.46099102	-1.4751647	0
DEPHOSPHORYLATION	70	-0.533475	-1.4831516	0.015486726
REGULATION_OF_PROTEIN_METABOLIC_PROCESS	173	-0.45909083	-1.4598484	0.002801121
NEGATIVE_REGULATION_OF_RNA_METABOLIC_PROCESS	128	-0.4752173	-1.4601523	0.002564103
NEGATIVE_REGULATION_OF_TRANSCRIPTION_DNA_DEPENDENT	128	-0.4752173	-1.4477495	0
REGULATION_OF_CELLULAR_PROTEIN_METABOLIC_PROCESS	162	-0.45675185	-1.4446474	0.007594937
VIRAL_GENOME_REPLICATION	21	-0.63773775	-1.4515631	0.054968286
DEFENSE_RESPONSE	267	-0.43196368	-1.4491235	0
MULTI_ORGANISM_PROCESS	163	-0.45150164	-1.4524878	0.002583979
NEGATIVE_REGULATION_OF_MULTICELLULAR_ORGANISMAL_PROCESS	31	-0.57850784	-1.4365535	0.055684455
HEMOSTASIS	47	-0.5385116	-1.4331837	0.052980132
POSITIVE_REGULATION_OF_DEVELOPMENTAL_PROCESS	217	-0.43132812	-1.4156424	0.002590674
POSITIVE_REGULATION_OF_LYMPHOCYTE_ACTIVATION	24	-0.6116233	-1.4172399	0.07692308
GO gene sets with an FDR q-value <0.25 for TMD8 cells treated with 100nM ITF-A				
LIPID_TRANSPORT	28	0.8087958	1.8977226	0
ENZYME_LINKED_RECEPTOR_PROTEIN_SIGNALING_PATHWAY	140	0.5911271	1.7486715	0
ANION_TRANSPORT	31	0.7405795	1.7599506	0.001908397
ENDOSOME_TRANSPORT	23	0.78966826	1.7744665	0
ION_TRANSPORT	184	0.5652366	1.7284015	0
TRANSMEMBRANE_RECEPTOR_PROTEIN_TYROSINE_KINASE_SIGNALING_PATHWAY	83	0.61772954	1.711549	0
CELL_SUBSTRATE_ADHESION	39	0.6890265	1.7031207	0
CELL_MATRIX_ADHESION	38	0.6910569	1.6763035	0.001733102
METAL_ION_TRANSPORT	117	0.5753082	1.6633807	0
CATION_TRANSPORT	146	0.5511388	1.6502247	0
INORGANIC_ANION_TRANSPORT	18	0.77605695	1.6428263	0.004016064
VESICLE_MEDIATED_TRANSPORT	193	0.52978915	1.631711	0.001567398
HETEROCYCLE_METABOLIC_PROCESS	27	0.6892776	1.6157184	0.003683241
REGULATION_OF_G_PROTEIN_COUPLED_RECEPTOR_PROTEIN_SIGNALING_PATHWAY	23	0.704845	1.6002221	0.009124087
MONOVALENT_INORGANIC_CATION_TRANSPORT	93	0.5535281	1.5756966	0.003565062
REGULATION_OF_ANATOMICAL_STRUCTURE_MORPHOGENESIS	25	0.6948523	1.5683546	0.005376344
CELLULAR_LIPID_METABOLIC_PROCESS	250	0.45589477	1.4403391	0.003210273
LIPID_CATABOLIC_PROCESS	38	0.6287114	1.5407213	0.023679417

CARBOHYDRATE_METABOLIC_PROCESS	180	0.47717592	1.4366406	0.0048
AMINO_ACID_DERIVATIVE_METABOLIC_PROCESS	24	0.643156	1.4412271	0.06378987
RESPONSE_TO_NUTRIENT	17	0.6614157	1.4515896	0.06517691
LIPID_HOMEOSTASIS	16	0.70640534	1.4419142	0.04494382
POTASSIUM_ION_TRANSPORT	58	0.5495904	1.447149	0.023809524
HOMOPHILIC_CELL_ADHESION	16	0.6899627	1.4533315	0.055238094
NERVOUS_SYSTEM_DEVELOPMENT	381	0.4457958	1.4434266	0
CELLULAR_LIPID_CATABOLIC_PROCESS	35	0.639095	1.5319171	0.011320755
FATTY_ACID_METABOLIC_PROCESS	61	0.54630923	1.4543504	0.02946593
CARBOXYLIC_ACID_METABOLIC_PROCESS	175	0.48015502	1.4590377	0.004909984
NITROGEN_COMPOUND_BIOSYNTHETIC_PROCESS	25	0.6345515	1.461912	0.04085603
SKELETAL_DEVELOPMENT	103	0.51081336	1.4547011	0.010186757
CYTOSKELETON_ORGANIZATION_AND_BIOGENESIS	207	0.4717112	1.4630829	0.001501502
