# Supplementary material for: Novel HDAC inhibitors exhibit pre-clinical efficacy in lymphoma models and point to the importance of CDKN1A expression levels in mediating their anti-tumor response
Source: Oncotarget. 2014 Dec 30;6(7):5059–71. doi: 10.18632/oncotarget.3239 (PMC4467133; doi:10.18632/oncotarget.3239)
Supplement: Supplementary file 6 [file oncotarget-06-5059-s006.pdf]

Supplementary table 5 . Genes that are commonly upregulated (positive log2 ratios) or downregulated (negative log2 ratios) following treament of DoHH2 and TMD8 cells with 200nM ITF-B

PROBE I.D.	SYMBOL	log2 ratio	P.Value	adj.P.Val
ILMN_1717934	SYT11	3.13E+00	2.26E-13	2.15E-10
ILMN_1757467	H1F0	2.80E+00	6.63E-07	1.26E-05
ILMN_1655595	SERPINE2	2.67E+00	1.02E-09	6.98E-08
ILMN_1715401	MT1G	2.62E+00	1.81E-06	2.93E-05
ILMN_1733851	DACT3	2.59E+00	3.42E-14	6.21E-11
ILMN_1680874	TUBB2B	2.57E+00	6.43E-09	2.92E-07
ILMN_2305225	NDRG4	2.51E+00	1.01E-04	9.01E-04
ILMN_1773964	H1FX	2.40E+00	1.22E-15	7.21E-12
ILMN_1775170	MT1X	2.34E+00	8.91E-07	1.62E-05
ILMN_1686664	MT2A	2.33E+00	4.60E-09	2.24E-07
ILMN_1668194	LMTK3	2.24E+00	3.03E-18	1.43E-13
ILMN_1691156	MT1A	2.22E+00	4.90E-08	1.47E-06
ILMN_1682717	IER3	2.21E+00	1.80E-07	4.30E-06
ILMN_1753342	SAT1	2.16E+00	4.79E-16	5.15E-12
ILMN_1692219	RAB11FIP1	2.15E+00	1.64E-09	9.98E-08
ILMN_1796177	GIPC1	2.14E+00	6.85E-13	4.05E-10
ILMN_1805807	SLC30A3	2.13E+00	1.14E-08	4.60E-07
ILMN_2364022	SLC16A3	2.11E+00	4.02E-05	4.10E-04
ILMN_1740185	TPMT	2.09E+00	1.49E-08	5.69E-07
ILMN_1732071	HIST2H2BE	2.06E+00	5.38E-10	4.32E-08
ILMN_1729453	TSPAN9	2.05E+00	3.71E-12	1.27E-09
ILMN_1689786	ASMTL	2.05E+00	5.94E-07	1.15E-05
ILMN_1682459	TUBB4	2.02E+00	2.85E-08	9.49E-07
ILMN_1779163	C11orf67	2.01E+00	2.08E-15	9.81E-12
ILMN_1690125	PDLIM7	1.98E+00	1.09E-09	7.31E-08
ILMN_1708934	ADM	1.98E+00	4.48E-09	2.20E-07
ILMN_1732615	ASMTL	1.96E+00	3.28E-08	1.06E-06
ILMN_1795429	VCL	1.96E+00	8.36E-14	1.07E-10
ILMN_1697448	TXNIP	1.96E+00	2.19E-12	9.01E-10
ILMN_2124802	MT1H	1.94E+00	1.10E-06	1.94E-05
ILMN_2384857	DHRS2	1.93E+00	1.88E-09	1.11E-07
ILMN_1781374	TUFT1	1.92E+00	3.42E-12	1.19E-09
ILMN_1680624	CREG1	1.92E+00	1.77E-13	1.78E-10
ILMN_1716195	HIST1H2BG	1.90E+00	1.27E-07	3.24E-06
ILMN_1849494		1.83E+00	6.88E-07	1.31E-05
ILMN_2096372	ALDH1A1	1.79E+00	2.08E-12	8.86E-10
ILMN_2347949	G6PD	1.78E+00	2.49E-10	2.45E-08
ILMN_1748124	TSC22D3	1.77E+00	9.46E-13	5.26E-10
ILMN_1695706	H3F3B	1.77E+00	1.73E-09	1.04E-07
ILMN_1775708	SLC2A3	1.76E+00	8.37E-12	2.12E-09
ILMN_1777190	CFD	1.76E+00	4.41E-15	1.60E-11
ILMN_1657996	LOC642035	1.75E+00	5.79E-03	2.93E-02

ILMN_1804384	ASMTL	1.74E+00	7.36E-11	1.01E-08
ILMN_1657111	C14orf78	1.74E+00	3.93E-10	3.41E-08
ILMN_1694268	HES6	1.71E+00	3.61E-16	5.15E-12
ILMN_1694432	CRIP2	1.70E+00	4.72E-12	1.44E-09
ILMN_1756006	ATG2A	1.69E+00	4.87E-07	9.76E-06
ILMN_1709348	ALDH1A1	1.69E+00	9.02E-16	6.26E-12
ILMN_1751956	MGST3	1.68E+00	4.78E-08	1.44E-06
ILMN_2128770	CDR2L	1.67E+00	1.13E-12	5.88E-10
ILMN_1656111	MYLIP	1.67E+00	4.57E-09	2.24E-07
ILMN_2115340	HIST2H4A	1.67E+00	4.33E-05	4.37E-04
ILMN_1789112	TMEM145	1.66E+00	5.51E-06	7.62E-05
ILMN_1750800	ACO1	1.66E+00	1.49E-04	1.27E-03
ILMN_1746801	CGN	1.66E+00	1.22E-09	8.02E-08
ILMN_1776157	SEPT4	1.65E+00	2.61E-08	8.85E-07
ILMN_1703316	LOC255783	1.65E+00	3.09E-10	2.85E-08
ILMN_1708728	H2AFJ	1.65E+00	3.68E-09	1.88E-07
ILMN_1777061	ZSWIM6	1.64E+00	5.85E-13	3.88E-10
ILMN_1718977	GADD45B	1.63E+00	4.90E-11	7.66E-09
ILMN_1780825	RRAS	1.63E+00	5.46E-16	5.15E-12
ILMN_1791726	TUBB3	1.63E+00	9.87E-08	2.63E-06
ILMN_1800602	GCA	1.62E+00	6.09E-05	5.86E-04
ILMN_1659490	LOC653158	1.61E+00	8.16E-10	5.94E-08
ILMN_1659047	HIST2H2AA3	1.61E+00	6.90E-10	5.26E-08
ILMN_1763144	NEU1	1.60E+00	3.86E-11	6.37E-09
ILMN_1651642	GPC2	1.60E+00	1.26E-10	1.48E-08
ILMN_1770629	SLC17A7	1.59E+00	2.38E-03	1.37E-02
ILMN_1747281	EVI5L	1.59E+00	9.09E-07	1.65E-05
ILMN_1653292	PFKFB4	1.59E+00	6.26E-13	3.92E-10
ILMN_1680453	ITM2C	1.58E+00	8.27E-06	1.07E-04
ILMN_1757406	HIST1H1C	1.58E+00	3.82E-10	3.35E-08
ILMN_1718607	TSPAN4	1.58E+00	6.39E-10	4.96E-08
ILMN_1757845	SPIRE1	1.58E+00	3.61E-05	3.74E-04
ILMN_1790680	PDE6D	1.58E+00	5.12E-08	1.52E-06
ILMN_1781752	CLEC16A	1.57E+00	1.12E-12	5.86E-10
ILMN_1751464	TNFSF9	1.57E+00	6.70E-06	8.98E-05
ILMN_2376403	TSC22D3	1.57E+00	1.15E-06	2.00E-05
ILMN_1713505	NPC1	1.57E+00	9.10E-04	6.04E-03
ILMN_1680925	SLC9A3R1	1.56E+00	2.69E-09	1.48E-07
ILMN_1730351	FLJ35767	1.55E+00	6.16E-12	1.68E-09
ILMN_2214678	MXD1	1.55E+00	4.59E-12	1.42E-09
ILMN_2225144	EIF4E3	1.54E+00	1.28E-08	5.03E-07
ILMN_1726388	ACBD7	1.53E+00	2.16E-11	4.24E-09
ILMN_1685441	ASAP3	1.53E+00	4.57E-10	3.82E-08
ILMN_1684158	GPT2	1.53E+00	3.21E-07	6.95E-06
ILMN_1765578	TIPARP	1.52E+00	3.22E-11	5.61E-09
ILMN_1723625	MAP4K2	1.51E+00	1.38E-03	8.56E-03

ILMN_1742382	RIMS3	1.51E+00	2.44E-06	3.79E-05
ILMN_1667295	VASN	1.51E+00	2.08E-10	2.11E-08
ILMN_2173611	MT1E	1.51E+00	4.87E-07	9.77E-06
ILMN_2038775	TUBB2A	1.50E+00	1.41E-05	1.68E-04
ILMN_1804117	FAM89B	1.50E+00	1.22E-10	1.45E-08
ILMN_1651496	HIST1H2BD	1.50E+00	1.97E-10	2.03E-08
ILMN_1736096	DLL3	1.49E+00	2.29E-08	7.99E-07
ILMN_2384591	HN1	1.49E+00	1.09E-06	1.93E-05
ILMN_2179726	C16orf93	1.49E+00	2.19E-12	9.01E-10
ILMN_1737611	VAMP1	1.49E+00	3.25E-11	5.62E-09
ILMN_3246330	LOC100133578	1.48E+00	3.55E-11	6.10E-09
ILMN_1735552	KIF1B	1.48E+00	2.60E-09	1.44E-07
ILMN_1747650	BMP6	1.48E+00	1.69E-11	3.60E-09
ILMN_1655117	WDR19	1.48E+00	6.88E-11	9.64E-09
ILMN_2374352	DBNDD1	1.48E+00	2.78E-05	3.01E-04
ILMN_2366041	ITM2C	1.48E+00	4.24E-08	1.31E-06
ILMN_2358652	NXF1	1.47E+00	3.31E-09	1.74E-07
ILMN_3242900	HIST2H2AA4	1.47E+00	7.00E-14	9.82E-11
ILMN_2355559	PSAP	1.46E+00	7.85E-10	5.80E-08
ILMN_1660806	CSRP2	1.46E+00	6.95E-09	3.09E-07
ILMN_3241692	LOC100129668	1.46E+00	5.52E-07	1.09E-05
ILMN_1691572	TST	1.45E+00	2.13E-06	3.37E-05
ILMN_1663080	LFNG	1.45E+00	7.08E-06	9.40E-05
ILMN_1738093	TMEM118	1.45E+00	2.44E-14	5.76E-11
ILMN_1713266	FAM46C	1.45E+00	3.71E-03	2.01E-02
ILMN_1723678	PRPH	1.44E+00	8.65E-09	3.68E-07
ILMN_1654629	TMEM175	1.44E+00	4.53E-06	6.49E-05
ILMN_1792689	HIST1H2AC	1.43E+00	3.62E-04	2.74E-03
ILMN_1758623	HIST1H2BD	1.43E+00	1.30E-08	5.12E-07
ILMN_1689908	ANKRD13A	1.42E+00	1.13E-13	1.37E-10
ILMN_1682864	SPSB3	1.42E+00	7.57E-09	3.32E-07
ILMN_1729237	CYB5R1	1.42E+00	1.31E-09	8.49E-08
ILMN_2136089	MTE	1.42E+00	1.18E-10	1.42E-08
ILMN_1747935	GOLGB1	1.41E+00	1.76E-12	7.85E-10
ILMN_1703244	MAP1LC3B	1.41E+00	6.68E-11	9.45E-09
ILMN_1669881	TSPAN13	1.41E+00	4.93E-07	9.88E-06
ILMN_1756071	MFGE8	1.41E+00	1.17E-06	2.03E-05
ILMN_2086095	ID2	1.41E+00	5.65E-04	4.01E-03
ILMN_1686478	HIST1H2AG	1.40E+00	7.07E-06	9.39E-05
ILMN_1744517	GNS	1.40E+00	6.94E-07	1.31E-05
ILMN_1881909		1.39E+00	1.38E-05	1.65E-04
ILMN_1705783	NXF1	1.39E+00	1.28E-09	8.30E-08
ILMN_1732296	ID3	1.39E+00	4.69E-03	2.46E-02
ILMN_1749834	LOC388588	1.38E+00	5.00E-15	1.69E-11
ILMN_1806037	TK1	1.38E+00	1.10E-08	4.47E-07
ILMN_2336595	ACSS2	1.37E+00	4.28E-11	6.89E-09

ILMN_1652409	SPATA7	1.37E+00	2.73E-09	1.49E-07
ILMN_1798659	CCDC28A	1.37E+00	6.33E-08	1.80E-06
ILMN_1750100	TUBB4Q	1.37E+00	1.20E-11	2.73E-09
ILMN_1664922	FLNB	1.36E+00	5.86E-07	1.14E-05
ILMN_2144426	HIST2H2AA3	1.36E+00	2.80E-10	2.68E-08
ILMN_1814333	SERPINI1	1.36E+00	2.52E-10	2.47E-08
ILMN_1661755	FAM129B	1.36E+00	6.94E-07	1.31E-05
ILMN_1700690	VAT1	1.36E+00	1.70E-09	1.03E-07
ILMN_1679725	PCYOX1	1.36E+00	7.84E-11	1.06E-08
ILMN_2082209	C20orf100	1.36E+00	7.53E-12	1.95E-09
ILMN_1766657	STOM	1.35E+00	5.33E-09	2.51E-07
ILMN_1838863		1.35E+00	2.01E-08	7.28E-07
ILMN_1714197	ACSS2	1.35E+00	3.92E-11	6.44E-09
ILMN_1671731	AVPI1	1.35E+00	4.40E-11	7.00E-09
ILMN_1780769	TUBB2C	1.35E+00	1.17E-08	4.71E-07
ILMN_1733675	MPP1	1.35E+00	4.46E-08	1.36E-06
ILMN_1813746	CORO2A	1.35E+00	2.33E-13	2.15E-10
ILMN_1747577	ALAD	1.35E+00	8.17E-11	1.09E-08
ILMN_1659766	BAG3	1.34E+00	2.73E-08	9.18E-07
ILMN_1660436	HSPA1B	1.34E+00	1.32E-10	1.54E-08
ILMN_1812721	LOC728014	1.34E+00	2.99E-04	2.32E-03
ILMN_1759023	WFS1	1.34E+00	1.50E-10	1.69E-08
ILMN_1695590	ADRB2	1.34E+00	5.63E-12	1.60E-09
ILMN_1767894	POLB	1.33E+00	4.59E-13	3.29E-10
ILMN_1784036	CDH15	1.33E+00	8.34E-08	2.27E-06
ILMN_1722056	ATP7B	1.33E+00	6.03E-09	2.78E-07
ILMN_1691436	BLVRA	1.33E+00	2.38E-07	5.42E-06
ILMN_1719972	PLXNA3	1.32E+00	2.33E-06	3.64E-05
ILMN_1755303	ZNF217	1.32E+00	3.16E-13	2.65E-10
ILMN_1793990	ID2	1.32E+00	1.90E-04	1.57E-03
ILMN_1768973	HIST2H2AC	1.32E+00	6.40E-13	3.93E-10
ILMN_3308138	RNU4-2	1.31E+00	8.76E-03	4.16E-02
ILMN_1694514	ZDHHC11	1.31E+00	1.64E-10	1.80E-08
ILMN_1665510	ERRFI1	1.31E+00	2.36E-05	2.61E-04
ILMN_2371055	EFNA1	1.30E+00	1.95E-08	7.08E-07
ILMN_2212999	KIF5C	1.30E+00	3.43E-05	3.59E-04
ILMN_1701655	SLC24A6	1.30E+00	1.24E-05	1.51E-04
ILMN_3238435	SNORA12	1.30E+00	1.47E-07	3.64E-06
ILMN_1764177	JARID2	1.29E+00	4.65E-14	7.74E-11
ILMN_1760727	ANG	1.29E+00	2.86E-07	6.27E-06
ILMN_1679797	ADARB1	1.29E+00	2.61E-04	2.06E-03
ILMN_1704284	LOC648164	1.29E+00	4.77E-05	4.74E-04
ILMN_1687277	IRGM	1.29E+00	1.38E-05	1.65E-04
ILMN_1666206	GSDMB	1.29E+00	2.36E-11	4.55E-09
ILMN_1718063	LIPA	1.29E+00	6.97E-07	1.32E-05
ILMN_1660691	RAB31	1.29E+00	2.71E-11	4.94E-09

ILMN_1800425	SLC9A1	1.29E+00	5.46E-08	1.60E-06
ILMN_1692865	VPS37D	1.29E+00	3.15E-12	1.13E-09
ILMN_1718132	ECHS1	1.28E+00	1.54E-12	7.33E-10
ILMN_1809566	ZSCAN16	1.28E+00	5.07E-12	1.53E-09
ILMN_1681670	SLC25A4	1.28E+00	2.84E-06	4.32E-05
ILMN_1748206	C20orf160	1.28E+00	3.23E-05	3.42E-04
ILMN_1757388	OCEL1	1.28E+00	2.80E-08	9.37E-07
ILMN_1787923	PNPLA2	1.28E+00	1.37E-05	1.64E-04
ILMN_1793017	DGKQ	1.28E+00	1.06E-06	1.88E-05
ILMN_1700257	C4orf32	1.27E+00	2.27E-09	1.29E-07
ILMN_2074044	PLS1	1.27E+00	1.97E-11	3.98E-09
ILMN_1787718	SLC27A1	1.27E+00	4.29E-15	1.60E-11
ILMN_1795826	ATP6V0D1	1.27E+00	3.32E-10	3.00E-08
ILMN_1738684	NRXN2	1.26E+00	8.56E-05	7.85E-04
ILMN_1675878	LOC285359	1.26E+00	2.83E-09	1.53E-07
ILMN_1677843	RAB24	1.26E+00	7.27E-14	9.82E-11
ILMN_1749109	PSAP	1.26E+00	1.36E-07	3.41E-06
ILMN_1761131	PECI	1.26E+00	2.90E-03	1.63E-02
ILMN_2222880	SLC25A42	1.26E+00	1.52E-12	7.33E-10
ILMN_1764729	JAG2	1.26E+00	6.61E-11	9.45E-09
ILMN_1754114	FLJ20021	1.25E+00	5.46E-06	7.56E-05
ILMN_1667796	HBA2	1.25E+00	6.07E-04	4.27E-03
ILMN_2159859	LYSMD4	1.25E+00	1.03E-14	3.24E-11
ILMN_2296843	GCDH	1.25E+00	1.63E-10	1.80E-08
ILMN_1767470	SCPEP1	1.25E+00	1.21E-07	3.11E-06
ILMN_1759436	NOSIP	1.25E+00	4.08E-10	3.52E-08
ILMN_3247159	SCARNA8	1.25E+00	3.43E-04	2.61E-03
ILMN_1746673	SEPT3	1.25E+00	1.36E-04	1.17E-03
ILMN_1791366	RCOR2	1.25E+00	6.27E-13	3.92E-10
ILMN_2334693	NARF	1.25E+00	2.91E-13	2.55E-10
ILMN_1713892	C4orf34	1.24E+00	5.69E-06	7.83E-05
ILMN_2149494	NPL	1.24E+00	5.14E-14	8.09E-11
ILMN_1680774	LOC730994	1.24E+00	2.27E-05	2.53E-04
ILMN_1792455	TMEM158	1.24E+00	9.78E-13	5.31E-10
ILMN_1661599	DDIT4	1.24E+00	1.25E-05	1.52E-04
ILMN_1780236	PMM1	1.23E+00	1.27E-13	1.42E-10
ILMN_1748883	CDKN2D	1.23E+00	1.15E-10	1.40E-08
ILMN_1737089	CAPN5	1.23E+00	8.58E-04	5.74E-03
ILMN_1662846	GPR160	1.23E+00	3.00E-11	5.31E-09
ILMN_1746206	AZI1	1.22E+00	7.25E-06	9.60E-05
ILMN_1712918	NQO2	1.21E+00	1.20E-06	2.08E-05
ILMN_2188722	GLS	1.21E+00	2.01E-07	4.72E-06
ILMN_2151281	GABARAPL1	1.21E+00	9.90E-14	1.23E-10
ILMN_1652631	GLIPR2	1.21E+00	1.58E-07	3.86E-06
ILMN_1693836	LOC653344	1.21E+00	2.63E-14	5.86E-11
ILMN_1744508	FAM53C	1.20E+00	2.33E-09	1.32E-07

ILMN_1663035	SREBF1	1.20E+00	3.49E-10	3.13E-08
ILMN_1752728	FUCA1	1.20E+00	3.44E-06	5.13E-05
ILMN_1696466	ROPN1L	1.20E+00	5.10E-11	7.87E-09
ILMN_2329679	TPST2	1.20E+00	1.53E-11	3.35E-09
ILMN_1749368	HIST1H3H	1.20E+00	2.15E-04	1.74E-03
ILMN_1718766	MT1F	1.19E+00	8.24E-07	1.52E-05
ILMN_1723123	FGFR3	1.19E+00	2.49E-05	2.73E-04
ILMN_1787843	HSDL2	1.19E+00	5.66E-11	8.28E-09
ILMN_1755974	ALDOC	1.19E+00	2.19E-06	3.45E-05
ILMN_1718961	BNIP3L	1.18E+00	1.78E-07	4.26E-06
ILMN_1659544	STX3	1.18E+00	2.95E-12	1.10E-09
ILMN_1756877	C14orf179	1.18E+00	1.12E-09	7.49E-08
ILMN_1766054	ABCA1	1.17E+00	4.12E-08	1.29E-06
ILMN_1707312	NFIL3	1.17E+00	3.68E-09	1.88E-07
ILMN_1797310	ATP6V1D	1.17E+00	2.97E-08	9.84E-07
ILMN_2163206	CCDC110	1.17E+00	1.16E-04	1.02E-03
ILMN_1727479	TPRG1L	1.17E+00	2.75E-13	2.45E-10
ILMN_1797793	BLVRB	1.16E+00	7.18E-13	4.13E-10
ILMN_1827736		1.16E+00	7.27E-14	9.82E-11
ILMN_1689200	DHDH	1.16E+00	1.74E-03	1.04E-02
ILMN_2075927	STK40	1.16E+00	3.42E-12	1.19E-09
ILMN_3307782	FBXL18	1.16E+00	9.05E-08	2.44E-06
ILMN_2367215	PRCP	1.16E+00	2.73E-06	4.17E-05
ILMN_1785356	DENND5A	1.15E+00	4.28E-07	8.75E-06
ILMN_1772316	UNC84A	1.15E+00	7.24E-06	9.59E-05
ILMN_1686082	C14orf79	1.13E+00	5.35E-11	8.00E-09
ILMN_1788489	HIST1H3F	1.13E+00	9.64E-04	6.34E-03
ILMN_1752579	ATP6V0A1	1.13E+00	2.28E-04	1.84E-03
ILMN_1798620	PQLC1	1.13E+00	7.84E-10	5.79E-08
ILMN_1772731	HAGH	1.13E+00	8.52E-05	7.81E-04
ILMN_1681437	DCXR	1.13E+00	1.69E-04	1.41E-03
ILMN_1701991	SYNJ1	1.13E+00	1.81E-08	6.68E-07
ILMN_1721842	RYBP	1.13E+00	1.94E-06	3.10E-05
ILMN_1712748	C14orf129	1.13E+00	6.32E-07	1.21E-05
ILMN_1755075	IDI1	1.12E+00	1.42E-05	1.69E-04
ILMN_1666976	PLD3	1.12E+00	3.37E-08	1.09E-06
ILMN_1802603	RFNG	1.12E+00	1.11E-06	1.94E-05
ILMN_2337974	PKIA	1.12E+00	3.68E-10	3.25E-08
ILMN_1737857	GTF2B	1.12E+00	9.54E-09	3.99E-07
ILMN_1696749	LMNA	1.11E+00	3.13E-06	4.72E-05
ILMN_1694780	GCHFR	1.11E+00	7.86E-07	1.46E-05
ILMN_1693269	GNG8	1.11E+00	1.69E-03	1.02E-02
ILMN_1795835	LOC338758	1.11E+00	1.14E-10	1.40E-08
ILMN_1799104	SPAG9	1.11E+00	8.28E-07	1.52E-05
ILMN_1773576	CPNE3	1.11E+00	1.10E-12	5.82E-10
ILMN_2328972	DNMT3B	1.11E+00	7.02E-09	3.12E-07

ILMN_1692731	TTYH3	1.11E+00	1.65E-09	1.00E-07
ILMN_1676036	LOC649679	1.11E+00	1.36E-10	1.57E-08
ILMN_1821280		1.10E+00	4.19E-07	8.63E-06
ILMN_1697559	G6PD	1.10E+00	5.36E-11	8.00E-09
ILMN_1723709	C9orf116	1.10E+00	2.91E-12	1.09E-09
ILMN_1733110	RASSF7	1.10E+00	3.05E-10	2.83E-08
ILMN_1756715	RUNDC3A	1.10E+00	1.85E-10	1.94E-08
ILMN_1654370	TESK2	1.09E+00	4.37E-08	1.34E-06
ILMN_1755727	KDM5B	1.09E+00	6.31E-13	3.92E-10
ILMN_1708508	PPM1E	1.09E+00	3.27E-07	7.05E-06
ILMN_1769705	LOC440093	1.09E+00	1.44E-09	9.10E-08
ILMN_1718771	CCDC24	1.09E+00	8.30E-14	1.07E-10
ILMN_1658702	HIST1H2BJ	1.09E+00	8.08E-05	7.46E-04
ILMN_2130525	TSPAN13	1.09E+00	9.17E-05	8.33E-04
ILMN_1712400	SERPINB6	1.09E+00	1.31E-03	8.21E-03
ILMN_1757877	HCFC1R1	1.09E+00	1.32E-06	2.24E-05
ILMN_1745655	PEX16	1.09E+00	3.51E-15	1.51E-11
ILMN_1749345	STX5	1.09E+00	7.86E-13	4.48E-10
ILMN_1764764	MUM1	1.09E+00	5.27E-05	5.17E-04
ILMN_1793859	ALDH2	1.08E+00	2.17E-07	5.02E-06
ILMN_1693452	GAL3ST4	1.08E+00	6.48E-08	1.83E-06
ILMN_1768754	PILRB	1.08E+00	1.14E-11	2.65E-09
ILMN_1748831	PPP1R13B	1.08E+00	1.39E-06	2.34E-05
ILMN_1815682	C3orf37	1.08E+00	6.07E-09	2.80E-07
ILMN_2107991	HABP4	1.08E+00	1.79E-10	1.88E-08
ILMN_1789492	ZDHHC8	1.08E+00	5.44E-08	1.60E-06
ILMN_1795865	FGFRL1	1.08E+00	1.91E-11	3.90E-09
ILMN_1810514	SLC25A44	1.08E+00	7.58E-10	5.67E-08
ILMN_1785095	ATP6V0E2	1.07E+00	7.32E-05	6.86E-04
ILMN_1815158	GPS2	1.07E+00	1.87E-10	1.95E-08
ILMN_1782305	NR4A2	1.07E+00	8.36E-10	6.03E-08
ILMN_2342033	F11R	1.07E+00	1.62E-11	3.50E-09
ILMN_2297626	PEG10	1.07E+00	2.67E-04	2.10E-03
ILMN_1778242	CALM1	1.07E+00	2.26E-07	5.21E-06
ILMN_1730539	NPHP3	1.07E+00	1.06E-07	2.78E-06
ILMN_3241046	MYBL1	1.07E+00	8.76E-06	1.13E-04
ILMN_2049021	PTTG3P	1.07E+00	1.71E-10	1.83E-08
ILMN_3238554	SNORA80	1.06E+00	9.49E-10	6.56E-08
ILMN_1681679	TSPO	1.06E+00	1.25E-04	1.09E-03
ILMN_1719097	C18orf8	1.06E+00	2.78E-12	1.08E-09
ILMN_1657361	CBX7	1.06E+00	1.17E-09	7.73E-08
ILMN_1672605	C7orf41	1.06E+00	3.31E-07	7.11E-06
ILMN_1668721	CCND3	1.06E+00	3.59E-13	2.83E-10
ILMN_1809437	RHBDD2	1.06E+00	4.30E-04	3.17E-03
ILMN_1690040	TM7SF2	1.05E+00	3.83E-11	6.34E-09
ILMN_1810055	ITFG3	1.05E+00	3.80E-06	5.59E-05

ILMN_1756920	ADAM15	1.05E+00	3.13E-04	2.42E-03
ILMN_1669523	FOS	1.05E+00	1.08E-09	7.28E-08
ILMN_1753196	PTTG1	1.05E+00	9.39E-11	1.21E-08
ILMN_1803392	TAX1BP3	1.05E+00	5.13E-03	2.65E-02
ILMN_3299905	RNFT2	1.05E+00	9.26E-06	1.18E-04
ILMN_1802557	HEBP1	1.05E+00	3.30E-10	2.99E-08
ILMN_2379718	RAB24	1.05E+00	7.10E-12	1.91E-09
ILMN_1781819	PAPSS1	1.05E+00	6.53E-07	1.25E-05
ILMN_2224907	C4orf34	1.05E+00	5.14E-12	1.53E-09
ILMN_1731107	CCDC92	1.04E+00	2.44E-06	3.79E-05
ILMN_2263718	SPAG9	1.04E+00	3.92E-06	5.73E-05
ILMN_3241870	FRMD8	1.04E+00	5.53E-08	1.62E-06
ILMN_1760792	KLHL7	1.04E+00	2.98E-09	1.58E-07
ILMN_2119774	CYP2R1	1.03E+00	8.99E-12	2.26E-09
ILMN_1680223	PNPLA8	1.03E+00	4.10E-08	1.28E-06
ILMN_1764158	NLGN2	1.03E+00	2.09E-10	2.11E-08
ILMN_1790534	MAP2K3	1.03E+00	1.03E-08	4.25E-07
ILMN_2226324	BRP44L	1.03E+00	1.22E-04	1.07E-03
ILMN_3238233	HIST2H4B	1.03E+00	4.94E-04	3.57E-03
ILMN_2380163	PTPRF	1.03E+00	8.73E-11	1.15E-08
ILMN_2403730	ATP6V1H	1.03E+00	4.18E-08	1.30E-06
ILMN_1778523	KLF9	1.03E+00	1.39E-11	3.12E-09
ILMN_1682147	HOOK2	1.03E+00	2.26E-11	4.40E-09
ILMN_2307455	UBE2A	1.03E+00	1.03E-08	4.23E-07
ILMN_1714349	GLCE	1.03E+00	4.15E-13	3.16E-10
ILMN_1705686	NRGN	1.03E+00	1.70E-05	1.98E-04
ILMN_1750256	ALS2	1.02E+00	9.20E-10	6.47E-08
ILMN_1693401	KLHL28	1.02E+00	4.93E-09	2.36E-07
ILMN_2253065	H2AFJ	1.02E+00	1.16E-06	2.03E-05
ILMN_1691760	FAM45A	1.02E+00	3.84E-08	1.21E-06
ILMN_1762308	LOC654191	1.02E+00	8.80E-09	3.73E-07
ILMN_1802257	PCTP	1.02E+00	1.23E-12	6.21E-10
ILMN_1690653	CDK2AP2	1.02E+00	3.60E-10	3.21E-08
ILMN_1708416	ARL6IP1	1.02E+00	8.68E-08	2.35E-06
ILMN_1660021	M6PRBP1	1.02E+00	4.22E-12	1.37E-09
ILMN_1659761	SNX29	1.02E+00	1.25E-08	4.95E-07
ILMN_3245413	DENND5A	1.02E+00	1.27E-07	3.24E-06
ILMN_1745964	IRAK2	1.02E+00	9.16E-12	2.29E-09
ILMN_1672504	PDXK	1.01E+00	1.08E-04	9.54E-04
ILMN_1761797	CSTB	1.01E+00	1.75E-09	1.05E-07
ILMN_2211780	SLC25A4	1.01E+00	5.26E-07	1.05E-05
ILMN_1708016	C20orf108	1.01E+00	1.54E-09	9.50E-08
ILMN_1698732	PALLD	1.01E+00	7.87E-03	3.80E-02
ILMN_2048607	ANKRD9	1.01E+00	5.23E-08	1.55E-06
ILMN_1668514	PIP5K1C	1.01E+00	6.34E-05	6.07E-04
ILMN_3236756	ACSF2	1.01E+00	3.96E-08	1.24E-06

ILMN_1781386	WIPI1	1.01E+00	2.47E-12	9.80E-10
ILMN_1785284	ALDH6A1	1.00E+00	1.79E-05	2.06E-04
ILMN_1659327	LOC283683	1.00E+00	1.25E-05	1.52E-04
ILMN_1707077	SORT1	1.00E+00	3.17E-10	2.90E-08
ILMN_1803018	KIFC2	1.00E+00	5.59E-10	4.43E-08
ILMN_1654060	MKNK2	1.00E+00	6.19E-03	3.10E-02
ILMN_1725726	DHRS2	9.99E-01	9.44E-10	6.56E-08
ILMN_1783156	LOC650832	9.98E-01	3.09E-09	1.64E-07
ILMN_1714433	MARCKSL1	9.96E-01	5.13E-12	1.53E-09
ILMN_1667711	HRASLS3	9.96E-01	4.96E-04	3.59E-03
ILMN_1737314	BCL6	9.96E-01	3.44E-04	2.62E-03
ILMN_2129161	LRRC32	9.96E-01	2.09E-10	2.11E-08
ILMN_2170209	RASD2	9.95E-01	6.03E-11	8.71E-09
ILMN_1669703	TNK2	9.94E-01	1.13E-08	4.57E-07
ILMN_1784602	CDKN1A	9.94E-01	2.44E-07	5.52E-06
ILMN_1657746	BPHL	9.94E-01	1.52E-12	7.33E-10
ILMN_1733248	NRBP2	9.94E-01	3.23E-10	2.94E-08
ILMN_1796458	GABARAPL2	9.91E-01	1.13E-08	4.57E-07
ILMN_1808238	RBPMS2	9.88E-01	6.65E-04	4.62E-03
ILMN_1752299	RAB6B	9.86E-01	1.12E-08	4.53E-07
ILMN_1769091	PRCP	9.85E-01	2.33E-05	2.58E-04
ILMN_2158336	SH3GLB2	9.84E-01	2.96E-07	6.48E-06
ILMN_2401779	FAM102A	9.83E-01	2.22E-08	7.80E-07
ILMN_1729115	LOC651816	9.82E-01	1.57E-10	1.74E-08
ILMN_1701466	PEX16	9.82E-01	1.64E-11	3.50E-09
ILMN_2406410	RHBDD2	9.81E-01	9.37E-04	6.19E-03
ILMN_2336186	LCMT1	9.77E-01	2.37E-07	5.41E-06
ILMN_1789005	ATP6V0C	9.77E-01	2.87E-08	9.57E-07
ILMN_1656482	OSBPL2	9.76E-01	4.20E-10	3.58E-08
ILMN_1731181	TEX2	9.74E-01	5.36E-06	7.44E-05
ILMN_2207988	SERPINI1	9.73E-01	1.60E-09	9.78E-08
ILMN_1712707	ABHD8	9.73E-01	1.73E-05	2.01E-04
ILMN_2383934	ITGB1	9.70E-01	1.53E-08	5.83E-07
ILMN_1767766	PRDX2	9.70E-01	6.57E-08	1.85E-06
ILMN_1775965	PPP4R4	9.69E-01	4.03E-06	5.87E-05
ILMN_1674650	C9orf95	9.68E-01	1.62E-08	6.07E-07
ILMN_1797482	GCDH	9.67E-01	5.31E-08	1.57E-06
ILMN_2150802	FLJ22795	9.66E-01	5.03E-11	7.79E-09
ILMN_3247261	RAPGEF2	9.66E-01	1.04E-07	2.75E-06
ILMN_1756910	PLA2G15	9.65E-01	6.02E-11	8.71E-09
ILMN_1872457		9.65E-01	4.75E-05	4.73E-04
ILMN_1797950	EXTL2	9.65E-01	6.96E-04	4.80E-03
ILMN_1685286	PPP1R12C	9.64E-01	2.15E-05	2.42E-04
ILMN_1695432	TPST2	9.64E-01	5.82E-11	8.46E-09
ILMN_2063586	CLIC4	9.63E-01	6.63E-10	5.11E-08
ILMN_1756469	GAMT	9.62E-01	5.23E-05	5.14E-04

ILMN_1728298	SBK1	9.61E-01	1.66E-10	1.81E-08
ILMN_2410713	FGFR4	9.61E-01	3.00E-10	2.79E-08
ILMN_2042771	PTTG1	9.59E-01	2.54E-08	8.65E-07
ILMN_2313730	RHOC	9.58E-01	5.03E-08	1.50E-06
ILMN_1656378	NMT2	9.57E-01	3.61E-09	1.85E-07
ILMN_1794914	UBTD1	9.57E-01	3.69E-11	6.18E-09
ILMN_1805643	RILPL1	9.56E-01	7.75E-07	1.44E-05
ILMN_1719343	WDR26	9.55E-01	5.05E-09	2.41E-07
ILMN_1778374	BSG	9.55E-01	1.28E-06	2.18E-05
ILMN_3235325	SCARNA13	9.55E-01	2.59E-04	2.05E-03
ILMN_3242315	SNORD3D	9.54E-01	9.68E-04	6.36E-03
ILMN_1666594	IRF8	9.54E-01	7.05E-04	4.85E-03
ILMN_1723494	SIRT2	9.52E-01	1.14E-06	2.00E-05
ILMN_1667306	RANBP10	9.51E-01	5.54E-11	8.18E-09
ILMN_1674135	RALGPS1	9.50E-01	9.59E-10	6.61E-08
ILMN_1745282	RAGE	9.47E-01	1.54E-03	9.40E-03
ILMN_1718071	AGTPBP1	9.46E-01	4.29E-13	3.21E-10
ILMN_1711699	LOC728014	9.46E-01	1.23E-05	1.50E-04
ILMN_1770245	EPB41L5	9.46E-01	6.14E-04	4.31E-03
ILMN_1706426	DSTN	9.45E-01	1.36E-07	3.42E-06
ILMN_1726434	UNC45A	9.45E-01	3.49E-07	7.40E-06
ILMN_1785756	LOC731314	9.43E-01	5.54E-10	4.41E-08
ILMN_1651296	LOC143666	9.43E-01	2.71E-11	4.94E-09
ILMN_1775762	GNAI2	9.42E-01	1.15E-07	2.99E-06
ILMN_1665526	TCEA2	9.42E-01	1.48E-11	3.26E-09
ILMN_1805007	SEMA4F	9.42E-01	5.54E-07	1.09E-05
ILMN_1651950	TPST1	9.41E-01	6.59E-09	2.98E-07
ILMN_1741869	WDR47	9.40E-01	2.69E-06	4.13E-05
ILMN_1695645	CETN2	9.39E-01	5.90E-05	5.70E-04
ILMN_1739726	JSRP1	9.38E-01	1.28E-10	1.50E-08
ILMN_3239460	OAZ2	9.37E-01	2.15E-05	2.42E-04
ILMN_1684183	RAD9A	9.36E-01	6.08E-09	2.80E-07
ILMN_1729749	HERC5	9.35E-01	3.96E-03	2.13E-02
ILMN_1734353	GPX4	9.33E-01	1.68E-04	1.40E-03
ILMN_1775448	PFN2	9.32E-01	5.50E-04	3.92E-03
ILMN_1751120	HIST1H4H	9.29E-01	6.06E-04	4.26E-03
ILMN_2359742	CTSB	9.29E-01	1.79E-08	6.62E-07
ILMN_1773849	ATP6V0C	9.27E-01	4.43E-08	1.36E-06
ILMN_1696360	CTSB	9.26E-01	2.22E-08	7.80E-07
ILMN_1690442	C18orf45	9.26E-01	4.56E-10	3.82E-08
ILMN_3236765	UPLP	9.25E-01	4.05E-12	1.33E-09
ILMN_2312709	LCMT1	9.25E-01	1.06E-06	1.88E-05
ILMN_3250273	TMOD2	9.24E-01	1.81E-11	3.77E-09
ILMN_1748434	LOC283683	9.23E-01	1.88E-06	3.03E-05
ILMN_1796925	CXADR	9.22E-01	5.66E-08	1.65E-06
ILMN_1763834	APLP1	9.21E-01	6.64E-13	4.02E-10

ILMN_1663092	CITED2	9.21E-01	1.35E-10	1.56E-08
ILMN_1659463	APAF1	9.20E-01	3.89E-07	8.12E-06
ILMN_1721127	HIST1H3D	9.19E-01	3.16E-03	1.75E-02
ILMN_1703279	CXorf57	9.19E-01	2.87E-10	2.72E-08
ILMN_1705346	NBEA	9.19E-01	3.34E-05	3.51E-04
ILMN_1717809	RNF24	9.18E-01	6.39E-06	8.62E-05
ILMN_1723978	LGALS1	9.17E-01	1.48E-06	2.48E-05
ILMN_2367239	RCAN1	9.17E-01	4.58E-06	6.54E-05
ILMN_1737394	LMNA	9.16E-01	1.22E-06	2.11E-05
ILMN_1809931	NDRG1	9.15E-01	1.30E-11	2.95E-09
ILMN_1664826	FBXO33	9.15E-01	2.89E-08	9.63E-07
ILMN_2413527	VCL	9.14E-01	1.34E-06	2.26E-05
ILMN_1763640	KIAA1602	9.13E-01	1.05E-12	5.62E-10
ILMN_1762531	FGF9	9.13E-01	6.66E-06	8.93E-05
ILMN_2382829	PRDX2	9.12E-01	9.42E-10	6.56E-08
ILMN_1743747	RUSC1	9.12E-01	4.34E-04	3.20E-03
ILMN_1791396	DGCR6	9.12E-01	2.70E-09	1.48E-07
ILMN_1892403	SNORD13	9.10E-01	8.08E-05	7.47E-04
ILMN_2128750	PTTG1IP	9.10E-01	1.96E-06	3.13E-05
ILMN_3247636	SCARNA14	9.09E-01	4.17E-04	3.09E-03
ILMN_1707551	AFMID	9.09E-01	2.21E-04	1.78E-03
ILMN_1702501	RPS6KA2	9.07E-01	1.53E-09	9.50E-08
ILMN_2347068	MKNK2	9.06E-01	4.93E-03	2.56E-02
ILMN_2150654	ZSWIM4	9.06E-01	1.01E-11	2.43E-09
ILMN_3241081	LOC100134361	9.06E-01	2.16E-12	9.01E-10
ILMN_1772796	DYNLL2	9.06E-01	4.16E-07	8.58E-06
ILMN_1739222	ETV5	9.05E-01	2.62E-06	4.03E-05
ILMN_1740441	CYB5R3	9.04E-01	7.13E-13	4.13E-10
ILMN_2308582	CYB5R3	9.03E-01	5.87E-09	2.72E-07
ILMN_1758457	TBC1D16	9.03E-01	4.59E-10	3.83E-08
ILMN_2360705	ACSL3	9.03E-01	4.59E-12	1.42E-09
ILMN_1669550	MAD2L2	9.03E-01	2.13E-08	7.55E-07
ILMN_1659189	C9orf89	9.03E-01	1.65E-08	6.19E-07
ILMN_1724700	RIOK3	9.02E-01	2.74E-08	9.19E-07
ILMN_1734290	MAPRE3	9.02E-01	1.96E-12	8.48E-10
ILMN_1788223	RSPH3	9.01E-01	1.30E-07	3.30E-06
ILMN_2413331	TMEM107	9.01E-01	1.42E-03	8.78E-03
ILMN_1747067	NPAS1	9.01E-01	2.02E-03	1.20E-02
ILMN_1667381	CAMKV	9.00E-01	1.75E-11	3.70E-09
ILMN_1678671	KLHL24	9.00E-01	7.83E-04	5.30E-03
ILMN_3249167	SNORA63	8.99E-01	4.29E-03	2.28E-02
ILMN_1800958	ALS2CR4	8.99E-01	1.95E-05	2.22E-04
ILMN_1709809	NHP2L1	8.99E-01	2.55E-06	3.94E-05
ILMN_1774604	PNKD	8.99E-01	5.11E-07	1.02E-05
ILMN_1782070	NPL	8.98E-01	4.35E-13	3.21E-10
ILMN_1677768	POR	8.98E-01	1.32E-03	8.29E-03

ILMN_2074860	RN7SK	8.98E-01	2.30E-03	1.34E-02
ILMN_2229940	C2orf7	8.96E-01	2.09E-08	7.48E-07
ILMN_1783709	RRAGA	8.95E-01	1.47E-04	1.25E-03
ILMN_1742052	SERPINB9	8.95E-01	4.97E-06	7.00E-05
ILMN_3241373	SCARNA18	8.93E-01	6.83E-07	1.30E-05
ILMN_1712095	FOXO4	8.93E-01	2.93E-10	2.75E-08
ILMN_1729430	FBXO18	8.92E-01	3.83E-09	1.94E-07
ILMN_1679641	FAM120B	8.92E-01	1.64E-09	9.98E-08
ILMN_1762262	PKIA	8.92E-01	7.07E-11	9.85E-09
ILMN_1784292	ANKMY2	8.91E-01	1.56E-08	5.93E-07
ILMN_1655498	FLJ25404	8.91E-01	1.51E-07	3.72E-06
ILMN_3228037	LOC729389	8.91E-01	1.32E-07	3.34E-06
ILMN_1655913	NUCB2	8.90E-01	7.96E-10	5.83E-08
ILMN_1695414	ASF1B	8.90E-01	3.63E-07	7.66E-06
ILMN_2363065	RTN3	8.90E-01	1.75E-07	4.20E-06
ILMN_3271092	KLRAQ1	8.90E-01	2.69E-09	1.47E-07
ILMN_1714820	ITGB1	8.89E-01	1.53E-08	5.84E-07
ILMN_1807719	CTNS	8.88E-01	2.36E-10	2.35E-08
ILMN_2221046	GM2A	8.88E-01	3.47E-07	7.38E-06
ILMN_2154052	FVT1	8.88E-01	8.45E-08	2.29E-06
ILMN_2098446	PMAIP1	8.88E-01	9.84E-07	1.76E-05
ILMN_2166524	CCNYL1	8.87E-01	2.49E-08	8.52E-07
ILMN_1675939	IFNGR1	8.86E-01	1.17E-10	1.41E-08
ILMN_1662795	CA2	8.85E-01	5.04E-04	3.63E-03
ILMN_2378952	GPX4	8.84E-01	2.04E-05	2.31E-04
ILMN_1700044	SAP130	8.84E-01	1.31E-09	8.51E-08
ILMN_1784985	PRRT3	8.84E-01	6.10E-08	1.75E-06
ILMN_2329569	C9orf116	8.83E-01	1.60E-11	3.46E-09
ILMN_2390310	C17orf91	8.82E-01	5.55E-08	1.62E-06
ILMN_3248781	SDHAP2	8.81E-01	2.13E-07	4.95E-06
ILMN_1688666	HIST1H2BH	8.81E-01	3.18E-04	2.45E-03
ILMN_1660341	LRPAP1	8.80E-01	8.40E-10	6.04E-08
ILMN_2173004	RAB8B	8.79E-01	4.05E-10	3.50E-08
ILMN_1779147	ENC1	8.78E-01	3.75E-07	7.87E-06
ILMN_2400326	DYRK3	8.78E-01	2.72E-07	6.02E-06
ILMN_1713529	SEMA6A	8.77E-01	1.60E-09	9.77E-08
ILMN_1695354	BMF	8.77E-01	6.03E-07	1.17E-05
ILMN_1793287	LOC642755	8.75E-01	6.29E-04	4.40E-03
ILMN_1716678	NPC2	8.75E-01	2.69E-06	4.12E-05
ILMN_1792409	AMOT	8.74E-01	4.24E-07	8.70E-06
ILMN_1766797	CCS	8.74E-01	7.45E-10	5.59E-08
ILMN_2197365	RGS2	8.73E-01	1.18E-05	1.45E-04
ILMN_1656415	CDKN2C	8.73E-01	1.12E-04	9.88E-04
ILMN_1675612	BLCAP	8.73E-01	6.52E-09	2.95E-07
ILMN_3245452	FAM149B1	8.73E-01	2.51E-07	5.63E-06
ILMN_1785191	TMEM14A	8.73E-01	1.86E-09	1.10E-07

ILMN_1730504	AGPAT4	8.73E-01	8.92E-07	1.62E-05
ILMN_1723467	ITGB1	8.73E-01	1.71E-06	2.80E-05
ILMN_1731619	DAD1	8.73E-01	6.55E-10	5.05E-08
ILMN_2321292	WIPI2	8.71E-01	1.78E-05	2.06E-04
ILMN_2404049	RBM38	8.71E-01	8.08E-08	2.22E-06
ILMN_1796180	CRY2	8.70E-01	2.46E-11	4.62E-09
ILMN_3243156	AHNAK2	8.70E-01	3.85E-07	8.04E-06
ILMN_1774596	BSCL2	8.69E-01	1.10E-06	1.93E-05
ILMN_1740604	RAB11FIP5	8.68E-01	1.20E-14	3.56E-11
ILMN_1744138	CHCHD7	8.68E-01	1.33E-07	3.36E-06
ILMN_1748707	CRELD2	8.68E-01	1.29E-03	8.09E-03
ILMN_2096405	WDR37	8.67E-01	5.32E-08	1.57E-06
ILMN_1755383	LRRC1	8.67E-01	1.83E-06	2.96E-05
ILMN_2051373	NEK2	8.67E-01	1.56E-06	2.59E-05
ILMN_1682368	LRWD1	8.67E-01	2.55E-06	3.94E-05
ILMN_1671621	PCMT1	8.66E-01	5.08E-12	1.53E-09
ILMN_1809695	CAMK2G	8.65E-01	7.51E-12	1.95E-09
ILMN_2055156	PAG1	8.64E-01	4.17E-07	8.60E-06
ILMN_1713978	SDF2	8.64E-01	1.52E-09	9.43E-08
ILMN_1654541	ATP6V1G2	8.64E-01	9.77E-12	2.37E-09
ILMN_1791576	CHSY1	8.63E-01	7.38E-05	6.90E-04
ILMN_3298423	TOX2	8.62E-01	6.15E-06	8.36E-05
ILMN_1733757	LOC374395	8.62E-01	1.11E-08	4.51E-07
ILMN_3235969	SNORA9	8.61E-01	1.17E-08	4.71E-07
ILMN_3236498	LOC253039	8.61E-01	2.30E-07	5.27E-06
ILMN_1812759	GCH1	8.60E-01	2.72E-06	4.17E-05
ILMN_1808591	LOC731049	8.60E-01	1.17E-10	1.41E-08
ILMN_1814213	PQLC3	8.60E-01	5.07E-09	2.42E-07
ILMN_1660727	ENPP5	8.58E-01	3.37E-10	3.04E-08
ILMN_1729217	FAM131A	8.56E-01	1.03E-04	9.17E-04
ILMN_1808860	STX5	8.55E-01	8.31E-09	3.58E-07
ILMN_1713668	TSNAX	8.55E-01	4.20E-06	6.07E-05
ILMN_1685580	CBLB	8.55E-01	9.63E-11	1.24E-08
ILMN_1694240	MAP2K1	8.54E-01	5.17E-08	1.54E-06
ILMN_1673820	HLTF	8.53E-01	6.19E-09	2.84E-07
ILMN_3239445	ZBTB42	8.53E-01	4.32E-10	3.66E-08
ILMN_1677440	ATP6AP2	8.53E-01	1.54E-09	9.53E-08
ILMN_2352121	NT5C3	8.51E-01	8.28E-04	5.56E-03
ILMN_2309180	SMARCD3	8.51E-01	2.84E-10	2.70E-08
ILMN_1678781	SNX26	8.51E-01	1.04E-05	1.30E-04
ILMN_1789558	FAM164A	8.51E-01	1.24E-09	8.11E-08
ILMN_1738866	DEXI	8.50E-01	3.85E-04	2.89E-03
ILMN_2387799	PDPK1	8.50E-01	1.86E-10	1.95E-08
ILMN_1770505	BIK	8.49E-01	7.83E-07	1.45E-05
ILMN_1744534	LYRM5	8.49E-01	1.27E-08	5.02E-07
ILMN_2181883	C14orf129	8.49E-01	6.87E-05	6.51E-04

ILMN_1774261	DOK4	8.48E-01	7.75E-06	1.02E-04
ILMN_1738749	MAST3	8.48E-01	1.45E-12	7.14E-10
ILMN_1681703	FOXO3	8.48E-01	1.66E-06	2.72E-05
ILMN_1801845	DNAL4	8.47E-01	3.09E-10	2.85E-08
ILMN_2175112	KCNS3	8.45E-01	2.94E-05	3.15E-04
ILMN_1667356	CCDC128	8.44E-01	3.77E-10	3.32E-08
ILMN_1711516	ATP6V1A	8.42E-01	3.36E-08	1.09E-06
ILMN_3297510	LOC729495	8.42E-01	8.10E-10	5.91E-08
ILMN_1798588	HLTF	8.41E-01	5.70E-11	8.31E-09
ILMN_1734190	TCEAL3	8.41E-01	7.47E-06	9.84E-05
ILMN_2384536	PECI	8.41E-01	3.02E-04	2.35E-03
ILMN_3241979	TMEM179B	8.40E-01	1.16E-10	1.40E-08
ILMN_1813490	FSD1	8.39E-01	3.61E-08	1.15E-06
ILMN_1741491	ZNHIT1	8.38E-01	4.49E-09	2.20E-07
ILMN_1788538	NCALD	8.37E-01	2.36E-05	2.62E-04
ILMN_2057981	FAM164A	8.37E-01	5.92E-13	3.88E-10
ILMN_3234762	RN5S9	8.35E-01	6.05E-03	3.04E-02
ILMN_1794595	GAMT	8.35E-01	1.06E-03	6.85E-03
ILMN_1703074	CPD	8.35E-01	3.21E-06	4.82E-05
ILMN_1708147	TBPL1	8.34E-01	1.12E-10	1.39E-08
ILMN_2348268	IFFO1	8.34E-01	7.67E-10	5.71E-08
ILMN_1801121	SENP2	8.34E-01	4.24E-05	4.28E-04
ILMN_2323385	TRIM4	8.33E-01	3.71E-08	1.18E-06
ILMN_1778803	ZFAND6	8.33E-01	1.51E-11	3.32E-09
ILMN_1701413	PIGQ	8.33E-01	2.47E-09	1.38E-07
ILMN_1746578	SLC23A2	8.33E-01	8.19E-10	5.95E-08
ILMN_1756022	HIST1H2AM	8.32E-01	2.27E-03	1.32E-02
ILMN_1766637	GLA	8.32E-01	2.26E-09	1.29E-07
ILMN_3239574	SNORD3A	8.32E-01	5.68E-03	2.89E-02
ILMN_1775304	DNAJB1	8.30E-01	1.00E-08	4.17E-07
ILMN_1733746	REEP1	8.29E-01	7.95E-07	1.47E-05
ILMN_1670539	LOC92017	8.29E-01	5.45E-11	8.09E-09
ILMN_2325574	CASC4	8.29E-01	1.54E-10	1.72E-08
ILMN_1758250	TRAFD1	8.29E-01	2.96E-09	1.58E-07
ILMN_1776602	RNASE4	8.28E-01	3.07E-07	6.68E-06
ILMN_1768050	SCOC	8.27E-01	4.19E-08	1.30E-06
ILMN_1708983	CASC1	8.27E-01	1.95E-07	4.60E-06
ILMN_1679949	SLC25A23	8.26E-01	6.32E-07	1.21E-05
ILMN_1776516	ITPKA	8.26E-01	7.32E-03	3.58E-02
ILMN_1814106	C9orf169	8.26E-01	3.86E-06	5.65E-05
ILMN_1720799	TECR	8.25E-01	2.39E-08	8.28E-07
ILMN_1730734	TMEM205	8.24E-01	4.02E-09	2.02E-07
ILMN_1653712	UAP1L1	8.23E-01	1.41E-10	1.60E-08
ILMN_2095133	SPTAN1	8.23E-01	3.00E-06	4.55E-05
ILMN_1695276	MAPRE2	8.23E-01	1.10E-07	2.87E-06
ILMN_2393450	C14orf173	8.23E-01	2.69E-03	1.53E-02

ILMN_1726114	SLC45A3	8.22E-01	1.67E-09	1.01E-07
ILMN_1724437	GCAT	8.21E-01	2.07E-08	7.44E-07
ILMN_1691112	PIGN	8.21E-01	4.60E-11	7.27E-09
ILMN_1658494	C13orf15	8.21E-01	7.63E-12	1.97E-09
ILMN_2072391	SNORD31	8.21E-01	4.40E-08	1.35E-06
ILMN_1664861	ID1	8.20E-01	9.22E-12	2.29E-09
ILMN_3239426	GPN3	8.19E-01	2.20E-08	7.77E-07
ILMN_1657868	SIRT4	8.19E-01	3.56E-08	1.14E-06
ILMN_2252408	CNPY4	8.19E-01	4.01E-06	5.85E-05
ILMN_1732127	RBKS	8.18E-01	9.22E-11	1.20E-08
ILMN_1765880	C16orf57	8.18E-01	1.01E-07	2.69E-06
ILMN_1659953	SEPT3	8.17E-01	5.28E-07	1.05E-05
ILMN_2186061	PFKFB3	8.17E-01	6.24E-09	2.86E-07
ILMN_1651826	BASP1	8.17E-01	1.78E-06	2.89E-05
ILMN_2404135	RIOK3	8.16E-01	1.98E-07	4.66E-06
ILMN_2154053	FVT1	8.15E-01	2.74E-07	6.05E-06
ILMN_2096985	ALDH6A1	8.15E-01	3.61E-06	5.35E-05
ILMN_1684628	ZFP90	8.14E-01	6.06E-11	8.73E-09
ILMN_1776519	RAP1GAP	8.13E-01	4.19E-04	3.11E-03
ILMN_1796336	MAP1S	8.13E-01	7.55E-09	3.32E-07
ILMN_1717219	C7orf70	8.11E-01	7.86E-09	3.42E-07
ILMN_1663685	DGCR6	8.11E-01	8.34E-11	1.11E-08
ILMN_1774982	CDC42EP5	8.11E-01	9.80E-06	1.24E-04
ILMN_2317730	ELMO2	8.10E-01	7.23E-07	1.36E-05
ILMN_1726327	AMY1B	8.09E-01	4.94E-04	3.57E-03
ILMN_1710482	APLP2	8.09E-01	1.60E-08	6.03E-07
ILMN_1881526		8.09E-01	7.25E-09	3.20E-07
ILMN_1690342	LTA4H	8.08E-01	8.99E-09	3.79E-07
ILMN_2138589	MERTK	8.08E-01	5.18E-03	2.67E-02
ILMN_1761844	ZCCHC17	8.07E-01	4.11E-11	6.68E-09
ILMN_1741440	SLC35A1	8.07E-01	3.62E-09	1.85E-07
ILMN_1670037	POLR2L	8.07E-01	2.24E-05	2.50E-04
ILMN_3235404	SNORA57	8.06E-01	2.61E-04	2.07E-03
ILMN_2139816	GPSM2	8.06E-01	1.21E-10	1.44E-08
ILMN_3246206	SNORA79	8.05E-01	4.02E-04	3.00E-03
ILMN_2069821	C4orf32	8.05E-01	9.63E-05	8.68E-04
ILMN_1661622	TBC1D7	8.05E-01	4.00E-11	6.54E-09
ILMN_2061310	ZNF280C	8.05E-01	2.49E-07	5.61E-06
ILMN_1772455	HDAC3	8.05E-01	1.94E-04	1.59E-03
ILMN_1698554	AACS	8.04E-01	7.86E-04	5.32E-03
ILMN_1802205	RHOB	8.03E-01	5.87E-03	2.97E-02
ILMN_1710124	CMTM8	8.03E-01	3.04E-07	6.63E-06
ILMN_1807206	DHRS1	8.03E-01	2.21E-06	3.48E-05
ILMN_1805225	LPCAT3	8.03E-01	5.80E-12	1.62E-09
ILMN_1666967	BRP44L	8.01E-01	6.46E-06	8.70E-05
ILMN_1805842	FHL1	8.01E-01	2.92E-10	2.75E-08

ILMN_3178529	FAM108A2	8.01E-01	6.19E-08	1.77E-06
ILMN_1810069	CCNYL1	8.00E-01	4.69E-07	9.47E-06
ILMN_1772527	C12orf44	8.00E-01	8.77E-09	3.72E-07
ILMN_2224833	ADIPOR2	8.00E-01	3.88E-10	3.39E-08
ILMN_1801377	SLC29A4	7.99E-01	6.31E-07	1.21E-05
ILMN_1800164	PPFIA1	7.98E-01	4.05E-05	4.12E-04
ILMN_2081883	IQCK	7.98E-01	2.29E-05	2.54E-04
ILMN_1692698	VASH2	7.98E-01	1.63E-07	3.96E-06
ILMN_2045419	BNIP3L	7.98E-01	1.99E-06	3.17E-05
ILMN_2403906	ARFIP1	7.97E-01	1.85E-09	1.10E-07
ILMN_1807540	CBARA1	7.97E-01	2.07E-09	1.21E-07
ILMN_1865056		7.96E-01	2.19E-12	9.01E-10
ILMN_1748109	PEX1	7.96E-01	3.80E-06	5.59E-05
ILMN_1673305	RHOC	7.96E-01	1.78E-07	4.26E-06
ILMN_1663407	SURF1	7.96E-01	9.95E-07	1.78E-05
ILMN_1749115	RTN2	7.96E-01	1.80E-05	2.07E-04
ILMN_2157421	STUB1	7.95E-01	9.50E-06	1.20E-04
ILMN_1685369	SLU7	7.95E-01	1.59E-10	1.76E-08
ILMN_1756126	STUB1	7.94E-01	3.25E-08	1.06E-06
ILMN_2308338	BMF	7.93E-01	6.62E-07	1.26E-05
ILMN_1710571	PAPD5	7.92E-01	1.85E-08	6.78E-07
ILMN_3244117	STMN3	7.91E-01	9.71E-04	6.38E-03
ILMN_1756826	MORN2	7.91E-01	5.93E-08	1.72E-06
ILMN_1676891	CDC2L6	7.91E-01	5.07E-05	5.00E-04
ILMN_2305112	CTH	7.91E-01	1.54E-07	3.79E-06
ILMN_1676197	LRP11	7.90E-01	1.86E-10	1.95E-08
ILMN_1806825	C14orf145	7.90E-01	7.47E-10	5.59E-08
ILMN_1792078	RNF114	7.88E-01	3.28E-07	7.07E-06
ILMN_1751195	LOC653438	7.88E-01	7.69E-10	5.72E-08
ILMN_1735735	KATNB1	7.88E-01	7.65E-06	1.00E-04
ILMN_1711208	CELSR2	7.88E-01	2.38E-07	5.41E-06
ILMN_1665217	C3orf34	7.87E-01	2.00E-08	7.25E-07
ILMN_1812701	C4orf33	7.87E-01	5.32E-03	2.73E-02
ILMN_1673282	LAMP2	7.87E-01	4.21E-10	3.58E-08
ILMN_1732923	SIPA1L2	7.86E-01	5.90E-03	2.98E-02
ILMN_1755281	FBXO15	7.86E-01	1.55E-06	2.57E-05
ILMN_1784333	SECISBP2L	7.86E-01	1.97E-05	2.24E-04
ILMN_1804562	SLC31A1	7.85E-01	2.52E-09	1.40E-07
ILMN_1703284	SPIRE2	7.85E-01	9.49E-09	3.97E-07
ILMN_1728360	MED29	7.82E-01	6.36E-08	1.81E-06
ILMN_1714599	CAMLG	7.82E-01	2.87E-06	4.36E-05
ILMN_1676504	RPRML	7.82E-01	6.72E-09	3.00E-07
ILMN_1748291	C1orf55	7.80E-01	4.91E-09	2.36E-07
ILMN_1714159	LUZP1	7.80E-01	1.09E-06	1.92E-05
ILMN_1716056	LMF2	7.80E-01	7.09E-09	3.14E-07
ILMN_1677098	YPEL2	7.80E-01	1.62E-08	6.08E-07

ILMN_1716071	PAQR3	7.80E-01	1.41E-09	9.01E-08
ILMN_1779014	TSPYL1	7.80E-01	1.93E-10	2.00E-08
ILMN_1762899	EGR1	7.80E-01	9.50E-06	1.20E-04
ILMN_1745784	ZNF324	7.79E-01	2.44E-07	5.53E-06
ILMN_1704876	USP38	7.79E-01	1.19E-09	7.85E-08
ILMN_1652072	MGC42105	7.78E-01	4.90E-03	2.55E-02
ILMN_1705302	FCGRT	7.78E-01	5.24E-11	7.91E-09
ILMN_1736700	ALDOA	7.77E-01	8.41E-07	1.54E-05
ILMN_1681008	CGRRF1	7.76E-01	1.71E-08	6.38E-07
ILMN_3305993	LOC728602	7.75E-01	1.73E-07	4.16E-06
ILMN_2400322	DYRK3	7.74E-01	2.19E-07	5.07E-06
ILMN_3305849	LOC728431	7.74E-01	2.25E-11	4.39E-09
ILMN_1884750		7.73E-01	1.29E-06	2.20E-05
ILMN_2217935	RFC1	7.72E-01	2.02E-09	1.18E-07
ILMN_1678678	SLC37A4	7.72E-01	3.43E-09	1.79E-07
ILMN_2157240	MNS1	7.72E-01	1.31E-06	2.22E-05
ILMN_1705144	ULK1	7.72E-01	1.70E-05	1.98E-04
ILMN_1687213	C8orf13	7.71E-01	4.26E-10	3.61E-08
ILMN_1747460	TMEM184B	7.70E-01	4.32E-09	2.15E-07
ILMN_1715804	PITPNA	7.70E-01	1.04E-06	1.85E-05
ILMN_1798212	LLGL1	7.70E-01	2.72E-11	4.94E-09
ILMN_1764571	ARHGAP23	7.70E-01	3.48E-03	1.90E-02
ILMN_2123567	SENP2	7.68E-01	3.36E-05	3.53E-04
ILMN_1683044	PPP1R2	7.68E-01	2.25E-07	5.19E-06
ILMN_1665291	NUB1	7.67E-01	7.01E-10	5.32E-08
ILMN_1723185	ELOF1	7.66E-01	1.40E-09	9.00E-08
ILMN_2312275	SRP54	7.66E-01	2.91E-09	1.55E-07
ILMN_2391861	GSTM1	7.66E-01	2.34E-04	1.87E-03
ILMN_2197846	HADHB	7.65E-01	2.54E-12	9.91E-10
ILMN_2343097	NCALD	7.65E-01	6.54E-06	8.78E-05
ILMN_2347193	GSDMB	7.65E-01	2.83E-10	2.70E-08
ILMN_1732985	PHF20L1	7.65E-01	3.41E-06	5.09E-05
ILMN_1794692	DNMT3B	7.65E-01	1.76E-12	7.85E-10
ILMN_1698259	TMEM100	7.65E-01	1.05E-11	2.48E-09
ILMN_1764769	VWA5A	7.65E-01	3.02E-05	3.23E-04
ILMN_1745954	CORO1C	7.64E-01	7.40E-08	2.05E-06
ILMN_1668369	CDC37	7.64E-01	1.85E-09	1.10E-07
ILMN_1740265	ACOT7	7.64E-01	5.62E-04	4.00E-03
ILMN_1667043	EIF4A3	7.64E-01	5.76E-03	2.92E-02
ILMN_1815154	MYH10	7.63E-01	1.78E-11	3.73E-09
ILMN_1654939	TMED2	7.63E-01	5.86E-07	1.14E-05
ILMN_3193306	C14orf109	7.63E-01	8.37E-10	6.03E-08
ILMN_1714384	PCCA	7.62E-01	2.81E-05	3.03E-04
ILMN_1760160	STX1A	7.62E-01	4.36E-09	2.16E-07
ILMN_1802251	PTTG1IP	7.62E-01	1.29E-05	1.56E-04
ILMN_1699631	GATS	7.62E-01	3.54E-09	1.83E-07

ILMN_1684205	CIB1	7.61E-01	2.31E-09	1.31E-07
ILMN_1800889	FIG4	7.61E-01	3.73E-03	2.02E-02
ILMN_2129388	FAM190B	7.60E-01	5.53E-07	1.09E-05
ILMN_1702487	SGK	7.59E-01	3.11E-04	2.41E-03
ILMN_2413779	SEZ6L2	7.58E-01	1.08E-09	7.27E-08
ILMN_1698323	PLEKHB2	7.58E-01	5.74E-07	1.12E-05
ILMN_1777519	ITGB7	7.57E-01	2.05E-07	4.80E-06
ILMN_1677138	POLR2J3	7.57E-01	2.49E-07	5.61E-06
ILMN_2388177	SPATA7	7.56E-01	2.07E-09	1.21E-07
ILMN_1750429	MKNK1	7.56E-01	3.68E-05	3.81E-04
ILMN_1681628	ZNF277	7.56E-01	5.46E-05	5.33E-04
ILMN_1678004	TMEM41B	7.56E-01	2.03E-11	4.06E-09
ILMN_1761566	C5orf32	7.56E-01	6.71E-04	4.65E-03
ILMN_1815874	NANS	7.55E-01	3.80E-07	7.96E-06
ILMN_3246935	SNORA74B	7.55E-01	4.81E-06	6.80E-05
ILMN_1658992	DPM1	7.53E-01	3.93E-08	1.24E-06
ILMN_1655244	LOC642755	7.53E-01	3.53E-04	2.68E-03
ILMN_1741350	CEP70	7.53E-01	1.76E-08	6.53E-07
ILMN_2113938	TOR1AIP2	7.53E-01	6.37E-10	4.95E-08
ILMN_1658504	CHKA	7.53E-01	7.83E-05	7.27E-04
ILMN_1784364	STARD5	7.52E-01	5.57E-04	3.96E-03
ILMN_1708041	PLEKHF1	7.51E-01	4.77E-06	6.74E-05
ILMN_2214144	TWSG1	7.51E-01	2.62E-04	2.07E-03
ILMN_3247906	RNF114	7.51E-01	1.90E-07	4.49E-06
ILMN_1792168	GALE	7.51E-01	1.44E-08	5.54E-07
ILMN_1793517	RASAL1	7.51E-01	2.84E-05	3.06E-04
ILMN_1728083	EIF4EBP2	7.50E-01	2.12E-08	7.55E-07
ILMN_1710495	PAPLN	7.50E-01	2.05E-10	2.08E-08
ILMN_1701386	STRADB	7.49E-01	6.02E-03	3.03E-02
ILMN_1689968	PLEKHO2	7.48E-01	9.18E-08	2.47E-06
ILMN_1728478	CXCL16	7.48E-01	1.69E-08	6.30E-07
ILMN_1746986	SLC39A3	7.48E-01	3.64E-03	1.98E-02
ILMN_1779486	FAM126B	7.48E-01	1.22E-06	2.10E-05
ILMN_1719998	C9orf45	7.47E-01	3.73E-12	1.27E-09
ILMN_3241034	SNORD3C	7.47E-01	7.55E-03	3.67E-02
ILMN_1685124	TCTN1	7.47E-01	1.88E-06	3.02E-05
ILMN_1757644	UBE2H	7.46E-01	6.04E-10	4.74E-08
ILMN_1802096	ABTB1	7.46E-01	2.41E-08	8.32E-07
ILMN_1734153	GDI1	7.46E-01	1.28E-10	1.50E-08
ILMN_3272500	IFI27L1	7.46E-01	2.98E-05	3.19E-04
ILMN_2349658	TSPO	7.45E-01	4.96E-06	7.00E-05
ILMN_1787324	C16orf48	7.45E-01	3.72E-05	3.84E-04
ILMN_2249018	LOC389816	7.45E-01	4.39E-05	4.42E-04
ILMN_1800739	SPINT2	7.45E-01	1.42E-09	9.05E-08
ILMN_2411915	ATG4B	7.44E-01	1.38E-09	8.90E-08
ILMN_1666545	GCNT1	7.44E-01	1.31E-04	1.14E-03

ILMN_1719622	RABEP1	7.44E-01	8.53E-09	3.65E-07
ILMN_2061950	RABGAP1	7.43E-01	5.37E-05	5.25E-04
ILMN_1774844	MAPKAPK2	7.43E-01	1.58E-09	9.70E-08
ILMN_1815035	DENND2C	7.42E-01	3.82E-08	1.21E-06
ILMN_1679438	MLF1IP	7.40E-01	1.16E-06	2.02E-05
ILMN_1660635	LACTB2	7.39E-01	1.03E-09	6.98E-08
ILMN_3307158	ATG4A	7.39E-01	1.02E-05	1.28E-04
ILMN_2239772	FAM108A3	7.39E-01	7.01E-10	5.32E-08
ILMN_2383516	WDR7	7.38E-01	1.58E-09	9.70E-08
ILMN_1691111	SPATA2L	7.38E-01	4.29E-08	1.33E-06
ILMN_2290998	CCS	7.37E-01	7.68E-07	1.43E-05
ILMN_1664283	C9orf75	7.37E-01	8.13E-06	1.06E-04
ILMN_1742431	LOC651309	7.37E-01	5.59E-10	4.43E-08
ILMN_2081682	SMAP2	7.36E-01	8.66E-07	1.58E-05
ILMN_1793384	JAK1	7.36E-01	3.02E-07	6.60E-06
ILMN_1770454	AGRN	7.35E-01	1.94E-07	4.59E-06
ILMN_1652223	WDR91	7.35E-01	5.26E-09	2.48E-07
ILMN_1743131	TOX4	7.35E-01	1.85E-09	1.10E-07
ILMN_1699644	MARCH3	7.35E-01	4.15E-09	2.07E-07
ILMN_1762712	HHEX	7.35E-01	2.83E-09	1.53E-07
ILMN_1792384	HABP4	7.34E-01	5.70E-13	3.86E-10
ILMN_1701643	GDPD5	7.34E-01	2.44E-08	8.41E-07
ILMN_1753862	SRP54	7.34E-01	5.70E-09	2.64E-07
ILMN_1811574	MAPK8IP3	7.33E-01	4.43E-11	7.01E-09
ILMN_2057573	FAM62B	7.33E-01	1.90E-08	6.96E-07
ILMN_1735052	ULK1	7.32E-01	2.04E-05	2.31E-04
ILMN_1656934	REPS2	7.32E-01	7.77E-08	2.14E-06
ILMN_1800626	SESN1	7.32E-01	1.96E-08	7.13E-07
ILMN_1666057	REEP2	7.32E-01	1.14E-07	2.95E-06
ILMN_1747271	ATP1B2	7.32E-01	1.33E-03	8.33E-03
ILMN_1655796	MARCH3	7.32E-01	4.95E-08	1.48E-06
ILMN_2350801	SLC25A29	7.31E-01	1.11E-09	7.44E-08
ILMN_1710303	TTC25	7.31E-01	1.50E-05	1.77E-04
ILMN_3178406	KLHL29	7.31E-01	1.09E-02	5.00E-02
ILMN_1677829	SLC9A6	7.30E-01	6.04E-13	3.91E-10
ILMN_1734542	OVGP1	7.30E-01	3.19E-05	3.37E-04
ILMN_2344850	VPS26A	7.30E-01	1.42E-09	9.03E-08
ILMN_1808404	RHBDF1	7.30E-01	2.03E-05	2.30E-04
ILMN_1657495	MLEC	7.30E-01	1.64E-05	1.92E-04
ILMN_1778240	GFOD1	7.29E-01	1.64E-03	9.95E-03
ILMN_2338323	CDC25B	7.29E-01	1.09E-07	2.85E-06
ILMN_1676799	MFAP1	7.28E-01	7.83E-10	5.79E-08
ILMN_1682034	HEY2	7.28E-01	3.84E-08	1.21E-06
ILMN_1749403	TSPAN33	7.27E-01	3.54E-04	2.69E-03
ILMN_1727309	FAM82A2	7.27E-01	1.19E-08	4.75E-07
ILMN_1771800	PRKCA	7.26E-01	5.86E-05	5.67E-04

ILMN_1770787	DDAH2	7.26E-01	1.38E-09	8.90E-08
ILMN_1794017	SERTAD1	7.25E-01	6.12E-07	1.18E-05
ILMN_1876924	WNK1	7.25E-01	5.62E-05	5.47E-04
ILMN_1671885	MLF2	7.25E-01	1.58E-07	3.85E-06
ILMN_1811560	SF4	7.25E-01	7.84E-07	1.46E-05
ILMN_1719303	P4HB	7.25E-01	3.99E-06	5.82E-05
ILMN_2099301	UNC84B	7.24E-01	1.55E-05	1.83E-04
ILMN_1814985	PDLIM7	7.24E-01	2.01E-05	2.28E-04
ILMN_1741171	TM2D2	7.23E-01	6.94E-08	1.94E-06
ILMN_2257833	BBS7	7.23E-01	4.63E-10	3.85E-08
ILMN_1764850	HPCAL1	7.23E-01	3.05E-08	1.00E-06
ILMN_1715324	HSD17B8	7.23E-01	1.93E-04	1.58E-03
ILMN_1745778	SLC45A4	7.23E-01	9.75E-05	8.77E-04
ILMN_1688452	LCMT1	7.23E-01	9.48E-07	1.71E-05
ILMN_1782938	SLC16A10	7.23E-01	2.73E-10	2.63E-08
ILMN_1753890	TMEM97	7.23E-01	4.33E-08	1.34E-06
ILMN_3269775	C14orf109	7.22E-01	1.53E-08	5.83E-07
ILMN_1711227	GMDS	7.22E-01	5.43E-10	4.34E-08
ILMN_1745904	CCDC6	7.21E-01	4.29E-11	6.89E-09
ILMN_3243381	MLEC	7.21E-01	8.92E-07	1.62E-05
ILMN_1655068	TOM1L2	7.20E-01	8.33E-10	6.02E-08
ILMN_2346997	RAB23	7.20E-01	4.36E-07	8.87E-06
ILMN_1666453	STK3	7.20E-01	7.66E-07	1.43E-05
ILMN_2374159	HERPUD1	7.20E-01	5.34E-03	2.74E-02
ILMN_2290118	MEGF9	7.19E-01	5.65E-09	2.63E-07
ILMN_1730575	GCLC	7.19E-01	4.44E-04	3.26E-03
ILMN_1659845	KIAA0355	7.19E-01	8.64E-10	6.15E-08
ILMN_1704961	ACTG1	7.18E-01	9.07E-04	6.02E-03
ILMN_1754489	FBXL20	7.17E-01	1.52E-06	2.53E-05
ILMN_2105308	DPM1	7.17E-01	9.17E-08	2.47E-06
ILMN_1780302	DYNC1H1	7.17E-01	5.61E-04	3.99E-03
ILMN_1806456	C14orf45	7.16E-01	2.47E-06	3.83E-05
ILMN_1787680	SELS	7.16E-01	4.51E-09	2.21E-07
ILMN_1800898	ARG2	7.16E-01	1.34E-07	3.38E-06
ILMN_2180582	PNPLA8	7.16E-01	9.88E-09	4.11E-07
ILMN_2352724	PIGN	7.16E-01	1.24E-07	3.19E-06
ILMN_2104106	XPR1	7.16E-01	4.69E-06	6.65E-05
ILMN_1776674	SAC3D1	7.14E-01	4.87E-04	3.53E-03
ILMN_1703335	LACTB	7.14E-01	2.66E-05	2.89E-04
ILMN_1703142	MARCH2	7.13E-01	4.48E-04	3.29E-03
ILMN_2156953	ZFAND6	7.13E-01	9.15E-09	3.85E-07
ILMN_1791226	NXN	7.13E-01	3.87E-10	3.39E-08
ILMN_1665831	CLPTM1	7.12E-01	2.79E-07	6.15E-06
ILMN_1687824	SEPHS2	7.12E-01	1.39E-06	2.33E-05
ILMN_1798030	XPR1	7.12E-01	3.47E-07	7.38E-06
ILMN_1695311	HLA-DMA	7.12E-01	2.64E-05	2.87E-04

ILMN_1734483	BSDC1	7.12E-01	8.96E-08	2.42E-06
ILMN_3201485	LOC644988	7.10E-01	8.47E-09	3.63E-07
ILMN_1719763	MORG1	7.10E-01	1.98E-07	4.66E-06
ILMN_1804652	PLEKHH3	7.10E-01	1.82E-03	1.09E-02
ILMN_1741214	NXPH4	7.09E-01	2.54E-06	3.92E-05
ILMN_1766245	SUPT4H1	7.09E-01	3.70E-13	2.87E-10
ILMN_1746579	UBE2O	7.08E-01	7.93E-10	5.83E-08
ILMN_2331266	NUMB	7.08E-01	3.47E-10	3.11E-08
ILMN_2162989	TMEM189	7.08E-01	2.06E-05	2.33E-04
ILMN_1768031	DEDD2	7.08E-01	2.51E-07	5.65E-06
ILMN_1779401	CHP	7.07E-01	1.10E-07	2.87E-06
ILMN_1803838	CNFN	7.07E-01	2.03E-03	1.20E-02
ILMN_2380698	DSTN	7.07E-01	1.18E-06	2.05E-05
ILMN_1718070	CASP9	7.07E-01	3.98E-07	8.26E-06
ILMN_1794190	CCPG1	7.06E-01	2.24E-05	2.50E-04
ILMN_1694731	CLCN7	7.06E-01	2.19E-09	1.26E-07
ILMN_3239785	LOC100134304	7.06E-01	7.62E-08	2.10E-06
ILMN_3248511	FAM167A	7.05E-01	2.97E-11	5.27E-09
ILMN_1653793	PDPK1	7.05E-01	1.23E-10	1.45E-08
ILMN_2399363	CLEC4A	7.04E-01	6.57E-08	1.85E-06
ILMN_1679232	KIDINS220	7.04E-01	1.05E-07	2.77E-06
ILMN_1731206	NKD2	7.04E-01	3.46E-12	1.19E-09
ILMN_1722738	ROGDI	7.03E-01	1.52E-06	2.53E-05
ILMN_1811682	CYLN2	7.03E-01	1.40E-09	8.97E-08
ILMN_1681754	GGH	7.03E-01	1.86E-06	3.00E-05
ILMN_1729455	EML1	7.03E-01	3.09E-03	1.72E-02
ILMN_2363586	SDCBP	7.02E-01	3.13E-10	2.87E-08
ILMN_1664912	IL11RA	7.02E-01	3.46E-04	2.64E-03
ILMN_1753241	SNTA1	7.02E-01	9.79E-05	8.79E-04
ILMN_1670532	GMCL1	7.02E-01	3.04E-08	9.99E-07
ILMN_2224103	PAPSS1	7.02E-01	4.71E-05	4.69E-04
ILMN_1665095	NELF	7.01E-01	1.33E-07	3.35E-06
ILMN_3229324	SGK1	7.00E-01	6.30E-03	3.15E-02
ILMN_1674811	OASL	6.98E-01	1.85E-08	6.80E-07
ILMN_1753426	KIAA0556	6.98E-01	2.46E-07	5.56E-06
ILMN_2310814	MAPT	6.97E-01	2.06E-08	7.42E-07
ILMN_1712786	AHCYL2	6.97E-01	7.26E-09	3.21E-07
ILMN_1744949	RHOBTB3	6.97E-01	4.33E-08	1.34E-06
ILMN_2313782	ATG4A	6.97E-01	2.22E-08	7.80E-07
ILMN_1798543	STK17B	6.96E-01	5.42E-10	4.34E-08
ILMN_2080751	ADNP2	6.95E-01	7.81E-07	1.45E-05
ILMN_1791147	YPEL3	6.95E-01	3.59E-07	7.60E-06
ILMN_1755954	CPEB3	6.94E-01	2.00E-12	8.59E-10
ILMN_1803744	SELS	6.94E-01	1.50E-09	9.39E-08
ILMN_2318568	HCFC1R1	6.93E-01	1.52E-07	3.73E-06
ILMN_1750880	AMN1	6.93E-01	2.48E-04	1.97E-03

ILMN_1711766	SKP1A	6.93E-01	1.05E-06	1.87E-05
ILMN_1720865	OSBPL7	6.93E-01	4.33E-05	4.37E-04
ILMN_1667460	SULF2	6.93E-01	1.42E-04	1.21E-03
ILMN_1657436	FGFR1OP2	6.92E-01	1.19E-08	4.75E-07
ILMN_1656920	CRIP1	6.92E-01	9.35E-05	8.46E-04
ILMN_1724145	CBX4	6.92E-01	5.08E-08	1.51E-06
ILMN_1654915	LOC646786	6.91E-01	1.22E-07	3.13E-06
ILMN_1654516	TMEM120A	6.90E-01	9.71E-12	2.36E-09
ILMN_1700695	SLC44A1	6.90E-01	8.50E-03	4.06E-02
ILMN_2340027	TSPAN4	6.90E-01	2.49E-11	4.64E-09
ILMN_1693233	KIAA0513	6.90E-01	4.73E-09	2.29E-07
ILMN_2331062	CBFA2T2	6.90E-01	1.55E-04	1.31E-03
ILMN_1758105	ZNF791	6.89E-01	3.62E-08	1.15E-06
ILMN_1667260	MAPK3	6.89E-01	1.10E-03	7.11E-03
ILMN_2083243	MNAT1	6.89E-01	6.32E-07	1.21E-05
ILMN_1669788	NUDT14	6.88E-01	3.72E-09	1.90E-07
ILMN_3246065	CCDC151	6.88E-01	3.55E-10	3.17E-08
ILMN_1769282	FRMD6	6.88E-01	1.02E-08	4.22E-07
ILMN_1809477	CARHSP1	6.88E-01	1.58E-07	3.85E-06
ILMN_2332250	ACOT7	6.87E-01	2.78E-03	1.57E-02
ILMN_1770030	C7orf43	6.87E-01	7.14E-10	5.41E-08
ILMN_3236010	SCARNA11	6.87E-01	7.61E-03	3.70E-02
ILMN_1678268	VPS8	6.87E-01	4.09E-08	1.28E-06
ILMN_2411282	QSOX1	6.87E-01	2.59E-07	5.78E-06
ILMN_3244516	C12orf51	6.86E-01	1.13E-08	4.59E-07
ILMN_1669607	PHKG2	6.86E-01	8.48E-08	2.30E-06
ILMN_3246538	LOC100133866	6.86E-01	1.02E-05	1.28E-04
ILMN_1766803	TUBGCP6	6.85E-01	2.20E-10	2.21E-08
ILMN_1745623	EFCAB4A	6.85E-01	2.92E-05	3.14E-04
ILMN_1756631	ZNF526	6.85E-01	4.12E-07	8.51E-06
ILMN_1671843	PSRC1	6.83E-01	1.02E-09	6.94E-08
ILMN_2288928	PPP4R4	6.83E-01	9.23E-05	8.37E-04
ILMN_3246273	RNU1-3	6.83E-01	4.30E-03	2.28E-02
ILMN_1796146	EIF4E3	6.82E-01	6.81E-05	6.46E-04
ILMN_1716913	TRAPPC1	6.82E-01	5.16E-11	7.90E-09
ILMN_1780141	TMEM66	6.82E-01	8.39E-09	3.60E-07
ILMN_1751708	ITM2B	6.82E-01	3.22E-06	4.83E-05
ILMN_3310351	RNU6-15	6.82E-01	5.99E-03	3.02E-02
ILMN_3243966	SCARNA23	6.81E-01	5.68E-04	4.03E-03
ILMN_2085922	WRB	6.81E-01	2.24E-09	1.28E-07
ILMN_1741954	SMYD3	6.80E-01	2.03E-10	2.07E-08
ILMN_2115490	NBPF20	6.80E-01	7.39E-03	3.61E-02
ILMN_1756849	HIST1H2AE	6.79E-01	8.13E-03	3.91E-02
ILMN_1656910	TRIM6	6.79E-01	2.97E-05	3.18E-04
ILMN_1667857	C12orf52	6.79E-01	4.43E-07	8.99E-06
ILMN_1669940	TMEM38B	6.79E-01	4.16E-04	3.09E-03

ILMN_1761281	LOC441019	6.78E-01	1.49E-04	1.26E-03
ILMN_1686555	FYN	6.78E-01	1.52E-09	9.43E-08
ILMN_1784207	C1orf128	6.78E-01	5.50E-06	7.61E-05
ILMN_1814247	TCFL5	6.78E-01	7.58E-04	5.15E-03
ILMN_3241469	LOC100134537	6.77E-01	7.93E-07	1.47E-05
ILMN_1708029	C9orf127	6.76E-01	8.77E-06	1.13E-04
ILMN_1791728	SLC25A25	6.76E-01	1.95E-06	3.12E-05
ILMN_2352190	CLIP2	6.76E-01	7.35E-11	1.01E-08
ILMN_1804090	SLC25A10	6.76E-01	9.21E-05	8.36E-04
ILMN_1764383	MCOLN1	6.76E-01	3.66E-10	3.24E-08
ILMN_2219512	NPHP4	6.75E-01	3.45E-07	7.36E-06
ILMN_1669433	KIAA0913	6.75E-01	2.25E-06	3.53E-05
ILMN_1676625	SS18L1	6.74E-01	3.30E-06	4.95E-05
ILMN_1773073	PHYH	6.74E-01	1.13E-10	1.39E-08
ILMN_1661432	NUP43	6.74E-01	9.47E-08	2.54E-06
ILMN_3305938	SGK1	6.74E-01	2.73E-03	1.55E-02
ILMN_1697544	SLC25A29	6.74E-01	1.00E-08	4.17E-07
ILMN_1701483	SYP	6.74E-01	2.98E-03	1.67E-02
ILMN_2364674	TRPT1	6.73E-01	6.60E-06	8.86E-05
ILMN_2339779	ATP6V1E1	6.73E-01	4.76E-08	1.44E-06
ILMN_2339955	NR4A2	6.73E-01	9.26E-09	3.88E-07
ILMN_1691616	LOC727935	6.72E-01	1.61E-06	2.66E-05
ILMN_1797964	ARL6IP6	6.72E-01	4.48E-06	6.43E-05
ILMN_1695092	WRB	6.71E-01	4.89E-07	9.80E-06
ILMN_1711608	SSBP2	6.71E-01	2.36E-04	1.89E-03
ILMN_2294762	AMY1A	6.71E-01	1.51E-03	9.28E-03
ILMN_1769702	GPAA1	6.70E-01	1.79E-08	6.61E-07
ILMN_1684210	NPAL3	6.70E-01	4.92E-10	4.05E-08
ILMN_1715332	TTC21A	6.70E-01	7.47E-10	5.59E-08
ILMN_2298818	RPS29	6.69E-01	2.31E-06	3.62E-05
ILMN_1717674	PEPD	6.69E-01	1.13E-04	9.92E-04
ILMN_2340065	UBL5	6.68E-01	1.55E-07	3.81E-06
ILMN_2381899	OPTN	6.67E-01	8.22E-07	1.51E-05
ILMN_3248575	SNORA42	6.67E-01	2.84E-03	1.60E-02
ILMN_1693490	SEC11A	6.66E-01	3.58E-08	1.15E-06
ILMN_2398711	SIRT2	6.66E-01	1.65E-05	1.93E-04
ILMN_1702197	C9orf140	6.65E-01	6.02E-03	3.03E-02
ILMN_1804735	CBS	6.65E-01	2.09E-03	1.23E-02
ILMN_1813386	CORO6	6.65E-01	8.14E-09	3.52E-07
ILMN_1730611	RTN4	6.65E-01	7.50E-05	7.01E-04
ILMN_1775048	DIRAS1	6.65E-01	8.05E-05	7.45E-04
ILMN_2205032	MAGEE1	6.64E-01	1.00E-06	1.79E-05
ILMN_1801822	C18orf25	6.64E-01	4.66E-10	3.87E-08
ILMN_1718303	PVRL2	6.62E-01	3.10E-05	3.30E-04
ILMN_1753002	RAB2B	6.62E-01	4.16E-12	1.36E-09
ILMN_1779241	CRYM	6.62E-01	1.32E-05	1.59E-04

ILMN_2201533	C17orf61	6.61E-01	7.81E-05	7.26E-04
ILMN_2113362	ARL6IP1	6.61E-01	4.27E-05	4.31E-04
ILMN_1696532	RBBP5	6.61E-01	6.20E-09	2.84E-07
ILMN_1759206	PLS1	6.61E-01	1.99E-09	1.17E-07
ILMN_1769734	NT5C3	6.61E-01	1.66E-03	1.00E-02
ILMN_1763359	PEG10	6.61E-01	3.84E-03	2.07E-02
ILMN_1781536	FAH	6.60E-01	1.77E-07	4.25E-06
ILMN_2220739	TMCO3	6.59E-01	4.31E-07	8.78E-06
ILMN_1689029	WDR7	6.59E-01	4.31E-07	8.78E-06
ILMN_2243687	LAMP2	6.59E-01	6.23E-10	4.86E-08
ILMN_1667839	UBR7	6.59E-01	8.47E-10	6.07E-08
ILMN_1780132	PELI2	6.58E-01	1.06E-06	1.87E-05
ILMN_3205424	LOC649917	6.58E-01	3.09E-09	1.64E-07
ILMN_1762224	HPS3	6.57E-01	8.20E-09	3.54E-07
ILMN_1666364	COQ10A	6.57E-01	5.25E-10	4.25E-08
ILMN_2217630	CDKL3	6.57E-01	1.45E-03	8.93E-03
ILMN_2352563	CLDND1	6.56E-01	2.71E-11	4.94E-09
ILMN_1729175	FBXO3	6.55E-01	2.41E-08	8.32E-07
ILMN_2043306	EPB41L5	6.55E-01	2.06E-03	1.21E-02
ILMN_1722218	MBOAT7	6.55E-01	1.46E-08	5.60E-07
ILMN_1691747	KHDRBS3	6.55E-01	3.41E-05	3.57E-04
ILMN_1663444	LIN7B	6.55E-01	8.56E-08	2.32E-06
ILMN_1730816	GPR162	6.54E-01	7.51E-03	3.65E-02
ILMN_2377240	AKTIP	6.54E-01	4.16E-05	4.22E-04
ILMN_1738989	GOLSYN	6.54E-01	1.99E-11	3.99E-09
ILMN_2363591	SDCBP	6.53E-01	1.23E-06	2.11E-05
ILMN_1876266	GJA3	6.52E-01	1.82E-04	1.50E-03
ILMN_2052163	YIPF1	6.52E-01	9.37E-06	1.19E-04
ILMN_1729234	TPP1	6.51E-01	7.49E-04	5.10E-03
ILMN_1787256	HCN3	6.50E-01	5.00E-11	7.79E-09
ILMN_2157075	LRCH4	6.50E-01	3.45E-08	1.11E-06
ILMN_3275575	LOC648980	6.50E-01	2.90E-04	2.27E-03
ILMN_2175474	MTRF1L	6.49E-01	2.07E-05	2.34E-04
ILMN_1751161	COL7A1	6.49E-01	2.10E-11	4.14E-09
ILMN_1716583	NME7	6.48E-01	3.71E-05	3.83E-04
ILMN_1659029	FAM116B	6.48E-01	1.54E-10	1.72E-08
ILMN_1672004	TOB1	6.48E-01	1.24E-06	2.13E-05
ILMN_2171640	ZNF650	6.47E-01	1.11E-03	7.17E-03
ILMN_1729180	GATM	6.47E-01	1.83E-04	1.51E-03
ILMN_2124082	PPP2R5B	6.47E-01	4.35E-12	1.40E-09
ILMN_1710697	BUD31	6.47E-01	9.41E-07	1.70E-05
ILMN_1652371	KIAA1324L	6.47E-01	1.89E-05	2.16E-04
ILMN_2384544	ADAM15	6.47E-01	3.13E-05	3.33E-04
ILMN_1694111	PNKP	6.46E-01	1.86E-06	3.01E-05
ILMN_1752351	LAMP2	6.46E-01	1.78E-11	3.73E-09
ILMN_1771697	VRK3	6.46E-01	4.45E-05	4.47E-04

ILMN_1676749	C3orf15	6.46E-01	1.08E-02	4.94E-02
ILMN_1738401	FOXC1	6.45E-01	5.93E-05	5.72E-04
ILMN_1716547	NAGK	6.45E-01	3.47E-04	2.64E-03
ILMN_2206722	FER1L4	6.44E-01	9.05E-04	6.01E-03
ILMN_1794968	LRRC28	6.44E-01	4.58E-06	6.54E-05
ILMN_1740772	APBB3	6.44E-01	2.02E-06	3.22E-05
ILMN_1767481	XRCC6BP1	6.44E-01	5.36E-05	5.24E-04
ILMN_1798485	ATP6V1E1	6.43E-01	1.09E-06	1.92E-05
ILMN_1797929	MICA	6.43E-01	1.17E-09	7.74E-08
ILMN_2234970	SLC39A3	6.43E-01	4.43E-04	3.26E-03
ILMN_3244646	RNU1G2	6.43E-01	6.58E-03	3.26E-02
ILMN_2320906	RTN3	6.42E-01	5.35E-06	7.44E-05
ILMN_1743205	ABCA7	6.42E-01	2.02E-03	1.20E-02
ILMN_1653220	PITPNM1	6.42E-01	8.62E-08	2.34E-06
ILMN_1813775	GAK	6.42E-01	2.08E-10	2.11E-08
ILMN_1655340	RNF181	6.42E-01	5.89E-07	1.14E-05
ILMN_1651838	RND1	6.42E-01	2.20E-05	2.46E-04
ILMN_2393296	GK	6.41E-01	7.36E-09	3.25E-07
ILMN_1774091	FECH	6.41E-01	1.97E-06	3.13E-05
ILMN_1665066	C4orf14	6.41E-01	2.77E-09	1.50E-07
ILMN_2112755	HSDL1	6.40E-01	3.04E-06	4.60E-05
ILMN_1735156	SLC4A11	6.40E-01	3.43E-04	2.62E-03
ILMN_1794038	FAM49A	6.40E-01	1.32E-04	1.14E-03
ILMN_1813256	CRIPT	6.40E-01	2.38E-11	4.55E-09
ILMN_1769961	DPF1	6.40E-01	2.72E-06	4.16E-05
ILMN_1691151	CHKA	6.40E-01	4.54E-04	3.32E-03
ILMN_1777526	MED20	6.37E-01	4.42E-08	1.35E-06
ILMN_1792597	CRYGS	6.37E-01	1.69E-08	6.30E-07
ILMN_2336781	SOD2	6.37E-01	1.39E-08	5.36E-07
ILMN_1676629	INSIG2	6.37E-01	8.98E-08	2.42E-06
ILMN_1752953	BCL2L12	6.36E-01	1.20E-10	1.43E-08
ILMN_2333766	HISPPD2A	6.36E-01	2.65E-08	8.97E-07
ILMN_1794825	ALDH3A2	6.36E-01	2.11E-08	7.52E-07
ILMN_1710543	SLC39A3	6.36E-01	3.44E-04	2.62E-03
ILMN_1753500	ARHGAP12	6.36E-01	1.58E-07	3.86E-06
ILMN_2234709	C12orf60	6.36E-01	3.35E-07	7.19E-06
ILMN_1673795	HSD17B4	6.36E-01	5.53E-11	8.18E-09
ILMN_1732885	BTBD12	6.36E-01	2.94E-05	3.16E-04
ILMN_3238785	SNHG9	6.35E-01	3.93E-03	2.11E-02
ILMN_1657554	TSPYL2	6.35E-01	9.75E-07	1.75E-05
ILMN_2187487	HEATR5B	6.34E-01	4.41E-06	6.34E-05
ILMN_1758938	SLC31A2	6.33E-01	5.43E-10	4.34E-08
ILMN_1798254	ACTR10	6.33E-01	3.12E-07	6.78E-06
ILMN_1676336	AADACL1	6.33E-01	5.12E-09	2.43E-07
ILMN_1793290	WDR60	6.33E-01	1.40E-08	5.42E-07
ILMN_1651315	HMG20B	6.32E-01	1.96E-07	4.62E-06

ILMN_1756705	CHTF18	6.32E-01	5.02E-06	7.06E-05
ILMN_1716563	PRKCB1	6.32E-01	1.34E-04	1.16E-03
ILMN_1812769	UBXN1	6.32E-01	2.61E-10	2.55E-08
ILMN_2228873	STARD3NL	6.32E-01	1.20E-06	2.08E-05
ILMN_1710326	CLDND1	6.31E-01	6.65E-09	2.99E-07
ILMN_1655429	TNFAIP1	6.30E-01	1.87E-10	1.95E-08
ILMN_1690484	KIAA0895	6.29E-01	3.92E-07	8.16E-06
ILMN_1674710	LOC647784	6.29E-01	4.86E-09	2.34E-07
ILMN_2064694	STIM1	6.29E-01	1.17E-05	1.44E-04
ILMN_3244929	LOC100133163	6.28E-01	1.33E-03	8.32E-03
ILMN_1786211	HERC1	6.28E-01	7.29E-03	3.56E-02
ILMN_1666599	SNORD30	6.28E-01	9.81E-05	8.81E-04
ILMN_1695491	WDYHV1	6.27E-01	2.53E-06	3.91E-05
ILMN_1697561	FBXL16	6.27E-01	6.74E-08	1.89E-06
ILMN_1716922	DHX16	6.27E-01	6.24E-05	5.98E-04
ILMN_1812441	C17orf63	6.27E-01	2.82E-07	6.21E-06
ILMN_2353202	PTK7	6.27E-01	3.18E-03	1.76E-02
ILMN_1739805	NDE1	6.26E-01	4.34E-09	2.15E-07
ILMN_1712505	KDELC1	6.26E-01	2.71E-04	2.14E-03
ILMN_1676358	RALB	6.26E-01	2.06E-07	4.81E-06
ILMN_2112301	DRAP1	6.25E-01	4.15E-06	6.01E-05
ILMN_1669268	MEX3D	6.25E-01	8.40E-04	5.63E-03
ILMN_2103919	LRFN3	6.25E-01	3.07E-08	1.01E-06
ILMN_1674458	KLHL7	6.25E-01	2.68E-07	5.95E-06
ILMN_1804150	HIBADH	6.25E-01	2.83E-09	1.53E-07
ILMN_1686750	MGEA5	6.25E-01	8.42E-09	3.61E-07
ILMN_2199439	CA2	6.25E-01	1.48E-04	1.26E-03
ILMN_1769876	TBC1D2	6.24E-01	3.62E-09	1.86E-07
ILMN_2297710	PLEKHB2	6.24E-01	6.16E-08	1.77E-06
ILMN_1790100	C11orf82	6.23E-01	1.33E-04	1.15E-03
ILMN_2317923	TMEM132A	6.23E-01	5.31E-08	1.57E-06
ILMN_2325506	BCAS4	6.23E-01	1.94E-03	1.15E-02
ILMN_1785202	STAT4	6.23E-01	8.48E-09	3.63E-07
ILMN_1664994	MINPP1	6.23E-01	3.47E-06	5.17E-05
ILMN_2082314	TOM1	6.22E-01	6.24E-04	4.38E-03
ILMN_2155719	NBPF10	6.22E-01	4.46E-03	2.36E-02
ILMN_1658411	CHD4	6.21E-01	1.00E-10	1.27E-08
ILMN_2194627	GMCL1	6.21E-01	2.46E-08	8.45E-07
ILMN_1704353	IGSF3	6.21E-01	2.34E-05	2.59E-04
ILMN_2113535	PCYOX1	6.20E-01	3.44E-09	1.79E-07
ILMN_1792997	NPTN	6.20E-01	1.89E-06	3.04E-05
ILMN_2225595	ACAD8	6.20E-01	3.59E-11	6.11E-09
ILMN_1671969	UGP2	6.20E-01	8.24E-08	2.25E-06
ILMN_2248589	DHX40	6.19E-01	2.63E-06	4.05E-05
ILMN_1808059	BCAS4	6.19E-01	1.12E-03	7.18E-03
ILMN_1716382	LOC387882	6.19E-01	8.19E-06	1.07E-04

ILMN_3245688	DGCR6L	6.18E-01	2.70E-10	2.61E-08
ILMN_1725791	PTPLA	6.17E-01	1.06E-02	4.87E-02
ILMN_1727080	MYO6	6.17E-01	1.69E-10	1.82E-08
ILMN_1730631	C2orf44	6.17E-01	1.99E-09	1.17E-07
ILMN_1771964	GSTA4	6.17E-01	2.64E-05	2.87E-04
ILMN_1792456	CCDC104	6.17E-01	3.72E-07	7.81E-06
ILMN_1859657		6.17E-01	3.71E-07	7.81E-06
ILMN_1811636	IFT57	6.17E-01	2.99E-07	6.54E-06
ILMN_1759766	CTXN1	6.16E-01	4.34E-04	3.20E-03
ILMN_1754584	POLR2J4	6.16E-01	7.71E-09	3.36E-07
ILMN_1909886		6.16E-01	2.83E-05	3.05E-04
ILMN_1684571	RSPH1	6.15E-01	1.85E-06	2.98E-05
ILMN_2197030	ZFYVE21	6.15E-01	5.13E-04	3.70E-03
ILMN_2133675	SGSH	6.15E-01	1.63E-07	3.96E-06
ILMN_1781285	DUSP1	6.15E-01	1.79E-07	4.28E-06
ILMN_1716019	RHBDL3	6.15E-01	2.13E-09	1.23E-07
ILMN_1743034	KIF1B	6.14E-01	1.03E-10	1.30E-08
ILMN_1764380	GLTP	6.14E-01	2.58E-04	2.04E-03
ILMN_1653134	TMEM188	6.14E-01	5.69E-07	1.11E-05
ILMN_1806692	HEXB	6.14E-01	4.43E-10	3.73E-08
ILMN_2410965	MRI1	6.14E-01	1.28E-09	8.30E-08
ILMN_1654016	MRLC2	6.14E-01	2.90E-05	3.12E-04
ILMN_2195482	CACNB3	6.13E-01	7.30E-03	3.57E-02
ILMN_1738816	FOXO1	6.13E-01	5.58E-07	1.10E-05
ILMN_1763265	CHMP1B	6.13E-01	1.03E-08	4.25E-07
ILMN_1672728	KCTD5	6.12E-01	3.13E-08	1.02E-06
ILMN_2370685	C6orf1	6.12E-01	4.44E-09	2.19E-07
ILMN_1704446	SLC6A10P	6.12E-01	5.58E-09	2.60E-07
ILMN_1798256	UPP1	6.12E-01	3.14E-12	1.13E-09
ILMN_1714108	TP53INP1	6.11E-01	7.37E-03	3.60E-02
ILMN_1751020	PACSIN1	6.11E-01	8.16E-08	2.23E-06
ILMN_2368292	TSEN34	6.11E-01	5.18E-10	4.20E-08
ILMN_2315979	LBH	6.11E-01	1.34E-05	1.61E-04
ILMN_3243961	ZNF252	6.10E-01	2.21E-07	5.10E-06
ILMN_3308335	RNU6-1	6.10E-01	5.98E-03	3.02E-02
ILMN_1801421	EMD	6.10E-01	1.57E-06	2.60E-05
ILMN_1713807	MAN1C1	6.10E-01	2.41E-05	2.66E-04
ILMN_1668092	ESAM	6.09E-01	4.65E-05	4.65E-04
ILMN_1689953	CD81	6.09E-01	2.51E-08	8.58E-07
ILMN_2325978	HDGF2	6.09E-01	1.56E-06	2.59E-05
ILMN_3242176	UBR3	6.09E-01	2.78E-03	1.57E-02
ILMN_1724333	TOR1B	6.09E-01	3.06E-10	2.84E-08
ILMN_3236904	LOC648740	6.09E-01	7.37E-03	3.60E-02
ILMN_1754126	SH2D5	6.09E-01	2.15E-05	2.41E-04
ILMN_1677487	ANKZF1	6.09E-01	1.11E-05	1.38E-04
ILMN_3261938	LOC100130154	6.08E-01	1.41E-07	3.51E-06

ILMN_1784753	PAIP2	6.08E-01	6.80E-10	5.20E-08
ILMN_2234710	C12orf60	6.08E-01	9.44E-09	3.95E-07
ILMN_1725534	ACTN4	6.07E-01	9.50E-05	8.58E-04
ILMN_2246510	TSC1	6.07E-01	2.90E-07	6.35E-06
ILMN_1736533	RND2	6.06E-01	2.64E-07	5.89E-06
ILMN_1696956	ARID3B	6.06E-01	2.86E-09	1.53E-07
ILMN_2367681	SPAG1	6.06E-01	3.30E-05	3.48E-04
ILMN_1732609	KIAA1539	6.06E-01	4.97E-05	4.91E-04
ILMN_2178587	ANKRD6	6.06E-01	2.80E-04	2.19E-03
ILMN_3292990	LOC100131727	6.05E-01	3.32E-05	3.49E-04
ILMN_1806705	ASB6	6.04E-01	1.91E-09	1.13E-07
ILMN_1655191	CASZ1	6.03E-01	4.08E-04	3.04E-03
ILMN_1695847	ZKSCAN5	6.03E-01	4.15E-10	3.57E-08
ILMN_1684346	TNFAIP8L1	6.03E-01	5.70E-07	1.11E-05
ILMN_2279961	LAMP2	6.03E-01	5.70E-09	2.64E-07
ILMN_1742124	KIAA1128	6.03E-01	2.45E-07	5.55E-06
ILMN_1694589	PAQR8	6.03E-01	1.86E-07	4.42E-06
ILMN_2053992	HIST4H4	6.03E-01	4.37E-04	3.22E-03
ILMN_1656676	ZYG11B	6.02E-01	2.08E-07	4.86E-06
ILMN_1678766	DYNLT1	6.02E-01	3.85E-05	3.96E-04
ILMN_1695290	FERMT2	6.02E-01	1.35E-11	3.03E-09
ILMN_1671933	CLCC1	6.01E-01	2.93E-10	2.75E-08
ILMN_1758398	GUK1	6.01E-01	7.54E-08	2.08E-06
ILMN_2228732	CCNG2	6.01E-01	2.42E-08	8.33E-07
ILMN_1657515	RPS6KA5	6.00E-01	1.42E-09	9.05E-08
ILMN_2059886	TTC38	6.00E-01	6.47E-03	3.22E-02
ILMN_1748911	SNAP23	6.00E-01	2.87E-05	3.09E-04
ILMN_1759772	LRRC56	6.00E-01	3.84E-09	1.94E-07
ILMN_1740742	UROD	5.99E-01	2.04E-06	3.24E-05
ILMN_3251132	TMOD2	5.99E-01	2.16E-09	1.25E-07
ILMN_3249110	CSRNP2	5.99E-01	6.65E-06	8.91E-05
ILMN_2328977	DNMT3B	5.98E-01	4.45E-05	4.46E-04
ILMN_1749405	KIAA1191	5.98E-01	5.91E-07	1.15E-05
ILMN_2382990	HK1	5.98E-01	2.03E-03	1.20E-02
ILMN_1778673	GOLGA7	5.98E-01	1.45E-04	1.23E-03
ILMN_1712705	RAB40C	5.98E-01	1.60E-03	9.73E-03
ILMN_1778064	FICD	5.97E-01	1.83E-08	6.74E-07
ILMN_3203189	LOC100133591	5.97E-01	2.60E-05	2.84E-04
ILMN_1705464	MRPL41	5.97E-01	4.53E-04	3.32E-03
ILMN_2336982	NPTN	5.96E-01	1.98E-07	4.65E-06
ILMN_2382724	C17orf95	5.96E-01	2.17E-07	5.02E-06
ILMN_2162253	NMU	5.96E-01	1.80E-05	2.07E-04
ILMN_2062687	FARP2	5.95E-01	1.47E-05	1.74E-04
ILMN_1803564	YIPF1	5.95E-01	1.70E-04	1.42E-03
ILMN_1711928	FLJ20920	5.94E-01	3.99E-06	5.83E-05
ILMN_1651800	GSTM4	5.94E-01	1.38E-04	1.18E-03

ILMN_1662618	SQSTM1	5.94E-01	1.65E-06	2.71E-05
ILMN_1758057	TOR1AIP2	5.94E-01	3.55E-09	1.83E-07
ILMN_1803824	ZDHHC9	5.94E-01	1.77E-10	1.88E-08
ILMN_1694106	GPD1L	5.93E-01	6.14E-04	4.31E-03
ILMN_3305735	LOC730101	5.93E-01	3.14E-06	4.73E-05
ILMN_3241970	POLR2J2	5.93E-01	4.67E-06	6.64E-05
ILMN_1746883	SAT2	5.93E-01	6.42E-04	4.48E-03
ILMN_1793474	INSIG1	5.92E-01	3.82E-03	2.06E-02
ILMN_2174369	ELOVL5	5.92E-01	8.07E-06	1.05E-04
ILMN_1721046	PTMS	5.92E-01	2.64E-03	1.50E-02
ILMN_2146372	KCTD6	5.91E-01	1.50E-09	9.39E-08
ILMN_1765258	HLA-E	5.91E-01	5.26E-05	5.16E-04
ILMN_1721651	LOC646463	5.91E-01	7.36E-07	1.38E-05
ILMN_1662243	ING1	5.91E-01	3.16E-04	2.44E-03
ILMN_1737561	LOC88523	5.90E-01	5.37E-05	5.25E-04
ILMN_1658911	LOC647349	5.90E-01	5.45E-04	3.89E-03
ILMN_1797384	UROS	5.90E-01	1.13E-08	4.58E-07
ILMN_1802808	LOC654103	5.90E-01	1.02E-03	6.66E-03
ILMN_1725471	GK	5.90E-01	9.19E-09	3.86E-07
ILMN_1800837	CFDP1	5.89E-01	1.33E-05	1.60E-04
ILMN_3299520	PRKCB	5.89E-01	2.04E-05	2.31E-04
ILMN_1810214	JUND	5.89E-01	5.93E-04	4.19E-03
ILMN_1801101	ZBTB48	5.89E-01	2.48E-08	8.51E-07
ILMN_1674282	PPARD	5.88E-01	8.12E-10	5.92E-08
ILMN_3301749	SPNS2	5.88E-01	2.78E-08	9.32E-07
ILMN_1785570	SUSD3	5.88E-01	2.35E-04	1.88E-03
ILMN_1795719	RPA1	5.88E-01	4.15E-06	6.01E-05
ILMN_1681118	CAPRIN2	5.88E-01	9.99E-07	1.79E-05
ILMN_2402341	MAPK3	5.88E-01	3.11E-05	3.31E-04
ILMN_1752837	ARL8B	5.87E-01	1.36E-08	5.28E-07
ILMN_1705114	NUMB	5.86E-01	4.13E-09	2.07E-07
ILMN_3243890	NDUFA2	5.86E-01	8.39E-06	1.08E-04
ILMN_1758672	FAM107B	5.86E-01	3.74E-09	1.90E-07
ILMN_2047599	TMEM50B	5.86E-01	7.72E-10	5.73E-08
ILMN_2317457	HSD11B1L	5.86E-01	2.27E-03	1.32E-02
ILMN_2398926	C17orf58	5.86E-01	5.81E-06	7.97E-05
ILMN_1764323	LOC124512	5.85E-01	1.41E-09	9.01E-08
ILMN_1671482	GALM	5.85E-01	1.68E-03	1.01E-02
ILMN_1744210	SDHA	5.84E-01	8.62E-06	1.11E-04
ILMN_1683127	ZNF281	5.84E-01	1.92E-07	4.53E-06
ILMN_2376133	KIAA1191	5.84E-01	8.03E-07	1.48E-05
ILMN_1701403	HIP1	5.82E-01	1.43E-03	8.86E-03
ILMN_1805330	KLHL26	5.82E-01	2.58E-03	1.48E-02
ILMN_1756139	LOC643310	5.82E-01	6.33E-08	1.80E-06
ILMN_2172174	NP	5.81E-01	9.38E-06	1.19E-04
ILMN_1808938	PIGF	5.81E-01	1.05E-07	2.77E-06

ILMN_1714170	SPSB1	5.80E-01	9.77E-07	1.75E-05
ILMN_1728256	SPAG9	5.80E-01	2.80E-05	3.03E-04
ILMN_1794074	MXI1	5.80E-01	1.30E-04	1.13E-03
ILMN_1751346	ERBB3	5.80E-01	1.05E-03	6.84E-03
ILMN_2388425	EXTL2	5.80E-01	3.48E-04	2.65E-03
ILMN_3228822	TMEM194A	5.79E-01	2.46E-07	5.55E-06
ILMN_2211728	GUCA1B	5.79E-01	3.34E-06	5.00E-05
ILMN_3249667	LOC100133678	5.79E-01	4.90E-08	1.47E-06
ILMN_1665219	LTBP4	5.78E-01	1.78E-11	3.73E-09
ILMN_1688639	FBXL2	5.78E-01	1.94E-03	1.15E-02
ILMN_1767015	BCORL1	5.78E-01	2.07E-05	2.34E-04
ILMN_1712277	LOC651285	5.77E-01	9.31E-10	6.53E-08
ILMN_1659857	SNAP29	5.77E-01	2.56E-07	5.72E-06
ILMN_1813314	HIST1H2BK	5.77E-01	2.02E-07	4.73E-06
ILMN_2369104	TRAPPC6B	5.77E-01	4.53E-07	9.17E-06
ILMN_1656145	GOT1	5.77E-01	4.40E-05	4.43E-04
ILMN_1758214	RARS2	5.77E-01	7.03E-05	6.63E-04
ILMN_1723048	GJC2	5.76E-01	3.75E-05	3.87E-04
ILMN_1773413	DOCK9	5.76E-01	2.64E-09	1.45E-07
ILMN_3233179	LOC728969	5.76E-01	2.19E-06	3.44E-05
ILMN_3288587	LOC100131785	5.75E-01	1.07E-06	1.89E-05
ILMN_1772605	FRS3	5.75E-01	3.49E-09	1.81E-07
ILMN_2195821	C5orf41	5.75E-01	2.14E-07	4.97E-06
ILMN_1691290	CELSR3	5.74E-01	2.73E-09	1.49E-07
ILMN_1722634	NUCB1	5.74E-01	7.75E-05	7.21E-04
ILMN_1760667	POLR3GL	5.74E-01	1.13E-03	7.23E-03
ILMN_1698404	ERN1	5.74E-01	7.49E-06	9.86E-05
ILMN_1723843	CSNK2A2	5.74E-01	2.12E-08	7.53E-07
ILMN_1698470	SYAP1	5.73E-01	1.18E-05	1.45E-04
ILMN_1687519	SNAP23	5.73E-01	6.25E-08	1.78E-06
ILMN_2311761	AP3S1	5.73E-01	7.79E-06	1.02E-04
ILMN_2056760	MKRN2	5.73E-01	2.28E-07	5.24E-06
ILMN_1703123	AXUD1	5.73E-01	3.96E-08	1.24E-06
ILMN_1700888	ENPP1	5.72E-01	2.28E-04	1.83E-03
ILMN_2393994	CSPP1	5.72E-01	4.17E-08	1.30E-06
ILMN_1718171	MED26	5.72E-01	2.14E-09	1.24E-07
ILMN_1701052	TUBG2	5.72E-01	2.32E-09	1.31E-07
ILMN_2216157	GNA12	5.72E-01	1.74E-10	1.86E-08
ILMN_1702279	KIF3B	5.71E-01	6.38E-09	2.91E-07
ILMN_2059535	PPM1F	5.71E-01	1.11E-06	1.95E-05
ILMN_1719661	SEPX1	5.70E-01	3.22E-05	3.41E-04
ILMN_1746917	LOC729843	5.70E-01	5.64E-11	8.28E-09
ILMN_3235312	LOC92659	5.70E-01	1.08E-06	1.90E-05
ILMN_1726901	KLC1	5.70E-01	7.44E-09	3.28E-07
ILMN_1766916	RPAP3	5.69E-01	2.40E-08	8.30E-07
ILMN_1735680	TMEM30A	5.69E-01	1.05E-08	4.31E-07

ILMN_1682996	VWA5A	5.69E-01	1.40E-04	1.20E-03
ILMN_3237446	SCARNA16	5.69E-01	5.99E-03	3.02E-02
ILMN_2401978	STAT3	5.69E-01	1.56E-03	9.52E-03
ILMN_2187533	CSRNP2	5.68E-01	2.23E-07	5.15E-06
ILMN_1762407	CABLES2	5.68E-01	2.62E-07	5.85E-06
ILMN_1774427	CALCOCO1	5.68E-01	1.40E-05	1.67E-04
ILMN_1672807	CA5B	5.68E-01	4.64E-05	4.63E-04
ILMN_2123665	SBF2	5.68E-01	5.67E-10	4.48E-08
ILMN_1699022	ENDOD1	5.68E-01	3.82E-07	8.00E-06
ILMN_1672121	LOC387856	5.68E-01	2.63E-10	2.56E-08
ILMN_1803211	FBXO2	5.68E-01	1.11E-03	7.14E-03
ILMN_1788604	WBP2	5.68E-01	1.62E-05	1.90E-04
ILMN_1775823	POFUT2	5.67E-01	4.73E-08	1.43E-06
ILMN_2366972	NUDT6	5.66E-01	1.02E-10	1.28E-08
ILMN_1788283	COTL1	5.66E-01	7.80E-05	7.24E-04
ILMN_1654112	PARD6A	5.66E-01	8.91E-09	3.77E-07
ILMN_1810176	MAP3K7	5.66E-01	6.20E-06	8.41E-05
ILMN_1712197	KCNMB3	5.66E-01	5.15E-07	1.03E-05
ILMN_1664068	ERGIC1	5.65E-01	4.27E-07	8.73E-06
ILMN_1783333	C16orf61	5.65E-01	2.51E-07	5.64E-06
ILMN_3249261	LOC100132299	5.64E-01	2.60E-06	4.00E-05
ILMN_1751851	CECR1	5.64E-01	1.64E-07	3.97E-06
ILMN_1730945	C19orf4	5.64E-01	9.57E-04	6.30E-03
ILMN_1689212	RSHL3	5.64E-01	4.14E-08	1.29E-06
ILMN_3300972	SIVA1	5.63E-01	2.06E-07	4.82E-06
ILMN_1751264	CCDC126	5.63E-01	7.54E-07	1.41E-05
ILMN_1754655	TTLL5	5.62E-01	2.89E-06	4.40E-05
ILMN_2334760	ARMCX3	5.61E-01	5.92E-07	1.15E-05
ILMN_1744471	ZNF654	5.61E-01	5.01E-06	7.06E-05
ILMN_1691702	ZNF775	5.61E-01	1.46E-08	5.60E-07
ILMN_2043918	DLEU1	5.60E-01	1.55E-04	1.31E-03
ILMN_1749634	PLRG1	5.60E-01	9.61E-05	8.66E-04
ILMN_1772074	C19orf51	5.60E-01	1.30E-10	1.51E-08
ILMN_2311779	TMUB2	5.60E-01	9.78E-09	4.08E-07
ILMN_1765204	ST13	5.59E-01	4.02E-05	4.10E-04
ILMN_2369756	CCDC46	5.59E-01	2.24E-03	1.31E-02
ILMN_1695946	TRNP1	5.59E-01	5.37E-07	1.06E-05
ILMN_2261379	SRGAP2	5.57E-01	3.18E-05	3.37E-04
ILMN_1852384		5.57E-01	5.74E-07	1.12E-05
ILMN_1688464	MAP6D1	5.57E-01	3.49E-07	7.41E-06
ILMN_1741224	GPR137C	5.57E-01	6.64E-09	2.99E-07
ILMN_2397846	SNCB	5.56E-01	8.96E-06	1.15E-04
ILMN_1727813	BRP44	5.56E-01	4.88E-04	3.54E-03
ILMN_1717052	STARD10	5.56E-01	1.93E-08	7.05E-07
ILMN_2184612	C3orf52	5.56E-01	3.27E-05	3.45E-04
ILMN_3214389	LOC100133583	5.56E-01	1.05E-05	1.31E-04

ILMN_1739259	UBE4A	5.55E-01	1.43E-06	2.40E-05
ILMN_1749424	C21orf122	5.55E-01	1.80E-04	1.49E-03
ILMN_3203444	LOC100132535	5.55E-01	3.09E-07	6.72E-06
ILMN_1699676	C14orf147	5.55E-01	1.25E-05	1.52E-04
ILMN_3238676	ULBP2	5.54E-01	4.43E-05	4.45E-04
ILMN_2209180	P15RS	5.53E-01	1.97E-06	3.13E-05
ILMN_1804854	CTNNA1	5.53E-01	1.34E-08	5.22E-07
ILMN_1731043	TRA2A	5.53E-01	3.78E-06	5.56E-05
ILMN_1732404	C2orf15	5.53E-01	6.58E-05	6.26E-04
ILMN_1730464	DNAL1	5.52E-01	1.43E-07	3.55E-06
ILMN_1718769	ITSN1	5.52E-01	1.63E-09	9.93E-08
ILMN_1762281	DCTN3	5.52E-01	2.04E-04	1.66E-03
ILMN_1716895	RPA3	5.52E-01	1.25E-05	1.51E-04
ILMN_2145396	AKR7A3	5.52E-01	2.85E-05	3.07E-04
ILMN_1677396	NDFIP2	5.51E-01	2.78E-03	1.57E-02
ILMN_1768798	SPAG9	5.50E-01	4.09E-05	4.17E-04
ILMN_2063500	IFT52	5.50E-01	2.04E-09	1.20E-07
ILMN_2386008	MPZL1	5.50E-01	6.85E-10	5.23E-08
ILMN_1761560	PHF13	5.50E-01	6.67E-09	3.00E-07
ILMN_1731287	ARFGAP3	5.49E-01	6.64E-04	4.61E-03
ILMN_1763516	SPINK2	5.49E-01	1.01E-04	9.07E-04
ILMN_1725169	INTS12	5.49E-01	9.72E-11	1.24E-08
ILMN_1706687	KLHL5	5.49E-01	9.98E-04	6.53E-03
ILMN_2409596	RAB11FIP1	5.49E-01	1.24E-09	8.09E-08
ILMN_2343048	ABCB9	5.49E-01	1.35E-06	2.28E-05
ILMN_1652333	FN3KRP	5.48E-01	1.96E-04	1.60E-03
ILMN_2081398	KIF3B	5.48E-01	9.00E-11	1.18E-08
ILMN_1803745	SUOX	5.47E-01	1.71E-03	1.03E-02
ILMN_1758831	RNF31	5.47E-01	1.08E-09	7.28E-08
ILMN_1813374	C19orf28	5.47E-01	7.61E-06	9.99E-05
ILMN_3228595	LOC729768	5.47E-01	2.10E-05	2.37E-04
ILMN_1759411	C16orf79	5.47E-01	1.72E-08	6.38E-07
ILMN_1736806	PAG1	5.47E-01	4.21E-06	6.08E-05
ILMN_1795930	PTGER4	5.47E-01	1.02E-02	4.73E-02
ILMN_1727194	CALU	5.47E-01	1.12E-07	2.92E-06
ILMN_1716089	KANK2	5.47E-01	2.64E-08	8.92E-07
ILMN_1737426	PCMTD1	5.46E-01	3.89E-05	3.99E-04
ILMN_1677446	TMEM189-UBE2V1	5.46E-01	9.83E-08	2.62E-06
ILMN_2347298	TSPYL2	5.46E-01	8.78E-05	8.01E-04
ILMN_1673172	IFT52	5.45E-01	5.39E-08	1.59E-06
ILMN_1692123	ELOVL3	5.45E-01	9.09E-05	8.26E-04
ILMN_1681890	DYNLT3	5.45E-01	8.28E-05	7.62E-04
ILMN_1832672		5.45E-01	2.42E-09	1.36E-07
ILMN_2335813	GCH1	5.44E-01	1.97E-07	4.64E-06
ILMN_1666179	HIST2H3C	5.44E-01	1.01E-02	4.67E-02
ILMN_1780799	ENPP2	5.44E-01	2.52E-08	8.59E-07

ILMN_1715069	TANK	5.44E-01	3.47E-03	1.90E-02
ILMN_3240003	LOC100133012	5.44E-01	1.75E-05	2.03E-04
ILMN_3244526	TCTN3	5.44E-01	3.77E-07	7.92E-06
ILMN_2138765	PLIN2	5.43E-01	2.38E-08	8.26E-07
ILMN_1725188	PRKCI	5.43E-01	3.33E-05	3.50E-04
ILMN_3192836	LOC100130562	5.43E-01	2.83E-03	1.60E-02
ILMN_1678814	UBL5	5.43E-01	1.41E-08	5.45E-07
ILMN_1796179	HIST1H2BK	5.43E-01	6.05E-04	4.26E-03
ILMN_1674032	ABCD3	5.42E-01	3.90E-04	2.92E-03
ILMN_1664440	TP53BP1	5.42E-01	6.36E-05	6.08E-04
ILMN_1698733	CNIH2	5.42E-01	4.60E-06	6.56E-05
ILMN_1708151	LAGE3	5.42E-01	2.62E-03	1.50E-02
ILMN_1810782	SH3KBP1	5.41E-01	6.85E-03	3.38E-02
ILMN_1738921	ACAA1	5.41E-01	3.47E-07	7.38E-06
ILMN_3306440	TMEM194A	5.41E-01	2.14E-10	2.15E-08
ILMN_1712298	ANKRD46	5.41E-01	9.14E-06	1.17E-04
ILMN_1657697	SAR1A	5.40E-01	4.66E-06	6.63E-05
ILMN_1736796	RB1CC1	5.40E-01	3.90E-09	1.97E-07
ILMN_3285153	LOC645979	5.40E-01	1.01E-03	6.60E-03
ILMN_1748093	PAFAH1B3	5.39E-01	2.08E-06	3.30E-05
ILMN_1811921	CSRP1	5.39E-01	2.13E-07	4.95E-06
ILMN_1693394	BCKDK	5.39E-01	9.84E-08	2.62E-06
ILMN_2376667	POFUT2	5.39E-01	2.00E-09	1.17E-07
ILMN_1741475	C7orf47	5.39E-01	3.38E-04	2.58E-03
ILMN_2387952	FAM134B	5.39E-01	8.38E-03	4.00E-02
ILMN_1713744	C14orf132	5.39E-01	3.34E-03	1.84E-02
ILMN_1660837	CLCN3	5.39E-01	5.68E-08	1.65E-06
ILMN_1760121	RRAGC	5.38E-01	9.12E-06	1.16E-04
ILMN_1703487	LMO4	5.38E-01	2.30E-04	1.84E-03
ILMN_1764230	GNPTG	5.38E-01	9.55E-04	6.29E-03
ILMN_1747589	HIST2H2AB	5.37E-01	5.29E-03	2.72E-02
ILMN_1800179	KCNJ4	5.37E-01	5.08E-06	7.12E-05
ILMN_1815306	AP2A1	5.37E-01	4.53E-04	3.32E-03
ILMN_2086238	SMYD4	5.37E-01	7.52E-09	3.31E-07
ILMN_1783636	COX6A1	5.37E-01	1.16E-09	7.67E-08
ILMN_1784287	TGFBR3	5.37E-01	9.99E-03	4.64E-02
ILMN_2412807	DCTN1	5.37E-01	1.42E-06	2.39E-05
ILMN_1783448	DYNC1LI2	5.36E-01	2.45E-07	5.55E-06
ILMN_1661500	B4GALT4	5.36E-01	1.12E-07	2.90E-06
ILMN_1722156	RWDD2A	5.36E-01	3.71E-03	2.01E-02
ILMN_1670609	ATOX1	5.36E-01	1.78E-09	1.06E-07
ILMN_1747627	ABCA2	5.36E-01	1.80E-07	4.30E-06
ILMN_3235472	WDYHV1	5.35E-01	3.14E-05	3.34E-04
ILMN_1683243	VPS45	5.35E-01	1.73E-04	1.44E-03
ILMN_1718633	LRP5L	5.35E-01	1.63E-06	2.69E-05
ILMN_1708105	EZH2	5.34E-01	2.23E-07	5.14E-06

ILMN_1797974	AIG1	5.34E-01	4.39E-06	6.31E-05
ILMN_1739210	NSL1	5.34E-01	1.67E-03	1.01E-02
ILMN_1849941		5.34E-01	1.75E-05	2.03E-04
ILMN_1657550	MVD	5.34E-01	2.29E-08	7.99E-07
ILMN_1801767	ABHD3	5.33E-01	3.40E-07	7.26E-06
ILMN_1674633	UBE2H	5.33E-01	5.73E-08	1.66E-06
ILMN_2170949	SNX10	5.33E-01	5.77E-03	2.92E-02
ILMN_2075051	PGS1	5.33E-01	2.10E-08	7.51E-07
ILMN_1803197	RAB3IP	5.33E-01	6.89E-07	1.31E-05
ILMN_1659610	TJP3	5.33E-01	1.06E-03	6.84E-03
ILMN_1788251	SNN	5.32E-01	6.81E-05	6.46E-04
ILMN_1770412	AHCYL1	5.32E-01	3.54E-04	2.68E-03
ILMN_1667016	FAF1	5.32E-01	5.97E-10	4.70E-08
ILMN_1703102	LOC731777	5.31E-01	4.65E-07	9.39E-06
ILMN_1678504	RHOT1	5.30E-01	2.61E-03	1.49E-02
ILMN_1670377	ZNF20	5.30E-01	4.24E-07	8.70E-06
ILMN_3240144	HBA1	5.30E-01	1.84E-03	1.10E-02
ILMN_1682774	C13orf27	5.30E-01	2.36E-07	5.39E-06
ILMN_1786722	ZNF385A	5.30E-01	7.13E-09	3.16E-07
ILMN_2396982	BCL2L12	5.30E-01	5.52E-08	1.62E-06
ILMN_1778152	FIGNL1	5.30E-01	1.06E-06	1.88E-05
ILMN_1656977	HIBCH	5.30E-01	1.04E-06	1.85E-05
ILMN_1756541	MXD4	5.30E-01	6.57E-04	4.57E-03
ILMN_1791067	TESK1	5.29E-01	1.37E-10	1.58E-08
ILMN_1716157	UBAP2	5.29E-01	7.57E-03	3.68E-02
ILMN_1795778	P4HA2	5.28E-01	1.40E-05	1.67E-04
ILMN_1666305	CDKN3	5.28E-01	1.93E-08	7.04E-07
ILMN_1808417	NPHP4	5.28E-01	6.01E-05	5.79E-04
ILMN_2158003	KIAA1683	5.28E-01	2.29E-08	7.99E-07
ILMN_3245659	ERI1	5.27E-01	4.17E-06	6.04E-05
ILMN_1760400	C8orf41	5.27E-01	1.74E-07	4.19E-06
ILMN_1714765	LOC389599	5.27E-01	2.95E-03	1.65E-02
ILMN_1775012	BBS7	5.26E-01	3.55E-08	1.14E-06
ILMN_1671486	HOMER2	5.26E-01	3.08E-05	3.28E-04
ILMN_1739751	SLC26A11	5.26E-01	3.37E-03	1.85E-02
ILMN_3238623	LYRM7	5.26E-01	2.23E-04	1.80E-03
ILMN_1752247	AKAP13	5.26E-01	5.60E-05	5.45E-04
ILMN_1739946	VKORC1	5.26E-01	2.09E-08	7.49E-07
ILMN_2064606	TBC1D2B	5.26E-01	6.31E-06	8.54E-05
ILMN_1736972	GKAP1	5.25E-01	7.10E-07	1.34E-05
ILMN_1767651	TECPR1	5.25E-01	4.55E-06	6.51E-05
ILMN_1732089	MRI1	5.25E-01	6.47E-08	1.83E-06
ILMN_1782560	CD86	5.25E-01	9.84E-04	6.45E-03
ILMN_1810229	ARID4A	5.25E-01	1.07E-09	7.23E-08
ILMN_2091347	IDH1	5.25E-01	3.42E-04	2.61E-03
ILMN_1866887		5.24E-01	6.54E-03	3.25E-02

ILMN_1726308	FAM10A4	5.23E-01	3.10E-08	1.02E-06
ILMN_2399503	UBN1	5.23E-01	1.32E-06	2.24E-05
ILMN_2364357	RPS6KB2	5.23E-01	5.95E-08	1.72E-06
ILMN_1814526	ADD3	5.23E-01	1.31E-05	1.58E-04
ILMN_1667791	PPFIA4	5.23E-01	1.58E-07	3.86E-06
ILMN_2230902	CTNNA1	5.23E-01	3.14E-11	5.48E-09
ILMN_2202481	UBLCP1	5.23E-01	1.57E-04	1.32E-03
ILMN_1815086	NINJ1	5.23E-01	1.57E-06	2.60E-05
ILMN_2246894	EDF1	5.22E-01	9.37E-08	2.51E-06
ILMN_1787248	SIVA	5.22E-01	3.82E-06	5.61E-05
ILMN_1734696	FRG1	5.22E-01	1.54E-05	1.82E-04
ILMN_1779470	ABCG4	5.22E-01	4.46E-09	2.20E-07
ILMN_1747673	RASL10A	5.22E-01	1.38E-03	8.58E-03
ILMN_1735474	R3HCC1	5.21E-01	8.69E-07	1.59E-05
ILMN_3287583	LOC648390	5.21E-01	1.50E-06	2.51E-05
ILMN_1807600	NPLOC4	5.21E-01	1.48E-03	9.12E-03
ILMN_2358626	ADK	5.21E-01	4.17E-06	6.04E-05
ILMN_1795937	VIL2	5.21E-01	7.33E-04	5.01E-03
ILMN_2142353	GRTP1	5.21E-01	5.01E-03	2.60E-02
ILMN_1766171	SNF8	5.21E-01	1.19E-03	7.55E-03
ILMN_2197519	ZNF627	5.20E-01	3.75E-03	2.03E-02
ILMN_2100458	RFESD	5.20E-01	1.43E-04	1.22E-03
ILMN_1674609	CLTB	5.20E-01	1.60E-10	1.77E-08
ILMN_1797367	TSC1	5.20E-01	2.99E-05	3.20E-04
ILMN_1669366	IFT88	5.20E-01	3.42E-09	1.79E-07
ILMN_2396956	AKAP13	5.19E-01	2.12E-07	4.94E-06
ILMN_1789457	GNL1	5.19E-01	2.06E-06	3.27E-05
ILMN_1787539	ZNF177	5.19E-01	1.45E-05	1.72E-04
ILMN_1711289	GYS1	5.19E-01	1.08E-02	4.94E-02
ILMN_1659976	CLP1	5.19E-01	1.20E-10	1.43E-08
ILMN_3245057	ASAP1	5.18E-01	1.86E-09	1.10E-07
ILMN_2123431	RPS6KC1	5.18E-01	1.87E-04	1.54E-03
ILMN_1661138	GON4L	5.18E-01	3.46E-06	5.16E-05
ILMN_1681812	HIF1AN	5.18E-01	3.27E-05	3.45E-04
ILMN_1676665	CUEDC1	5.18E-01	1.42E-04	1.22E-03
ILMN_2237428	SCD5	5.17E-01	2.72E-09	1.49E-07
ILMN_1717434	DFNB31	5.17E-01	4.77E-10	3.94E-08
ILMN_2061979	LOC440093	5.17E-01	9.23E-06	1.18E-04
ILMN_1693635	TSPYL2	5.17E-01	2.53E-06	3.91E-05
ILMN_1793829	TMCO1	5.16E-01	3.38E-04	2.58E-03
ILMN_1746588	TALDO1	5.16E-01	3.07E-08	1.01E-06
ILMN_1791006	AHI1	5.16E-01	1.73E-04	1.44E-03
ILMN_1741392	SLC25A20	5.16E-01	7.84E-07	1.46E-05
ILMN_1691480	LONP2	5.16E-01	1.92E-08	7.03E-07
ILMN_1691276	CXXC1	5.16E-01	9.15E-06	1.17E-04
ILMN_2162234	NEK1	5.16E-01	3.04E-05	3.24E-04

ILMN_2049727	C20orf111	5.15E-01	3.71E-07	7.81E-06
ILMN_2328029	EXT2	5.15E-01	6.77E-05	6.43E-04
ILMN_1749478	TCEAL3	5.14E-01	2.87E-10	2.72E-08
ILMN_1690894	TRA1P2	5.14E-01	6.19E-04	4.34E-03
ILMN_1694219	ARIH1	5.14E-01	3.01E-08	9.94E-07
ILMN_1674620	SGCE	5.14E-01	9.11E-04	6.05E-03
ILMN_1773865	HSPA5	5.14E-01	3.94E-05	4.04E-04
ILMN_1746968	PHF1	5.14E-01	1.22E-06	2.11E-05
ILMN_1757440	FAM69B	5.13E-01	4.36E-07	8.87E-06
ILMN_3243682	C1orf93	5.13E-01	6.96E-08	1.94E-06
ILMN_1774066	TMEM141	5.13E-01	1.79E-03	1.08E-02
ILMN_1679520	AGPAT1	5.13E-01	1.31E-07	3.30E-06
ILMN_1808501	SH3KBP1	5.12E-01	3.44E-03	1.88E-02
ILMN_1695435	LOC653610	5.12E-01	1.25E-04	1.08E-03
ILMN_1705985	PIGA	5.12E-01	1.23E-06	2.12E-05
ILMN_1798395	PIGH	5.12E-01	2.61E-07	5.84E-06
ILMN_1808500	CEP68	5.12E-01	5.52E-06	7.63E-05
ILMN_2089175	SYAP1	5.12E-01	3.50E-05	3.65E-04
ILMN_1762316	CPSF3L	5.11E-01	1.74E-05	2.02E-04
ILMN_1800993	CLUAP1	5.11E-01	2.79E-09	1.51E-07
ILMN_1682206	GCC1	5.11E-01	6.99E-04	4.81E-03
ILMN_1741204	KLHDC2	5.11E-01	4.32E-07	8.80E-06
ILMN_1680757	LRRC26	5.11E-01	2.17E-05	2.43E-04
ILMN_1761721	VPS35	5.11E-01	2.06E-08	7.41E-07
ILMN_1666546	DUSP14	5.11E-01	5.72E-05	5.54E-04
ILMN_1755221	LMAN2L	5.10E-01	4.81E-05	4.78E-04
ILMN_1726755	COPS4	5.10E-01	6.08E-07	1.17E-05
ILMN_1708502	AFF4	5.10E-01	4.16E-05	4.22E-04
ILMN_2364376	ILK	5.10E-01	5.02E-04	3.63E-03
ILMN_2055700	SLBP	5.09E-01	2.18E-04	1.76E-03
ILMN_1677043	AKR7A2	5.09E-01	5.96E-04	4.21E-03
ILMN_1799765	RAB24	5.09E-01	3.36E-08	1.09E-06
ILMN_1741003	ANXA5	5.09E-01	3.46E-07	7.36E-06
ILMN_1753370	ABTB2	5.09E-01	5.14E-04	3.70E-03
ILMN_1810441	TRIM3	5.09E-01	6.16E-05	5.91E-04
ILMN_1767509	DEF8	5.09E-01	1.67E-03	1.01E-02
ILMN_1651429	SELM	5.09E-01	1.37E-06	2.31E-05
ILMN_1675956	LYST	5.09E-01	3.40E-08	1.10E-06
ILMN_1726565	PIK3R2	5.08E-01	1.34E-06	2.26E-05
ILMN_1749792	SORBS1	5.08E-01	1.08E-05	1.34E-04
ILMN_2289093	KIAA1618	5.08E-01	8.83E-07	1.61E-05
ILMN_1807136	LOC729559	5.08E-01	6.47E-08	1.83E-06
ILMN_3229770	SKP1	5.07E-01	9.47E-06	1.20E-04
ILMN_1790317	RAB26	5.07E-01	3.36E-03	1.85E-02
ILMN_1738491	SNX30	5.07E-01	2.54E-09	1.41E-07
ILMN_1656452	C16orf59	5.07E-01	3.88E-03	2.09E-02

ILMN_1673553	PTH2	5.06E-01	2.79E-04	2.19E-03
ILMN_2220187	GFPT1	5.06E-01	2.28E-05	2.54E-04
ILMN_2313901	PAM	5.06E-01	6.82E-06	9.12E-05
ILMN_2397776	ASB6	5.06E-01	3.70E-08	1.18E-06
ILMN_1723141	OTUD1	5.06E-01	3.86E-08	1.22E-06
ILMN_1736103	ITPR2	5.06E-01	5.90E-06	8.07E-05
ILMN_1815366	FLJ21865	5.06E-01	3.12E-03	1.73E-02
ILMN_1755173	PLEKHA4	5.05E-01	3.59E-03	1.95E-02
ILMN_1778876	FAM179B	5.05E-01	1.14E-06	1.99E-05
ILMN_1755710	EFNA4	5.05E-01	1.93E-12	8.42E-10
ILMN_1728699	SPTY2D1	5.05E-01	1.81E-06	2.93E-05
ILMN_1696974	ANG	5.04E-01	5.13E-06	7.18E-05
ILMN_1666924	PINK1	5.04E-01	4.48E-09	2.20E-07
ILMN_1686562	KIF13B	5.04E-01	4.02E-04	3.00E-03
ILMN_1697652	PLEKHB2	5.04E-01	4.17E-05	4.22E-04
ILMN_1704351	WDR25	5.04E-01	1.55E-08	5.89E-07
ILMN_1781039	VPS26	5.04E-01	1.36E-05	1.63E-04
ILMN_2209027	RPS26	5.03E-01	3.82E-06	5.61E-05
ILMN_3245471	SCARNA20	5.03E-01	5.67E-03	2.88E-02
ILMN_1736256	CALR	5.03E-01	2.31E-08	8.04E-07
ILMN_1777915	STX6	5.03E-01	1.43E-06	2.40E-05
ILMN_1760718	ZMIZ2	5.03E-01	7.31E-03	3.57E-02
ILMN_2347349	CCNB1IP1	5.03E-01	7.57E-06	9.94E-05
ILMN_1773901	STX12	5.03E-01	8.22E-04	5.53E-03
ILMN_1729112	CHPT1	5.02E-01	5.64E-04	4.00E-03
ILMN_1772241	SQLE	5.02E-01	7.38E-08	2.05E-06
ILMN_1796464	WDR37	5.02E-01	3.29E-05	3.47E-04
ILMN_1771139	FBXO31	5.02E-01	4.09E-04	3.05E-03
ILMN_1655485	C1orf124	5.02E-01	7.36E-07	1.38E-05
ILMN_2373791	ENPP2	5.02E-01	9.84E-09	4.10E-07
ILMN_2117716	SFRS17A	5.02E-01	1.79E-10	1.88E-08
ILMN_2051232	SDHA	5.01E-01	3.27E-05	3.45E-04
ILMN_1745112	FAM102A	5.01E-01	8.47E-07	1.55E-05
ILMN_1781761	ENPP4	5.01E-01	1.60E-05	1.88E-04
ILMN_1689004	TNFRSF12A	5.01E-01	1.32E-04	1.14E-03
ILMN_1798346	KIAA1468	5.00E-01	8.89E-08	2.40E-06
ILMN_1684964	ZNF212	5.00E-01	5.17E-04	3.72E-03
ILMN_1661363	CDC14B	5.00E-01	3.76E-05	3.88E-04
ILMN_2352036	RTN4	5.00E-01	6.34E-04	4.44E-03
ILMN_2348090	MRPL55	5.00E-01	4.54E-08	1.38E-06
ILMN_1789243	VPS33B	5.00E-01	9.49E-10	6.56E-08
ILMN_1796165	GLRX5	4.99E-01	7.61E-08	2.10E-06
ILMN_1776788	C5orf41	4.99E-01	2.05E-08	7.40E-07
ILMN_2322842	PPHLN1	4.99E-01	6.49E-04	4.52E-03
ILMN_1704056	RPPH1	4.99E-01	2.30E-08	8.03E-07
ILMN_1693311	TMBIM6	4.99E-01	4.05E-04	3.02E-03

ILMN_2096116	HSP90B1	4.99E-01	2.44E-03	1.41E-02
ILMN_1782459	OSBPL8	4.98E-01	2.16E-06	3.40E-05
ILMN_1662359	HIST1H4K	4.98E-01	6.09E-05	5.85E-04
ILMN_1786396	ZZEF1	4.98E-01	3.42E-06	5.10E-05
ILMN_1811264	C15orf57	4.98E-01	1.77E-09	1.06E-07
ILMN_2379063	NDRG4	4.98E-01	6.97E-03	3.43E-02
ILMN_1739987	KCNH2	4.98E-01	2.11E-08	7.52E-07
ILMN_1737308	GLRX	4.98E-01	1.58E-06	2.62E-05
ILMN_1756935	OSBPL6	4.97E-01	9.82E-05	8.81E-04
ILMN_1702447	IGF2BP2	4.97E-01	2.90E-04	2.26E-03
ILMN_2163070	KHDC1	4.97E-01	3.86E-05	3.96E-04
ILMN_1695000	TFIP11	4.97E-01	8.63E-06	1.11E-04
ILMN_1759549	SRGAP2	4.97E-01	7.04E-04	4.85E-03
ILMN_1813669	ANKS1A	4.97E-01	9.64E-10	6.64E-08
ILMN_1725130	FAM50A	4.97E-01	4.79E-07	9.64E-06
ILMN_1765796	ENO2	4.96E-01	1.84E-03	1.10E-02
ILMN_1681984	GALNT10	4.96E-01	1.21E-05	1.48E-04
ILMN_2399523	JAG2	4.95E-01	1.17E-05	1.45E-04
ILMN_1743714	CARD10	4.95E-01	1.44E-05	1.70E-04
ILMN_1656368	ALDH4A1	4.95E-01	4.76E-04	3.47E-03
ILMN_1769787	SELO	4.95E-01	2.63E-06	4.04E-05
ILMN_2395375	GABBR1	4.95E-01	7.23E-06	9.58E-05
ILMN_1761259	EXT2	4.95E-01	2.05E-05	2.32E-04
ILMN_2049642	RPA1	4.95E-01	2.12E-07	4.94E-06
ILMN_3251634	SENP5	4.95E-01	5.43E-03	2.78E-02
ILMN_2395240	CHEK2	4.95E-01	3.11E-04	2.41E-03
ILMN_1753243	DNAJB11	4.94E-01	1.18E-07	3.04E-06
ILMN_1690963	ASAP1	4.94E-01	3.85E-08	1.21E-06
ILMN_2184884	FAM154B	4.94E-01	3.17E-06	4.76E-05
ILMN_1694708	LOC650737	4.93E-01	2.49E-03	1.43E-02
ILMN_1790471	CICE	4.93E-01	1.14E-05	1.41E-04
ILMN_3231944	LOC100130516	4.93E-01	1.33E-04	1.15E-03
ILMN_1742611	C1orf52	4.93E-01	1.21E-06	2.08E-05
ILMN_1758315	SLC9A9	4.92E-01	2.46E-07	5.56E-06
ILMN_1681542	HIST1H4E	4.92E-01	4.57E-03	2.40E-02
ILMN_3197097	TSTD1	4.92E-01	3.57E-08	1.14E-06
ILMN_1746408	MIDN	4.92E-01	9.41E-08	2.52E-06
ILMN_1654586	RASA3	4.92E-01	1.33E-10	1.54E-08
ILMN_1759464	C1orf124	4.92E-01	2.67E-03	1.52E-02
ILMN_2202940	CHPT1	4.91E-01	3.59E-05	3.73E-04
ILMN_1788701	PSIP1	4.91E-01	7.12E-07	1.34E-05
ILMN_1699623	FAM81A	4.91E-01	9.85E-04	6.45E-03
ILMN_1813503	SEMA6C	4.91E-01	6.59E-09	2.98E-07
ILMN_1813796	TMEM169	4.91E-01	1.12E-07	2.90E-06
ILMN_1686805	CRK	4.90E-01	3.58E-05	3.72E-04
ILMN_1689180	LOC644390	4.90E-01	2.29E-08	7.99E-07

ILMN_3233388	RELL1	4.90E-01	5.27E-07	1.05E-05
ILMN_1722945	C6orf52	4.89E-01	1.20E-06	2.08E-05
ILMN_1666096	ACSL3	4.89E-01	6.18E-09	2.84E-07
ILMN_1773059	GPR124	4.89E-01	6.65E-10	5.11E-08
ILMN_1737965	ELOVL4	4.89E-01	5.90E-04	4.17E-03
ILMN_1695745	DISP1	4.89E-01	4.58E-09	2.24E-07
ILMN_1734346	C19orf36	4.89E-01	1.04E-08	4.27E-07
ILMN_1892638		4.89E-01	2.40E-07	5.44E-06
ILMN_3235216	IFT20	4.89E-01	1.15E-07	2.98E-06
ILMN_1697694	ATP6AP1	4.89E-01	5.04E-06	7.08E-05
ILMN_2415170	VPS8	4.89E-01	5.52E-08	1.62E-06
ILMN_2403047	ARL13B	4.88E-01	1.19E-05	1.46E-04
ILMN_3290211	LOC644761	4.88E-01	2.05E-06	3.25E-05
ILMN_1718013	CPSF3L	4.88E-01	2.01E-05	2.28E-04
ILMN_1711919	SCYL2	4.87E-01	4.19E-05	4.24E-04
ILMN_1743911	SLC25A39	4.87E-01	5.71E-05	5.54E-04
ILMN_1758673	SLC44A1	4.87E-01	4.58E-08	1.39E-06
ILMN_1745130	RBM9	4.87E-01	6.19E-06	8.41E-05
ILMN_3238058	LOC151162	4.87E-01	3.57E-04	2.70E-03
ILMN_1806758	C9orf85	4.87E-01	1.12E-04	9.89E-04
ILMN_1720482	CEND1	4.87E-01	2.88E-04	2.25E-03
ILMN_1893511		4.86E-01	8.95E-06	1.15E-04
ILMN_1773313	USMG5	4.86E-01	6.55E-03	3.25E-02
ILMN_1772981	EPN1	4.86E-01	8.38E-07	1.54E-05
ILMN_1670439	FYTTD1	4.86E-01	1.86E-05	2.14E-04
ILMN_2345142	SULF2	4.86E-01	4.88E-03	2.54E-02
ILMN_2364529	EZH2	4.86E-01	4.48E-05	4.49E-04
ILMN_2338480	RHOT1	4.86E-01	3.44E-04	2.62E-03
ILMN_1678757	BCYRN1	4.86E-01	3.18E-04	2.45E-03
ILMN_1781691	TRAK2	4.85E-01	1.94E-05	2.21E-04
ILMN_2119945	NDUFB3	4.85E-01	4.08E-07	8.44E-06
ILMN_1662438	SOD1	4.85E-01	6.22E-05	5.97E-04
ILMN_1752793	SAP18	4.85E-01	3.94E-05	4.03E-04
ILMN_1764207	RPRD1A	4.84E-01	3.53E-05	3.67E-04
ILMN_1674160	BIN1	4.84E-01	1.74E-06	2.84E-05
ILMN_1816244		4.83E-01	9.02E-05	8.21E-04
ILMN_3307877	C21orf58	4.83E-01	1.48E-03	9.12E-03
ILMN_1689389	SF3B5	4.83E-01	3.25E-07	7.02E-06
ILMN_1672759	CCDC109A	4.82E-01	6.22E-05	5.97E-04
ILMN_1743316	FAM109A	4.82E-01	4.37E-08	1.34E-06
ILMN_1673275	TRAPPC2	4.82E-01	2.09E-03	1.23E-02
ILMN_1746716	LOC643035	4.82E-01	3.18E-08	1.04E-06
ILMN_1668619	KIAA1467	4.82E-01	1.02E-09	6.94E-08
ILMN_1686623	CSF1R	4.82E-01	9.48E-05	8.57E-04
ILMN_2400292	MAPK9	4.82E-01	6.32E-07	1.21E-05
ILMN_1683234	DNAJC1	4.81E-01	3.60E-08	1.15E-06

ILMN_1715024	LSS	4.81E-01	8.46E-06	1.09E-04
ILMN_2120340	RUVBL2	4.81E-01	1.17E-03	7.46E-03
ILMN_1776582	PDK3	4.81E-01	3.60E-09	1.85E-07
ILMN_1651710	IQCD	4.81E-01	1.48E-06	2.47E-05
ILMN_2094952	NUAK2	4.80E-01	7.12E-05	6.71E-04
ILMN_1751607	FOSB	4.80E-01	3.21E-05	3.40E-04
ILMN_1669905	DCP2	4.80E-01	7.15E-06	9.49E-05
ILMN_1712751	HADHA	4.80E-01	2.89E-08	9.63E-07
ILMN_1758633	CCDC130	4.80E-01	4.36E-05	4.39E-04
ILMN_1778734	MTMR10	4.80E-01	4.71E-06	6.67E-05
ILMN_1742731	SLC35A2	4.80E-01	2.55E-07	5.71E-06
ILMN_2360784	RRBP1	4.79E-01	3.81E-05	3.92E-04
ILMN_1811615	COPA	4.79E-01	3.46E-06	5.16E-05
ILMN_1765032	LOC440993	4.79E-01	6.37E-05	6.09E-04
ILMN_1708006	MICB	4.79E-01	3.38E-04	2.58E-03
ILMN_1785252	SLC26A6	4.79E-01	1.22E-08	4.85E-07
ILMN_1673896	MAP3K13	4.79E-01	8.13E-06	1.06E-04
ILMN_1745223	CDC42EP4	4.79E-01	3.64E-09	1.86E-07
ILMN_1692464	FLJ20699	4.79E-01	2.36E-03	1.37E-02
ILMN_1690524	VAMP7	4.79E-01	3.66E-03	1.98E-02
ILMN_1715823	FBXO16	4.79E-01	4.58E-07	9.27E-06
ILMN_1724497	ABI2	4.78E-01	3.25E-08	1.06E-06
ILMN_1656902	HECTD3	4.78E-01	1.79E-07	4.28E-06
ILMN_1718297	EML4	4.78E-01	1.94E-06	3.11E-05
ILMN_1687533	SEMA4D	4.78E-01	2.58E-07	5.76E-06
ILMN_1690695	PEX11A	4.77E-01	1.43E-09	9.05E-08
ILMN_1668629	LOC401115	4.77E-01	5.35E-04	3.83E-03
ILMN_1664369	DHTKD1	4.77E-01	3.92E-04	2.93E-03
ILMN_1724504	SETD3	4.77E-01	7.31E-05	6.86E-04
ILMN_2129927	EXT1	4.76E-01	1.08E-06	1.90E-05
ILMN_1752213	TMEM60	4.76E-01	1.70E-03	1.02E-02
ILMN_2201580	GSTM2	4.76E-01	2.55E-04	2.02E-03
ILMN_1763828	MTF1	4.76E-01	4.66E-06	6.64E-05
ILMN_1733627	NEDD4L	4.76E-01	1.25E-05	1.52E-04
ILMN_2199389	VIPR1	4.76E-01	3.09E-07	6.72E-06
ILMN_1755677	FAM158A	4.76E-01	1.81E-06	2.93E-05
ILMN_1703228	AGFG2	4.75E-01	8.94E-07	1.62E-05
ILMN_2379560	CDC14B	4.75E-01	5.37E-03	2.75E-02
ILMN_1788832	MIB1	4.75E-01	3.09E-03	1.72E-02
ILMN_2249920	FYN	4.75E-01	6.64E-09	2.99E-07
ILMN_2396639	PDLIM7	4.75E-01	3.52E-05	3.66E-04
ILMN_2046315	GABARAPL2	4.75E-01	1.72E-04	1.44E-03
ILMN_1779965	AK1	4.75E-01	1.17E-08	4.71E-07
ILMN_1811195	ZNF211	4.74E-01	2.37E-07	5.40E-06
ILMN_2225735	CRBN	4.74E-01	7.77E-05	7.23E-04
ILMN_3182893	LOC100128163	4.74E-01	8.59E-04	5.75E-03

ILMN_1760890	SEPN1	4.74E-01	1.98E-04	1.62E-03
ILMN_1657283	ALKBH5	4.74E-01	6.49E-05	6.19E-04
ILMN_2119937	NDUFB3	4.74E-01	4.13E-09	2.07E-07
ILMN_2400922	OPRL1	4.74E-01	3.86E-05	3.97E-04
ILMN_1790202	C1orf35	4.74E-01	5.66E-08	1.65E-06
ILMN_1705310	VEZF1	4.74E-01	1.33E-03	8.33E-03
ILMN_3303612	PABPC1L	4.73E-01	3.30E-03	1.82E-02
ILMN_2157951	STX6	4.73E-01	2.50E-06	3.88E-05
ILMN_3247939	SNORA23	4.73E-01	2.69E-07	5.96E-06
ILMN_1708987	HSD11B1L	4.73E-01	5.46E-04	3.90E-03
ILMN_2168347	EPOR	4.73E-01	8.32E-03	3.98E-02
ILMN_1754304	SNRNP48	4.73E-01	8.14E-07	1.50E-05
ILMN_2189458	SHQ1	4.73E-01	3.22E-09	1.70E-07
ILMN_2246661	FAHD1	4.73E-01	5.74E-07	1.12E-05
ILMN_1758311	NET1	4.72E-01	1.85E-03	1.10E-02
ILMN_2399264	SEPT6	4.72E-01	1.19E-03	7.59E-03
ILMN_1743303	TTC1	4.72E-01	1.63E-06	2.68E-05
ILMN_1791949	PGBD1	4.72E-01	6.62E-06	8.88E-05
ILMN_1673478	C5orf5	4.72E-01	8.40E-07	1.54E-05
ILMN_1684591	ZNF434	4.71E-01	5.08E-09	2.42E-07
ILMN_1757631	DBNDD1	4.71E-01	3.16E-04	2.44E-03
ILMN_2323774	RPAIN	4.71E-01	4.94E-05	4.89E-04
ILMN_1721901	CTNNAL1	4.71E-01	1.86E-07	4.42E-06
ILMN_1772814	NLK	4.71E-01	4.97E-06	7.00E-05
ILMN_1705116	C6orf85	4.71E-01	2.29E-06	3.58E-05
ILMN_1805916	NIPSNAP1	4.71E-01	3.18E-08	1.04E-06
ILMN_1726512	ZSCAN2	4.70E-01	4.56E-08	1.39E-06
ILMN_3238751	PMS2L4	4.70E-01	1.21E-05	1.48E-04
ILMN_1801606	AMZ2	4.70E-01	2.10E-07	4.89E-06
ILMN_1814120	PECR	4.70E-01	6.45E-05	6.15E-04
ILMN_1791093	PPHLN1	4.70E-01	5.88E-05	5.68E-04
ILMN_2126344	SEC16A	4.70E-01	8.95E-09	3.78E-07
ILMN_1749011	NECAP2	4.70E-01	1.36E-06	2.30E-05
ILMN_1657701	TMEM137	4.69E-01	2.72E-07	6.02E-06
ILMN_3251451	MED31	4.69E-01	1.58E-07	3.86E-06
ILMN_1709549	PLEKHM1	4.69E-01	1.27E-05	1.54E-04
ILMN_3240187	TMEM111	4.69E-01	1.76E-05	2.04E-04
ILMN_3237511	C4orf47	4.69E-01	1.18E-03	7.54E-03
ILMN_1726786	TNRC6B	4.69E-01	1.07E-03	6.90E-03
ILMN_1685625	UCP2	4.69E-01	7.61E-03	3.69E-02
ILMN_1754332	LOC85389	4.68E-01	1.42E-05	1.68E-04
ILMN_1704164	ATP13A2	4.68E-01	9.25E-06	1.18E-04
ILMN_1810836	PDE5A	4.68E-01	2.49E-07	5.61E-06
ILMN_1653404	NKIRAS2	4.68E-01	3.98E-07	8.26E-06
ILMN_1667519	RRAS2	4.68E-01	6.52E-06	8.77E-05
ILMN_1782292	LAMP1	4.68E-01	8.34E-08	2.27E-06

ILMN_1806908	PRKCB1	4.67E-01	3.83E-04	2.88E-03
ILMN_1713603	PRKCB1	4.67E-01	8.92E-06	1.14E-04
ILMN_2355042	CLUAP1	4.67E-01	1.04E-07	2.75E-06
ILMN_1667162	NKX3-1	4.67E-01	3.88E-06	5.68E-05
ILMN_2382127	PPFIA1	4.67E-01	5.13E-04	3.70E-03
ILMN_1676555	TTC26	4.66E-01	7.60E-06	9.98E-05
ILMN_1745021	SLC30A1	4.66E-01	7.62E-09	3.33E-07
ILMN_1778078	VPS16	4.66E-01	6.55E-06	8.80E-05
ILMN_2153373	LRBA	4.65E-01	1.84E-05	2.11E-04
ILMN_2101034	CAPN12	4.65E-01	1.88E-03	1.12E-02
ILMN_1755504	CALCOCO2	4.65E-01	1.56E-09	9.65E-08
ILMN_2408039	EEF1D	4.65E-01	1.46E-07	3.63E-06
ILMN_1694027	SESN3	4.65E-01	2.25E-07	5.17E-06
ILMN_1675472	LOC644799	4.64E-01	7.33E-04	5.01E-03
ILMN_1777794	PRKCSH	4.64E-01	8.96E-08	2.42E-06
ILMN_1714737	ASF1A	4.64E-01	1.49E-03	9.18E-03
ILMN_1805064	SCARNA9	4.64E-01	1.13E-03	7.28E-03
ILMN_1662587	PNPLA7	4.64E-01	3.46E-09	1.80E-07
ILMN_1738300	SLC4A8	4.64E-01	2.84E-05	3.06E-04
ILMN_1712985	C17orf58	4.64E-01	3.86E-07	8.05E-06
ILMN_1685916	KIF2C	4.63E-01	2.30E-07	5.27E-06
ILMN_2098418	LOC652968	4.63E-01	3.25E-06	4.88E-05
ILMN_1721093	TAF10	4.63E-01	1.63E-04	1.37E-03
ILMN_1777444	STX5	4.63E-01	7.00E-07	1.32E-05
ILMN_1758895	CTSK	4.63E-01	3.91E-10	3.40E-08
ILMN_1724826	DNM1L	4.63E-01	6.09E-05	5.85E-04
ILMN_2405297	NOTCH2	4.63E-01	1.31E-08	5.15E-07
ILMN_1781580	BRI3	4.62E-01	1.41E-05	1.68E-04
ILMN_1743770	SLC25A14	4.62E-01	4.20E-06	6.07E-05
ILMN_2195914	GGH	4.62E-01	4.78E-04	3.48E-03
ILMN_1799128	SLC30A9	4.62E-01	2.63E-04	2.08E-03
ILMN_1778360	PYGB	4.62E-01	4.81E-05	4.78E-04
ILMN_1658995	ACOT9	4.62E-01	1.81E-04	1.50E-03
ILMN_1784113	NAT14	4.62E-01	1.27E-04	1.10E-03
ILMN_1723124	GALK2	4.62E-01	4.16E-07	8.58E-06
ILMN_1706764	GOLPH3L	4.61E-01	8.57E-05	7.86E-04
ILMN_1685854	C5orf53	4.61E-01	4.93E-05	4.88E-04
ILMN_2185845	BRSK1	4.61E-01	1.05E-06	1.86E-05
ILMN_1862217		4.61E-01	6.16E-03	3.09E-02
ILMN_1808226	RGS16	4.61E-01	1.86E-03	1.11E-02
ILMN_1777378	COMMD6	4.61E-01	4.75E-03	2.48E-02
ILMN_1663618	STAT3	4.61E-01	2.85E-04	2.23E-03
ILMN_1714527	VAMP3	4.60E-01	4.29E-08	1.33E-06
ILMN_2193315	C14orf143	4.60E-01	4.17E-05	4.23E-04
ILMN_3290019	LOC646753	4.60E-01	2.13E-04	1.72E-03
ILMN_2129015	AFF1	4.60E-01	2.00E-03	1.18E-02

ILMN_1754842	DLGAP4	4.60E-01	4.56E-08	1.39E-06
ILMN_3296994	LOC728823	4.60E-01	7.35E-04	5.02E-03
ILMN_2415179	CLSTN1	4.60E-01	1.93E-05	2.20E-04
ILMN_1808202	C19orf22	4.59E-01	1.01E-06	1.80E-05
ILMN_1738383	EEF2	4.59E-01	1.23E-08	4.89E-07
ILMN_1735658	RTTN	4.59E-01	1.04E-07	2.75E-06
ILMN_2323944	FAM110A	4.59E-01	1.86E-04	1.54E-03
ILMN_1696419	STOM	4.59E-01	6.79E-07	1.29E-05
ILMN_2366388	PRDX1	4.59E-01	5.35E-08	1.58E-06
ILMN_1653165	AAMP	4.59E-01	9.16E-06	1.17E-04
ILMN_1726169	EDF1	4.58E-01	3.82E-06	5.62E-05
ILMN_1676631	CCNO	4.58E-01	5.46E-07	1.08E-05
ILMN_1705602	KLHL17	4.58E-01	2.39E-05	2.64E-04
ILMN_1701940	FAM73B	4.58E-01	9.33E-05	8.44E-04
ILMN_2412294	GNB5	4.58E-01	1.06E-07	2.80E-06
ILMN_1765109	TNFRSF25	4.58E-01	1.04E-04	9.28E-04
ILMN_1764770	MGC15763	4.57E-01	7.97E-03	3.84E-02
ILMN_1750549	PI4K2A	4.57E-01	1.83E-08	6.73E-07
ILMN_2307025	CPNE1	4.57E-01	2.81E-07	6.19E-06
ILMN_2128639	C10orf47	4.57E-01	1.02E-02	4.72E-02
ILMN_1813671	SLC25A1	4.57E-01	5.71E-05	5.54E-04
ILMN_1773119	CCNF	4.56E-01	1.16E-05	1.43E-04
ILMN_1706734	ZNF451	4.56E-01	5.59E-09	2.60E-07
ILMN_1779530	COG6	4.56E-01	7.13E-04	4.89E-03
ILMN_1711904	MXD3	4.56E-01	5.64E-05	5.49E-04
ILMN_2047112	RP11-529I10.4	4.56E-01	1.95E-03	1.15E-02
ILMN_1701457	FAHD1	4.56E-01	6.52E-07	1.25E-05
ILMN_3251246	PIGH	4.56E-01	1.20E-04	1.05E-03
ILMN_2359601	CAMK2G	4.55E-01	5.68E-08	1.65E-06
ILMN_1793770	DNAJB6	4.55E-01	1.83E-03	1.10E-02
ILMN_1654518	LRCH4	4.55E-01	6.77E-06	9.06E-05
ILMN_2343310	DYNC2H1	4.55E-01	3.55E-07	7.52E-06
ILMN_1683923	MT1H	4.55E-01	1.27E-06	2.17E-05
ILMN_1729161	NOTCH1	4.55E-01	8.27E-04	5.56E-03
ILMN_1665192	NUDT6	4.55E-01	4.59E-09	2.24E-07
ILMN_1702633	RETSAT	4.55E-01	6.98E-05	6.59E-04
ILMN_3231820	SIVA1	4.55E-01	3.49E-05	3.64E-04
ILMN_3245678	RNU1A3	4.54E-01	6.46E-03	3.21E-02
ILMN_1663042	SDC4	4.54E-01	1.48E-03	9.11E-03
ILMN_1738237	HS1BP3	4.54E-01	6.23E-08	1.78E-06
ILMN_2356654	LGALS8	4.54E-01	1.73E-07	4.16E-06
ILMN_1807201	FAM104A	4.54E-01	6.37E-08	1.81E-06
ILMN_1681603	UBE2A	4.54E-01	1.57E-05	1.84E-04
ILMN_2395932	UNC45A	4.54E-01	3.93E-04	2.94E-03
ILMN_1789410	ZSCAN21	4.54E-01	5.00E-05	4.94E-04
ILMN_2152711	ACVR2A	4.53E-01	4.75E-04	3.46E-03

ILMN_1663313	AMY1C	4.53E-01	9.75E-03	4.55E-02
ILMN_1713993	UBAC2	4.53E-01	4.57E-06	6.53E-05
ILMN_1689525	PMAIP1	4.53E-01	6.62E-04	4.60E-03
ILMN_1680579	ATP2B4	4.53E-01	4.69E-09	2.28E-07
ILMN_1763208	ZNF10	4.53E-01	2.59E-05	2.82E-04
ILMN_2131392	WDR70	4.52E-01	6.95E-06	9.25E-05
ILMN_1668582	CRBN	4.52E-01	1.81E-05	2.08E-04
ILMN_1803743	LOC196752	4.52E-01	2.00E-05	2.27E-04
ILMN_1721621	NKTR	4.52E-01	2.70E-07	5.99E-06
ILMN_3227994	LOC729992	4.52E-01	5.16E-08	1.53E-06
ILMN_3242077	LOC648742	4.51E-01	1.15E-06	2.01E-05
ILMN_2352921	BPGM	4.51E-01	7.26E-07	1.36E-05
ILMN_2050813	CLGN	4.51E-01	3.75E-06	5.53E-05
ILMN_1800942	KCTD6	4.51E-01	5.56E-07	1.09E-05
ILMN_1715569	CCDC53	4.51E-01	1.23E-05	1.50E-04
ILMN_2294978	RNASE4	4.51E-01	8.70E-05	7.95E-04
ILMN_1716488	PACS1	4.50E-01	1.65E-06	2.71E-05
ILMN_1660654	CDCA2	4.50E-01	1.46E-05	1.73E-04
ILMN_1780444	ARL3	4.50E-01	4.33E-05	4.37E-04
ILMN_2077623	RRAS2	4.50E-01	2.62E-05	2.86E-04
ILMN_1727332	ATPIF1	4.49E-01	3.39E-05	3.55E-04
ILMN_2411236	NRCAM	4.49E-01	1.43E-07	3.57E-06
ILMN_1666634	FAF1	4.49E-01	6.53E-06	8.77E-05
ILMN_2173919	MYO9A	4.49E-01	1.42E-06	2.39E-05
ILMN_2117171	LMO4	4.49E-01	9.76E-05	8.77E-04
ILMN_2385220	DFFA	4.49E-01	1.69E-06	2.77E-05
ILMN_1741727	QPCT	4.49E-01	6.64E-03	3.29E-02
ILMN_1706273	HCCA2	4.49E-01	3.26E-05	3.44E-04
ILMN_2294976	RNASE4	4.48E-01	1.60E-05	1.88E-04
ILMN_1775036	SEC22A	4.48E-01	1.11E-05	1.37E-04
ILMN_1772124	ATRN	4.48E-01	2.27E-09	1.29E-07
ILMN_1771822	ARL6	4.48E-01	1.25E-05	1.52E-04
ILMN_1662852	IQCK	4.47E-01	7.70E-05	7.17E-04
ILMN_1670145	DFNA5	4.47E-01	1.08E-02	4.97E-02
ILMN_2093231	WBP1	4.47E-01	2.83E-07	6.22E-06
ILMN_1719392	FH	4.47E-01	2.02E-03	1.20E-02
ILMN_2222074	PTPN12	4.47E-01	4.00E-07	8.30E-06
ILMN_2200331	H2AFX	4.47E-01	7.14E-06	9.48E-05
ILMN_1726359	NECAP1	4.47E-01	1.02E-06	1.81E-05
ILMN_1662129	RCN2	4.46E-01	9.34E-06	1.19E-04
ILMN_1685781	C14orf142	4.46E-01	1.88E-06	3.03E-05
ILMN_3237270	LOC100133609	4.46E-01	4.02E-08	1.26E-06
ILMN_1792986	RFC1	4.46E-01	1.76E-06	2.87E-05
ILMN_1748407	CCS	4.46E-01	1.35E-08	5.25E-07
ILMN_1657950	RPS26P10	4.46E-01	6.58E-04	4.57E-03
ILMN_3240389	CPOX	4.46E-01	9.47E-06	1.20E-04

ILMN_1709936	LOC90624	4.46E-01	1.05E-03	6.82E-03
ILMN_1721868	KPNA2	4.46E-01	1.43E-03	8.82E-03
ILMN_1756542	TBC1D17	4.45E-01	8.25E-04	5.55E-03
ILMN_3285959	LOC645515	4.45E-01	1.14E-10	1.40E-08
ILMN_1666399	RING1	4.45E-01	3.62E-05	3.75E-04
ILMN_2309245	BIN1	4.45E-01	3.25E-05	3.44E-04
ILMN_1802292	WDFY2	4.45E-01	1.70E-04	1.42E-03
ILMN_1691575	SNX2	4.45E-01	1.18E-06	2.05E-05
ILMN_1856480		4.45E-01	2.18E-04	1.76E-03
ILMN_1678546	PEX11B	4.44E-01	4.59E-04	3.36E-03
ILMN_2379520	HAGH	4.44E-01	5.86E-05	5.67E-04
ILMN_3239240	POLR2E	4.44E-01	8.63E-08	2.34E-06
ILMN_1690844	LOC387820	4.44E-01	2.58E-05	2.82E-04
ILMN_1765409	STAM	4.44E-01	1.28E-03	8.05E-03
ILMN_1808783	STRBP	4.44E-01	8.94E-04	5.94E-03
ILMN_1764522	LMBR1	4.44E-01	3.27E-04	2.52E-03
ILMN_1701308	COL1A1	4.44E-01	1.89E-05	2.16E-04
ILMN_2209993	PLIN5	4.44E-01	6.93E-03	3.41E-02
ILMN_1783798	GAS8	4.43E-01	1.45E-08	5.57E-07
ILMN_1658885	DAGLB	4.43E-01	1.38E-07	3.45E-06
ILMN_1811933	SHMT1	4.43E-01	1.38E-05	1.65E-04
ILMN_1696432	IDH1	4.43E-01	5.22E-07	1.04E-05
ILMN_2107184	SNRK	4.43E-01	4.44E-04	3.26E-03
ILMN_1772218	HLA-DPA1	4.43E-01	2.91E-08	9.66E-07
ILMN_1655915	MMP11	4.42E-01	4.79E-08	1.44E-06
ILMN_3235584	TRIM66	4.42E-01	2.57E-08	8.73E-07
ILMN_1685551	CHD4	4.42E-01	2.88E-07	6.31E-06
ILMN_1765132	LACTB	4.42E-01	5.42E-06	7.51E-05
ILMN_1688103	CTNNBIP1	4.42E-01	2.22E-09	1.27E-07
ILMN_1659415	MAP2K1IP1	4.42E-01	1.36E-03	8.47E-03
ILMN_1801175	MAP2K3	4.42E-01	3.39E-06	5.06E-05
ILMN_1776216	MMGT1	4.42E-01	3.27E-08	1.06E-06
ILMN_2324375	CHCHD7	4.42E-01	2.34E-04	1.88E-03
ILMN_1720303	OSTM1	4.41E-01	3.33E-09	1.75E-07
ILMN_1662640	C20orf127	4.41E-01	2.80E-03	1.58E-02
ILMN_2193980	ABCB6	4.41E-01	1.53E-05	1.81E-04
ILMN_1715131	CCR7	4.41E-01	4.03E-03	2.16E-02
ILMN_1702496	LOC441461	4.41E-01	1.84E-05	2.11E-04
ILMN_1917290		4.41E-01	1.03E-05	1.29E-04
ILMN_3251620	JMY	4.41E-01	1.32E-04	1.14E-03
ILMN_3244395	LOC728877	4.41E-01	3.57E-07	7.56E-06
ILMN_1705390	KLHL22	4.40E-01	3.57E-03	1.94E-02
ILMN_1780057	RENBP	4.40E-01	1.12E-05	1.38E-04
ILMN_1779343	SNCB	4.40E-01	1.96E-04	1.61E-03
ILMN_2402416	DNAJB6	4.40E-01	1.84E-04	1.52E-03
ILMN_1810805	HEATR5B	4.40E-01	9.55E-07	1.72E-05

ILMN_1716524	RAB7A	4.40E-01	2.35E-05	2.60E-04
ILMN_1655572	C6orf59	4.40E-01	1.97E-04	1.61E-03
ILMN_1680928	DNM3	4.40E-01	1.14E-07	2.96E-06
ILMN_1793458	LOC652668	4.39E-01	2.24E-05	2.50E-04
ILMN_2275098	DTX2	4.39E-01	7.50E-06	9.87E-05
ILMN_2329914	SPRY1	4.39E-01	1.95E-06	3.12E-05
ILMN_1749641	FBXO3	4.39E-01	1.20E-06	2.08E-05
ILMN_1693830	LACTB	4.39E-01	2.71E-07	6.00E-06
ILMN_1696270	PLAG1	4.39E-01	2.23E-05	2.49E-04
ILMN_1812403	BCAP31	4.39E-01	1.75E-05	2.03E-04
ILMN_3248707	KCNQ1OT1	4.39E-01	1.98E-04	1.62E-03
ILMN_3307742	CAPS2	4.38E-01	7.53E-04	5.12E-03
ILMN_1725260	CDC25C	4.38E-01	2.12E-04	1.72E-03
ILMN_1654064	LOC644334	4.38E-01	2.25E-06	3.52E-05
ILMN_1671839	TAF1C	4.38E-01	3.38E-07	7.23E-06
ILMN_2317463	INTS1	4.38E-01	8.71E-07	1.59E-05
ILMN_1678730	NOMO1	4.37E-01	4.32E-05	4.36E-04
ILMN_1743207	LOC391692	4.37E-01	4.85E-08	1.46E-06
ILMN_1671554	LPIN1	4.37E-01	5.85E-06	8.01E-05
ILMN_1747197	SLC41A2	4.37E-01	8.28E-07	1.52E-05
ILMN_1752897	RPL23AP13	4.37E-01	9.92E-09	4.12E-07
ILMN_1665909	LASP1	4.37E-01	1.66E-03	1.01E-02
ILMN_1754279	FBXW7	4.37E-01	6.35E-06	8.58E-05
ILMN_3208330	LOC100132797	4.36E-01	6.05E-07	1.17E-05
ILMN_1701558	MAP1A	4.36E-01	5.64E-09	2.62E-07
ILMN_2156267	EIF2AK1	4.36E-01	5.09E-06	7.13E-05
ILMN_2112599	C16orf80	4.36E-01	1.09E-04	9.66E-04
ILMN_1696494	CMTM6	4.36E-01	1.58E-09	9.68E-08
ILMN_3248833	LOC644928	4.36E-01	1.90E-04	1.56E-03
ILMN_1665945	ACBD3	4.36E-01	5.60E-05	5.45E-04
ILMN_1732516	KNTC1	4.35E-01	2.28E-06	3.57E-05
ILMN_1752394	CCNB1IP1	4.35E-01	1.55E-04	1.31E-03
ILMN_1728698	GDE1	4.35E-01	5.13E-09	2.43E-07
ILMN_1682180	VCPIP1	4.35E-01	5.47E-05	5.34E-04
ILMN_1698677	C4orf27	4.34E-01	1.90E-05	2.17E-04
ILMN_1665455	DCUN1D3	4.34E-01	9.40E-04	6.21E-03
ILMN_1714990	DBT	4.34E-01	3.37E-05	3.53E-04
ILMN_2080158	FAM10A7	4.34E-01	7.31E-04	5.01E-03
ILMN_1797522	DUSP3	4.34E-01	7.58E-03	3.68E-02
ILMN_1700515	C17orf58	4.34E-01	6.16E-05	5.91E-04
ILMN_1732080	SUMO1P3	4.33E-01	1.62E-04	1.36E-03
ILMN_1655365	C2orf77	4.33E-01	1.25E-03	7.87E-03
ILMN_1787378	ADD3	4.33E-01	1.80E-04	1.49E-03
ILMN_1752520	SLFN11	4.32E-01	7.51E-06	9.88E-05
ILMN_1740160	PLCG1	4.32E-01	8.31E-04	5.58E-03
ILMN_2164242	UBE2F	4.32E-01	5.49E-05	5.36E-04

ILMN_2367782	STARD7	4.32E-01	1.25E-05	1.52E-04
ILMN_1743836	MXRA7	4.32E-01	4.22E-04	3.12E-03
ILMN_2094587	USP8	4.32E-01	5.63E-04	4.00E-03
ILMN_2078697	ALPK1	4.31E-01	5.89E-03	2.98E-02
ILMN_1654217	MPP2	4.31E-01	7.08E-03	3.47E-02
ILMN_1804822	SRXN1	4.31E-01	1.49E-04	1.26E-03
ILMN_2329114	COLQ	4.31E-01	1.19E-07	3.06E-06
ILMN_1698243	C1orf85	4.31E-01	2.53E-07	5.67E-06
ILMN_1669592	MARCH2	4.31E-01	8.86E-04	5.90E-03
ILMN_2307266	ARL6	4.31E-01	1.87E-06	3.01E-05
ILMN_1670841	CPNE1	4.31E-01	2.31E-08	8.05E-07
ILMN_3194248	LOC100129539	4.31E-01	1.15E-06	2.00E-05
ILMN_1659599	ADC	4.31E-01	3.75E-07	7.87E-06
ILMN_1671221	GAPVD1	4.30E-01	1.03E-08	4.25E-07
ILMN_1762312	FOXRED1	4.30E-01	7.17E-03	3.51E-02
ILMN_1794492	HOXC6	4.30E-01	8.86E-06	1.13E-04
ILMN_1733366	MAST1	4.30E-01	3.57E-05	3.71E-04
ILMN_1741371	TMEM8	4.30E-01	6.85E-07	1.30E-05
ILMN_1664265	EPHA1	4.30E-01	1.25E-04	1.09E-03
ILMN_1768202	ANKRD24	4.30E-01	7.14E-03	3.50E-02
ILMN_1707434	LOC653778	4.30E-01	9.24E-03	4.35E-02
ILMN_2327276	STAU1	4.29E-01	8.93E-05	8.14E-04
ILMN_1745533	FAM117A	4.29E-01	1.51E-06	2.51E-05
ILMN_1686152	GGA2	4.29E-01	8.08E-07	1.49E-05
ILMN_1724062	LIN54	4.29E-01	3.57E-07	7.56E-06
ILMN_1873107		4.29E-01	1.27E-06	2.17E-05
ILMN_3231558	LOC100134648	4.28E-01	1.27E-07	3.24E-06
ILMN_1664698	UNC119	4.28E-01	5.77E-05	5.59E-04
ILMN_1741881	C9orf72	4.28E-01	3.49E-06	5.19E-05
ILMN_1805104	ABAT	4.28E-01	1.42E-04	1.22E-03
ILMN_3237665	COX7A2L	4.28E-01	1.02E-06	1.82E-05
ILMN_2299612	TMEM150A	4.28E-01	3.55E-05	3.69E-04
ILMN_2152581	STK38	4.28E-01	1.95E-06	3.11E-05
ILMN_2043452	FANCE	4.27E-01	2.72E-03	1.54E-02
ILMN_1730879	CBY1	4.27E-01	1.61E-04	1.35E-03
ILMN_2395236	CHEK2	4.27E-01	8.14E-08	2.23E-06
ILMN_1712432	PSMD2	4.27E-01	8.43E-07	1.55E-05
ILMN_1742069	ZSWIM5	4.27E-01	6.53E-03	3.24E-02
ILMN_1763036	CLCN6	4.27E-01	5.93E-03	3.00E-02
ILMN_2258543	PRDM2	4.26E-01	8.00E-03	3.85E-02
ILMN_3242288	LOC641768	4.26E-01	7.46E-03	3.63E-02
ILMN_1755910	LOC648366	4.26E-01	6.03E-08	1.73E-06
ILMN_1661000	RPS6KC1	4.25E-01	3.83E-06	5.62E-05
ILMN_1761159	ESYT1	4.25E-01	9.85E-03	4.59E-02
ILMN_1770084	TACC1	4.25E-01	6.00E-08	1.73E-06
ILMN_2385688	RABL2B	4.24E-01	8.38E-08	2.28E-06

ILMN_1879326		4.24E-01	7.76E-05	7.22E-04
ILMN_1784300	TUBA4A	4.24E-01	2.44E-04	1.95E-03
ILMN_2196569	NUP93	4.24E-01	2.38E-05	2.63E-04
ILMN_3202024	LOC392437	4.24E-01	5.08E-05	5.00E-04
ILMN_1656165	USP9X	4.23E-01	9.93E-07	1.78E-05
ILMN_1779071	FEZ1	4.23E-01	3.35E-03	1.84E-02
ILMN_1694491	CCNG1	4.23E-01	8.99E-05	8.18E-04
ILMN_1761058	ACAD11	4.23E-01	3.95E-05	4.04E-04
ILMN_1728802	SDCCAG8	4.23E-01	2.47E-07	5.57E-06
ILMN_2175114	KCNS3	4.22E-01	3.55E-03	1.93E-02
ILMN_1758232	GGCX	4.22E-01	2.88E-07	6.31E-06
ILMN_2065022	KIAA0672	4.22E-01	2.29E-07	5.26E-06
ILMN_1686985	MTM1	4.22E-01	2.33E-05	2.59E-04
ILMN_1703092	RECQL4	4.22E-01	3.36E-03	1.85E-02
ILMN_1750400	C19orf66	4.22E-01	1.28E-04	1.11E-03
ILMN_1769665	RAB5C	4.22E-01	9.25E-05	8.38E-04
ILMN_1767612	BBS2	4.22E-01	5.66E-06	7.79E-05
ILMN_1849013		4.22E-01	1.05E-02	4.85E-02
ILMN_1658289	WDR54	4.22E-01	2.06E-07	4.81E-06
ILMN_1719199	TULP3	4.22E-01	2.36E-07	5.39E-06
ILMN_2309848	FXYD5	4.22E-01	5.61E-04	3.99E-03
ILMN_1724181	IL15	4.21E-01	1.60E-07	3.89E-06
ILMN_1714393	RAB24	4.21E-01	5.00E-05	4.94E-04
ILMN_1765060	FBXO34	4.21E-01	3.88E-05	3.98E-04
ILMN_3238106	FAM161A	4.21E-01	1.38E-04	1.18E-03
ILMN_2131880	DPY30	4.21E-01	1.82E-04	1.51E-03
ILMN_2401873	DUSP10	4.21E-01	2.53E-03	1.45E-02
ILMN_1800512	HMOX1	4.21E-01	1.33E-03	8.32E-03
ILMN_2303955	FKBP1B	4.20E-01	8.05E-05	7.44E-04
ILMN_1769566	ATG3	4.20E-01	9.93E-06	1.25E-04
ILMN_1734184	P76	4.20E-01	1.33E-07	3.36E-06
ILMN_1750409	RAB9A	4.20E-01	6.95E-05	6.58E-04
ILMN_1727050	PPFIA1	4.20E-01	1.52E-06	2.53E-05
ILMN_1664216	NKIRAS1	4.20E-01	6.02E-08	1.73E-06
ILMN_3230215	TOX2	4.19E-01	6.43E-04	4.48E-03
ILMN_2211672	TSNAX	4.19E-01	8.60E-05	7.88E-04
ILMN_1807737	SFRS17A	4.19E-01	4.13E-07	8.53E-06
ILMN_3183517	LOC100130837	4.19E-01	4.67E-04	3.41E-03
ILMN_1672843	FBXO8	4.19E-01	3.10E-07	6.74E-06
ILMN_3245564	RICH2	4.19E-01	5.11E-07	1.02E-05
ILMN_1688160	WDR27	4.19E-01	3.58E-07	7.56E-06
ILMN_1814002	TEAD3	4.19E-01	2.47E-07	5.57E-06
ILMN_1683916	PEX13	4.19E-01	8.90E-05	8.12E-04
ILMN_1699226	UBR4	4.18E-01	3.80E-03	2.05E-02
ILMN_2096322	ADIPOR1	4.18E-01	2.38E-07	5.42E-06
ILMN_1757882	PPP1R16A	4.18E-01	6.13E-05	5.88E-04

ILMN_2216918	SHPK	4.18E-01	2.16E-03	1.27E-02
ILMN_1703471	ATF6	4.18E-01	9.23E-06	1.17E-04
ILMN_1725620	GGNBP2	4.18E-01	1.75E-10	1.86E-08
ILMN_1863484		4.17E-01	1.28E-06	2.18E-05
ILMN_1763147	NDUFB6	4.17E-01	1.84E-04	1.52E-03
ILMN_3236675	LOC100133823	4.17E-01	6.68E-05	6.36E-04
ILMN_1770044	CHRNA5	4.16E-01	5.49E-05	5.36E-04
ILMN_1656287	SPOCK2	4.16E-01	3.93E-03	2.11E-02
ILMN_1695893	NXPH4	4.16E-01	1.63E-05	1.91E-04
ILMN_2070896	BMPR2	4.16E-01	9.92E-07	1.78E-05
ILMN_1682818	TTLL3	4.16E-01	1.85E-03	1.10E-02
ILMN_2326713	CD151	4.16E-01	5.28E-07	1.05E-05
ILMN_1673509	RPL28	4.16E-01	8.88E-04	5.91E-03
ILMN_1789419	EXOC3	4.16E-01	3.15E-06	4.75E-05
ILMN_1789839	GTF3C1	4.15E-01	8.67E-06	1.12E-04
ILMN_1656186	SLC41A1	4.15E-01	9.35E-10	6.53E-08
ILMN_1683932	ZNF425	4.15E-01	1.26E-06	2.16E-05
ILMN_2339028	PKD1	4.15E-01	4.25E-04	3.14E-03
ILMN_2366391	PRDX1	4.15E-01	1.01E-06	1.80E-05
ILMN_1659523	USP39	4.14E-01	1.22E-09	8.00E-08
ILMN_1678652	C10orf61	4.14E-01	2.38E-05	2.63E-04
ILMN_1761812	DMD	4.14E-01	6.61E-03	3.28E-02
ILMN_1795876	GPS1	4.14E-01	5.26E-08	1.56E-06
ILMN_1714397	CRYL1	4.14E-01	3.36E-03	1.85E-02
ILMN_1690826	TNKS1BP1	4.13E-01	6.41E-06	8.64E-05
ILMN_1717099	DSCR3	4.13E-01	2.15E-09	1.24E-07
ILMN_1661342	SEPT6	4.13E-01	6.16E-07	1.19E-05
ILMN_1754757	SCNN1D	4.13E-01	8.17E-04	5.50E-03
ILMN_1661194	CLDN14	4.13E-01	2.69E-07	5.97E-06
ILMN_1760728	KIRREL2	4.13E-01	5.73E-08	1.66E-06
ILMN_2369286	NME7	4.13E-01	3.15E-03	1.75E-02
ILMN_2374633	ZWILCH	4.12E-01	2.35E-05	2.60E-04
ILMN_1678300	MGC40489	4.12E-01	1.54E-03	9.42E-03
ILMN_1737991	LOC650298	4.12E-01	8.79E-06	1.13E-04
ILMN_1753712	STX10	4.12E-01	5.21E-05	5.12E-04
ILMN_1770667	HECA	4.12E-01	7.30E-06	9.65E-05
ILMN_1709740	TXNDC16	4.12E-01	1.02E-07	2.71E-06
ILMN_2310589	DIABLO	4.12E-01	4.75E-08	1.44E-06
ILMN_2131336	TMEM194	4.12E-01	3.58E-07	7.56E-06
ILMN_1653263	GPRIN1	4.12E-01	1.13E-05	1.40E-04
ILMN_1772722	MRPS33	4.12E-01	9.70E-04	6.38E-03
ILMN_1754553	MED19	4.12E-01	4.41E-07	8.95E-06
ILMN_3188106	CYTH2	4.12E-01	1.01E-06	1.81E-05
ILMN_2041293	SQLE	4.12E-01	4.22E-06	6.09E-05
ILMN_1657624	NSFL1C	4.12E-01	8.66E-08	2.34E-06
ILMN_1715188	USP8	4.11E-01	5.19E-04	3.73E-03

ILMN_1711368	TRAPPC9	4.11E-01	5.26E-07	1.05E-05
ILMN_2401641	ALDH3A2	4.11E-01	2.53E-05	2.77E-04
ILMN_1686948	CASC4	4.11E-01	5.45E-08	1.60E-06
ILMN_1695991	COLQ	4.11E-01	2.13E-05	2.40E-04
ILMN_1813400	CBR4	4.11E-01	1.37E-05	1.64E-04
ILMN_2367458	UFD1L	4.11E-01	2.51E-08	8.57E-07
ILMN_1799589	NOXA1	4.11E-01	1.27E-08	5.01E-07
ILMN_3238006	LOC730323	4.11E-01	2.37E-06	3.70E-05
ILMN_1702389	ZC3H3	4.11E-01	5.16E-04	3.71E-03
ILMN_2414878	STXBP1	4.11E-01	1.46E-04	1.24E-03
ILMN_2366719	NSFL1C	4.10E-01	4.34E-07	8.84E-06
ILMN_1756862	APOL3	4.10E-01	2.88E-03	1.62E-02
ILMN_1874678		4.10E-01	1.77E-06	2.87E-05
ILMN_1810785	RNF11	4.10E-01	2.97E-09	1.58E-07
ILMN_3236344	CTGLF7	4.09E-01	1.46E-05	1.73E-04
ILMN_1672461	SPPL2B	4.09E-01	4.14E-05	4.21E-04
ILMN_3233871	ATXN1L	4.09E-01	3.36E-07	7.21E-06
ILMN_1701918	KLHDC9	4.09E-01	6.09E-06	8.29E-05
ILMN_1708805	NCOA3	4.09E-01	1.22E-04	1.06E-03
ILMN_1782543	EEF1D	4.08E-01	4.21E-07	8.64E-06
ILMN_1667213	DFFA	4.08E-01	5.32E-06	7.40E-05
ILMN_3266197	LOC100130171	4.07E-01	1.34E-03	8.38E-03
ILMN_1790985	dJ341D10.1	4.07E-01	2.63E-05	2.87E-04
ILMN_1788135	APITD1	4.07E-01	5.05E-08	1.51E-06
ILMN_2367753	ATP2B4	4.07E-01	7.88E-08	2.17E-06
ILMN_1659801	ATP6V1C1	4.07E-01	9.26E-07	1.68E-05
ILMN_1775111	SND1	4.07E-01	4.06E-07	8.41E-06
ILMN_1795388	INSM2	4.06E-01	4.99E-06	7.03E-05
ILMN_1773228	DLST	4.06E-01	1.26E-06	2.16E-05
ILMN_2136446	CTNNAL1	4.06E-01	6.90E-05	6.53E-04
ILMN_2408987	SMARCD3	4.06E-01	4.43E-07	8.99E-06
ILMN_3274045	LOC100130229	4.06E-01	1.03E-03	6.70E-03
ILMN_3228688	LOC730415	4.06E-01	7.07E-06	9.39E-05
ILMN_1659753	LAMP2	4.06E-01	1.61E-06	2.65E-05
ILMN_1657683	C1orf198	4.06E-01	1.68E-06	2.76E-05
ILMN_1696701	LOC344595	4.06E-01	2.98E-05	3.19E-04
ILMN_2048793	CIAO1	4.06E-01	8.05E-06	1.05E-04
ILMN_3239895	LOC100134053	4.06E-01	2.45E-04	1.95E-03
ILMN_1797298	ARMC7	4.05E-01	3.69E-06	5.45E-05
ILMN_2320513	APBB3	4.05E-01	5.59E-07	1.10E-05
ILMN_1741455	USP30	4.05E-01	1.19E-06	2.07E-05
ILMN_1785113	MUT	4.05E-01	9.47E-03	4.44E-02
ILMN_1734830	MTHFR	4.05E-01	5.35E-06	7.43E-05
ILMN_1679401	TRPM4	4.05E-01	4.80E-07	9.66E-06
ILMN_1704079	RBM38	4.05E-01	8.73E-05	7.98E-04
ILMN_1707156	LRRFIP2	4.05E-01	1.87E-04	1.54E-03

ILMN_1815012	EXOC7	4.05E-01	1.38E-07	3.45E-06
ILMN_1655126	PI4KAP2	4.05E-01	5.20E-06	7.26E-05
ILMN_3229424	LOC730101	4.05E-01	5.69E-08	1.65E-06
ILMN_1764186	LOC146517	4.05E-01	3.66E-07	7.72E-06
ILMN_1786718	NDUFV1	4.05E-01	8.82E-04	5.88E-03
ILMN_2153916	HSPA2	4.04E-01	2.83E-05	3.06E-04
ILMN_1756572	COQ2	4.04E-01	1.17E-07	3.02E-06
ILMN_1760617	IFT80	4.04E-01	1.33E-08	5.21E-07
ILMN_1784269	AASDH	4.04E-01	6.28E-08	1.79E-06
ILMN_1656822	DNM2	4.03E-01	4.25E-06	6.12E-05
ILMN_1781680	DAP3	4.03E-01	1.80E-05	2.07E-04
ILMN_2381476	SPG3A	4.03E-01	6.85E-06	9.13E-05
ILMN_2368575	GIYD1	4.03E-01	3.37E-08	1.09E-06
ILMN_2405400	GRK4	4.03E-01	5.32E-07	1.05E-05
ILMN_3287244	LOC728138	4.03E-01	8.85E-06	1.13E-04
ILMN_3250389	LOC440895	4.03E-01	1.24E-07	3.17E-06
ILMN_1706376	OSBP	4.03E-01	1.77E-07	4.24E-06
ILMN_1801383	SMG1	4.03E-01	2.73E-07	6.04E-06
ILMN_1773780	FAM173A	4.02E-01	1.10E-06	1.93E-05
ILMN_2359211	AP2A1	4.02E-01	1.42E-04	1.21E-03
ILMN_1703314	KLHL36	4.02E-01	2.59E-04	2.05E-03
ILMN_1700384	KIAA1522	4.02E-01	4.08E-06	5.93E-05
ILMN_2410986	STAT3	4.02E-01	3.57E-06	5.30E-05
ILMN_1653205	C8orf59	4.02E-01	6.43E-04	4.49E-03
ILMN_1717180	MTMR6	4.01E-01	8.29E-05	7.62E-04
ILMN_1655469	TSPAN3	4.01E-01	1.66E-04	1.39E-03
ILMN_2380938	SYT7	4.01E-01	7.33E-04	5.01E-03
ILMN_1757134	MPV17L2	4.01E-01	1.65E-04	1.38E-03
ILMN_1690329	FAM118B	4.01E-01	2.03E-06	3.22E-05
ILMN_1754538	C10orf58	4.01E-01	5.68E-03	2.89E-02
ILMN_2064655	CXorf40A	4.00E-01	3.40E-05	3.57E-04
ILMN_1708743	NT5DC2	4.00E-01	1.58E-03	9.62E-03
ILMN_1692834	C1orf26	4.00E-01	4.70E-07	9.48E-06
ILMN_1759252	ADD1	4.00E-01	1.31E-04	1.13E-03
ILMN_1727738	RAB33B	4.00E-01	1.28E-03	8.05E-03
ILMN_1785618	SMTN	4.00E-01	2.37E-07	5.40E-06
ILMN_1675501	SENP5	4.00E-01	5.30E-06	7.37E-05
ILMN_1807277	IFI30	3.99E-01	5.06E-04	3.65E-03
ILMN_2345739	CAPRIN2	3.99E-01	5.49E-05	5.35E-04
ILMN_1695509	PTPN12	3.99E-01	2.00E-04	1.63E-03
ILMN_1667417	RAB23	3.99E-01	2.63E-09	1.45E-07
ILMN_1685397	ITGA3	3.99E-01	4.08E-07	8.44E-06
ILMN_1768077	C10orf61	3.99E-01	5.75E-06	7.90E-05
ILMN_2343278	PPAP2A	3.99E-01	1.06E-08	4.32E-07
ILMN_1802380	RERE	3.98E-01	4.83E-04	3.51E-03
ILMN_1723418	CEL	3.98E-01	2.20E-06	3.46E-05

ILMN_1726466	HDHD3	3.98E-01	1.94E-06	3.10E-05
ILMN_1753515	SRR	3.98E-01	3.48E-06	5.17E-05
ILMN_1760246	BSN	3.98E-01	8.15E-08	2.23E-06
ILMN_1723211	L2HGDH	3.98E-01	3.79E-06	5.58E-05
ILMN_2325008	DHX40	3.98E-01	1.61E-06	2.65E-05
ILMN_1802162	RFESD	3.97E-01	4.80E-05	4.77E-04
ILMN_2061446	AADACL1	3.97E-01	4.28E-06	6.17E-05
ILMN_1771689	EXD2	3.97E-01	1.45E-03	8.94E-03
ILMN_1686748	TMEM9	3.97E-01	2.37E-09	1.33E-07
ILMN_1719232	DGCR14	3.97E-01	1.24E-05	1.51E-04
ILMN_1702396	PACSIN2	3.97E-01	3.61E-04	2.73E-03
ILMN_2373755	IFT88	3.96E-01	7.26E-06	9.60E-05
ILMN_1741755	TRIM29	3.96E-01	7.89E-07	1.46E-05
ILMN_1790807	XPC	3.96E-01	9.48E-06	1.20E-04
ILMN_3237584	LOC100133489	3.96E-01	7.69E-07	1.43E-05
ILMN_2160727	CYTH3	3.96E-01	4.02E-05	4.10E-04
ILMN_1703379	VPS41	3.96E-01	1.09E-03	7.03E-03
ILMN_1733615	MTF2	3.96E-01	4.52E-06	6.48E-05
ILMN_1673721	EXO1	3.96E-01	7.14E-03	3.50E-02
ILMN_1697629	PLA2G4B	3.96E-01	4.16E-05	4.22E-04
ILMN_2298365	PPP2R2B	3.95E-01	8.36E-06	1.08E-04
ILMN_1686135	CCDC45	3.95E-01	3.28E-05	3.46E-04
ILMN_2381697	P4HA2	3.95E-01	1.92E-04	1.57E-03
ILMN_1747251	LTB4R	3.95E-01	1.21E-03	7.66E-03
ILMN_2044226	PPP3CA	3.95E-01	7.52E-04	5.12E-03
ILMN_1742427	POLR1D	3.95E-01	1.38E-07	3.46E-06
ILMN_2120072	FLJ13305	3.95E-01	1.22E-05	1.49E-04
ILMN_2389273	FXR1	3.95E-01	2.22E-07	5.13E-06
ILMN_1666385	CALM3	3.95E-01	1.69E-06	2.77E-05
ILMN_2125869	ACTA1	3.94E-01	1.03E-03	6.68E-03
ILMN_1806415	TTLL1	3.94E-01	5.03E-04	3.63E-03
ILMN_1676946	AP3M2	3.94E-01	7.62E-08	2.10E-06
ILMN_3237396	AAGAB	3.94E-01	1.51E-06	2.51E-05
ILMN_1747775	STX2	3.93E-01	2.14E-03	1.25E-02
ILMN_1777745	FAM133B	3.93E-01	3.29E-08	1.07E-06
ILMN_1662174	ORMDL3	3.93E-01	4.48E-06	6.43E-05
ILMN_1753467	SAMD4B	3.93E-01	3.06E-06	4.63E-05
ILMN_1713006	CDC34	3.93E-01	1.85E-03	1.10E-02
ILMN_1651799	SLC38A2	3.93E-01	1.30E-07	3.29E-06
ILMN_1750636	RPS26L	3.93E-01	1.77E-03	1.06E-02
ILMN_3265365	CEP78	3.93E-01	2.05E-05	2.32E-04
ILMN_1655720	CNNM4	3.92E-01	8.52E-07	1.56E-05
ILMN_2177090	LOC200030	3.92E-01	4.48E-04	3.29E-03
ILMN_1673798	PPOX	3.92E-01	2.22E-03	1.30E-02
ILMN_2385647	ALAS1	3.92E-01	1.38E-06	2.33E-05
ILMN_1795911	TTC37	3.92E-01	2.69E-06	4.12E-05

ILMN_2149766	APPBP2	3.92E-01	6.07E-07	1.17E-05
ILMN_1765076	APPL2	3.92E-01	2.79E-05	3.02E-04
ILMN_1804415	SMAGP	3.92E-01	3.31E-08	1.07E-06
ILMN_1728071	KRAS	3.92E-01	4.06E-07	8.41E-06
ILMN_1656136	STAU1	3.92E-01	1.23E-03	7.79E-03
ILMN_1778144	FLJ20489	3.91E-01	8.39E-04	5.63E-03
ILMN_1716480	ACD	3.91E-01	4.03E-07	8.34E-06
ILMN_2404407	ABAT	3.91E-01	6.50E-03	3.23E-02
ILMN_1754421	NDUFAF1	3.91E-01	1.66E-04	1.39E-03
ILMN_1699570	TPD52L2	3.91E-01	1.97E-06	3.14E-05
ILMN_3245103	RNU11	3.91E-01	1.29E-03	8.09E-03
ILMN_2098743	THEM2	3.91E-01	8.23E-08	2.24E-06
ILMN_1739821	EIF2S1	3.90E-01	1.68E-05	1.96E-04
ILMN_1757072	LOC642489	3.90E-01	1.18E-03	7.53E-03
ILMN_1662426	AP2S1	3.90E-01	4.00E-06	5.83E-05
ILMN_2343047	ABCB9	3.90E-01	2.33E-07	5.32E-06
ILMN_2145997	SP4	3.89E-01	7.30E-06	9.65E-05
ILMN_1789733	CLIP3	3.89E-01	1.47E-05	1.74E-04
ILMN_1722532	JMJD1A	3.89E-01	1.32E-05	1.59E-04
ILMN_1738075	CMIP	3.89E-01	1.66E-05	1.94E-04
ILMN_1709227	CCDC84	3.89E-01	1.30E-05	1.57E-04
ILMN_1685289	C16orf58	3.89E-01	1.28E-03	8.05E-03
ILMN_3254492	LOC100129650	3.89E-01	2.06E-03	1.21E-02
ILMN_2180239	DOPEY2	3.88E-01	3.04E-04	2.36E-03
ILMN_1752592	HLA-DRB4	3.88E-01	5.70E-03	2.89E-02
ILMN_1805535	VRK3	3.88E-01	2.08E-06	3.29E-05
ILMN_1780659	NUDT6	3.88E-01	8.14E-06	1.06E-04
ILMN_2339835	PTGS1	3.88E-01	1.09E-07	2.84E-06
ILMN_1731349	HOXA13	3.88E-01	2.98E-03	1.67E-02
ILMN_1803988	MCL1	3.87E-01	1.81E-03	1.08E-02
ILMN_3239060	KRBA1	3.87E-01	4.00E-07	8.30E-06
ILMN_2065773	SCG5	3.87E-01	3.16E-03	1.75E-02
ILMN_1654571	FCHO1	3.87E-01	1.65E-05	1.93E-04
ILMN_1735979	BCKDHA	3.87E-01	8.21E-07	1.51E-05
ILMN_1780842	RANBP6	3.87E-01	2.75E-07	6.06E-06
ILMN_1801226	DOCK6	3.87E-01	3.79E-05	3.91E-04
ILMN_1808405	HLA-DQA1	3.86E-01	4.68E-05	4.67E-04
ILMN_1730670	FSTL3	3.86E-01	1.73E-03	1.04E-02
ILMN_2162799	AHR	3.86E-01	3.37E-03	1.85E-02
ILMN_1796245	DNASE2	3.86E-01	6.36E-03	3.17E-02
ILMN_1669015	XPNPEP1	3.86E-01	6.44E-04	4.49E-03
ILMN_1680434	MAPK8IP1	3.86E-01	1.89E-04	1.56E-03
ILMN_1766499	HSPA2	3.86E-01	7.51E-06	9.88E-05
ILMN_1688318	MGC72104	3.85E-01	6.41E-04	4.47E-03
ILMN_2044572	TBC1D20	3.85E-01	1.16E-05	1.43E-04
ILMN_1654653	KLC1	3.85E-01	4.57E-06	6.53E-05

ILMN_1674399	ZNF143	3.85E-01	2.73E-04	2.14E-03
ILMN_2330994	NKTR	3.85E-01	5.04E-06	7.08E-05
ILMN_2409395	CCNC	3.84E-01	5.56E-06	7.68E-05
ILMN_1739683	LRRC6	3.84E-01	5.43E-03	2.78E-02
ILMN_1725963	LOC647719	3.84E-01	5.50E-03	2.81E-02
ILMN_2046730	S100A10	3.84E-01	2.91E-04	2.27E-03
ILMN_1793118	TAX1BP1	3.84E-01	4.39E-07	8.93E-06
ILMN_2225746	C17orf59	3.84E-01	1.37E-07	3.44E-06
ILMN_1758825	ABLIM2	3.84E-01	6.27E-03	3.13E-02
ILMN_2265995	NBPF3	3.84E-01	9.48E-05	8.57E-04
ILMN_2381197	RNF19A	3.83E-01	5.69E-05	5.53E-04
ILMN_2380418	BICD2	3.83E-01	1.32E-05	1.59E-04
ILMN_1685534	PILRB	3.83E-01	2.75E-05	2.98E-04
ILMN_1771179	CYB561	3.83E-01	6.02E-08	1.73E-06
ILMN_1765021	TOP3B	3.83E-01	1.13E-04	9.92E-04
ILMN_1786039	RNF34	3.83E-01	1.94E-03	1.15E-02
ILMN_1814917	TLE2	3.83E-01	7.23E-06	9.58E-05
ILMN_2232177	ACTN1	3.82E-01	1.62E-08	6.08E-07
ILMN_1699545	PCSK7	3.82E-01	6.35E-05	6.07E-04
ILMN_2142117	LYPLAL1	3.82E-01	1.56E-05	1.83E-04
ILMN_1669070	MIPEP	3.82E-01	4.46E-06	6.40E-05
ILMN_1704091	DGAT1	3.81E-01	2.05E-04	1.67E-03
ILMN_1699206	FHDC1	3.81E-01	5.21E-07	1.04E-05
ILMN_1752741	TRIM23	3.81E-01	6.15E-06	8.36E-05
ILMN_1731194	STRAP	3.81E-01	7.50E-07	1.40E-05
ILMN_1796712	S100A10	3.81E-01	1.19E-03	7.58E-03
ILMN_2150352	CBWD5	3.80E-01	4.68E-05	4.67E-04
ILMN_2095840	MYST3	3.80E-01	7.75E-05	7.21E-04
ILMN_1792748	CPS1	3.80E-01	7.52E-04	5.12E-03
ILMN_3226181	NUDT7	3.80E-01	8.32E-03	3.98E-02
ILMN_1768062	ADK	3.80E-01	1.92E-05	2.19E-04
ILMN_2405521	MTHFD2	3.80E-01	5.69E-05	5.53E-04
ILMN_1690170	CRABP2	3.80E-01	3.66E-03	1.98E-02
ILMN_1674560	GBA2	3.80E-01	2.79E-06	4.25E-05
ILMN_1760982	ZNF187	3.79E-01	2.42E-06	3.76E-05
ILMN_1738773	HRC	3.79E-01	3.84E-03	2.07E-02
ILMN_1721741	ATPBD1B	3.79E-01	1.08E-03	6.99E-03
ILMN_1665290	LOC643995	3.79E-01	1.29E-05	1.56E-04
ILMN_1679771	LOC388969	3.79E-01	2.18E-05	2.45E-04
ILMN_1797531	PRKAG2	3.79E-01	1.13E-06	1.97E-05
ILMN_1676955	TYK2	3.79E-01	3.54E-05	3.68E-04
ILMN_1731783	ATP1A1	3.79E-01	3.70E-03	2.01E-02
ILMN_2388155	CASP3	3.78E-01	8.10E-06	1.06E-04
ILMN_2223350	C13orf1	3.78E-01	2.53E-04	2.01E-03
ILMN_1702763	ZMYM1	3.78E-01	2.23E-06	3.50E-05
ILMN_1724230	LOC642236	3.78E-01	1.81E-05	2.08E-04

ILMN_1799856	NOMO2	3.77E-01	8.42E-07	1.54E-05
ILMN_1653203	EFEMP2	3.77E-01	1.64E-05	1.92E-04
ILMN_2062620	NMT2	3.77E-01	5.96E-04	4.21E-03
ILMN_2336609	SYTL2	3.77E-01	2.46E-03	1.41E-02
ILMN_1694404	RASA4	3.77E-01	7.82E-05	7.26E-04
ILMN_1704286	FXYD5	3.77E-01	4.40E-06	6.33E-05
ILMN_1703246	SBF1	3.76E-01	1.94E-08	7.05E-07
ILMN_1762436	UBB	3.76E-01	8.05E-05	7.44E-04
ILMN_3293730	LOC100133169	3.76E-01	3.63E-05	3.76E-04
ILMN_3258795	FAM13B	3.76E-01	5.31E-05	5.20E-04
ILMN_1775703	TRAPPC6A	3.76E-01	5.52E-05	5.39E-04
ILMN_1794063	ANKRD27	3.76E-01	1.06E-04	9.40E-04
ILMN_1775034	LOC649987	3.76E-01	1.88E-05	2.16E-04
ILMN_1653429	SLC35A3	3.76E-01	1.21E-06	2.09E-05
ILMN_2067852	SLC30A1	3.75E-01	1.27E-04	1.10E-03
ILMN_1665559	CDK2	3.75E-01	1.17E-08	4.71E-07
ILMN_1802646	EPHB6	3.75E-01	2.42E-03	1.40E-02
ILMN_2307978	FAM108A3	3.75E-01	1.44E-05	1.70E-04
ILMN_2272074	TROVE2	3.75E-01	8.42E-05	7.73E-04
ILMN_1775566	ATP1A1	3.75E-01	4.08E-04	3.04E-03
ILMN_1796968	INTS5	3.75E-01	4.97E-06	7.00E-05
ILMN_1852793		3.75E-01	1.39E-07	3.48E-06
ILMN_2399036	SEPN1	3.75E-01	2.43E-05	2.67E-04
ILMN_1665737	UFD1L	3.75E-01	1.42E-06	2.39E-05
ILMN_3246634	LOC100134108	3.74E-01	7.43E-05	6.95E-04
ILMN_1709817	SLC35A5	3.74E-01	1.13E-04	9.98E-04
ILMN_1703006	MTCH1	3.74E-01	1.47E-05	1.74E-04
ILMN_1674297	HCFC2	3.74E-01	1.84E-04	1.52E-03
ILMN_1664921	PPP6C	3.74E-01	1.66E-04	1.39E-03
ILMN_2329958	ABI1	3.74E-01	4.02E-03	2.15E-02
ILMN_1725183	TBCE	3.73E-01	3.85E-07	8.04E-06
ILMN_1651684	KIAA0586	3.73E-01	4.12E-06	5.98E-05
ILMN_3272378	EZR	3.73E-01	9.78E-04	6.42E-03
ILMN_2373377	RTN2	3.73E-01	8.78E-03	4.17E-02
ILMN_3236130	LOC100132547	3.73E-01	6.71E-03	3.32E-02
ILMN_1688702	PJA2	3.73E-01	1.35E-05	1.62E-04
ILMN_1720965	TULP4	3.73E-01	3.94E-08	1.24E-06
ILMN_1796912	ARHGEF7	3.73E-01	3.67E-03	1.99E-02
ILMN_3301042	LQK1	3.72E-01	4.05E-06	5.90E-05
ILMN_1763694	RSPRY1	3.72E-01	4.25E-04	3.14E-03
ILMN_1787657	CLDN12	3.71E-01	2.02E-03	1.19E-02
ILMN_1655206	ZBTB34	3.71E-01	2.66E-07	5.91E-06
ILMN_2056032	CD99	3.71E-01	2.15E-06	3.38E-05
ILMN_1802905	PIAS4	3.71E-01	3.49E-07	7.41E-06
ILMN_2140389	TMEM185A	3.71E-01	1.59E-08	6.01E-07
ILMN_1681898	LOC647784	3.71E-01	1.82E-06	2.94E-05

ILMN_2232166	CCDC90B	3.71E-01	2.11E-04	1.71E-03
ILMN_1677697	LOC441377	3.70E-01	9.25E-03	4.36E-02
ILMN_1754529	SPG7	3.70E-01	3.70E-05	3.82E-04
ILMN_1696027	LOC642333	3.70E-01	6.76E-04	4.68E-03
ILMN_1656386	SEC24D	3.70E-01	1.02E-04	9.15E-04
ILMN_2286800	DNM1L	3.70E-01	4.71E-05	4.69E-04
ILMN_1667453	HINFP	3.70E-01	6.88E-08	1.92E-06
ILMN_1803483	KIAA2013	3.70E-01	4.70E-07	9.48E-06
ILMN_1652237	CBR3	3.69E-01	8.05E-03	3.88E-02
ILMN_1653367	TAF12	3.69E-01	3.51E-05	3.66E-04
ILMN_1736888	SAR1B	3.69E-01	7.35E-04	5.03E-03
ILMN_1727073	MEA1	3.69E-01	4.30E-09	2.14E-07
ILMN_2389114	FIGNL1	3.69E-01	7.06E-04	4.86E-03
ILMN_3301052	LOC728791	3.69E-01	2.68E-04	2.11E-03
ILMN_1767816	APH1B	3.69E-01	6.09E-03	3.06E-02
ILMN_1809208	KIAA1543	3.69E-01	1.95E-05	2.22E-04
ILMN_2202948	BUB1	3.69E-01	1.31E-03	8.19E-03
ILMN_1800447	PHKB	3.69E-01	8.94E-03	4.23E-02
ILMN_1704621	WDR44	3.68E-01	3.98E-04	2.97E-03
ILMN_1687501	MOXD1	3.68E-01	5.64E-04	4.01E-03
ILMN_1751338	NUP133	3.68E-01	2.61E-05	2.85E-04
ILMN_1665696	EFNA4	3.68E-01	8.85E-06	1.13E-04
ILMN_2096747	SNORA33	3.67E-01	1.07E-04	9.48E-04
ILMN_1732514	GALK2	3.67E-01	6.84E-06	9.13E-05
ILMN_2300186	DYNLL1	3.67E-01	4.83E-03	2.51E-02
ILMN_1701855	PPP1CC	3.67E-01	1.65E-07	3.99E-06
ILMN_2399140	RAB5C	3.67E-01	2.49E-04	1.98E-03
ILMN_1653412	RAXL1	3.67E-01	1.33E-05	1.60E-04
ILMN_2405470	KLHDC9	3.67E-01	4.92E-08	1.48E-06
ILMN_1808115	ATP7A	3.66E-01	3.35E-04	2.56E-03
ILMN_1689294	LOC85390	3.66E-01	2.84E-06	4.32E-05
ILMN_1810267	DNHD1	3.66E-01	1.25E-03	7.88E-03
ILMN_1737462	OXR1	3.66E-01	1.98E-07	4.65E-06
ILMN_1774974	CLUAP1	3.66E-01	8.77E-09	3.72E-07
ILMN_3244319	CCDC125	3.65E-01	1.73E-04	1.44E-03
ILMN_2311518	TROVE2	3.65E-01	1.24E-05	1.51E-04
ILMN_2069945	SNRNP27	3.65E-01	1.19E-05	1.46E-04
ILMN_2367233	ZNF654	3.65E-01	4.22E-04	3.12E-03
ILMN_3240594	RNU4ATAC	3.65E-01	2.66E-04	2.10E-03
ILMN_1659255	RP2	3.65E-01	1.90E-05	2.18E-04
ILMN_1788387	UGCGL2	3.65E-01	3.25E-06	4.88E-05
ILMN_2063584	CLIC4	3.64E-01	3.97E-04	2.97E-03
ILMN_1798081	PTPRF	3.64E-01	2.04E-06	3.24E-05
ILMN_2127328	PURA	3.64E-01	1.36E-04	1.17E-03
ILMN_1654032	ZER1	3.64E-01	1.10E-05	1.36E-04
ILMN_1661833	ANKRD12	3.64E-01	7.51E-06	9.88E-05

ILMN_1672571	TFIP11	3.64E-01	8.74E-07	1.59E-05
ILMN_3236239	LOC100129550	3.63E-01	1.32E-08	5.18E-07
ILMN_1697906	WBP4	3.63E-01	2.14E-05	2.41E-04
ILMN_2325394	MSH5	3.63E-01	9.13E-04	6.05E-03
ILMN_1728349	TMEM63B	3.63E-01	8.15E-08	2.23E-06
ILMN_1745573	TTC13	3.62E-01	2.06E-04	1.68E-03
ILMN_2320336	CLK3	3.62E-01	5.44E-06	7.54E-05
ILMN_2348367	FGFRL1	3.62E-01	1.57E-05	1.85E-04
ILMN_1795428	WDR59	3.62E-01	1.73E-06	2.82E-05
ILMN_1711462	MNS1	3.61E-01	9.57E-05	8.63E-04
ILMN_3309453	RNU4-1	3.61E-01	5.24E-03	2.70E-02
ILMN_2388484	MAP2	3.61E-01	1.05E-03	6.83E-03
ILMN_3251567	FBXW2	3.61E-01	1.85E-05	2.13E-04
ILMN_2352580	MBD1	3.61E-01	8.88E-07	1.62E-05
ILMN_2345319	PREPL	3.61E-01	1.82E-05	2.10E-04
ILMN_1651819	GALNT11	3.61E-01	1.52E-06	2.53E-05
ILMN_1696046	SIVA	3.61E-01	9.09E-05	8.26E-04
ILMN_1784946	ORC3L	3.61E-01	4.66E-05	4.65E-04
ILMN_2169152	SRGN	3.60E-01	1.01E-03	6.59E-03
ILMN_1669691	LOC650433	3.60E-01	1.02E-03	6.66E-03
ILMN_1702526	C17orf48	3.59E-01	2.51E-05	2.75E-04
ILMN_1665243	FKBP14	3.59E-01	1.18E-05	1.45E-04
ILMN_1783226	SSR2	3.59E-01	6.98E-05	6.60E-04
ILMN_1681641	DLEU1	3.59E-01	8.99E-03	4.25E-02
ILMN_3247587	SLC48A1	3.59E-01	3.72E-07	7.82E-06
ILMN_1769290	TTC39B	3.59E-01	1.50E-04	1.28E-03
ILMN_1799208	CSGALNACT2	3.59E-01	1.01E-07	2.68E-06
ILMN_1773741	GOLGA5	3.59E-01	7.46E-06	9.83E-05
ILMN_1718831	TMEM57	3.59E-01	8.17E-04	5.50E-03
ILMN_1697567	TPM3	3.58E-01	1.17E-03	7.47E-03
ILMN_2413650	STIL	3.58E-01	3.93E-04	2.94E-03
ILMN_1807031	C14orf28	3.58E-01	1.81E-06	2.93E-05
ILMN_2260991	TSPO	3.58E-01	2.09E-03	1.23E-02
ILMN_1785158	HERPUD2	3.58E-01	1.39E-07	3.47E-06
ILMN_1690064	RUNDC2C	3.58E-01	4.92E-04	3.56E-03
ILMN_1798006	ANKRD35	3.58E-01	2.31E-03	1.34E-02
ILMN_2358382	ZFYVE1	3.58E-01	3.25E-07	7.01E-06
ILMN_1814650	TRAPPC4	3.58E-01	1.41E-05	1.68E-04
ILMN_1655961	C7orf54	3.57E-01	4.31E-03	2.28E-02
ILMN_2186482	TMED7	3.57E-01	8.19E-03	3.93E-02
ILMN_3226769	LOC730074	3.57E-01	2.84E-03	1.60E-02
ILMN_1774828	VEZT	3.57E-01	1.55E-03	9.46E-03
ILMN_1744912	CTTN	3.57E-01	6.47E-04	4.51E-03
ILMN_1657509	TSEN54	3.57E-01	2.69E-03	1.53E-02
ILMN_1724609	SLC2A8	3.57E-01	8.20E-06	1.07E-04
ILMN_1698072	PITRM1	3.57E-01	4.72E-06	6.69E-05

ILMN_2332990	DIABLO	3.57E-01	4.68E-06	6.65E-05
ILMN_3222974	PRKCB	3.57E-01	6.30E-03	3.15E-02
ILMN_1659240	MTMR14	3.57E-01	2.19E-08	7.73E-07
ILMN_1680591	RTKN	3.57E-01	3.87E-03	2.08E-02
ILMN_1688621	C9orf80	3.57E-01	1.40E-04	1.20E-03
ILMN_1684634	LOC647135	3.57E-01	1.56E-06	2.59E-05
ILMN_3225358	LOC729495	3.56E-01	2.39E-06	3.72E-05
ILMN_2181968	CBL	3.56E-01	1.69E-08	6.31E-07
ILMN_2057566	PGP	3.56E-01	8.19E-05	7.54E-04
ILMN_1769694	ACCN2	3.56E-01	7.69E-04	5.22E-03
ILMN_1682935	LYPLAL1	3.56E-01	1.99E-05	2.26E-04
ILMN_3291511	LOC100131971	3.56E-01	3.11E-03	1.73E-02
ILMN_2062381	LCOR	3.55E-01	1.19E-03	7.56E-03
ILMN_3253304	BRI3P1	3.55E-01	3.01E-05	3.22E-04
ILMN_1688322	ADIPOR1	3.55E-01	2.08E-05	2.35E-04
ILMN_1735151	EIF5A2	3.54E-01	3.12E-05	3.32E-04
ILMN_1725079	TSPAN31	3.54E-01	1.47E-03	9.07E-03
ILMN_1803984	MAK	3.54E-01	4.25E-04	3.14E-03
ILMN_1782939	ALB	3.54E-01	1.62E-03	9.86E-03
ILMN_1660356	PPP4R4	3.54E-01	9.24E-03	4.35E-02
ILMN_1715392	PRPF3	3.53E-01	1.99E-04	1.63E-03
ILMN_1662166	PTK7	3.53E-01	5.20E-03	2.68E-02
ILMN_1677607	SC5DL	3.53E-01	7.64E-05	7.12E-04
ILMN_1766309	ANKRD54	3.52E-01	6.52E-04	4.54E-03
ILMN_1768097	RPGR	3.52E-01	6.93E-07	1.31E-05
ILMN_1807491	LAIR2	3.52E-01	9.02E-05	8.21E-04
ILMN_2363426	MAX	3.52E-01	4.63E-07	9.36E-06
ILMN_2151441	FAM103A1	3.52E-01	9.91E-03	4.61E-02
ILMN_1666615	PREPL	3.52E-01	4.16E-04	3.09E-03
ILMN_3241164	KDSR	3.52E-01	6.52E-06	8.77E-05
ILMN_1781983	AP1B1	3.52E-01	2.76E-04	2.17E-03
ILMN_1801476	CDS1	3.52E-01	5.74E-04	4.07E-03
ILMN_1667429	SLC25A20	3.52E-01	2.47E-07	5.58E-06
ILMN_1672547	MYO9B	3.52E-01	1.02E-05	1.27E-04
ILMN_1811972	MYCBP2	3.52E-01	1.81E-05	2.08E-04
ILMN_1713450	MYL6B	3.52E-01	1.05E-04	9.34E-04
ILMN_1737981	FSTL5	3.52E-01	4.03E-03	2.16E-02
ILMN_1715994	HGS	3.51E-01	2.19E-03	1.28E-02
ILMN_1808837	LOC644029	3.51E-01	9.64E-06	1.22E-04
ILMN_1720440	HELQ	3.51E-01	4.27E-07	8.73E-06
ILMN_2337941	COPS8	3.51E-01	2.21E-03	1.29E-02
ILMN_1692956	LOC643949	3.51E-01	1.82E-03	1.09E-02
ILMN_1729533	APOA1BP	3.51E-01	4.98E-06	7.01E-05
ILMN_1715555	DBP	3.51E-01	9.15E-03	4.32E-02
ILMN_1714759	CNIH4	3.51E-01	4.71E-05	4.69E-04
ILMN_1704793	MYPOP	3.50E-01	2.81E-05	3.04E-04

ILMN_1652313	RNF215	3.50E-01	9.75E-06	1.23E-04
ILMN_1654013	C17orf28	3.50E-01	4.01E-03	2.15E-02
ILMN_1673111	TSEN34	3.50E-01	1.62E-03	9.86E-03
ILMN_2243308	ACVR1B	3.50E-01	8.32E-06	1.08E-04
ILMN_1765858	CAB39	3.50E-01	6.43E-07	1.23E-05
ILMN_2405756	VAMP1	3.50E-01	5.30E-05	5.19E-04
ILMN_1783846	RAPH1	3.49E-01	1.28E-04	1.11E-03
ILMN_1799860	PIGM	3.49E-01	6.06E-03	3.05E-02
ILMN_2073184	S1PR5	3.49E-01	7.06E-05	6.66E-04
ILMN_1670093	HIST2H2BF	3.49E-01	2.12E-03	1.24E-02
ILMN_2175447	ZNF767	3.49E-01	5.45E-07	1.08E-05
ILMN_1757262	ZBTB5	3.49E-01	1.39E-06	2.33E-05
ILMN_1869913		3.49E-01	1.59E-04	1.33E-03
ILMN_2049766	NFE2L3	3.48E-01	2.27E-05	2.53E-04
ILMN_1721563	TMEM127	3.48E-01	7.34E-05	6.88E-04
ILMN_1792837	CIAO1	3.48E-01	5.03E-06	7.07E-05
ILMN_3289171	LOC100131572	3.48E-01	1.97E-03	1.17E-02
ILMN_1654319	HAPLN3	3.48E-01	5.69E-03	2.89E-02
ILMN_1655864	LOC653853	3.48E-01	2.00E-03	1.18E-02
ILMN_1735038	MARCH3	3.48E-01	2.37E-04	1.90E-03
ILMN_1779547	HPSE	3.48E-01	6.81E-04	4.71E-03
ILMN_2191568	TUSC4	3.48E-01	2.11E-05	2.37E-04
ILMN_2333367	FKBP1A	3.48E-01	8.97E-05	8.17E-04
ILMN_1795991	C22orf28	3.47E-01	3.97E-04	2.96E-03
ILMN_2173524	FOXD4	3.47E-01	2.11E-05	2.37E-04
ILMN_1652806	ATP5J	3.47E-01	3.70E-03	2.00E-02
ILMN_1688642	LAMC3	3.47E-01	1.66E-03	1.01E-02
ILMN_1843932		3.47E-01	1.26E-06	2.16E-05
ILMN_1730630	CXorf56	3.47E-01	4.11E-05	4.18E-04
ILMN_1724293	KDELR2	3.47E-01	1.94E-08	7.07E-07
ILMN_1794399	SNRK	3.46E-01	5.50E-03	2.81E-02
ILMN_1676288	ACBD4	3.46E-01	1.42E-04	1.21E-03
ILMN_3201643	LOC100133019	3.46E-01	1.00E-05	1.26E-04
ILMN_1789095	BMPR2	3.46E-01	5.36E-04	3.84E-03
ILMN_3237986	MMGT1	3.46E-01	1.14E-06	2.00E-05
ILMN_1883997		3.46E-01	1.28E-08	5.06E-07
ILMN_1682957	PACSIN3	3.46E-01	1.12E-03	7.20E-03
ILMN_2311989	CUTA	3.46E-01	8.06E-05	7.46E-04
ILMN_1674316	LOC727751	3.46E-01	8.07E-08	2.21E-06
ILMN_2157544	GBF1	3.46E-01	3.23E-07	6.98E-06
ILMN_2361862	VLDLR	3.45E-01	1.28E-03	8.05E-03
ILMN_1901419		3.45E-01	1.05E-04	9.35E-04
ILMN_1708907	MEIG1	3.45E-01	4.22E-05	4.27E-04
ILMN_2191428	UBB	3.45E-01	1.76E-05	2.03E-04
ILMN_3239621	SNRNP27	3.45E-01	1.93E-06	3.09E-05
ILMN_1711383	STK4	3.45E-01	1.52E-06	2.53E-05

ILMN_1740842	SALL2	3.45E-01	1.20E-06	2.08E-05
ILMN_2360710	TPM1	3.45E-01	1.61E-06	2.65E-05
ILMN_2119224	KIFAP3	3.45E-01	7.47E-05	6.98E-04
ILMN_3256325	CYB561D1	3.44E-01	2.90E-05	3.12E-04
ILMN_3245476	PHRF1	3.44E-01	6.60E-03	3.27E-02
ILMN_2190598	FGD6	3.44E-01	5.34E-07	1.06E-05
ILMN_1692706	DCUN1D2	3.44E-01	3.69E-03	2.00E-02
ILMN_1714216	TSC2	3.44E-01	7.46E-07	1.40E-05
ILMN_1653047	DHX40	3.44E-01	2.04E-05	2.31E-04
ILMN_2088172	POLR2B	3.44E-01	7.86E-04	5.32E-03
ILMN_1804445	ATF7IP2	3.43E-01	5.84E-05	5.65E-04
ILMN_1738239	RBM6	3.43E-01	2.92E-04	2.28E-03
ILMN_1786139	VKORC1	3.43E-01	9.49E-07	1.71E-05
ILMN_3242091	NCRNA00094	3.43E-01	1.61E-04	1.35E-03
ILMN_1675709	ARFGAP1	3.43E-01	1.11E-04	9.82E-04
ILMN_1736911	TMOD1	3.43E-01	7.36E-04	5.03E-03
ILMN_1690523	LRRC20	3.43E-01	6.74E-03	3.33E-02
ILMN_2198393	KATNA1	3.43E-01	3.71E-05	3.83E-04
ILMN_2401927	TTC8	3.43E-01	4.03E-03	2.16E-02
ILMN_2067032	TRAF3IP1	3.42E-01	5.85E-07	1.14E-05
ILMN_3251592	C9orf140	3.42E-01	3.34E-03	1.84E-02
ILMN_1660847	PFKFB3	3.42E-01	7.97E-05	7.38E-04
ILMN_1709860	UNC45A	3.42E-01	1.83E-04	1.51E-03
ILMN_1657194	TSNAXIP1	3.42E-01	1.08E-06	1.90E-05
ILMN_1666512	SKIV2L	3.42E-01	1.53E-04	1.29E-03
ILMN_2363668	YIF1B	3.42E-01	1.34E-09	8.69E-08
ILMN_2205963	C10orf54	3.42E-01	1.96E-06	3.13E-05
ILMN_1794956	BBS9	3.42E-01	3.02E-03	1.69E-02
ILMN_1654289	ELK1	3.41E-01	5.63E-04	4.00E-03
ILMN_1732534	CHMP5	3.41E-01	1.16E-04	1.02E-03
ILMN_2185563	ANKRA2	3.41E-01	8.17E-05	7.53E-04
ILMN_2361603	NDRG2	3.41E-01	6.49E-04	4.52E-03
ILMN_1688246	LOC642852	3.41E-01	5.84E-07	1.14E-05
ILMN_1723536	USP33	3.41E-01	1.50E-06	2.50E-05
ILMN_2234310	GLTPD1	3.41E-01	1.26E-04	1.10E-03
ILMN_1760360	RNF160	3.41E-01	2.35E-06	3.67E-05
ILMN_1702124	LNX2	3.41E-01	4.44E-05	4.45E-04
ILMN_2365549	BRPF1	3.40E-01	1.31E-06	2.23E-05
ILMN_1682658	EPM2AIP1	3.40E-01	5.25E-04	3.77E-03
ILMN_1815859	ERCC2	3.40E-01	6.38E-04	4.46E-03
ILMN_2242068	GSTCD	3.40E-01	1.52E-05	1.79E-04
ILMN_1655177	PIK4CA	3.40E-01	1.77E-06	2.88E-05
ILMN_1699265	TNFRSF10B	3.40E-01	1.09E-02	4.98E-02
ILMN_1694147	PUS3	3.40E-01	5.54E-04	3.95E-03
ILMN_1810652	LMBRD2	3.40E-01	1.79E-05	2.06E-04
ILMN_1792726	TDRKH	3.40E-01	2.22E-07	5.13E-06

ILMN_2356909	MOSPD3	3.40E-01	3.83E-05	3.94E-04
ILMN_1807633	HRSP12	3.40E-01	6.93E-04	4.78E-03
ILMN_3243452	SNORD95	3.39E-01	7.32E-05	6.87E-04
ILMN_1683595	MBD1	3.39E-01	6.91E-07	1.31E-05
ILMN_2369924	NDUFB6	3.39E-01	8.68E-05	7.94E-04
ILMN_1707901	DHRS7B	3.39E-01	4.64E-06	6.60E-05
ILMN_1652846	PCYT2	3.39E-01	1.33E-05	1.60E-04
ILMN_1768449	PRPSAP1	3.39E-01	1.06E-03	6.87E-03
ILMN_2094166	CHMP5	3.39E-01	4.43E-05	4.45E-04
ILMN_1793241	SRD5A1	3.39E-01	7.54E-06	9.92E-05
ILMN_1653822	NEK2	3.39E-01	5.65E-06	7.79E-05
ILMN_1769601	MGC16169	3.38E-01	1.45E-05	1.72E-04
ILMN_1764873	ELAVL1	3.38E-01	1.19E-03	7.58E-03
ILMN_1733929	DNMT3B	3.38E-01	8.89E-05	8.11E-04
ILMN_1779185	CYTSA	3.38E-01	5.18E-06	7.22E-05
ILMN_1758337	ZNF213	3.38E-01	9.94E-06	1.25E-04
ILMN_2166972	BBS12	3.38E-01	1.35E-05	1.62E-04
ILMN_1807243	PRPF18	3.38E-01	5.83E-04	4.12E-03
ILMN_2148913	TMEM45A	3.38E-01	2.71E-03	1.54E-02
ILMN_1762003	SEC62	3.38E-01	5.49E-03	2.81E-02
ILMN_1737343	FNIP1	3.38E-01	7.91E-04	5.35E-03
ILMN_1792710	DAPK3	3.38E-01	3.84E-05	3.95E-04
ILMN_1758816	UGT8	3.38E-01	1.18E-07	3.04E-06
ILMN_3237627	LOC154761	3.37E-01	6.10E-06	8.30E-05
ILMN_1747344	IL3RA	3.37E-01	2.30E-04	1.84E-03
ILMN_1707815	SDHALP1	3.37E-01	3.06E-03	1.71E-02
ILMN_1707326	TASP1	3.37E-01	5.03E-09	2.41E-07
ILMN_2047240	ARSA	3.37E-01	9.81E-04	6.43E-03
ILMN_2207505	LEP	3.37E-01	9.14E-04	6.06E-03
ILMN_1680130	DYM	3.37E-01	4.73E-06	6.70E-05
ILMN_1695759	AMDHD2	3.37E-01	1.63E-03	9.93E-03
ILMN_1755643	MGAT4A	3.37E-01	1.13E-04	9.92E-04
ILMN_1715905	DSN1	3.36E-01	1.79E-05	2.07E-04
ILMN_3250427	LOC646278	3.36E-01	1.37E-04	1.17E-03
ILMN_1694233	ACYP1	3.36E-01	3.12E-05	3.31E-04
ILMN_1705750	TGM2	3.36E-01	5.60E-03	2.85E-02
ILMN_1761425	OLFML2A	3.36E-01	5.01E-10	4.11E-08
ILMN_1735553	MAP3K9	3.36E-01	2.94E-06	4.47E-05
ILMN_1685022	CAPN7	3.36E-01	2.41E-06	3.75E-05
ILMN_2130635	FOXRED2	3.35E-01	1.08E-02	4.97E-02
ILMN_3242211	TMEM187	3.35E-01	3.39E-06	5.06E-05
ILMN_1691119	RNF122	3.35E-01	3.91E-03	2.10E-02
ILMN_1679558	LOC283874	3.35E-01	3.62E-05	3.76E-04
ILMN_2413318	C15orf57	3.35E-01	3.24E-08	1.05E-06
ILMN_1738276	TMEM185A	3.35E-01	2.77E-04	2.17E-03
ILMN_1691980	FAM126A	3.34E-01	1.78E-07	4.25E-06

ILMN_1804988	MOAP1	3.34E-01	4.35E-03	2.30E-02
ILMN_1761309	ADCK5	3.34E-01	5.43E-04	3.88E-03
ILMN_1671265	ING2	3.34E-01	1.72E-06	2.81E-05
ILMN_2045994	SEPW1	3.34E-01	6.56E-04	4.56E-03
ILMN_1665212	EDC4	3.34E-01	7.42E-06	9.79E-05
ILMN_1787705	ATP6V1B2	3.34E-01	2.25E-03	1.31E-02
ILMN_1912997		3.34E-01	1.23E-03	7.77E-03
ILMN_3190596	LOC100129552	3.34E-01	2.11E-05	2.37E-04
ILMN_1742379	IFT122	3.34E-01	5.08E-06	7.12E-05
ILMN_2066066	HLA-DRB6	3.34E-01	2.52E-04	2.00E-03
ILMN_1712577	FAM174A	3.33E-01	7.26E-05	6.82E-04
ILMN_3241218	ANKIB1	3.33E-01	1.65E-05	1.93E-04
ILMN_2085722	ING2	3.33E-01	2.23E-05	2.49E-04
ILMN_2361737	TRIM36	3.33E-01	4.99E-04	3.61E-03
ILMN_2390114	AP3D1	3.33E-01	3.74E-06	5.52E-05
ILMN_1844692	FOXO3	3.33E-01	6.00E-03	3.02E-02
ILMN_1797342	FNBP1	3.33E-01	3.36E-04	2.57E-03
ILMN_1762115	CRYZL1	3.33E-01	4.34E-04	3.20E-03
ILMN_3244343	SNORA21	3.33E-01	2.62E-03	1.50E-02
ILMN_1666372	ATP5H	3.32E-01	4.95E-05	4.90E-04
ILMN_1782094	PAIP2	3.32E-01	5.89E-05	5.69E-04
ILMN_1815656	SERINC3	3.32E-01	2.16E-05	2.42E-04
ILMN_1805395	LTBP3	3.32E-01	9.69E-07	1.74E-05
ILMN_1684042	BET1	3.32E-01	3.45E-07	7.35E-06
ILMN_1813817	MRPL55	3.32E-01	7.01E-06	9.32E-05
ILMN_2086077	JUNB	3.32E-01	3.24E-03	1.79E-02
ILMN_1670970	PPP3CA	3.32E-01	3.51E-04	2.66E-03
ILMN_1708787	ZNF627	3.31E-01	1.52E-03	9.34E-03
ILMN_1704619	VPS29	3.31E-01	1.05E-06	1.86E-05
ILMN_2342437	KLHL5	3.31E-01	3.86E-04	2.89E-03
ILMN_1800611	CEP72	3.31E-01	7.61E-05	7.10E-04
ILMN_2394296	SAR1B	3.31E-01	3.58E-04	2.71E-03
ILMN_1766425	REPS2	3.31E-01	1.05E-04	9.34E-04
ILMN_1798619	KCTD17	3.31E-01	4.47E-05	4.48E-04
ILMN_1845157		3.31E-01	8.72E-04	5.82E-03
ILMN_2181125	NAPB	3.30E-01	1.95E-04	1.60E-03
ILMN_1730491	FMNL2	3.30E-01	1.82E-04	1.51E-03
ILMN_1792518	STX7	3.30E-01	6.93E-04	4.78E-03
ILMN_1660729	ATP6V1C2	3.30E-01	1.97E-04	1.61E-03
ILMN_2103774	PIP5KL1	3.30E-01	3.35E-05	3.51E-04
ILMN_1754660	ZCCHC24	3.29E-01	2.71E-03	1.54E-02
ILMN_1693669	WDR79	3.29E-01	7.60E-05	7.09E-04
ILMN_2383455	SUOX	3.29E-01	6.98E-04	4.81E-03
ILMN_1781987	CDK5	3.29E-01	3.25E-04	2.50E-03
ILMN_1719064	KCTD10	3.29E-01	9.76E-07	1.75E-05
ILMN_1794560	TMEM93	3.29E-01	5.51E-03	2.81E-02

ILMN_2313821	AIFM1	3.29E-01	2.41E-03	1.39E-02
ILMN_1713301	DGCR2	3.28E-01	1.40E-05	1.67E-04
ILMN_1760490	ACVR1	3.28E-01	3.13E-04	2.42E-03
ILMN_1717165	IGBP1	3.28E-01	3.10E-03	1.73E-02
ILMN_1861057		3.28E-01	4.32E-06	6.23E-05
ILMN_1654287	ADCY9	3.28E-01	1.05E-02	4.86E-02
ILMN_2352023	RIPK5	3.28E-01	3.58E-08	1.15E-06
ILMN_2310703	RPS26L	3.28E-01	7.72E-04	5.24E-03
ILMN_2364072	CLCNKA	3.28E-01	1.16E-03	7.43E-03
ILMN_1707257	HIST1H3J	3.27E-01	9.90E-03	4.61E-02
ILMN_2144791	GOLGA6B	3.27E-01	1.10E-06	1.93E-05
ILMN_1724148	ORAI1	3.27E-01	1.63E-07	3.96E-06
ILMN_3200322	LOC387791	3.27E-01	1.30E-03	8.14E-03
ILMN_1693210	NSMCE2	3.27E-01	2.93E-05	3.14E-04
ILMN_1757347	C22orf9	3.27E-01	1.40E-03	8.66E-03
ILMN_1656886	LIN37	3.27E-01	1.51E-07	3.72E-06
ILMN_2391976	SLC45A4	3.27E-01	1.26E-05	1.53E-04
ILMN_1761049	EIF2C3	3.27E-01	5.65E-07	1.11E-05
ILMN_2228044	TBC1D23	3.27E-01	2.19E-05	2.45E-04
ILMN_1735062	KCNG2	3.26E-01	1.25E-05	1.52E-04
ILMN_1764163	LOC644330	3.26E-01	4.40E-03	2.32E-02
ILMN_1779616	SUCLG1	3.26E-01	2.38E-05	2.63E-04
ILMN_2399627	AP1G1	3.26E-01	3.03E-05	3.24E-04
ILMN_1754656	TCP10L	3.26E-01	3.56E-03	1.94E-02
ILMN_2379644	CD74	3.26E-01	9.29E-03	4.37E-02
ILMN_2382126	PPFIA1	3.26E-01	4.20E-03	2.23E-02
ILMN_1733045	RAB36	3.26E-01	9.85E-06	1.24E-04
ILMN_2094313	ZDHHC1	3.26E-01	6.99E-07	1.32E-05
ILMN_1741096	FDFT1	3.26E-01	3.25E-03	1.80E-02
ILMN_2175912	ITGB2	3.26E-01	4.66E-05	4.65E-04
ILMN_1791423	LOC401052	3.25E-01	8.66E-05	7.92E-04
ILMN_1734288	DUSP18	3.25E-01	2.03E-05	2.30E-04
ILMN_1776375	PIN1	3.25E-01	6.32E-04	4.42E-03
ILMN_1802190	FOXJ1	3.25E-01	2.93E-03	1.64E-02
ILMN_2065299	EDEM3	3.25E-01	2.84E-05	3.06E-04
ILMN_1655563	KIAA0427	3.24E-01	7.85E-07	1.46E-05
ILMN_1740685	LOC652541	3.24E-01	1.79E-05	2.07E-04
ILMN_3301740	LOC729887	3.24E-01	1.04E-03	6.75E-03
ILMN_1754103	CLDN11	3.24E-01	3.17E-05	3.36E-04
ILMN_3243744	EAPP	3.24E-01	1.58E-03	9.62E-03
ILMN_1712035	TMEM115	3.23E-01	3.28E-06	4.91E-05
ILMN_1736154	ProSAPiP1	3.23E-01	1.50E-05	1.77E-04
ILMN_1677038	FLJ21986	3.23E-01	1.06E-08	4.32E-07
ILMN_1806010	CUL9	3.23E-01	1.86E-04	1.54E-03
ILMN_2367165	ABTB1	3.23E-01	9.18E-07	1.66E-05
ILMN_1707308	IKBKG	3.23E-01	1.42E-08	5.49E-07

ILMN_3303673	LOC729852	3.22E-01	5.57E-06	7.69E-05
ILMN_1807712	PILRB	3.22E-01	1.28E-04	1.11E-03
ILMN_1726928	TCEA3	3.22E-01	7.64E-04	5.18E-03
ILMN_1731720	PDRG1	3.22E-01	9.90E-05	8.88E-04
ILMN_1808071	KIF14	3.22E-01	8.15E-03	3.91E-02
ILMN_1757914	C19orf56	3.22E-01	6.35E-04	4.44E-03
ILMN_1694539	MAP3K6	3.22E-01	3.62E-03	1.96E-02
ILMN_2376194	CAMK2B	3.22E-01	5.48E-03	2.80E-02
ILMN_1762932	CHMP2A	3.22E-01	5.94E-06	8.12E-05
ILMN_1804351	FZD7	3.21E-01	8.14E-06	1.06E-04
ILMN_1714700	TRIB2	3.21E-01	1.27E-07	3.24E-06
ILMN_1766094	MOSPD2	3.21E-01	1.30E-03	8.17E-03
ILMN_2347592	NMB	3.21E-01	1.36E-05	1.63E-04
ILMN_1793040	ADAMTSL5	3.21E-01	4.30E-07	8.78E-06
ILMN_1782611	LOC643870	3.21E-01	7.15E-06	9.48E-05
ILMN_1810875	SYNGR1	3.21E-01	1.06E-05	1.33E-04
ILMN_1741613	SERINC1	3.21E-01	3.46E-04	2.64E-03
ILMN_2117323	PIK3C2B	3.21E-01	2.53E-05	2.77E-04
ILMN_2346479	HOMER2	3.20E-01	2.18E-04	1.76E-03
ILMN_3235657	SRGAP2L	3.20E-01	1.58E-07	3.85E-06
ILMN_2134110	SELK	3.20E-01	1.21E-04	1.06E-03
ILMN_3258628	INPP5K	3.20E-01	1.02E-05	1.28E-04
ILMN_1809094	LOC643396	3.20E-01	4.98E-05	4.92E-04
ILMN_1766359	GATAD2B	3.20E-01	3.06E-04	2.37E-03
ILMN_2399622	AP1G1	3.20E-01	2.80E-05	3.02E-04
ILMN_1657050	ARSK	3.20E-01	1.50E-06	2.50E-05
ILMN_1722066	ARMC1	3.20E-01	5.94E-07	1.15E-05
ILMN_3269119	LOC100130155	3.20E-01	2.19E-03	1.28E-02
ILMN_1688479	LRRC42	3.19E-01	1.15E-04	1.01E-03
ILMN_2090123	DHX29	3.19E-01	1.17E-04	1.02E-03
ILMN_1910330		3.19E-01	3.22E-06	4.83E-05
ILMN_1777853	MBOAT2	3.19E-01	1.92E-06	3.08E-05
ILMN_1702696	AFAR3	3.19E-01	4.77E-07	9.60E-06
ILMN_1700147	VPREB3	3.19E-01	2.33E-04	1.86E-03
ILMN_1807448	FAM8A1	3.19E-01	7.52E-03	3.66E-02
ILMN_1770425	CDIPT	3.19E-01	3.34E-04	2.56E-03
ILMN_2162860	SLFN11	3.19E-01	1.10E-05	1.37E-04
ILMN_1763464	SLC25A42	3.19E-01	2.75E-07	6.06E-06
ILMN_1657317	POLR2J	3.19E-01	1.13E-03	7.25E-03
ILMN_1685260	DNM1L	3.19E-01	1.01E-05	1.27E-04
ILMN_1674706	MTHFD2	3.18E-01	1.04E-02	4.81E-02
ILMN_1721629	ZNF654	3.18E-01	1.18E-04	1.03E-03
ILMN_1768595	DLG4	3.18E-01	6.80E-06	9.10E-05
ILMN_1714756	YIPF5	3.18E-01	9.73E-03	4.55E-02
ILMN_2334765	ARMCX3	3.18E-01	3.57E-05	3.71E-04
ILMN_1652777	CDC42EP2	3.18E-01	2.36E-04	1.89E-03

ILMN_1733155	GIT1	3.18E-01	9.53E-05	8.60E-04
ILMN_2121207	FAM125A	3.18E-01	4.39E-06	6.31E-05
ILMN_2285713	TDP1	3.18E-01	8.65E-03	4.12E-02
ILMN_1688158	CYB5R4	3.18E-01	7.20E-05	6.78E-04
ILMN_1810127	ZNF789	3.17E-01	2.04E-03	1.21E-02
ILMN_2180866	RPS26P11	3.17E-01	5.03E-03	2.61E-02
ILMN_2402172	SEPT4	3.17E-01	2.82E-03	1.59E-02
ILMN_1815168	HVCN1	3.17E-01	9.39E-07	1.70E-05
ILMN_1719517	CTTNBP2NL	3.17E-01	1.12E-06	1.96E-05
ILMN_1763461	ALDH7A1	3.17E-01	1.92E-03	1.14E-02
ILMN_2348403	VRK3	3.16E-01	5.02E-05	4.95E-04
ILMN_1763000	ADAP2	3.16E-01	1.53E-07	3.77E-06
ILMN_1659206	RARA	3.16E-01	8.59E-06	1.11E-04
ILMN_2143261	CXorf40B	3.16E-01	2.81E-04	2.20E-03
ILMN_3250972	REPS2	3.16E-01	4.17E-07	8.59E-06
ILMN_3228294	LOC729279	3.16E-01	3.35E-03	1.84E-02
ILMN_2405078	OSBPL8	3.15E-01	1.57E-04	1.32E-03
ILMN_1857017		3.15E-01	8.31E-06	1.08E-04
ILMN_1772459	RPS23	3.15E-01	1.41E-04	1.21E-03
ILMN_3234089	N4BP2L2	3.15E-01	8.11E-05	7.49E-04
ILMN_1773809	FOXP4	3.15E-01	1.74E-07	4.19E-06
ILMN_1804396	C14orf4	3.14E-01	5.58E-07	1.10E-05
ILMN_1806408	ACADVL	3.14E-01	1.08E-05	1.34E-04
ILMN_2353358	LGALS8	3.14E-01	3.72E-06	5.49E-05
ILMN_3239284	B9D1	3.14E-01	3.38E-07	7.23E-06
ILMN_1772329	LRRFIP2	3.14E-01	1.63E-06	2.69E-05
ILMN_1691131	LSMD1	3.14E-01	1.09E-03	7.02E-03
ILMN_1686906	TP53INP2	3.14E-01	2.44E-05	2.69E-04
ILMN_1809708	KCTD21	3.14E-01	1.03E-06	1.83E-05
ILMN_1665164	CTR9	3.14E-01	1.27E-04	1.10E-03
ILMN_2396287	RFX2	3.14E-01	2.48E-06	3.85E-05
ILMN_3199780	LOC401076	3.14E-01	7.44E-03	3.62E-02
ILMN_3249807	SKA2	3.14E-01	8.19E-06	1.07E-04
ILMN_1739942	FAM117B	3.13E-01	4.87E-04	3.53E-03
ILMN_1693317	TTC15	3.13E-01	3.55E-03	1.94E-02
ILMN_1660519	C3orf70	3.13E-01	7.95E-03	3.83E-02
ILMN_1762972	CHD9	3.13E-01	5.97E-04	4.21E-03
ILMN_1879480		3.13E-01	1.03E-04	9.16E-04
ILMN_1803302	CRK	3.13E-01	9.19E-04	6.09E-03
ILMN_1775380	SMOX	3.13E-01	2.31E-05	2.56E-04
ILMN_1781867	FOXD4L1	3.13E-01	1.33E-05	1.60E-04
ILMN_1762835	HELZ	3.13E-01	2.04E-06	3.24E-05
ILMN_1699521	KIAA1641	3.13E-01	1.00E-03	6.54E-03
ILMN_1900998		3.13E-01	5.33E-06	7.42E-05
ILMN_3283772	LOC644237	3.13E-01	1.36E-05	1.63E-04
ILMN_2052871	TMEM116	3.13E-01	7.31E-06	9.65E-05

ILMN_1721349	MAGT1	3.13E-01	7.61E-04	5.16E-03
ILMN_1688637	TMEM198	3.13E-01	8.36E-04	5.61E-03
ILMN_2396148	HIP1R	3.13E-01	3.59E-04	2.71E-03
ILMN_1668134	GSTM1	3.12E-01	7.52E-04	5.12E-03
ILMN_1785765	TM9SF2	3.12E-01	2.76E-05	2.99E-04
ILMN_1655876	TMEM159	3.12E-01	5.26E-03	2.71E-02
ILMN_1659075	HLA-DOA	3.12E-01	9.05E-06	1.16E-04
ILMN_1800311	HSF2	3.12E-01	4.42E-05	4.44E-04
ILMN_1662161	TBC1D13	3.12E-01	6.84E-06	9.12E-05
ILMN_1681252	C17orf44	3.12E-01	5.07E-04	3.65E-03
ILMN_1688071	NAT1	3.12E-01	4.27E-03	2.27E-02
ILMN_1794157	CATSPER2P1	3.12E-01	4.43E-05	4.45E-04
ILMN_1777129	C16orf56	3.12E-01	1.36E-05	1.63E-04
ILMN_1779010	MAP3K3	3.12E-01	2.59E-05	2.83E-04
ILMN_1773307	NAP1L5	3.12E-01	6.29E-03	3.14E-02
ILMN_1665982	AKTIP	3.12E-01	2.86E-03	1.61E-02
ILMN_1786843	KCTD13	3.11E-01	7.37E-04	5.04E-03
ILMN_1755411	RBMS2	3.11E-01	1.24E-05	1.51E-04
ILMN_1760347	SRGN	3.11E-01	6.36E-06	8.59E-05
ILMN_1666007	TRAPPC6B	3.11E-01	7.57E-04	5.14E-03
ILMN_2276952	TSC22D3	3.11E-01	8.62E-03	4.11E-02
ILMN_1767848	PCMTD2	3.11E-01	5.40E-04	3.86E-03
ILMN_2407619	CDC25C	3.10E-01	1.96E-07	4.63E-06
ILMN_1830367		3.10E-01	3.99E-04	2.98E-03
ILMN_1743373	DLL1	3.10E-01	7.53E-04	5.12E-03
ILMN_1798164	PHF3	3.10E-01	1.74E-07	4.19E-06
ILMN_1769637	RNMT	3.10E-01	1.25E-03	7.91E-03
ILMN_1730568	ZDHHC7	3.10E-01	7.88E-04	5.33E-03
ILMN_1651438	ZFPM1	3.09E-01	2.36E-03	1.37E-02
ILMN_1735608	C19orf47	3.09E-01	2.36E-06	3.68E-05
ILMN_1786920	JARID1A	3.09E-01	1.51E-05	1.78E-04
ILMN_3235104	KIAA1731	3.09E-01	6.37E-03	3.18E-02
ILMN_1715832	PIK3R4	3.09E-01	3.61E-06	5.34E-05
ILMN_1793563	DCTN1	3.09E-01	7.60E-06	9.99E-05
ILMN_3213692	LOC441073	3.09E-01	1.97E-04	1.61E-03
ILMN_1659082	ZCRB1	3.09E-01	1.13E-05	1.40E-04
ILMN_1686319	USP37	3.09E-01	3.43E-06	5.11E-05
ILMN_1735045	A4GALT	3.09E-01	4.32E-04	3.19E-03
ILMN_1772189	ABCD1	3.09E-01	2.34E-05	2.59E-04
ILMN_2406501	SOD2	3.08E-01	1.65E-05	1.93E-04
ILMN_1731113	ZBTB43	3.08E-01	6.83E-07	1.30E-05
ILMN_2152828	KIF16B	3.08E-01	7.26E-04	4.98E-03
ILMN_1668246	TMEM52	3.08E-01	1.13E-05	1.39E-04
ILMN_1758055	YIF1B	3.08E-01	4.61E-05	4.61E-04
ILMN_2124187	TSC22D2	3.08E-01	6.57E-04	4.57E-03
ILMN_3249188	HIST2H3D	3.08E-01	6.55E-03	3.25E-02

ILMN_1733318	TEX9	3.08E-01	3.09E-05	3.29E-04
ILMN_1714956	PLA2G12A	3.07E-01	2.27E-05	2.53E-04
ILMN_1656185	DEF8	3.07E-01	3.62E-03	1.97E-02
ILMN_1651610	LOC730525	3.07E-01	4.60E-04	3.36E-03
ILMN_1682781	TEAD2	3.07E-01	7.01E-07	1.32E-05
ILMN_1740486	POLR2J4	3.07E-01	6.32E-06	8.55E-05
ILMN_3237507	LOC552889	3.07E-01	1.81E-04	1.49E-03
ILMN_1708881	RAB20	3.07E-01	8.77E-04	5.85E-03
ILMN_1681135	SPATA2	3.07E-01	7.01E-04	4.83E-03
ILMN_1794912	ATP5H	3.07E-01	3.73E-06	5.51E-05
ILMN_1807981	SIGIRR	3.07E-01	7.44E-04	5.07E-03
ILMN_1706664	FAM80A	3.07E-01	3.21E-06	4.82E-05
ILMN_1787514	CAPN12	3.07E-01	4.24E-03	2.25E-02
ILMN_1770742	TMEM55B	3.06E-01	6.44E-03	3.21E-02
ILMN_1672405	TMED7	3.06E-01	3.54E-06	5.26E-05
ILMN_1764945	AP3D1	3.06E-01	4.23E-06	6.10E-05
ILMN_1790549	TSPAN3	3.06E-01	1.47E-03	9.07E-03
ILMN_3241996	C6orf59	3.06E-01	1.47E-07	3.65E-06
ILMN_1804522	CCDC47	3.06E-01	9.47E-06	1.20E-04
ILMN_2328986	SREBF1	3.06E-01	2.64E-05	2.87E-04
ILMN_1756152	MFSD11	3.06E-01	5.23E-04	3.76E-03
ILMN_2380801	FYN	3.06E-01	7.35E-07	1.38E-05
ILMN_2218780	PPM2C	3.05E-01	1.17E-03	7.49E-03
ILMN_1772487	SFRS14	3.05E-01	1.50E-04	1.27E-03
ILMN_1741180	HEXDC	3.05E-01	4.13E-07	8.52E-06
ILMN_2166457	HPGD	3.05E-01	1.47E-03	9.09E-03
ILMN_3245912	TMEM59L	3.05E-01	3.31E-03	1.83E-02
ILMN_1724139	TMEM123	3.05E-01	2.79E-04	2.19E-03
ILMN_1663605	RNF123	3.05E-01	1.21E-05	1.49E-04
ILMN_1793854	INTS1	3.05E-01	2.62E-05	2.85E-04
ILMN_1789793	NUAK2	3.05E-01	2.98E-03	1.67E-02
ILMN_1664644	ATG16L2	3.05E-01	1.08E-06	1.91E-05
ILMN_2369666	CR2	3.05E-01	1.12E-04	9.86E-04
ILMN_1664776	EFR3A	3.04E-01	2.13E-06	3.37E-05
ILMN_1754531	AP4E1	3.04E-01	3.05E-04	2.37E-03
ILMN_1757660	CAPS	3.04E-01	8.68E-05	7.94E-04
ILMN_2402363	STK19	3.04E-01	5.72E-05	5.55E-04
ILMN_2387471	FLJ22184	3.04E-01	1.05E-02	4.84E-02
ILMN_1704253	C6orf106	3.04E-01	5.65E-05	5.49E-04
ILMN_2206554	SLC35F5	3.04E-01	1.24E-05	1.51E-04
ILMN_1710284	HES1	3.04E-01	1.05E-02	4.85E-02
ILMN_1761963	C4orf29	3.03E-01	6.38E-04	4.46E-03
ILMN_3231577	LOC100134648	3.03E-01	1.67E-07	4.03E-06
ILMN_2220184	GFPT1	3.03E-01	1.90E-03	1.13E-02
ILMN_1898022		3.03E-01	1.91E-04	1.57E-03
ILMN_1811555	C9orf119	3.03E-01	8.71E-03	4.14E-02

ILMN_1677301	LOC653082	3.03E-01	6.20E-06	8.41E-05
ILMN_1812096	CADM4	3.03E-01	7.22E-05	6.79E-04
ILMN_2349124	OSBPL2	3.03E-01	3.07E-03	1.71E-02
ILMN_2097185	PUS3	3.03E-01	6.03E-04	4.25E-03
ILMN_1773553	FLJ46906	3.03E-01	4.01E-03	2.15E-02
ILMN_2323933	LAIR2	3.03E-01	6.71E-06	8.98E-05
ILMN_1654685	MCTP1	3.03E-01	2.49E-04	1.98E-03
ILMN_2346831	MGAT2	3.02E-01	3.11E-04	2.41E-03
ILMN_1824151		3.02E-01	4.61E-03	2.42E-02
ILMN_1752333	SLC35E1	3.02E-01	3.74E-04	2.82E-03
ILMN_1761175	RPS6KB2	3.02E-01	7.78E-04	5.27E-03
ILMN_1784110	PCTK3	3.02E-01	6.64E-04	4.61E-03
ILMN_1736623	NCKIPSD	3.02E-01	1.87E-03	1.12E-02
ILMN_2097259	CYP2U1	3.02E-01	3.59E-08	1.15E-06
ILMN_2230035	BBS2	3.01E-01	4.21E-04	3.12E-03
ILMN_2412571	TUBGCP6	3.01E-01	1.42E-05	1.69E-04
ILMN_1708782	MFAP3	3.01E-01	4.23E-05	4.28E-04
ILMN_1736704	DIXDC1	3.01E-01	7.48E-08	2.07E-06
ILMN_1749474	FAM7A1	3.00E-01	1.00E-04	8.96E-04
ILMN_2364272	MBNL2	3.00E-01	3.41E-05	3.57E-04
ILMN_2309228	GPS1	3.00E-01	1.07E-05	1.33E-04
ILMN_1654697	ZNF280B	3.00E-01	2.99E-05	3.19E-04
ILMN_2158242	SHOC2	3.00E-01	5.08E-04	3.66E-03
ILMN_3187852	KIAA1310	3.00E-01	1.17E-04	1.03E-03
ILMN_1805345	MOSPD3	3.00E-01	1.94E-04	1.59E-03
ILMN_1752631	CGGBP1	2.99E-01	1.32E-05	1.59E-04
ILMN_1665331	AMT	2.99E-01	5.37E-03	2.75E-02
ILMN_2364088	GEMIN8	2.99E-01	7.79E-06	1.02E-04
ILMN_1673604	YIPF3	2.99E-01	2.37E-05	2.62E-04
ILMN_1757702	LOC647673	2.99E-01	4.93E-03	2.56E-02
ILMN_2311278	ADD3	2.99E-01	1.43E-04	1.22E-03
ILMN_1654396	ITGB2	2.99E-01	5.25E-06	7.32E-05
ILMN_1747223	FRYL	2.99E-01	4.68E-03	2.45E-02
ILMN_3215461	LOC100131989	2.99E-01	3.45E-03	1.89E-02
ILMN_2115379	ERP44	2.99E-01	2.08E-08	7.46E-07
ILMN_2353697	HINFP	2.99E-01	5.85E-05	5.65E-04
ILMN_1659874	SFRS15	2.98E-01	3.33E-05	3.50E-04
ILMN_1706357	RFX2	2.98E-01	2.01E-03	1.19E-02
ILMN_1657977	MSRB2	2.98E-01	4.27E-05	4.32E-04
ILMN_1813475	HERC2	2.98E-01	3.37E-04	2.58E-03
ILMN_1729976	ZNF828	2.98E-01	6.93E-03	3.41E-02
ILMN_2282352	PHYH	2.98E-01	3.48E-04	2.64E-03
ILMN_1687335	FLNA	2.98E-01	1.12E-03	7.18E-03
ILMN_1665117	C6orf89	2.98E-01	4.24E-04	3.14E-03
ILMN_1693630	C16orf7	2.98E-01	3.39E-05	3.55E-04
ILMN_1781468	SMAP2	2.98E-01	2.28E-04	1.84E-03

ILMN_1773066	CDKN2AIP	2.98E-01	3.54E-03	1.93E-02
ILMN_1660973	GAD1	2.98E-01	1.19E-03	7.56E-03
ILMN_2358980	ILK	2.98E-01	5.79E-04	4.10E-03
ILMN_1788239	AMDHD1	2.97E-01	2.27E-05	2.53E-04
ILMN_1695317	RCBTB1	2.97E-01	3.12E-05	3.32E-04
ILMN_1669718	PSENEN	2.97E-01	1.52E-04	1.29E-03
ILMN_1733931	PDCD6	2.97E-01	5.17E-06	7.22E-05
ILMN_1776109	ZNF622	2.97E-01	5.73E-04	4.07E-03
ILMN_1664577	DLD	2.97E-01	3.24E-04	2.49E-03
ILMN_1760688	SAMD14	2.96E-01	3.94E-05	4.03E-04
ILMN_1732725	SAPS3	2.96E-01	6.63E-03	3.29E-02
ILMN_1691053	LOC91561	2.96E-01	1.43E-04	1.22E-03
ILMN_1707070	PCOLCE	2.96E-01	5.06E-06	7.10E-05
ILMN_3286312	LOC642458	2.96E-01	2.27E-05	2.53E-04
ILMN_2408796	C19orf28	2.96E-01	3.21E-03	1.78E-02
ILMN_1678522	LOC644934	2.96E-01	2.26E-03	1.32E-02
ILMN_1695585	RPS26L	2.96E-01	3.33E-03	1.83E-02
ILMN_2269136	AGAP3	2.96E-01	1.93E-08	7.03E-07
ILMN_1780292	MSH5	2.96E-01	9.77E-04	6.41E-03
ILMN_1728605	TTC3	2.95E-01	5.29E-05	5.18E-04
ILMN_1687896	PIK3C3	2.95E-01	1.84E-03	1.10E-02
ILMN_1776656	BBS7	2.95E-01	1.00E-04	8.97E-04
ILMN_1748908	PROSC	2.95E-01	8.36E-04	5.61E-03
ILMN_1702229	CECR6	2.95E-01	3.84E-05	3.95E-04
ILMN_1686981	SULF2	2.95E-01	8.06E-03	3.88E-02
ILMN_2394498	SYF2	2.95E-01	3.77E-04	2.83E-03
ILMN_1742456	OSTF1	2.95E-01	1.94E-06	3.11E-05
ILMN_2278561	RABL2A	2.95E-01	7.74E-03	3.75E-02
ILMN_1683305	COMMD2	2.95E-01	7.70E-05	7.17E-04
ILMN_1689518	PECAM1	2.95E-01	1.92E-06	3.07E-05
ILMN_1671250	CLIC4	2.95E-01	3.75E-06	5.53E-05
ILMN_2060212	TBC1D24	2.95E-01	7.78E-04	5.27E-03
ILMN_1795338	YPEL1	2.94E-01	2.78E-04	2.18E-03
ILMN_1684217	AURKB	2.94E-01	3.33E-04	2.56E-03
ILMN_1752008	OTUD1	2.94E-01	5.83E-04	4.13E-03
ILMN_1855325		2.94E-01	9.17E-06	1.17E-04
ILMN_2054053	TMEM67	2.94E-01	7.88E-04	5.33E-03
ILMN_2151075	ZNF821	2.94E-01	2.46E-04	1.96E-03
ILMN_1712390	CUTA	2.94E-01	1.03E-05	1.29E-04
ILMN_2182120	SF3B14	2.94E-01	4.88E-03	2.54E-02
ILMN_2135339	C3orf70	2.94E-01	7.12E-04	4.89E-03
ILMN_2405797	REPS2	2.93E-01	4.34E-03	2.30E-02
ILMN_1786470	C1orf74	2.93E-01	6.29E-04	4.40E-03
ILMN_1750661	FBXW9	2.93E-01	6.35E-05	6.07E-04
ILMN_1781281	EPPB9	2.93E-01	9.67E-06	1.22E-04
ILMN_1719286	CTSA	2.93E-01	1.20E-03	7.62E-03

ILMN_1791656	C10orf137	2.93E-01	5.44E-05	5.32E-04
ILMN_1782110	ZNF295	2.93E-01	8.11E-03	3.90E-02
ILMN_2367070	ACOT9	2.93E-01	6.14E-03	3.08E-02
ILMN_3301033	LOC729217	2.93E-01	6.99E-06	9.31E-05
ILMN_1701613	RARRES3	2.93E-01	1.35E-03	8.42E-03
ILMN_2339202	KTN1	2.93E-01	1.18E-04	1.03E-03
ILMN_1750338	C10orf47	2.93E-01	7.92E-03	3.82E-02
ILMN_2196588	C18orf32	2.92E-01	4.75E-03	2.48E-02
ILMN_2150294	FKBP14	2.92E-01	4.75E-03	2.48E-02
ILMN_1781174	KIAA1009	2.92E-01	2.68E-04	2.11E-03
ILMN_1713402	FAM160A2	2.92E-01	3.77E-03	2.04E-02
ILMN_3238375	CENPT	2.92E-01	8.81E-06	1.13E-04
ILMN_1787308	PIP4K2C	2.92E-01	4.31E-04	3.18E-03
ILMN_1814823	FTL	2.92E-01	1.10E-04	9.76E-04
ILMN_1687303	ACAD10	2.92E-01	1.60E-03	9.75E-03
ILMN_1771048	LOC728153	2.92E-01	3.94E-03	2.12E-02
ILMN_1826531		2.92E-01	1.97E-03	1.17E-02
ILMN_1746784	SLAIN1	2.92E-01	6.94E-04	4.79E-03
ILMN_1676984	DDIT3	2.91E-01	6.07E-04	4.27E-03
ILMN_1787576	CLCNKA	2.91E-01	1.47E-03	9.07E-03
ILMN_1763433	TRIM9	2.91E-01	1.93E-03	1.15E-02
ILMN_1757910	HIP1R	2.91E-01	5.20E-05	5.11E-04
ILMN_1683658	FKBP1A	2.91E-01	2.07E-03	1.22E-02
ILMN_2344216	STX2	2.91E-01	2.98E-03	1.67E-02
ILMN_1786278	FAM149A	2.91E-01	1.73E-03	1.04E-02
ILMN_1679460	PPFIBP1	2.91E-01	3.76E-05	3.88E-04
ILMN_1656184	PI4KAP1	2.90E-01	3.53E-03	1.93E-02
ILMN_1791905	CCDC103	2.90E-01	1.97E-04	1.61E-03
ILMN_1790472	SLC25A28	2.90E-01	9.76E-04	6.40E-03
ILMN_1652533	DKFZp434K191	2.89E-01	3.48E-05	3.63E-04
ILMN_1809850	RCN3	2.89E-01	2.76E-03	1.56E-02
ILMN_2269564	ARID4B	2.89E-01	1.08E-02	4.97E-02
ILMN_1657983	TERF2IP	2.89E-01	4.48E-03	2.36E-02
ILMN_3235168	MUL1	2.89E-01	6.18E-07	1.19E-05
ILMN_1653711	FZD2	2.89E-01	2.37E-05	2.62E-04
ILMN_1812169	SERAC1	2.88E-01	2.93E-04	2.29E-03
ILMN_2407703	SYN1	2.88E-01	7.42E-04	5.06E-03
ILMN_2210729	STYXL1	2.88E-01	1.17E-06	2.03E-05
ILMN_1664978	TJP2	2.88E-01	1.39E-03	8.62E-03
ILMN_2241775	TROVE2	2.88E-01	2.20E-03	1.28E-02
ILMN_1790555	CCDC146	2.88E-01	1.57E-07	3.84E-06
ILMN_1745423	UTP3	2.88E-01	5.24E-03	2.70E-02
ILMN_1709043	C9orf46	2.87E-01	8.58E-05	7.86E-04
ILMN_1787885	NUDT18	2.87E-01	9.63E-05	8.67E-04
ILMN_1652369	ERCC1	2.87E-01	1.05E-04	9.34E-04
ILMN_1657475	GALT	2.87E-01	4.84E-05	4.80E-04

ILMN_2075847	ZC3H10	2.87E-01	2.42E-07	5.48E-06
ILMN_1669645	PKD1	2.87E-01	2.56E-03	1.47E-02
ILMN_1800590	BBS1	2.87E-01	1.08E-02	4.97E-02
ILMN_1756417	ANKRD37	2.87E-01	1.64E-03	9.98E-03
ILMN_1768962	AKAP8L	2.86E-01	4.29E-03	2.28E-02
ILMN_1682930	SIPA1	2.86E-01	4.60E-03	2.42E-02
ILMN_1783735	LOC649639	2.86E-01	2.14E-06	3.38E-05
ILMN_3216336	LOC285741	2.86E-01	1.16E-03	7.40E-03
ILMN_1774387	ZHX3	2.86E-01	4.91E-06	6.92E-05
ILMN_1692844	TBC1D19	2.86E-01	4.97E-05	4.91E-04
ILMN_2397721	GLB1	2.86E-01	8.27E-05	7.61E-04
ILMN_2211950	SRP14P1	2.86E-01	4.40E-05	4.43E-04
ILMN_1685415	HBP1	2.85E-01	6.74E-04	4.67E-03
ILMN_1700307	FLJ38969	2.85E-01	8.93E-05	8.14E-04
ILMN_1660439	LOC651149	2.85E-01	1.24E-03	7.85E-03
ILMN_3246097	LOC649839	2.85E-01	3.36E-04	2.57E-03
ILMN_2330787	FRMD6	2.85E-01	5.60E-06	7.73E-05
ILMN_2376416	UBXN11	2.85E-01	4.30E-03	2.28E-02
ILMN_2115949	ZNF394	2.85E-01	7.13E-03	3.49E-02
ILMN_3304111	LOC729978	2.85E-01	2.20E-05	2.47E-04
ILMN_1689274	NIPA1	2.85E-01	1.16E-03	7.42E-03
ILMN_1675354	TncRNA	2.85E-01	4.69E-03	2.45E-02
ILMN_1782829	GLTSCR1	2.85E-01	1.25E-03	7.90E-03
ILMN_2098437	FAM10A4	2.85E-01	3.43E-04	2.61E-03
ILMN_1700042	TLN2	2.85E-01	7.57E-06	9.95E-05
ILMN_2240597	TCEA2	2.85E-01	1.04E-04	9.28E-04
ILMN_2385866	PHF1	2.85E-01	5.83E-06	8.00E-05
ILMN_1740500	TSPYL4	2.85E-01	1.80E-03	1.08E-02
ILMN_2405305	ARNTL	2.84E-01	4.08E-03	2.18E-02
ILMN_1763887	SFI1	2.84E-01	2.71E-04	2.14E-03
ILMN_1781943	FAM83D	2.84E-01	8.46E-06	1.09E-04
ILMN_2408576	FAM129B	2.84E-01	1.62E-03	9.83E-03
ILMN_2116811	C7orf70	2.84E-01	9.37E-05	8.48E-04
ILMN_1684155	FLJ23584	2.84E-01	5.26E-04	3.77E-03
ILMN_1777322	FAM91A1	2.84E-01	8.88E-05	8.10E-04
ILMN_2201966	N4BP1	2.84E-01	4.06E-04	3.02E-03
ILMN_2285802	SEC14L1	2.83E-01	6.29E-05	6.03E-04
ILMN_1764166	BCKDHB	2.83E-01	7.74E-07	1.44E-05
ILMN_1655921	GTF2E1	2.83E-01	1.20E-03	7.61E-03
ILMN_1757230	ZNF250	2.83E-01	3.36E-05	3.53E-04
ILMN_2395373	GABBR1	2.83E-01	6.40E-04	4.47E-03
ILMN_2360291	UGCGL1	2.83E-01	1.73E-04	1.44E-03
ILMN_1812392	TMSB10	2.83E-01	5.98E-04	4.22E-03
ILMN_2295987	NBPF1	2.83E-01	5.85E-05	5.66E-04
ILMN_1746492	RABL4	2.83E-01	2.12E-04	1.72E-03
ILMN_1662880	FIS	2.82E-01	1.11E-05	1.38E-04

ILMN_2327974	CORO2A	2.82E-01	1.01E-05	1.26E-04
ILMN_1880425		2.82E-01	1.34E-04	1.15E-03
ILMN_1774547	MPRIP	2.82E-01	9.46E-04	6.24E-03
ILMN_1763852	ACACB	2.82E-01	7.29E-05	6.84E-04
ILMN_1795949	CORO7	2.82E-01	1.99E-03	1.18E-02
ILMN_1698478	SNAPC2	2.82E-01	3.13E-03	1.74E-02
ILMN_2066348	HERPUD2	2.82E-01	2.86E-03	1.61E-02
ILMN_3209180	LOC645094	2.82E-01	4.02E-04	3.00E-03
ILMN_1700628	DDX24	2.82E-01	7.29E-03	3.57E-02
ILMN_1904980		2.81E-01	2.24E-03	1.31E-02
ILMN_1699854	GRIN3B	2.81E-01	1.87E-03	1.12E-02
ILMN_3244987	KIAA0895L	2.81E-01	1.60E-03	9.74E-03
ILMN_1727790	KHDRBS3	2.81E-01	1.72E-03	1.04E-02
ILMN_2410262	MTMR14	2.81E-01	9.00E-06	1.15E-04
ILMN_1670666	RAB12	2.81E-01	3.08E-04	2.38E-03
ILMN_3284114	LOC399748	2.81E-01	7.13E-04	4.90E-03
ILMN_1796710	PHC1	2.81E-01	9.89E-04	6.48E-03
ILMN_1787931	LOC389517	2.81E-01	9.72E-06	1.23E-04
ILMN_2347044	SLC25A14	2.81E-01	2.82E-05	3.04E-04
ILMN_1758293	UBR7	2.81E-01	5.04E-06	7.08E-05
ILMN_1669215	SRPK2	2.81E-01	3.61E-03	1.96E-02
ILMN_1691430	GSTCD	2.81E-01	1.35E-04	1.16E-03
ILMN_1680279	USP49	2.81E-01	4.67E-03	2.45E-02
ILMN_1779428	LOC387856	2.81E-01	4.16E-06	6.02E-05
ILMN_2201347	ZDHHC3	2.80E-01	5.39E-05	5.27E-04
ILMN_1765159	ELMOD2	2.80E-01	6.81E-05	6.46E-04
ILMN_1751425	ERMP1	2.80E-01	1.56E-05	1.83E-04
ILMN_1733562	TFB1M	2.80E-01	3.79E-04	2.85E-03
ILMN_1674580	TRIM36	2.80E-01	7.72E-05	7.19E-04
ILMN_1706413	C1orf66	2.80E-01	1.34E-05	1.61E-04
ILMN_1744240	WDR67	2.80E-01	6.09E-04	4.29E-03
ILMN_2373010	TMEM70	2.79E-01	2.41E-03	1.39E-02
ILMN_2400644	SRGAP3	2.79E-01	1.03E-02	4.75E-02
ILMN_1700831	SLC27A2	2.79E-01	4.12E-05	4.19E-04
ILMN_2155480	RAB43	2.79E-01	5.36E-06	7.44E-05
ILMN_2160764	HBP1	2.79E-01	6.48E-03	3.22E-02
ILMN_2147105	LOC440348	2.79E-01	5.37E-04	3.85E-03
ILMN_3245672	SNORD84	2.79E-01	6.02E-03	3.03E-02
ILMN_1778168	ELMO2	2.79E-01	3.06E-05	3.26E-04
ILMN_1722798	PLCD3	2.79E-01	1.40E-06	2.35E-05
ILMN_1711166	WDR8	2.79E-01	3.79E-04	2.85E-03
ILMN_1700923	ATG4C	2.78E-01	2.19E-04	1.76E-03
ILMN_1795104	ACADS	2.78E-01	2.10E-07	4.90E-06
ILMN_1875123		2.78E-01	1.87E-03	1.12E-02
ILMN_1751598	SESN2	2.78E-01	3.05E-06	4.61E-05
ILMN_1772261	GLG1	2.78E-01	4.32E-04	3.19E-03

ILMN_1794875	AGPAT9	2.78E-01	8.12E-03	3.90E-02
ILMN_1740707	LOC646123	2.78E-01	8.13E-06	1.06E-04
ILMN_1685115	HEXIM1	2.78E-01	9.93E-03	4.62E-02
ILMN_1679640	FXR1	2.77E-01	2.30E-04	1.85E-03
ILMN_1695246	KLHDC8B	2.77E-01	4.57E-05	4.57E-04
ILMN_1781207	FYN	2.77E-01	1.87E-06	3.02E-05
ILMN_2327795	RERE	2.77E-01	1.01E-03	6.59E-03
ILMN_2174612	CNOT8	2.77E-01	1.64E-04	1.38E-03
ILMN_1689123	CCNK	2.77E-01	1.58E-04	1.33E-03
ILMN_1701696	LOC644863	2.77E-01	7.59E-05	7.09E-04
ILMN_1814165	SSBP3	2.77E-01	2.63E-04	2.08E-03
ILMN_1742400	CEP350	2.77E-01	7.31E-06	9.65E-05
ILMN_1728983	NR2C1	2.77E-01	1.36E-04	1.17E-03
ILMN_1657864	TMUB2	2.77E-01	1.33E-05	1.60E-04
ILMN_1729980	RNF216	2.77E-01	1.43E-05	1.70E-04
ILMN_2093748	ZNF669	2.77E-01	2.97E-04	2.31E-03
ILMN_1680037	FAM65A	2.77E-01	8.44E-06	1.09E-04
ILMN_2339748	RNF13	2.77E-01	2.08E-05	2.35E-04
ILMN_1786021	PRKAB2	2.76E-01	2.43E-04	1.94E-03
ILMN_1739798	C7orf30	2.76E-01	6.60E-05	6.28E-04
ILMN_3187771	C14orf167	2.76E-01	8.63E-06	1.11E-04
ILMN_1688772	C8orf51	2.76E-01	6.78E-04	4.69E-03
ILMN_3237617	RNU5A	2.76E-01	3.36E-04	2.57E-03
ILMN_1699112	COPB1	2.76E-01	1.60E-04	1.35E-03
ILMN_1734608	ZNF77	2.76E-01	2.76E-05	2.99E-04
ILMN_1681269	DYRK4	2.76E-01	3.37E-05	3.53E-04
ILMN_1865735		2.76E-01	6.95E-05	6.58E-04
ILMN_2041161	DENND4A	2.76E-01	9.01E-04	5.99E-03
ILMN_1669696	ZNF792	2.76E-01	1.86E-05	2.14E-04
ILMN_1722102	ANAPC11	2.76E-01	2.16E-04	1.75E-03
ILMN_1702168	HSD17B12	2.76E-01	1.54E-03	9.42E-03
ILMN_1771987	SLC44A2	2.75E-01	5.58E-07	1.10E-05
ILMN_1740083	ORC4L	2.75E-01	9.30E-05	8.42E-04
ILMN_2084353	M6PR	2.75E-01	6.33E-05	6.06E-04
ILMN_2292646	GAD1	2.75E-01	3.42E-06	5.10E-05
ILMN_1872564		2.75E-01	7.30E-06	9.65E-05
ILMN_1668228	LOC136143	2.75E-01	1.01E-03	6.60E-03
ILMN_1651506	NCOA6IP	2.75E-01	9.21E-08	2.48E-06
ILMN_3251379	SLC35E1	2.75E-01	8.21E-06	1.07E-04
ILMN_3275106	LOC100131866	2.75E-01	7.33E-05	6.87E-04
ILMN_1671583	MKRN1	2.75E-01	2.36E-04	1.89E-03
ILMN_2399877	COG5	2.75E-01	7.37E-04	5.04E-03
ILMN_3250850	RFESD	2.74E-01	4.45E-03	2.35E-02
ILMN_1771482	KIAA1324	2.74E-01	1.72E-03	1.04E-02
ILMN_1851492		2.74E-01	7.17E-05	6.76E-04
ILMN_3240117	AIDA	2.74E-01	1.13E-04	9.91E-04

ILMN_1700378	ZWILCH	2.74E-01	9.97E-04	6.52E-03
ILMN_2112493	DAP	2.74E-01	9.17E-06	1.17E-04
ILMN_1720235	ADSSL1	2.74E-01	8.18E-04	5.50E-03
ILMN_1653771	WDR63	2.74E-01	3.75E-03	2.03E-02
ILMN_2092850	HPSE	2.74E-01	3.77E-04	2.83E-03
ILMN_1696711	C6orf148	2.74E-01	5.95E-05	5.74E-04
ILMN_2073543	C15orf63	2.74E-01	4.62E-03	2.42E-02
ILMN_3241834	LOC100134504	2.73E-01	5.47E-03	2.80E-02
ILMN_1796411	TBCCD1	2.73E-01	3.98E-05	4.06E-04
ILMN_2349610	DPH3	2.73E-01	5.34E-05	5.23E-04
ILMN_1656477	ARSA	2.73E-01	1.61E-05	1.90E-04
ILMN_1756393	LOC90113	2.73E-01	2.37E-04	1.90E-03
ILMN_1684306	S100A4	2.73E-01	1.70E-04	1.42E-03
ILMN_1690999	MED23	2.73E-01	1.50E-03	9.19E-03
ILMN_1722820	KDELR3	2.73E-01	5.67E-07	1.11E-05
ILMN_1782377	LOC440354	2.73E-01	3.79E-03	2.04E-02
ILMN_1726678	HSPC047	2.73E-01	5.97E-08	1.72E-06
ILMN_1674038	CTSD	2.73E-01	3.28E-04	2.52E-03
ILMN_2055310	MBD4	2.72E-01	7.41E-03	3.61E-02
ILMN_2124757	RPL23AP13	2.72E-01	9.79E-04	6.42E-03
ILMN_1718023	APEH	2.72E-01	5.67E-04	4.02E-03
ILMN_1696003	GNAI3	2.72E-01	1.07E-03	6.92E-03
ILMN_1729487	GMPR	2.72E-01	4.88E-05	4.84E-04
ILMN_1739943	SBNO1	2.72E-01	1.81E-03	1.08E-02
ILMN_1703180	ETV3	2.72E-01	1.10E-05	1.37E-04
ILMN_1751615	COQ10B	2.72E-01	5.56E-04	3.96E-03
ILMN_3264073	LOC100130070	2.72E-01	1.72E-04	1.43E-03
ILMN_1665384	SH3BP5L	2.72E-01	3.06E-04	2.37E-03
ILMN_1798705	CCNC	2.72E-01	1.36E-04	1.17E-03
ILMN_1695468	SRPK2	2.71E-01	2.33E-05	2.58E-04
ILMN_2379326	MAP3K7	2.71E-01	7.21E-05	6.78E-04
ILMN_1703111	BBS7	2.71E-01	1.28E-03	8.07E-03
ILMN_1668924	BEGAIN	2.71E-01	1.27E-04	1.10E-03
ILMN_3216979	LOC646949	2.71E-01	1.74E-05	2.02E-04
ILMN_1660794	LBH	2.70E-01	1.09E-03	7.03E-03
ILMN_2093389	SNAPC1	2.70E-01	3.75E-03	2.03E-02
ILMN_1684391	PLOD1	2.70E-01	2.73E-03	1.55E-02
ILMN_1811754	NDUFB10	2.70E-01	6.17E-06	8.38E-05
ILMN_1810604	ELMOD1	2.70E-01	5.16E-06	7.21E-05
ILMN_1745343	ZMAT2	2.70E-01	4.22E-07	8.66E-06
ILMN_1847308		2.70E-01	7.83E-05	7.27E-04
ILMN_1774390	LOC441054	2.70E-01	7.11E-04	4.89E-03
ILMN_2358784	ASB3	2.70E-01	5.48E-06	7.58E-05
ILMN_1726496	SEL1L	2.69E-01	1.73E-05	2.01E-04
ILMN_2182704	BIRC2	2.69E-01	1.05E-02	4.84E-02
ILMN_3267451	GAPDHL6	2.69E-01	4.84E-04	3.51E-03

ILMN_3199655	LOC646784	2.69E-01	3.29E-03	1.82E-02
ILMN_2136635	ISCA2	2.69E-01	4.68E-03	2.45E-02
ILMN_1735275	WDSUB1	2.69E-01	2.69E-04	2.12E-03
ILMN_2332267	CSNK1G3	2.69E-01	6.74E-04	4.67E-03
ILMN_1764596	MPST	2.69E-01	1.81E-05	2.08E-04
ILMN_1739161	PPAP2A	2.68E-01	1.38E-03	8.61E-03
ILMN_1756352	MAPBPIP	2.68E-01	5.48E-03	2.80E-02
ILMN_1712523	MAP6	2.68E-01	9.44E-03	4.44E-02
ILMN_1754969	LMCD1	2.68E-01	5.56E-04	3.96E-03
ILMN_1737949	GALNS	2.68E-01	7.09E-05	6.69E-04
ILMN_1727798	MOSPD1	2.68E-01	1.10E-04	9.72E-04
ILMN_2186108	DGCR6	2.68E-01	2.58E-04	2.04E-03
ILMN_1803673	LOC113230	2.68E-01	1.79E-06	2.91E-05
ILMN_2404625	LAT	2.68E-01	6.85E-03	3.38E-02
ILMN_1816342		2.68E-01	1.21E-03	7.69E-03
ILMN_1788315	SIN3B	2.67E-01	3.98E-06	5.81E-05
ILMN_1723522	APOLD1	2.67E-01	7.96E-04	5.37E-03
ILMN_1740351	KIAA0174	2.67E-01	1.21E-04	1.06E-03
ILMN_1690993	NEUROG2	2.67E-01	2.14E-03	1.25E-02
ILMN_2405233	FAM133B	2.66E-01	4.22E-04	3.12E-03
ILMN_3187425	LOC100127975	2.66E-01	6.87E-03	3.39E-02
ILMN_2161832	VPS37A	2.66E-01	1.52E-03	9.32E-03
ILMN_1757370	SMPD1	2.66E-01	8.11E-05	7.49E-04
ILMN_1811489	OXSR1	2.66E-01	3.38E-04	2.58E-03
ILMN_1753064	TTC13	2.66E-01	9.49E-03	4.45E-02
ILMN_1655930	ELL2	2.66E-01	2.85E-04	2.23E-03
ILMN_2152178	MTMR15	2.66E-01	3.38E-06	5.05E-05
ILMN_1689251	SPG3A	2.66E-01	1.14E-03	7.30E-03
ILMN_1678292	PEX16	2.66E-01	8.33E-03	3.99E-02
ILMN_3237404	LOC100132585	2.65E-01	9.92E-03	4.62E-02
ILMN_1653385	KIF3A	2.65E-01	9.45E-04	6.24E-03
ILMN_1666178	TP53I13	2.65E-01	2.51E-03	1.44E-02
ILMN_1690114	PTPLAD2	2.65E-01	3.85E-03	2.07E-02
ILMN_1731484	RHOT1	2.65E-01	4.18E-03	2.23E-02
ILMN_1657139	ADAT1	2.64E-01	2.42E-04	1.93E-03
ILMN_3244607	FAM117B	2.64E-01	6.06E-04	4.26E-03
ILMN_1825249		2.64E-01	5.84E-04	4.13E-03
ILMN_1819783		2.64E-01	1.15E-05	1.42E-04
ILMN_3240236	SMCR5	2.64E-01	1.19E-04	1.04E-03
ILMN_2367384	EPHB2	2.64E-01	7.05E-03	3.46E-02
ILMN_2409298	NUSAP1	2.64E-01	1.12E-04	9.86E-04
ILMN_2315964	PSRC1	2.64E-01	7.49E-08	2.07E-06
ILMN_1683992	OXSM	2.64E-01	1.59E-06	2.63E-05
ILMN_1734991	PPM1B	2.64E-01	1.95E-04	1.59E-03
ILMN_2075334	HIST1H4C	2.63E-01	1.85E-03	1.11E-02
ILMN_1757781	SAP30L	2.63E-01	1.70E-04	1.42E-03

ILMN_1758487	CNPY4	2.63E-01	6.21E-06	8.42E-05
ILMN_2216265	LONP2	2.63E-01	1.42E-05	1.69E-04
ILMN_3258594	LOC100128168	2.63E-01	4.01E-05	4.09E-04
ILMN_1784523	ATP6V1G1	2.63E-01	6.28E-04	4.40E-03
ILMN_1712532	CARD9	2.63E-01	4.51E-05	4.52E-04
ILMN_2119692	CSAD	2.63E-01	1.07E-05	1.33E-04
ILMN_1699217	C1orf96	2.63E-01	1.93E-04	1.58E-03
ILMN_1696702	NEO1	2.62E-01	9.98E-05	8.94E-04
ILMN_1718646	MMP15	2.62E-01	2.56E-06	3.94E-05
ILMN_1742922	PRIM2A	2.62E-01	1.23E-05	1.50E-04
ILMN_1677534	SCAP	2.62E-01	2.40E-05	2.65E-04
ILMN_1692790	ITGB3BP	2.62E-01	4.03E-03	2.16E-02
ILMN_1751793	PCNXL2	2.62E-01	5.17E-06	7.22E-05
ILMN_2365479	SNX1	2.62E-01	7.27E-05	6.83E-04
ILMN_1794599	SNRPD3	2.62E-01	2.53E-03	1.45E-02
ILMN_1784783	NME5	2.62E-01	4.94E-03	2.57E-02
ILMN_1709948	LOC651453	2.62E-01	8.71E-04	5.82E-03
ILMN_2334242	CREB1	2.62E-01	4.88E-03	2.54E-02
ILMN_1761922	LOC647037	2.62E-01	6.48E-03	3.22E-02
ILMN_1675055	C1orf166	2.62E-01	3.59E-06	5.32E-05
ILMN_3248848	LOC100134407	2.62E-01	4.60E-04	3.36E-03
ILMN_2365484	SNX1	2.61E-01	1.09E-04	9.64E-04
ILMN_3245228	SNORA20	2.61E-01	1.34E-04	1.16E-03
ILMN_1693072	ELAC1	2.61E-01	2.31E-04	1.85E-03
ILMN_1709439	CHMP1A	2.61E-01	8.37E-04	5.61E-03
ILMN_3246678	NPW	2.61E-01	2.26E-03	1.32E-02
ILMN_3249546	RASA4P	2.61E-01	1.39E-03	8.65E-03
ILMN_1737163	SH3BGRL3	2.61E-01	5.61E-03	2.86E-02
ILMN_1755926	DBI	2.61E-01	4.13E-03	2.21E-02
ILMN_1813568	TRPV1	2.61E-01	1.79E-05	2.06E-04
ILMN_1670517	SULT1A3	2.61E-01	4.86E-05	4.82E-04
ILMN_2337789	MARCH2	2.61E-01	6.37E-05	6.09E-04
ILMN_2175712	NDUFA11	2.61E-01	3.06E-03	1.70E-02
ILMN_1735155	GLB1	2.61E-01	4.41E-05	4.44E-04
ILMN_1713285	NAPA	2.61E-01	8.59E-05	7.87E-04
ILMN_2087702	MYH9	2.61E-01	3.85E-04	2.88E-03
ILMN_1698846	SLC8A2	2.60E-01	9.52E-06	1.21E-04
ILMN_2373779	COPS8	2.60E-01	3.57E-04	2.71E-03
ILMN_1711102	B3GNT2	2.60E-01	1.08E-04	9.58E-04
ILMN_1656718	DEF8	2.60E-01	1.05E-02	4.84E-02
ILMN_1790917	HOM-TES-103	2.60E-01	2.88E-05	3.10E-04
ILMN_1748116	GEMIN8	2.60E-01	1.40E-05	1.67E-04
ILMN_1812640	AHR	2.60E-01	1.05E-02	4.86E-02
ILMN_1750596	CLUAP1	2.60E-01	6.29E-04	4.40E-03
ILMN_1721712	SYNGR1	2.60E-01	5.85E-06	8.01E-05
ILMN_1710738	RC3H2	2.59E-01	1.42E-03	8.77E-03

ILMN_1801130	STOML1	2.59E-01	6.00E-03	3.02E-02
ILMN_2384785	CCNE1	2.59E-01	6.80E-06	9.10E-05
ILMN_3202315	LOC643779	2.59E-01	3.04E-04	2.36E-03
ILMN_2376458	CSF2RA	2.59E-01	2.30E-04	1.85E-03
ILMN_1703558	FHL3	2.59E-01	2.14E-03	1.26E-02
ILMN_1775473	LOC400652	2.59E-01	4.50E-04	3.30E-03
ILMN_1724718	NCK2	2.59E-01	3.20E-04	2.46E-03
ILMN_1743583	MADD	2.59E-01	1.21E-04	1.06E-03
ILMN_1676731	C17orf65	2.59E-01	4.04E-04	3.01E-03
ILMN_1739641	MTMR3	2.59E-01	6.19E-05	5.94E-04
ILMN_1683969	FKBP1A	2.59E-01	1.00E-04	8.99E-04
ILMN_1674394	C20orf3	2.59E-01	6.10E-04	4.29E-03
ILMN_2210129	PRIM1	2.58E-01	3.62E-03	1.97E-02
ILMN_1813893	DDX43	2.58E-01	9.74E-03	4.55E-02
ILMN_1804329	TUSC2	2.58E-01	2.23E-06	3.50E-05
ILMN_1692276	GGPS1	2.58E-01	1.62E-05	1.90E-04
ILMN_1737586	LOC653994	2.58E-01	5.26E-03	2.71E-02
ILMN_3296002	LOC440461	2.57E-01	2.02E-04	1.65E-03
ILMN_1749789	HIST1H1D	2.57E-01	3.82E-03	2.06E-02
ILMN_1728747	STXBP1	2.57E-01	2.06E-07	4.82E-06
ILMN_1732226	DHX57	2.57E-01	2.92E-05	3.14E-04
ILMN_1661424	THAP6	2.57E-01	3.64E-05	3.77E-04
ILMN_1778917	CDK7	2.57E-01	1.09E-04	9.63E-04
ILMN_3188984	C20orf199	2.57E-01	2.48E-05	2.73E-04
ILMN_2400372	SULT1A2	2.57E-01	7.79E-05	7.24E-04
ILMN_1808566	TMEM180	2.56E-01	5.13E-03	2.65E-02
ILMN_2042343	MRPL42P5	2.56E-01	4.06E-06	5.90E-05
ILMN_1736460	MDM1	2.56E-01	6.17E-03	3.09E-02
ILMN_1747183	GXYLT1	2.56E-01	3.20E-03	1.77E-02
ILMN_2233050	PLA2G2D	2.56E-01	2.81E-03	1.59E-02
ILMN_1726041	FLJ30092	2.56E-01	2.32E-05	2.57E-04
ILMN_1780598	PIAS1	2.55E-01	1.42E-04	1.22E-03
ILMN_1690807	MKL2	2.55E-01	3.44E-03	1.89E-02
ILMN_2233366	ASAP1	2.55E-01	3.72E-04	2.80E-03
ILMN_2176931	PELI3	2.55E-01	1.30E-05	1.57E-04
ILMN_1763365	FAM103A1	2.55E-01	1.37E-03	8.52E-03
ILMN_1790008	CYP2U1	2.55E-01	1.69E-04	1.41E-03
ILMN_2374770	TAX1BP1	2.55E-01	2.10E-03	1.24E-02
ILMN_1717261	HLA-DRB3	2.55E-01	1.61E-04	1.35E-03
ILMN_1687351	ANKRA2	2.55E-01	2.37E-03	1.37E-02
ILMN_1808374	SNTB2	2.55E-01	8.54E-04	5.71E-03
ILMN_1882764	LOC730358	2.55E-01	4.54E-04	3.32E-03
ILMN_1733781	FSD1	2.54E-01	4.14E-06	6.00E-05
ILMN_1711270	SFRS14	2.54E-01	4.23E-04	3.13E-03
ILMN_1779034	NADSYN1	2.54E-01	6.49E-06	8.73E-05
ILMN_2090040	KIAA0495	2.54E-01	1.80E-04	1.49E-03

ILMN_2306540	PDE9A	2.54E-01	1.20E-04	1.05E-03
ILMN_2415748	WSB1	2.54E-01	5.16E-04	3.71E-03
ILMN_1694671	ZFAND2A	2.54E-01	2.09E-03	1.23E-02
ILMN_2339377	DNM2	2.54E-01	8.39E-05	7.70E-04
ILMN_1794470	ANKFY1	2.54E-01	3.49E-04	2.65E-03
ILMN_1677484	SNAPC4	2.54E-01	2.25E-05	2.51E-04
ILMN_1739384	USP35	2.54E-01	3.32E-04	2.55E-03
ILMN_1751171	IRF2BP1	2.54E-01	6.39E-03	3.18E-02
ILMN_1664153	SLC30A5	2.54E-01	7.31E-03	3.57E-02
ILMN_2321634	RAD17	2.54E-01	2.64E-03	1.51E-02
ILMN_1673640	PAG1	2.54E-01	7.47E-03	3.64E-02
ILMN_2393712	CTTN	2.54E-01	4.07E-04	3.03E-03
ILMN_1685978	ATPIF1	2.53E-01	6.20E-03	3.11E-02
ILMN_1722872	MYH9	2.53E-01	1.09E-03	7.06E-03
ILMN_1723895	GTF3C5	2.53E-01	4.55E-04	3.33E-03
ILMN_1707173	HIST3H3	2.53E-01	4.30E-04	3.18E-03
ILMN_1815238	MAP2K3	2.53E-01	3.34E-05	3.51E-04
ILMN_1723156	PTPRA	2.53E-01	6.69E-04	4.64E-03
ILMN_1654563	EFNB1	2.53E-01	8.19E-05	7.54E-04
ILMN_1812445	PRPSAP2	2.53E-01	3.13E-03	1.74E-02
ILMN_1762508	C9orf72	2.52E-01	1.98E-05	2.26E-04
ILMN_1687403	MRPL40	2.52E-01	1.44E-03	8.89E-03
ILMN_1707475	UBE2E2	2.52E-01	7.45E-04	5.07E-03
ILMN_1679912	DPH3	2.52E-01	3.66E-05	3.78E-04
ILMN_1740920	ACADSB	2.52E-01	2.38E-03	1.38E-02
ILMN_2191436	POLA1	2.52E-01	6.76E-05	6.42E-04
ILMN_2058251	VIM	2.52E-01	7.42E-04	5.06E-03
ILMN_1711023	CDK5RAP1	2.52E-01	1.68E-04	1.40E-03
ILMN_1784516	TTC18	2.52E-01	3.45E-08	1.11E-06
ILMN_3229570	LOC729500	2.51E-01	3.52E-03	1.92E-02
ILMN_1769388	GJB2	2.51E-01	3.42E-05	3.58E-04
ILMN_1690179	CRYM	2.51E-01	4.87E-04	3.53E-03
ILMN_2168952	DENR	2.51E-01	8.83E-04	5.89E-03
ILMN_2396948	PSMC3IP	2.51E-01	3.49E-04	2.65E-03
ILMN_1787951	RABL5	2.51E-01	9.04E-04	6.01E-03
ILMN_2305116	CTH	2.51E-01	1.24E-04	1.08E-03
ILMN_1703720	SF3B14	2.51E-01	3.78E-04	2.84E-03
ILMN_3187357	LOC100130746	2.51E-01	1.31E-03	8.21E-03
ILMN_1689474	PMS2L1	2.51E-01	7.30E-04	5.00E-03
ILMN_1677910	LOC196549	2.50E-01	4.76E-05	4.73E-04
ILMN_2342068	ERC1	2.50E-01	4.14E-05	4.21E-04
ILMN_1820244		2.50E-01	4.86E-05	4.82E-04
ILMN_3243714	LOC642073	2.50E-01	3.48E-05	3.63E-04
ILMN_2299045	BMP2K	2.50E-01	9.49E-05	8.57E-04
ILMN_1778951	C6orf203	2.50E-01	1.07E-03	6.91E-03
ILMN_1661255	LOC650526	2.50E-01	3.44E-05	3.60E-04

ILMN_1709204	CLEC4A	2.50E-01	5.13E-05	5.05E-04
ILMN_1687279	DHPS	2.50E-01	4.34E-04	3.20E-03
ILMN_1712312	RAB11A	2.50E-01	3.98E-03	2.13E-02
ILMN_1697503	DHX29	2.49E-01	1.06E-03	6.87E-03
ILMN_1687508	ALDH7A1	2.49E-01	1.01E-03	6.60E-03
ILMN_1656537	SNRPN	2.49E-01	1.55E-04	1.31E-03
ILMN_1890614		2.49E-01	6.51E-04	4.53E-03
ILMN_1745217	FLJ10081	2.49E-01	1.25E-03	7.87E-03
ILMN_2305721	POMT1	2.49E-01	1.16E-04	1.02E-03
ILMN_3242362	LOC92973	2.49E-01	4.07E-05	4.14E-04
ILMN_2054554	DTWD2	2.49E-01	2.53E-03	1.45E-02
ILMN_1692191	GNA12	2.48E-01	3.07E-05	3.27E-04
ILMN_2147345	C14orf45	2.48E-01	3.18E-05	3.37E-04
ILMN_1801156	RLF	2.48E-01	2.69E-05	2.92E-04
ILMN_1713829	PTGES	2.48E-01	5.73E-03	2.91E-02
ILMN_1779258	LOC644774	2.48E-01	3.83E-03	2.06E-02
ILMN_1652913	EZH2	2.48E-01	5.74E-04	4.07E-03
ILMN_1804051	SNX8	2.48E-01	4.31E-04	3.18E-03
ILMN_2346137	ZNF557	2.48E-01	6.26E-03	3.13E-02
ILMN_1755990	ATP6AP1L	2.48E-01	3.49E-04	2.65E-03
ILMN_1696028	ETNK1	2.48E-01	2.92E-03	1.64E-02
ILMN_3306215	LOC729926	2.48E-01	1.47E-03	9.06E-03
ILMN_3287309	LOC390578	2.47E-01	1.91E-05	2.18E-04
ILMN_1740819	STARD7	2.47E-01	4.83E-04	3.51E-03
ILMN_1721657	RSU1	2.47E-01	3.23E-05	3.42E-04
ILMN_1910550		2.47E-01	1.67E-03	1.01E-02
ILMN_3277850	LOC646942	2.47E-01	3.20E-05	3.39E-04
ILMN_2396444	CD14	2.47E-01	2.26E-05	2.53E-04
ILMN_2271894	ZNF654	2.47E-01	6.99E-04	4.82E-03
ILMN_1760089	LOC645018	2.47E-01	9.67E-04	6.35E-03
ILMN_1665319	NRTN	2.47E-01	6.44E-07	1.23E-05
ILMN_3248764	KIAA1908	2.46E-01	6.31E-06	8.54E-05
ILMN_1880052		2.46E-01	3.26E-04	2.51E-03
ILMN_2306955	ACPL2	2.46E-01	8.27E-03	3.96E-02
ILMN_1755364	RALA	2.46E-01	2.11E-04	1.71E-03
ILMN_1709101	AKAP13	2.46E-01	7.07E-03	3.47E-02
ILMN_1730698	ODF2	2.46E-01	5.31E-03	2.73E-02
ILMN_2085441	FOXD4L1	2.46E-01	4.15E-04	3.09E-03
ILMN_1797277	KIF3C	2.46E-01	6.00E-06	8.19E-05
ILMN_2185665	PGAP1	2.46E-01	8.81E-06	1.13E-04
ILMN_1774028	MTFR1	2.46E-01	1.65E-03	1.00E-02
ILMN_1700047	ALAS1	2.46E-01	9.94E-04	6.51E-03
ILMN_2186877	FLJ10213	2.46E-01	6.34E-05	6.07E-04
ILMN_1745826	KATNAL2	2.46E-01	2.98E-03	1.67E-02
ILMN_1679268	PELI1	2.46E-01	7.41E-05	6.93E-04
ILMN_2307598	SLC37A3	2.46E-01	1.50E-04	1.27E-03

ILMN_3236713	SNHG1	2.45E-01	4.51E-04	3.31E-03
ILMN_1674411	CKAP2	2.45E-01	5.63E-05	5.48E-04
ILMN_1679837	SGPP1	2.45E-01	2.23E-03	1.30E-02
ILMN_2408572	RNASE4	2.45E-01	1.50E-03	9.22E-03
ILMN_1683133	KLF15	2.45E-01	2.17E-05	2.43E-04
ILMN_1699362	IK	2.45E-01	6.00E-03	3.02E-02
ILMN_1773080	OAZ1	2.45E-01	4.71E-04	3.43E-03
ILMN_1904238		2.45E-01	2.71E-03	1.54E-02
ILMN_2400297	MAPK9	2.45E-01	2.34E-03	1.36E-02
ILMN_1772998	LOC647436	2.44E-01	9.48E-04	6.26E-03
ILMN_1811823	MED25	2.44E-01	1.90E-04	1.56E-03
ILMN_1716265	PGM2L1	2.44E-01	1.59E-03	9.68E-03
ILMN_1846517		2.44E-01	9.69E-03	4.53E-02
ILMN_1741711	DOPEY2	2.44E-01	9.51E-07	1.71E-05
ILMN_1717154	AQR	2.44E-01	2.96E-03	1.66E-02
ILMN_1758034	ETFDH	2.44E-01	7.34E-03	3.58E-02
ILMN_1813517	DISP2	2.44E-01	3.56E-06	5.27E-05
ILMN_1793371	KIAA0430	2.44E-01	1.14E-04	1.00E-03
ILMN_3231952	ARL17B	2.44E-01	5.63E-04	4.00E-03
ILMN_1738699	FXR2	2.44E-01	2.61E-03	1.49E-02
ILMN_1757742	ZRANB1	2.44E-01	8.38E-03	4.00E-02
ILMN_1653797	C6orf62	2.44E-01	5.65E-04	4.01E-03
ILMN_1839481		2.44E-01	4.33E-03	2.29E-02
ILMN_1779633	PSMD6	2.43E-01	1.05E-04	9.36E-04
ILMN_1765091	ANKHD1	2.43E-01	3.08E-03	1.71E-02
ILMN_3175415	LOC100128585	2.43E-01	1.03E-04	9.22E-04
ILMN_2134765	C6orf26	2.43E-01	1.72E-03	1.04E-02
ILMN_1693905	HAT1	2.43E-01	1.93E-03	1.15E-02
ILMN_3302456	LOC730029	2.43E-01	2.33E-03	1.35E-02
ILMN_2084059	SLC12A4	2.43E-01	4.74E-06	6.70E-05
ILMN_1727524	ADAM9	2.43E-01	1.68E-05	1.96E-04
ILMN_1702177	GLO1	2.43E-01	3.17E-04	2.45E-03
ILMN_1742074	C8orf76	2.43E-01	6.65E-05	6.33E-04
ILMN_1694057	EIF3K	2.43E-01	1.23E-05	1.50E-04
ILMN_1754130	TRIM52	2.43E-01	6.99E-05	6.61E-04
ILMN_1902594		2.43E-01	1.12E-04	9.90E-04
ILMN_1901304		2.43E-01	1.47E-03	9.04E-03
ILMN_1773427	KANK1	2.43E-01	1.31E-03	8.19E-03
ILMN_1787541	SPSB2	2.42E-01	1.07E-02	4.94E-02
ILMN_1709042	RBM4	2.42E-01	8.03E-06	1.05E-04
ILMN_1668345	OAF	2.42E-01	7.44E-03	3.62E-02
ILMN_1670809	NRM	2.42E-01	4.17E-06	6.04E-05
ILMN_3302484	LOC729774	2.42E-01	1.30E-03	8.15E-03
ILMN_2142554	NENF	2.42E-01	1.06E-03	6.87E-03
ILMN_2334303	SEC24B	2.42E-01	3.78E-04	2.84E-03
ILMN_3198499	LOC654350	2.42E-01	1.05E-02	4.84E-02

ILMN_3238652	LOC100133220	2.42E-01	4.92E-04	3.56E-03
ILMN_1786118	FXYD7	2.42E-01	2.53E-03	1.45E-02
ILMN_1704753	EPAS1	2.42E-01	5.34E-03	2.74E-02
ILMN_1694502	PRIM1	2.41E-01	4.72E-06	6.69E-05
ILMN_1718946	ADAM10	2.41E-01	9.67E-04	6.36E-03
ILMN_1697682	RECQL5	2.41E-01	2.26E-04	1.82E-03
ILMN_1702114	NOMO1	2.41E-01	1.28E-04	1.11E-03
ILMN_1686261	TOM1L2	2.41E-01	7.76E-06	1.02E-04
ILMN_1737964	HIATL1	2.41E-01	8.16E-04	5.50E-03
ILMN_1732772	PPME1	2.41E-01	4.59E-04	3.36E-03
ILMN_2273261	FBXO3	2.41E-01	2.74E-03	1.55E-02
ILMN_1794726	RNF167	2.41E-01	5.59E-03	2.85E-02
ILMN_3280402	LOC100132510	2.41E-01	5.60E-04	3.98E-03
ILMN_1680111	AHDC1	2.41E-01	2.56E-05	2.80E-04
ILMN_1815519	EPN2	2.40E-01	3.72E-04	2.81E-03
ILMN_3260286	LOC100128062	2.40E-01	8.53E-03	4.07E-02
ILMN_1706598	ACPL2	2.40E-01	7.41E-03	3.61E-02
ILMN_1660186	SYF2	2.40E-01	1.47E-04	1.25E-03
ILMN_3237385	NRBF2	2.40E-01	1.04E-03	6.74E-03
ILMN_1721922	NAB2	2.40E-01	1.79E-03	1.08E-02
ILMN_1700203	KIAA1984	2.40E-01	9.80E-04	6.43E-03
ILMN_1735822	TTC30A	2.40E-01	9.59E-03	4.49E-02
ILMN_1680388	FLJ45337	2.40E-01	3.36E-04	2.57E-03
ILMN_1736327	CDC42EP3	2.39E-01	1.76E-06	2.86E-05
ILMN_1656194	TSPAN10	2.39E-01	4.26E-04	3.15E-03
ILMN_1700584	IER2	2.39E-01	2.41E-04	1.92E-03
ILMN_2380999	RECQL	2.39E-01	1.06E-02	4.88E-02
ILMN_1652906	GBGT1	2.39E-01	4.65E-06	6.62E-05
ILMN_3275489	LOC100131905	2.39E-01	4.42E-04	3.25E-03
ILMN_2415189	ATP1A1	2.39E-01	5.75E-04	4.08E-03
ILMN_2365881	ATG16L1	2.39E-01	1.29E-04	1.12E-03
ILMN_1657058	PPIL6	2.39E-01	9.05E-04	6.01E-03
ILMN_1745421	ZMAT5	2.39E-01	1.65E-04	1.38E-03
ILMN_1680580	LOC148709	2.39E-01	2.13E-04	1.73E-03
ILMN_2255579	RAB37	2.39E-01	1.38E-04	1.19E-03
ILMN_3225432	MAPKSP1	2.39E-01	1.88E-03	1.12E-02
ILMN_1765979	LOC147645	2.39E-01	2.71E-04	2.13E-03
ILMN_2216852	PGK1	2.38E-01	1.43E-05	1.70E-04
ILMN_2121408	HBEGF	2.38E-01	3.84E-05	3.95E-04
ILMN_1782685	LOC652672	2.38E-01	7.47E-03	3.64E-02
ILMN_1748438	POLR2G	2.38E-01	9.16E-06	1.17E-04
ILMN_1721204	CSF2RA	2.38E-01	1.02E-02	4.73E-02
ILMN_1663532	RIC8B	2.38E-01	1.55E-05	1.83E-04
ILMN_1769546	RIN2	2.38E-01	3.15E-06	4.75E-05
ILMN_1750689	MPP5	2.38E-01	9.60E-04	6.32E-03
ILMN_1815951	PCYOX1L	2.38E-01	5.60E-03	2.85E-02

ILMN_1720266	LOC91431	2.38E-01	1.32E-03	8.24E-03
ILMN_2177732	RANBP6	2.37E-01	1.19E-05	1.46E-04
ILMN_1770388	VPS39	2.37E-01	1.43E-04	1.22E-03
ILMN_2094416	PLGLB1	2.37E-01	1.06E-02	4.87E-02
ILMN_3226045	LOC728533	2.37E-01	2.61E-04	2.07E-03
ILMN_3235825	UBE2QP2	2.37E-01	6.58E-05	6.26E-04
ILMN_1801303	AMY2A	2.37E-01	3.24E-04	2.49E-03
ILMN_1758717	SUPT6H	2.37E-01	8.22E-05	7.57E-04
ILMN_1687821	C16orf45	2.37E-01	3.95E-03	2.12E-02
ILMN_1809957	AP2S1	2.37E-01	3.45E-05	3.61E-04
ILMN_3294126	LOC100131160	2.37E-01	5.44E-04	3.89E-03
ILMN_2361163	SSBP3	2.37E-01	1.22E-03	7.75E-03
ILMN_2408938	LRRC20	2.37E-01	2.93E-03	1.64E-02
ILMN_1712798	ZNF608	2.37E-01	1.75E-03	1.05E-02
ILMN_2088825	CENTB2	2.37E-01	6.69E-06	8.96E-05
ILMN_1747244	CCNG2	2.37E-01	5.98E-05	5.76E-04
ILMN_1656293	GOSR2	2.37E-01	1.09E-05	1.35E-04
ILMN_1717029	FLJ33590	2.37E-01	1.07E-04	9.51E-04
ILMN_1815051	API5	2.36E-01	6.69E-05	6.36E-04
ILMN_1668634	FBXW7	2.36E-01	3.33E-03	1.83E-02
ILMN_2396947	PSMC3IP	2.36E-01	1.28E-03	8.05E-03
ILMN_1749521	SLC35E3	2.36E-01	6.84E-05	6.48E-04
ILMN_1693287	POMP	2.36E-01	4.74E-03	2.48E-02
ILMN_1733991	UBL7	2.36E-01	5.75E-06	7.90E-05
ILMN_1734653	FNDC1	2.36E-01	6.15E-03	3.09E-02
ILMN_3277072	LOC728178	2.36E-01	3.62E-04	2.73E-03
ILMN_1760556	C1orf63	2.36E-01	3.36E-04	2.57E-03
ILMN_1751776	CKAP2L	2.36E-01	7.85E-03	3.80E-02
ILMN_1699049	PMS2L3	2.36E-01	1.17E-04	1.02E-03
ILMN_3275345	LOC100132291	2.36E-01	8.13E-03	3.91E-02
ILMN_2157709	C14orf19	2.36E-01	2.30E-03	1.34E-02
ILMN_3188076	LOC100128060	2.36E-01	9.16E-03	4.32E-02
ILMN_1747016	CEP55	2.36E-01	1.27E-03	8.01E-03
ILMN_1732767	PSMD9	2.35E-01	8.47E-05	7.77E-04
ILMN_3240314	SCXA	2.35E-01	2.19E-03	1.28E-02
ILMN_3235917	DCAF10	2.35E-01	3.78E-06	5.56E-05
ILMN_1754643	DGCR14	2.35E-01	1.58E-03	9.62E-03
ILMN_1676719	LOC644330	2.34E-01	1.66E-03	1.00E-02
ILMN_2075794	NLRP8	2.34E-01	8.18E-03	3.93E-02
ILMN_1655663	SC65	2.34E-01	9.10E-04	6.04E-03
ILMN_1696099	ALDH4A1	2.34E-01	6.29E-04	4.41E-03
ILMN_2361186	PHF20L1	2.34E-01	3.99E-04	2.98E-03
ILMN_3250345	LOC283788	2.34E-01	2.30E-03	1.34E-02
ILMN_3269537	LOC100130562	2.34E-01	3.36E-03	1.84E-02
ILMN_1748473	GIMAP4	2.34E-01	4.91E-03	2.55E-02
ILMN_2400512	PRKCSH	2.34E-01	4.55E-05	4.56E-04

ILMN_1788783	TRAM2	2.34E-01	9.32E-06	1.18E-04
ILMN_1755120	MAN1A2	2.34E-01	1.55E-06	2.58E-05
ILMN_1771957	MAN1B1	2.34E-01	1.70E-03	1.03E-02
ILMN_1670033	HIST1H4I	2.33E-01	7.29E-03	3.57E-02
ILMN_1654445	LOC347292	2.33E-01	2.51E-03	1.44E-02
ILMN_1701131	C2orf49	2.32E-01	1.48E-04	1.25E-03
ILMN_1668639	TBC1D10B	2.32E-01	2.44E-04	1.95E-03
ILMN_1677402	LOC387763	2.32E-01	3.62E-03	1.97E-02
ILMN_1762747	RPL15	2.32E-01	1.95E-04	1.60E-03
ILMN_2143566	SLC39A6	2.32E-01	6.76E-05	6.42E-04
ILMN_2130180	RPL13L	2.32E-01	5.47E-03	2.80E-02
ILMN_1693259	PDCD6IP	2.31E-01	4.72E-04	3.44E-03
ILMN_1674308	LOC728308	2.31E-01	9.90E-05	8.88E-04
ILMN_3288755	LOC646808	2.31E-01	4.47E-03	2.36E-02
ILMN_2413278	RPL13	2.31E-01	2.61E-06	4.02E-05
ILMN_2319952	VDR	2.31E-01	5.46E-04	3.90E-03
ILMN_1724884	SNORD22	2.31E-01	4.39E-04	3.23E-03
ILMN_1796923	LOC81691	2.31E-01	4.03E-05	4.11E-04
ILMN_2382245	KIRREL2	2.31E-01	5.45E-05	5.33E-04
ILMN_1724699	ACAD8	2.31E-01	2.11E-03	1.24E-02
ILMN_2289844	SERHL	2.31E-01	1.03E-03	6.72E-03
ILMN_2153825	MEIG1	2.31E-01	7.05E-05	6.65E-04
ILMN_1755638	TAF7L	2.31E-01	4.58E-04	3.35E-03
ILMN_3178043	BEND7	2.31E-01	8.95E-03	4.24E-02
ILMN_1755749	PGK1	2.31E-01	3.36E-04	2.57E-03
ILMN_1665832	ID1	2.31E-01	2.16E-03	1.27E-02
ILMN_2359014	TBCE	2.31E-01	3.50E-06	5.20E-05
ILMN_1745075	RPLP0	2.31E-01	9.65E-04	6.35E-03
ILMN_3180989	DSTYK	2.31E-01	2.12E-04	1.72E-03
ILMN_1756767	EIF5B	2.31E-01	8.92E-03	4.23E-02
ILMN_1806103	LOC727849	2.31E-01	1.08E-06	1.91E-05
ILMN_1793743	DIRC2	2.31E-01	9.01E-04	5.99E-03
ILMN_1700144	ITGA10	2.31E-01	5.54E-03	2.82E-02
ILMN_1671158	MRPL13	2.31E-01	8.48E-06	1.10E-04
ILMN_2195957	RHOBTB2	2.30E-01	1.99E-04	1.63E-03
ILMN_1770690	CDKL3	2.30E-01	1.09E-02	4.98E-02
ILMN_2291455	FAM102A	2.30E-01	6.40E-05	6.11E-04
ILMN_1688780	S100A4	2.30E-01	8.96E-03	4.24E-02
ILMN_1792682	MCTP2	2.30E-01	1.09E-04	9.61E-04
ILMN_1680104	SLC35C1	2.30E-01	1.12E-03	7.19E-03
ILMN_1695370	LOC645968	2.30E-01	8.66E-04	5.79E-03
ILMN_3223826	SPNS2	2.29E-01	9.91E-04	6.49E-03
ILMN_1749875	LOC728715	2.29E-01	8.57E-05	7.86E-04
ILMN_1762674	NUP43	2.29E-01	4.48E-03	2.36E-02
ILMN_1735361	MAPK11	2.29E-01	2.43E-03	1.40E-02
ILMN_1692429	PQBP1	2.29E-01	1.55E-05	1.82E-04

ILMN_1772156	ERP44	2.29E-01	2.94E-04	2.29E-03
ILMN_2328224	MADD	2.29E-01	2.64E-05	2.88E-04
ILMN_1723287	SLC6A16	2.29E-01	3.19E-04	2.46E-03
ILMN_1738103	COPE	2.29E-01	1.76E-03	1.06E-02
ILMN_1724295	LOC643007	2.29E-01	6.32E-03	3.15E-02
ILMN_1705049	TMEM67	2.29E-01	6.81E-06	9.11E-05
ILMN_1735143	C1orf38	2.29E-01	6.34E-05	6.07E-04
ILMN_2326675	NR2C1	2.29E-01	7.40E-05	6.93E-04
ILMN_1743367	FZD4	2.29E-01	6.33E-05	6.06E-04
ILMN_3249645	HSPA13	2.29E-01	6.22E-06	8.44E-05
ILMN_2406557	ALDH4A1	2.28E-01	2.51E-03	1.44E-02
ILMN_1837428		2.28E-01	8.26E-03	3.96E-02
ILMN_1786168	LOC400464	2.28E-01	2.99E-06	4.54E-05
ILMN_1657497	KIAA0319	2.28E-01	1.40E-05	1.67E-04
ILMN_1867663		2.28E-01	3.22E-03	1.78E-02
ILMN_3257564	LOC100128163	2.28E-01	2.55E-03	1.46E-02
ILMN_1662417	LRPPRC	2.28E-01	2.42E-05	2.67E-04
ILMN_3235013	LOC730288	2.28E-01	1.43E-03	8.83E-03
ILMN_1714510	LOC651302	2.28E-01	2.43E-03	1.40E-02
ILMN_1773109	BAI2	2.28E-01	5.43E-05	5.30E-04
ILMN_1753692	LOC647135	2.28E-01	2.10E-05	2.37E-04
ILMN_1704713	CSNK1G1	2.28E-01	1.58E-03	9.62E-03
ILMN_1700276	C14orf100	2.28E-01	6.12E-04	4.30E-03
ILMN_1815705	LZTFL1	2.28E-01	5.71E-03	2.90E-02
ILMN_1682336	MASTL	2.27E-01	9.25E-03	4.36E-02
ILMN_2224990	HIST1H4J	2.27E-01	2.64E-03	1.51E-02
ILMN_1731644	SETDB2	2.27E-01	1.04E-05	1.30E-04
ILMN_2195236	PGRMC2	2.27E-01	6.23E-04	4.37E-03
ILMN_1704943	GPN3	2.27E-01	1.29E-04	1.11E-03
ILMN_2131882	DPY30	2.27E-01	2.93E-04	2.29E-03
ILMN_1738987	HS2ST1	2.27E-01	2.78E-03	1.57E-02
ILMN_1810554	PDPK1	2.27E-01	2.77E-04	2.18E-03
ILMN_1772943	CXorf23	2.27E-01	1.91E-04	1.57E-03
ILMN_1695430	DDB1	2.27E-01	7.56E-04	5.14E-03
ILMN_1703622	PPIB	2.26E-01	3.61E-03	1.96E-02
ILMN_2140700	CRIPAK	2.26E-01	6.81E-03	3.37E-02
ILMN_3293685	LOC286444	2.26E-01	9.13E-04	6.06E-03
ILMN_1687863	LSS	2.26E-01	3.37E-03	1.85E-02
ILMN_1776314	CHRNA10	2.26E-01	9.12E-05	8.28E-04
ILMN_1676423	CCNC	2.26E-01	1.09E-03	7.03E-03
ILMN_1667551	GUSBL1	2.26E-01	2.83E-03	1.59E-02
ILMN_1834842		2.25E-01	5.14E-04	3.70E-03
ILMN_3307733	C17orf42	2.25E-01	6.81E-04	4.71E-03
ILMN_1673363	CD97	2.25E-01	1.06E-03	6.85E-03
ILMN_3281563	LOC648771	2.25E-01	2.24E-05	2.50E-04
ILMN_1789999	SLC30A7	2.25E-01	2.72E-03	1.54E-02

ILMN_1809147	FAM118A	2.25E-01	2.09E-04	1.70E-03
ILMN_3243686	RNF160	2.25E-01	7.05E-04	4.85E-03
ILMN_1663646	DMXL1	2.24E-01	6.92E-03	3.41E-02
ILMN_1742869	TBC1D20	2.24E-01	1.69E-03	1.02E-02
ILMN_1700477	MRPL43	2.24E-01	6.46E-05	6.16E-04
ILMN_1704383	TRIM37	2.24E-01	7.52E-04	5.12E-03
ILMN_1721029	RHEBL1	2.24E-01	3.64E-06	5.38E-05
ILMN_1750394	SLC39A6	2.24E-01	3.57E-06	5.29E-05
ILMN_3302896	LOC728820	2.24E-01	1.92E-04	1.58E-03
ILMN_2055760	KIAA1715	2.24E-01	7.71E-04	5.23E-03
ILMN_1672589	SEMA4B	2.24E-01	7.95E-05	7.37E-04
ILMN_1725707	ATG16L1	2.24E-01	8.09E-05	7.47E-04
ILMN_1757391	TMEM50B	2.24E-01	5.47E-04	3.91E-03
ILMN_2290618	SEC22C	2.24E-01	1.15E-03	7.37E-03
ILMN_1684690	HDAC11	2.24E-01	1.38E-04	1.18E-03
ILMN_1667030	HSBP1	2.23E-01	1.12E-03	7.22E-03
ILMN_3289685	LOC645452	2.23E-01	5.18E-03	2.67E-02
ILMN_3244240	LOC646476	2.23E-01	1.12E-03	7.18E-03
ILMN_1786697	TRIM9	2.23E-01	3.94E-05	4.04E-04
ILMN_1715969	SLC25A37	2.23E-01	9.53E-03	4.47E-02
ILMN_2234758	SRP14	2.23E-01	6.01E-04	4.24E-03
ILMN_1797604	CAP1	2.23E-01	3.47E-04	2.64E-03
ILMN_2402936	LOC440926	2.23E-01	5.46E-04	3.90E-03
ILMN_2084073	UCN	2.23E-01	9.84E-03	4.59E-02
ILMN_1839051		2.23E-01	1.63E-03	9.93E-03
ILMN_1703955	FBXO32	2.23E-01	1.18E-04	1.04E-03
ILMN_2330845	NSF	2.22E-01	5.01E-03	2.60E-02
ILMN_1739325	LOC284023	2.22E-01	6.00E-03	3.02E-02
ILMN_1721977	ARD1A	2.22E-01	3.60E-03	1.96E-02
ILMN_1797594	NFAT5	2.22E-01	3.19E-04	2.46E-03
ILMN_1769671	RYK	2.22E-01	4.61E-04	3.37E-03
ILMN_1687864	POP5	2.22E-01	4.16E-03	2.22E-02
ILMN_2379835	SUMO1	2.22E-01	2.63E-03	1.50E-02
ILMN_1797425	DDX55	2.22E-01	4.44E-04	3.26E-03
ILMN_1777366	TTC35	2.22E-01	7.06E-04	4.85E-03
ILMN_1732182	FBXO44	2.22E-01	7.03E-04	4.84E-03
ILMN_1786108	TUT1	2.22E-01	1.69E-03	1.02E-02
ILMN_3278627	LOC391769	2.22E-01	3.30E-03	1.82E-02
ILMN_1803977	GALNT1	2.22E-01	2.06E-03	1.21E-02
ILMN_1666706	LOC645676	2.22E-01	2.27E-03	1.32E-02
ILMN_1716687	TPM1	2.22E-01	2.90E-04	2.26E-03
ILMN_3285611	LOC388556	2.21E-01	1.86E-03	1.11E-02
ILMN_1807609	SPTBN5	2.21E-01	8.76E-06	1.13E-04
ILMN_1740938	APOE	2.21E-01	3.61E-07	7.62E-06
ILMN_1804743	USP32	2.21E-01	3.87E-05	3.98E-04
ILMN_1751452	NDFIP1	2.21E-01	6.62E-06	8.88E-05

ILMN_1799028	TSPAN5	2.21E-01	7.30E-03	3.57E-02
ILMN_1668535	JOSD1	2.21E-01	1.11E-05	1.37E-04
ILMN_1680856	MAMLD1	2.21E-01	2.74E-04	2.15E-03
ILMN_3245869	LOC440957	2.21E-01	3.12E-03	1.73E-02
ILMN_3250032	XPR1	2.21E-01	2.75E-03	1.56E-02
ILMN_3243871	LOC642076	2.20E-01	7.79E-03	3.77E-02
ILMN_1732300	POLR2C	2.20E-01	9.38E-03	4.41E-02
ILMN_1771861	LOC642250	2.20E-01	6.69E-05	6.36E-04
ILMN_1721575	VPS18	2.20E-01	1.10E-03	7.11E-03
ILMN_2081465	APLP2	2.20E-01	3.04E-03	1.70E-02
ILMN_1696843	LOC613037	2.20E-01	1.00E-02	4.66E-02
ILMN_1783231	PLEKHB1	2.20E-01	8.05E-03	3.88E-02
ILMN_2048119	TBX19	2.19E-01	7.44E-04	5.07E-03
ILMN_1678579	CPT2	2.19E-01	3.88E-05	3.99E-04
ILMN_1672596	BCAR1	2.19E-01	2.45E-03	1.41E-02
ILMN_3262348	IP6K2	2.19E-01	7.08E-03	3.47E-02
ILMN_1775074	TUBGCP2	2.19E-01	1.01E-04	9.03E-04
ILMN_1772786	KIF16B	2.19E-01	1.87E-03	1.12E-02
ILMN_1670875	PPM1D	2.19E-01	2.61E-05	2.85E-04
ILMN_1705522	SPAG1	2.19E-01	8.87E-04	5.91E-03
ILMN_2307740	CD46	2.19E-01	3.52E-03	1.92E-02
ILMN_1693270	SUSD2	2.19E-01	7.80E-04	5.28E-03
ILMN_1658071	ATP1B1	2.19E-01	4.83E-04	3.51E-03
ILMN_1686645	UTP14C	2.18E-01	4.56E-03	2.40E-02
ILMN_3236367	IFFO2	2.18E-01	1.39E-03	8.62E-03
ILMN_1726589	CD248	2.18E-01	3.25E-03	1.80E-02
ILMN_1803925	MTMR3	2.18E-01	8.25E-06	1.07E-04
ILMN_2135709	C8orf47	2.18E-01	6.93E-04	4.78E-03
ILMN_1764188	C9orf93	2.18E-01	5.12E-03	2.65E-02
ILMN_1737255	C22orf36	2.18E-01	3.70E-03	2.00E-02
ILMN_1668408	AIFM1	2.18E-01	4.71E-03	2.47E-02
ILMN_1746819	C5	2.18E-01	1.56E-05	1.83E-04
ILMN_1666933	ASH2L	2.18E-01	6.52E-05	6.21E-04
ILMN_2083334	PMS2L5	2.18E-01	6.98E-04	4.81E-03
ILMN_1791840	RALBP1	2.18E-01	1.70E-04	1.42E-03
ILMN_3294222	LOC100132673	2.18E-01	1.83E-03	1.10E-02
ILMN_1726720	NUSAP1	2.18E-01	2.13E-03	1.25E-02
ILMN_3241524	ZBTB22	2.18E-01	9.52E-04	6.27E-03
ILMN_3225649	LOC731139	2.18E-01	1.27E-03	8.00E-03
ILMN_2237211	NXPH4	2.18E-01	2.23E-03	1.30E-02
ILMN_2365248	HVCN1	2.18E-01	1.36E-03	8.47E-03
ILMN_3290385	LOC401640	2.18E-01	9.21E-04	6.10E-03
ILMN_1660633	CAMK2N2	2.17E-01	1.73E-04	1.44E-03
ILMN_1759325	C16orf91	2.17E-01	2.06E-03	1.21E-02
ILMN_1815716	LMLN	2.17E-01	2.92E-04	2.28E-03
ILMN_2192385	TTC19	2.17E-01	5.10E-03	2.64E-02

ILMN_1711566	TIMP1	2.17E-01	9.25E-05	8.38E-04
ILMN_1658835	CAV2	2.17E-01	9.55E-03	4.48E-02
ILMN_3249658	LOC100134868	2.17E-01	8.95E-03	4.24E-02
ILMN_3265143	LOC100129502	2.17E-01	1.97E-03	1.17E-02
ILMN_1699598	AP2M1	2.17E-01	1.04E-06	1.84E-05
ILMN_1783394	ATF4	2.17E-01	4.18E-03	2.22E-02
ILMN_1772540	ATMIN	2.16E-01	1.02E-06	1.81E-05
ILMN_1740429	FTL	2.16E-01	7.24E-04	4.96E-03
ILMN_1801633	C20orf52	2.16E-01	6.36E-06	8.59E-05
ILMN_1726989	C1orf86	2.16E-01	2.48E-04	1.97E-03
ILMN_1749540	RBM20	2.16E-01	3.94E-03	2.11E-02
ILMN_1731193	BCL2L12	2.16E-01	1.98E-04	1.62E-03
ILMN_1702231	C1orf54	2.16E-01	9.69E-04	6.36E-03
ILMN_1704472	EID2	2.16E-01	8.55E-03	4.08E-02
ILMN_1704557	RPS6KB1	2.16E-01	1.00E-03	6.55E-03
ILMN_3236680	LOC100134393	2.16E-01	9.57E-03	4.48E-02
ILMN_2104924	C21orf70	2.16E-01	1.76E-03	1.06E-02
ILMN_1789732	TMEM189	2.15E-01	3.41E-03	1.87E-02
ILMN_1781416	FRAT1	2.15E-01	5.04E-04	3.64E-03
ILMN_1716979	GSTM4	2.15E-01	1.21E-04	1.05E-03
ILMN_2198185	CXorf12	2.15E-01	4.86E-06	6.86E-05
ILMN_3245194	LOC100132323	2.15E-01	7.48E-04	5.09E-03
ILMN_1812787	LOC653717	2.15E-01	2.71E-03	1.54E-02
ILMN_2363027	RAD51	2.15E-01	4.29E-03	2.28E-02
ILMN_1683279	PEX6	2.15E-01	5.53E-05	5.39E-04
ILMN_1812067	RER1	2.15E-01	1.33E-03	8.29E-03
ILMN_1652412	PHKB	2.15E-01	4.41E-03	2.33E-02
ILMN_3234081	LOC100129076	2.15E-01	4.78E-05	4.75E-04
ILMN_1738010	CTNS	2.15E-01	9.30E-06	1.18E-04
ILMN_1715540	TRAIP	2.15E-01	3.05E-05	3.25E-04
ILMN_1693090	CROT	2.15E-01	2.53E-03	1.45E-02
ILMN_1669308	BRPF1	2.15E-01	2.14E-05	2.41E-04
ILMN_1716838	C21orf56	2.14E-01	8.42E-04	5.65E-03
ILMN_1658399	KLRG1	2.14E-01	4.54E-03	2.39E-02
ILMN_1793267	ETHE1	2.14E-01	3.05E-04	2.37E-03
ILMN_1772646	LOC388681	2.14E-01	3.57E-04	2.70E-03
ILMN_2378376	CYB561	2.14E-01	3.88E-04	2.91E-03
ILMN_1812353	CSPP1	2.14E-01	3.52E-05	3.67E-04
ILMN_1698330	NIT1	2.14E-01	5.20E-04	3.74E-03
ILMN_1724376	C2orf30	2.14E-01	7.33E-05	6.87E-04
ILMN_2402947	USP14	2.14E-01	1.60E-03	9.72E-03
ILMN_3248263	CCDC93	2.14E-01	4.68E-06	6.65E-05
ILMN_1694699	HIST1H2AK	2.14E-01	4.18E-03	2.23E-02
ILMN_1815719	PLCG2	2.13E-01	6.17E-03	3.09E-02
ILMN_1678922	HERC4	2.13E-01	7.60E-04	5.16E-03
ILMN_2209163	CHD6	2.13E-01	6.89E-03	3.40E-02

ILMN_2336130	SULT1A4	2.13E-01	5.59E-05	5.44E-04
ILMN_3236928	ROBLD3	2.13E-01	6.96E-03	3.42E-02
ILMN_1705397	PDK2	2.13E-01	1.41E-03	8.73E-03
ILMN_3294213	LOC401098	2.13E-01	5.70E-03	2.89E-02
ILMN_1898938		2.13E-01	1.36E-04	1.17E-03
ILMN_2352009	ACADVL	2.13E-01	9.14E-03	4.31E-02
ILMN_2287653	PLAG1	2.13E-01	4.70E-03	2.46E-02
ILMN_3246962	CYTSA	2.12E-01	1.07E-03	6.92E-03
ILMN_1662188	WNT11	2.12E-01	6.52E-04	4.54E-03
ILMN_3246242	LOC388152	2.12E-01	2.05E-04	1.66E-03
ILMN_1811121	CXorf39	2.12E-01	1.34E-03	8.37E-03
ILMN_2367258	SMOX	2.12E-01	1.87E-05	2.15E-04
ILMN_1721081	SP4	2.12E-01	9.39E-06	1.19E-04
ILMN_3238358	SCARNA22	2.11E-01	9.60E-03	4.49E-02
ILMN_1743755	LOC441150	2.11E-01	6.98E-03	3.43E-02
ILMN_1887174	KIAA0146	2.11E-01	5.36E-04	3.84E-03
ILMN_2402798	AP2M1	2.11E-01	4.51E-03	2.38E-02
ILMN_3246766	LOC100132247	2.11E-01	2.72E-03	1.54E-02
ILMN_1744968	KCNAB1	2.11E-01	2.41E-04	1.92E-03
ILMN_2374425	CCNE1	2.11E-01	1.54E-03	9.43E-03
ILMN_2358783	ASB3	2.11E-01	8.52E-04	5.71E-03
ILMN_1793433	RAB10	2.11E-01	1.06E-03	6.85E-03
ILMN_2222317	DNAJB4	2.11E-01	6.80E-04	4.70E-03
ILMN_1760339	ZNF774	2.11E-01	2.20E-04	1.77E-03
ILMN_1734010	C10orf118	2.10E-01	2.08E-03	1.22E-02
ILMN_1717046	MOBKL2B	2.10E-01	2.83E-03	1.59E-02
ILMN_1691860	SPRY1	2.10E-01	1.56E-04	1.31E-03
ILMN_1705064	NDEL1	2.10E-01	3.44E-04	2.62E-03
ILMN_1663257	ATP6V1C1	2.10E-01	3.84E-03	2.07E-02
ILMN_1740269	WNT2B	2.10E-01	2.50E-04	1.99E-03
ILMN_1810852	LAMC1	2.10E-01	4.55E-05	4.56E-04
ILMN_2133187	POL3S	2.10E-01	5.43E-04	3.88E-03
ILMN_2274923	MOCS2	2.10E-01	3.10E-03	1.72E-02
ILMN_1705224	TMEM110	2.10E-01	9.09E-05	8.26E-04
ILMN_1801077	PLIN2	2.10E-01	1.06E-02	4.87E-02
ILMN_3283090	LOC391169	2.10E-01	1.13E-03	7.23E-03
ILMN_2120917	CTR9	2.10E-01	6.13E-03	3.08E-02
ILMN_1821887		2.10E-01	4.72E-04	3.44E-03
ILMN_1709604	SNORA64	2.10E-01	6.41E-03	3.19E-02
ILMN_1700337	TROAP	2.10E-01	4.15E-03	2.21E-02
ILMN_1749212	TMEM189-UBE2V1	2.10E-01	1.79E-04	1.48E-03
ILMN_2147251	MGC10997	2.10E-01	6.11E-05	5.87E-04
ILMN_1675482	LOC732425	2.10E-01	1.49E-04	1.27E-03
ILMN_2410421	NBPF1	2.09E-01	8.31E-06	1.08E-04
ILMN_3297898	LOC729769	2.09E-01	1.53E-03	9.36E-03
ILMN_1656254	NOTCH2NL	2.09E-01	3.32E-05	3.50E-04

ILMN_1686362	CRYBA4	2.09E-01	3.03E-03	1.69E-02
ILMN_2124361	LEAP2	2.09E-01	2.19E-03	1.28E-02
ILMN_1665717	EIF2S3	2.09E-01	9.95E-04	6.51E-03
ILMN_1763328	GZF1	2.08E-01	1.20E-04	1.05E-03
ILMN_1712719	MAP7	2.08E-01	6.99E-03	3.44E-02
ILMN_2272967	TRAPPC6B	2.08E-01	3.07E-04	2.38E-03
ILMN_2126957	NOMO1	2.08E-01	9.08E-04	6.03E-03
ILMN_1767111	ANO10	2.08E-01	1.20E-06	2.08E-05
ILMN_1681728	LOC643505	2.08E-01	8.48E-04	5.68E-03
ILMN_1655225	C19orf25	2.08E-01	8.32E-04	5.59E-03
ILMN_1684321	CYB5B	2.08E-01	7.94E-03	3.83E-02
ILMN_1788384	C9orf5	2.08E-01	2.19E-05	2.45E-04
ILMN_1730355	FGFR4	2.08E-01	7.97E-04	5.38E-03
ILMN_1653251	HIST1H1B	2.07E-01	1.84E-03	1.10E-02
ILMN_2193214	MPND	2.07E-01	1.03E-03	6.71E-03
ILMN_3247452	LOC100128731	2.07E-01	1.71E-04	1.43E-03
ILMN_3241364	CALHM3	2.07E-01	3.39E-06	5.06E-05
ILMN_1713290	GLT8D1	2.07E-01	5.29E-03	2.72E-02
ILMN_1778136	ZMYND15	2.06E-01	6.08E-06	8.28E-05
ILMN_2122420	HMGCL	2.06E-01	1.71E-04	1.43E-03
ILMN_1675268	LRP4	2.06E-01	7.72E-04	5.23E-03
ILMN_1719906	HADH	2.06E-01	2.16E-04	1.75E-03
ILMN_1771652	BAIAP2L2	2.06E-01	4.58E-04	3.35E-03
ILMN_1808333	PPP1R7	2.06E-01	1.41E-03	8.76E-03
ILMN_1731137	TXNDC9	2.06E-01	5.90E-03	2.98E-02
ILMN_1683441	NCAPD3	2.06E-01	7.25E-06	9.60E-05
ILMN_1693726	TBC1D10A	2.06E-01	1.38E-03	8.59E-03
ILMN_1738047	C10orf73	2.06E-01	9.97E-04	6.52E-03
ILMN_3235188	LOC100131187	2.06E-01	4.68E-06	6.65E-05
ILMN_1784948	SPOCD1	2.05E-01	6.01E-03	3.03E-02
ILMN_1652163	DVL2	2.05E-01	1.10E-03	7.11E-03
ILMN_1810901	RNASEH2A	2.05E-01	1.12E-03	7.18E-03
ILMN_1902251		2.05E-01	9.83E-03	4.59E-02
ILMN_3289745	LOC339352	2.05E-01	3.16E-04	2.44E-03
ILMN_3251545	CHMP5	2.05E-01	9.46E-03	4.44E-02
ILMN_1761490	ZNF75D	2.04E-01	3.58E-04	2.71E-03
ILMN_2371470	C1orf124	2.04E-01	8.94E-03	4.23E-02
ILMN_1661266	HLA-DQB1	2.04E-01	8.26E-03	3.96E-02
ILMN_1663538	CLYBL	2.04E-01	3.06E-04	2.37E-03
ILMN_1761314	NFS1	2.04E-01	3.70E-04	2.79E-03
ILMN_3185161	CEP78	2.04E-01	1.51E-05	1.78E-04
ILMN_1765704	CDC2L1	2.04E-01	9.32E-03	4.38E-02
ILMN_1850238		2.03E-01	2.39E-04	1.91E-03
ILMN_1710458	LOC646990	2.03E-01	4.27E-03	2.27E-02
ILMN_1754458	SDHALP1	2.03E-01	3.81E-04	2.86E-03
ILMN_2388142	CD99L2	2.03E-01	2.25E-06	3.53E-05

ILMN_1789266	CCDC25	2.03E-01	1.66E-03	1.00E-02
ILMN_3238274	BTBD2	2.03E-01	6.52E-03	3.24E-02
ILMN_1656335	RIT1	2.03E-01	1.35E-03	8.44E-03
ILMN_1756657	TRIM7	2.03E-01	4.61E-04	3.37E-03
ILMN_3304049	LOC729500	2.03E-01	3.65E-03	1.98E-02
ILMN_3235357	LOC100133329	2.03E-01	3.68E-04	2.78E-03
ILMN_2371053	EFNA1	2.02E-01	8.29E-04	5.57E-03
ILMN_1769013	ASGR1	2.02E-01	1.50E-03	9.20E-03
ILMN_1683065	C2orf64	2.02E-01	9.67E-03	4.52E-02
ILMN_2130514	FAM76B	2.02E-01	9.66E-04	6.35E-03
ILMN_3264543	LOC100128060	2.02E-01	3.19E-05	3.38E-04
ILMN_1692199	RNF103	2.02E-01	7.43E-03	3.62E-02
ILMN_1690262	SNAI3	2.02E-01	1.65E-03	1.00E-02
ILMN_1800354	CST3	2.02E-01	1.04E-02	4.81E-02
ILMN_1713901	KDELR3	2.02E-01	2.30E-06	3.61E-05
ILMN_1690638	LOC402677	2.02E-01	3.95E-04	2.96E-03
ILMN_1681927	HDGF2	2.01E-01	3.01E-04	2.34E-03
ILMN_1659024	TMCC2	2.01E-01	8.04E-05	7.44E-04
ILMN_2077858	SIRT7	2.01E-01	5.40E-04	3.86E-03
ILMN_2387919	PRKAG2	2.01E-01	8.57E-05	7.86E-04
ILMN_1784130	LOC647054	2.01E-01	8.17E-03	3.92E-02
ILMN_1683277	KIAA0319L	2.01E-01	6.47E-04	4.51E-03
ILMN_2375992	SPINT1	2.01E-01	2.42E-05	2.67E-04
ILMN_1714335	RDH10	2.00E-01	8.19E-05	7.54E-04
ILMN_1727458	HDAC1	2.00E-01	1.30E-04	1.12E-03
ILMN_1687546	HSP90AA1	2.00E-01	6.59E-03	3.27E-02
ILMN_1697510	ACSS2	2.00E-01	9.00E-05	8.19E-04
ILMN_1808712	RNF40	2.00E-01	2.44E-03	1.40E-02
ILMN_2407824	ATP1B1	2.00E-01	1.55E-03	9.48E-03
ILMN_1688606	RNFT1	2.00E-01	9.95E-03	4.63E-02
ILMN_2225577	C5orf37	2.00E-01	7.35E-04	5.03E-03
ILMN_1693789	ALPP	2.00E-01	7.52E-03	3.66E-02
ILMN_1692742	DENND3	2.00E-01	5.06E-05	4.99E-04
ILMN_1692948	CCDC90B	1.99E-01	4.33E-03	2.29E-02
ILMN_1769394	PLCD1	1.99E-01	8.81E-04	5.88E-03
ILMN_1791679	DNER	1.99E-01	8.88E-03	4.21E-02
ILMN_1689817	LCOR	1.99E-01	2.77E-03	1.56E-02
ILMN_1727287	PHF20L1	1.99E-01	5.07E-03	2.62E-02
ILMN_1776088	NAT9	1.99E-01	5.34E-04	3.83E-03
ILMN_3290340	LOC100132032	1.99E-01	1.52E-03	9.34E-03
ILMN_2214947	LOC619207	1.99E-01	6.24E-03	3.12E-02
ILMN_1746919	LOC643882	1.99E-01	6.22E-03	3.11E-02
ILMN_2396198	APLP1	1.99E-01	3.16E-04	2.44E-03
ILMN_3236021	LOC100133923	1.99E-01	4.92E-03	2.56E-02
ILMN_1687782	RAD17	1.99E-01	7.07E-03	3.47E-02
ILMN_2273609	KIAA1543	1.98E-01	2.68E-05	2.91E-04

ILMN_1773174	LOC653650	1.98E-01	8.46E-06	1.09E-04
ILMN_1680348	NBPF3	1.98E-01	1.60E-04	1.35E-03
ILMN_1745991	GNRH1	1.98E-01	1.79E-03	1.07E-02
ILMN_2129877	PARP11	1.98E-01	1.70E-04	1.42E-03
ILMN_1709626	FAM14B	1.98E-01	3.81E-03	2.05E-02
ILMN_1769382	KBTBD3	1.98E-01	6.09E-03	3.06E-02
ILMN_1740234	GSTO2	1.98E-01	8.86E-05	8.08E-04
ILMN_2207562	C4orf16	1.98E-01	2.51E-03	1.44E-02
ILMN_1653836	C11orf41	1.98E-01	4.28E-03	2.27E-02
ILMN_1796085	PPM1B	1.98E-01	1.05E-03	6.80E-03
ILMN_1804490	PRKRIP1	1.97E-01	7.02E-03	3.45E-02
ILMN_2146566	SFRS16	1.97E-01	1.03E-03	6.70E-03
ILMN_1768488	TERF2	1.97E-01	2.75E-04	2.16E-03
ILMN_1682894	TMEM191B	1.97E-01	6.01E-03	3.03E-02
ILMN_1744023	MGC18216	1.97E-01	2.59E-04	2.05E-03
ILMN_1785424	ABLIM1	1.97E-01	2.59E-04	2.05E-03
ILMN_1728714	SSSCA1	1.97E-01	3.39E-03	1.86E-02
ILMN_1706784	H2AFV	1.97E-01	6.99E-04	4.82E-03
ILMN_3193798	ATXN1L	1.97E-01	6.46E-04	4.51E-03
ILMN_3244011	LOC729597	1.97E-01	1.41E-03	8.76E-03
ILMN_1688104	TRDMT1	1.97E-01	4.51E-03	2.37E-02
ILMN_3241118	LOC641727	1.97E-01	4.69E-03	2.45E-02
ILMN_2396020	DUSP6	1.97E-01	2.32E-03	1.35E-02
ILMN_2353642	ATP6V0B	1.97E-01	1.42E-03	8.79E-03
ILMN_1684984	MAGED2	1.96E-01	1.29E-04	1.11E-03
ILMN_1783702	MORC3	1.96E-01	4.19E-04	3.11E-03
ILMN_2383807	CALB2	1.96E-01	8.45E-03	4.04E-02
ILMN_1789618	FER	1.96E-01	2.35E-03	1.36E-02
ILMN_1706548	SPTBN4	1.96E-01	2.11E-03	1.24E-02
ILMN_1761540	SEMA3F	1.96E-01	3.07E-04	2.38E-03
ILMN_1798085	EID2B	1.96E-01	7.44E-03	3.63E-02
ILMN_2198270	C20orf106	1.96E-01	7.25E-05	6.81E-04
ILMN_1747020	SGK3	1.96E-01	1.75E-05	2.03E-04
ILMN_1711703	C16orf70	1.96E-01	2.64E-05	2.87E-04
ILMN_1772991	CHPF2	1.96E-01	4.41E-04	3.25E-03
ILMN_2203807	MRP63	1.96E-01	1.81E-03	1.09E-02
ILMN_2380688	B4GALT4	1.95E-01	4.72E-03	2.47E-02
ILMN_1704765	ZSCAN22	1.95E-01	3.52E-05	3.67E-04
ILMN_1765500	NDUFV3	1.95E-01	5.79E-04	4.10E-03
ILMN_2273331	CCDC46	1.95E-01	4.87E-03	2.54E-02
ILMN_1804642	SMUG1	1.95E-01	8.50E-03	4.06E-02
ILMN_1776845	HIST1H3A	1.95E-01	1.04E-03	6.77E-03
ILMN_1813625	TRIM25	1.95E-01	6.23E-03	3.12E-02
ILMN_1813091	ARL1	1.95E-01	9.57E-06	1.21E-04
ILMN_1749009	REXO2	1.95E-01	3.64E-03	1.98E-02
ILMN_3247533	LOC100133840	1.95E-01	3.41E-03	1.87E-02

ILMN_1758918	BRD2	1.95E-01	4.78E-04	3.48E-03
ILMN_3180557	CYB561D1	1.95E-01	9.52E-04	6.28E-03
ILMN_1728914	PRUNE	1.95E-01	1.41E-03	8.74E-03
ILMN_2327947	SLC25A25	1.95E-01	6.47E-04	4.51E-03
ILMN_1656382	BOLA2	1.95E-01	4.48E-04	3.29E-03
ILMN_1796316	MMP9	1.94E-01	2.87E-05	3.09E-04
ILMN_1704063	KCNH3	1.94E-01	3.14E-03	1.74E-02
ILMN_2312296	PCBP2	1.94E-01	1.82E-03	1.09E-02
ILMN_1791792	C12orf5	1.94E-01	9.50E-04	6.27E-03
ILMN_1664294	LEPRE1	1.94E-01	3.76E-03	2.03E-02
ILMN_1772466	SH2D3A	1.94E-01	1.48E-03	9.13E-03
ILMN_1676941	LOC642809	1.94E-01	1.89E-05	2.16E-04
ILMN_1667432	HYAL3	1.94E-01	9.27E-03	4.37E-02
ILMN_1806906	SSR3	1.94E-01	1.95E-03	1.16E-02
ILMN_2252295	ELF2	1.94E-01	7.29E-05	6.84E-04
ILMN_3235912	C7orf38	1.94E-01	5.79E-04	4.10E-03
ILMN_1682245	IFNB1	1.94E-01	1.63E-03	9.92E-03
ILMN_1764780	SVOP	1.94E-01	1.03E-02	4.76E-02
ILMN_1660111	UCHL3	1.94E-01	8.17E-03	3.92E-02
ILMN_2209115	MAK	1.93E-01	3.84E-06	5.62E-05
ILMN_1767441	FBXL14	1.93E-01	6.67E-04	4.63E-03
ILMN_1674874	MFSD10	1.93E-01	1.67E-03	1.01E-02
ILMN_1678477	C9orf85	1.93E-01	4.55E-03	2.39E-02
ILMN_1904135		1.93E-01	9.00E-06	1.15E-04
ILMN_1730799	PHF12	1.93E-01	8.01E-05	7.42E-04
ILMN_1697440	PRPF4	1.93E-01	3.56E-03	1.94E-02
ILMN_1712773	SPAG1	1.93E-01	1.02E-02	4.73E-02
ILMN_1736701	LOC650580	1.93E-01	1.35E-03	8.41E-03
ILMN_1760303	PIK3R1	1.93E-01	2.30E-03	1.34E-02
ILMN_3281594	LOC646093	1.93E-01	4.44E-04	3.26E-03
ILMN_1750158	ACOX1	1.93E-01	4.27E-04	3.16E-03
ILMN_1671905	C10orf78	1.93E-01	1.01E-02	4.68E-02
ILMN_1762608	KIAA1632	1.93E-01	5.35E-04	3.83E-03
ILMN_1880406		1.93E-01	9.29E-03	4.37E-02
ILMN_1811616	EEPD1	1.93E-01	1.33E-03	8.33E-03
ILMN_1726873	TPCN2	1.93E-01	2.30E-03	1.34E-02
ILMN_1736068	CNOT8	1.93E-01	3.03E-03	1.69E-02
ILMN_2337740	TULP4	1.93E-01	5.24E-04	3.76E-03
ILMN_1779536	NCRNA00153	1.92E-01	7.01E-05	6.62E-04
ILMN_1691293	RNF185	1.92E-01	2.06E-04	1.67E-03
ILMN_1671039	GALNT3	1.92E-01	1.92E-03	1.14E-02
ILMN_3225669	RPS10P3	1.92E-01	6.17E-03	3.09E-02
ILMN_1665622	WDR5B	1.92E-01	1.39E-03	8.66E-03
ILMN_1903021		1.92E-01	4.09E-04	3.04E-03
ILMN_1664960	KIAA0284	1.92E-01	1.16E-05	1.43E-04
ILMN_1675435	ANKRD16	1.92E-01	1.10E-04	9.71E-04

ILMN_1718336	C7orf50	1.92E-01	7.64E-04	5.18E-03
ILMN_1688755	AAK1	1.91E-01	8.33E-05	7.66E-04
ILMN_1655241	PURG	1.91E-01	2.45E-03	1.41E-02
ILMN_1725346	SNAPC1	1.91E-01	4.73E-04	3.45E-03
ILMN_3206064	LOC646674	1.91E-01	6.85E-05	6.49E-04
ILMN_1768510	MAN2B2	1.91E-01	3.37E-03	1.85E-02
ILMN_1789001	SLC35B2	1.91E-01	1.05E-03	6.82E-03
ILMN_2367743	TUBG1	1.91E-01	2.79E-04	2.19E-03
ILMN_1753498	COASY	1.91E-01	6.01E-03	3.03E-02
ILMN_1651705	CAT	1.91E-01	2.33E-05	2.59E-04
ILMN_1902094		1.91E-01	4.15E-03	2.21E-02
ILMN_1661172	LOC650034	1.91E-01	3.75E-05	3.87E-04
ILMN_1657446	C1orf57	1.90E-01	3.57E-04	2.71E-03
ILMN_2280731	C17orf95	1.90E-01	4.92E-04	3.56E-03
ILMN_1773200	CP110	1.90E-01	1.73E-04	1.44E-03
ILMN_3297675	LOC729438	1.90E-01	7.96E-04	5.38E-03
ILMN_3199737	LOC727865	1.90E-01	6.10E-03	3.07E-02
ILMN_1682929	SYTL2	1.90E-01	2.07E-03	1.22E-02
ILMN_1682518	LOC653197	1.90E-01	6.27E-04	4.39E-03
ILMN_1761834	LOC401525	1.90E-01	2.54E-04	2.01E-03
ILMN_2038773	UBC	1.90E-01	7.68E-05	7.16E-04
ILMN_3237124	LOC728125	1.90E-01	1.49E-03	9.15E-03
ILMN_3200597	LOC441154	1.89E-01	9.42E-03	4.42E-02
ILMN_3276019	LOC728060	1.89E-01	6.89E-03	3.40E-02
ILMN_2328378	OSBPL3	1.89E-01	1.06E-05	1.32E-04
ILMN_3207490	LOC441896	1.89E-01	1.09E-02	5.00E-02
ILMN_1791296	DPY19L1	1.89E-01	1.41E-04	1.21E-03
ILMN_1807176	TAF3	1.89E-01	8.71E-05	7.95E-04
ILMN_1751378	RARS	1.88E-01	7.28E-04	4.99E-03
ILMN_1740606	RTKN2	1.88E-01	1.76E-04	1.46E-03
ILMN_2362847	PPARA	1.88E-01	1.81E-03	1.09E-02
ILMN_1728498	PCBP4	1.88E-01	7.89E-03	3.81E-02
ILMN_1674366	LHX4	1.88E-01	4.01E-06	5.85E-05
ILMN_1656900	SULT1A1	1.88E-01	1.07E-03	6.93E-03
ILMN_2055781	KLRF1	1.88E-01	4.87E-03	2.54E-02
ILMN_1660775	LOC650152	1.88E-01	6.66E-03	3.30E-02
ILMN_1752877	PHF1	1.88E-01	2.86E-04	2.24E-03
ILMN_1733869	OGDH	1.88E-01	2.34E-03	1.35E-02
ILMN_3286813	LOC391019	1.88E-01	2.82E-03	1.59E-02
ILMN_3239276	SNORA46	1.88E-01	2.66E-03	1.51E-02
ILMN_3276209	LOC727865	1.87E-01	4.25E-04	3.14E-03
ILMN_1742250	CCNH	1.87E-01	2.26E-04	1.82E-03
ILMN_3208014	LOC100131866	1.87E-01	7.98E-03	3.84E-02
ILMN_1724598	RABL2A	1.87E-01	8.06E-03	3.88E-02
ILMN_1873621	NTN1	1.87E-01	1.92E-03	1.14E-02
ILMN_2400583	USP9X	1.87E-01	2.16E-03	1.27E-02

ILMN_1703866	SUPT5H	1.87E-01	8.74E-03	4.15E-02
ILMN_1703697	LANCL1	1.87E-01	2.30E-03	1.34E-02
ILMN_1703072	CREB3	1.87E-01	2.71E-03	1.54E-02
ILMN_1730291	ATP1B1	1.87E-01	3.46E-04	2.64E-03
ILMN_1874735		1.87E-01	6.80E-04	4.70E-03
ILMN_1711459	CCNDBP1	1.87E-01	7.43E-03	3.62E-02
ILMN_1794122	ZNF79	1.87E-01	2.71E-03	1.54E-02
ILMN_3236270	ACAP2	1.87E-01	1.14E-03	7.33E-03
ILMN_2246131	BCAN	1.86E-01	6.78E-03	3.35E-02
ILMN_1685574	TSC22D2	1.86E-01	8.72E-03	4.14E-02
ILMN_1743204	DUSP8	1.86E-01	1.48E-03	9.12E-03
ILMN_3292320	LOC642513	1.86E-01	3.66E-03	1.99E-02
ILMN_1795767	GRHL1	1.86E-01	1.22E-03	7.71E-03
ILMN_1733377	FASTKD5	1.86E-01	3.81E-04	2.86E-03
ILMN_3301197	MAPKSP1	1.86E-01	2.43E-03	1.40E-02
ILMN_1782178	LOC642946	1.86E-01	1.35E-03	8.40E-03
ILMN_1772369	PDHA1	1.86E-01	6.63E-03	3.28E-02
ILMN_1738704	TRIM26	1.86E-01	2.49E-03	1.43E-02
ILMN_3201419	LOC387924	1.85E-01	2.38E-04	1.91E-03
ILMN_1720270	CDR2	1.85E-01	2.51E-06	3.89E-05
ILMN_3225300	LOC728532	1.85E-01	5.18E-03	2.67E-02
ILMN_1671661	HSD17B7	1.85E-01	8.15E-03	3.91E-02
ILMN_3244096	ZFC3H1	1.85E-01	4.50E-04	3.30E-03
ILMN_1673409	MGC16121	1.85E-01	9.51E-03	4.46E-02
ILMN_1674551	SMAD5	1.85E-01	2.33E-03	1.35E-02
ILMN_1697317	DYNLRB2	1.85E-01	2.08E-03	1.22E-02
ILMN_1705605	MGC13005	1.84E-01	1.70E-04	1.42E-03
ILMN_1699100	SOAT1	1.84E-01	4.33E-04	3.19E-03
ILMN_3243664	LOC440353	1.84E-01	9.76E-03	4.56E-02
ILMN_1775944	ABI2	1.84E-01	2.56E-03	1.47E-02
ILMN_2081335	C7orf44	1.84E-01	2.23E-03	1.30E-02
ILMN_2305544	DBI	1.84E-01	5.42E-03	2.77E-02
ILMN_1759326	P2RX7	1.84E-01	1.96E-04	1.61E-03
ILMN_1838310		1.84E-01	2.01E-04	1.63E-03
ILMN_1653599	ATP5D	1.84E-01	4.85E-04	3.52E-03
ILMN_1789500	KIAA1875	1.84E-01	3.81E-03	2.05E-02
ILMN_1789642	DNAJC5	1.84E-01	1.47E-03	9.04E-03
ILMN_3247504	HAUS5	1.84E-01	3.14E-03	1.74E-02
ILMN_1769508	PIGX	1.84E-01	2.99E-04	2.32E-03
ILMN_1784661	TMEM2	1.84E-01	4.28E-04	3.16E-03
ILMN_1694755	FSD1	1.84E-01	3.62E-04	2.74E-03
ILMN_1665335	DIABLO	1.84E-01	2.69E-03	1.53E-02
ILMN_1855286		1.84E-01	4.84E-04	3.51E-03
ILMN_2089167	RHOD	1.83E-01	4.63E-03	2.43E-02
ILMN_1791039	RTF1	1.83E-01	6.56E-05	6.25E-04
ILMN_2321416	DIAPH1	1.83E-01	1.32E-03	8.28E-03

ILMN_1720708	CSNK1D	1.83E-01	5.83E-06	7.99E-05
ILMN_3247325	LOC100190939	1.83E-01	3.36E-05	3.53E-04
ILMN_2208903	CD52	1.83E-01	2.04E-04	1.66E-03
ILMN_1791650	C5orf36	1.83E-01	9.51E-06	1.20E-04
ILMN_1727183	ZNF763	1.83E-01	8.26E-04	5.56E-03
ILMN_1724825	PCBP2	1.83E-01	8.80E-04	5.87E-03
ILMN_3178050	CYTSB	1.83E-01	6.31E-03	3.15E-02
ILMN_1656200	KATNAL2	1.83E-01	1.72E-04	1.44E-03
ILMN_1799024	VAC14	1.83E-01	7.39E-04	5.05E-03
ILMN_1716797	CD302	1.83E-01	3.66E-04	2.76E-03
ILMN_1786433	BCCIP	1.83E-01	1.77E-03	1.07E-02
ILMN_1732154	BCAN	1.83E-01	2.39E-03	1.38E-02
ILMN_1654414	ACSL3	1.83E-01	1.26E-03	7.92E-03
ILMN_2376980	FSD1	1.83E-01	2.17E-04	1.75E-03
ILMN_1815063	PXK	1.83E-01	1.03E-02	4.78E-02
ILMN_1724806	RAPSN	1.83E-01	4.02E-03	2.15E-02
ILMN_1653163	ZSCAN2	1.82E-01	5.83E-05	5.64E-04
ILMN_2094266	HES2	1.82E-01	5.39E-03	2.76E-02
ILMN_2092223	TMEM92	1.82E-01	1.65E-04	1.38E-03
ILMN_3264466	FAM54B	1.82E-01	2.82E-03	1.59E-02
ILMN_2207363	RABAC1	1.82E-01	2.40E-03	1.39E-02
ILMN_1815283	SULT1A3	1.82E-01	5.48E-04	3.91E-03
ILMN_1676411	PMS2L2	1.82E-01	3.10E-03	1.72E-02
ILMN_1727361	LEMD3	1.81E-01	3.92E-03	2.11E-02
ILMN_3290353	LOC644790	1.81E-01	8.26E-03	3.96E-02
ILMN_3241051	LOC644907	1.81E-01	2.60E-03	1.49E-02
ILMN_1768194	BIRC2	1.81E-01	6.71E-04	4.65E-03
ILMN_1654602	SDHALP1	1.81E-01	6.48E-03	3.22E-02
ILMN_1911503		1.81E-01	1.02E-04	9.08E-04
ILMN_1664242	C20orf12	1.81E-01	2.11E-03	1.24E-02
ILMN_1814315	PBXIP1	1.81E-01	1.43E-03	8.84E-03
ILMN_3244030	OXNAD1	1.81E-01	4.03E-03	2.16E-02
ILMN_1738099	C2orf34	1.81E-01	6.91E-03	3.40E-02
ILMN_1722985	ZNF658B	1.81E-01	2.67E-03	1.52E-02
ILMN_1680353	NSF	1.81E-01	2.61E-03	1.49E-02
ILMN_2295252	C9orf72	1.81E-01	8.89E-04	5.92E-03
ILMN_1774901	GDPD3	1.81E-01	9.19E-05	8.34E-04
ILMN_1783253	DDX12	1.81E-01	1.90E-03	1.13E-02
ILMN_1778377	ERGIC1	1.81E-01	1.01E-02	4.70E-02
ILMN_2050654	SAV1	1.81E-01	1.53E-04	1.29E-03
ILMN_1660440	FAM108B1	1.80E-01	3.12E-04	2.41E-03
ILMN_2338447	ARID4A	1.80E-01	4.01E-04	2.99E-03
ILMN_1753393	OSGEP	1.80E-01	8.82E-04	5.88E-03
ILMN_2070052	LOC613037	1.80E-01	2.39E-03	1.38E-02
ILMN_1661492	ARRDC1	1.80E-01	5.44E-04	3.89E-03
ILMN_1786046	CASP9	1.80E-01	3.76E-03	2.03E-02

ILMN_2183389	TTC9C	1.80E-01	1.50E-04	1.27E-03
ILMN_1652309	TTC8	1.80E-01	5.23E-03	2.69E-02
ILMN_2356284	PFDN5	1.80E-01	2.18E-03	1.28E-02
ILMN_1697529	RNF10	1.80E-01	4.08E-03	2.18E-02
ILMN_2255310	RPS15A	1.80E-01	9.52E-03	4.46E-02
ILMN_1806510	VPS53	1.80E-01	1.61E-03	9.77E-03
ILMN_1781431	GLCCI1	1.79E-01	7.40E-04	5.05E-03
ILMN_1726333	RALGAPA2	1.79E-01	2.91E-04	2.27E-03
ILMN_2215211	ZNF514	1.79E-01	3.00E-03	1.68E-02
ILMN_1754207	PLAC1	1.79E-01	1.65E-04	1.38E-03
ILMN_1779989	GLI1	1.79E-01	1.00E-03	6.54E-03
ILMN_2387175	WDR31	1.79E-01	1.28E-04	1.11E-03
ILMN_2075829	LMBRD2	1.79E-01	2.72E-04	2.14E-03
ILMN_1724581	EDC3	1.79E-01	3.35E-05	3.52E-04
ILMN_3246708	SNORA5C	1.79E-01	1.47E-03	9.05E-03
ILMN_1896967		1.78E-01	3.48E-03	1.90E-02
ILMN_3249669	HEATR5A	1.78E-01	3.15E-03	1.75E-02
ILMN_3190833	CCRL2	1.78E-01	5.19E-03	2.68E-02
ILMN_1679267	TGM2	1.78E-01	8.77E-03	4.16E-02
ILMN_1805696	DFFA	1.78E-01	9.61E-04	6.33E-03
ILMN_1699852	CES8	1.78E-01	3.83E-03	2.06E-02
ILMN_1795852	CCNE1	1.77E-01	1.92E-03	1.14E-02
ILMN_2129910	SLC12A5	1.77E-01	4.64E-05	4.63E-04
ILMN_1753805	PRKD2	1.77E-01	9.63E-04	6.34E-03
ILMN_1677968	KIAA0649	1.77E-01	6.50E-05	6.20E-04
ILMN_3238417	LOC100133662	1.77E-01	1.01E-03	6.58E-03
ILMN_1670398	BCR	1.77E-01	4.86E-04	3.53E-03
ILMN_1748591	ODC1	1.77E-01	5.32E-03	2.73E-02
ILMN_3250899	SULT1A3	1.77E-01	4.22E-04	3.12E-03
ILMN_3238221	FLJ42627	1.77E-01	2.98E-03	1.67E-02
ILMN_1660927	MGC35361	1.77E-01	2.09E-03	1.23E-02
ILMN_1812380	CHKA	1.77E-01	9.20E-04	6.09E-03
ILMN_1726967	TWSG1	1.77E-01	6.23E-03	3.12E-02
ILMN_3240069	SCARNA4	1.77E-01	6.23E-03	3.12E-02
ILMN_1811148	BMP2K	1.77E-01	4.17E-03	2.22E-02
ILMN_2113728	FLJ31568	1.77E-01	2.41E-03	1.39E-02
ILMN_1796900	NUDCD3	1.76E-01	7.18E-03	3.52E-02
ILMN_1798971	FZD5	1.76E-01	8.23E-03	3.94E-02
ILMN_2383300	PTPRU	1.76E-01	2.87E-03	1.62E-02
ILMN_1693941	IGSF9	1.76E-01	9.13E-05	8.29E-04
ILMN_1744980	ZCCHC7	1.76E-01	1.01E-02	4.68E-02
ILMN_1763891	FLJ35258	1.76E-01	5.70E-05	5.54E-04
ILMN_3240986	LOC100134584	1.76E-01	2.17E-03	1.27E-02
ILMN_2376625	VHL	1.76E-01	1.03E-02	4.75E-02
ILMN_2103591	MORC2	1.76E-01	7.51E-04	5.11E-03
ILMN_3235718	LOC91316	1.76E-01	6.75E-04	4.68E-03

ILMN_1656868	LOC23117	1.76E-01	7.37E-03	3.60E-02
ILMN_3202373	LOC392288	1.75E-01	2.02E-03	1.19E-02
ILMN_1758093	C14orf101	1.75E-01	4.95E-03	2.57E-02
ILMN_1657483	SEC23B	1.75E-01	1.63E-04	1.36E-03
ILMN_1795085	CCDC116	1.75E-01	3.69E-04	2.78E-03
ILMN_1783350	PCNXL3	1.75E-01	3.63E-03	1.97E-02
ILMN_2390974	DNAJB2	1.75E-01	1.39E-03	8.64E-03
ILMN_1776119	ABCC10	1.75E-01	2.54E-04	2.02E-03
ILMN_1671748	LOC644399	1.75E-01	4.08E-03	2.18E-02
ILMN_1782751	GLI4	1.74E-01	6.06E-04	4.26E-03
ILMN_1739335	LOC400948	1.74E-01	4.02E-03	2.15E-02
ILMN_1767349	ABCB4	1.74E-01	7.02E-03	3.45E-02
ILMN_1799289	MRPL55	1.74E-01	7.94E-03	3.83E-02
ILMN_1802414	CA13	1.74E-01	3.95E-04	2.95E-03
ILMN_1670881	CHST6	1.74E-01	2.09E-04	1.70E-03
ILMN_1711109	TOM1L2	1.74E-01	6.50E-04	4.53E-03
ILMN_2275502	RAPH1	1.74E-01	5.28E-04	3.79E-03
ILMN_1864900	MIAT	1.74E-01	9.20E-04	6.09E-03
ILMN_1675172	RGS9BP	1.74E-01	1.61E-04	1.35E-03
ILMN_1799672	CROCC	1.73E-01	6.87E-03	3.39E-02
ILMN_1658010	CENPI	1.73E-01	3.78E-03	2.04E-02
ILMN_2202930	PCYT2	1.73E-01	5.48E-03	2.80E-02
ILMN_1662686	USPL1	1.73E-01	2.06E-04	1.67E-03
ILMN_3241136	RNASEK	1.73E-01	1.61E-03	9.79E-03
ILMN_1737484	RTN4R	1.73E-01	5.75E-03	2.91E-02
ILMN_1775405	ARL4A	1.73E-01	7.22E-03	3.53E-02
ILMN_1755732	ARMC9	1.73E-01	2.34E-03	1.36E-02
ILMN_1833212		1.73E-01	3.59E-03	1.95E-02
ILMN_2366177	IFT122	1.73E-01	1.39E-03	8.62E-03
ILMN_1755487	TTC28	1.73E-01	1.05E-03	6.82E-03
ILMN_1745469	SEC24B	1.73E-01	1.24E-03	7.85E-03
ILMN_1660900	SNORA7B	1.73E-01	3.55E-04	2.69E-03
ILMN_2121068	ADAM17	1.72E-01	8.12E-04	5.47E-03
ILMN_2262462	C19orf29	1.72E-01	3.47E-03	1.90E-02
ILMN_1873967		1.72E-01	7.79E-03	3.77E-02
ILMN_1676626	ARID4A	1.72E-01	5.76E-04	4.08E-03
ILMN_1697773	ZNF517	1.72E-01	4.77E-04	3.47E-03
ILMN_3222425	LOC729852	1.72E-01	7.70E-05	7.17E-04
ILMN_1716983	LILRA2	1.72E-01	2.13E-04	1.72E-03
ILMN_1765644	COMMD8	1.72E-01	6.70E-03	3.32E-02
ILMN_2326071	MYL6	1.72E-01	3.37E-03	1.85E-02
ILMN_1679577	SETD3	1.72E-01	1.20E-03	7.60E-03
ILMN_2326075	NTRK1	1.72E-01	9.83E-03	4.59E-02
ILMN_1668484	LRRC47	1.72E-01	1.87E-03	1.12E-02
ILMN_1663145	NOMO3	1.71E-01	1.18E-05	1.45E-04
ILMN_1703016	CACNA1G	1.71E-01	2.19E-03	1.28E-02

ILMN_1810834	MSH5	1.71E-01	5.50E-03	2.81E-02
ILMN_1815102	LCAT	1.71E-01	6.10E-05	5.86E-04
ILMN_1666742	C9orf72	1.70E-01	4.20E-05	4.26E-04
ILMN_3257566	DSTYK	1.70E-01	2.63E-03	1.50E-02
ILMN_1806349	SLC6A8	1.70E-01	8.00E-03	3.85E-02
ILMN_1718950	ZNF433	1.70E-01	5.44E-03	2.78E-02
ILMN_1787509	PRIC285	1.70E-01	2.46E-03	1.41E-02
ILMN_3246910	LOC100190986	1.70E-01	5.14E-03	2.66E-02
ILMN_2341487	C11orf49	1.70E-01	1.02E-03	6.64E-03
ILMN_2413780	SEZ6L2	1.70E-01	2.43E-06	3.77E-05
ILMN_1722845	RAB3B	1.70E-01	1.05E-02	4.86E-02
ILMN_1750075	DMTF1	1.70E-01	2.61E-03	1.49E-02
ILMN_3247681	C2orf68	1.70E-01	1.27E-03	8.01E-03
ILMN_2083818	EXOC8	1.69E-01	4.30E-03	2.28E-02
ILMN_1802082	PRDM8	1.69E-01	4.87E-03	2.54E-02
ILMN_2388585	GOPC	1.69E-01	7.80E-04	5.28E-03
ILMN_1723984	PILRB	1.69E-01	2.38E-03	1.37E-02
ILMN_2222651	MAK10	1.69E-01	1.59E-03	9.67E-03
ILMN_1750961	TM6SF1	1.69E-01	2.53E-03	1.45E-02
ILMN_2413323	GRP	1.69E-01	1.68E-03	1.01E-02
ILMN_1797107	SCLT1	1.69E-01	9.98E-03	4.64E-02
ILMN_1754400	AYP1p1	1.69E-01	1.64E-03	9.97E-03
ILMN_2333687	CD59	1.69E-01	1.09E-03	7.00E-03
ILMN_1804663	THBS3	1.69E-01	3.38E-03	1.86E-02
ILMN_2124816	ZNF34	1.69E-01	1.05E-03	6.80E-03
ILMN_1703564	DYNLRB1	1.68E-01	2.90E-03	1.63E-02
ILMN_1660864	RHBDL1	1.68E-01	3.04E-03	1.70E-02
ILMN_1723414	HACL1	1.68E-01	1.25E-03	7.88E-03
ILMN_1660277	LOC731999	1.68E-01	7.33E-04	5.01E-03
ILMN_1764619	FLJ45244	1.68E-01	3.04E-03	1.69E-02
ILMN_1740291	POLQ	1.68E-01	3.33E-03	1.83E-02
ILMN_1652160	LRBA	1.68E-01	4.68E-03	2.45E-02
ILMN_1677736	TMEM104	1.68E-01	9.37E-04	6.19E-03
ILMN_1745900	LOC641849	1.68E-01	7.85E-03	3.79E-02
ILMN_1769934	GRHL1	1.67E-01	2.60E-03	1.48E-02
ILMN_2148944	ADCY4	1.67E-01	8.16E-03	3.92E-02
ILMN_2303669	SLC4A8	1.67E-01	1.09E-04	9.63E-04
ILMN_3245351	UNC119B	1.67E-01	3.10E-03	1.73E-02
ILMN_1798163	UPF3B	1.67E-01	8.92E-03	4.23E-02
ILMN_1856609		1.67E-01	8.36E-04	5.61E-03
ILMN_3233272	MGC23284	1.67E-01	5.58E-03	2.84E-02
ILMN_1750967	ZKSCAN3	1.67E-01	1.28E-03	8.06E-03
ILMN_1777708	C14orf121	1.66E-01	1.47E-03	9.07E-03
ILMN_2155228	CRLF3	1.66E-01	6.65E-03	3.30E-02
ILMN_3307874	AIM1L	1.66E-01	6.23E-05	5.97E-04
ILMN_1809813	PGF	1.66E-01	1.05E-02	4.83E-02

ILMN_1803357	FLJ40125	1.66E-01	3.85E-04	2.89E-03
ILMN_1723111	HIST1H4A	1.66E-01	6.12E-03	3.08E-02
ILMN_2180352	DIP2B	1.66E-01	4.08E-06	5.93E-05
ILMN_1724407	TACC3	1.66E-01	5.90E-04	4.17E-03
ILMN_3246869	SCARNA21	1.65E-01	6.83E-03	3.37E-02
ILMN_2333146	BCR	1.65E-01	9.34E-03	4.39E-02
ILMN_1801043	GSN	1.65E-01	4.71E-03	2.46E-02
ILMN_3248975	PPP4C	1.65E-01	1.73E-03	1.04E-02
ILMN_3246783	LOC100131330	1.65E-01	4.18E-04	3.10E-03
ILMN_1699170	C5orf45	1.65E-01	1.70E-03	1.03E-02
ILMN_2057389	PEX3	1.64E-01	8.83E-03	4.19E-02
ILMN_1737036	GGT7	1.64E-01	3.58E-03	1.95E-02
ILMN_1661142	TMF1	1.64E-01	1.53E-03	9.36E-03
ILMN_1680196	LAPTM4B	1.64E-01	5.15E-03	2.66E-02
ILMN_1785452	LOC442421	1.64E-01	6.12E-04	4.30E-03
ILMN_1680436	CSHL1	1.64E-01	1.50E-03	9.20E-03
ILMN_1682831	NEB	1.64E-01	2.04E-03	1.20E-02
ILMN_1721623	APOO	1.64E-01	4.22E-03	2.25E-02
ILMN_2119555	TMTC3	1.64E-01	1.06E-02	4.90E-02
ILMN_2060086	ADAM23	1.64E-01	7.23E-03	3.54E-02
ILMN_1797189	MAP3K12	1.64E-01	3.07E-04	2.38E-03
ILMN_1785762	RHOT1	1.64E-01	1.37E-03	8.52E-03
ILMN_1688727	C17orf95	1.64E-01	8.49E-04	5.68E-03
ILMN_1764095	LOC641785	1.64E-01	2.89E-04	2.26E-03
ILMN_1724233	LOC145783	1.63E-01	1.07E-03	6.89E-03
ILMN_1814575	LOC642423	1.63E-01	2.81E-03	1.59E-02
ILMN_1655940	LOC652068	1.63E-01	5.41E-03	2.77E-02
ILMN_1665280	SPCS1	1.63E-01	4.81E-04	3.49E-03
ILMN_1753567	ZNF451	1.63E-01	7.00E-04	4.82E-03
ILMN_2283597	FAM134B	1.63E-01	7.79E-03	3.77E-02
ILMN_1779813	FAM96B	1.63E-01	8.35E-03	3.99E-02
ILMN_3187530	LOC100128765	1.63E-01	6.28E-06	8.51E-05
ILMN_3194508	ASAP2	1.63E-01	3.58E-03	1.95E-02
ILMN_1705746	LSG1	1.63E-01	3.55E-03	1.94E-02
ILMN_1667298	LOC201229	1.63E-01	5.60E-04	3.98E-03
ILMN_1795317	SCAND1	1.63E-01	3.04E-03	1.70E-02
ILMN_2221673	ASNSD1	1.63E-01	8.18E-04	5.50E-03
ILMN_1716080	CBL	1.63E-01	1.08E-02	4.94E-02
ILMN_1751811	PLGLB1	1.63E-01	2.13E-03	1.25E-02
ILMN_2408102	TFIP11	1.63E-01	6.85E-03	3.38E-02
ILMN_1658176	ABCG1	1.63E-01	1.95E-03	1.16E-02
ILMN_1785900	LOC653108	1.63E-01	4.19E-04	3.11E-03
ILMN_1695334	PYGO2	1.62E-01	1.20E-03	7.60E-03
ILMN_1786310	MVK	1.62E-01	7.98E-04	5.39E-03
ILMN_1717975	CYHR1	1.62E-01	4.27E-03	2.27E-02
ILMN_1802315	SPPL2B	1.62E-01	5.83E-04	4.12E-03

ILMN_1655733	RFC2	1.62E-01	1.32E-04	1.14E-03
ILMN_1720988	ABI2	1.62E-01	1.24E-03	7.85E-03
ILMN_1807501	GINS4	1.62E-01	1.41E-03	8.72E-03
ILMN_2050911	SLC22A4	1.62E-01	4.74E-04	3.45E-03
ILMN_1826285		1.62E-01	1.58E-03	9.66E-03
ILMN_1675131	PIH1D1	1.62E-01	4.51E-03	2.37E-02
ILMN_3235036	LOC728024	1.62E-01	4.75E-04	3.46E-03
ILMN_1658635	CLGN	1.62E-01	1.19E-03	7.59E-03
ILMN_2326793	NUP98	1.61E-01	4.76E-04	3.46E-03
ILMN_1709124	ANO8	1.61E-01	2.55E-03	1.46E-02
ILMN_1659259	PRPF6	1.61E-01	1.48E-03	9.11E-03
ILMN_1766798	CENTB2	1.61E-01	2.14E-04	1.73E-03
ILMN_1782504	MTERFD1	1.61E-01	9.84E-03	4.59E-02
ILMN_1718807	SMC3	1.61E-01	4.84E-04	3.51E-03
ILMN_2374683	PTPN13	1.61E-01	3.31E-03	1.82E-02
ILMN_2414436	RBM3	1.61E-01	1.62E-03	9.84E-03
ILMN_1687721	PROC	1.60E-01	5.35E-03	2.75E-02
ILMN_2366246	SEC23B	1.60E-01	2.53E-03	1.45E-02
ILMN_1722127	RAD54B	1.60E-01	8.12E-04	5.47E-03
ILMN_1655577	TIAM1	1.60E-01	9.45E-04	6.24E-03
ILMN_1753165	WNK1	1.60E-01	6.54E-04	4.55E-03
ILMN_1773389	PLTP	1.60E-01	7.80E-04	5.28E-03
ILMN_1713936	GOLGA6B	1.60E-01	2.98E-03	1.67E-02
ILMN_2335669	ZC3H14	1.60E-01	6.26E-03	3.13E-02
ILMN_1754811	FBXO38	1.60E-01	7.07E-04	4.86E-03
ILMN_1715372	CAMKK1	1.60E-01	4.45E-04	3.27E-03
ILMN_1798957	C12orf47	1.60E-01	1.91E-03	1.13E-02
ILMN_1787212	CDKN1A	1.59E-01	1.62E-05	1.90E-04
ILMN_1912827		1.59E-01	7.82E-03	3.78E-02
ILMN_1809013	MYL6	1.59E-01	1.52E-03	9.32E-03
ILMN_1799198	OTUB2	1.59E-01	4.96E-04	3.59E-03
ILMN_2153466	FAM50B	1.59E-01	8.99E-03	4.25E-02
ILMN_3244611	LOC100132060	1.58E-01	4.36E-03	2.30E-02
ILMN_2389984	RGS9	1.58E-01	1.16E-03	7.39E-03
ILMN_1676899	YEATS2	1.58E-01	5.58E-04	3.97E-03
ILMN_1676737	SEPT2	1.58E-01	3.12E-03	1.73E-02
ILMN_1852022	KIAA1881	1.58E-01	1.23E-04	1.07E-03
ILMN_1777528	NCBP1	1.58E-01	9.87E-03	4.60E-02
ILMN_2244841	ALDH4A1	1.58E-01	1.12E-03	7.20E-03
ILMN_1718525	ITIH4	1.58E-01	8.05E-03	3.88E-02
ILMN_1749738	NECAB3	1.57E-01	3.39E-05	3.55E-04
ILMN_2105549	TMEM220	1.57E-01	3.19E-03	1.77E-02
ILMN_1912083		1.57E-01	2.22E-03	1.30E-02
ILMN_3300358	ZNF84	1.57E-01	7.79E-04	5.28E-03
ILMN_1727199	KLC4	1.57E-01	1.28E-03	8.05E-03
ILMN_1653146	MSH4	1.57E-01	7.07E-04	4.86E-03

ILMN_1722809	NRCAM	1.57E-01	9.25E-03	4.36E-02
ILMN_1811995	FANCM	1.57E-01	3.41E-04	2.60E-03
ILMN_2342903	GIPC1	1.57E-01	4.76E-03	2.48E-02
ILMN_1781225	FLJ35801	1.56E-01	1.24E-03	7.84E-03
ILMN_1652490	MANSC1	1.56E-01	6.53E-05	6.22E-04
ILMN_1750180	HIST1H2BB	1.56E-01	9.24E-03	4.35E-02
ILMN_1784780	CIC	1.56E-01	5.07E-03	2.63E-02
ILMN_1783563	TBX19	1.56E-01	1.19E-03	7.57E-03
ILMN_1698673	EFCAB7	1.56E-01	9.36E-04	6.18E-03
ILMN_1716279	CENPE	1.56E-01	5.50E-04	3.92E-03
ILMN_1807072	GOSR2	1.56E-01	4.92E-04	3.56E-03
ILMN_3227263	SLC22A23	1.56E-01	9.40E-03	4.42E-02
ILMN_3248260	LOC100132377	1.56E-01	8.17E-03	3.92E-02
ILMN_2352097	GPR56	1.56E-01	1.13E-04	9.92E-04
ILMN_2135991	C16orf69	1.56E-01	1.22E-03	7.73E-03
ILMN_3241196	LOC729353	1.56E-01	8.70E-04	5.81E-03
ILMN_1780987	RFXANK	1.56E-01	2.08E-03	1.22E-02
ILMN_1779572	LOC440456	1.55E-01	1.23E-03	7.81E-03
ILMN_3236215	LOC100144603	1.55E-01	9.28E-04	6.14E-03
ILMN_1801999	LOC644474	1.55E-01	5.63E-03	2.86E-02
ILMN_1653125	PER1	1.55E-01	4.02E-03	2.15E-02
ILMN_1740010	PCNX	1.55E-01	3.98E-03	2.14E-02
ILMN_1908274		1.55E-01	5.39E-03	2.76E-02
ILMN_1755589	DIP2B	1.55E-01	3.21E-03	1.78E-02
ILMN_2142695	RNF4	1.55E-01	2.26E-03	1.32E-02
ILMN_3236970	LOC339290	1.55E-01	5.09E-03	2.63E-02
ILMN_1789405	C22orf25	1.55E-01	2.66E-04	2.10E-03
ILMN_1869897		1.55E-01	2.15E-03	1.26E-02
ILMN_2354478	CYFIP2	1.55E-01	9.05E-04	6.01E-03
ILMN_1768261	LOC653591	1.55E-01	5.60E-04	3.98E-03
ILMN_1723177	ANAPC5	1.54E-01	4.26E-03	2.26E-02
ILMN_2101810	ARHGAP12	1.54E-01	6.96E-03	3.42E-02
ILMN_1700770	PSKH1	1.54E-01	2.04E-03	1.20E-02
ILMN_1675038	PRMT2	1.54E-01	1.16E-03	7.42E-03
ILMN_1806828	MRI1	1.54E-01	1.74E-03	1.05E-02
ILMN_1804895	C7orf53	1.54E-01	2.71E-03	1.54E-02
ILMN_1684440	PXN	1.54E-01	8.67E-04	5.80E-03
ILMN_1656342	FBXO48	1.54E-01	9.20E-04	6.09E-03
ILMN_3240296	CROCCL2	1.54E-01	4.55E-03	2.39E-02
ILMN_2367186	PIGA	1.54E-01	3.96E-04	2.96E-03
ILMN_2389935	FYTTD1	1.54E-01	3.44E-03	1.88E-02
ILMN_1801105	PRKCD	1.54E-01	2.76E-03	1.56E-02
ILMN_3261811	C14orf167	1.53E-01	8.75E-04	5.84E-03
ILMN_1696806	CTNND1	1.53E-01	1.02E-03	6.66E-03
ILMN_3176840	LOC100128265	1.53E-01	9.49E-04	6.26E-03
ILMN_3236404	SNORD7	1.53E-01	1.37E-03	8.56E-03

ILMN_2143685	CLDN7	1.53E-01	1.81E-03	1.08E-02
ILMN_1825640		1.53E-01	2.51E-03	1.44E-02
ILMN_2261784	CCNY	1.53E-01	1.45E-03	8.94E-03
ILMN_2290338	RCAN1	1.53E-01	2.50E-03	1.43E-02
ILMN_2400143	RPL32	1.53E-01	6.91E-03	3.41E-02
ILMN_1734380	DPF3	1.52E-01	2.40E-04	1.92E-03
ILMN_1686852	LOC401002	1.52E-01	7.49E-04	5.10E-03
ILMN_2337058	PORCN	1.52E-01	1.05E-03	6.83E-03
ILMN_2413615	LLGL2	1.52E-01	2.35E-06	3.67E-05
ILMN_1759732	RFNG	1.52E-01	2.94E-03	1.65E-02
ILMN_2085236	SNX24	1.52E-01	5.97E-04	4.21E-03
ILMN_1756878	SLC39A9	1.52E-01	3.82E-03	2.06E-02
ILMN_1898662		1.52E-01	2.05E-03	1.21E-02
ILMN_1716925	FSIP1	1.51E-01	2.31E-03	1.34E-02
ILMN_1667564	ALDH3A2	1.51E-01	2.32E-03	1.35E-02
ILMN_1746435	HIST1H1E	1.51E-01	1.05E-02	4.83E-02
ILMN_3260017	HNRNPK	1.51E-01	9.03E-03	4.27E-02
ILMN_1779040	INO80B	1.51E-01	4.98E-03	2.58E-02
ILMN_2213199	KIAA1712	1.51E-01	1.50E-04	1.27E-03
ILMN_1666494	CRK	1.51E-01	4.65E-03	2.44E-02
ILMN_3243069	LOC100133692	1.51E-01	5.14E-04	3.70E-03
ILMN_1911115		1.50E-01	4.29E-04	3.17E-03
ILMN_1753885	YTHDF1	1.50E-01	5.90E-03	2.98E-02
ILMN_3276656	LOC728249	1.50E-01	5.51E-03	2.81E-02
ILMN_1684620	SPAG4	1.50E-01	4.06E-04	3.02E-03
ILMN_1748437	LOC646568	1.50E-01	2.81E-03	1.59E-02
ILMN_2392286	IP6K1	1.50E-01	1.97E-03	1.17E-02
ILMN_1691959	AKAP2	1.50E-01	6.99E-03	3.44E-02
ILMN_3266944	LOC100129599	1.50E-01	1.71E-03	1.03E-02
ILMN_1758086	SNAI1	1.50E-01	1.24E-03	7.86E-03
ILMN_1899760		1.50E-01	4.43E-03	2.34E-02
ILMN_2161746	TRIP10	1.49E-01	7.02E-03	3.45E-02
ILMN_1752589	TMEM183A	1.49E-01	3.70E-03	2.00E-02
ILMN_1828216		1.49E-01	3.41E-03	1.87E-02
ILMN_1812321	FAM131C	1.49E-01	8.52E-03	4.06E-02
ILMN_1751500	ADAM15	1.49E-01	3.44E-03	1.88E-02
ILMN_2050921	SNHG8	1.49E-01	8.98E-03	4.25E-02
ILMN_2389429	DCUN1D4	1.49E-01	5.19E-03	2.68E-02
ILMN_2374362	FAM108B1	1.49E-01	2.19E-03	1.28E-02
ILMN_1767927	PTCHD2	1.49E-01	5.57E-03	2.84E-02
ILMN_1686668	GFM2	1.48E-01	7.92E-03	3.82E-02
ILMN_1771728	PXMP4	1.48E-01	4.74E-03	2.48E-02
ILMN_1810712	ARHGEF12	1.48E-01	7.85E-03	3.79E-02
ILMN_1715437	CASC5	1.48E-01	3.76E-03	2.03E-02
ILMN_1674380	TRPC1	1.48E-01	2.39E-04	1.91E-03
ILMN_1758104	PRPS2	1.48E-01	4.84E-04	3.51E-03

ILMN_2074748	HAL	1.48E-01	9.21E-03	4.34E-02
ILMN_3229552	MED7	1.48E-01	1.15E-03	7.37E-03
ILMN_2068698	RSHL3	1.48E-01	1.70E-05	1.98E-04
ILMN_1709399	SLC25A34	1.48E-01	7.43E-03	3.62E-02
ILMN_1794740	CD151	1.48E-01	2.01E-04	1.63E-03
ILMN_1698533	IDH3A	1.48E-01	5.16E-03	2.66E-02
ILMN_1906110		1.48E-01	9.78E-05	8.78E-04
ILMN_1725831	PLAC2	1.48E-01	5.38E-03	2.76E-02
ILMN_1669390	PPP1R13L	1.48E-01	2.52E-03	1.44E-02
ILMN_2141157	RANBP9	1.47E-01	8.10E-03	3.90E-02
ILMN_2177460	AQR	1.47E-01	5.64E-03	2.87E-02
ILMN_1779347	GARNL3	1.47E-01	1.48E-03	9.10E-03
ILMN_1736939	UGCG	1.47E-01	5.12E-03	2.65E-02
ILMN_1675594	YY1AP1	1.47E-01	1.61E-04	1.35E-03
ILMN_1754468	RGS9	1.47E-01	9.95E-04	6.51E-03
ILMN_1689876	LOC648189	1.47E-01	2.51E-03	1.44E-02
ILMN_1801516	GPC1	1.47E-01	4.33E-03	2.29E-02
ILMN_1789599	NBL1	1.47E-01	1.50E-03	9.22E-03
ILMN_1695378	SREBF1	1.47E-01	6.11E-04	4.30E-03
ILMN_1733511	GOLGA3	1.47E-01	1.14E-03	7.32E-03
ILMN_1745807	TMEM62	1.47E-01	5.04E-03	2.61E-02
ILMN_2312906	MIA3	1.46E-01	7.92E-03	3.82E-02
ILMN_1686884	IL1RAP	1.46E-01	4.23E-04	3.13E-03
ILMN_1751092	AMBN	1.46E-01	8.83E-04	5.89E-03
ILMN_1741768	TMPRSS3	1.46E-01	4.19E-05	4.25E-04
ILMN_1882315		1.46E-01	2.00E-03	1.18E-02
ILMN_1663149	SRP54	1.46E-01	6.14E-04	4.31E-03
ILMN_2278152	TPM1	1.46E-01	4.76E-03	2.48E-02
ILMN_3181411	ATL1	1.46E-01	3.59E-03	1.95E-02
ILMN_1680109	COL4A3BP	1.45E-01	2.41E-03	1.39E-02
ILMN_2216815	MAP7	1.45E-01	6.90E-04	4.77E-03
ILMN_1736180	FRAT1	1.45E-01	4.96E-03	2.58E-02
ILMN_1801464	ANKFY1	1.45E-01	6.48E-03	3.22E-02
ILMN_2394027	CLK3	1.45E-01	7.27E-03	3.55E-02
ILMN_1785037	SSR2	1.45E-01	1.42E-03	8.82E-03
ILMN_2380243	SMAGP	1.45E-01	1.10E-04	9.74E-04
ILMN_2118663	ERV3	1.45E-01	1.67E-03	1.01E-02
ILMN_1891857		1.45E-01	7.83E-03	3.79E-02
ILMN_1754370	SPAG8	1.45E-01	3.12E-03	1.73E-02
ILMN_1838885	KIAA1632	1.45E-01	7.96E-04	5.38E-03
ILMN_3253126	FLJ41484	1.44E-01	7.40E-04	5.05E-03
ILMN_1795055	LRRC3	1.44E-01	1.08E-02	4.96E-02
ILMN_1802458	AGTRAP	1.44E-01	4.66E-04	3.40E-03
ILMN_1916292		1.44E-01	7.60E-03	3.69E-02
ILMN_1660674	LOC642181	1.44E-01	7.55E-04	5.13E-03
ILMN_1782079	ZFP3	1.44E-01	4.21E-04	3.12E-03

ILMN_2353033	FUBP3	1.44E-01	1.10E-03	7.07E-03
ILMN_1720996	SLC12A2	1.44E-01	1.02E-02	4.73E-02
ILMN_1806147	GNG3	1.44E-01	2.47E-03	1.42E-02
ILMN_1689712	LOC440927	1.44E-01	4.80E-03	2.50E-02
ILMN_2278850	RAB24	1.44E-01	1.96E-03	1.16E-02
ILMN_1678707	TAF15	1.44E-01	7.50E-03	3.65E-02
ILMN_1653646	UBL7	1.44E-01	7.11E-03	3.49E-02
ILMN_1678494	ZNF438	1.43E-01	4.12E-04	3.07E-03
ILMN_3236741	HCG26	1.43E-01	9.13E-03	4.31E-02
ILMN_1721167	MYT1	1.43E-01	2.84E-03	1.60E-02
ILMN_3187470	LOC100129094	1.43E-01	5.35E-03	2.74E-02
ILMN_1779882	ANKRD43	1.43E-01	1.40E-03	8.67E-03
ILMN_2244009	LBH	1.43E-01	7.60E-04	5.16E-03
ILMN_1656404	ZNF251	1.43E-01	3.33E-03	1.83E-02
ILMN_1709484	BLM	1.43E-01	3.06E-03	1.70E-02
ILMN_1674795	FLJ33996	1.43E-01	4.69E-03	2.46E-02
ILMN_1677532	TARDBP	1.43E-01	9.23E-03	4.35E-02
ILMN_3235974	THNSL2	1.43E-01	4.44E-03	2.34E-02
ILMN_2193175	C3orf1	1.42E-01	1.04E-02	4.82E-02
ILMN_2253246	DNAJC28	1.42E-01	1.91E-04	1.57E-03
ILMN_1818632		1.42E-01	8.11E-04	5.47E-03
ILMN_1788711	HSN2	1.42E-01	3.31E-03	1.83E-02
ILMN_1801378	COQ3	1.42E-01	6.80E-04	4.70E-03
ILMN_1744347	LOC127295	1.42E-01	1.02E-02	4.73E-02
ILMN_2155998	PSMD6	1.42E-01	1.01E-03	6.58E-03
ILMN_3286375	LOC728179	1.42E-01	1.58E-03	9.65E-03
ILMN_1749218	TOPORS	1.42E-01	5.03E-03	2.61E-02
ILMN_3307483	MCF2L	1.42E-01	2.92E-03	1.64E-02
ILMN_1748730	CTRC	1.42E-01	2.86E-05	3.07E-04
ILMN_2148497	C20orf107	1.42E-01	4.54E-03	2.39E-02
ILMN_1720024	IL11RA	1.42E-01	7.18E-04	4.93E-03
ILMN_1873034		1.42E-01	2.50E-04	1.98E-03
ILMN_3274671	LOC283481	1.42E-01	8.15E-03	3.91E-02
ILMN_1667748	ANKRD33	1.42E-01	9.98E-04	6.53E-03
ILMN_1692588	C14orf125	1.42E-01	5.77E-03	2.92E-02
ILMN_1743055	NAT1	1.41E-01	3.76E-04	2.83E-03
ILMN_1814661	PHLPP1	1.41E-01	6.05E-03	3.04E-02
ILMN_2154223	CEP76	1.41E-01	2.27E-03	1.32E-02
ILMN_1709634	CMBL	1.41E-01	5.87E-03	2.97E-02
ILMN_2336133	SULT1A4	1.41E-01	1.05E-02	4.86E-02
ILMN_2172269	TMEM183B	1.41E-01	4.31E-03	2.28E-02
ILMN_1752758	BTN2A2	1.41E-01	1.20E-04	1.05E-03
ILMN_2382505	SLC22A18	1.41E-01	1.79E-03	1.08E-02
ILMN_1800871	RAB6A	1.41E-01	5.83E-03	2.95E-02
ILMN_1689378	CCRN4L	1.41E-01	1.05E-02	4.86E-02
ILMN_1749070	HLA-DPB1	1.41E-01	2.30E-04	1.85E-03

ILMN_1651769	ZFYVE16	1.41E-01	5.27E-03	2.71E-02
ILMN_2070355	SDHAF1	1.41E-01	8.06E-04	5.44E-03
ILMN_1682316	TRIM33	1.40E-01	1.74E-04	1.44E-03
ILMN_1765523	TOLLIP	1.40E-01	2.95E-03	1.65E-02
ILMN_3239885	LOC100134100	1.40E-01	5.62E-03	2.86E-02
ILMN_1764314	FGD1	1.40E-01	3.40E-04	2.59E-03
ILMN_2376833	ZNF200	1.40E-01	3.21E-03	1.78E-02
ILMN_2350421	C19orf29	1.40E-01	3.83E-03	2.07E-02
ILMN_1746836	PTAFR	1.40E-01	3.68E-03	2.00E-02
ILMN_1655504	IFT81	1.40E-01	6.46E-05	6.16E-04
ILMN_1784178	IFT172	1.40E-01	9.52E-03	4.46E-02
ILMN_1766560	ANKHD1-EIF4EBP3	1.40E-01	6.75E-03	3.34E-02
ILMN_1661427	RFNG	1.40E-01	7.69E-03	3.73E-02
ILMN_1718852	PLCL1	1.40E-01	1.39E-03	8.65E-03
ILMN_2323633	TPD52L2	1.39E-01	1.08E-03	6.98E-03
ILMN_3228269	LOC730236	1.39E-01	5.82E-04	4.12E-03
ILMN_3239164	SEPT13	1.39E-01	2.27E-04	1.83E-03
ILMN_1869442		1.39E-01	6.47E-03	3.22E-02
ILMN_1661066	LOC644596	1.39E-01	3.93E-03	2.11E-02
ILMN_1668270	ZDHHC18	1.39E-01	5.33E-03	2.74E-02
ILMN_1653443	CDK2	1.39E-01	6.85E-03	3.38E-02
ILMN_3304012	LOC729806	1.39E-01	5.94E-03	3.00E-02
ILMN_1664186	MFN1	1.38E-01	2.45E-04	1.95E-03
ILMN_3272299	FLJ42562	1.38E-01	3.71E-04	2.79E-03
ILMN_1815057	PDGFRB	1.38E-01	3.02E-03	1.69E-02
ILMN_1811272	GPR81	1.38E-01	4.24E-03	2.25E-02
ILMN_1880086		1.38E-01	8.11E-03	3.90E-02
ILMN_1655283	INPP4A	1.38E-01	1.63E-03	9.89E-03
ILMN_3237574	LOC732445	1.38E-01	1.17E-03	7.49E-03
ILMN_1786186	GGTL3	1.38E-01	4.16E-03	2.22E-02
ILMN_2117613	PDE4C	1.38E-01	6.87E-04	4.75E-03
ILMN_1679256	AP3B2	1.38E-01	9.70E-03	4.53E-02
ILMN_1704236	MAX	1.37E-01	3.04E-03	1.69E-02
ILMN_1781537	KDM1B	1.37E-01	4.55E-03	2.39E-02
ILMN_2118864	RAB1A	1.37E-01	7.43E-03	3.62E-02
ILMN_2406132	LILRB3	1.37E-01	4.69E-03	2.45E-02
ILMN_1656883	LOC643201	1.37E-01	3.58E-04	2.71E-03
ILMN_1674506	MED23	1.37E-01	5.19E-03	2.68E-02
ILMN_1751887	PREP	1.36E-01	2.66E-03	1.52E-02
ILMN_3251550	PHLDA1	1.36E-01	4.86E-03	2.53E-02
ILMN_1777650	LOC441151	1.36E-01	8.20E-05	7.55E-04
ILMN_1772821	KIAA1671	1.36E-01	5.70E-04	4.05E-03
ILMN_2362982	ARID4B	1.36E-01	7.78E-04	5.27E-03
ILMN_1810826	MAK10	1.35E-01	1.54E-03	9.44E-03
ILMN_2283726	SPATA7	1.35E-01	7.34E-04	5.02E-03
ILMN_1689473	ATP6V1H	1.35E-01	1.34E-03	8.38E-03

ILMN_1725594	FAM188A	1.35E-01	2.03E-03	1.20E-02
ILMN_1725312	RASA1	1.35E-01	4.52E-03	2.38E-02
ILMN_1731086	ISL2	1.35E-01	5.81E-03	2.94E-02
ILMN_1689646	FSD1	1.35E-01	9.09E-03	4.29E-02
ILMN_1794282	KCNMA1	1.35E-01	1.67E-03	1.01E-02
ILMN_1704101	HSF4	1.35E-01	1.37E-03	8.53E-03
ILMN_1797181	LOC93622	1.35E-01	7.62E-03	3.70E-02
ILMN_1676088	MSRB3	1.35E-01	1.91E-03	1.14E-02
ILMN_1758852	ENTPD7	1.34E-01	3.57E-03	1.94E-02
ILMN_1737631	PAQR6	1.34E-01	1.70E-03	1.02E-02
ILMN_1680683	BCR	1.34E-01	4.81E-03	2.51E-02
ILMN_3211302	LOC646909	1.34E-01	5.34E-03	2.74E-02
ILMN_1731596	AP3S2	1.34E-01	7.96E-03	3.84E-02
ILMN_2352326	COASY	1.34E-01	2.47E-03	1.42E-02
ILMN_2078334	CNOT10	1.34E-01	2.62E-03	1.49E-02
ILMN_1726181	C19orf52	1.34E-01	4.37E-04	3.22E-03
ILMN_1700024	UST	1.34E-01	3.92E-03	2.11E-02
ILMN_1750563	CERCAM	1.34E-01	1.97E-03	1.17E-02
ILMN_3239965	IDO1	1.34E-01	4.77E-04	3.47E-03
ILMN_1692674	NKD1	1.34E-01	7.10E-04	4.88E-03
ILMN_1715698	MGC71993	1.34E-01	1.05E-03	6.81E-03
ILMN_2276758	POFUT1	1.33E-01	7.85E-03	3.79E-02
ILMN_1659444	KIF1B	1.33E-01	6.47E-03	3.22E-02
ILMN_1697880	PLD2	1.33E-01	2.83E-04	2.22E-03
ILMN_2224031	CETN3	1.32E-01	9.48E-03	4.45E-02
ILMN_1698726	SLC25A27	1.32E-01	4.17E-03	2.22E-02
ILMN_1813338	LAG3	1.32E-01	1.07E-02	4.92E-02
ILMN_1733096	ANAPC2	1.32E-01	1.81E-03	1.08E-02
ILMN_1729167	EFTUD1	1.32E-01	9.83E-03	4.59E-02
ILMN_1757532	SNPH	1.32E-01	5.23E-03	2.69E-02
ILMN_2361427	PMS2L3	1.32E-01	4.14E-03	2.21E-02
ILMN_1694547	IFFO1	1.32E-01	9.63E-03	4.51E-02
ILMN_1799743	MYBPC2	1.32E-01	6.75E-04	4.68E-03
ILMN_1677122	FAM168B	1.32E-01	5.38E-03	2.76E-02
ILMN_1659000	XPNPEP3	1.32E-01	3.85E-03	2.07E-02
ILMN_1667034	PDPR	1.31E-01	1.06E-02	4.87E-02
ILMN_1688730	DNAJC18	1.31E-01	8.80E-03	4.18E-02
ILMN_1855869		1.31E-01	1.20E-03	7.64E-03
ILMN_1658261	MAPKBP1	1.31E-01	4.42E-03	2.33E-02
ILMN_1806015	LOC391045	1.31E-01	4.81E-04	3.49E-03
ILMN_2134855	LOC728758	1.31E-01	1.58E-03	9.66E-03
ILMN_1716651	RUNX2	1.30E-01	1.03E-02	4.78E-02
ILMN_2316083	DNAJC28	1.30E-01	5.24E-04	3.76E-03
ILMN_1812191	C12orf57	1.30E-01	3.54E-03	1.93E-02
ILMN_1669624	LINS1	1.30E-01	1.94E-04	1.59E-03
ILMN_1702383	CNGB1	1.30E-01	5.36E-03	2.75E-02

ILMN_1769478	LOC202051	1.30E-01	5.03E-03	2.61E-02
ILMN_1737993	ERBB3	1.30E-01	2.27E-04	1.82E-03
ILMN_1802524	ICAM5	1.30E-01	2.54E-03	1.45E-02
ILMN_1680380	ATXN2L	1.30E-01	7.44E-05	6.96E-04
ILMN_1700681	CD99L2	1.29E-01	2.02E-03	1.19E-02
ILMN_1762080	JMY	1.29E-01	3.26E-03	1.80E-02
ILMN_1745132	GDF11	1.29E-01	4.26E-03	2.27E-02
ILMN_3244855	LOC100134540	1.29E-01	8.16E-04	5.50E-03
ILMN_1714667	C20orf107	1.29E-01	4.86E-04	3.53E-03
ILMN_3191596	LOC100130008	1.29E-01	1.14E-03	7.30E-03
ILMN_3256801	C1orf203	1.29E-01	6.12E-03	3.07E-02
ILMN_1802669	PPP3CB	1.29E-01	2.20E-03	1.28E-02
ILMN_2408730	GCC2	1.29E-01	4.21E-03	2.24E-02
ILMN_2355586	ATRN	1.29E-01	6.36E-03	3.17E-02
ILMN_1733175	LOC647044	1.29E-01	9.75E-04	6.40E-03
ILMN_3200070	LOC643534	1.28E-01	3.89E-03	2.09E-02
ILMN_3238116	LOC200030	1.28E-01	5.96E-03	3.00E-02
ILMN_1730995	AFAP1L2	1.28E-01	1.43E-03	8.84E-03
ILMN_1709925	GPR155	1.28E-01	5.32E-03	2.73E-02
ILMN_1681721	OASL	1.28E-01	3.28E-03	1.81E-02
ILMN_1788005	PLEC1	1.28E-01	5.80E-03	2.94E-02
ILMN_1832155		1.28E-01	6.03E-03	3.04E-02
ILMN_3274586	LOC401817	1.28E-01	9.37E-04	6.19E-03
ILMN_1759828	HARBI1	1.28E-01	1.06E-03	6.87E-03
ILMN_1698395	GNB5	1.28E-01	1.07E-03	6.93E-03
ILMN_1749606	ZNF778	1.28E-01	8.89E-04	5.92E-03
ILMN_1812278	LY9	1.27E-01	1.61E-04	1.35E-03
ILMN_1719792	PHLDB2	1.27E-01	9.05E-03	4.28E-02
ILMN_1743803	LOC653291	1.27E-01	2.27E-03	1.32E-02
ILMN_1746031	RIMS4	1.27E-01	7.17E-04	4.92E-03
ILMN_1682357	LOC646130	1.27E-01	1.10E-04	9.76E-04
ILMN_2200562	LOC595101	1.27E-01	7.70E-03	3.73E-02
ILMN_1769277	LOC651436	1.27E-01	9.32E-03	4.38E-02
ILMN_1714952	ZNF703	1.27E-01	2.66E-04	2.10E-03
ILMN_3227119	MGC23284	1.27E-01	2.47E-03	1.42E-02
ILMN_1735488	FRRS1	1.27E-01	5.84E-04	4.13E-03
ILMN_1657857	TMEM14C	1.27E-01	2.95E-03	1.65E-02
ILMN_2120965	NPAT	1.27E-01	4.62E-03	2.42E-02
ILMN_1719344	LOC730820	1.27E-01	8.97E-03	4.24E-02
ILMN_1758548	NEK7	1.27E-01	7.18E-04	4.93E-03
ILMN_1797236	TGM2	1.26E-01	1.72E-03	1.03E-02
ILMN_1757837	C6orf165	1.26E-01	1.66E-04	1.38E-03
ILMN_3203507	LOC100131888	1.26E-01	2.83E-03	1.59E-02
ILMN_1685097	ASCC1	1.26E-01	1.65E-04	1.38E-03
ILMN_1733674	LOC653103	1.25E-01	9.86E-03	4.59E-02
ILMN_1780357	PRRT1	1.25E-01	6.54E-03	3.25E-02

ILMN_2083333	PMS2L5	1.25E-01	2.54E-03	1.46E-02
ILMN_1658271	LSS	1.25E-01	1.24E-03	7.84E-03
ILMN_1721338	C22orf23	1.25E-01	1.55E-03	9.49E-03
ILMN_1761031	PTPDC1	1.25E-01	5.38E-03	2.76E-02
ILMN_1713918	CYTH3	1.25E-01	1.07E-02	4.91E-02
ILMN_2381603	ING3	1.25E-01	1.41E-04	1.21E-03
ILMN_3307901	GAN	1.25E-01	8.04E-03	3.87E-02
ILMN_1701487	TAOK2	1.25E-01	1.80E-03	1.08E-02
ILMN_1758025	LOC645659	1.24E-01	1.19E-03	7.56E-03
ILMN_1656933	NKPD1	1.24E-01	5.00E-03	2.59E-02
ILMN_2323848	PARD6A	1.24E-01	9.64E-03	4.51E-02
ILMN_3201900	LOC728147	1.24E-01	2.69E-03	1.53E-02
ILMN_1813955	SERTAD3	1.23E-01	9.09E-03	4.29E-02
ILMN_2090351	OR7E156P	1.23E-01	7.20E-05	6.78E-04
ILMN_1750079	PURB	1.23E-01	5.03E-03	2.61E-02
ILMN_3247088	TMEM151B	1.23E-01	1.67E-03	1.01E-02
ILMN_1724532	C11orf49	1.23E-01	1.16E-03	7.42E-03
ILMN_2108493	TMEM120B	1.23E-01	3.70E-04	2.79E-03
ILMN_3307602	EPHA10	1.23E-01	4.22E-03	2.24E-02
ILMN_1712088	CLYBL	1.23E-01	3.57E-03	1.94E-02
ILMN_1768399	ARFIP1	1.22E-01	1.14E-03	7.29E-03
ILMN_1732023	PRDM7	1.22E-01	4.03E-03	2.16E-02
ILMN_2366703	SGK3	1.22E-01	2.33E-03	1.35E-02
ILMN_1791838	LYG1	1.22E-01	1.94E-03	1.15E-02
ILMN_1655880	CHST11	1.22E-01	4.31E-03	2.29E-02
ILMN_2352881	NR6A1	1.22E-01	4.82E-03	2.51E-02
ILMN_3291352	LOC643778	1.22E-01	2.48E-03	1.43E-02
ILMN_1727138	PPM1B	1.22E-01	4.02E-03	2.15E-02
ILMN_1742929	HESX1	1.22E-01	2.48E-03	1.42E-02
ILMN_2367440	WDR17	1.22E-01	2.23E-03	1.30E-02
ILMN_1726624	YPEL4	1.22E-01	1.03E-04	9.16E-04
ILMN_2171783	CPEB3	1.22E-01	9.72E-03	4.54E-02
ILMN_2288402	HIP1R	1.21E-01	9.80E-03	4.57E-02
ILMN_1675674	UBE4B	1.21E-01	5.10E-03	2.64E-02
ILMN_1729117	COL5A2	1.21E-01	1.24E-03	7.82E-03
ILMN_1755909	C20orf11	1.21E-01	4.42E-03	2.33E-02
ILMN_1778213	STK33	1.21E-01	1.00E-04	8.98E-04
ILMN_1688154	MST1R	1.21E-01	1.22E-04	1.06E-03
ILMN_1687536	LOC646207	1.21E-01	8.71E-03	4.14E-02
ILMN_2384561	TJP2	1.21E-01	8.20E-03	3.93E-02
ILMN_2340877	MEN1	1.21E-01	9.12E-03	4.30E-02
ILMN_1669898	EGFL7	1.21E-01	1.08E-04	9.54E-04
ILMN_1725946	IRF6	1.20E-01	9.25E-03	4.35E-02
ILMN_1694711	MAD2L1BP	1.20E-01	7.32E-03	3.58E-02
ILMN_1672991	LOC648213	1.20E-01	2.91E-03	1.63E-02
ILMN_1786984	DCTN3	1.20E-01	1.59E-03	9.71E-03

ILMN_1655819	LOC728919	1.20E-01	5.21E-03	2.68E-02
ILMN_3298167	ZSWIM7	1.20E-01	5.94E-03	3.00E-02
ILMN_2403542	CATSPER2	1.20E-01	5.39E-03	2.76E-02
ILMN_1747146	TSG101	1.20E-01	6.91E-03	3.40E-02
ILMN_2285618	DIP2A	1.19E-01	8.71E-03	4.14E-02
ILMN_1721614	LOC442501	1.19E-01	6.40E-03	3.19E-02
ILMN_1765714	UBE2E3	1.19E-01	2.38E-03	1.38E-02
ILMN_3289655	LOC643960	1.19E-01	7.10E-03	3.48E-02
ILMN_1659099	ROCK2	1.19E-01	2.34E-03	1.36E-02
ILMN_1759652	C1orf61	1.19E-01	7.54E-04	5.13E-03
ILMN_1791467	LOC440503	1.18E-01	3.92E-04	2.93E-03
ILMN_1803180	PRDX6	1.18E-01	5.78E-03	2.93E-02
ILMN_1652331	KCTD5	1.18E-01	1.22E-04	1.07E-03
ILMN_1652940	MGC16703	1.18E-01	1.86E-04	1.54E-03
ILMN_2225718	CENPE	1.18E-01	7.15E-03	3.50E-02
ILMN_1793006	TAC3	1.18E-01	1.39E-03	8.65E-03
ILMN_3241237	LOC100132503	1.18E-01	3.26E-03	1.80E-02
ILMN_1696827	PARS2	1.18E-01	6.13E-03	3.08E-02
ILMN_1713013	PLD3	1.18E-01	4.19E-03	2.23E-02
ILMN_3199634	LOC645321	1.18E-01	5.45E-03	2.78E-02
ILMN_2106573	DENND2C	1.18E-01	2.36E-03	1.37E-02
ILMN_1753287	DLL3	1.17E-01	5.86E-03	2.96E-02
ILMN_2408663	RHCE	1.17E-01	4.58E-03	2.41E-02
ILMN_3183760	LOC100130736	1.17E-01	9.27E-03	4.37E-02
ILMN_3240715	LOC100133191	1.16E-01	8.41E-03	4.02E-02
ILMN_2336728	DLG3	1.16E-01	1.01E-02	4.70E-02
ILMN_3279927	LOC100131675	1.16E-01	8.71E-03	4.14E-02
ILMN_2174341	GPR83	1.16E-01	4.24E-04	3.14E-03
ILMN_1913498		1.16E-01	4.75E-03	2.48E-02
ILMN_3291053	LOC346085	1.16E-01	4.60E-03	2.42E-02
ILMN_1722387	C9orf126	1.16E-01	3.82E-03	2.06E-02
ILMN_1653856	STS-1	1.16E-01	6.16E-04	4.33E-03
ILMN_2385672	ELN	1.16E-01	6.01E-03	3.03E-02
ILMN_1655201	C21orf70	1.15E-01	2.69E-03	1.53E-02
ILMN_1654630	FAM193B	1.15E-01	2.09E-03	1.23E-02
ILMN_1698706	NID2	1.15E-01	8.78E-03	4.17E-02
ILMN_1751444	NCAPG	1.15E-01	9.53E-03	4.47E-02
ILMN_1790801	RPS6KA2	1.15E-01	5.06E-03	2.62E-02
ILMN_1897187		1.15E-01	1.37E-03	8.55E-03
ILMN_1711909	EDEM2	1.15E-01	8.17E-04	5.50E-03
ILMN_1870940		1.15E-01	6.91E-03	3.40E-02
ILMN_1743847	ACAP3	1.15E-01	7.87E-04	5.33E-03
ILMN_3223798	ZNF84	1.14E-01	2.73E-03	1.55E-02
ILMN_3298037	LOC728453	1.14E-01	2.22E-03	1.30E-02
ILMN_1718900	KCTD2	1.14E-01	5.90E-03	2.98E-02
ILMN_2075436	PDIA3P	1.14E-01	2.83E-03	1.59E-02

ILMN_2329171	SPDYE1	1.14E-01	1.17E-03	7.45E-03
ILMN_1730325	FLAD1	1.14E-01	3.06E-03	1.70E-02
ILMN_1720053	ZFAND3	1.14E-01	7.79E-03	3.77E-02
ILMN_3240267	LOC728323	1.14E-01	1.91E-03	1.13E-02
ILMN_3251516	KIAA1609	1.13E-01	3.26E-03	1.80E-02
ILMN_1717010	C2orf86	1.13E-01	1.04E-02	4.81E-02
ILMN_1721391	ATP6V0B	1.13E-01	4.17E-03	2.22E-02
ILMN_1656840	VPS13D	1.13E-01	2.47E-03	1.42E-02
ILMN_3237035	SNORA54	1.13E-01	3.16E-03	1.75E-02
ILMN_1674231	CHAF1B	1.13E-01	4.56E-03	2.40E-02
ILMN_1805932	C6orf163	1.13E-01	2.63E-03	1.50E-02
ILMN_1660292	MRPS21	1.13E-01	8.93E-03	4.23E-02
ILMN_1686889	C6orf225	1.13E-01	1.43E-03	8.82E-03
ILMN_1748907	UBQLNL	1.13E-01	7.00E-03	3.44E-02
ILMN_1779997	MEIS3	1.12E-01	2.17E-03	1.27E-02
ILMN_1758597	NAGS	1.12E-01	8.59E-03	4.09E-02
ILMN_1658483	IL1A	1.12E-01	8.15E-03	3.91E-02
ILMN_1675937	ANKRD9	1.12E-01	1.65E-03	1.00E-02
ILMN_1653278	MUC20	1.12E-01	7.05E-04	4.85E-03
ILMN_2407605	GIYD2	1.12E-01	3.79E-03	2.04E-02
ILMN_1797055	LIN52	1.11E-01	5.21E-03	2.68E-02
ILMN_3247844	C4orf10	1.11E-01	1.71E-03	1.03E-02
ILMN_1709607	FLJ44881	1.11E-01	4.14E-03	2.21E-02
ILMN_1661055	LOC440080	1.11E-01	4.32E-03	2.29E-02
ILMN_1690392	COMMD3	1.11E-01	8.42E-03	4.02E-02
ILMN_1651238	TRPV1	1.10E-01	2.91E-03	1.63E-02
ILMN_2280135	P4HA2	1.10E-01	1.18E-03	7.55E-03
ILMN_1763231	TTBK1	1.10E-01	4.49E-03	2.36E-02
ILMN_1765641	SEMA3A	1.10E-01	9.05E-04	6.01E-03
ILMN_1764364	FAM19A3	1.10E-01	1.01E-02	4.69E-02
ILMN_1719864	PACS2	1.10E-01	8.57E-03	4.08E-02
ILMN_1651232	LOC653113	1.09E-01	9.56E-03	4.48E-02
ILMN_2307032	OSBPL5	1.09E-01	8.91E-03	4.22E-02
ILMN_3234888	NCRNA00181	1.09E-01	2.64E-03	1.51E-02
ILMN_1805725	B4GALNT1	1.09E-01	6.98E-03	3.43E-02
ILMN_1805200	DNM1	1.09E-01	8.49E-04	5.68E-03
ILMN_1762167	GTDC1	1.09E-01	6.52E-03	3.24E-02
ILMN_1659110	MGC40489	1.09E-01	3.79E-04	2.85E-03
ILMN_2388800	PPAP2B	1.08E-01	4.45E-03	2.35E-02
ILMN_1676296	PPAP2A	1.08E-01	5.51E-03	2.81E-02
ILMN_1681325	LOC727825	1.08E-01	1.46E-03	9.01E-03
ILMN_3280126	LOC100131323	1.08E-01	8.98E-03	4.25E-02
ILMN_1752269	ACSS1	1.08E-01	9.17E-03	4.32E-02
ILMN_1668714	SIK2	1.08E-01	1.50E-03	9.18E-03
ILMN_2140342	CCDC57	1.08E-01	4.98E-03	2.58E-02
ILMN_1715672	HPCAL1	1.08E-01	1.82E-03	1.09E-02

ILMN_1690035	FLYWCH1	1.08E-01	5.34E-04	3.83E-03
ILMN_1803810	RRBP1	1.08E-01	1.00E-02	4.65E-02
ILMN_1744129	DTX2	1.08E-01	7.97E-04	5.38E-03
ILMN_2165975	CES3	1.07E-01	1.01E-03	6.58E-03
ILMN_2056687	C17orf56	1.07E-01	4.33E-03	2.29E-02
ILMN_1795257	GPT	1.07E-01	5.91E-03	2.99E-02
ILMN_2307656	AGTRAP	1.07E-01	5.33E-03	2.74E-02
ILMN_1756358	FBXO36	1.07E-01	1.37E-03	8.52E-03
ILMN_1729077	WHDC1L2	1.07E-01	5.87E-03	2.97E-02
ILMN_1740572	TCN2	1.07E-01	1.15E-03	7.38E-03
ILMN_1777660	RNF144	1.07E-01	5.33E-03	2.73E-02
ILMN_3205604	LOC641808	1.07E-01	1.40E-03	8.67E-03
ILMN_1887403	DBT	1.07E-01	7.26E-03	3.55E-02
ILMN_2395314	PRKACA	1.07E-01	1.35E-03	8.41E-03
ILMN_2162496	FLJ36166	1.06E-01	1.01E-02	4.70E-02
ILMN_1811973	PPARD	1.06E-01	2.01E-03	1.19E-02
ILMN_1756525	KLHL20	1.06E-01	2.76E-03	1.56E-02
ILMN_1740024	NAALAD2	1.05E-01	6.78E-03	3.35E-02
ILMN_1877156		1.05E-01	1.19E-03	7.59E-03
ILMN_1796082	DHX57	1.05E-01	5.29E-03	2.72E-02
ILMN_1660282	POPDC2	1.05E-01	4.66E-03	2.44E-02
ILMN_1720004	FLJ27354	1.04E-01	4.73E-03	2.47E-02
ILMN_1668683	MLL	1.04E-01	6.43E-03	3.20E-02
ILMN_1754562	ARHGEF17	1.04E-01	3.47E-03	1.90E-02
ILMN_1775569	CRISP2	1.04E-01	3.80E-03	2.05E-02
ILMN_1812327	RNF19A	1.04E-01	7.13E-03	3.50E-02
ILMN_3244769	BREA2	1.04E-01	3.16E-03	1.75E-02
ILMN_1704290	SPTLC2	1.04E-01	8.52E-03	4.06E-02
ILMN_1700633	ABHD4	1.03E-01	3.10E-03	1.73E-02
ILMN_1693338	CYP1B1	1.03E-01	1.66E-03	1.01E-02
ILMN_3299393	PNMAL1	1.03E-01	9.24E-03	4.35E-02
ILMN_2212909	MELK	1.03E-01	1.04E-02	4.81E-02
ILMN_2301677	MEIS3	1.03E-01	8.78E-03	4.17E-02
ILMN_1731777	NTRK1	1.02E-01	6.93E-03	3.41E-02
ILMN_1672591	SCN9A	1.02E-01	5.54E-03	2.83E-02
ILMN_1698731	SEC31B	1.02E-01	1.01E-03	6.59E-03
ILMN_1658206	C1orf102	1.02E-01	8.90E-03	4.22E-02
ILMN_1767523	IL17RB	1.01E-01	2.44E-03	1.41E-02
ILMN_1651428	SYTL2	1.01E-01	5.25E-03	2.70E-02
ILMN_1666022	TNFRSF10D	1.01E-01	3.85E-03	2.07E-02
ILMN_1673370	FBXL5	1.01E-01	4.75E-03	2.48E-02
ILMN_3199483	LOC100131064	1.01E-01	2.94E-03	1.65E-02
ILMN_1672925	LOC652837	1.00E-01	5.55E-03	2.83E-02
ILMN_3267438	LOC100130291	1.00E-01	1.88E-04	1.55E-03
ILMN_1801104	LOC642316	1.00E-01	5.89E-03	2.98E-02
ILMN_1655532	ASB6	1.00E-01	1.03E-03	6.71E-03

ILMN_1730077	RPUSD2	9.98E-02	2.92E-03	1.64E-02
ILMN_1672443	QDPR	9.93E-02	2.38E-03	1.38E-02
ILMN_1874806		9.93E-02	5.12E-03	2.65E-02
ILMN_1801341	DNHD2	9.90E-02	6.20E-03	3.10E-02
ILMN_3237808	LOC100133111	9.89E-02	6.78E-03	3.35E-02
ILMN_2384122	GPR56	9.82E-02	4.39E-03	2.32E-02
ILMN_1678827	POLR2J4	9.80E-02	1.06E-02	4.88E-02
ILMN_1769473	SETD2	9.80E-02	9.45E-03	4.44E-02
ILMN_1792270	C10orf46	9.78E-02	4.62E-03	2.42E-02
ILMN_2415235	CSNK1E	9.78E-02	9.08E-03	4.29E-02
ILMN_3200018	LOC442609	9.76E-02	9.24E-03	4.35E-02
ILMN_3273885	LOC100127983	9.75E-02	7.47E-03	3.64E-02
ILMN_1740276	CLDN9	9.72E-02	6.27E-03	3.14E-02
ILMN_1758619	SAG	9.72E-02	3.54E-03	1.93E-02
ILMN_1759117	XK	9.72E-02	7.92E-03	3.82E-02
ILMN_1670378	LOC653199	9.71E-02	3.01E-03	1.68E-02
ILMN_3241201	LOC100132967	9.70E-02	8.14E-03	3.91E-02
ILMN_1673969	LOC374491	9.67E-02	5.32E-03	2.73E-02
ILMN_1898004		9.63E-02	2.74E-03	1.55E-02
ILMN_1746871	TNRC6C	9.63E-02	7.74E-03	3.75E-02
ILMN_1842533		9.62E-02	9.47E-03	4.45E-02
ILMN_1803945	HCP5	9.61E-02	6.13E-03	3.08E-02
ILMN_1789166	SHD	9.61E-02	6.87E-03	3.39E-02
ILMN_1745862	LOC642169	9.58E-02	6.62E-03	3.28E-02
ILMN_1666560	IFT88	9.57E-02	9.89E-03	4.61E-02
ILMN_1808295	SMCR7	9.56E-02	6.48E-03	3.22E-02
ILMN_1667110	LOC642960	9.48E-02	6.88E-03	3.39E-02
ILMN_2102422	ABCB8	9.46E-02	5.59E-03	2.85E-02
ILMN_3240626	LOC100134424	9.43E-02	1.53E-03	9.36E-03
ILMN_1780840	C2orf16	9.41E-02	4.84E-03	2.52E-02
ILMN_1727001	DDX46	9.40E-02	5.10E-03	2.64E-02
ILMN_3245031	LOC100134550	9.37E-02	1.03E-03	6.71E-03
ILMN_1716924	LOC643482	9.37E-02	8.99E-03	4.25E-02
ILMN_1670452	ANKRD20A1	9.35E-02	2.72E-03	1.54E-02
ILMN_1678080	FKBP2	9.30E-02	1.06E-02	4.87E-02
ILMN_1730791	LOC646783	9.29E-02	6.42E-03	3.20E-02
ILMN_1713650	FLJ40113	9.28E-02	5.31E-03	2.73E-02
ILMN_1669557	AIM1L	9.23E-02	5.32E-03	2.73E-02
ILMN_1698085	C14orf21	9.21E-02	3.06E-03	1.71E-02
ILMN_1893788		9.18E-02	1.26E-03	7.95E-03
ILMN_3241292	LOC100134081	9.15E-02	1.04E-02	4.82E-02
ILMN_3284607	LOC728207	9.11E-02	1.01E-02	4.68E-02
ILMN_1718898	HOXC9	9.06E-02	6.53E-03	3.24E-02
ILMN_3241262	PABPC4L	9.04E-02	8.60E-03	4.10E-02
ILMN_1794239	C4orf26	8.95E-02	8.46E-03	4.04E-02
ILMN_1651745	TMEM25	8.92E-02	7.81E-03	3.78E-02

ILMN_3273383	LOC100127885	8.91E-02	9.44E-03	4.44E-02
ILMN_3243152	AKR7L	8.81E-02	1.01E-02	4.69E-02
ILMN_1808053	LOC550631	8.80E-02	8.17E-03	3.92E-02
ILMN_2357062	IL1RAP	8.79E-02	3.61E-03	1.96E-02
ILMN_3242174	LOC652900	8.79E-02	8.10E-03	3.90E-02
ILMN_1764456	TECPR2	8.78E-02	4.50E-03	2.37E-02
ILMN_1811437	C11orf9	8.76E-02	8.15E-03	3.91E-02
ILMN_1735765	RAB14	8.76E-02	9.68E-03	4.53E-02
ILMN_1793012	FLJ10803	8.74E-02	9.56E-03	4.48E-02
ILMN_1734736	VPS16	8.71E-02	7.55E-03	3.67E-02
ILMN_1720093	PCP2	8.65E-02	1.04E-02	4.82E-02
ILMN_2246134	PDE4DIP	8.63E-02	9.52E-04	6.27E-03
ILMN_2161285	FAM40B	8.62E-02	9.50E-03	4.46E-02
ILMN_2171588	SLC6A1	8.61E-02	7.64E-03	3.71E-02
ILMN_1694593	FAHD1	8.60E-02	7.36E-03	3.59E-02
ILMN_1713436	LOC648164	8.54E-02	4.88E-03	2.54E-02
ILMN_2364521	AXL	8.54E-02	3.29E-03	1.82E-02
ILMN_1678831	GCC2	8.51E-02	3.52E-03	1.92E-02
ILMN_1696415	LOC652076	8.50E-02	8.14E-03	3.91E-02
ILMN_1779979	SLC37A3	8.50E-02	6.17E-03	3.09E-02
ILMN_1652314	SPAG1	8.44E-02	9.53E-03	4.47E-02
ILMN_1686712	FLJ30092	8.42E-02	1.08E-02	4.96E-02
ILMN_1672307	BMX	8.42E-02	6.31E-03	3.15E-02
ILMN_1668617	AKR1C2	8.39E-02	1.61E-03	9.80E-03
ILMN_1694778	LOC646723	8.38E-02	8.92E-03	4.23E-02
ILMN_1761910	BDNF	8.33E-02	8.19E-03	3.93E-02
ILMN_1726266	ADAM12	8.32E-02	9.85E-03	4.59E-02
ILMN_1746837	UPP1	8.11E-02	3.01E-03	1.68E-02
ILMN_1721774	MPP7	8.09E-02	8.15E-03	3.91E-02
ILMN_3309709	PISRT1	8.04E-02	8.46E-03	4.04E-02
ILMN_1736007	A1BG	8.01E-02	9.88E-03	4.60E-02
ILMN_1806242	PSD	7.87E-02	3.79E-03	2.04E-02
ILMN_2046611	MCOLN3	7.82E-02	5.94E-03	3.00E-02
ILMN_2224231	TTTY21	7.79E-02	3.69E-03	2.00E-02
ILMN_1785919	LOC728229	7.76E-02	7.35E-03	3.59E-02
ILMN_3274563	LOC100132701	7.73E-02	6.18E-03	3.10E-02
ILMN_3247082	FAM150B	7.72E-02	2.30E-03	1.34E-02
ILMN_1788337	C19orf36	7.61E-02	2.84E-03	1.60E-02
ILMN_1791585	LOC653353	7.47E-02	8.39E-03	4.01E-02
ILMN_1702335	BRD8	7.42E-02	1.77E-03	1.06E-02
ILMN_3185313	LOC100128667	7.30E-02	1.07E-02	4.91E-02
ILMN_2293631	MGAT5B	7.27E-02	8.85E-03	4.20E-02
ILMN_1900604		7.23E-02	6.94E-03	3.42E-02
ILMN_1752952	MMP28	7.22E-02	7.56E-03	3.67E-02
ILMN_1686096	SPATA17	7.07E-02	1.01E-02	4.69E-02
ILMN_3237094	LOC100134252	6.97E-02	4.90E-03	2.55E-02

ILMN_3240182	UBTFL5	6.91E-02	6.17E-03	3.10E-02
ILMN_1657442	ADARB1	6.79E-02	9.86E-03	4.60E-02
ILMN_1809971	NBPF4	6.73E-02	6.44E-03	3.21E-02
ILMN_2094424	TNRC6C	6.64E-02	9.94E-03	4.63E-02
ILMN_1670807	FAM84B	6.41E-02	8.38E-03	4.00E-02
ILMN_1683487	ZNF154	6.40E-02	9.21E-03	4.34E-02
ILMN_1732806	LOC642849	6.36E-02	1.01E-02	4.68E-02
ILMN_1656364	ASB12	6.08E-02	4.59E-03	2.41E-02
ILMN_1702809	LOC388237	6.07E-02	1.03E-02	4.78E-02
ILMN_1734676	SLC5A12	6.04E-02	7.08E-03	3.47E-02
ILMN_1800169	LOC642176	5.76E-02	6.35E-03	3.17E-02
ILMN_1655167	ZNF502	5.63E-02	5.80E-03	2.94E-02
ILMN_1750497	GPR109A	5.57E-02	9.16E-03	4.32E-02
ILMN_2399016	MMP28	5.27E-02	1.03E-02	4.78E-02
ILMN_1680320	GBX2	-5.15E-02	8.10E-03	3.90E-02
ILMN_3294149	LOC727894	-5.34E-02	5.69E-03	2.89E-02
ILMN_1676307	FIGN	-5.78E-02	6.96E-03	3.42E-02
ILMN_1715584	SOCS5	-5.92E-02	3.47E-03	1.90E-02
ILMN_1654288	LOC652540	-6.08E-02	1.09E-02	4.99E-02
ILMN_1729368	FZD8	-6.15E-02	1.03E-02	4.75E-02
ILMN_1710734	GZMK	-6.22E-02	8.17E-03	3.92E-02
ILMN_1716814	PAIP1	-6.23E-02	1.01E-02	4.70E-02
ILMN_1702604	KCNA3	-6.26E-02	6.48E-03	3.22E-02
ILMN_1891229	LOC650879	-6.34E-02	1.04E-02	4.82E-02
ILMN_1746941	HIPK3	-6.42E-02	8.28E-03	3.96E-02
ILMN_1719831	C1orf213	-6.58E-02	7.48E-03	3.64E-02
ILMN_3228393	LOC728752	-6.63E-02	6.72E-03	3.33E-02
ILMN_3246087	LOC100134672	-6.68E-02	8.27E-03	3.96E-02
ILMN_1718539	SDHALP1	-6.71E-02	4.04E-03	2.16E-02
ILMN_1823477		-6.74E-02	9.62E-03	4.50E-02
ILMN_3254147	LOC100128657	-6.86E-02	8.96E-03	4.24E-02
ILMN_1676689	PPT2	-6.89E-02	4.62E-03	2.42E-02
ILMN_1738009	LOC401399	-6.95E-02	4.26E-03	2.26E-02
ILMN_1685761	WFDC6	-6.96E-02	6.38E-03	3.18E-02
ILMN_3276002	LOC441957	-7.03E-02	8.39E-03	4.01E-02
ILMN_3268238	LOC100128904	-7.13E-02	3.47E-03	1.90E-02
ILMN_1913786		-7.19E-02	9.84E-03	4.59E-02
ILMN_1684614	TNXB	-7.22E-02	6.45E-03	3.21E-02
ILMN_1667663	LOC650193	-7.26E-02	6.85E-03	3.38E-02
ILMN_1776915	LOC648846	-7.33E-02	6.07E-03	3.05E-02
ILMN_3245759	SNORD92	-7.38E-02	9.45E-03	4.44E-02
ILMN_1691861	FASTK	-7.39E-02	6.28E-03	3.14E-02
ILMN_2194678	LOC153328	-7.46E-02	6.83E-03	3.37E-02
ILMN_1683355	LRSAM1	-7.57E-02	5.90E-03	2.98E-02
ILMN_1744539	LOC727832	-7.61E-02	7.54E-03	3.66E-02
ILMN_1652660	LOC647357	-7.64E-02	7.95E-03	3.83E-02

ILMN_2381769	ATE1	-7.70E-02	3.79E-03	2.05E-02
ILMN_1692439	OR5L1	-7.71E-02	9.01E-03	4.26E-02
ILMN_1723094	LOC645070	-7.73E-02	4.23E-03	2.25E-02
ILMN_1814799	S100A13	-7.73E-02	8.52E-03	4.06E-02
ILMN_2123521	SYCP3	-7.74E-02	5.53E-03	2.82E-02
ILMN_3285346	LOC643387	-7.76E-02	9.01E-03	4.26E-02
ILMN_2278112	PRKACB	-7.76E-02	5.58E-03	2.84E-02
ILMN_1709065	LOC440918	-7.81E-02	6.00E-03	3.02E-02
ILMN_1762231	ZEB1	-7.84E-02	1.01E-02	4.67E-02
ILMN_1739025	LOC645367	-7.84E-02	5.27E-03	2.71E-02
ILMN_1777491	LOC646257	-7.91E-02	4.52E-03	2.38E-02
ILMN_2203858	OR10J1	-7.94E-02	1.02E-02	4.73E-02
ILMN_1760449	TSPAN32	-7.94E-02	4.35E-03	2.30E-02
ILMN_1784870	CES7	-7.96E-02	8.07E-03	3.88E-02
ILMN_2139061	COPS2	-7.99E-02	1.08E-02	4.95E-02
ILMN_3180722	FUSSEL18	-8.02E-02	4.51E-03	2.37E-02
ILMN_3291329	LOC389024	-8.03E-02	3.53E-03	1.93E-02
ILMN_1872462	KCNA1	-8.05E-02	2.45E-03	1.41E-02
ILMN_1705018	LOC648695	-8.11E-02	8.89E-03	4.22E-02
ILMN_2266228	VPS13D	-8.14E-02	8.49E-03	4.05E-02
ILMN_1738793	ZNF71	-8.14E-02	9.00E-03	4.25E-02
ILMN_1696214	NHEJ1	-8.17E-02	5.96E-03	3.01E-02
ILMN_1673705	LOC644668	-8.19E-02	1.07E-02	4.92E-02
ILMN_1656434	IP6K1	-8.21E-02	6.75E-03	3.34E-02
ILMN_3200438	LOC100132839	-8.27E-02	6.31E-03	3.15E-02
ILMN_2085012	TMEM176B	-8.29E-02	8.24E-03	3.95E-02
ILMN_1881472		-8.29E-02	3.09E-03	1.72E-02
ILMN_1652291	EVX2	-8.31E-02	1.31E-03	8.21E-03
ILMN_1890829		-8.32E-02	5.78E-03	2.93E-02
ILMN_1672102	PTPRB	-8.32E-02	3.54E-03	1.93E-02
ILMN_1804217	LOC650850	-8.33E-02	8.41E-03	4.02E-02
ILMN_1658016	ZNF831	-8.35E-02	4.48E-03	2.36E-02
ILMN_1716679	FAIM	-8.36E-02	1.47E-03	9.08E-03
ILMN_2409898	AFG3L1	-8.36E-02	6.57E-03	3.26E-02
ILMN_1709253	KCNK10	-8.38E-02	3.21E-03	1.78E-02
ILMN_1713613	PIAS2	-8.41E-02	6.74E-03	3.33E-02
ILMN_1794729	KRT20	-8.44E-02	5.99E-03	3.02E-02
ILMN_3236695	LOC100133747	-8.47E-02	3.21E-03	1.78E-02
ILMN_1713935	C3orf33	-8.48E-02	7.37E-03	3.60E-02
ILMN_1659878	PAK2	-8.48E-02	8.56E-03	4.08E-02
ILMN_1779835	ARL6IP4	-8.50E-02	1.51E-03	9.28E-03
ILMN_1898818		-8.50E-02	8.15E-03	3.91E-02
ILMN_1680887	LOC643550	-8.51E-02	1.84E-03	1.10E-02
ILMN_1673601	LRRC37B	-8.51E-02	4.31E-03	2.29E-02
ILMN_1843585		-8.53E-02	1.04E-02	4.81E-02
ILMN_1756908	LOC652119	-8.56E-02	7.99E-03	3.85E-02

ILMN_1682857	NDUFAF2	-8.57E-02	7.35E-03	3.59E-02
ILMN_1727360	MAOB	-8.58E-02	1.06E-02	4.88E-02
ILMN_1872864		-8.61E-02	1.04E-02	4.82E-02
ILMN_1804772	LOC643121	-8.64E-02	8.62E-03	4.10E-02
ILMN_1702140	RACGAP1	-8.66E-02	9.54E-03	4.47E-02
ILMN_1854201		-8.66E-02	1.12E-03	7.22E-03
ILMN_3285607	LOC100133695	-8.66E-02	1.09E-02	4.98E-02
ILMN_1743992	DCP1B	-8.68E-02	1.06E-02	4.86E-02
ILMN_1819939		-8.72E-02	3.61E-03	1.96E-02
ILMN_3307196	FLJ14107	-8.73E-02	9.94E-03	4.63E-02
ILMN_1658420	DSCAM	-8.75E-02	1.84E-03	1.10E-02
ILMN_3202942	LOC100131243	-8.75E-02	5.90E-03	2.98E-02
ILMN_2253286	PRKCZ	-8.80E-02	1.09E-03	7.03E-03
ILMN_1781984	LOC644719	-8.82E-02	6.19E-03	3.10E-02
ILMN_1660868	ZBTB8OS	-8.83E-02	6.08E-03	3.06E-02
ILMN_1815840	IGLL1	-8.88E-02	5.43E-03	2.78E-02
ILMN_1762670	C20orf70	-8.93E-02	6.49E-03	3.22E-02
ILMN_2077758	CTDSPL2	-8.93E-02	9.96E-03	4.63E-02
ILMN_1663165	LOC652808	-8.94E-02	3.88E-03	2.09E-02
ILMN_3244329	NCRNA00160	-8.97E-02	7.07E-03	3.47E-02
ILMN_2159290	LOC441376	-8.99E-02	3.04E-03	1.69E-02
ILMN_1692973	BICD1	-9.00E-02	7.61E-03	3.70E-02
ILMN_3249456	LOC728034	-9.01E-02	9.79E-03	4.57E-02
ILMN_1697445	LOC389748	-9.01E-02	1.25E-03	7.88E-03
ILMN_2229464	LRRC37A2	-9.01E-02	3.30E-03	1.82E-02
ILMN_1659851	LOC389832	-9.03E-02	7.05E-03	3.46E-02
ILMN_3247569	LOC729891	-9.06E-02	6.94E-03	3.42E-02
ILMN_1728055	ASTL	-9.10E-02	2.08E-03	1.22E-02
ILMN_2189314	HDGFL1	-9.10E-02	3.29E-03	1.81E-02
ILMN_1728887	PAK4	-9.11E-02	8.69E-03	4.13E-02
ILMN_1771043	CBFA2T3	-9.12E-02	1.12E-03	7.18E-03
ILMN_2408771	SPPL2B	-9.12E-02	8.94E-03	4.23E-02
ILMN_3256478	LOC100129034	-9.15E-02	1.08E-02	4.94E-02
ILMN_3246459	LOC100134383	-9.17E-02	7.52E-03	3.66E-02
ILMN_3272518	LOC100129169	-9.17E-02	5.71E-03	2.90E-02
ILMN_2284077	SLC38A8	-9.21E-02	5.79E-03	2.93E-02
ILMN_1700563	ZNF552	-9.22E-02	1.03E-02	4.76E-02
ILMN_3246902	ABCC6P1	-9.23E-02	7.66E-03	3.72E-02
ILMN_3235409	LOC100133585	-9.25E-02	2.69E-03	1.53E-02
ILMN_2055930	FARSB	-9.27E-02	1.00E-02	4.65E-02
ILMN_2062468	IGFBP7	-9.29E-02	5.16E-03	2.66E-02
ILMN_1653209	ZWINT	-9.29E-02	8.53E-03	4.07E-02
ILMN_3221888	NAP1L6	-9.29E-02	1.17E-03	7.48E-03
ILMN_2371911	MUC1	-9.30E-02	1.63E-03	9.91E-03
ILMN_1782730	ZNF473	-9.33E-02	2.76E-03	1.56E-02
ILMN_1685446	NARG1L	-9.36E-02	7.02E-03	3.45E-02

ILMN_1697121	LOC388526	-9.37E-02	4.83E-03	2.52E-02
ILMN_1815933	FTSJ2	-9.38E-02	2.15E-03	1.26E-02
ILMN_1803279	TMED5	-9.40E-02	1.09E-02	4.99E-02
ILMN_1728528	ZNF655	-9.44E-02	6.93E-03	3.41E-02
ILMN_1707002	TMLHE	-9.45E-02	8.87E-04	5.91E-03
ILMN_1682724	FANCF	-9.46E-02	6.27E-03	3.14E-02
ILMN_1689528	ZNF136	-9.46E-02	9.64E-03	4.51E-02
ILMN_2195015	MRGPRE	-9.47E-02	8.56E-03	4.08E-02
ILMN_3281517	LOC100132771	-9.48E-02	6.94E-04	4.79E-03
ILMN_1805766	POU6F1	-9.50E-02	1.07E-02	4.93E-02
ILMN_3247499	LOC732443	-9.52E-02	1.95E-03	1.16E-02
ILMN_1670816	MZF1	-9.53E-02	7.49E-03	3.65E-02
ILMN_1873536		-9.55E-02	4.61E-03	2.42E-02
ILMN_1697228	GPR56	-9.57E-02	4.09E-03	2.19E-02
ILMN_3280667	LOC644619	-9.59E-02	7.50E-03	3.65E-02
ILMN_1661359	CXorf65	-9.59E-02	1.00E-02	4.65E-02
ILMN_1668825	DKFZp686I15217	-9.60E-02	1.84E-03	1.10E-02
ILMN_1723320	FLJ37307	-9.61E-02	9.31E-03	4.38E-02
ILMN_1852349		-9.63E-02	1.01E-02	4.69E-02
ILMN_3242357	LOC100132418	-9.64E-02	6.24E-03	3.12E-02
ILMN_1838575		-9.64E-02	3.87E-03	2.08E-02
ILMN_2332440	KCNMB2	-9.65E-02	5.84E-03	2.95E-02
ILMN_1689623	LOC653765	-9.65E-02	8.78E-03	4.17E-02
ILMN_3248078	FAM27A	-9.66E-02	9.53E-03	4.47E-02
ILMN_1679678	FSD1L	-9.66E-02	6.19E-03	3.10E-02
ILMN_1753482	FAM86C	-9.67E-02	7.13E-03	3.50E-02
ILMN_2391458	ACSL4	-9.67E-02	8.84E-04	5.89E-03
ILMN_2387742	HNRNPA1L2	-9.75E-02	7.12E-04	4.89E-03
ILMN_1654689	ZNF197	-9.75E-02	3.39E-03	1.86E-02
ILMN_1803911	IIP45	-9.76E-02	5.49E-03	2.80E-02
ILMN_3211615	LOC728262	-9.77E-02	2.15E-03	1.26E-02
ILMN_1743086	UBE2Z	-9.79E-02	4.14E-03	2.21E-02
ILMN_1664124	FLJ13224	-9.79E-02	8.72E-03	4.14E-02
ILMN_1670523	SAPS2	-9.79E-02	4.22E-03	2.24E-02
ILMN_1734740	GPR65	-9.81E-02	9.69E-03	4.53E-02
ILMN_1853631		-9.84E-02	3.17E-03	1.76E-02
ILMN_1690365	USP41	-9.87E-02	4.08E-03	2.18E-02
ILMN_3309854	SNORD113-9	-9.87E-02	9.10E-03	4.30E-02
ILMN_2295879	NGDN	-9.87E-02	3.95E-03	2.12E-02
ILMN_1784070	LOC644681	-9.87E-02	8.00E-03	3.85E-02
ILMN_3308738	MIR204	-9.88E-02	4.01E-03	2.15E-02
ILMN_1744862	TGFBR2	-9.88E-02	9.06E-03	4.28E-02
ILMN_1679381	LOC286310	-9.91E-02	8.01E-03	3.86E-02
ILMN_1767337	SFXN5	-9.91E-02	3.51E-03	1.92E-02
ILMN_1704797	CLEC10A	-9.94E-02	8.31E-03	3.98E-02
ILMN_2373335	LIG3	-9.94E-02	1.05E-03	6.81E-03

ILMN_1712481	LOC650253	-9.94E-02	3.29E-03	1.82E-02
ILMN_1705928	SNRNP200	-9.95E-02	1.04E-02	4.82E-02
ILMN_2043845	IMPACT	-9.98E-02	3.30E-04	2.53E-03
ILMN_1654945	DNMT3A	-9.99E-02	8.29E-03	3.97E-02
ILMN_1682407	CYB5R2	-1.00E-01	7.69E-03	3.73E-02
ILMN_1826462		-1.00E-01	9.68E-03	4.52E-02
ILMN_1771410	LOC653346	-1.00E-01	1.04E-03	6.78E-03
ILMN_1655613	GSTA2	-1.01E-01	1.08E-02	4.94E-02
ILMN_3310875	MIR708	-1.01E-01	7.16E-03	3.51E-02
ILMN_1898691		-1.01E-01	9.53E-03	4.47E-02
ILMN_1880195		-1.01E-01	6.92E-03	3.41E-02
ILMN_1774585	CASP4	-1.01E-01	1.16E-03	7.41E-03
ILMN_1657634	FANCD2	-1.01E-01	1.06E-02	4.87E-02
ILMN_2188959	ACOT2	-1.01E-01	4.15E-03	2.21E-02
ILMN_1904301		-1.01E-01	9.48E-03	4.45E-02
ILMN_3248122	MED1	-1.01E-01	4.83E-03	2.52E-02
ILMN_1824337	ACVR2B	-1.01E-01	6.73E-03	3.33E-02
ILMN_2381559	ASTN2	-1.02E-01	8.15E-03	3.91E-02
ILMN_2393254	CAPNS1	-1.02E-01	5.56E-03	2.83E-02
ILMN_1707791	TAZ	-1.02E-01	7.52E-03	3.66E-02
ILMN_1880446		-1.02E-01	4.82E-03	2.51E-02
ILMN_1711599	LOC642573	-1.02E-01	1.94E-03	1.15E-02
ILMN_1715404	GPR174	-1.02E-01	5.71E-03	2.90E-02
ILMN_1765257	CINP	-1.03E-01	2.74E-03	1.55E-02
ILMN_2353027	GRAP	-1.03E-01	6.15E-03	3.09E-02
ILMN_2174045	RNF126P1	-1.03E-01	5.82E-03	2.94E-02
ILMN_1654402	IL18BP	-1.03E-01	8.69E-03	4.13E-02
ILMN_1768551	LOC197135	-1.03E-01	6.15E-03	3.09E-02
ILMN_1739582	HOXA9	-1.04E-01	3.09E-03	1.72E-02
ILMN_3209232	LOC644496	-1.04E-01	6.54E-03	3.25E-02
ILMN_2078141	ZNF587	-1.04E-01	5.36E-03	2.75E-02
ILMN_1783969	FAM24A	-1.04E-01	9.49E-03	4.45E-02
ILMN_1714014	LOC644491	-1.05E-01	3.36E-03	1.85E-02
ILMN_1860487		-1.05E-01	1.01E-02	4.70E-02
ILMN_1688728	TRMT2A	-1.05E-01	3.29E-03	1.82E-02
ILMN_1733859	DCAF15	-1.05E-01	1.06E-02	4.90E-02
ILMN_1753925	LOC645609	-1.05E-01	4.09E-04	3.05E-03
ILMN_1654250	MRPL21	-1.05E-01	1.27E-03	7.97E-03
ILMN_1661636	ZMYM2	-1.05E-01	2.62E-03	1.50E-02
ILMN_1788024	PCID2	-1.06E-01	1.22E-03	7.75E-03
ILMN_1786734	EIF5	-1.06E-01	4.94E-03	2.57E-02
ILMN_1662747	EVI2A	-1.06E-01	3.89E-04	2.91E-03
ILMN_1774802	LOC646481	-1.06E-01	1.17E-03	7.48E-03
ILMN_2070013	SLC25A15	-1.06E-01	3.06E-03	1.71E-02
ILMN_1685318	EN2	-1.06E-01	2.80E-03	1.58E-02
ILMN_1690939	ECGF1	-1.06E-01	2.16E-03	1.27E-02

ILMN_3245897	LOC728190	-1.06E-01	9.32E-03	4.38E-02
ILMN_1809582	VPS13C	-1.06E-01	1.04E-02	4.82E-02
ILMN_3248008	LOC442308	-1.06E-01	3.09E-04	2.40E-03
ILMN_2218450	LSM1	-1.06E-01	8.76E-03	4.16E-02
ILMN_1809963	RSAD1	-1.06E-01	6.81E-03	3.37E-02
ILMN_2342250	ZXDA	-1.07E-01	1.72E-03	1.03E-02
ILMN_1885273		-1.07E-01	1.32E-03	8.26E-03
ILMN_1874557		-1.07E-01	1.04E-03	6.78E-03
ILMN_2366998	CHTF8	-1.07E-01	9.30E-03	4.38E-02
ILMN_2257015	AGER	-1.07E-01	2.99E-03	1.67E-02
ILMN_1786895	KATNAL1	-1.07E-01	9.21E-03	4.34E-02
ILMN_2235086	CCDC4	-1.07E-01	1.05E-02	4.86E-02
ILMN_3179844	LOC100129426	-1.07E-01	1.01E-02	4.68E-02
ILMN_1788078	ACOT7	-1.08E-01	7.34E-03	3.59E-02
ILMN_1750234	PRSS2	-1.08E-01	3.77E-04	2.83E-03
ILMN_1713031	PGCP	-1.08E-01	6.39E-03	3.18E-02
ILMN_1657286	LOC728734	-1.08E-01	2.50E-03	1.44E-02
ILMN_1766211	LOC440287	-1.09E-01	7.60E-03	3.69E-02
ILMN_1686862	HLX	-1.09E-01	9.81E-03	4.58E-02
ILMN_1708769	LOC652789	-1.09E-01	7.07E-03	3.47E-02
ILMN_3240280	SNORA56	-1.09E-01	9.99E-04	6.53E-03
ILMN_1823160		-1.09E-01	2.54E-03	1.46E-02
ILMN_3310663	MIR665	-1.09E-01	1.22E-03	7.74E-03
ILMN_2293692	CREBBP	-1.09E-01	7.95E-03	3.83E-02
ILMN_1736954	ZBTB7B	-1.09E-01	5.30E-03	2.73E-02
ILMN_1675687	LOC644701	-1.09E-01	8.81E-03	4.18E-02
ILMN_1676833	CYB5RL	-1.10E-01	2.45E-03	1.41E-02
ILMN_3239014	LOC100134598	-1.10E-01	2.28E-03	1.33E-02
ILMN_3202432	LOC644464	-1.10E-01	1.25E-03	7.90E-03
ILMN_1696468	LOC644544	-1.10E-01	1.51E-03	9.25E-03
ILMN_3199507	LOC283922	-1.10E-01	5.97E-03	3.01E-02
ILMN_1802963	LOC391157	-1.10E-01	1.87E-03	1.12E-02
ILMN_1756308	NAE1	-1.10E-01	7.56E-03	3.67E-02
ILMN_1806249	IL1RN	-1.10E-01	7.28E-04	4.99E-03
ILMN_2405031	TRIM24	-1.10E-01	5.44E-03	2.78E-02
ILMN_1668771	LOC642946	-1.10E-01	1.04E-02	4.81E-02
ILMN_1737935	MACF1	-1.10E-01	1.04E-02	4.81E-02
ILMN_2081645	RSPO2	-1.10E-01	7.80E-03	3.78E-02
ILMN_1782349	LOC648066	-1.10E-01	3.38E-03	1.85E-02
ILMN_3233135	FAM178A	-1.10E-01	2.44E-03	1.41E-02
ILMN_3245541	SNORD53	-1.10E-01	7.25E-03	3.55E-02
ILMN_2154671	COX6B1	-1.11E-01	7.35E-03	3.59E-02
ILMN_1762033	NAPEPLD	-1.11E-01	2.27E-03	1.32E-02
ILMN_1798952	KDELR3	-1.11E-01	7.30E-03	3.57E-02
ILMN_2387496	C1orf152	-1.11E-01	4.81E-03	2.51E-02
ILMN_1906300		-1.11E-01	4.50E-03	2.37E-02

ILMN_3300604	ZNF37B	-1.11E-01	9.44E-03	4.43E-02
ILMN_1801391	SKP2	-1.12E-01	1.00E-02	4.66E-02
ILMN_1793732	FARS2	-1.12E-01	4.83E-03	2.52E-02
ILMN_1670540	SMAD1	-1.12E-01	4.67E-03	2.45E-02
ILMN_3203098	LOC400836	-1.12E-01	5.86E-03	2.96E-02
ILMN_1685279	LOC375748	-1.12E-01	3.42E-03	1.88E-02
ILMN_3262015	FLJ14100	-1.12E-01	4.97E-03	2.58E-02
ILMN_1835443		-1.12E-01	7.02E-03	3.45E-02
ILMN_3177615	LOC100128816	-1.12E-01	2.71E-03	1.54E-02
ILMN_3245616	CWC22	-1.12E-01	7.11E-03	3.49E-02
ILMN_1686735	LOC654135	-1.12E-01	1.07E-02	4.93E-02
ILMN_2310019	ZNF565	-1.13E-01	8.64E-03	4.11E-02
ILMN_3208216	LOC100131940	-1.13E-01	3.32E-03	1.83E-02
ILMN_2052863	RNF5P1	-1.13E-01	6.21E-03	3.11E-02
ILMN_3240781	SNORD17	-1.13E-01	2.92E-03	1.64E-02
ILMN_2249769	PCDH11X	-1.13E-01	7.60E-03	3.69E-02
ILMN_1666490	DST	-1.13E-01	4.10E-03	2.19E-02
ILMN_1672604	LOC642869	-1.13E-01	4.76E-03	2.48E-02
ILMN_1799293	MINA	-1.13E-01	1.04E-02	4.82E-02
ILMN_3229083	LOC728467	-1.13E-01	1.07E-02	4.91E-02
ILMN_1699722	LOC646074	-1.13E-01	7.79E-03	3.77E-02
ILMN_1679693	TMEM87B	-1.13E-01	2.74E-03	1.55E-02
ILMN_3230572	NCRNA00152	-1.14E-01	4.99E-04	3.61E-03
ILMN_1693039	ZFPL1	-1.14E-01	8.22E-04	5.53E-03
ILMN_1831563		-1.14E-01	1.04E-02	4.80E-02
ILMN_1748488	TXNL4B	-1.14E-01	3.79E-03	2.05E-02
ILMN_1694798	C5orf28	-1.14E-01	2.68E-03	1.52E-02
ILMN_1879857		-1.14E-01	5.74E-04	4.07E-03
ILMN_1733288	C1RL	-1.14E-01	6.83E-03	3.37E-02
ILMN_3248811	SNORA27	-1.14E-01	5.38E-03	2.76E-02
ILMN_1727173	SMARCD1	-1.14E-01	9.76E-04	6.41E-03
ILMN_1813246	LOC728554	-1.14E-01	4.37E-03	2.31E-02
ILMN_1828197		-1.14E-01	1.90E-03	1.13E-02
ILMN_1715693	LOC440160	-1.14E-01	6.96E-03	3.43E-02
ILMN_1666610	NARG2	-1.14E-01	7.08E-03	3.47E-02
ILMN_2170643	FLJ45032	-1.15E-01	4.14E-03	2.21E-02
ILMN_1894983		-1.15E-01	2.77E-04	2.18E-03
ILMN_1663580	DPP6	-1.15E-01	5.49E-03	2.80E-02
ILMN_1781104	MAP2K7	-1.15E-01	5.08E-03	2.63E-02
ILMN_2336393	SMN2	-1.15E-01	2.23E-03	1.30E-02
ILMN_1781601	LOC653421	-1.15E-01	6.90E-04	4.77E-03
ILMN_1664098	FASTK	-1.15E-01	9.57E-03	4.48E-02
ILMN_1684582	LOC648689	-1.15E-01	6.26E-03	3.13E-02
ILMN_1694890	UCHL5IP	-1.15E-01	1.37E-03	8.51E-03
ILMN_1732336	RFC2	-1.15E-01	3.66E-03	1.99E-02
ILMN_2395913	ARHGAP11A	-1.15E-01	4.53E-03	2.38E-02

ILMN_1685136	LOC339047	-1.16E-01	6.00E-03	3.02E-02
ILMN_3280618	LOC728115	-1.16E-01	4.00E-03	2.15E-02
ILMN_1771093	ZNF414	-1.16E-01	9.79E-04	6.42E-03
ILMN_1753275	DENND1A	-1.16E-01	6.33E-03	3.16E-02
ILMN_1899404		-1.16E-01	6.73E-03	3.33E-02
ILMN_1777168	CSRP2BP	-1.16E-01	4.28E-03	2.27E-02
ILMN_1702759	TMX4	-1.16E-01	1.03E-02	4.78E-02
ILMN_1654331	HOXB4	-1.16E-01	4.77E-03	2.49E-02
ILMN_1846537		-1.17E-01	1.04E-02	4.82E-02
ILMN_1724479	NR2C2	-1.17E-01	6.75E-04	4.68E-03
ILMN_1711157	NOTCH4	-1.17E-01	2.81E-03	1.58E-02
ILMN_1698237	TRIM17	-1.17E-01	2.42E-04	1.93E-03
ILMN_1681203	TWF1	-1.17E-01	8.69E-03	4.13E-02
ILMN_1726591	CARD16	-1.18E-01	9.00E-03	4.25E-02
ILMN_2082489	PRMT7	-1.18E-01	1.49E-03	9.14E-03
ILMN_1787366	ZNF335	-1.18E-01	2.74E-03	1.55E-02
ILMN_1818218		-1.18E-01	3.63E-04	2.74E-03
ILMN_1677824	RAB4A	-1.18E-01	8.72E-03	4.15E-02
ILMN_2348146	ERCC8	-1.18E-01	2.86E-03	1.61E-02
ILMN_3205837	LOC727833	-1.18E-01	1.21E-03	7.67E-03
ILMN_1783131	SAMD13	-1.19E-01	1.12E-03	7.19E-03
ILMN_1793712	SCAMP3	-1.19E-01	5.64E-03	2.87E-02
ILMN_2058795	PGCP	-1.19E-01	5.60E-03	2.85E-02
ILMN_1711311	PODXL	-1.19E-01	7.40E-03	3.61E-02
ILMN_1699091	C14orf118	-1.19E-01	1.12E-03	7.21E-03
ILMN_1687392	NRK	-1.19E-01	1.53E-03	9.35E-03
ILMN_1833009		-1.19E-01	6.84E-03	3.38E-02
ILMN_1831098		-1.20E-01	2.80E-03	1.58E-02
ILMN_1910307		-1.20E-01	4.28E-03	2.27E-02
ILMN_2382431	ANKRD36	-1.20E-01	1.92E-03	1.14E-02
ILMN_1703989	ARFRP1	-1.20E-01	7.73E-03	3.75E-02
ILMN_2131381	PDE3B	-1.20E-01	7.29E-03	3.57E-02
ILMN_2381206	DHODH	-1.20E-01	3.06E-03	1.71E-02
ILMN_1670504	TMEM103	-1.20E-01	1.44E-03	8.90E-03
ILMN_1737168	THTPA	-1.20E-01	2.67E-03	1.52E-02
ILMN_3267739	LOC100130667	-1.20E-01	3.03E-03	1.69E-02
ILMN_1687149	BDP1	-1.21E-01	7.44E-03	3.62E-02
ILMN_2106994	RABIF	-1.21E-01	1.66E-03	1.01E-02
ILMN_1818562		-1.21E-01	9.04E-03	4.27E-02
ILMN_2165251	RGPD6	-1.21E-01	4.95E-03	2.57E-02
ILMN_1849430		-1.21E-01	4.12E-03	2.20E-02
ILMN_2094351	NUDT9P1	-1.21E-01	3.98E-03	2.14E-02
ILMN_1714499	LOC646920	-1.21E-01	6.26E-03	3.13E-02
ILMN_3301319	LOC729296	-1.21E-01	1.60E-03	9.72E-03
ILMN_1883239		-1.21E-01	8.07E-04	5.45E-03
ILMN_3240301	LOC652776	-1.21E-01	1.34E-03	8.38E-03

ILMN_2087941	ENTPD3	-1.21E-01	6.16E-04	4.33E-03
ILMN_1774086	CBX3	-1.21E-01	3.90E-03	2.10E-02
ILMN_1706265	TRIM61	-1.22E-01	1.13E-03	7.25E-03
ILMN_3237788	LOC728945	-1.22E-01	1.60E-03	9.74E-03
ILMN_1732921	ITGB8	-1.23E-01	4.29E-03	2.28E-02
ILMN_2344907	FBXO43	-1.23E-01	6.31E-03	3.15E-02
ILMN_1894034		-1.23E-01	7.73E-03	3.75E-02
ILMN_1756236	C1QL2	-1.23E-01	3.92E-03	2.11E-02
ILMN_1756211	MOV10L1	-1.23E-01	5.05E-03	2.61E-02
ILMN_1774839	RBM19	-1.23E-01	4.30E-03	2.28E-02
ILMN_1671642	CSNK1A1L	-1.24E-01	2.43E-03	1.40E-02
ILMN_1700627	LOC653800	-1.24E-01	8.59E-03	4.09E-02
ILMN_1684714	LOC641944	-1.24E-01	2.87E-03	1.61E-02
ILMN_1670256	PDK1	-1.24E-01	9.00E-04	5.98E-03
ILMN_1792860	MED22	-1.24E-01	5.78E-03	2.93E-02
ILMN_1778650	VILL	-1.24E-01	1.65E-03	1.00E-02
ILMN_1653601	LOC646144	-1.24E-01	5.95E-03	3.00E-02
ILMN_3235383	LOC388248	-1.24E-01	2.92E-03	1.64E-02
ILMN_1692267	B4GALT3	-1.25E-01	3.56E-03	1.94E-02
ILMN_1805131	C17orf90	-1.25E-01	2.75E-03	1.56E-02
ILMN_1713948	FPGS	-1.25E-01	4.15E-03	2.22E-02
ILMN_1679685	WDR38	-1.25E-01	9.63E-03	4.51E-02
ILMN_1846771		-1.25E-01	4.48E-03	2.36E-02
ILMN_1654452	LOC441378	-1.25E-01	2.21E-03	1.29E-02
ILMN_1692575	SFRS8	-1.25E-01	5.72E-03	2.90E-02
ILMN_1796141	PKP3	-1.25E-01	1.12E-03	7.20E-03
ILMN_1682588	LOC643461	-1.25E-01	3.21E-03	1.77E-02
ILMN_3183789	LOC100128525	-1.25E-01	3.35E-03	1.84E-02
ILMN_2265093	C14orf118	-1.25E-01	7.70E-03	3.73E-02
ILMN_1672390	ZAK	-1.25E-01	3.49E-03	1.90E-02
ILMN_1762639	MED11	-1.25E-01	3.64E-04	2.75E-03
ILMN_2251804	SMC4	-1.25E-01	2.77E-03	1.57E-02
ILMN_1774373	NIPAL2	-1.25E-01	8.19E-03	3.93E-02
ILMN_1738468	KEL	-1.26E-01	1.66E-03	1.01E-02
ILMN_1686194	SDCCAG10	-1.26E-01	2.50E-03	1.43E-02
ILMN_3244483	LOC100134571	-1.26E-01	7.34E-03	3.59E-02
ILMN_3290640	LOC345645	-1.26E-01	1.83E-03	1.09E-02
ILMN_2377025	TCOF1	-1.26E-01	6.12E-03	3.07E-02
ILMN_1706818	THADA	-1.26E-01	1.55E-03	9.48E-03
ILMN_1864813	LOC728081	-1.26E-01	2.02E-03	1.19E-02
ILMN_1713605	RPAP1	-1.26E-01	3.18E-03	1.76E-02
ILMN_3201556	LOC642546	-1.27E-01	3.35E-04	2.56E-03
ILMN_2396292	ZNF655	-1.27E-01	4.78E-03	2.49E-02
ILMN_1702715	MECP2	-1.27E-01	4.73E-03	2.47E-02
ILMN_2413259	SOCS4	-1.27E-01	9.46E-03	4.44E-02
ILMN_1770589	NDUFB4	-1.27E-01	2.31E-03	1.34E-02

ILMN_1718069	MIS12	-1.27E-01	1.21E-03	7.67E-03
ILMN_1749832	LOC653037	-1.27E-01	1.53E-04	1.29E-03
ILMN_1685824	B4GALT5	-1.28E-01	7.37E-03	3.60E-02
ILMN_3279322	LOC645174	-1.28E-01	5.54E-03	2.82E-02
ILMN_2355776	GIT2	-1.28E-01	6.69E-03	3.32E-02
ILMN_2258268	GLRX2	-1.28E-01	2.27E-03	1.32E-02
ILMN_1683575	TMLHE	-1.28E-01	5.28E-03	2.72E-02
ILMN_1792107	ERCC8	-1.28E-01	8.48E-03	4.05E-02
ILMN_3300198	LOC729580	-1.28E-01	1.00E-02	4.67E-02
ILMN_2044927	RNF5	-1.28E-01	7.92E-03	3.82E-02
ILMN_2268241	ABL1	-1.28E-01	1.25E-03	7.88E-03
ILMN_1751289	DERPC	-1.28E-01	5.21E-03	2.69E-02
ILMN_1784608	CLEC2B	-1.28E-01	1.42E-03	8.79E-03
ILMN_1687524	KBTBD4	-1.28E-01	5.05E-03	2.61E-02
ILMN_1671854	SEPT1	-1.28E-01	9.66E-03	4.52E-02
ILMN_1874933		-1.28E-01	1.09E-02	4.98E-02
ILMN_3258356	LOC100128888	-1.28E-01	7.23E-03	3.54E-02
ILMN_1688346	ZNF800	-1.29E-01	3.67E-04	2.77E-03
ILMN_3280926	LOC100131473	-1.29E-01	1.15E-03	7.34E-03
ILMN_3227768	LOC729669	-1.29E-01	8.24E-03	3.95E-02
ILMN_1701213	PIP4K2B	-1.29E-01	5.47E-03	2.80E-02
ILMN_1726952	FLJ12331	-1.29E-01	1.32E-03	8.24E-03
ILMN_1745785	NR2F2	-1.29E-01	2.18E-03	1.28E-02
ILMN_1731123	RNF7	-1.29E-01	7.72E-03	3.74E-02
ILMN_3251365	PGGT1B	-1.29E-01	4.36E-03	2.31E-02
ILMN_2124790	dJ222E13.2	-1.29E-01	9.57E-03	4.48E-02
ILMN_1741260	MDN1	-1.30E-01	2.72E-03	1.54E-02
ILMN_1750511	NT5C3L	-1.30E-01	6.31E-04	4.42E-03
ILMN_3194911	ATF6B	-1.30E-01	8.67E-03	4.13E-02
ILMN_2389347	NR3C1	-1.30E-01	4.25E-03	2.26E-02
ILMN_2058975	KIAA0776	-1.30E-01	2.73E-03	1.55E-02
ILMN_1745447	HIVEP2	-1.30E-01	3.41E-03	1.87E-02
ILMN_1766974	ZNF107	-1.30E-01	2.41E-03	1.39E-02
ILMN_3263694	LOC100128771	-1.30E-01	3.07E-03	1.71E-02
ILMN_1757084	LOC642443	-1.30E-01	4.61E-03	2.42E-02
ILMN_1676233	INTS4	-1.30E-01	2.62E-03	1.50E-02
ILMN_1765640	LOC642773	-1.30E-01	3.46E-03	1.90E-02
ILMN_1696311	IMPAD1	-1.30E-01	6.12E-03	3.07E-02
ILMN_1809751	EIF2C3	-1.30E-01	7.59E-04	5.16E-03
ILMN_2241953	PILRA	-1.31E-01	6.40E-03	3.19E-02
ILMN_1762098	ZNF747	-1.31E-01	6.63E-04	4.61E-03
ILMN_1660433	ARID1B	-1.31E-01	1.98E-03	1.17E-02
ILMN_1779480	KCMF1	-1.31E-01	5.86E-04	4.14E-03
ILMN_1706075	SF3B1	-1.31E-01	6.31E-03	3.15E-02
ILMN_3263099	LOC100129195	-1.31E-01	4.96E-03	2.58E-02
ILMN_2401033	GOSR1	-1.31E-01	1.86E-03	1.11E-02

ILMN_1774955	LOC440345	-1.31E-01	7.34E-03	3.58E-02
ILMN_1666482	SP2	-1.31E-01	6.23E-03	3.12E-02
ILMN_1777340	DDX6	-1.31E-01	1.19E-03	7.59E-03
ILMN_1730931	RUFY3	-1.31E-01	2.84E-04	2.23E-03
ILMN_2388254	GCET2	-1.31E-01	5.43E-03	2.78E-02
ILMN_1673395	PIGO	-1.32E-01	3.54E-03	1.93E-02
ILMN_1760049	SERGEF	-1.32E-01	2.98E-03	1.67E-02
ILMN_1760430	FLJ27465	-1.32E-01	8.38E-04	5.62E-03
ILMN_1679087	RABEP2	-1.32E-01	4.54E-04	3.32E-03
ILMN_1705477	CAMK1D	-1.32E-01	9.60E-03	4.49E-02
ILMN_3235597	LOC147727	-1.32E-01	3.44E-03	1.89E-02
ILMN_1715351	SCYE1	-1.32E-01	8.16E-03	3.92E-02
ILMN_1769751	PIGG	-1.33E-01	5.05E-03	2.62E-02
ILMN_1674975	MANEAL	-1.33E-01	2.78E-03	1.57E-02
ILMN_1672553	SLC43A3	-1.33E-01	2.96E-04	2.31E-03
ILMN_1805342	LOC652634	-1.33E-01	2.25E-03	1.31E-02
ILMN_1776153	AGGF1	-1.33E-01	8.77E-03	4.16E-02
ILMN_1722648	SF3B4	-1.33E-01	2.88E-03	1.62E-02
ILMN_2090786	GCSH	-1.33E-01	8.83E-03	4.19E-02
ILMN_3244738	JAKMIP3	-1.34E-01	3.16E-03	1.75E-02
ILMN_2370336	MS4A4A	-1.34E-01	4.64E-04	3.38E-03
ILMN_1658093	LOC652470	-1.34E-01	6.39E-04	4.46E-03
ILMN_2385373	DHRS4L1	-1.34E-01	3.09E-04	2.40E-03
ILMN_1788813	RASL10B	-1.35E-01	7.64E-03	3.71E-02
ILMN_2050112	LOC388524	-1.35E-01	6.51E-03	3.24E-02
ILMN_1765606	YAF2	-1.35E-01	9.66E-04	6.35E-03
ILMN_1652205	LOC645785	-1.35E-01	1.18E-03	7.50E-03
ILMN_1660844	INTS4	-1.35E-01	7.46E-03	3.63E-02
ILMN_3289659	LOC645548	-1.35E-01	2.30E-03	1.34E-02
ILMN_2276290	RALGPS2	-1.35E-01	2.75E-03	1.56E-02
ILMN_1748904	WTAP	-1.35E-01	1.36E-03	8.48E-03
ILMN_1811171	GPR132	-1.35E-01	9.29E-03	4.37E-02
ILMN_1774083	TRIAP1	-1.35E-01	1.66E-03	1.00E-02
ILMN_1690703	C21orf34	-1.35E-01	2.49E-03	1.43E-02
ILMN_1714093	RMND5A	-1.35E-01	3.57E-03	1.94E-02
ILMN_1791873	COG1	-1.35E-01	3.11E-03	1.73E-02
ILMN_1798372	ANXA2P3	-1.35E-01	3.58E-03	1.95E-02
ILMN_1787815	TRIB3	-1.36E-01	5.05E-03	2.62E-02
ILMN_1791332	ATP5O	-1.36E-01	2.03E-03	1.20E-02
ILMN_1667183	AIFM1	-1.36E-01	1.52E-04	1.29E-03
ILMN_3242223	LOC100132228	-1.36E-01	6.01E-05	5.79E-04
ILMN_1724207	IVD	-1.36E-01	3.48E-03	1.90E-02
ILMN_1772113	U2AF1	-1.36E-01	3.90E-03	2.10E-02
ILMN_1906397		-1.36E-01	9.93E-03	4.62E-02
ILMN_1714401	SNX27	-1.37E-01	3.47E-03	1.90E-02
ILMN_1810977	CDV3	-1.37E-01	9.30E-03	4.38E-02

ILMN_1671902	THUMPD3	-1.37E-01	6.06E-05	5.83E-04
ILMN_3298163	LOC729683	-1.37E-01	4.72E-03	2.47E-02
ILMN_2192394	ERCC4	-1.37E-01	4.42E-03	2.33E-02
ILMN_1850594		-1.37E-01	8.57E-03	4.08E-02
ILMN_1673175	TNFSF11	-1.38E-01	1.32E-03	8.29E-03
ILMN_1728471	ARFGEF1	-1.38E-01	9.37E-03	4.41E-02
ILMN_1689747	NOL8	-1.38E-01	8.76E-03	4.16E-02
ILMN_2380494	ANXA11	-1.38E-01	3.05E-03	1.70E-02
ILMN_1697919	WHSC1L1	-1.38E-01	1.68E-03	1.02E-02
ILMN_1788887	LOC338799	-1.38E-01	1.09E-02	4.99E-02
ILMN_1687567	CUX1	-1.38E-01	5.68E-04	4.03E-03
ILMN_2278819	PDE7A	-1.38E-01	6.54E-04	4.55E-03
ILMN_3235185	SNRNP200	-1.38E-01	1.06E-02	4.89E-02
ILMN_2324994	IKBIP	-1.38E-01	1.36E-03	8.48E-03
ILMN_2096012	UHMK1	-1.38E-01	3.85E-03	2.07E-02
ILMN_1693009	FGL2	-1.38E-01	7.41E-04	5.05E-03
ILMN_2197247	POLR3A	-1.38E-01	1.50E-03	9.22E-03
ILMN_2382758	CREB1	-1.38E-01	1.49E-03	9.15E-03
ILMN_1715760	LGALS9	-1.38E-01	2.30E-03	1.34E-02
ILMN_2325112	C22orf40	-1.39E-01	1.30E-03	8.13E-03
ILMN_3240092	MED17	-1.39E-01	1.43E-03	8.86E-03
ILMN_1684271	ACBD6	-1.39E-01	9.52E-05	8.59E-04
ILMN_2134176	CXorf15	-1.39E-01	6.57E-03	3.26E-02
ILMN_3242582	LOC652627	-1.39E-01	1.20E-03	7.60E-03
ILMN_2225698	NDUFA10	-1.39E-01	1.24E-03	7.86E-03
ILMN_1782034	ZXDA	-1.39E-01	2.81E-03	1.59E-02
ILMN_1805720	LOC644096	-1.39E-01	2.56E-03	1.47E-02
ILMN_1914073		-1.39E-01	5.15E-04	3.71E-03
ILMN_2217289	UBE2S	-1.39E-01	3.84E-04	2.88E-03
ILMN_1738517	FCRL4	-1.39E-01	3.33E-04	2.55E-03
ILMN_1759792	CLIP4	-1.39E-01	4.35E-03	2.30E-02
ILMN_2284794	PSMB8	-1.39E-01	4.74E-03	2.48E-02
ILMN_3235800	LOC100134528	-1.39E-01	3.28E-03	1.81E-02
ILMN_1751596	HIVEP3	-1.40E-01	2.06E-03	1.21E-02
ILMN_1872456		-1.40E-01	2.42E-03	1.40E-02
ILMN_2244484	CCBL2	-1.40E-01	5.77E-03	2.92E-02
ILMN_1789567	MAGED2	-1.40E-01	1.59E-03	9.72E-03
ILMN_1814153	LOC729486	-1.40E-01	1.27E-03	8.02E-03
ILMN_2383964	CDCA4	-1.40E-01	3.72E-03	2.01E-02
ILMN_2199298	C19orf20	-1.40E-01	2.38E-03	1.37E-02
ILMN_1742276	DKFZP564O0523	-1.40E-01	1.77E-03	1.06E-02
ILMN_1723607	GPHN	-1.40E-01	7.85E-03	3.79E-02
ILMN_2108339	THUMPD1	-1.40E-01	6.96E-03	3.42E-02
ILMN_1734472	PEBP4	-1.40E-01	3.42E-04	2.61E-03
ILMN_1733107	NOC4L	-1.41E-01	2.12E-03	1.25E-02
ILMN_3307762	SDHAP3	-1.41E-01	4.43E-03	2.34E-02

ILMN_1680129	NSUN2	-1.41E-01	3.76E-04	2.83E-03
ILMN_3243312	ZC3H12D	-1.41E-01	6.20E-04	4.35E-03
ILMN_3239735	WASH5P	-1.41E-01	2.24E-03	1.31E-02
ILMN_1742808	SF1	-1.41E-01	5.56E-04	3.96E-03
ILMN_1760335	ADPRHL1	-1.41E-01	4.25E-03	2.26E-02
ILMN_1673844	LOC650251	-1.41E-01	7.38E-03	3.60E-02
ILMN_3240002	SNORD12B	-1.41E-01	2.37E-03	1.37E-02
ILMN_1736974	SOX12	-1.41E-01	3.45E-04	2.63E-03
ILMN_2303170	MBNL3	-1.41E-01	3.54E-04	2.69E-03
ILMN_1858700		-1.42E-01	3.66E-03	1.98E-02
ILMN_2351029	MTMR2	-1.42E-01	4.19E-05	4.24E-04
ILMN_3249624	LOC641298	-1.42E-01	1.41E-03	8.74E-03
ILMN_2364131	TTPAL	-1.42E-01	5.16E-03	2.66E-02
ILMN_1736816	C13orf3	-1.42E-01	2.46E-03	1.41E-02
ILMN_3304421	LOC730974	-1.42E-01	6.85E-03	3.38E-02
ILMN_1656898	LOC653324	-1.42E-01	7.58E-03	3.68E-02
ILMN_1739532	DPRXP4	-1.42E-01	3.55E-04	2.69E-03
ILMN_1693717	RPH3AL	-1.42E-01	6.74E-03	3.34E-02
ILMN_1675398	ALDH1B1	-1.43E-01	2.12E-03	1.24E-02
ILMN_2278433	LOC285074	-1.43E-01	1.31E-03	8.23E-03
ILMN_1734895	SFT2D1	-1.43E-01	2.78E-04	2.18E-03
ILMN_1687353	CDK10	-1.43E-01	9.71E-04	6.38E-03
ILMN_1805024	ERBB2IP	-1.43E-01	4.68E-03	2.45E-02
ILMN_1784766	MCM3AP	-1.43E-01	2.74E-03	1.55E-02
ILMN_2211263	RFK	-1.43E-01	5.71E-03	2.90E-02
ILMN_1690880	CTAGE5	-1.43E-01	4.41E-03	2.33E-02
ILMN_1768392	LOC648059	-1.44E-01	5.22E-03	2.69E-02
ILMN_1765746	SFT2D3	-1.44E-01	6.80E-04	4.70E-03
ILMN_2384629	RGS12	-1.44E-01	6.01E-03	3.03E-02
ILMN_2337551	HIPK3	-1.44E-01	3.56E-04	2.70E-03
ILMN_1651507	LOC642732	-1.44E-01	7.51E-04	5.11E-03
ILMN_1795609	RAD51	-1.44E-01	9.00E-04	5.98E-03
ILMN_2251375	ZFP64	-1.44E-01	4.36E-04	3.21E-03
ILMN_1824297		-1.44E-01	4.96E-04	3.59E-03
ILMN_1686454	TIFA	-1.45E-01	9.95E-03	4.63E-02
ILMN_1680420	NUDT13	-1.45E-01	4.32E-03	2.29E-02
ILMN_3241729	EMX2OS	-1.45E-01	1.78E-03	1.07E-02
ILMN_3251733	C19orf43	-1.45E-01	4.91E-04	3.56E-03
ILMN_2230672	MRPL18	-1.45E-01	1.08E-02	4.94E-02
ILMN_2092232	TSR1	-1.45E-01	6.60E-03	3.27E-02
ILMN_1798189	COX7C	-1.45E-01	3.41E-03	1.87E-02
ILMN_2233552	POLD3	-1.46E-01	2.44E-03	1.41E-02
ILMN_1672811	C1orf159	-1.46E-01	5.09E-04	3.67E-03
ILMN_2332558	ARL5A	-1.46E-01	9.47E-03	4.45E-02
ILMN_1784651	NAGA	-1.46E-01	7.96E-03	3.84E-02
ILMN_1698369	LOC728127	-1.46E-01	1.70E-03	1.03E-02

ILMN_1767934	PCSK5	-1.46E-01	3.27E-03	1.80E-02
ILMN_1737849	LOC647363	-1.46E-01	8.56E-03	4.08E-02
ILMN_1772064	LOC54103	-1.46E-01	1.98E-04	1.62E-03
ILMN_1777971	RNF126P1	-1.46E-01	8.32E-03	3.98E-02
ILMN_1812479	ATE1	-1.47E-01	2.69E-03	1.53E-02
ILMN_1705141	CACYBP	-1.47E-01	5.20E-05	5.11E-04
ILMN_2064132	NANP	-1.47E-01	8.26E-03	3.96E-02
ILMN_1667561	IFRD1	-1.47E-01	4.00E-03	2.15E-02
ILMN_2100693	MAP2K4	-1.47E-01	1.25E-03	7.90E-03
ILMN_1708605	LOC652481	-1.47E-01	2.93E-03	1.64E-02
ILMN_3248614	LOC152217	-1.47E-01	2.98E-03	1.67E-02
ILMN_1715931	ISCA1	-1.47E-01	1.65E-04	1.38E-03
ILMN_2325338	APOL2	-1.47E-01	9.64E-04	6.34E-03
ILMN_2268990	DRD3	-1.48E-01	9.33E-03	4.39E-02
ILMN_1904853		-1.48E-01	1.17E-03	7.49E-03
ILMN_3253235	LOC100127915	-1.48E-01	5.95E-03	3.00E-02
ILMN_1894895		-1.48E-01	5.42E-03	2.77E-02
ILMN_3290497	LOC643863	-1.48E-01	8.67E-04	5.79E-03
ILMN_1658978	LOC650155	-1.48E-01	1.22E-03	7.73E-03
ILMN_1757129	TMEM88	-1.48E-01	2.75E-04	2.16E-03
ILMN_3276676	LOC392382	-1.48E-01	2.39E-03	1.38E-02
ILMN_1677200	CYFIP2	-1.48E-01	8.63E-04	5.77E-03
ILMN_1774826	FXN	-1.49E-01	5.76E-04	4.08E-03
ILMN_1678236	LOC643558	-1.49E-01	2.29E-04	1.84E-03
ILMN_2086064	SNRPC	-1.49E-01	4.54E-04	3.32E-03
ILMN_1776267	CLN6	-1.49E-01	3.93E-03	2.11E-02
ILMN_1765994	ZBP1	-1.49E-01	2.20E-04	1.77E-03
ILMN_1696670	LOC92497	-1.49E-01	3.19E-03	1.77E-02
ILMN_2379695	XRCC4	-1.49E-01	5.14E-04	3.70E-03
ILMN_3238797	FAM72A	-1.49E-01	1.20E-03	7.64E-03
ILMN_1695079	ZNF101	-1.49E-01	6.53E-03	3.24E-02
ILMN_2318685	ABHD12	-1.50E-01	7.63E-03	3.70E-02
ILMN_1743347	AKT2	-1.50E-01	1.49E-03	9.18E-03
ILMN_2169025	JOSD2	-1.50E-01	1.86E-05	2.13E-04
ILMN_1809344	BTBD10	-1.50E-01	1.44E-03	8.88E-03
ILMN_1665871	ANGEL1	-1.50E-01	6.78E-03	3.35E-02
ILMN_3204275	LOC100131859	-1.50E-01	6.35E-03	3.17E-02
ILMN_1675239	NDUFA7	-1.50E-01	1.53E-03	9.37E-03
ILMN_1777584	KARS	-1.50E-01	5.01E-03	2.60E-02
ILMN_3213261	LOC646330	-1.50E-01	1.76E-03	1.06E-02
ILMN_1659703	WWP2	-1.50E-01	5.52E-03	2.82E-02
ILMN_2115336	GNB3	-1.51E-01	4.31E-03	2.28E-02
ILMN_2143671	KPTN	-1.51E-01	1.89E-03	1.13E-02
ILMN_1669096	LOC641788	-1.51E-01	1.05E-04	9.36E-04
ILMN_3301343	LOC730883	-1.51E-01	9.42E-04	6.22E-03
ILMN_2223836	CHORDC1	-1.51E-01	1.02E-02	4.73E-02

ILMN_1753111	NAMPT	-1.51E-01	1.79E-03	1.07E-02
ILMN_1851547		-1.51E-01	7.14E-03	3.50E-02
ILMN_1722953	USP47	-1.52E-01	4.32E-04	3.19E-03
ILMN_1771870	FAM98C	-1.52E-01	9.70E-05	8.73E-04
ILMN_2394381	CLN3	-1.52E-01	5.64E-03	2.87E-02
ILMN_2385191	QRICH1	-1.52E-01	7.36E-04	5.03E-03
ILMN_1700432	ITPKB	-1.52E-01	2.97E-04	2.31E-03
ILMN_1790715	LOC653105	-1.52E-01	1.51E-03	9.23E-03
ILMN_2347789	C4BPB	-1.52E-01	1.78E-03	1.07E-02
ILMN_1703724	LOC649182	-1.52E-01	2.31E-04	1.85E-03
ILMN_2381121	UQCC	-1.52E-01	3.99E-03	2.14E-02
ILMN_1731941	APOM	-1.52E-01	6.78E-03	3.35E-02
ILMN_1863592		-1.52E-01	7.12E-04	4.89E-03
ILMN_3251227	TBC1D22A	-1.52E-01	9.58E-03	4.49E-02
ILMN_1784037	ZBTB40	-1.53E-01	5.56E-03	2.83E-02
ILMN_1720794	SNORD4B	-1.53E-01	4.09E-04	3.04E-03
ILMN_1891455		-1.53E-01	5.38E-03	2.76E-02
ILMN_1739257	EIF3E	-1.53E-01	3.13E-03	1.74E-02
ILMN_1812622	NEIL1	-1.53E-01	8.88E-03	4.21E-02
ILMN_1752895	STX8	-1.53E-01	3.35E-03	1.84E-02
ILMN_1690494	RPL6	-1.53E-01	3.60E-03	1.96E-02
ILMN_1671191	UQCRC1	-1.54E-01	2.26E-03	1.32E-02
ILMN_1907705		-1.54E-01	4.16E-03	2.22E-02
ILMN_1655052	TRNT1	-1.54E-01	1.58E-03	9.64E-03
ILMN_1651752	CXorf21	-1.54E-01	1.15E-03	7.34E-03
ILMN_3238001	LOC100132112	-1.54E-01	1.07E-03	6.91E-03
ILMN_1744442	TTPAL	-1.54E-01	6.35E-03	3.17E-02
ILMN_1755235	XPO6	-1.54E-01	1.04E-02	4.82E-02
ILMN_2394102	STRN4	-1.54E-01	6.04E-05	5.81E-04
ILMN_1692390	CCNT1	-1.54E-01	8.71E-03	4.14E-02
ILMN_1905094		-1.54E-01	2.47E-04	1.96E-03
ILMN_1691636	DAZL	-1.54E-01	1.07E-02	4.92E-02
ILMN_1904931		-1.54E-01	2.41E-03	1.39E-02
ILMN_1699682	LOC646431	-1.55E-01	2.13E-03	1.25E-02
ILMN_1805990	BAK1	-1.55E-01	5.69E-03	2.89E-02
ILMN_1656496	EPC2	-1.55E-01	3.94E-03	2.12E-02
ILMN_1662719	GPBP1L1	-1.55E-01	3.12E-03	1.73E-02
ILMN_3200465	LOC100132724	-1.55E-01	7.31E-03	3.57E-02
ILMN_1766346	ZNF512B	-1.55E-01	4.52E-03	2.38E-02
ILMN_1742827	EXOC4	-1.55E-01	8.09E-04	5.46E-03
ILMN_1695697	LOC650862	-1.55E-01	2.33E-03	1.35E-02
ILMN_1678312	LOC388397	-1.55E-01	5.69E-03	2.89E-02
ILMN_2044453	LPAR5	-1.55E-01	3.93E-04	2.94E-03
ILMN_1766902	LOC653904	-1.56E-01	2.47E-03	1.42E-02
ILMN_3246801	DNAJC30	-1.56E-01	2.75E-03	1.56E-02
ILMN_1787749	CASP8	-1.56E-01	1.58E-03	9.64E-03

ILMN_2177413	TBL3	-1.56E-01	4.29E-03	2.28E-02
ILMN_1733970	BAG1	-1.56E-01	2.56E-03	1.47E-02
ILMN_1835521		-1.56E-01	1.93E-05	2.20E-04
ILMN_1815079	TICAM1	-1.56E-01	1.50E-04	1.28E-03
ILMN_1663416	LOC642250	-1.56E-01	3.70E-03	2.01E-02
ILMN_1690909	LOC440498	-1.56E-01	1.07E-03	6.89E-03
ILMN_2329744	PMS2	-1.56E-01	1.05E-03	6.81E-03
ILMN_2110532	RPL26L1	-1.56E-01	8.45E-04	5.66E-03
ILMN_1777318	C9orf64	-1.56E-01	5.45E-03	2.78E-02
ILMN_1663664	MRPS10	-1.56E-01	1.03E-02	4.75E-02
ILMN_1670748	GALNT7	-1.56E-01	1.69E-03	1.02E-02
ILMN_1687921	JMJD8	-1.57E-01	2.25E-03	1.31E-02
ILMN_3238012	LOC100133033	-1.57E-01	7.54E-03	3.66E-02
ILMN_1741334	IKZF4	-1.57E-01	3.56E-04	2.70E-03
ILMN_1737574	LOC648605	-1.57E-01	6.29E-03	3.14E-02
ILMN_3240754	FAM90A3	-1.57E-01	6.18E-04	4.34E-03
ILMN_3302701	LOC729524	-1.57E-01	1.33E-03	8.30E-03
ILMN_2056795	USP9Y	-1.57E-01	1.33E-03	8.33E-03
ILMN_1824766		-1.57E-01	1.20E-04	1.05E-03
ILMN_2402972	GGA3	-1.57E-01	1.11E-03	7.16E-03
ILMN_1669206	CNOT1	-1.57E-01	4.56E-04	3.34E-03
ILMN_1671237	GNGT2	-1.57E-01	1.75E-03	1.05E-02
ILMN_2398274	PYCARD	-1.57E-01	1.95E-03	1.16E-02
ILMN_1915958		-1.57E-01	1.07E-02	4.90E-02
ILMN_1802951	CDCA1	-1.57E-01	1.21E-03	7.69E-03
ILMN_3310108	MIR571	-1.57E-01	5.27E-03	2.71E-02
ILMN_1722698	RCHY1	-1.57E-01	1.20E-03	7.60E-03
ILMN_1830069		-1.58E-01	1.54E-04	1.30E-03
ILMN_2307883	ATP5J2	-1.58E-01	2.19E-04	1.77E-03
ILMN_1772712	UBE2J2	-1.58E-01	9.33E-04	6.17E-03
ILMN_2350114	TRIM13	-1.58E-01	1.02E-03	6.64E-03
ILMN_3218538	LOC345645	-1.58E-01	1.54E-03	9.44E-03
ILMN_1732328	LOC646200	-1.58E-01	6.71E-03	3.32E-02
ILMN_1691487	TRAF2	-1.58E-01	1.86E-04	1.53E-03
ILMN_1806502	ZNF165	-1.58E-01	2.66E-03	1.52E-02
ILMN_3239298	OTUD7B	-1.58E-01	8.71E-04	5.82E-03
ILMN_3298544	LOC729157	-1.58E-01	4.93E-03	2.56E-02
ILMN_1658746	PTPLAD1	-1.58E-01	1.84E-03	1.10E-02
ILMN_1676385	PAK2	-1.59E-01	3.67E-03	1.99E-02
ILMN_1803510	LOC650803	-1.59E-01	1.02E-03	6.64E-03
ILMN_1683096	ASB1	-1.59E-01	2.66E-03	1.51E-02
ILMN_2042651	EVI2B	-1.59E-01	9.51E-04	6.27E-03
ILMN_3200565	LOC643015	-1.59E-01	2.73E-06	4.18E-05
ILMN_2333440	TM9SF1	-1.59E-01	5.55E-04	3.96E-03
ILMN_1679358	ZDHHC5	-1.59E-01	7.59E-03	3.69E-02
ILMN_1696375	TTC31	-1.59E-01	3.09E-03	1.72E-02

ILMN_1784465	FAM91A2	-1.59E-01	5.40E-03	2.77E-02
ILMN_1723815	NPEPPS	-1.59E-01	1.01E-03	6.60E-03
ILMN_1707943	C19orf39	-1.60E-01	1.37E-04	1.18E-03
ILMN_1652790	CLK1	-1.60E-01	3.30E-03	1.82E-02
ILMN_3266964	LOC100128191	-1.60E-01	1.02E-03	6.66E-03
ILMN_1692394	KIAA1024	-1.60E-01	4.58E-03	2.41E-02
ILMN_3230286	LOC731605	-1.60E-01	3.81E-03	2.05E-02
ILMN_3298124	LOC728816	-1.60E-01	8.37E-03	4.00E-02
ILMN_1807095	MRPS36	-1.60E-01	1.04E-02	4.81E-02
ILMN_2204754	TMX4	-1.60E-01	3.77E-03	2.04E-02
ILMN_2278518	NFATC2IP	-1.60E-01	1.32E-03	8.28E-03
ILMN_1766200	CALHM2	-1.60E-01	3.65E-03	1.98E-02
ILMN_1693822	LOC402562	-1.60E-01	3.61E-03	1.96E-02
ILMN_2365544	NHP2	-1.60E-01	1.30E-04	1.12E-03
ILMN_1747924	LOC644254	-1.61E-01	5.25E-04	3.77E-03
ILMN_3271711	LOC100128691	-1.61E-01	1.02E-02	4.73E-02
ILMN_1909247		-1.61E-01	4.23E-03	2.25E-02
ILMN_1810531	DRG2	-1.61E-01	2.78E-03	1.57E-02
ILMN_1673892	GK5	-1.61E-01	9.60E-05	8.65E-04
ILMN_3182275	FAM160B1	-1.61E-01	1.25E-04	1.09E-03
ILMN_1795344	GOLPH4	-1.61E-01	6.21E-03	3.11E-02
ILMN_1790918	ZNF236	-1.61E-01	6.85E-03	3.38E-02
ILMN_1801909	IGHMBP2	-1.61E-01	4.21E-03	2.24E-02
ILMN_1705677	C1orf220	-1.61E-01	5.14E-05	5.06E-04
ILMN_3302937	LOC729793	-1.62E-01	9.89E-05	8.88E-04
ILMN_1789186	OBFC1	-1.62E-01	4.16E-04	3.09E-03
ILMN_2415467	AP1GBP1	-1.62E-01	1.53E-03	9.36E-03
ILMN_1779279	C14orf32	-1.62E-01	7.22E-03	3.54E-02
ILMN_1697665	LOC653972	-1.62E-01	7.74E-03	3.75E-02
ILMN_1801605	BIRC6	-1.62E-01	1.24E-03	7.87E-03
ILMN_1659854	PRPF40A	-1.62E-01	3.74E-03	2.02E-02
ILMN_1742779	CENPL	-1.62E-01	3.93E-03	2.11E-02
ILMN_1675684	APOBEC3C	-1.62E-01	9.32E-04	6.17E-03
ILMN_1726913	RBPJ	-1.62E-01	6.76E-05	6.42E-04
ILMN_1702946	THUMPD1	-1.62E-01	1.13E-03	7.24E-03
ILMN_1761911	SCYL1BP1	-1.63E-01	7.90E-03	3.82E-02
ILMN_1891368		-1.63E-01	1.58E-03	9.66E-03
ILMN_3295847	LOC645387	-1.63E-01	1.15E-03	7.38E-03
ILMN_1744709	DLG5	-1.63E-01	4.93E-05	4.88E-04
ILMN_1764361	DUSP16	-1.63E-01	3.32E-03	1.83E-02
ILMN_1812934	DIDO1	-1.63E-01	1.73E-03	1.04E-02
ILMN_1741005	RG9MTD2	-1.63E-01	4.05E-04	3.02E-03
ILMN_1661860	ASPSCR1	-1.63E-01	5.04E-03	2.61E-02
ILMN_2156250	SOCS3	-1.63E-01	4.79E-03	2.50E-02
ILMN_3220861	LOC729952	-1.63E-01	3.53E-04	2.68E-03
ILMN_1775423	C10orf88	-1.63E-01	1.32E-03	8.24E-03

ILMN_1841620		-1.63E-01	5.98E-04	4.22E-03
ILMN_1764494	ATP5A1	-1.63E-01	1.71E-03	1.03E-02
ILMN_1803036	TARBP1	-1.63E-01	7.61E-03	3.69E-02
ILMN_1780824	PCTK2	-1.63E-01	1.54E-04	1.30E-03
ILMN_3291921	LOC645086	-1.64E-01	2.58E-03	1.47E-02
ILMN_1684289	PNPO	-1.64E-01	3.75E-04	2.82E-03
ILMN_1879879		-1.64E-01	1.74E-03	1.05E-02
ILMN_2121189	C1orf156	-1.64E-01	1.81E-03	1.08E-02
ILMN_1709795	RAC2	-1.64E-01	2.32E-04	1.86E-03
ILMN_1672393	CUL7	-1.64E-01	2.79E-04	2.19E-03
ILMN_2070044	PPM1K	-1.64E-01	1.54E-03	9.40E-03
ILMN_2398077	MEIS2	-1.64E-01	8.35E-04	5.61E-03
ILMN_1662263	MDP1	-1.64E-01	5.36E-03	2.75E-02
ILMN_1757272	THAP1	-1.65E-01	5.30E-04	3.80E-03
ILMN_2152402	BAT5	-1.65E-01	2.54E-04	2.01E-03
ILMN_1773505	USP4	-1.65E-01	2.23E-03	1.30E-02
ILMN_1798224	JARID1C	-1.65E-01	4.46E-03	2.35E-02
ILMN_1813833	NARFL	-1.65E-01	3.50E-04	2.66E-03
ILMN_2288784	CCDC34	-1.65E-01	4.01E-04	2.99E-03
ILMN_1806907	PAWR	-1.65E-01	8.40E-03	4.01E-02
ILMN_1777118	INTS9	-1.65E-01	9.26E-04	6.13E-03
ILMN_2368713	TMEM189-UBE2V1	-1.66E-01	2.93E-04	2.29E-03
ILMN_3266186	HDAC7	-1.66E-01	2.56E-05	2.80E-04
ILMN_1673024	RBM15B	-1.66E-01	2.02E-03	1.19E-02
ILMN_1726387	NF1	-1.66E-01	3.28E-03	1.81E-02
ILMN_1676458	MRPL11	-1.66E-01	1.05E-02	4.83E-02
ILMN_1770818	LOC642342	-1.66E-01	8.79E-04	5.86E-03
ILMN_3249366	JMJD8	-1.66E-01	2.73E-03	1.55E-02
ILMN_1839019		-1.66E-01	4.29E-04	3.17E-03
ILMN_3263007	LOC100129608	-1.66E-01	6.40E-04	4.47E-03
ILMN_1670124	LOC150223	-1.66E-01	5.08E-03	2.63E-02
ILMN_1823273		-1.67E-01	5.08E-06	7.12E-05
ILMN_1754532	LOC442517	-1.67E-01	9.57E-03	4.48E-02
ILMN_1703330	FEM1C	-1.67E-01	1.42E-03	8.79E-03
ILMN_1676285	DHX9	-1.67E-01	1.49E-03	9.18E-03
ILMN_1800465	LOC654042	-1.67E-01	4.75E-03	2.48E-02
ILMN_1791438	MBLAC1	-1.67E-01	6.57E-04	4.57E-03
ILMN_3240326	LOC732160	-1.68E-01	8.19E-03	3.93E-02
ILMN_1671048	ZNF644	-1.68E-01	4.58E-03	2.41E-02
ILMN_2219351	CENPO	-1.68E-01	3.45E-05	3.61E-04
ILMN_1772055	VARS	-1.68E-01	1.49E-04	1.27E-03
ILMN_1724304	NLE1	-1.68E-01	1.94E-04	1.59E-03
ILMN_3210921	LOC650515	-1.68E-01	7.41E-04	5.06E-03
ILMN_1692651	PHB	-1.68E-01	1.21E-03	7.66E-03
ILMN_2292748	ING5	-1.68E-01	2.76E-03	1.56E-02
ILMN_1697817	PANX1	-1.68E-01	1.50E-03	9.22E-03

ILMN_1752283	ITCH	-1.68E-01	2.88E-03	1.62E-02
ILMN_1675721	UBE2R2	-1.69E-01	7.81E-06	1.02E-04
ILMN_1667977	TAF1B	-1.69E-01	1.52E-04	1.28E-03
ILMN_1730329	CRSP6	-1.69E-01	1.14E-03	7.33E-03
ILMN_1708482	TMEM80	-1.69E-01	5.00E-03	2.59E-02
ILMN_1774432	DTD1	-1.69E-01	1.33E-03	8.33E-03
ILMN_1658083	ABT1	-1.69E-01	1.32E-03	8.28E-03
ILMN_1678435	SRD5A3	-1.69E-01	2.16E-04	1.74E-03
ILMN_1685170	ANXA11	-1.69E-01	1.36E-04	1.17E-03
ILMN_2297453	ST6GALNAC4	-1.69E-01	2.79E-03	1.57E-02
ILMN_1712155	RPL6	-1.69E-01	3.73E-03	2.02E-02
ILMN_3237153	FAM123B	-1.69E-01	4.18E-04	3.10E-03
ILMN_1751958	NSUN5	-1.69E-01	5.15E-03	2.66E-02
ILMN_1738718	CYB561D2	-1.69E-01	3.33E-03	1.83E-02
ILMN_1689296	ARMC10	-1.69E-01	1.01E-04	9.00E-04
ILMN_1684038	CLIP1	-1.69E-01	3.70E-03	2.00E-02
ILMN_1771039	GTSE1	-1.70E-01	1.08E-02	4.97E-02
ILMN_2344956	ACP1	-1.70E-01	4.90E-04	3.55E-03
ILMN_3279092	LOC645691	-1.70E-01	1.04E-04	9.27E-04
ILMN_1728684	PELP1	-1.70E-01	2.61E-03	1.49E-02
ILMN_1751743	XRCC1	-1.70E-01	7.02E-03	3.45E-02
ILMN_2310621	ATP5J2	-1.70E-01	1.43E-04	1.22E-03
ILMN_3240962	DDRGK1	-1.70E-01	9.29E-04	6.15E-03
ILMN_1803819	IQGAP1	-1.70E-01	1.26E-03	7.96E-03
ILMN_3235647	SIK1	-1.70E-01	7.90E-03	3.82E-02
ILMN_1658104	KBTBD6	-1.70E-01	9.04E-03	4.27E-02
ILMN_1713491	VAMP2	-1.70E-01	6.55E-03	3.25E-02
ILMN_1677610	OVCA2	-1.70E-01	1.44E-04	1.23E-03
ILMN_1717326	SLC29A3	-1.70E-01	2.08E-03	1.22E-02
ILMN_1651259	FLJ36848	-1.70E-01	5.88E-03	2.97E-02
ILMN_1767837	GOLT1B	-1.70E-01	6.43E-04	4.49E-03
ILMN_1773493	TIMM23	-1.71E-01	2.91E-03	1.63E-02
ILMN_1668752	FLJ20850	-1.71E-01	2.48E-03	1.42E-02
ILMN_1674574	VNN1	-1.71E-01	4.19E-03	2.23E-02
ILMN_1692947	LOC148137	-1.71E-01	7.81E-03	3.78E-02
ILMN_1779512	AP4M1	-1.71E-01	2.50E-04	1.98E-03
ILMN_3266666	LOC100128196	-1.71E-01	1.08E-02	4.95E-02
ILMN_1748651	PSMB3	-1.71E-01	4.44E-03	2.35E-02
ILMN_1677113	RNF8	-1.71E-01	3.49E-03	1.91E-02
ILMN_1693804	LOC652675	-1.71E-01	7.42E-03	3.62E-02
ILMN_1682812	C21orf33	-1.71E-01	5.13E-03	2.65E-02
ILMN_1664956	SSU72	-1.71E-01	3.24E-03	1.79E-02
ILMN_3241332	LOC100133916	-1.71E-01	8.00E-04	5.40E-03
ILMN_2300636	HMGXB4	-1.71E-01	1.29E-03	8.10E-03
ILMN_1652472	BRAF	-1.71E-01	5.67E-03	2.88E-02
ILMN_1774949	PIGP	-1.71E-01	1.14E-03	7.34E-03

ILMN_2276504	SCMH1	-1.71E-01	2.97E-04	2.31E-03
ILMN_1794386	IL2RG	-1.71E-01	9.05E-05	8.23E-04
ILMN_1683995	HCG18	-1.71E-01	3.84E-05	3.95E-04
ILMN_1830314		-1.71E-01	1.67E-03	1.01E-02
ILMN_1714896	SART3	-1.72E-01	6.98E-04	4.81E-03
ILMN_1698512	LOC730063	-1.72E-01	1.07E-02	4.93E-02
ILMN_1728199	POLE	-1.72E-01	6.53E-04	4.54E-03
ILMN_1671573	LOC388755	-1.72E-01	1.84E-04	1.52E-03
ILMN_1702866	FLJ22639	-1.72E-01	3.71E-04	2.80E-03
ILMN_2227533	ABHD14B	-1.72E-01	1.93E-03	1.15E-02
ILMN_1846306		-1.72E-01	6.71E-04	4.65E-03
ILMN_1911717		-1.72E-01	7.89E-03	3.81E-02
ILMN_1660357	LOC641825	-1.72E-01	2.89E-03	1.63E-02
ILMN_2262198	PMS2CL	-1.73E-01	8.51E-04	5.70E-03
ILMN_1750457	ZNF268	-1.73E-01	4.49E-05	4.50E-04
ILMN_1765923	C17orf101	-1.73E-01	6.29E-03	3.14E-02
ILMN_3243055	LOC732432	-1.73E-01	1.28E-03	8.06E-03
ILMN_1676459	ZNF785	-1.73E-01	1.03E-03	6.70E-03
ILMN_2352574	ZNF274	-1.73E-01	7.83E-03	3.79E-02
ILMN_3197549	FLJ77644	-1.73E-01	3.43E-04	2.62E-03
ILMN_1678805	POMT2	-1.73E-01	9.19E-04	6.09E-03
ILMN_1732166	SDHAP3	-1.73E-01	1.03E-02	4.78E-02
ILMN_1690138	PHF10	-1.74E-01	6.12E-03	3.07E-02
ILMN_3238818	LOC649076	-1.74E-01	2.58E-03	1.48E-02
ILMN_1732039	DDX3Y	-1.74E-01	4.08E-03	2.18E-02
ILMN_3245569	LOC731528	-1.74E-01	1.08E-02	4.96E-02
ILMN_1710710	DEDD	-1.74E-01	2.53E-03	1.45E-02
ILMN_1801124	KIAA1826	-1.74E-01	1.38E-03	8.59E-03
ILMN_1763875	ABCF1	-1.74E-01	5.94E-04	4.20E-03
ILMN_1802027	MGST2	-1.74E-01	5.17E-03	2.67E-02
ILMN_1707858	H2AFZ	-1.74E-01	5.52E-04	3.93E-03
ILMN_1901532		-1.74E-01	4.14E-06	6.00E-05
ILMN_2285568	NAAA	-1.74E-01	8.33E-03	3.98E-02
ILMN_3283015	LOC100133185	-1.75E-01	5.64E-03	2.87E-02
ILMN_1658821	SAMD1	-1.75E-01	5.70E-03	2.89E-02
ILMN_1742260	SSFA2	-1.75E-01	2.27E-04	1.83E-03
ILMN_1718449	NSUN5C	-1.75E-01	3.54E-04	2.68E-03
ILMN_1758705	IRX6	-1.75E-01	2.72E-03	1.54E-02
ILMN_1660179	MATR3	-1.75E-01	5.50E-04	3.92E-03
ILMN_1783606	MLL5	-1.75E-01	5.84E-03	2.96E-02
ILMN_1711899	ANXA2	-1.75E-01	2.69E-03	1.53E-02
ILMN_1768251	SLC25A26	-1.76E-01	5.38E-03	2.76E-02
ILMN_1703327	PSTPIP1	-1.76E-01	1.04E-03	6.78E-03
ILMN_1753665	PRR4	-1.76E-01	1.75E-04	1.45E-03
ILMN_1727076	OR10H4	-1.76E-01	6.06E-05	5.83E-04
ILMN_3280294	LOC100131737	-1.76E-01	2.03E-04	1.65E-03

ILMN_1766435	WBP11	-1.76E-01	5.21E-03	2.69E-02
ILMN_1782745	RBL1	-1.76E-01	5.58E-05	5.44E-04
ILMN_1672024	ISCA1L	-1.76E-01	9.18E-04	6.08E-03
ILMN_1664353	LOC644655	-1.76E-01	9.64E-04	6.34E-03
ILMN_1767365	PAK1	-1.76E-01	2.40E-03	1.39E-02
ILMN_1777487	ZNF839	-1.76E-01	2.35E-03	1.36E-02
ILMN_3296121	LOC728142	-1.76E-01	5.09E-04	3.67E-03
ILMN_1701477	CCDC101	-1.76E-01	2.65E-03	1.51E-02
ILMN_2375003	MAP4K4	-1.76E-01	3.53E-03	1.93E-02
ILMN_1802708	BTN3A1	-1.77E-01	9.04E-04	6.01E-03
ILMN_1880834	FBXO41	-1.77E-01	3.60E-03	1.96E-02
ILMN_1704070	BCL10	-1.77E-01	5.94E-03	3.00E-02
ILMN_1811966	LOC653895	-1.77E-01	1.80E-03	1.08E-02
ILMN_2395043	HSPA4	-1.77E-01	1.64E-03	9.94E-03
ILMN_1697160	TTC7A	-1.77E-01	6.33E-03	3.16E-02
ILMN_1710406	PSMC1	-1.77E-01	1.06E-02	4.89E-02
ILMN_3308813	MIR599	-1.78E-01	1.48E-03	9.12E-03
ILMN_2189859	FLJ38482	-1.78E-01	3.51E-03	1.92E-02
ILMN_1727023	AMMECR1L	-1.78E-01	1.63E-03	9.91E-03
ILMN_1719870	FAM72D	-1.78E-01	9.34E-03	4.39E-02
ILMN_1652487	RIOK1	-1.78E-01	4.69E-03	2.45E-02
ILMN_1793522	PRKAB1	-1.78E-01	4.51E-03	2.38E-02
ILMN_1669275	ZNF16	-1.78E-01	1.22E-03	7.71E-03
ILMN_1696407	SFRS2	-1.79E-01	8.22E-04	5.53E-03
ILMN_1857265		-1.79E-01	6.32E-05	6.06E-04
ILMN_1780618	TMEM192	-1.79E-01	3.55E-05	3.69E-04
ILMN_1800952	PSMD11	-1.79E-01	2.71E-03	1.54E-02
ILMN_1661409	C3orf63	-1.79E-01	4.77E-04	3.47E-03
ILMN_2125880	ZNF566	-1.79E-01	5.87E-05	5.68E-04
ILMN_1722158	CASP5	-1.79E-01	1.02E-02	4.72E-02
ILMN_3239378	TADA2B	-1.79E-01	2.22E-05	2.48E-04
ILMN_2386967	VPS54	-1.79E-01	6.19E-04	4.34E-03
ILMN_1893909	IKZF2	-1.79E-01	4.25E-03	2.26E-02
ILMN_1706779	LIG1	-1.79E-01	1.29E-03	8.09E-03
ILMN_3187254	LOC100128016	-1.79E-01	6.18E-04	4.34E-03
ILMN_3245507	LOC100133627	-1.79E-01	1.48E-03	9.11E-03
ILMN_1758087	TAOK1	-1.79E-01	1.60E-03	9.77E-03
ILMN_1680967	CIP29	-1.80E-01	2.85E-03	1.61E-02
ILMN_2048326	RPS27A	-1.80E-01	1.14E-03	7.30E-03
ILMN_3299804	ZNF638	-1.80E-01	3.69E-04	2.78E-03
ILMN_2415926	THOC3	-1.80E-01	1.30E-03	8.16E-03
ILMN_1771403	PWWP2A	-1.80E-01	3.32E-03	1.83E-02
ILMN_3221865	RCADH5	-1.80E-01	7.55E-03	3.67E-02
ILMN_1684663	ZDHHC13	-1.80E-01	6.63E-03	3.29E-02
ILMN_1773148	C11orf61	-1.80E-01	8.30E-06	1.08E-04
ILMN_3250798	C11orf58	-1.80E-01	8.47E-03	4.04E-02

ILMN_1660942	LOC653352	-1.80E-01	4.60E-04	3.36E-03
ILMN_2330243	NUDT1	-1.80E-01	4.52E-03	2.38E-02
ILMN_1722409	LOC644816	-1.80E-01	7.94E-03	3.83E-02
ILMN_1815882	HNRNPA1	-1.80E-01	7.50E-03	3.65E-02
ILMN_1669633	ACP1	-1.80E-01	4.62E-04	3.38E-03
ILMN_1775269	EID1	-1.81E-01	4.52E-04	3.31E-03
ILMN_1792910	MNT	-1.81E-01	4.65E-03	2.44E-02
ILMN_3246732	C16orf88	-1.81E-01	7.39E-03	3.61E-02
ILMN_1709630	CCDC107	-1.81E-01	3.67E-03	1.99E-02
ILMN_2289301	TCERG1	-1.81E-01	2.16E-03	1.27E-02
ILMN_3257233	LOC100128340	-1.81E-01	1.05E-02	4.86E-02
ILMN_2130411	KDELR1	-1.81E-01	2.71E-05	2.94E-04
ILMN_1822542		-1.81E-01	1.02E-02	4.73E-02
ILMN_3176040	LOC100130446	-1.81E-01	2.25E-05	2.51E-04
ILMN_1663541	B4GALT7	-1.82E-01	2.84E-04	2.23E-03
ILMN_3276859	LOC401127	-1.82E-01	1.66E-04	1.39E-03
ILMN_1792931	LYSMD1	-1.82E-01	6.39E-05	6.10E-04
ILMN_2332691	CAPN3	-1.82E-01	6.92E-04	4.78E-03
ILMN_1730731	ERLIN1	-1.82E-01	1.53E-04	1.30E-03
ILMN_1809511	GRINL1A	-1.82E-01	4.06E-03	2.17E-02
ILMN_1790987	HUWE1	-1.82E-01	3.64E-03	1.98E-02
ILMN_2299221	RABGGTA	-1.82E-01	1.17E-03	7.47E-03
ILMN_3284137	LOC646208	-1.82E-01	1.72E-03	1.03E-02
ILMN_1718832	SPHAR	-1.83E-01	8.84E-03	4.19E-02
ILMN_2174081	ZNF133	-1.83E-01	3.54E-04	2.69E-03
ILMN_1726417	MRPL33	-1.83E-01	6.59E-04	4.58E-03
ILMN_1682232	MIER1	-1.83E-01	5.81E-03	2.94E-02
ILMN_1896630		-1.83E-01	2.22E-03	1.30E-02
ILMN_1768273	POP1	-1.83E-01	8.65E-03	4.12E-02
ILMN_1655702	ABHD5	-1.83E-01	4.06E-03	2.17E-02
ILMN_1667257	SDHB	-1.83E-01	1.42E-03	8.79E-03
ILMN_3279960	LOC642784	-1.83E-01	4.78E-03	2.50E-02
ILMN_1781276	TMCO4	-1.83E-01	1.89E-06	3.04E-05
ILMN_3245620	NAIF1	-1.83E-01	1.16E-04	1.02E-03
ILMN_1769406	PIAS2	-1.84E-01	6.87E-04	4.75E-03
ILMN_1678808	KIAA0831	-1.84E-01	2.94E-03	1.65E-02
ILMN_1812256	GANC	-1.84E-01	8.74E-04	5.83E-03
ILMN_1717925	SIGMAR1	-1.84E-01	2.65E-05	2.88E-04
ILMN_1736002	COPS5	-1.84E-01	5.68E-03	2.89E-02
ILMN_3199798	LOC389342	-1.84E-01	1.15E-03	7.35E-03
ILMN_1794632	POLR1B	-1.84E-01	7.75E-03	3.75E-02
ILMN_2374692	WAC	-1.84E-01	2.34E-03	1.35E-02
ILMN_1745737	DIS3L2	-1.84E-01	2.98E-04	2.32E-03
ILMN_3256742	LOC100129902	-1.84E-01	7.74E-03	3.75E-02
ILMN_1812062	SGPP2	-1.84E-01	5.48E-03	2.80E-02
ILMN_2199554	LOC388692	-1.84E-01	3.03E-03	1.69E-02

ILMN_2117240	DCK	-1.84E-01	1.83E-03	1.10E-02
ILMN_1666306	SRRD	-1.84E-01	4.40E-04	3.24E-03
ILMN_1767514	LOC441155	-1.84E-01	1.09E-02	4.99E-02
ILMN_1671142	GPR68	-1.85E-01	3.44E-03	1.88E-02
ILMN_1813456	PCBD1	-1.85E-01	8.31E-05	7.64E-04
ILMN_2378868	SFRS5	-1.85E-01	1.34E-03	8.36E-03
ILMN_1722239	TIMM8A	-1.85E-01	1.66E-03	1.01E-02
ILMN_1770768	SLAMF1	-1.85E-01	1.90E-04	1.57E-03
ILMN_1727938	ZNF764	-1.85E-01	1.39E-04	1.19E-03
ILMN_1712754	NFKBIB	-1.85E-01	1.36E-04	1.17E-03
ILMN_1757646	UFM1	-1.85E-01	7.44E-03	3.62E-02
ILMN_3291437	LOC391044	-1.85E-01	4.97E-05	4.92E-04
ILMN_1786658	BOLA3	-1.86E-01	2.88E-03	1.62E-02
ILMN_1671291	EIF3I	-1.86E-01	8.76E-03	4.16E-02
ILMN_1659744	PRMT1	-1.86E-01	2.42E-03	1.40E-02
ILMN_1714444	KLF12	-1.86E-01	1.36E-04	1.17E-03
ILMN_1730824	ZNF512	-1.86E-01	2.16E-03	1.27E-02
ILMN_2296037	PTPN2	-1.87E-01	5.07E-03	2.62E-02
ILMN_1717403	C9orf100	-1.87E-01	7.36E-04	5.03E-03
ILMN_1814820	LOC643109	-1.87E-01	3.62E-05	3.75E-04
ILMN_1678290	HMG20A	-1.87E-01	4.09E-03	2.18E-02
ILMN_3237977	AVL9	-1.87E-01	1.63E-03	9.92E-03
ILMN_1830806		-1.87E-01	7.71E-03	3.74E-02
ILMN_2415776	WWOX	-1.87E-01	4.03E-03	2.16E-02
ILMN_2155272	PIF1	-1.87E-01	6.84E-03	3.38E-02
ILMN_1764489	TBL2	-1.87E-01	8.66E-03	4.12E-02
ILMN_1699489	TUBB6	-1.87E-01	9.70E-03	4.53E-02
ILMN_1859127		-1.87E-01	6.37E-04	4.45E-03
ILMN_2358541	RBMS1	-1.87E-01	8.57E-03	4.08E-02
ILMN_1748393	MARCH9	-1.88E-01	1.54E-04	1.30E-03
ILMN_3230508	NCRNA00152	-1.88E-01	6.12E-04	4.30E-03
ILMN_1730304	ALG10B	-1.88E-01	5.62E-05	5.48E-04
ILMN_3238955	SNORD10	-1.88E-01	5.01E-04	3.62E-03
ILMN_2207419	DDX26B	-1.88E-01	9.58E-05	8.64E-04
ILMN_1709483	MRE11A	-1.88E-01	3.51E-05	3.65E-04
ILMN_1810810	EEF1A1	-1.88E-01	2.27E-04	1.82E-03
ILMN_1690965	DHX9	-1.88E-01	8.44E-03	4.03E-02
ILMN_1694220	LOC339782	-1.88E-01	7.17E-03	3.51E-02
ILMN_1666192	DCTN5	-1.89E-01	6.45E-04	4.50E-03
ILMN_1759789	KAT5	-1.89E-01	7.92E-04	5.35E-03
ILMN_2344007	SIP1	-1.89E-01	1.37E-04	1.18E-03
ILMN_1750321	LBA1	-1.89E-01	1.26E-03	7.94E-03
ILMN_1814600	DEPDC1B	-1.89E-01	1.70E-03	1.03E-02
ILMN_1718424	MRPS28	-1.89E-01	2.51E-03	1.44E-02
ILMN_3239922	TCAM1	-1.89E-01	6.68E-05	6.35E-04
ILMN_2388605	ACTR2	-1.89E-01	9.95E-03	4.63E-02

ILMN_1785177	DNAJC14	-1.90E-01	1.31E-05	1.58E-04
ILMN_1764871	PIGP	-1.90E-01	7.33E-06	9.67E-05
ILMN_1708946	VPS4A	-1.90E-01	4.15E-06	6.01E-05
ILMN_1790412	FLJ39639	-1.90E-01	3.11E-04	2.41E-03
ILMN_2398903	HAX1	-1.90E-01	1.15E-03	7.36E-03
ILMN_3248453	LOC100134101	-1.90E-01	1.74E-05	2.02E-04
ILMN_1754988	N6AMT1	-1.90E-01	5.06E-04	3.65E-03
ILMN_1695490	PLD4	-1.90E-01	1.06E-04	9.44E-04
ILMN_1653871	NAMPT	-1.90E-01	1.97E-03	1.17E-02
ILMN_3274226	LOC642546	-1.91E-01	1.68E-04	1.40E-03
ILMN_3225586	LOC728732	-1.91E-01	1.50E-03	9.23E-03
ILMN_1665107	ITGB1BP1	-1.91E-01	3.09E-04	2.40E-03
ILMN_2385318	ARMC8	-1.91E-01	1.01E-04	9.03E-04
ILMN_2386040	MYO19	-1.91E-01	1.73E-03	1.04E-02
ILMN_1803611	SNAPC3	-1.91E-01	3.11E-04	2.41E-03
ILMN_3247826	PYROXD1	-1.91E-01	2.33E-03	1.35E-02
ILMN_1781672	GAB1	-1.91E-01	3.11E-03	1.73E-02
ILMN_1785831	ZDHHC13	-1.92E-01	2.83E-03	1.60E-02
ILMN_1684770	USF2	-1.92E-01	1.13E-03	7.25E-03
ILMN_2064917	AGGF1	-1.92E-01	7.68E-04	5.21E-03
ILMN_2293167	PPIL5	-1.92E-01	6.00E-04	4.23E-03
ILMN_3282937	LOC646049	-1.92E-01	1.67E-03	1.01E-02
ILMN_1676864	DDX31	-1.92E-01	2.51E-05	2.76E-04
ILMN_1771903	NUP37	-1.92E-01	4.76E-04	3.47E-03
ILMN_1665058	TCHP	-1.92E-01	2.41E-04	1.92E-03
ILMN_1741331	MUDENG	-1.92E-01	5.18E-03	2.67E-02
ILMN_2357193	DDX59	-1.92E-01	1.25E-04	1.09E-03
ILMN_1696583	ZNF169	-1.92E-01	1.60E-04	1.35E-03
ILMN_1758731	CYP2J2	-1.92E-01	9.83E-03	4.59E-02
ILMN_1761181	NOL9	-1.93E-01	1.37E-03	8.55E-03
ILMN_1715901	FBXL17	-1.93E-01	2.87E-04	2.25E-03
ILMN_2145050	POM121	-1.93E-01	9.49E-04	6.26E-03
ILMN_2408908	MAP4K5	-1.93E-01	6.48E-04	4.51E-03
ILMN_1703229	HIATL1	-1.93E-01	4.49E-03	2.37E-02
ILMN_1792353	PHC3	-1.93E-01	1.82E-03	1.09E-02
ILMN_1805673	ALKBH4	-1.93E-01	2.16E-05	2.43E-04
ILMN_1673757	CASP8	-1.93E-01	2.34E-03	1.35E-02
ILMN_1675365	LARP4	-1.93E-01	5.58E-05	5.44E-04
ILMN_1729450	C16orf62	-1.93E-01	1.66E-03	1.01E-02
ILMN_1806818	MCM3	-1.93E-01	5.94E-03	3.00E-02
ILMN_1757636	C5orf35	-1.93E-01	6.85E-05	6.49E-04
ILMN_1798373	ITPRIPL1	-1.94E-01	2.27E-03	1.32E-02
ILMN_1759277	OIP5	-1.94E-01	7.46E-03	3.63E-02
ILMN_1917089		-1.94E-01	1.42E-03	8.79E-03
ILMN_3293025	LOC728170	-1.94E-01	2.48E-04	1.97E-03
ILMN_1709747	EXOG	-1.95E-01	9.70E-06	1.22E-04

ILMN_2083593	TTC32	-1.95E-01	3.20E-04	2.47E-03
ILMN_2387784	DEAF1	-1.95E-01	3.37E-05	3.54E-04
ILMN_1720623	SYTL3	-1.95E-01	4.70E-04	3.43E-03
ILMN_2059505	ARPP19	-1.95E-01	7.72E-04	5.23E-03
ILMN_1694983	DDX20	-1.96E-01	1.99E-03	1.18E-02
ILMN_1780026	ZNF430	-1.96E-01	1.18E-03	7.54E-03
ILMN_1849399		-1.96E-01	1.56E-03	9.52E-03
ILMN_3191636	LOC100128781	-1.96E-01	4.83E-03	2.52E-02
ILMN_1668463	SON	-1.96E-01	1.06E-03	6.85E-03
ILMN_1815083	WHSC2	-1.96E-01	2.25E-03	1.31E-02
ILMN_1727809	STK35	-1.96E-01	2.48E-04	1.97E-03
ILMN_1771393	C3orf23	-1.96E-01	7.29E-05	6.84E-04
ILMN_1786872	C11orf31	-1.96E-01	3.07E-03	1.71E-02
ILMN_2391789	TDP1	-1.96E-01	8.01E-05	7.41E-04
ILMN_1667707	SPCS3	-1.96E-01	2.30E-03	1.34E-02
ILMN_1710522	RUNX1T1	-1.96E-01	1.72E-04	1.43E-03
ILMN_2391765	C6orf48	-1.96E-01	8.57E-04	5.73E-03
ILMN_1720764	UBE1	-1.96E-01	5.71E-05	5.54E-04
ILMN_2342515	FXN	-1.96E-01	6.57E-03	3.26E-02
ILMN_1729269	LOC285074	-1.96E-01	1.31E-05	1.58E-04
ILMN_1772677	CNOT4	-1.97E-01	3.24E-04	2.49E-03
ILMN_1652434	MTHFD2L	-1.97E-01	4.59E-03	2.41E-02
ILMN_3219631	LOC647081	-1.97E-01	3.89E-04	2.91E-03
ILMN_1688565	ZNF580	-1.97E-01	1.46E-03	9.00E-03
ILMN_2204726	UBR5	-1.97E-01	3.21E-04	2.48E-03
ILMN_1779076	HINT3	-1.97E-01	5.48E-06	7.59E-05
ILMN_2405018	PPP1CB	-1.97E-01	9.35E-04	6.18E-03
ILMN_1781360	MPHOSPH6	-1.97E-01	3.86E-04	2.89E-03
ILMN_1781099	ISY1	-1.97E-01	9.98E-03	4.64E-02
ILMN_1726698	PSME1	-1.97E-01	3.51E-03	1.92E-02
ILMN_1787064	ANKRD17	-1.97E-01	8.03E-05	7.43E-04
ILMN_1880184		-1.97E-01	8.33E-06	1.08E-04
ILMN_1673788	CDV3	-1.97E-01	1.26E-05	1.52E-04
ILMN_3237641	MUDENG	-1.98E-01	1.53E-03	9.39E-03
ILMN_1724490	PSPC1	-1.98E-01	2.29E-05	2.55E-04
ILMN_2186858	C14orf131	-1.98E-01	3.11E-03	1.73E-02
ILMN_1810953	LOC653496	-1.98E-01	9.37E-05	8.48E-04
ILMN_2186626	ZNF485	-1.98E-01	7.89E-03	3.81E-02
ILMN_3234513	LOC728416	-1.98E-01	2.59E-03	1.48E-02
ILMN_3266128	LOC100129553	-1.98E-01	7.06E-03	3.47E-02
ILMN_2108135	TAS1R3	-1.98E-01	2.13E-05	2.40E-04
ILMN_1786328	WDR40A	-1.98E-01	5.52E-04	3.93E-03
ILMN_1771376	PEA15	-1.98E-01	8.59E-03	4.09E-02
ILMN_3242851	SENP1	-1.98E-01	2.74E-06	4.18E-05
ILMN_1655734	RPF1	-1.98E-01	8.90E-03	4.22E-02
ILMN_2259633	MLL5	-1.98E-01	2.75E-03	1.56E-02

ILMN_1672176	SMPD2	-1.98E-01	1.05E-03	6.80E-03
ILMN_1703718	CCT7	-1.99E-01	3.42E-03	1.88E-02
ILMN_1814589	LOC728037	-1.99E-01	3.36E-04	2.57E-03
ILMN_3199489	LOC282997	-1.99E-01	5.24E-05	5.15E-04
ILMN_3217625	LOC100132402	-1.99E-01	3.25E-03	1.80E-02
ILMN_2276002	CPNE1	-1.99E-01	9.19E-05	8.34E-04
ILMN_2328835	IP6K2	-1.99E-01	4.15E-04	3.08E-03
ILMN_3241568	LOC100134152	-1.99E-01	9.34E-04	6.18E-03
ILMN_1720059	HMBOX1	-1.99E-01	5.52E-03	2.82E-02
ILMN_2215881	ARHGAP11B	-1.99E-01	3.69E-03	2.00E-02
ILMN_1678962	DFFB	-1.99E-01	3.16E-04	2.44E-03
ILMN_1694888	TAF2	-1.99E-01	7.09E-03	3.48E-02
ILMN_3238481	SNORD60	-1.99E-01	7.68E-03	3.72E-02
ILMN_2323302	SON	-1.99E-01	2.17E-05	2.44E-04
ILMN_1669635	NUP85	-1.99E-01	1.73E-03	1.04E-02
ILMN_2053281	C14orf149	-1.99E-01	9.91E-03	4.61E-02
ILMN_1662331	PDSS2	-2.00E-01	6.15E-03	3.08E-02
ILMN_2129859	FASTKD3	-2.00E-01	4.16E-03	2.22E-02
ILMN_2192693	EIF3M	-2.00E-01	2.43E-03	1.40E-02
ILMN_1720373	SLC7A5	-2.00E-01	5.62E-03	2.86E-02
ILMN_1815361	XIAP	-2.00E-01	5.73E-03	2.91E-02
ILMN_2103107	ADAMDEC1	-2.00E-01	4.89E-03	2.54E-02
ILMN_1777721	MAPRE1	-2.00E-01	6.04E-05	5.81E-04
ILMN_1768396	AURKAIP1	-2.00E-01	1.30E-03	8.14E-03
ILMN_1659517	WDR89	-2.00E-01	9.70E-05	8.72E-04
ILMN_1706326	MRPL33	-2.00E-01	3.66E-03	1.98E-02
ILMN_1776076	POFUT1	-2.01E-01	1.11E-04	9.83E-04
ILMN_1662192	ZNF248	-2.01E-01	2.82E-04	2.21E-03
ILMN_2359500	NCDN	-2.01E-01	1.84E-03	1.10E-02
ILMN_2123402	TMEM4	-2.01E-01	1.08E-02	4.95E-02
ILMN_1759003	SNX12	-2.01E-01	8.70E-06	1.12E-04
ILMN_1654692	RALGPS2	-2.01E-01	1.52E-03	9.30E-03
ILMN_1857915	LOC401397	-2.01E-01	7.92E-03	3.82E-02
ILMN_1728521	HDAC7A	-2.01E-01	2.04E-03	1.21E-02
ILMN_1690099	ITGB1BP1	-2.02E-01	5.70E-03	2.90E-02
ILMN_1903568		-2.02E-01	1.17E-04	1.02E-03
ILMN_2130838	UTP11L	-2.02E-01	1.79E-03	1.07E-02
ILMN_2222991	ETF1	-2.02E-01	7.90E-04	5.34E-03
ILMN_1712766	ERGIC2	-2.02E-01	8.36E-03	4.00E-02
ILMN_2171183	C21orf45	-2.02E-01	1.89E-03	1.13E-02
ILMN_2192620	DCAF15	-2.02E-01	8.12E-05	7.49E-04
ILMN_3245693	ADAT2	-2.02E-01	1.62E-06	2.66E-05
ILMN_1731135	RAB11B	-2.02E-01	3.63E-05	3.76E-04
ILMN_1712211	FLJ10246	-2.02E-01	3.11E-04	2.41E-03
ILMN_1900456		-2.02E-01	1.76E-05	2.03E-04
ILMN_1731023	REPIN1	-2.02E-01	6.39E-03	3.19E-02

ILMN_1794230	SCAND1	-2.03E-01	3.22E-03	1.78E-02
ILMN_3297644	TMEM214	-2.03E-01	2.22E-03	1.30E-02
ILMN_1676844	NR1D2	-2.03E-01	5.15E-03	2.66E-02
ILMN_3300663	LOC728661	-2.03E-01	3.00E-03	1.67E-02
ILMN_1814156	PSMB7	-2.03E-01	1.21E-04	1.05E-03
ILMN_1706511	TEF	-2.03E-01	1.10E-03	7.08E-03
ILMN_2226751	ZC3HAV1L	-2.03E-01	2.56E-04	2.03E-03
ILMN_1727855	PEMT	-2.03E-01	5.25E-03	2.70E-02
ILMN_2287157	DST	-2.03E-01	1.02E-05	1.27E-04
ILMN_1811866	LOC642393	-2.03E-01	1.06E-04	9.39E-04
ILMN_2401761	NUDT9	-2.03E-01	2.15E-03	1.26E-02
ILMN_1654457	TXN2	-2.03E-01	8.26E-05	7.60E-04
ILMN_1771601	UFSP1	-2.03E-01	5.33E-05	5.22E-04
ILMN_2291249	RASSF6	-2.04E-01	6.96E-05	6.58E-04
ILMN_2329165	MYO1C	-2.04E-01	5.18E-03	2.67E-02
ILMN_1804451	LEO1	-2.04E-01	7.47E-04	5.09E-03
ILMN_1732216	NARS	-2.04E-01	6.33E-05	6.06E-04
ILMN_1766366	ICA1L	-2.04E-01	1.61E-05	1.89E-04
ILMN_1730957	NBPF10	-2.04E-01	1.86E-04	1.54E-03
ILMN_1667060	HSF1	-2.04E-01	4.14E-05	4.21E-04
ILMN_2213247	SPCS2	-2.04E-01	1.25E-05	1.52E-04
ILMN_1807611	PPIG	-2.04E-01	5.11E-04	3.68E-03
ILMN_1882560		-2.05E-01	2.46E-04	1.96E-03
ILMN_3268993	LOC100130556	-2.05E-01	6.76E-03	3.34E-02
ILMN_2190051	CCDC91	-2.05E-01	3.22E-03	1.78E-02
ILMN_2171295	PFTK1	-2.05E-01	6.43E-05	6.14E-04
ILMN_2260756	GSDMB	-2.05E-01	9.76E-03	4.56E-02
ILMN_1741264	MRPS33	-2.05E-01	3.12E-03	1.73E-02
ILMN_1675979	RBMS3	-2.05E-01	7.90E-03	3.81E-02
ILMN_1678097	KIAA2010	-2.05E-01	1.20E-04	1.05E-03
ILMN_2102069	SLC16A4	-2.05E-01	2.89E-03	1.62E-02
ILMN_2280630	SERF1A	-2.05E-01	5.74E-05	5.56E-04
ILMN_1690063	LOC651143	-2.05E-01	3.46E-04	2.63E-03
ILMN_2257432	RAD51	-2.06E-01	1.48E-03	9.11E-03
ILMN_1811991	C17orf101	-2.06E-01	5.03E-03	2.61E-02
ILMN_1737124	PRPF4B	-2.06E-01	3.28E-03	1.81E-02
ILMN_1658302	PIAS2	-2.06E-01	6.29E-03	3.14E-02
ILMN_1778543	LOC653874	-2.06E-01	9.71E-03	4.54E-02
ILMN_1799516	DNAJC9	-2.06E-01	6.98E-05	6.59E-04
ILMN_2100815	TMEM9B	-2.07E-01	3.82E-04	2.87E-03
ILMN_1874613		-2.07E-01	8.26E-04	5.55E-03
ILMN_1669669	KCMF1	-2.07E-01	1.22E-05	1.50E-04
ILMN_1769201	ELF3	-2.07E-01	9.74E-03	4.55E-02
ILMN_2394750	EXOC4	-2.07E-01	9.75E-04	6.40E-03
ILMN_1789109	LOC643176	-2.07E-01	6.59E-03	3.27E-02
ILMN_1793673	ZNF766	-2.07E-01	1.52E-04	1.29E-03

ILMN_2345908	DDX11	-2.07E-01	9.04E-03	4.27E-02
ILMN_1771238	CHM	-2.07E-01	9.90E-04	6.48E-03
ILMN_1660345	NGRN	-2.07E-01	2.84E-04	2.23E-03
ILMN_1806951	CSTF3	-2.07E-01	2.32E-03	1.34E-02
ILMN_2372398	ALDH5A1	-2.07E-01	9.18E-03	4.33E-02
ILMN_1716658	ZNF543	-2.07E-01	7.98E-03	3.84E-02
ILMN_2222992	ETF1	-2.08E-01	6.31E-04	4.42E-03
ILMN_1814281	SPC25	-2.08E-01	1.12E-03	7.21E-03
ILMN_2191759	LOC440396	-2.08E-01	2.18E-03	1.27E-02
ILMN_1795564	C11orf84	-2.08E-01	5.23E-04	3.76E-03
ILMN_1655229	SLC7A11	-2.08E-01	2.95E-05	3.16E-04
ILMN_3256004	LOC100130003	-2.08E-01	1.04E-03	6.74E-03
ILMN_1738712	GPR180	-2.08E-01	1.48E-03	9.13E-03
ILMN_1718206	KIAA0100	-2.08E-01	7.14E-04	4.90E-03
ILMN_2395474	REV1	-2.08E-01	4.66E-03	2.44E-02
ILMN_3281195	LOC440459	-2.09E-01	1.77E-04	1.47E-03
ILMN_1739199	FAM39DP	-2.09E-01	2.18E-03	1.27E-02
ILMN_3236373	MSL2	-2.09E-01	7.26E-04	4.97E-03
ILMN_1808898	NPR2	-2.09E-01	1.91E-03	1.14E-02
ILMN_1665571	LOC644869	-2.09E-01	1.09E-04	9.64E-04
ILMN_1671452	MRPL44	-2.09E-01	7.77E-05	7.23E-04
ILMN_1802377	IQCG	-2.09E-01	2.57E-04	2.04E-03
ILMN_2276000	CPNE1	-2.09E-01	3.88E-03	2.09E-02
ILMN_2400250	FAM86A	-2.09E-01	2.06E-03	1.21E-02
ILMN_1803045	TUBGCP5	-2.10E-01	1.15E-05	1.42E-04
ILMN_3238712	SNRNP25	-2.10E-01	1.12E-03	7.18E-03
ILMN_1772845	SNRNP35	-2.10E-01	4.72E-03	2.47E-02
ILMN_1678629	DOCK7	-2.10E-01	2.88E-03	1.62E-02
ILMN_1755664	RPS26	-2.10E-01	1.13E-03	7.23E-03
ILMN_1692133	ZNF226	-2.10E-01	2.05E-03	1.21E-02
ILMN_1784880	TAF9L	-2.10E-01	6.26E-03	3.13E-02
ILMN_1708369	EPS15L1	-2.10E-01	9.67E-03	4.52E-02
ILMN_1655046	NUTF2	-2.10E-01	8.18E-04	5.51E-03
ILMN_2322935	MAPKAPK5	-2.10E-01	7.31E-04	5.00E-03
ILMN_2349006	USP21	-2.10E-01	9.70E-05	8.73E-04
ILMN_1743579	WDR4	-2.10E-01	1.55E-04	1.31E-03
ILMN_3309534	MIR25	-2.11E-01	9.79E-03	4.57E-02
ILMN_2374036	CTSL1	-2.11E-01	1.19E-03	7.56E-03
ILMN_1657144	C1orf69	-2.11E-01	1.69E-05	1.96E-04
ILMN_1684771	PGRMC1	-2.11E-01	1.01E-04	9.00E-04
ILMN_3234884	KIF22	-2.11E-01	7.29E-04	5.00E-03
ILMN_1769451	ILVBL	-2.11E-01	3.26E-04	2.51E-03
ILMN_1659285	PSMG1	-2.11E-01	8.66E-04	5.79E-03
ILMN_1772713	BMS1	-2.11E-01	5.72E-03	2.90E-02
ILMN_1741131	CHRNB1	-2.11E-01	3.74E-04	2.82E-03
ILMN_3307025	ZDHHC4	-2.11E-01	4.10E-03	2.19E-02

ILMN_2383871	ZNF74	-2.11E-01	2.10E-04	1.71E-03
ILMN_1799669	PSORS1C1	-2.11E-01	6.54E-05	6.23E-04
ILMN_1677221	PTCD2	-2.11E-01	6.01E-03	3.03E-02
ILMN_1737195	CENPK	-2.11E-01	3.12E-03	1.73E-02
ILMN_2341645	HNRPH3	-2.11E-01	5.01E-05	4.95E-04
ILMN_1843403		-2.11E-01	1.37E-04	1.17E-03
ILMN_1810233	UGT2B11	-2.12E-01	1.04E-02	4.81E-02
ILMN_1680314	TXN	-2.12E-01	2.57E-06	3.97E-05
ILMN_2282959	DNA2	-2.12E-01	7.87E-03	3.80E-02
ILMN_1799688	CDC23	-2.12E-01	4.47E-04	3.28E-03
ILMN_2137066	ZNF7	-2.12E-01	4.56E-06	6.52E-05
ILMN_2358647	FBXO21	-2.12E-01	1.69E-05	1.96E-04
ILMN_2319544	CAMK2D	-2.12E-01	1.34E-04	1.15E-03
ILMN_1755834	FEN1	-2.13E-01	1.67E-03	1.01E-02
ILMN_1709800	POMZP3	-2.13E-01	2.98E-03	1.67E-02
ILMN_1738333	CCDC50	-2.13E-01	1.57E-04	1.32E-03
ILMN_3297141	LOC729570	-2.13E-01	4.46E-03	2.35E-02
ILMN_2203147	TMPRSS12	-2.13E-01	3.35E-04	2.56E-03
ILMN_1716907	FLJ20254	-2.14E-01	3.18E-05	3.37E-04
ILMN_1687711	ZNF576	-2.14E-01	1.03E-03	6.69E-03
ILMN_1724925	LOC647147	-2.14E-01	6.03E-05	5.81E-04
ILMN_3224290	LOC730052	-2.14E-01	6.67E-06	8.95E-05
ILMN_1657627	CBFA2T3	-2.14E-01	3.63E-03	1.97E-02
ILMN_1764628	LYPLA2	-2.14E-01	1.50E-04	1.27E-03
ILMN_1789007	APOC1	-2.14E-01	2.26E-03	1.32E-02
ILMN_1893764	LOC732172	-2.14E-01	6.86E-04	4.74E-03
ILMN_2317618	TCF19	-2.15E-01	4.04E-03	2.16E-02
ILMN_1703427	SON	-2.15E-01	1.37E-04	1.18E-03
ILMN_1698715	NHLRC2	-2.15E-01	6.95E-04	4.79E-03
ILMN_2380566	SIAH1	-2.15E-01	1.67E-03	1.01E-02
ILMN_1712019	ANKRD17	-2.15E-01	1.36E-05	1.63E-04
ILMN_3235282	ATAD2B	-2.15E-01	1.24E-03	7.83E-03
ILMN_1797576	PLEKHA3	-2.15E-01	3.31E-05	3.49E-04
ILMN_1694126	KIF24	-2.15E-01	1.39E-03	8.63E-03
ILMN_3188110	C19orf60	-2.15E-01	4.76E-03	2.48E-02
ILMN_1665621	LOC642255	-2.16E-01	6.42E-06	8.65E-05
ILMN_1717324	C16orf52	-2.16E-01	3.34E-03	1.84E-02
ILMN_1678404	FBXO11	-2.16E-01	6.84E-04	4.73E-03
ILMN_1775348	KCNH8	-2.16E-01	1.30E-03	8.17E-03
ILMN_1652000	FAM156A	-2.16E-01	9.15E-04	6.06E-03
ILMN_1786976	RAB22A	-2.16E-01	4.74E-05	4.72E-04
ILMN_1652164	LOC729196	-2.16E-01	3.71E-03	2.01E-02
ILMN_1813800	NUFIP1	-2.16E-01	1.37E-05	1.64E-04
ILMN_1797209	TSGA14	-2.16E-01	8.86E-03	4.20E-02
ILMN_1790309	PINX1	-2.16E-01	5.80E-03	2.94E-02
ILMN_1682919	PAFAH2	-2.17E-01	1.07E-03	6.89E-03

ILMN_2050761	EIF4E	-2.17E-01	1.06E-02	4.87E-02
ILMN_3218003	LOC728142	-2.17E-01	1.15E-03	7.36E-03
ILMN_1781419	C11orf73	-2.17E-01	7.28E-05	6.83E-04
ILMN_3236423	RAB3GAP2	-2.17E-01	5.14E-03	2.65E-02
ILMN_1695917	C5orf15	-2.17E-01	5.60E-03	2.85E-02
ILMN_2038776	TXN	-2.17E-01	1.06E-06	1.88E-05
ILMN_1667050	PRPS1	-2.17E-01	6.21E-04	4.35E-03
ILMN_1682494	RSRC1	-2.17E-01	7.05E-04	4.85E-03
ILMN_1651692	STK10	-2.18E-01	2.63E-04	2.08E-03
ILMN_1710150	EED	-2.18E-01	5.24E-03	2.70E-02
ILMN_3246145	EXOG	-2.18E-01	8.13E-05	7.50E-04
ILMN_1772645	AGK	-2.18E-01	7.40E-04	5.05E-03
ILMN_1753016	MRPL35	-2.18E-01	6.24E-04	4.38E-03
ILMN_1781098	DNAJB12	-2.18E-01	4.70E-04	3.43E-03
ILMN_1805590	LSM8	-2.18E-01	4.87E-05	4.83E-04
ILMN_3226700	ZNF837	-2.18E-01	1.23E-05	1.50E-04
ILMN_3309739	MIRLET7D	-2.18E-01	4.49E-03	2.37E-02
ILMN_1701402	IKBIP	-2.18E-01	5.20E-04	3.74E-03
ILMN_3248379	LOC727758	-2.18E-01	1.21E-03	7.69E-03
ILMN_2359029	C11orf17	-2.18E-01	1.03E-03	6.72E-03
ILMN_1807492	LOC643668	-2.19E-01	9.42E-04	6.22E-03
ILMN_1665094	SPTLC1	-2.19E-01	1.88E-03	1.12E-02
ILMN_1667374	C14orf149	-2.19E-01	1.61E-04	1.35E-03
ILMN_1824898	LOC728653	-2.19E-01	5.49E-03	2.80E-02
ILMN_1654421	MPHOSPH9	-2.19E-01	8.94E-05	8.15E-04
ILMN_1675819	FAM101A	-2.19E-01	6.97E-04	4.80E-03
ILMN_1684647	ILKAP	-2.19E-01	5.17E-05	5.08E-04
ILMN_1771629	C14orf124	-2.19E-01	2.93E-04	2.29E-03
ILMN_1652763	LOC389203	-2.19E-01	1.46E-03	9.01E-03
ILMN_1751941	LOC728772	-2.19E-01	1.41E-03	8.74E-03
ILMN_1882000		-2.20E-01	4.93E-04	3.57E-03
ILMN_1712046	CPXM1	-2.20E-01	5.48E-03	2.80E-02
ILMN_1706706	WDR68	-2.20E-01	3.21E-04	2.47E-03
ILMN_2101719	C10orf84	-2.20E-01	3.61E-04	2.73E-03
ILMN_1724367	NDUFB1	-2.20E-01	2.40E-04	1.92E-03
ILMN_2298511	BOLA2	-2.20E-01	1.43E-03	8.83E-03
ILMN_1775235	AFF3	-2.20E-01	1.17E-03	7.48E-03
ILMN_1722674	MGC3196	-2.20E-01	1.75E-05	2.03E-04
ILMN_1696946	LOC654174	-2.20E-01	1.21E-04	1.06E-03
ILMN_2379080	NFATC2IP	-2.20E-01	1.23E-03	7.76E-03
ILMN_1747857	SMARCE1	-2.20E-01	7.38E-04	5.04E-03
ILMN_2324056	GNL3	-2.20E-01	7.77E-03	3.76E-02
ILMN_1698487	SDHD	-2.20E-01	1.28E-03	8.05E-03
ILMN_1683827	VPS24	-2.21E-01	8.57E-05	7.86E-04
ILMN_1762002	CSTF3	-2.21E-01	1.52E-04	1.29E-03
ILMN_3250077	EIF4E	-2.21E-01	2.32E-05	2.58E-04

ILMN_3237589	PHAX	-2.21E-01	1.42E-03	8.80E-03
ILMN_1676280	NSUN3	-2.21E-01	4.07E-05	4.14E-04
ILMN_1661593	FAM18B	-2.21E-01	6.78E-04	4.69E-03
ILMN_2384496	ST6GAL1	-2.21E-01	3.90E-05	4.00E-04
ILMN_2147306	PNRC2	-2.21E-01	7.21E-04	4.95E-03
ILMN_1739083	SIRT1	-2.21E-01	4.61E-03	2.42E-02
ILMN_1668814	CENPM	-2.21E-01	5.26E-03	2.71E-02
ILMN_1782273	N4BP2	-2.21E-01	1.08E-02	4.94E-02
ILMN_1742089	NLN	-2.21E-01	8.53E-03	4.07E-02
ILMN_1767579	KRR1	-2.22E-01	1.15E-03	7.38E-03
ILMN_1744830	ARHGAP11A	-2.22E-01	2.48E-05	2.73E-04
ILMN_1760575	PTP4A1	-2.22E-01	1.90E-03	1.13E-02
ILMN_1756867	ATE1	-2.22E-01	3.16E-03	1.75E-02
ILMN_1664371	HIATL1	-2.22E-01	5.22E-04	3.75E-03
ILMN_1653709	TIMM9	-2.22E-01	1.14E-04	1.00E-03
ILMN_3236135	FAM86D	-2.22E-01	9.80E-06	1.24E-04
ILMN_1757298	BTBD7	-2.22E-01	3.52E-05	3.67E-04
ILMN_3237966	FBXL11	-2.22E-01	4.66E-04	3.40E-03
ILMN_1792314	ACTR1A	-2.22E-01	5.79E-04	4.10E-03
ILMN_2150894	ALDH1B1	-2.22E-01	3.40E-03	1.86E-02
ILMN_1736353	PSMC1	-2.22E-01	1.27E-06	2.17E-05
ILMN_2249720	LAT2	-2.23E-01	3.59E-03	1.95E-02
ILMN_2366785	DDX52	-2.23E-01	4.14E-05	4.21E-04
ILMN_2385278	WDR21A	-2.23E-01	1.20E-04	1.05E-03
ILMN_2134062	ALKBH3	-2.23E-01	9.86E-03	4.60E-02
ILMN_1756998	LOC647691	-2.23E-01	5.07E-06	7.12E-05
ILMN_1774836	PLOD3	-2.23E-01	8.68E-05	7.94E-04
ILMN_2094332	CYorf15B	-2.23E-01	1.74E-04	1.45E-03
ILMN_1799299	PRPF38B	-2.23E-01	7.50E-03	3.65E-02
ILMN_1755811	FBXO4	-2.23E-01	3.47E-03	1.90E-02
ILMN_1785926	ZNF621	-2.23E-01	8.61E-06	1.11E-04
ILMN_2259223	TMTC4	-2.24E-01	8.07E-05	7.46E-04
ILMN_2102330	COL8A2	-2.24E-01	3.31E-03	1.83E-02
ILMN_1673073	WDR62	-2.24E-01	2.94E-05	3.16E-04
ILMN_1678680	DBR1	-2.24E-01	1.91E-04	1.57E-03
ILMN_1769135	DPP7	-2.24E-01	4.23E-03	2.25E-02
ILMN_1774890	LAS1L	-2.25E-01	4.10E-04	3.05E-03
ILMN_3272424	LOC100128836	-2.25E-01	2.13E-03	1.25E-02
ILMN_1783610	HELLS	-2.25E-01	1.49E-03	9.14E-03
ILMN_1863738		-2.25E-01	1.33E-05	1.60E-04
ILMN_3287239	LOC642956	-2.25E-01	7.81E-05	7.25E-04
ILMN_2112829	ANKAR	-2.25E-01	6.88E-03	3.39E-02
ILMN_3235517	ZNF777	-2.25E-01	5.94E-06	8.13E-05
ILMN_2414848	TBRG4	-2.25E-01	6.76E-03	3.34E-02
ILMN_1662970	ZP3	-2.25E-01	7.83E-03	3.79E-02
ILMN_1662334	DNAJA3	-2.26E-01	4.28E-03	2.27E-02

ILMN_1813028	CBX5	-2.26E-01	4.19E-03	2.23E-02
ILMN_3251737	EEF1A1	-2.26E-01	1.37E-03	8.54E-03
ILMN_2173891	C19orf40	-2.26E-01	1.44E-04	1.22E-03
ILMN_1734559	SORD	-2.26E-01	2.17E-06	3.42E-05
ILMN_1823714		-2.26E-01	9.27E-06	1.18E-04
ILMN_1709953	DONSON	-2.26E-01	2.01E-03	1.19E-02
ILMN_2411658	HPS1	-2.26E-01	1.20E-03	7.63E-03
ILMN_1804820	ZNF431	-2.27E-01	7.85E-03	3.79E-02
ILMN_1782257	METT11D1	-2.27E-01	6.70E-03	3.32E-02
ILMN_2408001	RFWD2	-2.27E-01	3.13E-03	1.74E-02
ILMN_1718354	INTS7	-2.27E-01	4.25E-04	3.14E-03
ILMN_1651415	DERL3	-2.27E-01	2.97E-06	4.51E-05
ILMN_1655093	LOC727773	-2.27E-01	1.01E-04	9.01E-04
ILMN_1656540	RUVBL1	-2.27E-01	6.62E-03	3.28E-02
ILMN_1719468	EPM2A	-2.27E-01	6.43E-06	8.65E-05
ILMN_1680682	TADA2A	-2.27E-01	4.26E-05	4.31E-04
ILMN_1883050		-2.27E-01	2.32E-04	1.86E-03
ILMN_1810100	PBX3	-2.27E-01	2.62E-03	1.49E-02
ILMN_3295075	LOC100131531	-2.27E-01	4.33E-05	4.36E-04
ILMN_1809245	PITPNB	-2.28E-01	2.17E-04	1.76E-03
ILMN_2354334	ATXN2L	-2.28E-01	3.39E-04	2.59E-03
ILMN_1705900	ATXN10	-2.28E-01	3.24E-05	3.42E-04
ILMN_1796106	MGAT4B	-2.28E-01	6.70E-03	3.32E-02
ILMN_1696087	PHB2	-2.28E-01	3.32E-04	2.55E-03
ILMN_1734476	KIF2A	-2.28E-01	7.84E-04	5.31E-03
ILMN_1832151		-2.28E-01	1.16E-03	7.41E-03
ILMN_3188099	LOC100130190	-2.28E-01	6.78E-03	3.35E-02
ILMN_1747099	LUC7L2	-2.28E-01	2.88E-04	2.25E-03
ILMN_3284530	LOC647012	-2.28E-01	2.81E-03	1.59E-02
ILMN_1677530	LOC728944	-2.29E-01	5.25E-05	5.15E-04
ILMN_1771203	SMAD2	-2.29E-01	3.16E-05	3.35E-04
ILMN_3237414	LOC653877	-2.29E-01	1.01E-04	9.02E-04
ILMN_1670769	CENPQ	-2.29E-01	5.03E-06	7.07E-05
ILMN_2199284	ANAPC7	-2.29E-01	1.08E-02	4.95E-02
ILMN_1687971	CAPN3	-2.29E-01	1.79E-06	2.91E-05
ILMN_1689371	GIMAP2	-2.29E-01	1.46E-03	8.99E-03
ILMN_3300991	LOC728275	-2.29E-01	4.17E-04	3.10E-03
ILMN_1707337	MSTO1	-2.29E-01	4.71E-04	3.43E-03
ILMN_2235354	PWWP2	-2.29E-01	5.95E-03	3.00E-02
ILMN_1706652	OGG1	-2.29E-01	5.25E-05	5.15E-04
ILMN_1691949	LOC728554	-2.30E-01	1.06E-04	9.43E-04
ILMN_3210946	LOC645969	-2.30E-01	4.05E-03	2.17E-02
ILMN_3203666	LOC645001	-2.30E-01	1.18E-05	1.45E-04
ILMN_2294878	NSUN5	-2.30E-01	3.60E-03	1.96E-02
ILMN_1702198	LOC643790	-2.30E-01	2.21E-05	2.47E-04
ILMN_1701882	LOC653820	-2.30E-01	8.70E-04	5.82E-03

ILMN_1707062	REV1	-2.31E-01	1.85E-03	1.10E-02
ILMN_1677483	EXOSC1	-2.31E-01	1.02E-03	6.64E-03
ILMN_2312719	EXOSC9	-2.31E-01	1.94E-04	1.59E-03
ILMN_2395926	MANBAL	-2.31E-01	5.78E-04	4.10E-03
ILMN_2391231	SORD	-2.31E-01	1.07E-05	1.33E-04
ILMN_1684034	STAT5B	-2.31E-01	2.47E-04	1.97E-03
ILMN_1663827	SMYD5	-2.31E-01	1.25E-03	7.87E-03
ILMN_3228585	LOC728661	-2.31E-01	9.17E-03	4.33E-02
ILMN_3215268	LOC441714	-2.31E-01	6.39E-05	6.10E-04
ILMN_2104830	ACP2	-2.31E-01	4.63E-04	3.38E-03
ILMN_1745499	UXT	-2.31E-01	8.11E-05	7.49E-04
ILMN_1764794	PSMB2	-2.31E-01	2.88E-04	2.25E-03
ILMN_1783170	ING3	-2.32E-01	2.29E-03	1.33E-02
ILMN_1718061	FLJ12688	-2.32E-01	6.41E-04	4.48E-03
ILMN_1743506	CCDC137	-2.32E-01	5.30E-05	5.20E-04
ILMN_1772703	OTUD6B	-2.32E-01	8.55E-06	1.10E-04
ILMN_1720158	ETS2	-2.32E-01	7.33E-05	6.87E-04
ILMN_1811692	FTSJ3	-2.32E-01	5.93E-05	5.73E-04
ILMN_1657470	YTHDF3	-2.32E-01	4.62E-05	4.62E-04
ILMN_1867188		-2.32E-01	7.31E-04	5.00E-03
ILMN_3239959	C17orf106	-2.32E-01	1.90E-03	1.13E-02
ILMN_1798187	MYST2	-2.32E-01	8.70E-05	7.95E-04
ILMN_2067101	POLR1B	-2.32E-01	4.93E-05	4.88E-04
ILMN_1748923	SMC2	-2.32E-01	2.05E-03	1.21E-02
ILMN_1722045	ARSB	-2.32E-01	2.18E-04	1.76E-03
ILMN_3305871	LOC732360	-2.33E-01	7.47E-04	5.09E-03
ILMN_3260111	LOC100129269	-2.33E-01	3.17E-04	2.45E-03
ILMN_2323526	WAC	-2.33E-01	2.31E-03	1.34E-02
ILMN_2356672	EIF2B4	-2.33E-01	3.88E-06	5.68E-05
ILMN_1680465	ARL5B	-2.33E-01	3.48E-03	1.90E-02
ILMN_1799387	INO80	-2.33E-01	5.97E-04	4.22E-03
ILMN_1736481	SECISBP2	-2.33E-01	7.05E-05	6.65E-04
ILMN_2140207	ATPBD4	-2.33E-01	2.35E-05	2.60E-04
ILMN_1808768	ROCK1	-2.33E-01	5.83E-03	2.95E-02
ILMN_3247608	LOC100132289	-2.34E-01	3.39E-04	2.59E-03
ILMN_2232368	PCIF1	-2.34E-01	7.95E-05	7.37E-04
ILMN_2336220	ERAP1	-2.34E-01	1.88E-04	1.55E-03
ILMN_1900110		-2.34E-01	3.25E-04	2.50E-03
ILMN_1734702	MANEA	-2.34E-01	1.91E-04	1.57E-03
ILMN_1762792	MAGED1	-2.34E-01	1.36E-05	1.63E-04
ILMN_1800654	MCM7	-2.34E-01	9.64E-03	4.51E-02
ILMN_1679867	LOC642255	-2.34E-01	1.52E-04	1.28E-03
ILMN_1679501	CSNK2A1	-2.34E-01	7.17E-04	4.92E-03
ILMN_1766762	DYNLRB1	-2.35E-01	1.39E-03	8.61E-03
ILMN_1739274	PDHB	-2.35E-01	1.76E-03	1.06E-02
ILMN_2180624	TMCO6	-2.35E-01	1.15E-06	2.00E-05

ILMN_1720858	C6orf115	-2.35E-01	8.20E-03	3.93E-02
ILMN_1724309	FAM35A	-2.35E-01	3.11E-03	1.73E-02
ILMN_1778464	TMEM1	-2.35E-01	4.52E-04	3.32E-03
ILMN_1735679	DHX38	-2.35E-01	3.46E-05	3.61E-04
ILMN_1669281	CLN3	-2.35E-01	1.93E-04	1.58E-03
ILMN_1804137	ATP11C	-2.35E-01	1.10E-05	1.37E-04
ILMN_1713482	CWC15	-2.35E-01	8.93E-04	5.94E-03
ILMN_1676302	FAM113A	-2.35E-01	5.97E-05	5.75E-04
ILMN_3277321	LOC392264	-2.35E-01	1.07E-04	9.47E-04
ILMN_1770433	PIK3CG	-2.35E-01	6.29E-03	3.14E-02
ILMN_1867321		-2.35E-01	1.03E-04	9.16E-04
ILMN_1737164	TM9SF1	-2.35E-01	4.97E-04	3.59E-03
ILMN_2258363	KLC4	-2.35E-01	8.18E-05	7.54E-04
ILMN_1654560	TLR6	-2.35E-01	1.47E-03	9.06E-03
ILMN_1684802	TAF5	-2.36E-01	7.85E-04	5.31E-03
ILMN_2095506	SPOPL	-2.36E-01	1.07E-02	4.91E-02
ILMN_1659343	BOLA2	-2.36E-01	7.34E-05	6.87E-04
ILMN_1763882	LOC644634	-2.36E-01	3.46E-04	2.64E-03
ILMN_2300396	COMMD5	-2.36E-01	8.99E-06	1.15E-04
ILMN_2310968	RUFY1	-2.36E-01	9.43E-05	8.52E-04
ILMN_2196479	XRN2	-2.36E-01	3.30E-04	2.54E-03
ILMN_2393573	RASSF1	-2.36E-01	6.78E-04	4.70E-03
ILMN_1676611	PHPT1	-2.36E-01	4.38E-03	2.32E-02
ILMN_3244954	TMEM194B	-2.36E-01	2.49E-05	2.73E-04
ILMN_1683774	IL2RA	-2.36E-01	3.44E-03	1.88E-02
ILMN_1685742	NIPA2	-2.36E-01	3.21E-03	1.78E-02
ILMN_1689156	MMAB	-2.36E-01	3.51E-05	3.65E-04
ILMN_1652486	THAP7	-2.37E-01	8.56E-05	7.85E-04
ILMN_2095660	TMEM156	-2.37E-01	5.93E-03	2.99E-02
ILMN_2287147	UBTF	-2.37E-01	1.66E-05	1.94E-04
ILMN_3227136	LOC729020	-2.37E-01	4.34E-04	3.20E-03
ILMN_1765851	TRADD	-2.37E-01	9.24E-03	4.35E-02
ILMN_1898682		-2.37E-01	1.77E-04	1.47E-03
ILMN_2413084	HSPA8	-2.37E-01	1.42E-03	8.79E-03
ILMN_1752281	DNAJC13	-2.38E-01	7.19E-05	6.77E-04
ILMN_1696383	POP4	-2.38E-01	1.81E-03	1.09E-02
ILMN_1750144	C3orf19	-2.38E-01	6.38E-07	1.22E-05
ILMN_1791057	IFNAR2	-2.38E-01	9.39E-04	6.21E-03
ILMN_1658695	NUF2	-2.38E-01	2.67E-04	2.11E-03
ILMN_1790978	ATG7	-2.38E-01	3.21E-03	1.78E-02
ILMN_3219808	LOC391670	-2.38E-01	1.45E-04	1.24E-03
ILMN_1770676	CAPRIN1	-2.38E-01	3.27E-04	2.51E-03
ILMN_2289775	HAX1	-2.38E-01	5.06E-06	7.10E-05
ILMN_1799598	SIRT5	-2.38E-01	1.03E-05	1.29E-04
ILMN_2341363	ATP5A1	-2.38E-01	4.29E-05	4.34E-04
ILMN_1722292	AVL9	-2.38E-01	3.41E-05	3.57E-04

ILMN_2373632	IDH3B	-2.38E-01	1.78E-04	1.47E-03
ILMN_1685535	LARP1B	-2.38E-01	4.27E-07	8.73E-06
ILMN_1769027	CDC42SE1	-2.38E-01	2.13E-04	1.72E-03
ILMN_1704084	CMAH	-2.39E-01	1.69E-03	1.02E-02
ILMN_1802888	ZNF185	-2.39E-01	1.67E-03	1.01E-02
ILMN_1723117	IPO9	-2.39E-01	2.34E-03	1.35E-02
ILMN_1704418	FOXD1	-2.39E-01	1.01E-03	6.59E-03
ILMN_1740976	NONO	-2.39E-01	7.34E-05	6.87E-04
ILMN_1753183	CDCA4	-2.39E-01	3.56E-03	1.94E-02
ILMN_1794187	FBXL3	-2.39E-01	5.95E-04	4.20E-03
ILMN_1712386	C21orf45	-2.39E-01	2.60E-03	1.49E-02
ILMN_1766125	LONP1	-2.39E-01	4.87E-03	2.54E-02
ILMN_3288437	LOC100132715	-2.39E-01	2.40E-06	3.75E-05
ILMN_1751395	KRI1	-2.39E-01	8.50E-06	1.10E-04
ILMN_2414533	ARMCX6	-2.39E-01	3.71E-04	2.79E-03
ILMN_1705151	SF3A3	-2.39E-01	4.72E-03	2.47E-02
ILMN_1661346	LOC648210	-2.40E-01	4.20E-03	2.24E-02
ILMN_1669832	TCF12	-2.40E-01	4.80E-03	2.50E-02
ILMN_1758846	ANKRD40	-2.40E-01	2.28E-04	1.83E-03
ILMN_3304887	LOC729423	-2.40E-01	2.99E-03	1.67E-02
ILMN_1771805	ELK4	-2.40E-01	3.64E-04	2.75E-03
ILMN_1798354	PAPOLA	-2.40E-01	1.12E-03	7.21E-03
ILMN_1713884	C16orf42	-2.40E-01	1.44E-03	8.89E-03
ILMN_1713682	FBXO11	-2.40E-01	4.92E-03	2.55E-02
ILMN_3206111	LOC399881	-2.40E-01	2.13E-06	3.36E-05
ILMN_2389844	SP3	-2.40E-01	5.18E-03	2.67E-02
ILMN_1671742	UPF3A	-2.40E-01	1.05E-03	6.81E-03
ILMN_3252621	LOC100129361	-2.40E-01	2.63E-07	5.87E-06
ILMN_2057768	SLC35D1	-2.40E-01	2.41E-05	2.65E-04
ILMN_1712122	FANCD2	-2.41E-01	1.53E-04	1.29E-03
ILMN_1747423	LOC389901	-2.41E-01	8.90E-04	5.92E-03
ILMN_1696935	RBM39	-2.41E-01	1.73E-05	2.01E-04
ILMN_1721703	PNN	-2.41E-01	4.60E-03	2.41E-02
ILMN_3294318	LOC644173	-2.41E-01	1.03E-05	1.29E-04
ILMN_1683052	PTRH2	-2.41E-01	3.14E-05	3.34E-04
ILMN_2395214	FMNL3	-2.41E-01	8.75E-03	4.16E-02
ILMN_2412101	PRKAG1	-2.41E-01	9.76E-03	4.56E-02
ILMN_1711361	ZNF319	-2.42E-01	4.49E-04	3.29E-03
ILMN_1672191	ATP5F1	-2.42E-01	5.71E-05	5.54E-04
ILMN_1762046	ZNF551	-2.42E-01	8.60E-05	7.87E-04
ILMN_1669851	STAG3L4	-2.42E-01	1.05E-03	6.82E-03
ILMN_2411264	BTBD1	-2.42E-01	3.10E-04	2.40E-03
ILMN_1730005	RFP	-2.42E-01	2.37E-03	1.37E-02
ILMN_1728535	COPZ1	-2.42E-01	3.02E-03	1.68E-02
ILMN_1708164	EIF3A	-2.42E-01	7.45E-04	5.07E-03
ILMN_1792990	ZNF202	-2.42E-01	1.58E-03	9.64E-03

ILMN_3246219	LOC729623	-2.42E-01	2.81E-03	1.58E-02
ILMN_2301624	MACF1	-2.42E-01	4.04E-04	3.01E-03
ILMN_1694980	NAGLU	-2.42E-01	1.04E-04	9.27E-04
ILMN_1654861	ACO2	-2.42E-01	1.38E-04	1.19E-03
ILMN_1785528	FAM39DP	-2.43E-01	1.99E-03	1.18E-02
ILMN_2099798	LYPLA1	-2.43E-01	5.07E-04	3.66E-03
ILMN_1718353	LOC652458	-2.43E-01	4.78E-04	3.47E-03
ILMN_1731609	CHMP6	-2.43E-01	5.87E-04	4.15E-03
ILMN_1732926	PVT1	-2.43E-01	1.18E-04	1.03E-03
ILMN_1716728	C6orf64	-2.43E-01	1.34E-06	2.27E-05
ILMN_1794473	PHF17	-2.43E-01	5.49E-04	3.92E-03
ILMN_2057399	ZBTB8OS	-2.43E-01	5.20E-03	2.68E-02
ILMN_1745885	POLR2F	-2.43E-01	2.30E-03	1.34E-02
ILMN_2414366	KAT5	-2.43E-01	5.77E-05	5.58E-04
ILMN_1729791	LOC650339	-2.43E-01	7.44E-04	5.07E-03
ILMN_1771333	CD47	-2.43E-01	9.49E-04	6.26E-03
ILMN_1788356	C11orf17	-2.43E-01	1.98E-04	1.62E-03
ILMN_1700067	BTN3A2	-2.44E-01	1.33E-04	1.15E-03
ILMN_2204983	DCTN6	-2.44E-01	8.46E-04	5.67E-03
ILMN_1659895	MSN	-2.44E-01	9.02E-03	4.26E-02
ILMN_1672389	CRYZ	-2.44E-01	9.96E-03	4.63E-02
ILMN_3306950	CDK10	-2.44E-01	7.77E-05	7.23E-04
ILMN_1817377		-2.44E-01	3.89E-06	5.69E-05
ILMN_1683854	ZNF484	-2.44E-01	2.80E-07	6.16E-06
ILMN_3281294	LOC643384	-2.45E-01	2.56E-03	1.47E-02
ILMN_1783337	DECR2	-2.45E-01	9.30E-04	6.16E-03
ILMN_1752273	KIAA1143	-2.45E-01	4.36E-04	3.21E-03
ILMN_1809818	PRCC	-2.46E-01	4.71E-04	3.44E-03
ILMN_1756043	WDHD1	-2.46E-01	1.03E-04	9.16E-04
ILMN_1797816	RPTOR	-2.46E-01	7.35E-05	6.88E-04
ILMN_1695034	LOC642817	-2.46E-01	9.78E-03	4.56E-02
ILMN_1780298	FAM86A	-2.46E-01	3.91E-03	2.10E-02
ILMN_1798804	SRPK1	-2.46E-01	7.38E-03	3.60E-02
ILMN_1669094	ZNF672	-2.46E-01	1.15E-03	7.37E-03
ILMN_1676759	DDX27	-2.46E-01	1.20E-04	1.05E-03
ILMN_2062112	ZC3H15	-2.46E-01	1.56E-04	1.32E-03
ILMN_2131936	ATAD3B	-2.46E-01	8.55E-03	4.08E-02
ILMN_2196984	OIP5	-2.47E-01	5.25E-05	5.16E-04
ILMN_1658743	CCNDBP1	-2.47E-01	3.97E-05	4.06E-04
ILMN_2401826	FTSJ1	-2.47E-01	9.25E-05	8.38E-04
ILMN_1728517	FNTB	-2.47E-01	3.95E-07	8.21E-06
ILMN_1694259	NSA2	-2.47E-01	6.69E-03	3.32E-02
ILMN_1780887	USP21	-2.47E-01	1.46E-06	2.44E-05
ILMN_1660723	RDH13	-2.47E-01	6.91E-04	4.77E-03
ILMN_2174574	HNRNPA3P1	-2.47E-01	2.98E-04	2.32E-03
ILMN_1663858	ZNF286A	-2.47E-01	8.94E-04	5.94E-03

ILMN_1719606	LOC644762	-2.47E-01	4.42E-04	3.25E-03
ILMN_1756506	CYorf15B	-2.47E-01	2.65E-05	2.88E-04
ILMN_2223380	PPWD1	-2.48E-01	6.80E-03	3.36E-02
ILMN_1785379	ZNF8	-2.48E-01	2.15E-03	1.26E-02
ILMN_1809894	TMEM117	-2.48E-01	9.30E-03	4.37E-02
ILMN_2405915	MRPS11	-2.48E-01	8.84E-04	5.89E-03
ILMN_2401770	PHF14	-2.48E-01	1.25E-04	1.09E-03
ILMN_1722423	SHPRH	-2.48E-01	1.25E-05	1.52E-04
ILMN_1809027	ATP5SL	-2.48E-01	1.69E-03	1.02E-02
ILMN_1859524		-2.48E-01	1.79E-05	2.07E-04
ILMN_1728975	SCO1	-2.48E-01	6.77E-05	6.42E-04
ILMN_1790625	CBX3	-2.48E-01	4.18E-04	3.10E-03
ILMN_2151579	HMGN1	-2.48E-01	3.43E-05	3.59E-04
ILMN_1724016	C15orf44	-2.49E-01	7.66E-05	7.14E-04
ILMN_1668469	KIAA0922	-2.49E-01	1.52E-03	9.33E-03
ILMN_1791222	GLYCTK	-2.49E-01	1.12E-04	9.86E-04
ILMN_1703408	FZD3	-2.49E-01	1.54E-04	1.30E-03
ILMN_1870111		-2.49E-01	1.73E-03	1.04E-02
ILMN_1655748	ZNF323	-2.49E-01	4.64E-04	3.39E-03
ILMN_1790951	C19orf50	-2.49E-01	5.71E-03	2.90E-02
ILMN_3232696	LOC729816	-2.49E-01	9.59E-06	1.21E-04
ILMN_1709728	SLC30A5	-2.49E-01	3.35E-03	1.84E-02
ILMN_2245180	BUB3	-2.49E-01	8.57E-04	5.74E-03
ILMN_1654392	KHNYN	-2.49E-01	4.36E-05	4.39E-04
ILMN_1788022	CDKAL1	-2.50E-01	1.94E-05	2.21E-04
ILMN_2397571	PIGC	-2.50E-01	1.06E-03	6.84E-03
ILMN_1651504	FAM193A	-2.50E-01	1.38E-05	1.65E-04
ILMN_1782851	TAPBP	-2.50E-01	2.43E-06	3.78E-05
ILMN_1660193	ZNF529	-2.50E-01	3.22E-04	2.48E-03
ILMN_1675462	LSM6	-2.50E-01	2.63E-04	2.08E-03
ILMN_2217574	FABP5L3	-2.50E-01	2.47E-03	1.42E-02
ILMN_1686929	GPATCH3	-2.50E-01	7.93E-06	1.04E-04
ILMN_1673682	GATAD2A	-2.50E-01	6.45E-04	4.50E-03
ILMN_1661351	C17orf81	-2.51E-01	6.37E-05	6.09E-04
ILMN_1713983	FLJ41766	-2.51E-01	2.50E-04	1.98E-03
ILMN_3224868	LOC729200	-2.51E-01	2.09E-04	1.70E-03
ILMN_3191227	LOC100129267	-2.51E-01	3.24E-04	2.49E-03
ILMN_2372011	SCAND1	-2.51E-01	6.93E-06	9.23E-05
ILMN_1669722	LRRC61	-2.51E-01	4.66E-03	2.44E-02
ILMN_2319414	BTF3	-2.51E-01	1.85E-07	4.40E-06
ILMN_2399310	MLLT10	-2.52E-01	7.25E-05	6.81E-04
ILMN_1709882	ICK	-2.52E-01	9.98E-05	8.94E-04
ILMN_2212690	ZC3H7A	-2.52E-01	1.03E-04	9.22E-04
ILMN_1663627	ELMO1	-2.52E-01	3.46E-04	2.64E-03
ILMN_2276431	DPH2	-2.52E-01	1.55E-06	2.57E-05
ILMN_1674152	NFKBIB	-2.52E-01	8.06E-07	1.49E-05

ILMN_1664449	ALG5	-2.52E-01	6.28E-03	3.14E-02
ILMN_1670096	NRBP1	-2.52E-01	1.29E-04	1.11E-03
ILMN_3282773	LOC389322	-2.53E-01	1.27E-07	3.24E-06
ILMN_3240247	NOP10	-2.53E-01	2.89E-05	3.10E-04
ILMN_3278754	LOC649445	-2.53E-01	3.06E-04	2.38E-03
ILMN_1698402	NFX1	-2.53E-01	7.60E-05	7.09E-04
ILMN_1792076	TRERF1	-2.53E-01	4.37E-08	1.34E-06
ILMN_1744713	PARK7	-2.53E-01	1.28E-05	1.54E-04
ILMN_1712639	AIFM2	-2.53E-01	7.38E-04	5.04E-03
ILMN_1654612	ZNF589	-2.53E-01	6.04E-04	4.26E-03
ILMN_2091375	KRCC1	-2.53E-01	3.24E-04	2.49E-03
ILMN_1713803	C17orf97	-2.53E-01	1.17E-05	1.44E-04
ILMN_1725612	NUP50	-2.53E-01	2.53E-03	1.45E-02
ILMN_2372403	ALDH5A1	-2.53E-01	5.66E-03	2.88E-02
ILMN_1672122	P4HTM	-2.53E-01	1.25E-03	7.88E-03
ILMN_1686235	GNPNAT1	-2.53E-01	2.48E-03	1.43E-02
ILMN_1759250	TAP2	-2.53E-01	3.00E-03	1.68E-02
ILMN_1794085	SAPS1	-2.53E-01	3.01E-06	4.56E-05
ILMN_1704704	LOC344405	-2.53E-01	1.42E-05	1.69E-04
ILMN_1739441	GANAB	-2.54E-01	4.33E-03	2.29E-02
ILMN_1729051	MSH6	-2.54E-01	5.38E-04	3.85E-03
ILMN_1807088	TTC33	-2.54E-01	1.58E-03	9.66E-03
ILMN_1657838	JMJD5	-2.54E-01	3.16E-05	3.36E-04
ILMN_1698441	MYST4	-2.54E-01	2.59E-06	4.00E-05
ILMN_1679178	ATP5D	-2.54E-01	2.88E-03	1.62E-02
ILMN_2226314	DBR1	-2.54E-01	2.09E-03	1.23E-02
ILMN_1753568	LRRC34	-2.54E-01	8.36E-06	1.08E-04
ILMN_2046024	DUSP11	-2.54E-01	1.64E-03	9.96E-03
ILMN_1740418	CYP27B1	-2.54E-01	1.38E-04	1.18E-03
ILMN_1757697	NEIL3	-2.54E-01	2.47E-03	1.42E-02
ILMN_2397880	CSTF3	-2.55E-01	2.61E-04	2.06E-03
ILMN_1672042	DOLPP1	-2.55E-01	5.57E-04	3.96E-03
ILMN_2224143	MCM3	-2.55E-01	8.06E-07	1.49E-05
ILMN_1685796	CSDE1	-2.55E-01	4.75E-05	4.73E-04
ILMN_1680347	ZNF317	-2.55E-01	9.56E-03	4.48E-02
ILMN_3241091	LOC100130886	-2.55E-01	7.34E-04	5.02E-03
ILMN_3293049	LOC284167	-2.55E-01	2.49E-05	2.73E-04
ILMN_3263702	LOC100128881	-2.55E-01	7.95E-06	1.04E-04
ILMN_1839422	JRK	-2.55E-01	5.59E-04	3.98E-03
ILMN_3243351	LOC646214	-2.55E-01	2.14E-04	1.74E-03
ILMN_2296036	PTPN2	-2.55E-01	9.51E-04	6.27E-03
ILMN_3227060	LOC728931	-2.56E-01	1.12E-04	9.87E-04
ILMN_3237991	LOC645166	-2.56E-01	5.07E-05	5.00E-04
ILMN_1743939	C5orf24	-2.56E-01	6.43E-04	4.49E-03
ILMN_2276811	METT11D1	-2.56E-01	8.98E-04	5.97E-03
ILMN_2166686	NUFIP1	-2.56E-01	3.52E-03	1.92E-02

ILMN_1794967	EIF4ENIF1	-2.56E-01	1.92E-04	1.58E-03
ILMN_1795128	C13orf23	-2.56E-01	3.03E-03	1.69E-02
ILMN_1802699	PCK2	-2.56E-01	5.29E-03	2.72E-02
ILMN_1778557	CDC2L5	-2.56E-01	1.20E-03	7.64E-03
ILMN_2391551	C13orf23	-2.56E-01	8.32E-06	1.08E-04
ILMN_1749709	NDUFB11	-2.56E-01	2.60E-05	2.84E-04
ILMN_1804789	KIAA1967	-2.57E-01	1.67E-05	1.95E-04
ILMN_1744574	TRUB1	-2.57E-01	2.72E-05	2.95E-04
ILMN_1712487	LGTN	-2.57E-01	3.38E-03	1.86E-02
ILMN_3245773	PION	-2.57E-01	5.01E-04	3.62E-03
ILMN_1759460	TAF7	-2.57E-01	9.05E-03	4.28E-02
ILMN_1732187	TMEM143	-2.57E-01	2.23E-05	2.49E-04
ILMN_1704261	RANGRF	-2.57E-01	6.02E-04	4.24E-03
ILMN_1720542	POLR2I	-2.57E-01	1.59E-05	1.86E-04
ILMN_1747506	DHX34	-2.57E-01	2.16E-04	1.75E-03
ILMN_2248725	TYSND1	-2.58E-01	7.45E-05	6.96E-04
ILMN_2077886	C1orf109	-2.58E-01	2.91E-03	1.64E-02
ILMN_2049063	KBTBD6	-2.58E-01	2.03E-06	3.23E-05
ILMN_1708047	LOC648581	-2.58E-01	9.91E-04	6.49E-03
ILMN_2325337	APOL2	-2.58E-01	2.75E-03	1.56E-02
ILMN_1685845	POLDIP3	-2.58E-01	1.40E-03	8.67E-03
ILMN_1761474	LOC654189	-2.58E-01	1.94E-05	2.21E-04
ILMN_1652123	HMGN1	-2.58E-01	2.26E-03	1.32E-02
ILMN_3235832	LOC728835	-2.58E-01	8.73E-03	4.15E-02
ILMN_1677385	C8orf40	-2.58E-01	2.69E-03	1.53E-02
ILMN_1755658	ABI3	-2.58E-01	1.43E-05	1.69E-04
ILMN_1875342		-2.58E-01	4.06E-03	2.17E-02
ILMN_1707137	C17orf97	-2.59E-01	1.44E-05	1.71E-04
ILMN_1701077	LOC642897	-2.59E-01	1.38E-04	1.18E-03
ILMN_2358540	RBMS1	-2.59E-01	1.33E-03	8.33E-03
ILMN_1778488	WDR41	-2.59E-01	2.05E-03	1.21E-02
ILMN_1774800	KTELC1	-2.59E-01	1.03E-02	4.76E-02
ILMN_1699357	SLC22A5	-2.59E-01	3.50E-07	7.43E-06
ILMN_1696151	MON1A	-2.59E-01	8.31E-05	7.64E-04
ILMN_2186216	GOLPH4	-2.60E-01	2.63E-04	2.08E-03
ILMN_1721636	TSC22D4	-2.60E-01	8.34E-04	5.60E-03
ILMN_2355033	KIAA1147	-2.60E-01	1.00E-03	6.53E-03
ILMN_2356991	CD47	-2.60E-01	2.73E-06	4.17E-05
ILMN_1781097	UBXN4	-2.60E-01	6.37E-03	3.18E-02
ILMN_2116127	NPEPPS	-2.60E-01	3.55E-03	1.94E-02
ILMN_2414007	NME2	-2.60E-01	8.45E-03	4.03E-02
ILMN_1805742	DHX37	-2.60E-01	9.53E-07	1.72E-05
ILMN_2401844	PATZ1	-2.60E-01	2.09E-06	3.31E-05
ILMN_1706583	DLAT	-2.60E-01	1.59E-03	9.70E-03
ILMN_1764431	COPS6	-2.60E-01	2.89E-03	1.63E-02
ILMN_2074880	GTF3C6	-2.60E-01	9.57E-05	8.63E-04

ILMN_1699188	LOC144481	-2.61E-01	2.25E-04	1.82E-03
ILMN_1765525	WBSCR16	-2.61E-01	2.75E-03	1.56E-02
ILMN_2169736	PGBD4	-2.61E-01	2.90E-03	1.63E-02
ILMN_1708510	NFATC2IP	-2.61E-01	1.83E-04	1.52E-03
ILMN_1696160	TRQ1	-2.61E-01	1.03E-02	4.75E-02
ILMN_2311089	BRCA1	-2.61E-01	4.60E-06	6.56E-05
ILMN_3220769	LOC729964	-2.61E-01	5.98E-04	4.22E-03
ILMN_3201239	LOC389873	-2.61E-01	1.31E-04	1.13E-03
ILMN_2412214	LGALS9	-2.61E-01	1.53E-04	1.29E-03
ILMN_2076940	C1orf149	-2.61E-01	1.27E-05	1.53E-04
ILMN_1693466	RABEP2	-2.61E-01	3.51E-06	5.21E-05
ILMN_1722858	PPP2CA	-2.62E-01	1.51E-04	1.28E-03
ILMN_2048982	ZBTB25	-2.62E-01	7.21E-04	4.95E-03
ILMN_3271218	LOC100128737	-2.62E-01	7.82E-07	1.45E-05
ILMN_2089656	C1orf107	-2.62E-01	1.82E-04	1.50E-03
ILMN_1735347	MCEE	-2.62E-01	3.13E-06	4.72E-05
ILMN_2183331	RBM7	-2.62E-01	9.58E-04	6.31E-03
ILMN_1706859	C22orf32	-2.62E-01	2.03E-04	1.66E-03
ILMN_1678140	TTC4	-2.62E-01	1.69E-06	2.77E-05
ILMN_1713966	MRPL52	-2.62E-01	4.59E-03	2.41E-02
ILMN_2174394	MMS19L	-2.62E-01	1.66E-06	2.73E-05
ILMN_1753110	ZMYND11	-2.62E-01	3.30E-04	2.53E-03
ILMN_1693862	MGC70857	-2.62E-01	1.42E-05	1.69E-04
ILMN_1777499	LOC731007	-2.62E-01	2.76E-05	2.99E-04
ILMN_3304691	LOC729366	-2.62E-01	3.43E-05	3.58E-04
ILMN_1673960	MAT2B	-2.63E-01	7.75E-06	1.02E-04
ILMN_1854833		-2.63E-01	7.94E-03	3.83E-02
ILMN_1793632	TMEM222	-2.63E-01	2.03E-04	1.65E-03
ILMN_1668027	LOC727762	-2.63E-01	2.11E-06	3.34E-05
ILMN_1717334	VAV1	-2.63E-01	2.41E-06	3.75E-05
ILMN_1691927	BTBD1	-2.63E-01	2.73E-04	2.15E-03
ILMN_1743910	PANK4	-2.63E-01	6.69E-04	4.64E-03
ILMN_1659564	SEC61A1	-2.63E-01	6.20E-04	4.35E-03
ILMN_1728074	PHAX	-2.63E-01	1.70E-04	1.42E-03
ILMN_1683562	SNRPG	-2.63E-01	1.04E-04	9.24E-04
ILMN_2375484	CPEB2	-2.63E-01	4.62E-03	2.42E-02
ILMN_1724734	UQCC	-2.63E-01	1.25E-05	1.52E-04
ILMN_1806106	GNL3	-2.63E-01	1.54E-07	3.78E-06
ILMN_1698770	C5orf33	-2.64E-01	1.36E-04	1.17E-03
ILMN_1814113	ZFR	-2.64E-01	8.15E-03	3.91E-02
ILMN_3305751	LOC729858	-2.64E-01	6.28E-05	6.01E-04
ILMN_1781516	SUPT16H	-2.64E-01	4.69E-03	2.45E-02
ILMN_1679188	ATP5S	-2.64E-01	1.41E-04	1.21E-03
ILMN_3274351	LOC644037	-2.64E-01	4.45E-05	4.47E-04
ILMN_1665647	CD180	-2.64E-01	5.83E-07	1.14E-05
ILMN_3239225	RNY3	-2.64E-01	1.53E-05	1.80E-04

ILMN_2234412	TLE3	-2.65E-01	7.53E-04	5.12E-03
ILMN_1694686	KIAA0194	-2.65E-01	3.53E-05	3.67E-04
ILMN_1763634	PEX14	-2.65E-01	2.69E-03	1.53E-02
ILMN_1778371	CCBL2	-2.65E-01	3.61E-04	2.73E-03
ILMN_2319910	DGKA	-2.65E-01	8.82E-06	1.13E-04
ILMN_1684929	TOPBP1	-2.66E-01	1.43E-03	8.86E-03
ILMN_2059211	KIAA0195	-2.66E-01	4.29E-05	4.34E-04
ILMN_1673543	PGM2	-2.66E-01	1.14E-04	9.99E-04
ILMN_1805828	VRK1	-2.66E-01	2.42E-03	1.40E-02
ILMN_1800573	RPS21	-2.66E-01	1.36E-03	8.46E-03
ILMN_1745116	ABHD12	-2.66E-01	2.03E-04	1.66E-03
ILMN_1722034	KIAA1586	-2.67E-01	5.53E-06	7.64E-05
ILMN_1677714	LOC727773	-2.67E-01	4.80E-06	6.79E-05
ILMN_1733581	C16orf35	-2.67E-01	3.61E-05	3.74E-04
ILMN_3236732	LOC100129637	-2.67E-01	2.43E-05	2.68E-04
ILMN_2362581	FNDC3A	-2.67E-01	4.53E-06	6.49E-05
ILMN_2395981	PYHIN1	-2.67E-01	5.54E-04	3.95E-03
ILMN_1737084	TXLNA	-2.67E-01	3.32E-04	2.55E-03
ILMN_3298070	LOC728698	-2.67E-01	9.54E-05	8.61E-04
ILMN_1776104	NDUFS5	-2.67E-01	1.39E-04	1.19E-03
ILMN_1708660	RWDD4A	-2.67E-01	2.21E-06	3.47E-05
ILMN_1653980	METTL8	-2.67E-01	1.49E-05	1.76E-04
ILMN_1697701	PLEKHJ1	-2.67E-01	6.16E-03	3.09E-02
ILMN_1743538	MLLT10	-2.67E-01	4.50E-07	9.12E-06
ILMN_1660976	LOC653204	-2.68E-01	2.20E-04	1.77E-03
ILMN_3237256	BEND4	-2.68E-01	1.21E-03	7.67E-03
ILMN_1806486	LOC389137	-2.68E-01	5.92E-07	1.15E-05
ILMN_1787081	TADA2A	-2.68E-01	8.28E-06	1.07E-04
ILMN_1844611		-2.68E-01	1.11E-05	1.38E-04
ILMN_1807873	SNX6	-2.68E-01	8.61E-05	7.88E-04
ILMN_1726930	C5orf44	-2.68E-01	3.61E-04	2.73E-03
ILMN_1760620	TMEM33	-2.68E-01	9.21E-04	6.10E-03
ILMN_1721225	C20orf4	-2.68E-01	3.00E-04	2.33E-03
ILMN_2401822	FTSJ1	-2.69E-01	6.36E-04	4.44E-03
ILMN_3244281	SNORD1A	-2.69E-01	3.65E-04	2.76E-03
ILMN_2262901	RUFY3	-2.69E-01	5.61E-03	2.86E-02
ILMN_1668834	RSF1	-2.69E-01	9.74E-05	8.75E-04
ILMN_2337835	ZNF182	-2.69E-01	5.97E-05	5.75E-04
ILMN_1685547	ZXDB	-2.69E-01	1.99E-06	3.16E-05
ILMN_1656521	CGI-96	-2.69E-01	5.66E-06	7.80E-05
ILMN_1712161	BCOR	-2.69E-01	4.08E-06	5.93E-05
ILMN_2375002	MAP4K4	-2.69E-01	7.02E-05	6.63E-04
ILMN_1769883	IDE	-2.69E-01	3.63E-05	3.76E-04
ILMN_1677452	REXO4	-2.70E-01	1.50E-03	9.22E-03
ILMN_2401618	MLX	-2.70E-01	9.74E-06	1.23E-04
ILMN_3247592	ZNF830	-2.70E-01	1.69E-05	1.96E-04

ILMN_3275696	LOC100131940	-2.70E-01	3.61E-04	2.73E-03
ILMN_1777220	VCP	-2.71E-01	1.07E-05	1.33E-04
ILMN_1757437	UMPS	-2.71E-01	2.35E-04	1.88E-03
ILMN_3246560	EMB	-2.71E-01	6.12E-04	4.30E-03
ILMN_1763627	TNPO1	-2.71E-01	1.86E-04	1.54E-03
ILMN_2056975	HPRT1	-2.71E-01	1.97E-05	2.24E-04
ILMN_3295419	LOC644363	-2.71E-01	3.10E-03	1.72E-02
ILMN_1668526	GVIN1	-2.71E-01	2.50E-03	1.44E-02
ILMN_1799579	CCDC51	-2.71E-01	2.34E-04	1.88E-03
ILMN_1729591	LOC642701	-2.71E-01	5.41E-05	5.29E-04
ILMN_2186597	RPP21	-2.71E-01	5.93E-03	2.99E-02
ILMN_1783815	COG7	-2.71E-01	4.17E-07	8.60E-06
ILMN_1676058	MAGOHB	-2.71E-01	1.99E-04	1.63E-03
ILMN_1659553	ANAPC1	-2.71E-01	2.83E-05	3.06E-04
ILMN_3181296	LOC100130623	-2.71E-01	2.92E-04	2.28E-03
ILMN_1740490	ZFP82	-2.72E-01	2.48E-06	3.84E-05
ILMN_3289650	LOC402112	-2.72E-01	2.70E-04	2.13E-03
ILMN_1748481	TMEM199	-2.72E-01	2.13E-06	3.37E-05
ILMN_3291778	LOC441714	-2.72E-01	1.57E-06	2.60E-05
ILMN_1741300	ZNF407	-2.72E-01	1.33E-04	1.14E-03
ILMN_3247163	TET1	-2.72E-01	1.86E-05	2.14E-04
ILMN_2392472	CENPA	-2.72E-01	3.83E-06	5.62E-05
ILMN_1719256	CKS1B	-2.72E-01	8.48E-04	5.68E-03
ILMN_1670134	FADS1	-2.72E-01	8.68E-05	7.94E-04
ILMN_3240721	LOC645233	-2.72E-01	1.07E-05	1.34E-04
ILMN_2275583	LOC344405	-2.72E-01	1.45E-05	1.72E-04
ILMN_1673069	DPP9	-2.72E-01	1.83E-04	1.52E-03
ILMN_1685109	POLR3D	-2.73E-01	2.69E-04	2.12E-03
ILMN_2148150	CHAC2	-2.73E-01	4.55E-05	4.56E-04
ILMN_1715718	ZNF784	-2.73E-01	5.03E-04	3.63E-03
ILMN_1754315	MMAA	-2.73E-01	2.98E-05	3.19E-04
ILMN_1800197	MRPL36	-2.73E-01	8.46E-03	4.04E-02
ILMN_3229859	MOBKL3	-2.73E-01	3.17E-04	2.45E-03
ILMN_1739283	UPF2	-2.73E-01	4.15E-03	2.21E-02
ILMN_1728380	PHOSPHO2	-2.73E-01	1.14E-05	1.41E-04
ILMN_1660817	DDB2	-2.73E-01	2.32E-06	3.63E-05
ILMN_1671925	SDHAP3	-2.73E-01	6.13E-03	3.08E-02
ILMN_1729832	LOC653994	-2.73E-01	2.36E-04	1.89E-03
ILMN_3203874	LOC648927	-2.74E-01	1.18E-06	2.05E-05
ILMN_1690708	SPTBN1	-2.74E-01	7.20E-06	9.54E-05
ILMN_1906187		-2.74E-01	1.03E-02	4.75E-02
ILMN_3309349	SNHG8	-2.74E-01	7.83E-05	7.27E-04
ILMN_3183346	LOC100129466	-2.74E-01	1.27E-03	8.02E-03
ILMN_1664028	CENPB	-2.75E-01	1.73E-03	1.04E-02
ILMN_1666960	NOP2	-2.75E-01	3.90E-05	4.00E-04
ILMN_2411190	SMC2	-2.75E-01	9.07E-05	8.25E-04

ILMN_2299843	ATP5S	-2.75E-01	3.95E-07	8.22E-06
ILMN_1724837	ZC3HAV1	-2.75E-01	1.20E-03	7.62E-03
ILMN_1688180	ASPSCR1	-2.75E-01	4.15E-03	2.21E-02
ILMN_2387090	CGGBP1	-2.75E-01	3.53E-03	1.93E-02
ILMN_1661170	NDUFB8	-2.75E-01	6.48E-04	4.51E-03
ILMN_2056551	RBMX2	-2.75E-01	6.22E-03	3.11E-02
ILMN_3213176	LOC728263	-2.75E-01	1.77E-05	2.05E-04
ILMN_1758906	GNA13	-2.75E-01	8.89E-04	5.92E-03
ILMN_1665601	LOC728944	-2.75E-01	6.82E-05	6.47E-04
ILMN_1679450	LOC730746	-2.75E-01	3.39E-03	1.86E-02
ILMN_1718907	TSHZ1	-2.75E-01	1.98E-05	2.25E-04
ILMN_2291644	SIRT5	-2.76E-01	6.33E-06	8.56E-05
ILMN_3189621	LOC100128221	-2.76E-01	3.14E-06	4.73E-05
ILMN_1748827	LOC388564	-2.76E-01	1.41E-03	8.73E-03
ILMN_1753286	MYO19	-2.76E-01	6.05E-04	4.26E-03
ILMN_1734317	DPF2	-2.76E-01	9.06E-06	1.16E-04
ILMN_2356890	MRPL42	-2.76E-01	3.44E-04	2.62E-03
ILMN_1687316	NCDN	-2.76E-01	2.03E-04	1.66E-03
ILMN_2359789	RAC1	-2.76E-01	2.95E-04	2.30E-03
ILMN_1730101	GSPT2	-2.76E-01	3.07E-05	3.27E-04
ILMN_1720526	CENPN	-2.77E-01	4.37E-04	3.22E-03
ILMN_1807074	MIF	-2.77E-01	2.34E-03	1.36E-02
ILMN_2259818	MFI2	-2.77E-01	2.00E-05	2.27E-04
ILMN_2347917	EED	-2.77E-01	7.18E-03	3.52E-02
ILMN_1755405	FRAG1	-2.77E-01	8.05E-04	5.43E-03
ILMN_1798880	RPA4	-2.77E-01	5.69E-06	7.83E-05
ILMN_3306028	LOC730183	-2.78E-01	8.67E-08	2.34E-06
ILMN_3244154	SNORA84	-2.78E-01	5.54E-06	7.65E-05
ILMN_1782993	DHODH	-2.78E-01	1.02E-05	1.27E-04
ILMN_1767642	C11orf46	-2.78E-01	3.38E-05	3.55E-04
ILMN_2221076	C17orf85	-2.78E-01	3.94E-05	4.03E-04
ILMN_1763628	LOC653188	-2.78E-01	8.04E-03	3.87E-02
ILMN_1721713	EXOSC9	-2.78E-01	2.67E-04	2.10E-03
ILMN_1726138	EI24	-2.78E-01	2.88E-03	1.62E-02
ILMN_1734205	RASSF1	-2.78E-01	8.87E-04	5.91E-03
ILMN_1738173	METTL4	-2.78E-01	9.86E-04	6.46E-03
ILMN_1679891	NAF1	-2.78E-01	5.52E-03	2.82E-02
ILMN_1697546	BRCC3	-2.78E-01	1.60E-04	1.35E-03
ILMN_2375651	SCNM1	-2.79E-01	1.64E-04	1.37E-03
ILMN_1694367	SNORD35B	-2.79E-01	8.10E-03	3.90E-02
ILMN_1801913	PPIH	-2.79E-01	1.43E-03	8.82E-03
ILMN_1738767	PLP2	-2.79E-01	1.03E-03	6.72E-03
ILMN_1656424	SNRPE	-2.79E-01	1.38E-03	8.59E-03
ILMN_1678863	C7orf29	-2.79E-01	5.03E-05	4.96E-04
ILMN_2062524	RBBP4	-2.79E-01	2.97E-04	2.31E-03
ILMN_1660199	ACAA2	-2.79E-01	4.04E-06	5.88E-05

ILMN_1662954	CCT7	-2.79E-01	1.42E-03	8.77E-03
ILMN_2074258	BARD1	-2.80E-01	1.44E-04	1.23E-03
ILMN_1720241	TRIP12	-2.80E-01	1.11E-05	1.37E-04
ILMN_1733703	TRMU	-2.80E-01	3.34E-03	1.84E-02
ILMN_1763408	CCBL2	-2.80E-01	2.10E-04	1.70E-03
ILMN_2268026	C15orf44	-2.80E-01	1.23E-05	1.50E-04
ILMN_1810374	TMEM156	-2.80E-01	1.55E-03	9.45E-03
ILMN_1680644	MIOS	-2.80E-01	3.62E-04	2.74E-03
ILMN_1743806	MIF4GD	-2.80E-01	7.95E-03	3.83E-02
ILMN_3287058	LOC100132086	-2.80E-01	1.87E-04	1.54E-03
ILMN_1801939	CCNB2	-2.80E-01	5.10E-07	1.02E-05
ILMN_3287422	LOC650515	-2.80E-01	1.17E-03	7.45E-03
ILMN_3251145	LDHA	-2.80E-01	1.69E-04	1.41E-03
ILMN_1796949	TPX2	-2.80E-01	1.82E-05	2.09E-04
ILMN_1683980	PLEKHM2	-2.80E-01	4.14E-05	4.21E-04
ILMN_1808811	SBNO2	-2.80E-01	7.00E-05	6.61E-04
ILMN_1756578	CHRNA6	-2.80E-01	6.21E-03	3.11E-02
ILMN_1682572	KIAA0528	-2.81E-01	2.46E-05	2.70E-04
ILMN_1799069	LOC440280	-2.81E-01	3.59E-05	3.72E-04
ILMN_1697975	PHF14	-2.81E-01	3.29E-07	7.08E-06
ILMN_1680867	C6orf61	-2.81E-01	8.67E-06	1.12E-04
ILMN_1676523	CCDC91	-2.81E-01	1.07E-03	6.92E-03
ILMN_2182647	PINX1	-2.81E-01	1.38E-04	1.18E-03
ILMN_2233539	SLC39A8	-2.81E-01	6.88E-03	3.39E-02
ILMN_1913336		-2.81E-01	1.43E-06	2.40E-05
ILMN_1771233	CHMP4B	-2.81E-01	5.08E-05	5.01E-04
ILMN_1667079	SPTBN2	-2.81E-01	6.94E-04	4.79E-03
ILMN_1671353	IL12A	-2.82E-01	1.73E-04	1.44E-03
ILMN_2101278	RGS18	-2.82E-01	3.76E-04	2.83E-03
ILMN_3243268	ALG10	-2.82E-01	3.53E-04	2.68E-03
ILMN_1802649	C11orf58	-2.82E-01	1.48E-04	1.26E-03
ILMN_2354855	OTUB1	-2.82E-01	2.62E-07	5.84E-06
ILMN_1804597	C2orf37	-2.83E-01	4.60E-03	2.41E-02
ILMN_1799939	TAF1L	-2.83E-01	4.79E-05	4.76E-04
ILMN_1756049	NT5DC3	-2.83E-01	3.79E-07	7.94E-06
ILMN_1738819	EFTUD2	-2.83E-01	8.16E-07	1.50E-05
ILMN_1798816	GOSR1	-2.83E-01	2.53E-06	3.92E-05
ILMN_1703791	ANXA7	-2.83E-01	1.06E-04	9.41E-04
ILMN_2100689	MAP2K4	-2.83E-01	8.80E-05	8.03E-04
ILMN_1679543	KPTN	-2.83E-01	5.14E-06	7.19E-05
ILMN_1772123	ACACA	-2.83E-01	4.54E-05	4.55E-04
ILMN_1655924	TRNT1	-2.83E-01	8.41E-04	5.63E-03
ILMN_1733164	FBXO11	-2.84E-01	8.18E-06	1.06E-04
ILMN_1726107	UBE2V1	-2.84E-01	2.55E-05	2.79E-04
ILMN_1683250	LOC440731	-2.84E-01	6.26E-07	1.21E-05
ILMN_1797307	BUB1B	-2.84E-01	6.65E-04	4.62E-03

ILMN_1687430	EIF2B4	-2.84E-01	4.86E-04	3.53E-03
ILMN_2415949	MRRF	-2.84E-01	9.69E-05	8.72E-04
ILMN_1741483	HPS1	-2.84E-01	8.60E-05	7.88E-04
ILMN_1755758	RIF1	-2.85E-01	1.99E-04	1.63E-03
ILMN_1709334	TM9SF1	-2.85E-01	2.36E-08	8.20E-07
ILMN_3242977	FLJ33630	-2.85E-01	5.78E-04	4.10E-03
ILMN_1652300	ZNF507	-2.85E-01	5.98E-07	1.16E-05
ILMN_1703053	ZFP91	-2.85E-01	1.05E-04	9.35E-04
ILMN_1725441	NFATC2IP	-2.85E-01	3.33E-04	2.55E-03
ILMN_2103295	TINP1	-2.85E-01	1.56E-04	1.32E-03
ILMN_1804610	NFX1	-2.85E-01	1.17E-03	7.46E-03
ILMN_2383754	GTPBP10	-2.85E-01	4.03E-05	4.11E-04
ILMN_1687958	SLC25A22	-2.85E-01	1.72E-03	1.03E-02
ILMN_1659777	RUFY1	-2.85E-01	2.97E-06	4.51E-05
ILMN_1767020	DENND1C	-2.85E-01	1.51E-05	1.79E-04
ILMN_3290380	LOC387703	-2.85E-01	2.67E-05	2.90E-04
ILMN_1723007	ZCCHC9	-2.85E-01	7.01E-04	4.82E-03
ILMN_1770892	YY1	-2.86E-01	1.01E-02	4.67E-02
ILMN_3257475	LOC100129866	-2.86E-01	8.21E-06	1.07E-04
ILMN_1889752		-2.86E-01	9.22E-05	8.37E-04
ILMN_1724863	TICAM1	-2.86E-01	6.39E-05	6.10E-04
ILMN_1669424	LOC646531	-2.86E-01	2.29E-03	1.33E-02
ILMN_1657204	SAE1	-2.86E-01	4.49E-05	4.50E-04
ILMN_1749907	LOC441241	-2.86E-01	1.07E-04	9.48E-04
ILMN_2414014	RBM10	-2.86E-01	1.45E-06	2.42E-05
ILMN_2328776	MST4	-2.86E-01	4.22E-03	2.25E-02
ILMN_1743137	BAT2D1	-2.86E-01	1.27E-03	8.02E-03
ILMN_1773561	RAP2C	-2.87E-01	1.26E-03	7.94E-03
ILMN_1772506	ATP5I	-2.87E-01	1.27E-05	1.54E-04
ILMN_1805863	WDR81	-2.87E-01	2.06E-04	1.68E-03
ILMN_1687998	LPGAT1	-2.87E-01	1.87E-06	3.02E-05
ILMN_3273229	LOC100129781	-2.87E-01	2.42E-04	1.93E-03
ILMN_1753755	B4GALT2	-2.87E-01	1.60E-04	1.35E-03
ILMN_1657744	C15orf17	-2.87E-01	4.16E-04	3.09E-03
ILMN_3290800	LOC647081	-2.87E-01	1.12E-06	1.96E-05
ILMN_2181432	SPC24	-2.87E-01	2.22E-05	2.49E-04
ILMN_1652735	RFXAP	-2.88E-01	3.18E-04	2.45E-03
ILMN_1741017	PIP4K2B	-2.88E-01	1.27E-04	1.10E-03
ILMN_1786834	PRKX	-2.88E-01	2.97E-03	1.66E-02
ILMN_1669286	YWHAZ	-2.88E-01	5.22E-05	5.12E-04
ILMN_2236800	SON	-2.88E-01	2.05E-05	2.32E-04
ILMN_1820787		-2.88E-01	4.34E-03	2.30E-02
ILMN_1795383	RPUSD3	-2.88E-01	7.59E-04	5.15E-03
ILMN_1745415	BBX	-2.88E-01	5.15E-04	3.71E-03
ILMN_1752435	SEC22C	-2.88E-01	1.24E-06	2.14E-05
ILMN_1718830	NARG2	-2.88E-01	3.83E-04	2.87E-03

ILMN_1756590	SYS1	-2.88E-01	2.16E-04	1.75E-03
ILMN_3176746	LOC100128191	-2.88E-01	1.03E-04	9.15E-04
ILMN_1779214	ATM	-2.89E-01	1.50E-03	9.22E-03
ILMN_2339863	VPS28	-2.89E-01	3.09E-05	3.29E-04
ILMN_1734410	BNIP1	-2.89E-01	1.85E-05	2.12E-04
ILMN_2181445	BCL2L13	-2.89E-01	7.95E-04	5.37E-03
ILMN_2351611	UBQLN1	-2.89E-01	2.16E-08	7.65E-07
ILMN_3310113	MIR586	-2.89E-01	2.42E-04	1.93E-03
ILMN_1809607	PPIF	-2.89E-01	7.29E-04	4.99E-03
ILMN_1726289	C12orf35	-2.89E-01	3.20E-04	2.46E-03
ILMN_1718093	ALG1	-2.89E-01	1.93E-06	3.09E-05
ILMN_1670948	TSR2	-2.90E-01	4.05E-06	5.90E-05
ILMN_1793302	WDR4	-2.90E-01	5.08E-05	5.01E-04
ILMN_2363250	BCL2	-2.90E-01	4.41E-05	4.43E-04
ILMN_1721316	TNFRSF10A	-2.90E-01	3.75E-05	3.87E-04
ILMN_3247802	BAT2L	-2.90E-01	1.96E-05	2.23E-04
ILMN_1710962	TMEM97	-2.90E-01	3.98E-03	2.14E-02
ILMN_1784977	DOHH	-2.90E-01	2.96E-05	3.17E-04
ILMN_1787477	CENPO	-2.90E-01	9.07E-06	1.16E-04
ILMN_1735453	FAM98A	-2.90E-01	1.07E-03	6.89E-03
ILMN_1678517	ACSL5	-2.90E-01	2.01E-07	4.71E-06
ILMN_2171289	SAMSN1	-2.90E-01	3.47E-04	2.64E-03
ILMN_1765122	MAP3K2	-2.90E-01	1.41E-05	1.68E-04
ILMN_1772370	ARHGEF1	-2.90E-01	1.07E-03	6.92E-03
ILMN_1716053	AK2	-2.90E-01	7.01E-03	3.45E-02
ILMN_1765085	TRMT6	-2.91E-01	3.82E-04	2.87E-03
ILMN_2306565	MTX2	-2.91E-01	4.92E-04	3.56E-03
ILMN_1721669	IDH3B	-2.91E-01	6.90E-07	1.31E-05
ILMN_3201658	LOC642585	-2.91E-01	1.31E-03	8.18E-03
ILMN_1742798	SFRS10	-2.91E-01	3.25E-03	1.80E-02
ILMN_1701229	RBM12	-2.91E-01	1.85E-04	1.53E-03
ILMN_1683927	ITGAE	-2.91E-01	9.84E-03	4.59E-02
ILMN_1801091	CERKL	-2.91E-01	4.89E-05	4.85E-04
ILMN_1724406	INO80E	-2.91E-01	5.38E-03	2.76E-02
ILMN_1809034	GATC	-2.91E-01	4.50E-06	6.45E-05
ILMN_2142979	PTBP2	-2.91E-01	2.55E-04	2.02E-03
ILMN_1732074	LOC648210	-2.91E-01	1.79E-03	1.07E-02
ILMN_1739045	LOC647009	-2.91E-01	4.41E-05	4.44E-04
ILMN_2364174	CSNK1A1	-2.91E-01	7.69E-07	1.43E-05
ILMN_1652819	OPA3	-2.91E-01	3.71E-05	3.83E-04
ILMN_1773742	DNAJB9	-2.91E-01	1.68E-03	1.02E-02
ILMN_1669201	ABCF2	-2.92E-01	3.85E-03	2.07E-02
ILMN_1745887	FBXO21	-2.92E-01	1.04E-04	9.24E-04
ILMN_1693108	RUVBL1	-2.92E-01	5.96E-03	3.01E-02
ILMN_1705111	FNDC3A	-2.92E-01	1.55E-04	1.31E-03
ILMN_2186369	NCOR1	-2.92E-01	1.02E-04	9.15E-04

ILMN_1788059	PCGF5	-2.92E-01	1.14E-03	7.29E-03
ILMN_2060105	PPAN-P2RY11	-2.92E-01	1.10E-07	2.87E-06
ILMN_1707240	PTBP2	-2.92E-01	1.21E-05	1.48E-04
ILMN_1659975	C1orf216	-2.92E-01	4.91E-05	4.87E-04
ILMN_2052790	NONO	-2.93E-01	1.49E-07	3.69E-06
ILMN_1777058	HIATL1	-2.93E-01	1.46E-06	2.44E-05
ILMN_1801118	C16orf33	-2.93E-01	7.43E-04	5.07E-03
ILMN_2135272	GIMAP2	-2.93E-01	3.03E-03	1.69E-02
ILMN_1661307	JRK	-2.93E-01	6.87E-08	1.92E-06
ILMN_1708936	EXOSC3	-2.93E-01	4.25E-07	8.71E-06
ILMN_1788886	TOX	-2.93E-01	1.03E-03	6.71E-03
ILMN_1734827	MKI67	-2.93E-01	3.82E-04	2.87E-03
ILMN_1738750	TFCP2	-2.93E-01	4.54E-04	3.33E-03
ILMN_3243011	LOC641844	-2.93E-01	2.56E-05	2.80E-04
ILMN_1748578	RAD21	-2.93E-01	1.97E-03	1.17E-02
ILMN_1717973	TMEM1	-2.93E-01	2.26E-04	1.82E-03
ILMN_1700604	RBM14	-2.93E-01	9.69E-05	8.72E-04
ILMN_1664718	CYP51A1	-2.94E-01	2.65E-03	1.51E-02
ILMN_2346358	TAF1	-2.94E-01	2.55E-05	2.79E-04
ILMN_1680010	KIAA0753	-2.94E-01	7.23E-05	6.80E-04
ILMN_2322986	MINA	-2.94E-01	1.40E-04	1.20E-03
ILMN_2094938	OMA1	-2.94E-01	2.82E-03	1.59E-02
ILMN_1672526	LOC389834	-2.95E-01	1.85E-04	1.53E-03
ILMN_3229733	SIGMAR1	-2.95E-01	5.97E-05	5.75E-04
ILMN_2085525	SNORA32	-2.95E-01	7.88E-04	5.33E-03
ILMN_1778173	AK3	-2.95E-01	1.07E-03	6.91E-03
ILMN_1667112	FBXO7	-2.95E-01	5.10E-06	7.15E-05
ILMN_1734229	SPPL2A	-2.95E-01	1.16E-03	7.41E-03
ILMN_1772486	ELF2	-2.95E-01	6.90E-05	6.53E-04
ILMN_2116075	TRUB2	-2.95E-01	1.06E-05	1.33E-04
ILMN_2396648	EXOSC1	-2.95E-01	4.66E-05	4.65E-04
ILMN_1775579	ACAD9	-2.95E-01	5.44E-07	1.08E-05
ILMN_1802761	LOC728505	-2.96E-01	9.21E-06	1.17E-04
ILMN_1698367	CD84	-2.96E-01	9.86E-04	6.46E-03
ILMN_1720926	PSMD5	-2.96E-01	4.36E-05	4.39E-04
ILMN_1745385	HNRPM	-2.96E-01	8.30E-06	1.08E-04
ILMN_2230592	MRPL3	-2.96E-01	2.08E-05	2.35E-04
ILMN_1814808	BFAR	-2.96E-01	2.34E-04	1.87E-03
ILMN_1790162	LOC441155	-2.96E-01	5.71E-06	7.86E-05
ILMN_2310253	TARBP2	-2.96E-01	4.95E-05	4.90E-04
ILMN_1742872	UBA2	-2.97E-01	3.91E-04	2.93E-03
ILMN_1672149	CHCHD1	-2.97E-01	3.16E-06	4.76E-05
ILMN_1777663	TOP2B	-2.97E-01	1.87E-04	1.54E-03
ILMN_1664030	RAB1B	-2.97E-01	1.12E-05	1.38E-04
ILMN_1663220	MRPL22	-2.97E-01	9.50E-05	8.58E-04
ILMN_1682399	CLOCK	-2.97E-01	4.04E-05	4.12E-04

ILMN_3244439	DDTL	-2.97E-01	1.18E-05	1.45E-04
ILMN_1796397	CISD2	-2.97E-01	5.20E-04	3.73E-03
ILMN_3215206	LOC100133836	-2.97E-01	7.85E-05	7.28E-04
ILMN_1779190	ALKBH8	-2.97E-01	9.46E-07	1.71E-05
ILMN_1782633	BOLA2	-2.97E-01	2.74E-04	2.15E-03
ILMN_1761996	SFRS5	-2.98E-01	2.92E-04	2.28E-03
ILMN_1768117	RBM25	-2.98E-01	1.54E-03	9.42E-03
ILMN_2196097	PPP2CA	-2.98E-01	1.06E-03	6.89E-03
ILMN_1801762	PFDN4	-2.98E-01	6.92E-04	4.78E-03
ILMN_2233604	ECSIT	-2.98E-01	3.51E-05	3.65E-04
ILMN_1750130	GSPT1	-2.98E-01	1.14E-03	7.29E-03
ILMN_1734138	TATDN2	-2.98E-01	3.79E-03	2.04E-02
ILMN_1798602	PCF11	-2.98E-01	1.15E-04	1.01E-03
ILMN_1733356	PREI3	-2.98E-01	1.67E-03	1.01E-02
ILMN_1779828	EDEM1	-2.98E-01	4.16E-04	3.09E-03
ILMN_3218820	LOC645387	-2.98E-01	6.62E-03	3.28E-02
ILMN_1680860	LEMD2	-2.98E-01	1.09E-05	1.35E-04
ILMN_2392674	PRR3	-2.98E-01	5.38E-04	3.85E-03
ILMN_1751530	GADD45GIP1	-2.98E-01	7.32E-05	6.87E-04
ILMN_3247783	MGC21881	-2.98E-01	8.41E-06	1.09E-04
ILMN_3307935	ATP10A	-2.98E-01	2.54E-04	2.01E-03
ILMN_3182942	LOC100129958	-2.98E-01	1.08E-07	2.83E-06
ILMN_1735004	C4orf43	-2.99E-01	2.87E-04	2.24E-03
ILMN_1736242	PLEKHG4	-2.99E-01	1.64E-04	1.37E-03
ILMN_1788955	PDLIM1	-2.99E-01	7.87E-05	7.30E-04
ILMN_1905310		-2.99E-01	1.04E-03	6.75E-03
ILMN_2411076	MATR3	-2.99E-01	2.73E-04	2.15E-03
ILMN_2394242	AMMECR1	-2.99E-01	1.73E-04	1.44E-03
ILMN_2103362	ARHGAP27	-2.99E-01	1.49E-04	1.27E-03
ILMN_2339284	CHD2	-2.99E-01	6.34E-03	3.16E-02
ILMN_1663090	SON	-2.99E-01	3.26E-05	3.44E-04
ILMN_1659952	MTMR2	-2.99E-01	1.78E-03	1.07E-02
ILMN_1796835	RWDD3	-2.99E-01	1.01E-05	1.27E-04
ILMN_1678605	CDC123	-2.99E-01	1.29E-03	8.10E-03
ILMN_1682197	NFXL1	-2.99E-01	8.71E-04	5.82E-03
ILMN_1735064	PPP1R3E	-2.99E-01	1.55E-04	1.31E-03
ILMN_1813423	NAT15	-3.00E-01	2.89E-04	2.26E-03
ILMN_1736995	C12orf66	-3.00E-01	1.65E-07	4.01E-06
ILMN_1894072		-3.00E-01	3.63E-06	5.36E-05
ILMN_1708537	RBPJ	-3.00E-01	3.84E-03	2.07E-02
ILMN_2114422	NOD1	-3.00E-01	1.30E-04	1.12E-03
ILMN_2112460	MAD2L1	-3.00E-01	4.20E-04	3.12E-03
ILMN_1798380	UBQLN1	-3.00E-01	9.29E-05	8.42E-04
ILMN_1802894	VKORC1L1	-3.00E-01	5.08E-05	5.01E-04
ILMN_1700967	C3orf59	-3.01E-01	1.71E-06	2.80E-05
ILMN_1760683	SFRS9	-3.01E-01	4.20E-06	6.07E-05

ILMN_1729157	LOC652903	-3.01E-01	5.38E-05	5.26E-04
ILMN_1680386	RPP38	-3.01E-01	1.25E-04	1.09E-03
ILMN_3241856	LOC100134301	-3.01E-01	2.01E-05	2.28E-04
ILMN_1749583	KIAA1285	-3.01E-01	9.11E-04	6.05E-03
ILMN_1710863	GATAD1	-3.02E-01	3.07E-07	6.68E-06
ILMN_1746375	CSNK2A1P	-3.02E-01	1.46E-06	2.44E-05
ILMN_2146766	FABP5	-3.02E-01	7.98E-03	3.84E-02
ILMN_1770732	COPS3	-3.02E-01	3.70E-06	5.46E-05
ILMN_1673544	C1orf83	-3.02E-01	4.32E-06	6.22E-05
ILMN_2089458	SASS6	-3.02E-01	3.24E-05	3.43E-04
ILMN_1753582	RPA2	-3.02E-01	7.08E-06	9.40E-05
ILMN_1768433	CCDC71	-3.03E-01	2.26E-04	1.82E-03
ILMN_1855430	LOC199800	-3.03E-01	6.37E-06	8.60E-05
ILMN_3269405	HNRNPM	-3.03E-01	6.14E-06	8.36E-05
ILMN_1709032	FYCO1	-3.03E-01	1.44E-06	2.42E-05
ILMN_1689162	ACTR8	-3.03E-01	4.50E-07	9.12E-06
ILMN_3251415	RBM43	-3.03E-01	1.31E-04	1.13E-03
ILMN_2410742	JMJD1C	-3.03E-01	8.33E-03	3.98E-02
ILMN_1795454	CPSF6	-3.03E-01	8.20E-06	1.07E-04
ILMN_1900270		-3.03E-01	8.64E-06	1.11E-04
ILMN_1669102	ATP5G2	-3.04E-01	1.34E-04	1.15E-03
ILMN_1746846	TTLL4	-3.04E-01	3.23E-07	6.98E-06
ILMN_1729130	C7orf42	-3.04E-01	4.12E-05	4.19E-04
ILMN_1703369	LOC647474	-3.04E-01	1.99E-04	1.63E-03
ILMN_1755024	IKBKE	-3.04E-01	3.10E-07	6.74E-06
ILMN_1669553	UBE2E3	-3.04E-01	2.75E-03	1.56E-02
ILMN_1660412	MGC27345	-3.04E-01	5.78E-04	4.10E-03
ILMN_1812474	TFG	-3.05E-01	6.84E-06	9.12E-05
ILMN_2124386	RGL2	-3.05E-01	2.41E-04	1.92E-03
ILMN_1802973	ANAPC4	-3.05E-01	3.81E-06	5.60E-05
ILMN_1770127	DNAJA2	-3.05E-01	2.42E-06	3.76E-05
ILMN_1685631	KIAA0892	-3.05E-01	4.43E-05	4.45E-04
ILMN_2090607	IRF2	-3.05E-01	4.61E-05	4.61E-04
ILMN_1680171	MFF	-3.05E-01	1.08E-04	9.56E-04
ILMN_1701006	HEXIM2	-3.06E-01	4.57E-03	2.40E-02
ILMN_3296923	LOC341784	-3.06E-01	1.34E-06	2.26E-05
ILMN_1753122	MCPH1	-3.06E-01	1.27E-05	1.54E-04
ILMN_1732550	KLHL23	-3.06E-01	1.90E-06	3.05E-05
ILMN_1657673	LOC90120	-3.06E-01	4.05E-03	2.17E-02
ILMN_1772700	TMEM18	-3.06E-01	7.07E-06	9.39E-05
ILMN_1798288	MOBKL2C	-3.06E-01	3.72E-04	2.80E-03
ILMN_2135232	SFRS3	-3.07E-01	3.11E-05	3.31E-04
ILMN_1671387	C3orf23	-3.07E-01	2.52E-05	2.76E-04
ILMN_1660125	SFMBT2	-3.07E-01	1.43E-04	1.22E-03
ILMN_2375557	SCMH1	-3.07E-01	7.23E-04	4.96E-03
ILMN_1775759	NRAS	-3.07E-01	2.85E-07	6.27E-06

ILMN_2388363	IMPDH1	-3.07E-01	2.92E-04	2.28E-03
ILMN_1654493	LOC649169	-3.07E-01	1.13E-05	1.40E-04
ILMN_1677877	UBE2L3	-3.07E-01	6.17E-04	4.33E-03
ILMN_1710220	LOC729985	-3.08E-01	2.07E-05	2.34E-04
ILMN_2391324	RCCD1	-3.08E-01	3.01E-06	4.56E-05
ILMN_1690706	SNRPB2	-3.08E-01	6.35E-09	2.90E-07
ILMN_1721989	ATP5F1	-3.08E-01	3.71E-07	7.81E-06
ILMN_1689652	RNMTL1	-3.08E-01	2.41E-05	2.66E-04
ILMN_1705447	AFG3L1	-3.08E-01	5.27E-04	3.78E-03
ILMN_1738677	PRPF8	-3.08E-01	9.65E-04	6.35E-03
ILMN_3234124	C17orf101	-3.08E-01	4.16E-05	4.22E-04
ILMN_1682694	LOC203547	-3.08E-01	2.80E-07	6.16E-06
ILMN_3239108	SNORA3	-3.08E-01	6.84E-05	6.49E-04
ILMN_1815134	PI4K2B	-3.09E-01	9.50E-03	4.46E-02
ILMN_2352401	ERBB2IP	-3.09E-01	8.27E-06	1.07E-04
ILMN_1782579	IMMT	-3.09E-01	2.83E-06	4.31E-05
ILMN_1866286		-3.09E-01	5.53E-06	7.64E-05
ILMN_1663866	TGFBI	-3.09E-01	3.98E-08	1.25E-06
ILMN_1808354	SLC4A7	-3.09E-01	1.30E-03	8.16E-03
ILMN_1804679	MYST1	-3.10E-01	1.86E-05	2.13E-04
ILMN_1835092		-3.10E-01	1.10E-03	7.11E-03
ILMN_1690085	STK11IP	-3.10E-01	3.41E-04	2.60E-03
ILMN_1808301	MRPL45	-3.10E-01	5.26E-06	7.34E-05
ILMN_2072603	MRPL14	-3.10E-01	1.34E-03	8.36E-03
ILMN_1752559	FBS1	-3.10E-01	1.32E-06	2.25E-05
ILMN_1728224	OGFR	-3.10E-01	2.39E-04	1.91E-03
ILMN_2126423	ZNF480	-3.11E-01	4.09E-03	2.19E-02
ILMN_1774380	LOC650898	-3.11E-01	9.58E-07	1.72E-05
ILMN_2096191	AASDHPPT	-3.11E-01	1.27E-05	1.53E-04
ILMN_1737312	SLC25A17	-3.11E-01	1.24E-06	2.13E-05
ILMN_1803853	NOL7	-3.11E-01	7.94E-06	1.04E-04
ILMN_2406043	VPS24	-3.11E-01	5.28E-06	7.35E-05
ILMN_1670821	CYorf15A	-3.11E-01	1.05E-03	6.81E-03
ILMN_1654671	SLMO1	-3.12E-01	9.93E-03	4.62E-02
ILMN_2131493	VISA	-3.12E-01	1.70E-06	2.78E-05
ILMN_1682120	THEM4	-3.12E-01	7.87E-04	5.32E-03
ILMN_3238803	RASAL3	-3.12E-01	1.34E-03	8.36E-03
ILMN_1766010	YARS	-3.12E-01	5.12E-05	5.04E-04
ILMN_1775939	SF3B2	-3.12E-01	4.71E-06	6.67E-05
ILMN_1691611	LOC645436	-3.12E-01	5.06E-03	2.62E-02
ILMN_1666325	ALG10B	-3.12E-01	1.56E-05	1.84E-04
ILMN_3306977	IMPDH1	-3.12E-01	1.80E-04	1.49E-03
ILMN_3233091	LOC100130224	-3.13E-01	3.90E-04	2.92E-03
ILMN_3289346	LOC442075	-3.13E-01	7.95E-03	3.83E-02
ILMN_1797903	ZNF544	-3.13E-01	1.75E-05	2.02E-04
ILMN_1781231	SLC25A38	-3.13E-01	6.09E-06	8.29E-05

ILMN_1804851	MRPS17	-3.13E-01	4.53E-03	2.38E-02
ILMN_1749502	ZNF215	-3.13E-01	5.93E-04	4.19E-03
ILMN_1743097	XRCC6	-3.13E-01	2.15E-03	1.26E-02
ILMN_1798014	EIF2S2	-3.13E-01	3.03E-03	1.69E-02
ILMN_1790575	METTL13	-3.13E-01	1.01E-02	4.68E-02
ILMN_1690476	QKI	-3.14E-01	1.09E-07	2.85E-06
ILMN_1813344	C20orf7	-3.14E-01	7.49E-05	7.00E-04
ILMN_1680805	IL28RA	-3.14E-01	6.33E-04	4.43E-03
ILMN_1815148	MAN2A2	-3.14E-01	4.40E-04	3.23E-03
ILMN_1792092	ZCCHC8	-3.14E-01	2.84E-04	2.23E-03
ILMN_1737947	LSM5	-3.14E-01	3.04E-05	3.24E-04
ILMN_2340565	ATP2C1	-3.14E-01	9.69E-06	1.22E-04
ILMN_1736828	CHST10	-3.14E-01	1.77E-06	2.88E-05
ILMN_3199658	LOC646626	-3.14E-01	4.88E-03	2.54E-02
ILMN_2366490	ZNF706	-3.15E-01	4.01E-06	5.84E-05
ILMN_1739586	FEZ2	-3.15E-01	3.18E-03	1.76E-02
ILMN_1797046	MTHFSD	-3.15E-01	2.55E-04	2.02E-03
ILMN_2242463	CTSC	-3.15E-01	3.73E-03	2.02E-02
ILMN_1803348	EHBP1	-3.15E-01	1.88E-06	3.02E-05
ILMN_1652003	GNG10	-3.15E-01	8.31E-03	3.98E-02
ILMN_1684789	CCDC101	-3.15E-01	3.65E-05	3.78E-04
ILMN_1738883	RNF135	-3.16E-01	9.01E-06	1.15E-04
ILMN_1672554	C17orf81	-3.16E-01	5.97E-08	1.72E-06
ILMN_1744914	FUCA2	-3.16E-01	3.90E-07	8.12E-06
ILMN_1805636	PGAP3	-3.16E-01	4.94E-04	3.57E-03
ILMN_3242603	GTF2H2B	-3.17E-01	3.23E-05	3.42E-04
ILMN_2386100	BUB3	-3.18E-01	1.14E-04	1.00E-03
ILMN_1733305	EIF2A	-3.18E-01	7.21E-06	9.55E-05
ILMN_1784218	DDX23	-3.18E-01	7.73E-05	7.20E-04
ILMN_1801553	LEO1	-3.18E-01	2.59E-05	2.83E-04
ILMN_2214603	PPP2R3C	-3.18E-01	2.64E-04	2.09E-03
ILMN_2083833	CNOT6L	-3.18E-01	3.84E-07	8.03E-06
ILMN_2374244	DYRK2	-3.18E-01	2.68E-06	4.11E-05
ILMN_1753597	FKRP	-3.18E-01	8.86E-07	1.61E-05
ILMN_1778161	DNAJC25	-3.18E-01	5.19E-04	3.73E-03
ILMN_2408450	UBE1DC1	-3.19E-01	8.06E-04	5.44E-03
ILMN_1688413	SNW1	-3.19E-01	1.77E-05	2.04E-04
ILMN_1751572	TLE1	-3.19E-01	2.51E-03	1.44E-02
ILMN_1652928	ORAI2	-3.19E-01	1.82E-06	2.95E-05
ILMN_3237721	TMCO7	-3.19E-01	4.93E-04	3.57E-03
ILMN_2196078	SLAMF6	-3.19E-01	3.36E-03	1.84E-02
ILMN_2144663	GUCY2C	-3.19E-01	3.30E-04	2.53E-03
ILMN_2111739	MAN2C1	-3.20E-01	5.28E-07	1.05E-05
ILMN_1685327	SON	-3.20E-01	2.29E-05	2.55E-04
ILMN_2058512	PSMA2	-3.20E-01	6.41E-04	4.48E-03
ILMN_1765547	IRF2	-3.20E-01	1.16E-06	2.03E-05

ILMN_1700028	C9orf156	-3.20E-01	6.17E-05	5.92E-04
ILMN_1702384	ZNF706	-3.20E-01	2.38E-07	5.41E-06
ILMN_2125675	LOC728643	-3.20E-01	1.23E-03	7.78E-03
ILMN_2285112	FBXO11	-3.21E-01	5.49E-04	3.92E-03
ILMN_1815121	PLAGL1	-3.21E-01	5.22E-08	1.55E-06
ILMN_2261882	KIAA0368	-3.21E-01	1.22E-04	1.06E-03
ILMN_1730765	DUSP22	-3.21E-01	2.30E-04	1.84E-03
ILMN_1815130	MICALL1	-3.21E-01	3.34E-05	3.51E-04
ILMN_1794349	XYLB	-3.21E-01	8.90E-05	8.11E-04
ILMN_1715947	LOC648210	-3.21E-01	1.06E-02	4.86E-02
ILMN_2331636	ACACA	-3.21E-01	2.00E-03	1.18E-02
ILMN_2112417	PGAM1	-3.21E-01	3.08E-03	1.71E-02
ILMN_1751816	MCTS1	-3.21E-01	1.20E-03	7.64E-03
ILMN_1656134	CNOT7	-3.21E-01	6.42E-06	8.65E-05
ILMN_3242011	MOBKL1B	-3.22E-01	1.71E-03	1.03E-02
ILMN_1702247	CCNDBP1	-3.22E-01	5.21E-03	2.69E-02
ILMN_1771738	ARL5A	-3.22E-01	2.02E-04	1.65E-03
ILMN_1665428	GSDMD	-3.22E-01	8.75E-03	4.16E-02
ILMN_1736752	COMTD1	-3.22E-01	1.45E-03	8.95E-03
ILMN_1726520	TDP1	-3.22E-01	2.48E-05	2.73E-04
ILMN_1736577	ZNF688	-3.22E-01	7.40E-06	9.76E-05
ILMN_1657332	CCDC85B	-3.22E-01	4.30E-03	2.28E-02
ILMN_1791770	SMARCC2	-3.22E-01	2.10E-04	1.71E-03
ILMN_1810147	ZNF524	-3.22E-01	2.67E-07	5.93E-06
ILMN_2296369	MATR3	-3.22E-01	4.73E-05	4.71E-04
ILMN_1749210	BUD13	-3.23E-01	4.45E-07	9.02E-06
ILMN_3239236	NCRNA00095	-3.23E-01	4.77E-06	6.75E-05
ILMN_1759801	DPP8	-3.23E-01	2.30E-05	2.55E-04
ILMN_1779735	C7orf59	-3.23E-01	8.28E-04	5.56E-03
ILMN_1737170	FLII	-3.23E-01	1.97E-06	3.14E-05
ILMN_2319344	APEX1	-3.23E-01	2.08E-05	2.35E-04
ILMN_2122374	FAM49B	-3.23E-01	2.70E-03	1.54E-02
ILMN_2324998	NGDN	-3.24E-01	2.14E-07	4.98E-06
ILMN_1684549	RNPC2	-3.24E-01	1.73E-07	4.16E-06
ILMN_2046896	ESRRAP2	-3.24E-01	1.91E-04	1.57E-03
ILMN_1692896	JMJD4	-3.24E-01	5.78E-06	7.94E-05
ILMN_1790797	VPS28	-3.24E-01	8.75E-06	1.12E-04
ILMN_1783598	CAB39L	-3.24E-01	7.77E-04	5.27E-03
ILMN_1728073	DENND1A	-3.24E-01	1.28E-03	8.03E-03
ILMN_1769409	C9orf123	-3.24E-01	3.13E-04	2.42E-03
ILMN_2174296	DNAJC2	-3.24E-01	2.78E-04	2.18E-03
ILMN_1839609		-3.24E-01	1.66E-05	1.94E-04
ILMN_1655194	PHF17	-3.24E-01	5.75E-05	5.57E-04
ILMN_2232430	NMD3	-3.24E-01	3.24E-03	1.79E-02
ILMN_1684746	IPO11	-3.24E-01	1.05E-07	2.77E-06
ILMN_1707783	CCDC72	-3.24E-01	2.87E-05	3.09E-04

ILMN_2212354	WDR46	-3.25E-01	5.23E-06	7.30E-05
ILMN_1795228	ZFAND5	-3.25E-01	4.41E-04	3.24E-03
ILMN_1659937	ZBTB24	-3.25E-01	1.67E-05	1.95E-04
ILMN_1757847	C11orf68	-3.25E-01	2.35E-05	2.60E-04
ILMN_1811328	DPP7	-3.25E-01	4.13E-03	2.21E-02
ILMN_1743049	PWP1	-3.25E-01	2.39E-06	3.72E-05
ILMN_1652085	MPHOSPH10	-3.26E-01	1.51E-06	2.51E-05
ILMN_2323418	KRIT1	-3.26E-01	3.76E-05	3.88E-04
ILMN_1747078	HYLS1	-3.26E-01	1.12E-06	1.95E-05
ILMN_1797698	RBM12	-3.26E-01	9.87E-04	6.47E-03
ILMN_2096604	NIP30	-3.26E-01	3.13E-05	3.33E-04
ILMN_1767892	DUSP12	-3.26E-01	1.04E-05	1.30E-04
ILMN_1736048	ELL	-3.26E-01	1.52E-03	9.30E-03
ILMN_1804148	TMED4	-3.27E-01	6.44E-07	1.23E-05
ILMN_1661485	RBM34	-3.27E-01	1.85E-03	1.11E-02
ILMN_3290261	LOC644877	-3.27E-01	2.20E-05	2.46E-04
ILMN_1796130	LOC221710	-3.27E-01	9.78E-08	2.61E-06
ILMN_3229033	LOC732360	-3.28E-01	5.58E-04	3.97E-03
ILMN_1741801	CDC7	-3.28E-01	9.83E-04	6.44E-03
ILMN_1730082	RPUSD4	-3.28E-01	2.95E-06	4.49E-05
ILMN_1670895	ZNF207	-3.28E-01	4.69E-06	6.66E-05
ILMN_3245559	CDK2AP1	-3.29E-01	1.06E-06	1.88E-05
ILMN_1710001	RPL41	-3.29E-01	3.15E-07	6.84E-06
ILMN_1683883	ACY1	-3.29E-01	9.56E-05	8.62E-04
ILMN_1736548	PHACTR4	-3.29E-01	1.41E-05	1.68E-04
ILMN_2398388	APH1A	-3.29E-01	2.84E-04	2.22E-03
ILMN_3192791	HNRNPM	-3.29E-01	2.54E-04	2.01E-03
ILMN_3236346	LOC100132901	-3.29E-01	1.24E-05	1.51E-04
ILMN_2175601	VDAC1	-3.29E-01	4.61E-03	2.42E-02
ILMN_1790943	LOC96597	-3.30E-01	3.66E-04	2.76E-03
ILMN_1782417	LOC651064	-3.30E-01	3.54E-06	5.25E-05
ILMN_2077733	C12orf30	-3.30E-01	3.17E-06	4.76E-05
ILMN_1744046	DIAPH2	-3.30E-01	1.55E-06	2.57E-05
ILMN_1718034	LOC441454	-3.30E-01	4.22E-05	4.27E-04
ILMN_1718808	AKAP10	-3.30E-01	3.58E-08	1.15E-06
ILMN_1686136	NSD1	-3.30E-01	5.91E-06	8.08E-05
ILMN_1715013	ERMAP	-3.31E-01	1.89E-07	4.47E-06
ILMN_1744611	WDSOF1	-3.31E-01	8.33E-03	3.99E-02
ILMN_1735199	CIAPIN1	-3.31E-01	1.10E-03	7.11E-03
ILMN_1773018	CUEDC2	-3.31E-01	2.34E-06	3.65E-05
ILMN_1664682	DNA2	-3.32E-01	6.41E-04	4.48E-03
ILMN_1806778	UBE2E1	-3.32E-01	8.51E-07	1.56E-05
ILMN_1760320	GNB1	-3.32E-01	4.73E-05	4.71E-04
ILMN_1799020	MUC12	-3.32E-01	7.22E-03	3.53E-02
ILMN_1814122	MDC1	-3.32E-01	4.56E-04	3.34E-03
ILMN_3202885	LOC643856	-3.33E-01	2.04E-05	2.31E-04

ILMN_2386354	CSNK2A1	-3.33E-01	1.79E-06	2.90E-05
ILMN_2134224	ATP13A1	-3.33E-01	6.09E-05	5.86E-04
ILMN_3270542	LOC100128525	-3.33E-01	6.35E-03	3.17E-02
ILMN_1798826	MRPS25	-3.33E-01	4.49E-06	6.44E-05
ILMN_2411559	PUS1	-3.33E-01	1.35E-03	8.39E-03
ILMN_1738027	BRCA1	-3.33E-01	7.46E-06	9.83E-05
ILMN_1776337	CHORDC1	-3.33E-01	1.32E-03	8.28E-03
ILMN_1677906	LOC643287	-3.33E-01	3.29E-03	1.82E-02
ILMN_1710979	ANKRD39	-3.34E-01	4.79E-04	3.48E-03
ILMN_1684402	STXBP5	-3.34E-01	5.27E-05	5.17E-04
ILMN_1676393	ATP5G1	-3.34E-01	6.02E-05	5.80E-04
ILMN_1685954	HMBS	-3.34E-01	3.27E-04	2.51E-03
ILMN_1683598	ACSL4	-3.34E-01	8.47E-05	7.77E-04
ILMN_2229922	C12orf35	-3.34E-01	4.57E-06	6.53E-05
ILMN_2355738	INCENP	-3.34E-01	4.44E-05	4.46E-04
ILMN_1667068	ZC3HAV1	-3.34E-01	1.19E-07	3.07E-06
ILMN_1717707	PSTK	-3.35E-01	4.96E-08	1.49E-06
ILMN_1704431	LOC554203	-3.35E-01	1.09E-06	1.92E-05
ILMN_1697286	SF3A1	-3.35E-01	3.80E-05	3.92E-04
ILMN_1709085	GSG2	-3.35E-01	9.21E-07	1.67E-05
ILMN_1695717	RBM41	-3.35E-01	2.43E-04	1.94E-03
ILMN_2154101	UPRT	-3.35E-01	8.84E-04	5.89E-03
ILMN_1683300	BAX	-3.36E-01	3.89E-04	2.91E-03
ILMN_1682953	PGAM4	-3.36E-01	2.00E-03	1.18E-02
ILMN_2341690	C17orf81	-3.36E-01	5.81E-06	7.97E-05
ILMN_1741564	DCTN4	-3.36E-01	2.30E-03	1.34E-02
ILMN_1699082	MAPKAPK5	-3.36E-01	3.96E-05	4.05E-04
ILMN_2407529	RNF135	-3.36E-01	8.04E-09	3.48E-07
ILMN_1746020	MDM4	-3.36E-01	7.96E-06	1.04E-04
ILMN_2179873	PHC3	-3.36E-01	4.26E-07	8.72E-06
ILMN_2341793	CCT7	-3.36E-01	5.72E-05	5.55E-04
ILMN_1720745	LOC645385	-3.36E-01	1.51E-04	1.28E-03
ILMN_1715616	PPIL5	-3.36E-01	2.18E-04	1.76E-03
ILMN_1780937	MUS81	-3.36E-01	1.04E-04	9.29E-04
ILMN_3250585	KIAA0194	-3.36E-01	1.80E-06	2.92E-05
ILMN_2110252	NPM3	-3.37E-01	4.08E-06	5.93E-05
ILMN_2368597	SMG7	-3.37E-01	1.75E-05	2.03E-04
ILMN_1708798	EAF2	-3.37E-01	8.88E-03	4.21E-02
ILMN_3237376	GRAMD1B	-3.38E-01	2.36E-05	2.61E-04
ILMN_1761722	ZNF579	-3.38E-01	3.49E-04	2.65E-03
ILMN_1701930	EEF1B2	-3.38E-01	2.48E-03	1.42E-02
ILMN_2227385	SLC16A14	-3.38E-01	8.25E-03	3.95E-02
ILMN_1753279	HNRNPA0	-3.38E-01	3.79E-05	3.90E-04
ILMN_1794132	NDUFS8	-3.38E-01	9.98E-05	8.94E-04
ILMN_1748819	MRPL22	-3.38E-01	1.36E-03	8.47E-03
ILMN_1815039	C6orf153	-3.39E-01	3.36E-07	7.21E-06

ILMN_1668540	ZNHIT6	-3.39E-01	1.15E-06	2.00E-05
ILMN_1755737	TRABD	-3.39E-01	5.99E-03	3.02E-02
ILMN_1671893	CHMP2A	-3.39E-01	5.54E-04	3.95E-03
ILMN_1737157	GRAMD1A	-3.39E-01	1.69E-03	1.02E-02
ILMN_1728163	CTDSP1	-3.39E-01	2.22E-06	3.49E-05
ILMN_1698189	AASDHPPT	-3.39E-01	5.65E-06	7.79E-05
ILMN_1718960	SERPINB8	-3.39E-01	9.07E-07	1.65E-05
ILMN_1723139	GPD2	-3.39E-01	8.11E-07	1.50E-05
ILMN_2387636	ITGB4BP	-3.40E-01	5.49E-04	3.92E-03
ILMN_2294751	ASCC3	-3.40E-01	1.56E-04	1.32E-03
ILMN_1767459	POLR3B	-3.40E-01	7.62E-03	3.70E-02
ILMN_3227811	LOC729423	-3.40E-01	1.12E-04	9.89E-04
ILMN_2149566	VPS25	-3.40E-01	6.03E-06	8.22E-05
ILMN_1680220	Jan-01	-3.40E-01	4.15E-05	4.21E-04
ILMN_1768279	NME6	-3.40E-01	7.37E-07	1.38E-05
ILMN_1694799	PIAS2	-3.41E-01	3.27E-05	3.45E-04
ILMN_2182750	DDX1	-3.41E-01	6.31E-06	8.54E-05
ILMN_1665442	NOL6	-3.41E-01	6.32E-07	1.21E-05
ILMN_3309468	MGC12982	-3.41E-01	6.40E-06	8.63E-05
ILMN_1662184	C5orf34	-3.41E-01	8.96E-05	8.16E-04
ILMN_1659682	GALK2	-3.41E-01	5.39E-04	3.86E-03
ILMN_1743145	ERAP2	-3.41E-01	1.25E-03	7.87E-03
ILMN_1773485	QKI	-3.41E-01	1.75E-06	2.86E-05
ILMN_1706553	SMG7	-3.41E-01	1.01E-06	1.80E-05
ILMN_1758806	C21orf2	-3.41E-01	1.48E-07	3.67E-06
ILMN_1780189	PSMC5	-3.42E-01	3.77E-07	7.92E-06
ILMN_2356068	CDC2L5	-3.42E-01	1.36E-05	1.63E-04
ILMN_1810941	COMT	-3.42E-01	2.13E-03	1.25E-02
ILMN_1795007	C2orf47	-3.42E-01	1.90E-04	1.56E-03
ILMN_2252309	DPP7	-3.42E-01	1.49E-04	1.26E-03
ILMN_3303965	ZC3H11B	-3.42E-01	1.57E-06	2.60E-05
ILMN_1664466	KLHL9	-3.42E-01	6.32E-03	3.15E-02
ILMN_1726906	AKT1S1	-3.42E-01	4.46E-03	2.35E-02
ILMN_1755023	RAD50	-3.42E-01	5.84E-04	4.13E-03
ILMN_1707088	DENND2D	-3.43E-01	7.79E-05	7.24E-04
ILMN_3265895	HNRNPR	-3.43E-01	5.85E-05	5.65E-04
ILMN_1697742	C3orf38	-3.43E-01	1.17E-04	1.03E-03
ILMN_1703301	LOC653479	-3.43E-01	1.54E-07	3.78E-06
ILMN_1759030	MAP4K5	-3.43E-01	2.44E-05	2.69E-04
ILMN_1664761	TMEM138	-3.43E-01	5.69E-06	7.83E-05
ILMN_3226810	LOC729666	-3.43E-01	3.86E-03	2.08E-02
ILMN_2166384	IPO5	-3.43E-01	8.11E-05	7.49E-04
ILMN_1660200	LOC652175	-3.43E-01	1.70E-03	1.03E-02
ILMN_1776073	CCT4	-3.44E-01	3.79E-05	3.90E-04
ILMN_2156786	PGGT1B	-3.44E-01	1.54E-07	3.78E-06
ILMN_2319913	DGKA	-3.44E-01	4.69E-05	4.68E-04

ILMN_1655497	EIF4B	-3.44E-01	1.37E-06	2.31E-05
ILMN_1815043	MRPS2	-3.44E-01	7.93E-03	3.83E-02
ILMN_1811006	E2F8	-3.44E-01	9.54E-06	1.21E-04
ILMN_1795561	CAMK1D	-3.44E-01	1.33E-05	1.60E-04
ILMN_1670870	ALCAM	-3.45E-01	4.42E-04	3.25E-03
ILMN_1781173	HDAC9	-3.45E-01	8.78E-03	4.17E-02
ILMN_1653529	TEX10	-3.45E-01	7.61E-07	1.42E-05
ILMN_1676191	DARS2	-3.45E-01	1.28E-07	3.26E-06
ILMN_2395285	U1SNRNPBP	-3.45E-01	9.48E-04	6.26E-03
ILMN_3247045	LOC100132442	-3.45E-01	6.85E-06	9.13E-05
ILMN_2362902	RASSF5	-3.45E-01	9.41E-04	6.22E-03
ILMN_1813207	MRPS9	-3.45E-01	1.02E-05	1.28E-04
ILMN_3237739	LOC100134468	-3.46E-01	7.26E-05	6.82E-04
ILMN_1754062	BEND3	-3.46E-01	5.96E-04	4.21E-03
ILMN_2174884	XPO7	-3.46E-01	1.03E-07	2.74E-06
ILMN_1753010	PET112L	-3.46E-01	6.09E-07	1.17E-05
ILMN_1664434	TCF3	-3.46E-01	1.28E-03	8.06E-03
ILMN_3237679	PTAR1	-3.46E-01	7.30E-05	6.85E-04
ILMN_3249949	C11orf58	-3.46E-01	8.73E-04	5.82E-03
ILMN_1794588	DYRK2	-3.46E-01	1.89E-04	1.55E-03
ILMN_1659960	IL4I1	-3.46E-01	4.37E-07	8.88E-06
ILMN_1703477	ARHGEF2	-3.47E-01	5.12E-05	5.04E-04
ILMN_2221006	RAD21	-3.47E-01	1.55E-04	1.31E-03
ILMN_1710136	HDHD1A	-3.47E-01	8.68E-06	1.12E-04
ILMN_2325185	TYSND1	-3.47E-01	2.08E-06	3.30E-05
ILMN_1768743	FIP1L1	-3.47E-01	3.85E-06	5.64E-05
ILMN_1776577	DSCC1	-3.47E-01	2.31E-04	1.85E-03
ILMN_2397028	SERPINB8	-3.47E-01	9.73E-06	1.23E-04
ILMN_2169856	C12orf43	-3.47E-01	1.77E-06	2.88E-05
ILMN_1698777	ADCK1	-3.47E-01	2.90E-03	1.63E-02
ILMN_1760315	VWCE	-3.47E-01	6.28E-04	4.40E-03
ILMN_1807994	PCNP	-3.47E-01	1.18E-06	2.04E-05
ILMN_2041327	MRPL37	-3.47E-01	1.67E-05	1.95E-04
ILMN_1751803	LSM10	-3.47E-01	1.54E-07	3.79E-06
ILMN_1791466	HMGB1	-3.47E-01	5.03E-03	2.61E-02
ILMN_1908989		-3.48E-01	2.17E-07	5.02E-06
ILMN_1753413	TRIOBP	-3.49E-01	2.59E-06	3.99E-05
ILMN_2350607	C20orf7	-3.49E-01	3.22E-04	2.48E-03
ILMN_1674703	UBE1DC1	-3.49E-01	5.55E-07	1.09E-05
ILMN_2157020	SNORD48	-3.49E-01	2.02E-03	1.19E-02
ILMN_2070300	LSM2	-3.49E-01	2.59E-04	2.05E-03
ILMN_2054442	ZNF146	-3.49E-01	2.17E-05	2.44E-04
ILMN_1726547	MAP3K5	-3.49E-01	1.24E-05	1.51E-04
ILMN_3219455	LOC644745	-3.49E-01	9.76E-03	4.56E-02
ILMN_1797184	PKN3	-3.49E-01	1.87E-05	2.15E-04
ILMN_1657682	GFM1	-3.49E-01	5.96E-06	8.14E-05

ILMN_2109708	ECGF1	-3.49E-01	4.34E-03	2.29E-02
ILMN_1758963	NADK	-3.50E-01	5.48E-03	2.80E-02
ILMN_1763404	LOC653226	-3.50E-01	5.64E-05	5.48E-04
ILMN_2257665	PARL	-3.50E-01	9.69E-05	8.72E-04
ILMN_2408400	NSUN5	-3.50E-01	1.09E-03	7.03E-03
ILMN_1794333	POU2F1	-3.50E-01	3.59E-04	2.71E-03
ILMN_3238511	LOC730020	-3.50E-01	4.13E-03	2.21E-02
ILMN_1756104	LARP7	-3.50E-01	2.79E-05	3.02E-04
ILMN_1747184	PUS7L	-3.50E-01	1.94E-03	1.15E-02
ILMN_1761068	MGC52000	-3.50E-01	8.31E-04	5.58E-03
ILMN_1743021	CAMKK2	-3.50E-01	6.22E-03	3.11E-02
ILMN_1694759	C19orf42	-3.50E-01	2.67E-06	4.10E-05
ILMN_3231881	LOC728026	-3.51E-01	8.19E-04	5.51E-03
ILMN_1809400	FAM49B	-3.51E-01	4.79E-04	3.48E-03
ILMN_2401701	PCGF6	-3.51E-01	2.78E-04	2.18E-03
ILMN_1762678	NMT1	-3.51E-01	1.26E-03	7.97E-03
ILMN_1807710	HINT1	-3.51E-01	3.24E-04	2.49E-03
ILMN_1652505	APEX2	-3.51E-01	9.80E-06	1.24E-04
ILMN_1712678	RPS27L	-3.51E-01	6.58E-06	8.84E-05
ILMN_1774779	LOC647121	-3.51E-01	1.22E-06	2.10E-05
ILMN_1815107	MATR3	-3.51E-01	4.16E-04	3.09E-03
ILMN_2312386	PAIP1	-3.51E-01	3.17E-05	3.36E-04
ILMN_3248521	UBXN8	-3.51E-01	6.83E-06	9.12E-05
ILMN_1706342	ZNF746	-3.51E-01	7.59E-07	1.42E-05
ILMN_1801600	CCDC97	-3.51E-01	8.57E-03	4.08E-02
ILMN_1740716	RBM26	-3.52E-01	1.30E-04	1.12E-03
ILMN_1778684	BRE	-3.52E-01	1.36E-04	1.17E-03
ILMN_2343624	METTL13	-3.52E-01	8.86E-03	4.20E-02
ILMN_1724422	SELL	-3.52E-01	9.59E-03	4.49E-02
ILMN_1736054	SUB1	-3.52E-01	1.71E-03	1.03E-02
ILMN_3201115	LOC440043	-3.52E-01	2.06E-03	1.21E-02
ILMN_1661416	LOC400958	-3.52E-01	4.29E-06	6.19E-05
ILMN_1667577	LCMT2	-3.53E-01	1.24E-05	1.51E-04
ILMN_1694223	DGCR8	-3.53E-01	1.93E-05	2.20E-04
ILMN_1810181	PCGF6	-3.53E-01	3.85E-07	8.04E-06
ILMN_1702065	MFSD5	-3.53E-01	1.96E-06	3.13E-05
ILMN_1789596	ETV6	-3.53E-01	2.60E-05	2.84E-04
ILMN_2140974	TPM4	-3.53E-01	4.85E-07	9.74E-06
ILMN_1783843	MIIP	-3.53E-01	1.56E-06	2.58E-05
ILMN_1757287	MAPK6	-3.53E-01	1.51E-07	3.72E-06
ILMN_3258136	LOC100130387	-3.53E-01	4.84E-06	6.83E-05
ILMN_1659054	CHKB	-3.54E-01	5.67E-07	1.11E-05
ILMN_1805344	DDX5	-3.54E-01	2.02E-03	1.19E-02
ILMN_1717639	SIK1	-3.54E-01	6.71E-04	4.65E-03
ILMN_1702541	CCDC55	-3.54E-01	2.49E-07	5.61E-06
ILMN_1659470	PBRM1	-3.54E-01	1.30E-07	3.29E-06

ILMN_1731048	TLR1	-3.54E-01	5.76E-08	1.67E-06
ILMN_1795524	C15orf44	-3.55E-01	4.38E-07	8.90E-06
ILMN_1656574	PCGF6	-3.55E-01	7.66E-09	3.34E-07
ILMN_1758679	TMEM168	-3.55E-01	2.10E-06	3.32E-05
ILMN_2252136	YWHAE	-3.55E-01	9.33E-04	6.17E-03
ILMN_1722905	MRPS11	-3.55E-01	4.19E-04	3.11E-03
ILMN_1734915	C1orf156	-3.55E-01	2.17E-08	7.69E-07
ILMN_2367530	ZNF280D	-3.56E-01	1.56E-07	3.83E-06
ILMN_1728934	PRC1	-3.56E-01	1.18E-06	2.05E-05
ILMN_1862001		-3.56E-01	1.20E-05	1.47E-04
ILMN_2104696	ERICH1	-3.56E-01	5.17E-05	5.08E-04
ILMN_1722774	VPS72	-3.56E-01	5.23E-07	1.04E-05
ILMN_3241099	FAM172A	-3.56E-01	1.72E-05	2.00E-04
ILMN_1689070	COQ7	-3.56E-01	1.31E-05	1.58E-04
ILMN_1653828	CHFR	-3.56E-01	4.14E-06	6.00E-05
ILMN_1661627	LZIC	-3.56E-01	4.84E-09	2.33E-07
ILMN_2348975	NASP	-3.56E-01	1.25E-03	7.90E-03
ILMN_1686610	APBA3	-3.56E-01	1.23E-04	1.07E-03
ILMN_1727618	C8orf38	-3.57E-01	6.39E-06	8.62E-05
ILMN_1791593	DENND5B	-3.57E-01	6.22E-03	3.12E-02
ILMN_1797172	ERCC1	-3.57E-01	6.57E-07	1.26E-05
ILMN_1807283	NDST1	-3.57E-01	1.64E-07	3.99E-06
ILMN_1802843	PRCC	-3.57E-01	2.59E-06	3.99E-05
ILMN_1743499	POLDIP2	-3.57E-01	1.43E-05	1.70E-04
ILMN_1764970	JMJD1C	-3.57E-01	4.98E-03	2.59E-02
ILMN_1748926	TMEM209	-3.57E-01	7.78E-06	1.02E-04
ILMN_3266894	LOC100128816	-3.57E-01	1.65E-08	6.19E-07
ILMN_2136455	C3orf64	-3.57E-01	2.76E-06	4.22E-05
ILMN_3250201	CNBP	-3.57E-01	2.46E-05	2.70E-04
ILMN_1659365	LOC653071	-3.57E-01	1.51E-04	1.28E-03
ILMN_1655645	AK2	-3.57E-01	4.54E-06	6.50E-05
ILMN_1705953	LONRF1	-3.58E-01	7.45E-04	5.08E-03
ILMN_1710668	LETM1	-3.58E-01	8.18E-06	1.06E-04
ILMN_1676938	LOC649214	-3.58E-01	1.03E-04	9.20E-04
ILMN_2247664	SON	-3.58E-01	9.08E-08	2.44E-06
ILMN_1781565	MESDC1	-3.58E-01	6.61E-07	1.26E-05
ILMN_1765082	RBM10	-3.58E-01	1.14E-05	1.41E-04
ILMN_2083588	TTC32	-3.59E-01	6.87E-03	3.39E-02
ILMN_1665289	FLJ22222	-3.59E-01	1.75E-03	1.05E-02
ILMN_1713406	FAM39DP	-3.59E-01	8.02E-03	3.86E-02
ILMN_1767801	SAMD10	-3.59E-01	4.63E-03	2.43E-02
ILMN_1744725	BTBD6	-3.60E-01	2.12E-06	3.35E-05
ILMN_1661886	APEX1	-3.60E-01	1.31E-06	2.22E-05
ILMN_1653613	PIGO	-3.60E-01	1.16E-06	2.02E-05
ILMN_2079803	LSM14A	-3.60E-01	6.07E-07	1.17E-05
ILMN_1769520	UBE2L6	-3.60E-01	1.60E-06	2.65E-05

ILMN_1696591	RB1	-3.60E-01	1.09E-02	4.98E-02
ILMN_1727142	IKBKB	-3.60E-01	1.61E-03	9.79E-03
ILMN_1689868	TMEM80	-3.60E-01	3.03E-05	3.23E-04
ILMN_1683415	CAMK2D	-3.60E-01	7.84E-06	1.03E-04
ILMN_1680955	AURKA	-3.61E-01	6.44E-08	1.82E-06
ILMN_1723846	FAM119B	-3.61E-01	1.60E-03	9.72E-03
ILMN_1726603	ATP5I	-3.61E-01	6.06E-07	1.17E-05
ILMN_1858599		-3.61E-01	5.62E-06	7.76E-05
ILMN_1766275	PIK3CD	-3.61E-01	1.03E-03	6.72E-03
ILMN_2307450	ZNF302	-3.62E-01	5.01E-04	3.62E-03
ILMN_1670931	PDS5A	-3.62E-01	9.76E-04	6.40E-03
ILMN_1782743	LETMD1	-3.62E-01	3.77E-04	2.84E-03
ILMN_2403946	FEZ2	-3.62E-01	1.44E-04	1.23E-03
ILMN_1774027	ZNF37A	-3.62E-01	1.81E-05	2.08E-04
ILMN_2050617	CCT6P1	-3.63E-01	2.05E-05	2.32E-04
ILMN_1764861	ISOC1	-3.63E-01	1.48E-03	9.13E-03
ILMN_2066667	RRP8	-3.63E-01	7.29E-06	9.65E-05
ILMN_1754149	LETMD1	-3.63E-01	1.91E-05	2.18E-04
ILMN_2192032	SRP19	-3.63E-01	1.49E-03	9.16E-03
ILMN_1751773	POLD3	-3.63E-01	3.87E-06	5.67E-05
ILMN_3258346	LOC100130009	-3.63E-01	1.02E-04	9.11E-04
ILMN_1756501	ST6GAL1	-3.63E-01	1.63E-05	1.91E-04
ILMN_1661335	SPTBN1	-3.63E-01	4.75E-04	3.46E-03
ILMN_1873300		-3.63E-01	2.90E-05	3.12E-04
ILMN_2415267	RREB1	-3.64E-01	3.00E-05	3.20E-04
ILMN_1712517	ZNF696	-3.64E-01	4.69E-05	4.67E-04
ILMN_1796063	TRIM44	-3.64E-01	1.96E-03	1.16E-02
ILMN_1794505	SHFM1	-3.64E-01	1.62E-04	1.36E-03
ILMN_1731354	PARL	-3.64E-01	2.85E-07	6.26E-06
ILMN_3243461	RPRD1B	-3.64E-01	8.31E-07	1.53E-05
ILMN_1789018	ILF3	-3.64E-01	1.67E-05	1.95E-04
ILMN_2381296	GSTZ1	-3.64E-01	8.04E-08	2.21E-06
ILMN_1701753	LOC644063	-3.64E-01	2.21E-03	1.29E-02
ILMN_3275672	LOC643873	-3.64E-01	5.70E-05	5.53E-04
ILMN_1655377	MRPS22	-3.64E-01	7.64E-04	5.18E-03
ILMN_3237562	SNORA76	-3.64E-01	3.85E-07	8.04E-06
ILMN_1654010	AGPAT3	-3.64E-01	6.31E-06	8.54E-05
ILMN_1783771	UBE2Z	-3.64E-01	2.77E-05	3.00E-04
ILMN_1798063	NLRP7	-3.64E-01	5.62E-03	2.86E-02
ILMN_1793203	SMCR7L	-3.64E-01	4.01E-03	2.15E-02
ILMN_1702265	HDHD2	-3.65E-01	1.96E-03	1.16E-02
ILMN_1763409	LRRC8D	-3.65E-01	2.75E-05	2.98E-04
ILMN_1671911	MTA1	-3.65E-01	1.99E-04	1.63E-03
ILMN_1719627	SLC27A3	-3.65E-01	9.57E-03	4.48E-02
ILMN_1663631	BANP	-3.65E-01	2.23E-04	1.80E-03
ILMN_2198376	PSMA4	-3.65E-01	1.66E-07	4.02E-06

ILMN_2120022	ARL5B	-3.65E-01	1.16E-06	2.02E-05
ILMN_1779663	TRMT2B	-3.65E-01	4.27E-07	8.73E-06
ILMN_1781986	UCRC	-3.65E-01	3.93E-05	4.02E-04
ILMN_1693430	NME1-NME2	-3.65E-01	1.45E-04	1.24E-03
ILMN_1680239	NUDT9	-3.66E-01	3.94E-07	8.21E-06
ILMN_1784630	KBTBD11	-3.66E-01	7.39E-05	6.91E-04
ILMN_1729318	TOR1AIP1	-3.66E-01	6.67E-09	3.00E-07
ILMN_1664488	C10orf64	-3.66E-01	5.35E-06	7.44E-05
ILMN_2393693	LRRC37A4	-3.66E-01	6.83E-06	9.12E-05
ILMN_1698968	ASXL2	-3.66E-01	6.38E-05	6.10E-04
ILMN_2115633	CD320	-3.67E-01	1.64E-03	9.94E-03
ILMN_2290628	IL16	-3.67E-01	1.53E-04	1.29E-03
ILMN_1730307	MED16	-3.67E-01	4.28E-07	8.75E-06
ILMN_3297996	LOC728732	-3.67E-01	2.14E-03	1.26E-02
ILMN_1759297	PATZ1	-3.67E-01	2.31E-05	2.56E-04
ILMN_1802631	AGA	-3.67E-01	2.91E-09	1.55E-07
ILMN_1706246	CCT5	-3.67E-01	6.06E-06	8.26E-05
ILMN_1793959	ADPGK	-3.68E-01	9.42E-05	8.52E-04
ILMN_1661428	ATP11C	-3.68E-01	2.74E-07	6.06E-06
ILMN_2082324	SLC36A4	-3.68E-01	7.24E-04	4.97E-03
ILMN_1745811	TDRD3	-3.68E-01	2.28E-04	1.83E-03
ILMN_1676173	PARVB	-3.68E-01	4.11E-03	2.20E-02
ILMN_1778321	SLC2A6	-3.68E-01	1.20E-04	1.05E-03
ILMN_1730658	YTHDF2	-3.68E-01	1.87E-04	1.54E-03
ILMN_3305339	UBA5	-3.68E-01	3.46E-08	1.11E-06
ILMN_1794612	UBA7	-3.68E-01	2.44E-03	1.40E-02
ILMN_1764891	ZNF384	-3.68E-01	5.09E-07	1.02E-05
ILMN_1812777	MRPL35	-3.69E-01	8.82E-06	1.13E-04
ILMN_2141807	C15orf23	-3.69E-01	1.14E-04	9.99E-04
ILMN_1727617	XRN2	-3.69E-01	6.22E-04	4.36E-03
ILMN_1701306	MRRF	-3.69E-01	9.14E-08	2.46E-06
ILMN_1754068	LOC643233	-3.69E-01	4.50E-05	4.51E-04
ILMN_1705629	STEAP1	-3.69E-01	1.52E-05	1.79E-04
ILMN_1882112		-3.69E-01	9.58E-08	2.57E-06
ILMN_2386016	C2orf28	-3.69E-01	5.83E-06	7.99E-05
ILMN_1771593	RRM1	-3.70E-01	2.40E-08	8.30E-07
ILMN_1682375	ATPBD3	-3.70E-01	2.93E-03	1.64E-02
ILMN_1710078	TMEM181	-3.70E-01	2.94E-04	2.29E-03
ILMN_3283592	LOC442609	-3.70E-01	1.39E-04	1.19E-03
ILMN_1686458	CEP152	-3.70E-01	3.64E-07	7.69E-06
ILMN_2077094	C11orf2	-3.70E-01	4.11E-07	8.49E-06
ILMN_3243291	LOC100133372	-3.70E-01	1.58E-05	1.85E-04
ILMN_3217276	LOC644517	-3.70E-01	2.28E-06	3.57E-05
ILMN_3273706	LOC100130131	-3.70E-01	1.05E-04	9.34E-04
ILMN_1810838	MTDH	-3.71E-01	7.45E-05	6.96E-04
ILMN_3235065	ZNHIT6	-3.71E-01	9.64E-06	1.22E-04

ILMN_1719656	MRPL38	-3.71E-01	3.70E-05	3.82E-04
ILMN_2381397	HSPD1	-3.71E-01	2.80E-03	1.58E-02
ILMN_2364852	BTN2A1	-3.71E-01	8.29E-03	3.97E-02
ILMN_1756220	DDX18	-3.71E-01	9.14E-07	1.66E-05
ILMN_3242205	GMPS	-3.71E-01	1.32E-04	1.14E-03
ILMN_3238269	NDUFA6	-3.71E-01	9.68E-05	8.72E-04
ILMN_1747241	IWS1	-3.71E-01	3.71E-06	5.48E-05
ILMN_1742889	WDR77	-3.71E-01	1.93E-04	1.58E-03
ILMN_1773968	SERBP1	-3.72E-01	1.23E-05	1.50E-04
ILMN_1777564	MAD2L1	-3.72E-01	2.65E-04	2.09E-03
ILMN_1810832	ZNF343	-3.72E-01	1.00E-04	8.99E-04
ILMN_2356895	MRPL42	-3.73E-01	3.70E-05	3.82E-04
ILMN_3187680	ACCS	-3.73E-01	8.95E-03	4.24E-02
ILMN_1700025	LOC732007	-3.73E-01	3.75E-03	2.03E-02
ILMN_1690252	ALKBH2	-3.73E-01	1.45E-04	1.24E-03
ILMN_1722491	APRT	-3.73E-01	9.86E-04	6.46E-03
ILMN_1747903	DNAJC11	-3.73E-01	5.23E-07	1.04E-05
ILMN_2391333	CYP20A1	-3.74E-01	6.90E-07	1.31E-05
ILMN_3257884	HMGXB4	-3.74E-01	2.83E-06	4.31E-05
ILMN_2357438	AURKA	-3.74E-01	2.78E-06	4.24E-05
ILMN_1753063	KIF15	-3.74E-01	4.72E-05	4.70E-04
ILMN_2165473	MID1IP1	-3.74E-01	1.02E-02	4.71E-02
ILMN_1674128	CWC22	-3.74E-01	5.36E-05	5.24E-04
ILMN_3269484	LOC100128899	-3.74E-01	9.96E-04	6.52E-03
ILMN_1669727	WAC	-3.75E-01	3.83E-06	5.62E-05
ILMN_2312732	DPP8	-3.75E-01	9.72E-07	1.75E-05
ILMN_2306661	UNC13C	-3.75E-01	4.74E-03	2.48E-02
ILMN_2383693	UPF2	-3.75E-01	1.06E-05	1.32E-04
ILMN_1691393	DNPEP	-3.76E-01	1.61E-03	9.79E-03
ILMN_2173975	RTP4	-3.76E-01	1.50E-04	1.27E-03
ILMN_1690586	HNRPA1P4	-3.76E-01	4.71E-05	4.69E-04
ILMN_3215712	LOC100131609	-3.76E-01	2.03E-05	2.30E-04
ILMN_2041046	CKS1B	-3.76E-01	2.67E-06	4.10E-05
ILMN_1773363	CIITA	-3.76E-01	8.07E-05	7.46E-04
ILMN_1757026	MANEA	-3.76E-01	5.30E-05	5.19E-04
ILMN_1694730	RNPEPL1	-3.76E-01	2.31E-08	8.05E-07
ILMN_1766045	SH3GLB1	-3.76E-01	4.31E-05	4.35E-04
ILMN_1769264	MCCC2	-3.76E-01	3.59E-05	3.72E-04
ILMN_1758090	BCCIP	-3.76E-01	8.67E-06	1.12E-04
ILMN_1746598	SCNM1	-3.77E-01	8.18E-07	1.51E-05
ILMN_1688178	RRP7A	-3.77E-01	3.42E-07	7.29E-06
ILMN_3307409	RREB1	-3.77E-01	1.77E-04	1.47E-03
ILMN_1712929	DNAJB12	-3.77E-01	1.33E-06	2.25E-05
ILMN_1760682	STAG3L1	-3.77E-01	7.59E-07	1.42E-05
ILMN_1814737	LNPEP	-3.77E-01	5.13E-03	2.65E-02
ILMN_2153280	KIAA0090	-3.78E-01	1.36E-06	2.30E-05

ILMN_1754179	AP1G2	-3.78E-01	8.55E-04	5.73E-03
ILMN_1717049	ZNF561	-3.78E-01	9.04E-07	1.64E-05
ILMN_2382558	SERF1A	-3.78E-01	2.13E-07	4.95E-06
ILMN_1762095	TMTC4	-3.78E-01	5.01E-04	3.62E-03
ILMN_1708427	KPNA3	-3.79E-01	1.16E-04	1.02E-03
ILMN_1753008	REXO1	-3.79E-01	2.30E-07	5.27E-06
ILMN_1820572		-3.79E-01	9.28E-06	1.18E-04
ILMN_1727444	C16orf53	-3.79E-01	3.28E-03	1.81E-02
ILMN_1746561	BCL2L2	-3.79E-01	2.69E-08	9.07E-07
ILMN_1805377	ZP3	-3.79E-01	8.77E-07	1.60E-05
ILMN_1774584	C2orf28	-3.79E-01	1.24E-04	1.08E-03
ILMN_2086417	NDUFV2	-3.79E-01	3.34E-04	2.56E-03
ILMN_2393763	ARPC4	-3.79E-01	8.32E-04	5.59E-03
ILMN_2363621	RBBP8	-3.80E-01	4.99E-05	4.93E-04
ILMN_2220283	HNRPA1L-2	-3.80E-01	1.89E-05	2.16E-04
ILMN_1779832	LOC400214	-3.80E-01	1.32E-05	1.59E-04
ILMN_1670576	IRF5	-3.80E-01	2.81E-03	1.59E-02
ILMN_2077896	TTTY15	-3.80E-01	7.97E-06	1.04E-04
ILMN_2318733	BNIP1	-3.80E-01	5.56E-07	1.10E-05
ILMN_1681304	PAN3	-3.80E-01	3.61E-09	1.85E-07
ILMN_1680831	BAZ1B	-3.80E-01	1.94E-05	2.21E-04
ILMN_1692225	DOK3	-3.80E-01	3.35E-03	1.84E-02
ILMN_3203801	LOC442041	-3.80E-01	1.17E-03	7.49E-03
ILMN_1671314	UXT	-3.81E-01	6.78E-05	6.44E-04
ILMN_1802627	PSMG3	-3.81E-01	5.56E-03	2.83E-02
ILMN_2347888	LARP4	-3.81E-01	2.96E-04	2.31E-03
ILMN_2148819	TUBA1A	-3.81E-01	2.74E-05	2.97E-04
ILMN_1661484	ZBTB45	-3.82E-01	4.06E-05	4.13E-04
ILMN_1668179	HNRNPF	-3.82E-01	1.72E-04	1.44E-03
ILMN_1789342	NDUFS2	-3.82E-01	1.34E-08	5.23E-07
ILMN_1704672	OBFC2B	-3.82E-01	1.91E-03	1.14E-02
ILMN_1691506	NGRN	-3.82E-01	5.68E-09	2.64E-07
ILMN_1675695	PDS5B	-3.82E-01	3.39E-07	7.24E-06
ILMN_1776879	HMGN2	-3.82E-01	5.93E-07	1.15E-05
ILMN_3185198	ARP11	-3.82E-01	3.11E-03	1.73E-02
ILMN_1691188	UIMC1	-3.82E-01	2.48E-05	2.72E-04
ILMN_1738938	TIMM8B	-3.82E-01	2.38E-05	2.63E-04
ILMN_1792951	ZHX2	-3.83E-01	9.22E-03	4.34E-02
ILMN_1659583	LOC644617	-3.83E-01	2.38E-05	2.63E-04
ILMN_1739810	RAI1	-3.83E-01	3.96E-04	2.96E-03
ILMN_1791569	PLXNA1	-3.83E-01	4.75E-03	2.48E-02
ILMN_3240418	SNORA72	-3.83E-01	1.40E-05	1.67E-04
ILMN_1679655	WDR82	-3.83E-01	1.12E-05	1.38E-04
ILMN_1652638	LRRC58	-3.83E-01	2.36E-05	2.61E-04
ILMN_1794046	MTX2	-3.83E-01	2.00E-07	4.70E-06
ILMN_1696347	CTSC	-3.83E-01	3.48E-04	2.65E-03

ILMN_1696713	POLA2	-3.83E-01	1.50E-03	9.22E-03
ILMN_1655635	METTL3	-3.83E-01	1.10E-04	9.76E-04
ILMN_1740927	LYRM4	-3.83E-01	1.17E-05	1.44E-04
ILMN_1741599	MEMO1	-3.84E-01	1.17E-05	1.44E-04
ILMN_1691559	ELF2	-3.84E-01	1.25E-04	1.09E-03
ILMN_1694603	SMARCC1	-3.84E-01	1.04E-04	9.25E-04
ILMN_1666597	PI4KB	-3.84E-01	6.10E-05	5.86E-04
ILMN_1708841	GOLPH3	-3.84E-01	1.36E-05	1.63E-04
ILMN_1652512	C2CD2	-3.85E-01	6.55E-04	4.55E-03
ILMN_1733453	LOC284988	-3.85E-01	1.05E-02	4.84E-02
ILMN_3272590	LOC100129975	-3.85E-01	1.89E-06	3.04E-05
ILMN_1725862	USP3	-3.85E-01	8.17E-06	1.06E-04
ILMN_1778890	PPIL5	-3.85E-01	1.41E-05	1.68E-04
ILMN_1795336	PTER	-3.85E-01	1.66E-04	1.39E-03
ILMN_1691570	METTL5	-3.85E-01	1.63E-04	1.36E-03
ILMN_1665205	ZNF260	-3.86E-01	1.25E-06	2.14E-05
ILMN_1763739	SOLH	-3.86E-01	4.25E-04	3.14E-03
ILMN_1706344	ABHD6	-3.86E-01	6.78E-04	4.69E-03
ILMN_1679995	MPP6	-3.86E-01	1.20E-05	1.47E-04
ILMN_1770356	POLRMT	-3.86E-01	2.64E-04	2.09E-03
ILMN_1807114	LOC255620	-3.86E-01	9.27E-05	8.39E-04
ILMN_2156936	SCCPDH	-3.86E-01	1.32E-04	1.14E-03
ILMN_1725787	RFX1	-3.86E-01	1.45E-04	1.24E-03
ILMN_3308663	MIR1228	-3.87E-01	1.20E-05	1.47E-04
ILMN_1751362	FASTKD1	-3.87E-01	2.90E-03	1.63E-02
ILMN_1805396	LOC168474	-3.87E-01	3.78E-07	7.92E-06
ILMN_2187727	NOC3L	-3.87E-01	6.59E-06	8.85E-05
ILMN_3247139	C17orf96	-3.87E-01	4.29E-03	2.28E-02
ILMN_1675542	LOC729148	-3.87E-01	5.07E-04	3.66E-03
ILMN_3243142	KAT2B	-3.87E-01	2.60E-04	2.06E-03
ILMN_1706386	SLC39A4	-3.87E-01	6.79E-04	4.70E-03
ILMN_3185092	LOC100128760	-3.87E-01	1.35E-08	5.25E-07
ILMN_1801257	CENPA	-3.87E-01	6.82E-06	9.11E-05
ILMN_2191822	ALG14	-3.88E-01	1.13E-03	7.25E-03
ILMN_2208413	ARHGAP15	-3.88E-01	7.66E-03	3.72E-02
ILMN_1756696	USF2	-3.88E-01	3.30E-03	1.82E-02
ILMN_2205050	PRKX	-3.88E-01	6.52E-07	1.25E-05
ILMN_1796855	TIAL1	-3.88E-01	2.86E-04	2.23E-03
ILMN_1737685	CRLS1	-3.88E-01	2.42E-06	3.76E-05
ILMN_2147503	ALG13	-3.88E-01	1.69E-05	1.97E-04
ILMN_1756942	SP3	-3.88E-01	5.13E-05	5.05E-04
ILMN_2129349	TSSC1	-3.88E-01	8.71E-06	1.12E-04
ILMN_3305949	LOC730246	-3.89E-01	7.54E-08	2.08E-06
ILMN_2388539	C17orf101	-3.89E-01	3.52E-06	5.23E-05
ILMN_1783684	LOC648695	-3.89E-01	4.13E-05	4.20E-04
ILMN_1748077	DDX59	-3.89E-01	8.86E-04	5.90E-03

ILMN_1697088	ARMC5	-3.89E-01	7.69E-07	1.43E-05
ILMN_1690822	VAPA	-3.89E-01	1.66E-03	1.01E-02
ILMN_2139035	CASD1	-3.89E-01	9.74E-05	8.76E-04
ILMN_1658439	C5orf44	-3.90E-01	6.85E-07	1.30E-05
ILMN_1753607	PNO1	-3.90E-01	4.32E-05	4.36E-04
ILMN_1657129	SKAP2	-3.90E-01	2.22E-05	2.49E-04
ILMN_1811188	BCCIP	-3.90E-01	2.70E-06	4.13E-05
ILMN_1761086	VPS54	-3.90E-01	6.06E-07	1.17E-05
ILMN_1761939	TIPIN	-3.90E-01	1.38E-08	5.33E-07
ILMN_1815924	NUP107	-3.90E-01	2.54E-05	2.78E-04
ILMN_1771651	MON1B	-3.90E-01	8.54E-07	1.56E-05
ILMN_1750008	SUPV3L1	-3.91E-01	1.48E-06	2.47E-05
ILMN_2372379	MGA	-3.91E-01	2.21E-07	5.11E-06
ILMN_1719403	MTX1	-3.91E-01	1.33E-05	1.60E-04
ILMN_2181089	VPRBP	-3.91E-01	2.94E-07	6.42E-06
ILMN_1710756	ENO1	-3.91E-01	8.20E-03	3.93E-02
ILMN_1791002	SKP2	-3.91E-01	5.27E-06	7.35E-05
ILMN_3309021	MIR142	-3.91E-01	2.34E-03	1.36E-02
ILMN_3241139	SNORD57	-3.91E-01	1.87E-04	1.54E-03
ILMN_1670801	MTR	-3.91E-01	3.88E-04	2.91E-03
ILMN_1756146	WDR45	-3.91E-01	3.20E-06	4.80E-05
ILMN_1794781	VAV2	-3.92E-01	7.96E-04	5.38E-03
ILMN_1796305	UPF2	-3.92E-01	1.75E-05	2.03E-04
ILMN_2370910	RAD51L3	-3.92E-01	2.32E-06	3.63E-05
ILMN_1799744	GALC	-3.92E-01	7.89E-07	1.46E-05
ILMN_1711543	C14orf169	-3.93E-01	1.29E-04	1.11E-03
ILMN_2109526	CSNK2A1P	-3.93E-01	8.23E-07	1.51E-05
ILMN_2318011	PSMA3	-3.93E-01	1.05E-02	4.83E-02
ILMN_1764090	AK3L1	-3.93E-01	4.84E-04	3.51E-03
ILMN_1799667	KIF4A	-3.93E-01	1.95E-05	2.22E-04
ILMN_1721008	DUT	-3.93E-01	1.53E-07	3.77E-06
ILMN_1697614	NHP2L1	-3.94E-01	3.70E-03	2.01E-02
ILMN_1781999	ABCF2	-3.94E-01	5.71E-03	2.90E-02
ILMN_3234436	LOC100132528	-3.94E-01	1.90E-03	1.13E-02
ILMN_2100209	CCL4L1	-3.94E-01	1.74E-04	1.44E-03
ILMN_3304022	LOC729102	-3.94E-01	4.81E-07	9.67E-06
ILMN_2302075	MSI2	-3.94E-01	4.02E-07	8.34E-06
ILMN_1729546	C19orf54	-3.94E-01	3.56E-04	2.70E-03
ILMN_2175265	TMEM11	-3.95E-01	6.71E-09	3.00E-07
ILMN_2394264	UBTF	-3.95E-01	1.20E-06	2.08E-05
ILMN_1811261	KRIT1	-3.95E-01	7.87E-05	7.30E-04
ILMN_1729095	PDZD2	-3.95E-01	4.66E-07	9.41E-06
ILMN_1766613	ZNF121	-3.95E-01	6.88E-05	6.51E-04
ILMN_1803317	C9orf114	-3.95E-01	4.35E-04	3.20E-03
ILMN_1699636	ACIN1	-3.95E-01	1.51E-04	1.28E-03
ILMN_1777565	TAP2	-3.96E-01	1.22E-06	2.11E-05

ILMN_3244963	WDR42A	-3.96E-01	1.03E-05	1.28E-04
ILMN_1682098	PSMA4	-3.96E-01	3.12E-08	1.02E-06
ILMN_1889555		-3.96E-01	3.08E-05	3.28E-04
ILMN_1740903	C7orf49	-3.96E-01	6.01E-06	8.20E-05
ILMN_1813572	IL16	-3.97E-01	4.30E-05	4.34E-04
ILMN_1813766	RCL1	-3.97E-01	6.36E-08	1.81E-06
ILMN_1713147	MCRS1	-3.97E-01	5.59E-09	2.60E-07
ILMN_1685480	TARS	-3.98E-01	3.86E-05	3.97E-04
ILMN_1712530	AKAP1	-3.98E-01	6.01E-04	4.24E-03
ILMN_1660869	LOC643438	-3.98E-01	1.46E-05	1.73E-04
ILMN_1780533	RNASE6	-3.98E-01	3.03E-06	4.59E-05
ILMN_1695853	CLK4	-3.98E-01	1.09E-06	1.92E-05
ILMN_1730685	MRPL16	-3.98E-01	3.66E-07	7.72E-06
ILMN_1764168	STXBP3	-3.98E-01	1.17E-08	4.71E-07
ILMN_1737254	USP1	-3.98E-01	3.16E-07	6.85E-06
ILMN_3238613	SNORA26	-3.99E-01	3.09E-04	2.39E-03
ILMN_1743032	CTSS	-3.99E-01	4.68E-06	6.65E-05
ILMN_1738326	EIF4E2	-3.99E-01	3.57E-05	3.71E-04
ILMN_1697962	NSMCE1	-3.99E-01	8.17E-05	7.53E-04
ILMN_2278235	CTBP1	-3.99E-01	3.32E-06	4.96E-05
ILMN_2378081	BCL7A	-3.99E-01	4.75E-07	9.56E-06
ILMN_1745962	FBXO7	-4.00E-01	6.82E-03	3.37E-02
ILMN_1730572	HNRPDL	-4.00E-01	2.58E-05	2.82E-04
ILMN_1781721	DDX31	-4.00E-01	3.07E-06	4.64E-05
ILMN_1736546	SLC16A14	-4.00E-01	1.08E-03	6.99E-03
ILMN_1799488	ZNF383	-4.00E-01	4.77E-06	6.74E-05
ILMN_1660757	LOC644422	-4.00E-01	4.03E-05	4.11E-04
ILMN_3229606	LOC729406	-4.01E-01	5.35E-06	7.44E-05
ILMN_1733311	PIGB	-4.01E-01	4.65E-04	3.39E-03
ILMN_1765044	CUTC	-4.01E-01	4.19E-05	4.25E-04
ILMN_3238078	SNORA45	-4.01E-01	5.65E-04	4.01E-03
ILMN_1745620	KRCC1	-4.01E-01	6.76E-07	1.29E-05
ILMN_1796005	TERT	-4.01E-01	9.91E-06	1.25E-04
ILMN_1749432	MRPL32	-4.01E-01	2.49E-03	1.43E-02
ILMN_3247424	ADAP1	-4.01E-01	1.39E-05	1.66E-04
ILMN_2330371	TATDN3	-4.02E-01	1.02E-02	4.74E-02
ILMN_1745772	ASCC3	-4.02E-01	1.24E-06	2.14E-05
ILMN_1695719	EIF2C2	-4.02E-01	1.31E-06	2.22E-05
ILMN_1734428	LSM12	-4.03E-01	4.28E-05	4.33E-04
ILMN_1695763	PDIA5	-4.03E-01	4.06E-06	5.90E-05
ILMN_1795856	LOC644935	-4.03E-01	1.12E-05	1.38E-04
ILMN_1812312	NDUFS4	-4.03E-01	1.87E-07	4.43E-06
ILMN_1750101	S100A11	-4.03E-01	7.39E-03	3.61E-02
ILMN_1809488	SPCS2	-4.03E-01	5.75E-03	2.92E-02
ILMN_1695658	KIF20A	-4.03E-01	1.66E-03	1.01E-02
ILMN_2397230	USP16	-4.03E-01	4.92E-08	1.48E-06

ILMN_1798249	AK3L1	-4.03E-01	2.77E-04	2.18E-03
ILMN_1751164	ARHGAP30	-4.04E-01	1.60E-06	2.64E-05
ILMN_1724410	USP46	-4.04E-01	1.10E-08	4.49E-07
ILMN_2321064	BAX	-4.04E-01	4.27E-03	2.27E-02
ILMN_3306742	SIGMAR1	-4.04E-01	2.49E-04	1.98E-03
ILMN_1705774	TIGD5	-4.05E-01	7.60E-04	5.16E-03
ILMN_1718537	HPS6	-4.05E-01	2.44E-05	2.68E-04
ILMN_2370414	CHD3	-4.05E-01	1.90E-06	3.05E-05
ILMN_1811104	KTELC1	-4.05E-01	8.00E-05	7.41E-04
ILMN_1717690	LOC650826	-4.05E-01	1.27E-05	1.53E-04
ILMN_1722522	CCNT2	-4.05E-01	4.63E-06	6.60E-05
ILMN_2217809	TMEM126A	-4.06E-01	5.50E-03	2.81E-02
ILMN_1727300	ZNF444	-4.06E-01	4.09E-06	5.94E-05
ILMN_1774074	RXRB	-4.06E-01	1.48E-05	1.75E-04
ILMN_1743299	PXMP3	-4.06E-01	4.53E-06	6.49E-05
ILMN_3283680	LOC345041	-4.06E-01	3.18E-04	2.45E-03
ILMN_2352609	OGG1	-4.06E-01	2.46E-05	2.70E-04
ILMN_1743340	CHST14	-4.06E-01	3.03E-05	3.24E-04
ILMN_1665630	LOC643872	-4.06E-01	4.87E-05	4.83E-04
ILMN_1859160		-4.06E-01	4.22E-05	4.27E-04
ILMN_3265343	LOC100130633	-4.07E-01	8.11E-07	1.50E-05
ILMN_2341467	PARL	-4.07E-01	6.72E-06	9.00E-05
ILMN_1763670	TM2D1	-4.07E-01	5.21E-08	1.55E-06
ILMN_2363489	BRE	-4.07E-01	2.97E-05	3.18E-04
ILMN_1750981	SLC25A26	-4.07E-01	7.60E-05	7.09E-04
ILMN_1784774	P2RY10	-4.07E-01	4.21E-03	2.24E-02
ILMN_1747598	PPP1R11	-4.08E-01	4.05E-09	2.03E-07
ILMN_1683538	CSDE1	-4.08E-01	4.85E-08	1.46E-06
ILMN_2336280	QKI	-4.08E-01	2.58E-05	2.81E-04
ILMN_1796682	PARP3	-4.08E-01	2.56E-06	3.94E-05
ILMN_1726809	BHLHB3	-4.08E-01	2.15E-07	4.98E-06
ILMN_1664630	CHEK1	-4.08E-01	1.91E-03	1.14E-02
ILMN_3251482	ALG10B	-4.08E-01	7.23E-07	1.36E-05
ILMN_2117904	ZNF22	-4.08E-01	2.85E-05	3.07E-04
ILMN_2379931	PIGO	-4.08E-01	9.52E-06	1.21E-04
ILMN_1697227	USP36	-4.08E-01	1.84E-04	1.52E-03
ILMN_1766221	B4GALT1	-4.08E-01	3.17E-03	1.76E-02
ILMN_1751901	TMEM163	-4.09E-01	4.32E-04	3.18E-03
ILMN_1658464	GTF3A	-4.09E-01	7.24E-05	6.81E-04
ILMN_1843949		-4.09E-01	1.19E-05	1.47E-04
ILMN_1688865	PPP1R9B	-4.09E-01	1.82E-07	4.33E-06
ILMN_1797530	CHCHD5	-4.09E-01	1.80E-07	4.29E-06
ILMN_1768480	VGLL4	-4.10E-01	9.76E-08	2.61E-06
ILMN_1684563	SPIN4	-4.10E-01	1.18E-05	1.45E-04
ILMN_1682323	DDX51	-4.11E-01	3.21E-06	4.82E-05
ILMN_3238945	LOC100134261	-4.11E-01	3.13E-08	1.02E-06

ILMN_1683888	SRP72	-4.11E-01	3.87E-05	3.98E-04
ILMN_1773620	SMARCC2	-4.11E-01	7.03E-05	6.64E-04
ILMN_1670542	AK2	-4.11E-01	5.28E-04	3.79E-03
ILMN_1717855	PFDN1	-4.11E-01	3.36E-07	7.21E-06
ILMN_1738095	PER2	-4.12E-01	1.83E-04	1.51E-03
ILMN_1771326	C15orf44	-4.12E-01	2.23E-06	3.51E-05
ILMN_1655482	TRIM27	-4.12E-01	8.75E-05	7.98E-04
ILMN_1801928	YWHAZ	-4.12E-01	4.20E-07	8.63E-06
ILMN_1679209	HSPA9	-4.12E-01	1.31E-03	8.18E-03
ILMN_1783497	PANK1	-4.13E-01	4.27E-07	8.73E-06
ILMN_1687724	RAP1GDS1	-4.13E-01	4.61E-05	4.61E-04
ILMN_1657708	MGLL	-4.13E-01	3.84E-06	5.63E-05
ILMN_1788160	KIF5B	-4.13E-01	6.35E-08	1.81E-06
ILMN_1726410	APRT	-4.13E-01	1.50E-04	1.27E-03
ILMN_2231189	HIAT1	-4.14E-01	2.26E-05	2.52E-04
ILMN_1768870	CAPZA2	-4.14E-01	2.24E-06	3.52E-05
ILMN_1735788	TRIOBP	-4.14E-01	2.99E-06	4.54E-05
ILMN_1750658	HAX1	-4.14E-01	2.96E-05	3.17E-04
ILMN_2368617	FKRP	-4.14E-01	3.86E-06	5.65E-05
ILMN_2278636	CUTL1	-4.14E-01	1.37E-06	2.31E-05
ILMN_1718177	CYP20A1	-4.14E-01	4.38E-08	1.34E-06
ILMN_1652008	C15orf23	-4.14E-01	1.05E-07	2.76E-06
ILMN_2141941	TOR1AIP1	-4.14E-01	1.43E-06	2.39E-05
ILMN_2398489	SIGMAR1	-4.14E-01	1.62E-04	1.36E-03
ILMN_2349138	CDC42SE1	-4.15E-01	2.12E-05	2.38E-04
ILMN_1713088	MSI2	-4.15E-01	3.47E-05	3.62E-04
ILMN_2045911	FBXO28	-4.15E-01	1.27E-04	1.10E-03
ILMN_1790282	NUDT19	-4.16E-01	1.03E-05	1.29E-04
ILMN_1784860	RFC3	-4.16E-01	6.07E-03	3.05E-02
ILMN_3242459	DCTPP1	-4.16E-01	9.38E-05	8.48E-04
ILMN_1747217	C15orf41	-4.16E-01	2.78E-05	3.01E-04
ILMN_1795063	ZADH2	-4.16E-01	4.01E-05	4.10E-04
ILMN_3251467	LRRC58	-4.16E-01	4.73E-07	9.52E-06
ILMN_1662147	MANEAL	-4.16E-01	9.39E-03	4.41E-02
ILMN_1764165	TRIM65	-4.17E-01	4.96E-03	2.58E-02
ILMN_1678087	MAP3K4	-4.17E-01	9.48E-05	8.57E-04
ILMN_1703108	UBE2L6	-4.17E-01	3.72E-05	3.84E-04
ILMN_2397231	USP16	-4.17E-01	2.45E-07	5.55E-06
ILMN_1881081		-4.17E-01	3.46E-05	3.61E-04
ILMN_2415529	CDK5RAP2	-4.18E-01	2.49E-05	2.73E-04
ILMN_1720513	SETBP1	-4.18E-01	5.33E-03	2.74E-02
ILMN_1765860	DOCK11	-4.18E-01	5.12E-03	2.65E-02
ILMN_1799381	SNORD14A	-4.18E-01	1.44E-06	2.41E-05
ILMN_1705849	SPR	-4.19E-01	2.09E-08	7.50E-07
ILMN_1701216	BANP	-4.19E-01	2.03E-07	4.77E-06
ILMN_1749930	TMEM48	-4.19E-01	9.88E-10	6.78E-08

ILMN_1794213	ABHD14A	-4.19E-01	1.05E-03	6.80E-03
ILMN_3221828	LOC728787	-4.20E-01	6.94E-06	9.24E-05
ILMN_1745593	STMN1	-4.20E-01	1.75E-06	2.85E-05
ILMN_1662741	EDG4	-4.20E-01	1.07E-02	4.91E-02
ILMN_1668185	ZNF282	-4.20E-01	5.95E-06	8.13E-05
ILMN_1815745	SOX4	-4.20E-01	2.67E-03	1.52E-02
ILMN_1727184	WDR36	-4.20E-01	4.58E-06	6.54E-05
ILMN_1660965	PRR22	-4.20E-01	1.27E-07	3.24E-06
ILMN_1789508	GTF3C3	-4.21E-01	8.25E-08	2.25E-06
ILMN_1723822	PBRM1	-4.21E-01	1.24E-05	1.51E-04
ILMN_1744628	FDX1L	-4.21E-01	1.71E-07	4.13E-06
ILMN_1715702	LOC653171	-4.21E-01	3.81E-08	1.21E-06
ILMN_1808634	TMEM77	-4.21E-01	2.63E-07	5.86E-06
ILMN_2302654	LRP8	-4.22E-01	4.00E-05	4.08E-04
ILMN_1762990	KIAA0141	-4.22E-01	4.00E-08	1.25E-06
ILMN_1791827	LOC731878	-4.22E-01	4.32E-05	4.35E-04
ILMN_2341952	MRPL35	-4.23E-01	2.82E-05	3.04E-04
ILMN_1803799	LOC649555	-4.23E-01	7.85E-05	7.28E-04
ILMN_1784031	TIMM44	-4.23E-01	6.40E-07	1.23E-05
ILMN_3266606	FABP5L2	-4.23E-01	3.01E-03	1.68E-02
ILMN_2298159	PRDM1	-4.23E-01	2.04E-04	1.66E-03
ILMN_1773031	TAPBP	-4.23E-01	5.06E-08	1.51E-06
ILMN_1805028	THOC7	-4.23E-01	2.66E-06	4.08E-05
ILMN_1811624	THADA	-4.23E-01	1.80E-05	2.07E-04
ILMN_2409220	HMMR	-4.24E-01	3.76E-04	2.83E-03
ILMN_1816713		-4.24E-01	1.20E-05	1.47E-04
ILMN_1763129	DCTPP1	-4.24E-01	1.93E-06	3.09E-05
ILMN_1690049	NGDN	-4.24E-01	1.73E-07	4.17E-06
ILMN_3240420	USP18	-4.24E-01	2.66E-04	2.10E-03
ILMN_3243677	SNORA73B	-4.24E-01	1.16E-03	7.44E-03
ILMN_1696975	USP1	-4.24E-01	2.63E-04	2.08E-03
ILMN_3236358	NOP14	-4.25E-01	4.98E-04	3.60E-03
ILMN_3243302	C8orf30B	-4.25E-01	1.07E-06	1.90E-05
ILMN_1696568	ATP2C1	-4.25E-01	1.31E-07	3.31E-06
ILMN_1689119	ZC3H5	-4.25E-01	6.86E-06	9.14E-05
ILMN_1707631	MED10	-4.25E-01	4.62E-08	1.40E-06
ILMN_1664516	CENPF	-4.25E-01	1.54E-06	2.55E-05
ILMN_1658802	KRTCAP2	-4.26E-01	2.12E-06	3.36E-05
ILMN_2054145	PAK1IP1	-4.26E-01	1.02E-04	9.15E-04
ILMN_1694923	PTPN9	-4.26E-01	8.31E-05	7.64E-04
ILMN_1791754	CPT1B	-4.26E-01	9.43E-07	1.70E-05
ILMN_1658472	APH1A	-4.27E-01	1.89E-04	1.56E-03
ILMN_1708627	LOC653226	-4.27E-01	5.96E-05	5.75E-04
ILMN_1788254	PGAM5	-4.27E-01	1.65E-06	2.71E-05
ILMN_3248562	SNORD49A	-4.27E-01	4.61E-03	2.42E-02
ILMN_1669598	SUV420H1	-4.27E-01	5.71E-08	1.66E-06

ILMN_1681675	RBM16	-4.27E-01	3.88E-05	3.98E-04
ILMN_1679476	GART	-4.28E-01	3.02E-06	4.57E-05
ILMN_1775444	FLJ12078	-4.28E-01	4.03E-06	5.87E-05
ILMN_1712687	PAK2	-4.28E-01	4.70E-03	2.46E-02
ILMN_1754045	FANCL	-4.28E-01	2.79E-07	6.16E-06
ILMN_1697827	ATP2A3	-4.28E-01	8.95E-05	8.15E-04
ILMN_3244574	KIAA1430	-4.29E-01	1.57E-07	3.85E-06
ILMN_1676848	LOC728844	-4.29E-01	1.27E-04	1.10E-03
ILMN_2349831	DICER1	-4.29E-01	2.81E-05	3.04E-04
ILMN_1758545	DNAJB12	-4.29E-01	4.44E-08	1.36E-06
ILMN_3233589	LOC728467	-4.30E-01	8.44E-06	1.09E-04
ILMN_3298582	LOC728873	-4.30E-01	2.24E-05	2.50E-04
ILMN_1746696	PDS5B	-4.30E-01	9.81E-06	1.24E-04
ILMN_2092041	PSCDBP	-4.30E-01	1.11E-04	9.83E-04
ILMN_1688725	UTP14A	-4.30E-01	2.06E-03	1.21E-02
ILMN_1662340	ZNF358	-4.30E-01	4.34E-06	6.24E-05
ILMN_1739749	B3GALT6	-4.30E-01	2.41E-04	1.92E-03
ILMN_1725981	LOC654189	-4.31E-01	5.49E-04	3.92E-03
ILMN_2222984	RDH14	-4.31E-01	4.58E-05	4.58E-04
ILMN_1746135	PHF23	-4.31E-01	3.35E-06	5.00E-05
ILMN_1797372	C3orf58	-4.31E-01	2.00E-08	7.25E-07
ILMN_1793578	ZFP37	-4.32E-01	1.01E-05	1.26E-04
ILMN_1666713	LYPLA1	-4.32E-01	1.03E-04	9.16E-04
ILMN_1691772	ZSCAN29	-4.32E-01	1.09E-04	9.66E-04
ILMN_3238735	LOC100132346	-4.32E-01	1.03E-04	9.16E-04
ILMN_1804884	C22orf39	-4.32E-01	1.53E-04	1.29E-03
ILMN_3235113	TOMM6	-4.32E-01	9.24E-05	8.38E-04
ILMN_1662617	PPP2R3C	-4.33E-01	5.14E-06	7.19E-05
ILMN_2374778	DUT	-4.33E-01	3.24E-04	2.49E-03
ILMN_3291472	LOC442727	-4.33E-01	9.77E-05	8.78E-04
ILMN_1720088	SFRS12	-4.33E-01	4.25E-07	8.71E-06
ILMN_1773760	PAICS	-4.33E-01	5.13E-06	7.18E-05
ILMN_1655935	ADCY7	-4.33E-01	1.18E-03	7.54E-03
ILMN_3239361	LOC100133298	-4.33E-01	2.97E-04	2.31E-03
ILMN_2217329	IAH1	-4.34E-01	2.54E-04	2.02E-03
ILMN_1665538	SKP2	-4.34E-01	2.77E-06	4.22E-05
ILMN_2286334	SR140	-4.34E-01	3.82E-05	3.94E-04
ILMN_1785660	SRPR	-4.34E-01	6.52E-09	2.95E-07
ILMN_2367428	FAM96A	-4.34E-01	2.15E-04	1.74E-03
ILMN_1723481	CHST3	-4.34E-01	7.49E-03	3.65E-02
ILMN_1669394	EI24	-4.35E-01	9.27E-05	8.40E-04
ILMN_1774336	POLE2	-4.35E-01	2.83E-03	1.59E-02
ILMN_2099594	SRP9	-4.35E-01	1.94E-05	2.21E-04
ILMN_1680770	UBQLN2	-4.35E-01	1.39E-06	2.34E-05
ILMN_1716237	ACOT2	-4.35E-01	1.19E-07	3.05E-06
ILMN_2199676	CEP152	-4.36E-01	1.72E-06	2.80E-05

ILMN_1896406		-4.36E-01	5.64E-09	2.62E-07
ILMN_1798533	ZNF22	-4.36E-01	8.62E-09	3.68E-07
ILMN_3251232	HMGN2	-4.36E-01	1.14E-06	1.99E-05
ILMN_1732688	DUT	-4.36E-01	6.07E-03	3.05E-02
ILMN_1798657	TBL1XR1	-4.36E-01	4.00E-07	8.30E-06
ILMN_2333319	PTBP1	-4.36E-01	4.37E-03	2.31E-02
ILMN_1666553	SLC25A19	-4.36E-01	6.08E-06	8.29E-05
ILMN_1775182	GSR	-4.37E-01	1.53E-05	1.80E-04
ILMN_1701512	KIAA0391	-4.37E-01	2.97E-05	3.18E-04
ILMN_2209748	DERL1	-4.37E-01	1.47E-04	1.25E-03
ILMN_1811102	LRSAM1	-4.37E-01	1.28E-07	3.25E-06
ILMN_1711617	GMFG	-4.37E-01	5.19E-04	3.73E-03
ILMN_1704139	DHRSX	-4.37E-01	1.15E-09	7.64E-08
ILMN_1781526	PPP1R8	-4.37E-01	6.26E-06	8.48E-05
ILMN_1658902	DEAF1	-4.38E-01	3.19E-07	6.91E-06
ILMN_1716766	CEBPG	-4.38E-01	3.47E-03	1.90E-02
ILMN_2332795	ZNF16	-4.38E-01	1.28E-07	3.25E-06
ILMN_2149053	RIF1	-4.38E-01	1.68E-10	1.82E-08
ILMN_1750029	GABPA	-4.38E-01	1.09E-04	9.62E-04
ILMN_1681741	C1orf31	-4.39E-01	1.31E-06	2.22E-05
ILMN_1793033	RBM28	-4.39E-01	4.36E-08	1.34E-06
ILMN_1788468	ARGLU1	-4.39E-01	1.03E-04	9.19E-04
ILMN_1795839	SCCPDH	-4.39E-01	5.40E-04	3.86E-03
ILMN_1770719	KIAA0664	-4.39E-01	4.26E-04	3.15E-03
ILMN_1760676	MORF4L1	-4.39E-01	1.05E-07	2.77E-06
ILMN_1707484	GEMIN6	-4.39E-01	1.51E-05	1.78E-04
ILMN_1763207	BATF3	-4.39E-01	8.94E-03	4.23E-02
ILMN_3248803	LOC729680	-4.39E-01	5.69E-05	5.53E-04
ILMN_1807945	ANP32A	-4.40E-01	1.06E-06	1.88E-05
ILMN_1771695	RCBTB2	-4.40E-01	4.28E-04	3.16E-03
ILMN_3289508	LOC339192	-4.40E-01	1.99E-05	2.26E-04
ILMN_1804958	ZFY	-4.40E-01	1.62E-05	1.90E-04
ILMN_1712587	ARSB	-4.40E-01	2.54E-07	5.70E-06
ILMN_2380850	SDCCAG3	-4.40E-01	4.92E-04	3.56E-03
ILMN_1813836	DARS	-4.40E-01	4.38E-08	1.34E-06
ILMN_2049303	DCI	-4.41E-01	3.06E-06	4.62E-05
ILMN_1729775	OPA1	-4.41E-01	4.32E-08	1.33E-06
ILMN_1823231		-4.41E-01	2.05E-08	7.40E-07
ILMN_1684553	RHOH	-4.41E-01	2.27E-05	2.53E-04
ILMN_2085760	ARPC1B	-4.41E-01	3.48E-03	1.90E-02
ILMN_2405324	IL28RA	-4.41E-01	6.73E-03	3.33E-02
ILMN_1807535	YWHAE	-4.41E-01	6.32E-06	8.55E-05
ILMN_2123871	TMEM18	-4.42E-01	2.42E-08	8.34E-07
ILMN_1724789	CD59	-4.42E-01	3.48E-05	3.63E-04
ILMN_1734826	NUP88	-4.42E-01	1.63E-06	2.68E-05
ILMN_1741459	CDK10	-4.42E-01	2.42E-06	3.76E-05

ILMN_1724341	CXorf45	-4.42E-01	4.68E-06	6.65E-05
ILMN_1689720	PPP2R3B	-4.42E-01	4.75E-03	2.48E-02
ILMN_1683273	SNAPC5	-4.42E-01	2.12E-05	2.39E-04
ILMN_1691930	CBX6	-4.42E-01	1.26E-03	7.97E-03
ILMN_1864422		-4.43E-01	4.54E-04	3.32E-03
ILMN_2231021	TMEM185B	-4.43E-01	1.78E-05	2.05E-04
ILMN_1813834	PRMT6	-4.43E-01	2.56E-07	5.74E-06
ILMN_1665730	ABCB10	-4.43E-01	1.44E-08	5.55E-07
ILMN_1746025	MINA	-4.44E-01	2.75E-05	2.98E-04
ILMN_1733276	CYSLTR1	-4.44E-01	2.44E-06	3.78E-05
ILMN_1736340	ANGEL2	-4.44E-01	2.20E-07	5.10E-06
ILMN_1768393	SNRPD1	-4.44E-01	2.93E-05	3.14E-04
ILMN_1671932	SAMM50	-4.44E-01	8.06E-09	3.48E-07
ILMN_2230862	GYG1	-4.45E-01	6.80E-04	4.70E-03
ILMN_2147435	MAN2A1	-4.45E-01	7.32E-04	5.01E-03
ILMN_2200917	SLC4A7	-4.45E-01	2.01E-04	1.64E-03
ILMN_1736176	PLK1	-4.45E-01	3.21E-07	6.95E-06
ILMN_1795181	DDX60	-4.45E-01	5.13E-03	2.65E-02
ILMN_1812616	MYO1C	-4.45E-01	5.32E-06	7.40E-05
ILMN_1689704	TMEM5	-4.45E-01	2.87E-07	6.29E-06
ILMN_2362545	ZWINT	-4.46E-01	1.10E-08	4.47E-07
ILMN_2397954	PARP3	-4.46E-01	1.21E-05	1.49E-04
ILMN_1774823	RPL34	-4.46E-01	3.96E-05	4.05E-04
ILMN_1841970		-4.46E-01	6.19E-09	2.84E-07
ILMN_1696302	FABP5	-4.47E-01	1.41E-03	8.77E-03
ILMN_2352295	PRDM10	-4.47E-01	1.96E-04	1.60E-03
ILMN_3240022	SNORA73A	-4.47E-01	1.08E-06	1.90E-05
ILMN_1733407	QTRTD1	-4.48E-01	8.28E-05	7.62E-04
ILMN_2362549	ZWINT	-4.48E-01	5.57E-07	1.10E-05
ILMN_1685928	WDR34	-4.48E-01	1.36E-04	1.17E-03
ILMN_1797764	RPL22L1	-4.48E-01	1.15E-04	1.01E-03
ILMN_1813236	C6orf136	-4.48E-01	4.93E-04	3.57E-03
ILMN_1795906	PCBD1	-4.49E-01	2.00E-05	2.27E-04
ILMN_1679798	TLR9	-4.49E-01	5.41E-03	2.77E-02
ILMN_1752111	SMARCAL1	-4.49E-01	2.12E-08	7.53E-07
ILMN_1696276	ZNF653	-4.49E-01	1.65E-05	1.93E-04
ILMN_1705297	MYBPH	-4.49E-01	2.59E-05	2.83E-04
ILMN_1708954	GAR1	-4.50E-01	1.95E-05	2.22E-04
ILMN_1661650	SMEK2	-4.50E-01	1.23E-06	2.11E-05
ILMN_1798712	USP4	-4.50E-01	1.23E-05	1.50E-04
ILMN_1664243	USE1	-4.50E-01	1.80E-06	2.91E-05
ILMN_1774661	SNRPB	-4.50E-01	3.10E-05	3.30E-04
ILMN_2364828	OGT	-4.50E-01	4.61E-06	6.57E-05
ILMN_1676091	LOC388275	-4.50E-01	1.03E-07	2.74E-06
ILMN_2389810	ATP11C	-4.50E-01	2.32E-05	2.58E-04
ILMN_1700625	ATP5S	-4.51E-01	5.75E-07	1.12E-05

ILMN_1658351	FIS1	-4.51E-01	3.52E-03	1.92E-02
ILMN_1679044	LOC644584	-4.51E-01	2.50E-05	2.74E-04
ILMN_1803398	SRF	-4.51E-01	1.78E-04	1.47E-03
ILMN_2050255	UCKL1	-4.51E-01	5.40E-05	5.28E-04
ILMN_3239217	LOC729057	-4.51E-01	2.98E-06	4.52E-05
ILMN_1660602	C1orf43	-4.51E-01	2.08E-07	4.86E-06
ILMN_1766505	COMMD10	-4.51E-01	3.41E-06	5.09E-05
ILMN_1851376		-4.51E-01	2.64E-03	1.50E-02
ILMN_2373495	H2AFY	-4.52E-01	4.05E-08	1.27E-06
ILMN_1778536	BTLA	-4.52E-01	5.78E-03	2.93E-02
ILMN_1775744	MRPS16	-4.52E-01	6.50E-07	1.24E-05
ILMN_1734312	GCN1L1	-4.52E-01	3.03E-04	2.36E-03
ILMN_2143148	TM2D1	-4.52E-01	3.13E-05	3.33E-04
ILMN_2102580	UTP20	-4.53E-01	5.90E-08	1.71E-06
ILMN_2231020	TMEM185B	-4.53E-01	1.32E-05	1.59E-04
ILMN_2061452	ORC2L	-4.53E-01	1.19E-05	1.47E-04
ILMN_1690464	TMEM20	-4.53E-01	4.18E-09	2.08E-07
ILMN_1688152	IL27RA	-4.53E-01	2.35E-03	1.36E-02
ILMN_1763884	Magmas	-4.53E-01	2.19E-04	1.76E-03
ILMN_1717745	TIAL1	-4.53E-01	3.04E-06	4.60E-05
ILMN_1657836	PLEKHG2	-4.54E-01	1.77E-05	2.04E-04
ILMN_3251251	GNL3L	-4.54E-01	9.63E-09	4.02E-07
ILMN_2345512	PPP4R1	-4.54E-01	7.63E-06	1.00E-04
ILMN_3237419	LOC727980	-4.54E-01	3.31E-06	4.95E-05
ILMN_1755911	ING5	-4.54E-01	1.93E-08	7.04E-07
ILMN_1773369	MRPL48	-4.54E-01	1.37E-03	8.52E-03
ILMN_1654357	ZNF24	-4.55E-01	7.45E-07	1.40E-05
ILMN_2346460	NARG2	-4.55E-01	5.64E-08	1.65E-06
ILMN_1804174	FCGR2B	-4.55E-01	8.65E-07	1.58E-05
ILMN_1657884	NME2	-4.55E-01	3.08E-06	4.65E-05
ILMN_1732810	SNX17	-4.56E-01	8.17E-06	1.06E-04
ILMN_1687857	ST6GALNAC4	-4.56E-01	9.96E-04	6.52E-03
ILMN_1744665	EP300	-4.56E-01	1.79E-04	1.48E-03
ILMN_1698406	ORMDL1	-4.56E-01	1.10E-06	1.94E-05
ILMN_1739587	UTY	-4.56E-01	2.66E-04	2.10E-03
ILMN_1696556	KIAA0133	-4.57E-01	3.92E-05	4.02E-04
ILMN_1739847	EIF3D	-4.57E-01	1.32E-04	1.14E-03
ILMN_1695316	SLC39A8	-4.57E-01	4.97E-03	2.58E-02
ILMN_1677747	TMPO	-4.57E-01	6.29E-07	1.21E-05
ILMN_1760858	RAB8A	-4.57E-01	5.13E-06	7.18E-05
ILMN_1814573	FTSJD1	-4.58E-01	1.27E-08	5.01E-07
ILMN_1869087		-4.58E-01	5.87E-06	8.04E-05
ILMN_1658160	FAM156A	-4.58E-01	2.95E-05	3.16E-04
ILMN_2383349	STEAP3	-4.58E-01	1.78E-04	1.48E-03
ILMN_2210837	CDC26	-4.58E-01	7.99E-08	2.20E-06
ILMN_1787762	HEATR1	-4.58E-01	7.87E-11	1.06E-08

ILMN_2409793	MAZ	-4.58E-01	2.76E-05	2.99E-04
ILMN_1743783	CCDC43	-4.58E-01	1.24E-06	2.13E-05
ILMN_1786759	C11orf10	-4.59E-01	1.50E-05	1.78E-04
ILMN_1656316	ZMYM3	-4.59E-01	1.48E-07	3.67E-06
ILMN_3207233	LOC646791	-4.59E-01	1.04E-07	2.75E-06
ILMN_1727315	DENND1A	-4.59E-01	5.75E-08	1.67E-06
ILMN_2373982	PICK1	-4.59E-01	9.72E-07	1.75E-05
ILMN_1705594	NAT10	-4.59E-01	1.28E-06	2.19E-05
ILMN_2349444	NUDT2	-4.59E-01	1.98E-03	1.17E-02
ILMN_1776195	TMSB4Y	-4.60E-01	9.63E-04	6.34E-03
ILMN_1678032	NR1H2	-4.60E-01	1.59E-06	2.63E-05
ILMN_2205350	C6orf66	-4.60E-01	6.73E-05	6.39E-04
ILMN_1776464	PARP4	-4.61E-01	2.17E-04	1.76E-03
ILMN_1894500		-4.61E-01	2.72E-03	1.54E-02
ILMN_1789909	TBC1D9B	-4.61E-01	3.80E-08	1.21E-06
ILMN_1770848	SFXN4	-4.61E-01	9.21E-06	1.17E-04
ILMN_3240155	RNU105A	-4.61E-01	4.06E-04	3.02E-03
ILMN_1776173	PSMD7	-4.61E-01	8.05E-05	7.44E-04
ILMN_3195203	C17orf101	-4.61E-01	2.19E-04	1.77E-03
ILMN_3178258	FABP5L2	-4.61E-01	4.31E-03	2.29E-02
ILMN_1676002	QRICH1	-4.62E-01	2.57E-07	5.75E-06
ILMN_3282506	LOC728098	-4.62E-01	2.17E-09	1.25E-07
ILMN_1670218	EXOSC6	-4.62E-01	1.92E-05	2.19E-04
ILMN_1700461	AARSD1	-4.62E-01	4.33E-08	1.34E-06
ILMN_1658416	MRPS18C	-4.62E-01	1.07E-05	1.33E-04
ILMN_1678966	SNRPF	-4.62E-01	3.38E-06	5.05E-05
ILMN_1686626	BAT1	-4.62E-01	2.33E-07	5.33E-06
ILMN_1708077	DAXX	-4.62E-01	1.26E-05	1.52E-04
ILMN_1795419	C2orf24	-4.62E-01	1.61E-05	1.89E-04
ILMN_1731001	ERICH1	-4.63E-01	1.99E-08	7.21E-07
ILMN_1788095	SPRYD3	-4.63E-01	1.73E-05	2.01E-04
ILMN_1815733	EIF5	-4.63E-01	3.28E-04	2.52E-03
ILMN_1736234	C1orf77	-4.63E-01	1.03E-05	1.28E-04
ILMN_2263144	MGC3196	-4.63E-01	3.16E-05	3.35E-04
ILMN_1729748	PEF1	-4.63E-01	3.58E-07	7.56E-06
ILMN_1675844	WDR1	-4.63E-01	6.69E-07	1.28E-05
ILMN_1693290	METT10D	-4.63E-01	5.19E-07	1.04E-05
ILMN_1785198	POLE3	-4.63E-01	1.33E-08	5.21E-07
ILMN_3232573	MSL3	-4.64E-01	2.59E-03	1.48E-02
ILMN_1796642	NCF2	-4.64E-01	1.39E-03	8.63E-03
ILMN_2235137	FANCD2	-4.64E-01	1.82E-06	2.95E-05
ILMN_3215381	LOC645175	-4.64E-01	2.48E-09	1.38E-07
ILMN_1693664	POMGNT1	-4.64E-01	6.92E-04	4.78E-03
ILMN_1676528	BTN3A2	-4.64E-01	3.59E-08	1.15E-06
ILMN_2103720	MRPL15	-4.64E-01	6.60E-05	6.28E-04
ILMN_2404454	NLE1	-4.64E-01	8.43E-05	7.74E-04

ILMN_1809433	XBP1	-4.65E-01	1.43E-04	1.22E-03
ILMN_2389151	UGP2	-4.65E-01	8.18E-04	5.51E-03
ILMN_1761010	PCCB	-4.65E-01	2.47E-08	8.49E-07
ILMN_1798728	C19orf2	-4.65E-01	2.49E-07	5.61E-06
ILMN_1705733	KIAA0460	-4.65E-01	7.71E-09	3.36E-07
ILMN_1741175	RAB11FIP2	-4.66E-01	6.84E-08	1.91E-06
ILMN_1710844	PARP10	-4.66E-01	1.26E-07	3.22E-06
ILMN_1716400	FOXM1	-4.67E-01	1.07E-07	2.82E-06
ILMN_1734194	EXOSC3	-4.67E-01	4.37E-08	1.34E-06
ILMN_3289090	LOC728059	-4.67E-01	1.91E-04	1.57E-03
ILMN_1697639	OGT	-4.67E-01	3.83E-08	1.21E-06
ILMN_2372136	P4HTM	-4.67E-01	2.70E-08	9.10E-07
ILMN_1782331	TDG	-4.68E-01	1.25E-04	1.08E-03
ILMN_3246315	LOC100133697	-4.68E-01	8.12E-05	7.50E-04
ILMN_1758778	CEP110	-4.68E-01	4.33E-06	6.23E-05
ILMN_2094905	COMMD10	-4.68E-01	6.68E-04	4.64E-03
ILMN_1657796	STMN1	-4.68E-01	6.77E-03	3.35E-02
ILMN_1664010	ELF1	-4.68E-01	3.06E-05	3.26E-04
ILMN_1719611	CCT6A	-4.69E-01	1.77E-04	1.47E-03
ILMN_1756982	CLIC1	-4.69E-01	1.29E-06	2.21E-05
ILMN_1715809	OGFRL1	-4.70E-01	2.01E-08	7.27E-07
ILMN_1811592	ARHGAP21	-4.70E-01	7.72E-06	1.01E-04
ILMN_2215640	TUBA3D	-4.70E-01	2.00E-03	1.19E-02
ILMN_2194828	C16orf53	-4.70E-01	5.34E-03	2.74E-02
ILMN_1660577	ATP5G2	-4.70E-01	8.31E-06	1.08E-04
ILMN_1737005	C19orf61	-4.70E-01	1.68E-06	2.75E-05
ILMN_2152502	MGC72080	-4.71E-01	2.55E-05	2.79E-04
ILMN_2195703	PPARGC1B	-4.71E-01	2.35E-04	1.88E-03
ILMN_1727840	SLC35B1	-4.71E-01	8.06E-09	3.48E-07
ILMN_1747162	DDX47	-4.71E-01	4.79E-08	1.44E-06
ILMN_1860288		-4.71E-01	1.49E-06	2.48E-05
ILMN_2347541	NIN	-4.71E-01	6.93E-03	3.41E-02
ILMN_2093720	THG1L	-4.72E-01	6.41E-06	8.64E-05
ILMN_2394561	IRF2BP2	-4.72E-01	6.43E-05	6.14E-04
ILMN_1747412	DPP3	-4.72E-01	5.75E-06	7.90E-05
ILMN_1780153	QTRT1	-4.72E-01	1.61E-07	3.92E-06
ILMN_1795507	ABCA6	-4.72E-01	4.99E-03	2.59E-02
ILMN_1696190	STRN4	-4.72E-01	3.25E-09	1.71E-07
ILMN_1842582		-4.72E-01	6.55E-03	3.25E-02
ILMN_1750052	NOP14	-4.73E-01	2.44E-04	1.95E-03
ILMN_2390227	TBC1D9B	-4.73E-01	3.85E-08	1.22E-06
ILMN_1661673	SNHG4	-4.73E-01	1.82E-08	6.70E-07
ILMN_1790650	C16orf63	-4.73E-01	8.10E-04	5.46E-03
ILMN_1781623	TEX264	-4.73E-01	2.48E-08	8.51E-07
ILMN_2400030	PTPN2	-4.73E-01	9.86E-06	1.24E-04
ILMN_1678052	C19orf24	-4.74E-01	6.96E-07	1.31E-05

ILMN_1663113	TTLL12	-4.74E-01	6.44E-03	3.21E-02
ILMN_3226904	NOP2	-4.74E-01	5.66E-06	7.79E-05
ILMN_2353240	USF1	-4.75E-01	1.04E-04	9.24E-04
ILMN_1785644	C20orf29	-4.75E-01	5.66E-05	5.50E-04
ILMN_1703695	C19orf12	-4.75E-01	5.43E-06	7.52E-05
ILMN_1682038	SNORA25	-4.75E-01	1.69E-06	2.77E-05
ILMN_1723871	OTUB1	-4.75E-01	1.85E-07	4.40E-06
ILMN_1750864	NIN	-4.75E-01	8.67E-05	7.94E-04
ILMN_1768127	EBNA1BP2	-4.75E-01	3.47E-05	3.63E-04
ILMN_2054392	PPIL1	-4.75E-01	9.59E-08	2.57E-06
ILMN_1659725	EXOSC5	-4.75E-01	9.91E-06	1.25E-04
ILMN_1762888	FAM119A	-4.76E-01	1.76E-08	6.53E-07
ILMN_2396996	PPCS	-4.76E-01	8.09E-04	5.45E-03
ILMN_1703132	LYRM2	-4.76E-01	1.08E-04	9.53E-04
ILMN_1803376	AEBP2	-4.77E-01	6.85E-07	1.30E-05
ILMN_1797933	MRPL17	-4.77E-01	1.43E-06	2.40E-05
ILMN_1751028	SERPINH1	-4.77E-01	4.73E-06	6.70E-05
ILMN_1651237	CDT1	-4.77E-01	3.11E-07	6.76E-06
ILMN_1741780	DUSP28	-4.77E-01	1.11E-07	2.88E-06
ILMN_1786347	TNPO1	-4.77E-01	2.34E-05	2.59E-04
ILMN_1759883	SRP9	-4.77E-01	2.65E-07	5.90E-06
ILMN_1687840	ABCB7	-4.78E-01	3.44E-05	3.60E-04
ILMN_3236463	LOC285296	-4.78E-01	1.04E-04	9.26E-04
ILMN_1721138	GRPEL2	-4.78E-01	3.21E-07	6.95E-06
ILMN_1752914	CDGAP	-4.78E-01	3.26E-09	1.71E-07
ILMN_1660368	TRRAP	-4.78E-01	6.74E-07	1.28E-05
ILMN_1789138	PLEKHA2	-4.78E-01	1.10E-07	2.86E-06
ILMN_1778611	GBAS	-4.78E-01	2.86E-05	3.07E-04
ILMN_2144116	CPSF2	-4.78E-01	3.93E-07	8.19E-06
ILMN_1723768	NLRX1	-4.79E-01	2.60E-09	1.44E-07
ILMN_3245973	MSL1	-4.79E-01	1.73E-06	2.82E-05
ILMN_1865013		-4.79E-01	1.64E-05	1.92E-04
ILMN_1683059	SIRT5	-4.79E-01	3.19E-05	3.37E-04
ILMN_1659800	BCL11A	-4.79E-01	4.01E-03	2.15E-02
ILMN_1845037		-4.79E-01	1.90E-03	1.13E-02
ILMN_1781942	HMMR	-4.79E-01	1.85E-05	2.13E-04
ILMN_3224758	LOC92755	-4.79E-01	7.99E-07	1.48E-05
ILMN_2345015	PTGES2	-4.79E-01	2.85E-05	3.07E-04
ILMN_1751561	CAMK1D	-4.79E-01	9.85E-06	1.24E-04
ILMN_1660663	DYRK1A	-4.79E-01	1.39E-07	3.48E-06
ILMN_2355225	LSP1	-4.80E-01	7.11E-05	6.70E-04
ILMN_1718207	SETDB1	-4.80E-01	3.37E-07	7.22E-06
ILMN_2404085	CLIP1	-4.80E-01	1.27E-04	1.10E-03
ILMN_1785405	SLC17A9	-4.80E-01	5.20E-04	3.74E-03
ILMN_1736729	OAS2	-4.81E-01	2.24E-08	7.86E-07
ILMN_1782069	TRAK1	-4.81E-01	4.82E-03	2.51E-02

ILMN_1692707	C2orf79	-4.81E-01	4.44E-09	2.19E-07
ILMN_1797534	RIOK1	-4.81E-01	2.47E-09	1.38E-07
ILMN_1741976	SMARCAD1	-4.81E-01	1.59E-04	1.34E-03
ILMN_3238707	SNORA8	-4.81E-01	9.85E-04	6.46E-03
ILMN_1766000	PM20D2	-4.81E-01	5.14E-06	7.19E-05
ILMN_1803742	CAPZA1	-4.82E-01	1.92E-03	1.14E-02
ILMN_1810608	PNPT1	-4.82E-01	8.54E-10	6.11E-08
ILMN_1719316	TMED3	-4.82E-01	1.31E-06	2.23E-05
ILMN_1726990	DOM3Z	-4.82E-01	5.86E-07	1.14E-05
ILMN_1674421	TM9SF4	-4.82E-01	1.91E-06	3.07E-05
ILMN_1690473	NFKBIB	-4.82E-01	5.34E-05	5.22E-04
ILMN_1713732	ABL1	-4.83E-01	5.78E-07	1.13E-05
ILMN_1703324	PDSS1	-4.83E-01	4.21E-04	3.12E-03
ILMN_1710177	LOC644670	-4.83E-01	4.85E-07	9.74E-06
ILMN_1671005	IRF2BP2	-4.83E-01	4.52E-09	2.21E-07
ILMN_1709814	NMRAL1	-4.83E-01	6.71E-07	1.28E-05
ILMN_2059294	RTCD1	-4.83E-01	4.01E-03	2.15E-02
ILMN_3237209	LOC642661	-4.84E-01	1.66E-06	2.73E-05
ILMN_3306388	LOC728312	-4.84E-01	2.21E-06	3.48E-05
ILMN_2369682	HNRPA2B1	-4.84E-01	7.87E-05	7.30E-04
ILMN_1673252	AIMP2	-4.85E-01	2.03E-03	1.20E-02
ILMN_3258321	LOC100130932	-4.85E-01	4.97E-06	7.00E-05
ILMN_1712357	HNRPK	-4.85E-01	3.27E-05	3.45E-04
ILMN_1705469	RDH14	-4.85E-01	1.51E-07	3.73E-06
ILMN_1804737	RAVER2	-4.86E-01	1.22E-09	8.01E-08
ILMN_1769250	ARFGAP2	-4.86E-01	6.34E-07	1.22E-05
ILMN_1712888	HSPH1	-4.86E-01	2.53E-06	3.92E-05
ILMN_1739497	GTF2H5	-4.86E-01	2.48E-08	8.51E-07
ILMN_1763228	MEF2D	-4.86E-01	5.40E-04	3.86E-03
ILMN_3213568	LOC402112	-4.86E-01	3.62E-04	2.74E-03
ILMN_1714148	C9orf90	-4.87E-01	1.25E-06	2.14E-05
ILMN_1693685	LOC205251	-4.87E-01	2.21E-07	5.11E-06
ILMN_1777096	TDG	-4.87E-01	1.52E-04	1.29E-03
ILMN_1811955	PRMT5	-4.87E-01	4.07E-06	5.91E-05
ILMN_1760628	C9orf86	-4.87E-01	1.49E-08	5.70E-07
ILMN_2324561	SLC7A6	-4.87E-01	8.99E-09	3.79E-07
ILMN_2345016	PTGES2	-4.87E-01	8.39E-07	1.54E-05
ILMN_1700159	NIPSNAP3A	-4.87E-01	2.86E-09	1.53E-07
ILMN_2207393	CNOT3	-4.88E-01	1.31E-06	2.22E-05
ILMN_1709937	KCNN4	-4.88E-01	5.00E-08	1.50E-06
ILMN_1804530	LOC653888	-4.88E-01	7.07E-04	4.86E-03
ILMN_1691717	RHBDF2	-4.88E-01	7.10E-03	3.48E-02
ILMN_1737205	MCM4	-4.88E-01	2.94E-04	2.29E-03
ILMN_2122103	ETS1	-4.88E-01	5.65E-06	7.79E-05
ILMN_1655990	CDK5RAP2	-4.88E-01	4.91E-08	1.47E-06
ILMN_1703743	AATF	-4.88E-01	6.74E-09	3.01E-07

ILMN_1654542	C5orf21	-4.89E-01	1.25E-08	4.97E-07
ILMN_1687825	CD226	-4.89E-01	1.84E-03	1.10E-02
ILMN_1772163	PRKY	-4.89E-01	4.31E-08	1.33E-06
ILMN_2125562	MOBK1B	-4.89E-01	1.64E-05	1.92E-04
ILMN_1696330	GUF1	-4.89E-01	6.93E-04	4.78E-03
ILMN_2320850	UBE2D3	-4.89E-01	1.13E-06	1.97E-05
ILMN_1664863	CTRL	-4.89E-01	9.17E-10	6.46E-08
ILMN_3188124	LOC100130511	-4.90E-01	5.25E-03	2.70E-02
ILMN_1728540	FUNDC1	-4.90E-01	2.04E-06	3.24E-05
ILMN_2154836	BTG3	-4.90E-01	5.52E-08	1.62E-06
ILMN_2206716	JTB	-4.90E-01	7.91E-07	1.47E-05
ILMN_3274914	LOC648927	-4.90E-01	5.11E-07	1.02E-05
ILMN_2395974	PRDX3	-4.91E-01	3.24E-04	2.50E-03
ILMN_1733603	NDUFC1	-4.91E-01	5.92E-06	8.10E-05
ILMN_1792173	TUBGCP4	-4.91E-01	4.05E-05	4.13E-04
ILMN_1726306	HMBS	-4.92E-01	6.17E-06	8.38E-05
ILMN_1714515	MRPS12	-4.92E-01	1.52E-04	1.29E-03
ILMN_1678957	WDR55	-4.92E-01	1.22E-07	3.13E-06
ILMN_2265654	UBE2C	-4.92E-01	1.89E-07	4.47E-06
ILMN_1716093	KRT10	-4.93E-01	7.21E-05	6.78E-04
ILMN_1750722	RPS7	-4.93E-01	2.25E-03	1.31E-02
ILMN_3195815	LOC100130561	-4.93E-01	5.00E-05	4.94E-04
ILMN_1775901	PHF5A	-4.93E-01	1.22E-05	1.50E-04
ILMN_3246274	RRN3P2	-4.93E-01	2.97E-10	2.78E-08
ILMN_2095820	UTP14A	-4.93E-01	3.36E-04	2.57E-03
ILMN_1780773	LOC400027	-4.94E-01	1.20E-05	1.47E-04
ILMN_1661695	IRAK3	-4.94E-01	1.35E-03	8.41E-03
ILMN_2044832	NOP56	-4.94E-01	2.29E-03	1.33E-02
ILMN_1664920	C19orf12	-4.94E-01	8.09E-08	2.22E-06
ILMN_1733396	CDC25A	-4.95E-01	5.10E-06	7.14E-05
ILMN_2098947	LOC338799	-4.95E-01	3.04E-07	6.63E-06
ILMN_1775224	NOS3	-4.95E-01	3.84E-09	1.94E-07
ILMN_1800261	TUBA1B	-4.96E-01	7.87E-04	5.32E-03
ILMN_1666019	ADNP	-4.96E-01	2.40E-08	8.29E-07
ILMN_2182198	ICT1	-4.96E-01	6.96E-07	1.31E-05
ILMN_1726104	C11orf51	-4.96E-01	6.96E-08	1.94E-06
ILMN_2354211	RCC1	-4.97E-01	3.40E-04	2.59E-03
ILMN_3294033	LOC339970	-4.98E-01	2.12E-04	1.72E-03
ILMN_1764851	TP53RK	-4.98E-01	1.46E-06	2.44E-05
ILMN_3251691	POLR3G	-4.98E-01	3.08E-08	1.01E-06
ILMN_2189605	FAM122B	-4.98E-01	5.03E-11	7.79E-09
ILMN_1671404	SVIL	-4.98E-01	1.09E-03	7.03E-03
ILMN_1744316	TATDN3	-4.98E-01	4.92E-04	3.56E-03
ILMN_3200830	LOC649553	-4.99E-01	1.18E-04	1.04E-03
ILMN_1689624	MINA	-4.99E-01	1.15E-09	7.66E-08
ILMN_1812559	SLC7A6	-4.99E-01	6.51E-09	2.95E-07

ILMN_2142935	BLNK	-4.99E-01	9.78E-07	1.75E-05
ILMN_1777976	SLC25A26	-4.99E-01	6.82E-07	1.30E-05
ILMN_1766408	CBFB	-4.99E-01	1.02E-05	1.27E-04
ILMN_1729319	USP7	-4.99E-01	2.60E-06	4.01E-05
ILMN_1747205	JDP2	-4.99E-01	4.06E-03	2.17E-02
ILMN_1695110	BCAT2	-4.99E-01	3.97E-07	8.26E-06
ILMN_1768958	RASGRP1	-4.99E-01	4.88E-03	2.54E-02
ILMN_1746171	H2AFY	-4.99E-01	1.62E-05	1.90E-04
ILMN_1662768	NHEDC2	-5.00E-01	8.41E-03	4.02E-02
ILMN_2358041	NBN	-5.00E-01	6.10E-06	8.31E-05
ILMN_1717524	LIPT1	-5.00E-01	7.96E-06	1.04E-04
ILMN_1787628	NOP56	-5.00E-01	5.13E-03	2.65E-02
ILMN_1733094	STEAP1	-5.00E-01	1.21E-05	1.48E-04
ILMN_1651378	AUP1	-5.00E-01	5.96E-05	5.75E-04
ILMN_2392717	LARP7	-5.00E-01	6.99E-07	1.32E-05
ILMN_1745049	UQCR	-5.00E-01	1.23E-05	1.50E-04
ILMN_1771734	LOC728564	-5.01E-01	1.31E-06	2.23E-05
ILMN_1731742	TNFRSF13C	-5.01E-01	1.18E-04	1.03E-03
ILMN_3248882	KIAA0114	-5.01E-01	9.09E-05	8.26E-04
ILMN_1809478	SSBP1	-5.01E-01	3.14E-06	4.73E-05
ILMN_2395969	PRDX3	-5.01E-01	2.29E-03	1.33E-02
ILMN_1673944	MANBAL	-5.01E-01	2.73E-09	1.49E-07
ILMN_3236694	LOC100132139	-5.02E-01	5.72E-03	2.90E-02
ILMN_1779399	SNRPA	-5.02E-01	1.01E-07	2.70E-06
ILMN_1721411	PARP10	-5.03E-01	2.45E-09	1.37E-07
ILMN_1692295	MYO1G	-5.03E-01	5.23E-04	3.76E-03
ILMN_2062754	SRA1	-5.03E-01	2.48E-10	2.44E-08
ILMN_1786189	MKI67IP	-5.03E-01	1.08E-10	1.35E-08
ILMN_2095759	OGFRL1	-5.03E-01	8.10E-06	1.06E-04
ILMN_1723212	SFRS3	-5.03E-01	3.92E-04	2.93E-03
ILMN_1742167	TUBA1C	-5.03E-01	4.60E-04	3.36E-03
ILMN_1746664	WSB2	-5.04E-01	2.32E-05	2.58E-04
ILMN_2324672	USF2	-5.04E-01	1.98E-07	4.65E-06
ILMN_1697736	EXOSC2	-5.04E-01	6.83E-08	1.91E-06
ILMN_1733667	DHX35	-5.04E-01	2.80E-08	9.38E-07
ILMN_1657837	ZC3H8	-5.04E-01	2.35E-04	1.88E-03
ILMN_1695797	NCOA6	-5.04E-01	1.11E-06	1.94E-05
ILMN_1763688	C17orf49	-5.05E-01	1.26E-04	1.10E-03
ILMN_1662383	ZRANB2	-5.05E-01	2.97E-08	9.84E-07
ILMN_1791306	C9orf103	-5.05E-01	4.16E-05	4.22E-04
ILMN_1655165	RNF138	-5.07E-01	4.36E-08	1.34E-06
ILMN_1680675	MRRF	-5.07E-01	1.62E-09	9.89E-08
ILMN_1798123	ELOVL1	-5.07E-01	1.55E-07	3.81E-06
ILMN_1679071	MTX3	-5.07E-01	4.22E-08	1.31E-06
ILMN_1741556	MTMR1	-5.07E-01	4.45E-09	2.20E-07
ILMN_2104877	CMPK1	-5.08E-01	2.71E-04	2.13E-03

ILMN_1801923	ATF1	-5.08E-01	1.30E-06	2.22E-05
ILMN_1912619		-5.08E-01	5.99E-05	5.77E-04
ILMN_2329834	RBM12	-5.08E-01	1.26E-04	1.09E-03
ILMN_1714623	TOMM22	-5.08E-01	7.03E-09	3.12E-07
ILMN_3220934	NCRNA00152	-5.08E-01	2.46E-03	1.41E-02
ILMN_1740171	DUSP11	-5.09E-01	5.19E-10	4.20E-08
ILMN_1676241	BCOR	-5.10E-01	2.68E-06	4.12E-05
ILMN_2365465	XBP1	-5.10E-01	5.51E-06	7.62E-05
ILMN_1671791	PCK2	-5.10E-01	1.17E-03	7.47E-03
ILMN_2358919	TP53I3	-5.10E-01	2.38E-07	5.41E-06
ILMN_3210741	LOC642956	-5.10E-01	1.26E-03	7.95E-03
ILMN_1716004	NSUN4	-5.10E-01	2.18E-08	7.71E-07
ILMN_2285375	SORD	-5.10E-01	9.35E-06	1.19E-04
ILMN_1732060	ARHGAP1	-5.10E-01	6.03E-07	1.17E-05
ILMN_1756898	COQ9	-5.11E-01	2.30E-08	8.03E-07
ILMN_1662658	PUS1	-5.11E-01	2.52E-05	2.76E-04
ILMN_1703650	TNIP1	-5.11E-01	2.18E-03	1.28E-02
ILMN_1747630	DEK	-5.12E-01	1.63E-04	1.36E-03
ILMN_1736510	FOXN2	-5.12E-01	1.01E-06	1.81E-05
ILMN_1810228	TTF2	-5.12E-01	4.29E-08	1.33E-06
ILMN_1653026	PLAC8	-5.12E-01	6.02E-05	5.79E-04
ILMN_1774513	DDX17	-5.12E-01	1.18E-08	4.75E-07
ILMN_1678252	HDLBP	-5.12E-01	2.83E-08	9.45E-07
ILMN_1782015	FCRLB	-5.12E-01	2.78E-05	3.01E-04
ILMN_1756439	SCRN1	-5.12E-01	4.48E-03	2.36E-02
ILMN_1690145	LOC653930	-5.12E-01	5.11E-09	2.43E-07
ILMN_3236656	LOC286367	-5.13E-01	3.72E-03	2.01E-02
ILMN_3246409	HNRNPH1	-5.13E-01	9.35E-04	6.18E-03
ILMN_1814789	UBAP2L	-5.13E-01	2.66E-07	5.91E-06
ILMN_1783712	LOC400506	-5.13E-01	3.13E-06	4.72E-05
ILMN_2386355	CSNK2A1	-5.14E-01	5.31E-08	1.57E-06
ILMN_1743104	RBM4B	-5.14E-01	8.24E-04	5.54E-03
ILMN_1724493	LYSMD2	-5.14E-01	3.47E-07	7.38E-06
ILMN_1748650	MRPL45	-5.14E-01	6.86E-09	3.06E-07
ILMN_1720322	PTS	-5.14E-01	4.54E-04	3.32E-03
ILMN_1653438	PHF14	-5.14E-01	1.03E-09	7.01E-08
ILMN_1740045	ESR2	-5.14E-01	3.23E-09	1.70E-07
ILMN_1802706	IDH3G	-5.15E-01	3.35E-07	7.20E-06
ILMN_1703573	DNAJC17	-5.15E-01	5.31E-05	5.20E-04
ILMN_1678037	HIRIP3	-5.15E-01	3.19E-06	4.80E-05
ILMN_1808305	RTCD1	-5.15E-01	3.02E-04	2.35E-03
ILMN_2412860	MCM4	-5.15E-01	6.46E-04	4.51E-03
ILMN_1794512	ADPRH	-5.15E-01	2.10E-04	1.71E-03
ILMN_2233099	SSRP1	-5.16E-01	4.64E-06	6.60E-05
ILMN_3221790	LOC729123	-5.16E-01	3.61E-09	1.85E-07
ILMN_3279712	LOC642590	-5.16E-01	2.95E-03	1.65E-02

ILMN_2352590	ZNF33A	-5.17E-01	1.36E-08	5.28E-07
ILMN_1660880	RNH1	-5.17E-01	5.70E-05	5.53E-04
ILMN_2048636	ME2	-5.17E-01	5.11E-09	2.43E-07
ILMN_2370573	XAF1	-5.17E-01	2.00E-04	1.63E-03
ILMN_1679978	WBSCR16	-5.17E-01	4.24E-06	6.11E-05
ILMN_1782922	PDE4B	-5.17E-01	1.02E-03	6.65E-03
ILMN_2316540	MRPL11	-5.18E-01	1.41E-07	3.51E-06
ILMN_1687538	ETS1	-5.18E-01	5.83E-04	4.12E-03
ILMN_1733616	TFEB	-5.18E-01	4.91E-08	1.47E-06
ILMN_1806999	B9D2	-5.18E-01	2.29E-07	5.26E-06
ILMN_1664167	RPF2	-5.18E-01	3.69E-07	7.78E-06
ILMN_3245824	C17orf89	-5.19E-01	6.41E-08	1.82E-06
ILMN_1715416	NUP188	-5.19E-01	1.21E-06	2.09E-05
ILMN_1689342	NUBP1	-5.19E-01	9.20E-07	1.67E-05
ILMN_1660079	RNF44	-5.19E-01	3.70E-05	3.83E-04
ILMN_2370772	EIF4G1	-5.19E-01	7.73E-04	5.24E-03
ILMN_2166506	XRCC6	-5.20E-01	6.15E-06	8.36E-05
ILMN_3276822	LOC645726	-5.20E-01	9.90E-10	6.79E-08
ILMN_1805449	TAPBPL	-5.20E-01	2.12E-09	1.23E-07
ILMN_2352245	RASSF6	-5.20E-01	1.98E-06	3.14E-05
ILMN_3288830	LOC100132918	-5.20E-01	1.24E-06	2.13E-05
ILMN_1766981	UNC50	-5.21E-01	5.42E-04	3.88E-03
ILMN_1732750	CHCHD8	-5.21E-01	5.75E-06	7.90E-05
ILMN_3241798	SNORA41	-5.21E-01	1.39E-04	1.19E-03
ILMN_2194106	TSPAN12	-5.21E-01	8.31E-03	3.98E-02
ILMN_1763663	FLJ20718	-5.21E-01	1.47E-07	3.65E-06
ILMN_1689110	NOB1	-5.21E-01	4.43E-07	8.99E-06
ILMN_1690546	PPP3CC	-5.21E-01	2.19E-03	1.28E-02
ILMN_1809010	PSMC3	-5.21E-01	1.08E-09	7.27E-08
ILMN_1788166	TTK	-5.21E-01	2.48E-06	3.84E-05
ILMN_2070072	RPS7	-5.22E-01	7.83E-04	5.30E-03
ILMN_2302716	ALDH18A1	-5.22E-01	2.24E-08	7.86E-07
ILMN_3213640	LOC100132444	-5.22E-01	3.26E-06	4.89E-05
ILMN_1754145	CAPRIN1	-5.22E-01	9.05E-04	6.01E-03
ILMN_1672350	JAM2	-5.22E-01	1.05E-02	4.84E-02
ILMN_1772692	DICER1	-5.23E-01	1.61E-08	6.05E-07
ILMN_1790971	LOC652324	-5.23E-01	6.45E-09	2.93E-07
ILMN_2331205	CHKB	-5.23E-01	7.29E-07	1.37E-05
ILMN_1742147	UBL4A	-5.23E-01	3.31E-03	1.82E-02
ILMN_1738642	CMPK1	-5.24E-01	3.98E-07	8.27E-06
ILMN_3236935	LOC728908	-5.24E-01	2.71E-04	2.13E-03
ILMN_2135798	NR2C2AP	-5.24E-01	8.39E-03	4.01E-02
ILMN_3300313	P4HTM	-5.24E-01	9.39E-10	6.55E-08
ILMN_1672878	ABR	-5.25E-01	1.87E-03	1.12E-02
ILMN_1750167	PRR3	-5.25E-01	5.64E-05	5.48E-04
ILMN_1756355	NDUFS3	-5.25E-01	6.29E-10	4.90E-08

ILMN_1737025	PLCL2	-5.26E-01	2.76E-03	1.56E-02
ILMN_2370907	RAD51L3	-5.26E-01	3.27E-07	7.05E-06
ILMN_1679382	CCT2	-5.26E-01	4.50E-08	1.37E-06
ILMN_2403555	DHX30	-5.27E-01	3.40E-08	1.09E-06
ILMN_1697118	ARMC6	-5.27E-01	1.62E-06	2.67E-05
ILMN_1714278	C9orf30	-5.27E-01	2.08E-08	7.48E-07
ILMN_1729767	TARBP2	-5.27E-01	3.21E-08	1.05E-06
ILMN_3280735	LOC643507	-5.27E-01	9.40E-07	1.70E-05
ILMN_1711886	ALG3	-5.27E-01	1.23E-04	1.07E-03
ILMN_2413064	ST6GALNAC4	-5.27E-01	3.35E-04	2.57E-03
ILMN_1788531	SIT1	-5.27E-01	3.55E-03	1.94E-02
ILMN_1693227	ZC3H7A	-5.28E-01	9.28E-08	2.49E-06
ILMN_1707175	NSD1	-5.28E-01	2.38E-07	5.41E-06
ILMN_1758750	EARS2	-5.28E-01	8.65E-08	2.34E-06
ILMN_1766247	ZBTB2	-5.28E-01	3.32E-05	3.50E-04
ILMN_1699525	SRI	-5.28E-01	1.48E-07	3.67E-06
ILMN_1784822	PPP1R3F	-5.28E-01	6.83E-08	1.91E-06
ILMN_3238982	PATE3	-5.29E-01	9.80E-06	1.24E-04
ILMN_3244110	FAM156B	-5.29E-01	5.10E-05	5.02E-04
ILMN_1709333	OAS2	-5.29E-01	1.26E-08	5.01E-07
ILMN_3249748	LDHA	-5.29E-01	2.20E-03	1.29E-02
ILMN_1802819	DEPDC1	-5.30E-01	2.11E-06	3.34E-05
ILMN_1810392	ZNHIT2	-5.30E-01	4.79E-08	1.44E-06
ILMN_3250870	USP46	-5.30E-01	2.38E-08	8.26E-07
ILMN_3242462	UHRF1BP1	-5.30E-01	3.44E-06	5.13E-05
ILMN_1726743	MRPS30	-5.30E-01	7.22E-07	1.36E-05
ILMN_2169839	CNBP	-5.31E-01	2.18E-03	1.27E-02
ILMN_1710186	CCL17	-5.31E-01	1.24E-04	1.08E-03
ILMN_1730019	BRD1	-5.31E-01	4.58E-09	2.24E-07
ILMN_1665435	GPER	-5.31E-01	8.79E-06	1.13E-04
ILMN_1754235	SLC35C2	-5.31E-01	9.81E-05	8.80E-04
ILMN_1815264	RHBDD3	-5.32E-01	2.59E-08	8.78E-07
ILMN_2391141	UBE3A	-5.32E-01	1.28E-06	2.18E-05
ILMN_1806757	MYBBP1A	-5.32E-01	4.36E-05	4.39E-04
ILMN_1811258	RELB	-5.32E-01	1.91E-06	3.07E-05
ILMN_1790518	PHF16	-5.33E-01	1.33E-06	2.26E-05
ILMN_1711799	C9orf40	-5.33E-01	1.72E-05	2.00E-04
ILMN_1689046	FLJ20273	-5.33E-01	4.31E-05	4.35E-04
ILMN_1790782	MED16	-5.33E-01	2.48E-09	1.38E-07
ILMN_3245625	RFX7	-5.34E-01	5.69E-09	2.64E-07
ILMN_1715543	ACOT1	-5.34E-01	1.86E-08	6.80E-07
ILMN_1760741	NDUFA9	-5.34E-01	9.44E-10	6.56E-08
ILMN_2274180	MKKS	-5.34E-01	2.36E-07	5.39E-06
ILMN_1810992	CAD	-5.34E-01	4.13E-06	5.99E-05
ILMN_2138435	MRPS27	-5.34E-01	4.50E-06	6.45E-05
ILMN_1772651	CNOT2	-5.35E-01	4.69E-06	6.66E-05

ILMN_1806123	MRPL23	-5.35E-01	1.71E-05	1.99E-04
ILMN_1703015	ZRANB2	-5.35E-01	3.62E-05	3.75E-04
ILMN_3181328	LOC100130179	-5.36E-01	6.35E-04	4.44E-03
ILMN_1656621	CHMP2A	-5.37E-01	3.12E-10	2.87E-08
ILMN_1704238	C14orf126	-5.37E-01	5.37E-04	3.84E-03
ILMN_1726769	CNDP2	-5.37E-01	1.57E-04	1.32E-03
ILMN_1699476	RPE	-5.37E-01	3.38E-07	7.23E-06
ILMN_1798308	AHSA2	-5.37E-01	9.09E-05	8.26E-04
ILMN_3235326	LOC388796	-5.37E-01	5.32E-09	2.51E-07
ILMN_1707336	ARPC4	-5.38E-01	3.38E-04	2.58E-03
ILMN_1792885	CTSC	-5.38E-01	4.35E-04	3.20E-03
ILMN_2190084	VAMP8	-5.38E-01	1.63E-03	9.89E-03
ILMN_1810680	BOLA2	-5.38E-01	3.50E-09	1.81E-07
ILMN_1797828	DDRGK1	-5.38E-01	7.00E-06	9.31E-05
ILMN_2137084	LIN9	-5.38E-01	4.27E-09	2.13E-07
ILMN_2347748	FLJ12949	-5.39E-01	7.87E-09	3.42E-07
ILMN_1693145	BUB3	-5.39E-01	5.44E-08	1.60E-06
ILMN_1654812	UNC93B1	-5.40E-01	9.30E-05	8.42E-04
ILMN_1722900	EIF4A1	-5.40E-01	1.66E-05	1.93E-04
ILMN_1742577	GTPBP4	-5.40E-01	4.11E-11	6.68E-09
ILMN_2267787	HPS1	-5.40E-01	1.00E-05	1.26E-04
ILMN_1734878	CD79A	-5.41E-01	1.03E-04	9.16E-04
ILMN_1651513	SKIV2L2	-5.41E-01	7.93E-09	3.44E-07
ILMN_1785324	MTHFD1	-5.41E-01	7.79E-05	7.24E-04
ILMN_2200636	KIAA1267	-5.41E-01	2.84E-05	3.06E-04
ILMN_2150402	TMEM64	-5.42E-01	4.92E-08	1.48E-06
ILMN_2156172	HK2	-5.42E-01	5.40E-06	7.49E-05
ILMN_2221564	LYAR	-5.42E-01	2.85E-03	1.60E-02
ILMN_1811650	DUS2L	-5.42E-01	4.16E-08	1.30E-06
ILMN_1709611	PSMA1	-5.42E-01	3.83E-06	5.62E-05
ILMN_1719985	FEM1A	-5.42E-01	2.96E-05	3.17E-04
ILMN_2413251	EWSR1	-5.42E-01	1.20E-07	3.10E-06
ILMN_1795247	ARID2	-5.42E-01	9.55E-10	6.60E-08
ILMN_2391512	NAAA	-5.43E-01	6.22E-08	1.78E-06
ILMN_3179620	LOC100129673	-5.43E-01	2.72E-03	1.54E-02
ILMN_3244693	LOC100130557	-5.43E-01	2.39E-11	4.55E-09
ILMN_1656372	PES1	-5.43E-01	1.21E-07	3.11E-06
ILMN_1699496	PHF21A	-5.43E-01	5.03E-07	1.01E-05
ILMN_1814859	DDX47	-5.43E-01	2.29E-07	5.26E-06
ILMN_1759453	UQCRB	-5.43E-01	1.82E-07	4.33E-06
ILMN_1706645	C6orf150	-5.43E-01	4.30E-05	4.34E-04
ILMN_1794594	RASGRP2	-5.43E-01	1.01E-03	6.60E-03
ILMN_2229032	NME6	-5.43E-01	2.20E-07	5.09E-06
ILMN_1805512	WDR13	-5.43E-01	7.22E-10	5.45E-08
ILMN_1779353	PUS7	-5.44E-01	3.61E-09	1.85E-07
ILMN_1674250	NCKAP1L	-5.45E-01	3.89E-05	3.99E-04

ILMN_1655561	ARPC3	-5.45E-01	6.91E-03	3.40E-02
ILMN_1771320	FLJ38717	-5.45E-01	3.27E-05	3.45E-04
ILMN_2338038	AK3L1	-5.45E-01	9.58E-04	6.31E-03
ILMN_1696031	C15orf21	-5.46E-01	7.43E-05	6.95E-04
ILMN_1742166	GRWD1	-5.47E-01	1.74E-08	6.46E-07
ILMN_1682428	C1orf59	-5.47E-01	1.07E-06	1.89E-05
ILMN_2392546	PAICS	-5.48E-01	9.89E-06	1.24E-04
ILMN_1807106	LDHA	-5.48E-01	2.67E-03	1.52E-02
ILMN_1800634	NME4	-5.48E-01	1.20E-04	1.05E-03
ILMN_1713756	GLUD1	-5.49E-01	3.76E-09	1.91E-07
ILMN_3294365	LOC646993	-5.49E-01	1.60E-07	3.90E-06
ILMN_1809417	LRFN4	-5.49E-01	1.51E-04	1.28E-03
ILMN_1751143	C7orf23	-5.50E-01	5.19E-09	2.45E-07
ILMN_2323979	WARS2	-5.50E-01	5.54E-09	2.59E-07
ILMN_1742544	MEF2C	-5.50E-01	1.86E-05	2.14E-04
ILMN_1813573	SETD1B	-5.51E-01	3.78E-10	3.33E-08
ILMN_3234547	LOC100133803	-5.51E-01	3.99E-05	4.07E-04
ILMN_1662905	NME1-NME2	-5.51E-01	5.29E-06	7.36E-05
ILMN_1663575	MGC87042	-5.52E-01	4.91E-05	4.86E-04
ILMN_2321485	PPP1R8	-5.52E-01	2.79E-08	9.34E-07
ILMN_1725528	LOC400657	-5.53E-01	5.89E-07	1.14E-05
ILMN_1669584	ILF3	-5.53E-01	2.11E-05	2.38E-04
ILMN_1746138	RLTPR	-5.54E-01	1.24E-03	7.82E-03
ILMN_2188374	XPOT	-5.54E-01	9.64E-06	1.22E-04
ILMN_2093343	PLAC8	-5.54E-01	1.28E-06	2.18E-05
ILMN_1693004	C20orf117	-5.54E-01	3.30E-03	1.82E-02
ILMN_3226082	LOC728620	-5.55E-01	2.15E-04	1.74E-03
ILMN_1702171	LPCAT1	-5.55E-01	6.04E-07	1.17E-05
ILMN_1810922	PCNT	-5.55E-01	2.12E-07	4.93E-06
ILMN_1784380	DTX3L	-5.55E-01	9.10E-09	3.83E-07
ILMN_1808219	HEATR3	-5.56E-01	8.93E-09	3.78E-07
ILMN_1763162	DPH2	-5.56E-01	1.23E-07	3.14E-06
ILMN_2321578	P2RY10	-5.56E-01	8.42E-03	4.02E-02
ILMN_1668498	CWF19L2	-5.56E-01	4.44E-06	6.38E-05
ILMN_1743677	HNRNPU	-5.56E-01	2.45E-10	2.43E-08
ILMN_2234229	PRMT6	-5.56E-01	1.81E-08	6.69E-07
ILMN_1802089	SYMPK	-5.57E-01	5.77E-07	1.13E-05
ILMN_1656691	FBXO4	-5.58E-01	3.26E-07	7.04E-06
ILMN_2228710	PDCD5	-5.58E-01	1.19E-08	4.75E-07
ILMN_1786125	CCNA2	-5.58E-01	7.30E-06	9.65E-05
ILMN_2414165	PRDM1	-5.58E-01	5.23E-04	3.76E-03
ILMN_2381138	SEH1L	-5.58E-01	2.59E-04	2.05E-03
ILMN_1789123	PLK4	-5.58E-01	4.65E-07	9.39E-06
ILMN_1768470	EIF4G1	-5.59E-01	5.96E-05	5.75E-04
ILMN_2053527	PARP9	-5.59E-01	9.42E-03	4.42E-02
ILMN_1700518	HMGN4	-5.59E-01	2.69E-06	4.13E-05

ILMN_1795218	DHX30	-5.59E-01	3.34E-08	1.08E-06
ILMN_1800033	LOC649214	-5.59E-01	7.49E-05	7.00E-04
ILMN_1709894	CLPX	-5.60E-01	6.47E-08	1.83E-06
ILMN_3238633	SDHAF2	-5.60E-01	5.01E-05	4.95E-04
ILMN_1799604	OCIAD1	-5.61E-01	6.35E-06	8.58E-05
ILMN_1803953	LOC388789	-5.61E-01	6.20E-08	1.78E-06
ILMN_2044085	RQCD1	-5.61E-01	1.11E-07	2.90E-06
ILMN_1693410	BRI3BP	-5.62E-01	1.07E-08	4.37E-07
ILMN_1778032	SURF6	-5.62E-01	1.29E-08	5.06E-07
ILMN_1906437		-5.62E-01	2.67E-10	2.59E-08
ILMN_1778764	BUB3	-5.62E-01	3.33E-05	3.50E-04
ILMN_1753823	IL17D	-5.63E-01	4.83E-03	2.52E-02
ILMN_1763104	TRAF4	-5.63E-01	1.31E-03	8.21E-03
ILMN_2392356	CTPS2	-5.64E-01	5.02E-10	4.11E-08
ILMN_1763007	HIAT1	-5.64E-01	8.69E-10	6.15E-08
ILMN_2146761	FABP5	-5.64E-01	4.53E-05	4.54E-04
ILMN_1658182	MEX3C	-5.64E-01	2.64E-05	2.88E-04
ILMN_1790757	ADSL	-5.65E-01	1.73E-08	6.44E-07
ILMN_1786016	CHD1L	-5.66E-01	1.18E-07	3.05E-06
ILMN_2158705	ACYP2	-5.66E-01	1.48E-03	9.11E-03
ILMN_1782897	CAPRIN1	-5.66E-01	9.20E-04	6.09E-03
ILMN_1793386	MED12	-5.66E-01	4.69E-09	2.28E-07
ILMN_1724544	PPP4R1	-5.66E-01	2.78E-08	9.32E-07
ILMN_2411963	RBM39	-5.67E-01	3.82E-05	3.93E-04
ILMN_1721337	MRPS18B	-5.67E-01	6.50E-07	1.24E-05
ILMN_1771149	MRPL19	-5.67E-01	5.22E-07	1.04E-05
ILMN_3301372	LOC730167	-5.68E-01	2.27E-04	1.83E-03
ILMN_2403889	PRMT5	-5.68E-01	1.41E-07	3.51E-06
ILMN_1693352	MRPL20	-5.69E-01	1.94E-06	3.10E-05
ILMN_1690209	C1orf186	-5.69E-01	1.38E-07	3.46E-06
ILMN_1776487	TADA1L	-5.69E-01	2.26E-08	7.91E-07
ILMN_1779374	AMMECR1	-5.70E-01	1.49E-08	5.69E-07
ILMN_1733256	PSMD8	-5.70E-01	7.17E-05	6.75E-04
ILMN_1695868	PRICKLE4	-5.70E-01	1.75E-06	2.85E-05
ILMN_1741736	DDX28	-5.70E-01	5.37E-10	4.32E-08
ILMN_1829845		-5.71E-01	2.05E-04	1.67E-03
ILMN_2061732	YRDC	-5.71E-01	4.49E-03	2.37E-02
ILMN_2371964	MRPS12	-5.71E-01	2.19E-05	2.45E-04
ILMN_3215212	LOC653375	-5.71E-01	2.12E-05	2.39E-04
ILMN_2051381	ALDH16A1	-5.71E-01	6.96E-08	1.94E-06
ILMN_1753164	IPO8	-5.72E-01	1.18E-03	7.51E-03
ILMN_1776586	RPL26L1	-5.72E-01	1.48E-09	9.28E-08
ILMN_2415439	NAE1	-5.72E-01	1.36E-06	2.30E-05
ILMN_1792489	ARPC2	-5.72E-01	2.71E-08	9.13E-07
ILMN_2358914	SLC35C2	-5.73E-01	2.27E-05	2.53E-04
ILMN_2225318	SMS	-5.73E-01	1.29E-03	8.10E-03

ILMN_2172202	NUDT15	-5.73E-01	1.68E-05	1.96E-04
ILMN_2382403	FCGR2B	-5.73E-01	6.58E-08	1.85E-06
ILMN_2151817	PFN1	-5.74E-01	5.78E-05	5.59E-04
ILMN_1660582	LIG3	-5.74E-01	1.14E-07	2.95E-06
ILMN_2328433	NOP2	-5.74E-01	1.09E-06	1.92E-05
ILMN_2404385	REPIN1	-5.75E-01	3.43E-07	7.32E-06
ILMN_1768751	MTA3	-5.75E-01	6.39E-06	8.62E-05
ILMN_1795118	SIDT1	-5.75E-01	6.23E-03	3.12E-02
ILMN_1705907	NUP153	-5.75E-01	1.86E-05	2.14E-04
ILMN_1673185	CPSF2	-5.76E-01	2.81E-05	3.03E-04
ILMN_1742450	TAPBP	-5.76E-01	1.48E-03	9.11E-03
ILMN_1751963	ZCWPW1	-5.76E-01	5.16E-10	4.19E-08
ILMN_2266948	SLC38A1	-5.76E-01	1.31E-04	1.13E-03
ILMN_1675483	ANKMY1	-5.77E-01	2.45E-05	2.69E-04
ILMN_1728305	PUM2	-5.77E-01	3.91E-11	6.44E-09
ILMN_3242196	LOC100132728	-5.77E-01	1.55E-06	2.57E-05
ILMN_1680692	NUCKS1	-5.77E-01	2.95E-05	3.17E-04
ILMN_1767253	RRP12	-5.79E-01	1.59E-07	3.87E-06
ILMN_3200414	LOC441131	-5.79E-01	3.25E-03	1.80E-02
ILMN_2105966	SLC35A4	-5.79E-01	1.88E-06	3.02E-05
ILMN_1681503	MCM2	-5.79E-01	4.70E-06	6.66E-05
ILMN_1782488	RNASEH2B	-5.79E-01	3.36E-04	2.57E-03
ILMN_1812776	FBXO28	-5.79E-01	1.07E-08	4.38E-07
ILMN_1669142	NARG1	-5.80E-01	2.60E-09	1.44E-07
ILMN_1750401	C17orf62	-5.80E-01	3.53E-05	3.67E-04
ILMN_1769118	SEPT9	-5.81E-01	1.13E-07	2.94E-06
ILMN_1699859	GNPAT	-5.81E-01	3.80E-05	3.92E-04
ILMN_1668605	NAAA	-5.81E-01	1.16E-07	3.00E-06
ILMN_1760441	MRPS5	-5.81E-01	9.97E-08	2.66E-06
ILMN_1690371	MRPL11	-5.81E-01	4.76E-05	4.73E-04
ILMN_1742410	BCL2L1	-5.81E-01	7.34E-06	9.69E-05
ILMN_2205935	SFXN1	-5.82E-01	7.70E-05	7.17E-04
ILMN_1740737	DCPS	-5.82E-01	3.97E-08	1.25E-06
ILMN_1915076		-5.82E-01	9.07E-03	4.28E-02
ILMN_1746465	FJX1	-5.82E-01	2.20E-09	1.26E-07
ILMN_2117987	TFDP1	-5.83E-01	2.43E-05	2.68E-04
ILMN_1662306	RABL3	-5.84E-01	8.55E-09	3.65E-07
ILMN_2092756	TMEM109	-5.85E-01	6.64E-07	1.26E-05
ILMN_1805693	GMIP	-5.85E-01	4.33E-06	6.23E-05
ILMN_1770035	NCOA5	-5.85E-01	1.04E-07	2.75E-06
ILMN_2320853	UBE2D3	-5.85E-01	7.00E-07	1.32E-05
ILMN_1730118	ZNF644	-5.86E-01	1.57E-05	1.85E-04
ILMN_2356311	C21orf51	-5.86E-01	2.43E-07	5.50E-06
ILMN_1671281	RNASEL	-5.86E-01	2.23E-04	1.80E-03
ILMN_1670079	OMA1	-5.86E-01	4.34E-09	2.15E-07
ILMN_1714082	CMAS	-5.87E-01	6.30E-05	6.03E-04

ILMN_1729868	SPRYD4	-5.87E-01	1.77E-07	4.25E-06
ILMN_1664034	ZNF485	-5.87E-01	8.62E-11	1.14E-08
ILMN_1737833	ATN1	-5.87E-01	3.67E-04	2.77E-03
ILMN_1661717	TFDP1	-5.88E-01	3.15E-05	3.35E-04
ILMN_2378048	HNRPK	-5.88E-01	2.74E-07	6.05E-06
ILMN_1678454	CASP4	-5.89E-01	2.27E-09	1.29E-07
ILMN_1683462	GSS	-5.89E-01	3.00E-10	2.79E-08
ILMN_1724990	C3orf75	-5.89E-01	1.61E-08	6.05E-07
ILMN_1672940	ZNF562	-5.89E-01	2.19E-09	1.26E-07
ILMN_1723158	NOP2	-5.89E-01	8.85E-09	3.75E-07
ILMN_1801387	YEATS4	-5.89E-01	4.68E-05	4.67E-04
ILMN_1664303	HTATIP2	-5.89E-01	9.93E-07	1.78E-05
ILMN_1813489	RAF1	-5.89E-01	2.27E-12	9.16E-10
ILMN_1714738	SCMH1	-5.90E-01	2.49E-07	5.61E-06
ILMN_1799814	WDR57	-5.90E-01	4.22E-07	8.66E-06
ILMN_1698213	RBM3	-5.90E-01	6.36E-05	6.08E-04
ILMN_1675186	ME2	-5.90E-01	2.68E-11	4.94E-09
ILMN_2390338	UBE2E3	-5.90E-01	9.70E-05	8.72E-04
ILMN_1813389	MRPS7	-5.91E-01	2.97E-07	6.48E-06
ILMN_3210491	LOC389049	-5.91E-01	3.74E-06	5.51E-05
ILMN_3200921	LOC642590	-5.91E-01	1.17E-05	1.44E-04
ILMN_3248890	SNORA24	-5.92E-01	3.79E-03	2.04E-02
ILMN_1698996	SLC19A1	-5.92E-01	4.86E-05	4.82E-04
ILMN_1793643	MRM1	-5.92E-01	3.91E-10	3.40E-08
ILMN_1684887	SAMSN1	-5.92E-01	1.49E-09	9.33E-08
ILMN_1762275	CSE1L	-5.93E-01	1.00E-04	8.98E-04
ILMN_1813975	ADI1	-5.93E-01	6.43E-08	1.82E-06
ILMN_1665736	LOC648024	-5.93E-01	5.83E-04	4.13E-03
ILMN_1657898	MTP18	-5.93E-01	1.95E-11	3.95E-09
ILMN_1709039	RPL13	-5.94E-01	5.73E-03	2.91E-02
ILMN_1803110	SF3B3	-5.94E-01	1.70E-06	2.78E-05
ILMN_1750711	MYO19	-5.94E-01	5.43E-07	1.07E-05
ILMN_1692026	SUV420H1	-5.94E-01	2.69E-07	5.96E-06
ILMN_1669692	IKZF3	-5.94E-01	1.51E-07	3.72E-06
ILMN_1708619	SEH1L	-5.94E-01	4.44E-07	9.01E-06
ILMN_1668996	C1QBP	-5.95E-01	3.23E-06	4.85E-05
ILMN_1692168	UBE2Z	-5.95E-01	1.21E-08	4.84E-07
ILMN_1751403	NUDT15	-5.95E-01	2.02E-06	3.21E-05
ILMN_1806432	NT5C	-5.95E-01	8.60E-09	3.67E-07
ILMN_1722309	ENDOG	-5.95E-01	4.80E-04	3.49E-03
ILMN_1694504	RNF220	-5.95E-01	4.18E-10	3.57E-08
ILMN_1786893	RBM5	-5.96E-01	1.30E-07	3.30E-06
ILMN_2067370	SNRPF	-5.96E-01	5.13E-09	2.43E-07
ILMN_1784320	ELMO1	-5.96E-01	1.02E-07	2.72E-06
ILMN_1778347	NUDT2	-5.96E-01	6.00E-04	4.23E-03
ILMN_1720850	BAZ2B	-5.96E-01	7.49E-06	9.86E-05

ILMN_1666376	TRIM56	-5.96E-01	7.59E-08	2.10E-06
ILMN_2177965	RPS19BP1	-5.97E-01	7.65E-09	3.34E-07
ILMN_2080611	PDSS1	-5.97E-01	4.37E-04	3.21E-03
ILMN_1653129	CSTF2	-5.97E-01	5.67E-07	1.11E-05
ILMN_2119421	LOC143543	-5.97E-01	9.13E-07	1.66E-05
ILMN_1742569	PAPD1	-5.97E-01	5.33E-08	1.57E-06
ILMN_1753790	ZNF259	-5.97E-01	1.88E-06	3.03E-05
ILMN_1807042	MARCKS	-5.98E-01	9.76E-05	8.77E-04
ILMN_1691731	PARP14	-5.98E-01	4.40E-03	2.33E-02
ILMN_1749243	BANF1	-5.98E-01	1.74E-04	1.44E-03
ILMN_1761450	DHRS4L2	-5.98E-01	1.40E-08	5.40E-07
ILMN_1722390	CHRAC1	-5.99E-01	5.42E-09	2.54E-07
ILMN_1669502	E2F3	-5.99E-01	2.20E-08	7.77E-07
ILMN_1752526	RNF144B	-6.00E-01	8.29E-04	5.57E-03
ILMN_1803312	DIMT1L	-6.00E-01	9.61E-04	6.33E-03
ILMN_1705032	SEH1L	-6.01E-01	5.21E-05	5.12E-04
ILMN_1696463	SPI1	-6.01E-01	2.91E-08	9.66E-07
ILMN_1773797	LOC652615	-6.01E-01	2.71E-08	9.11E-07
ILMN_2162328	PTS	-6.01E-01	2.39E-05	2.64E-04
ILMN_1772359	LAPTM5	-6.01E-01	8.14E-05	7.51E-04
ILMN_1667716	TMEM101	-6.02E-01	6.28E-09	2.88E-07
ILMN_1672097	CD86	-6.02E-01	1.22E-04	1.06E-03
ILMN_1688515	ZNF195	-6.02E-01	6.19E-08	1.77E-06
ILMN_1679134	NSMCE4A	-6.03E-01	1.21E-07	3.11E-06
ILMN_1768181	TOR3A	-6.03E-01	5.65E-08	1.65E-06
ILMN_1803997	SDCCAG3	-6.04E-01	9.48E-05	8.57E-04
ILMN_1754839	DHX15	-6.04E-01	3.89E-08	1.22E-06
ILMN_2409318	RCCD1	-6.04E-01	5.20E-11	7.90E-09
ILMN_1813581	CNR1	-6.05E-01	3.30E-07	7.11E-06
ILMN_1749752	NOLA1	-6.05E-01	4.23E-08	1.31E-06
ILMN_2105441	IGJ	-6.05E-01	7.53E-08	2.08E-06
ILMN_1702175	ST7	-6.05E-01	4.74E-08	1.44E-06
ILMN_1718815	SLTM	-6.05E-01	5.23E-11	7.91E-09
ILMN_1732410	SLC16A9	-6.06E-01	1.73E-04	1.44E-03
ILMN_1653861	SCMH1	-6.06E-01	3.93E-05	4.02E-04
ILMN_1784540	KBTBD2	-6.06E-01	2.51E-08	8.57E-07
ILMN_2066124	AFG3L2	-6.07E-01	3.79E-05	3.90E-04
ILMN_2112811	RPL36A	-6.07E-01	1.43E-05	1.70E-04
ILMN_2380588	C6orf108	-6.08E-01	4.79E-05	4.76E-04
ILMN_1726153	MGC4677	-6.08E-01	1.07E-02	4.91E-02
ILMN_2408851	ARHGAP30	-6.08E-01	8.64E-07	1.58E-05
ILMN_2311548	PTRH2	-6.09E-01	1.05E-07	2.76E-06
ILMN_1787919	PARVB	-6.09E-01	4.60E-03	2.41E-02
ILMN_3226012	LOC728698	-6.10E-01	3.01E-04	2.34E-03
ILMN_1677292	C5orf30	-6.10E-01	3.43E-10	3.08E-08
ILMN_1776094	PPCS	-6.10E-01	5.64E-06	7.77E-05

ILMN_1776777	ADAR	-6.10E-01	9.44E-04	6.23E-03
ILMN_1676893	ADCY3	-6.10E-01	7.26E-03	3.55E-02
ILMN_3204117	LOC728115	-6.10E-01	1.19E-06	2.07E-05
ILMN_1715968	MLL4	-6.11E-01	3.13E-07	6.78E-06
ILMN_1655868	ANP32C	-6.11E-01	6.35E-07	1.22E-05
ILMN_2351309	TIAL1	-6.12E-01	1.75E-07	4.20E-06
ILMN_1762883	ECE2	-6.12E-01	3.31E-05	3.49E-04
ILMN_1767142	ZNF280D	-6.12E-01	5.91E-06	8.09E-05
ILMN_1701331	UBE2M	-6.12E-01	1.07E-07	2.81E-06
ILMN_1773763	MTA2	-6.13E-01	2.47E-06	3.83E-05
ILMN_1761147	GABPB2	-6.13E-01	3.43E-03	1.88E-02
ILMN_1693293	LOC648176	-6.13E-01	3.49E-08	1.12E-06
ILMN_1709772	SNX5	-6.14E-01	4.45E-07	9.01E-06
ILMN_1729509	C1orf43	-6.14E-01	1.29E-08	5.09E-07
ILMN_2343105	LIPT1	-6.14E-01	3.76E-08	1.19E-06
ILMN_1905548		-6.15E-01	7.61E-10	5.68E-08
ILMN_3302139	LOC729687	-6.15E-01	5.03E-07	1.01E-05
ILMN_1651828	CCT3	-6.15E-01	2.45E-05	2.70E-04
ILMN_2400219	SRI	-6.15E-01	5.06E-09	2.41E-07
ILMN_2284591	OPA3	-6.15E-01	1.17E-09	7.75E-08
ILMN_1708101	LMNB2	-6.15E-01	4.61E-06	6.57E-05
ILMN_3198367	LOC646347	-6.16E-01	1.73E-04	1.44E-03
ILMN_1694479	WDR18	-6.17E-01	4.55E-06	6.51E-05
ILMN_1709750	SUSD1	-6.17E-01	1.89E-11	3.90E-09
ILMN_2133638	DULLARD	-6.17E-01	8.70E-10	6.15E-08
ILMN_2043728	ZNF341	-6.17E-01	4.74E-10	3.92E-08
ILMN_2108938	FNBP4	-6.18E-01	1.08E-07	2.84E-06
ILMN_1719471	MSH3	-6.18E-01	1.74E-09	1.05E-07
ILMN_1806651	PARP8	-6.19E-01	1.08E-05	1.34E-04
ILMN_1793672	SIX5	-6.20E-01	6.59E-03	3.27E-02
ILMN_1780036	WDR1	-6.20E-01	4.38E-09	2.17E-07
ILMN_1790819	LOC728556	-6.20E-01	1.68E-05	1.96E-04
ILMN_1804248	FDPS	-6.21E-01	3.40E-07	7.27E-06
ILMN_1853876		-6.21E-01	1.16E-05	1.43E-04
ILMN_1792435	STAG1	-6.22E-01	3.04E-11	5.35E-09
ILMN_1765684	C19orf70	-6.22E-01	2.27E-09	1.29E-07
ILMN_1689665	NAE1	-6.23E-01	6.67E-11	9.45E-09
ILMN_2156982	IMP4	-6.23E-01	8.00E-10	5.85E-08
ILMN_2234873	NME2	-6.23E-01	2.08E-05	2.35E-04
ILMN_1746276	EPC1	-6.24E-01	4.40E-11	7.00E-09
ILMN_2319077	FAS	-6.24E-01	2.85E-03	1.60E-02
ILMN_1778691	TIA1	-6.24E-01	3.70E-06	5.47E-05
ILMN_1675577	TRMT61A	-6.25E-01	6.66E-11	9.45E-09
ILMN_1784737	S1PR4	-6.25E-01	9.77E-03	4.56E-02
ILMN_2371590	DDX17	-6.26E-01	5.03E-05	4.96E-04
ILMN_3281502	LOC653375	-6.27E-01	1.02E-07	2.71E-06

ILMN_1778836	SFRS7	-6.27E-01	9.06E-07	1.65E-05
ILMN_2154603	CCDC16	-6.27E-01	1.12E-08	4.54E-07
ILMN_1810275	SLC7A7	-6.28E-01	4.15E-10	3.57E-08
ILMN_1709044	TGIF2	-6.29E-01	8.66E-05	7.92E-04
ILMN_1764609	PWWP2B	-6.29E-01	4.30E-05	4.34E-04
ILMN_3226392	LOC729608	-6.29E-01	2.04E-06	3.23E-05
ILMN_2168449	DHX15	-6.30E-01	7.60E-09	3.33E-07
ILMN_3297455	LOC729082	-6.30E-01	8.27E-10	5.99E-08
ILMN_3235928	CYTIP	-6.30E-01	5.05E-08	1.51E-06
ILMN_1689336	HOXA10	-6.31E-01	4.23E-05	4.28E-04
ILMN_3217285	LOC389322	-6.31E-01	1.64E-06	2.69E-05
ILMN_2320964	ADAR	-6.31E-01	6.19E-04	4.34E-03
ILMN_2382290	KREMEN2	-6.32E-01	1.39E-04	1.19E-03
ILMN_1725071	CCDC12	-6.32E-01	1.05E-08	4.30E-07
ILMN_1730809	SLC29A2	-6.32E-01	9.47E-10	6.56E-08
ILMN_1811373	FAM20B	-6.32E-01	2.84E-08	9.49E-07
ILMN_3297577	LOC729841	-6.33E-01	1.43E-03	8.86E-03
ILMN_1680134	CARM1	-6.33E-01	7.18E-09	3.18E-07
ILMN_1776325	UBE2Q1	-6.33E-01	2.51E-08	8.57E-07
ILMN_2180848	COCH	-6.33E-01	4.07E-05	4.15E-04
ILMN_1799103	SNRPB	-6.34E-01	2.47E-05	2.71E-04
ILMN_1810486	RAB34	-6.34E-01	2.34E-07	5.35E-06
ILMN_1741133	NME1	-6.34E-01	3.03E-06	4.59E-05
ILMN_1701243	C10orf2	-6.34E-01	5.47E-07	1.08E-05
ILMN_3251511	SFRS2IP	-6.34E-01	1.02E-08	4.23E-07
ILMN_1683204	GMEB2	-6.34E-01	1.65E-05	1.93E-04
ILMN_2176955	CUL5	-6.35E-01	2.10E-07	4.89E-06
ILMN_1757956	PCGF1	-6.36E-01	2.88E-07	6.31E-06
ILMN_1651872	UBIAD1	-6.36E-01	1.45E-08	5.57E-07
ILMN_1682792	BYSL	-6.36E-01	3.60E-11	6.11E-09
ILMN_1722662	RAD23B	-6.36E-01	5.39E-09	2.54E-07
ILMN_1683175	C9orf23	-6.36E-01	3.30E-03	1.82E-02
ILMN_1673215	PCBP1	-6.36E-01	6.99E-07	1.32E-05
ILMN_1732967	KIAA1949	-6.37E-01	1.92E-07	4.53E-06
ILMN_2392043	SPI1	-6.37E-01	6.68E-08	1.88E-06
ILMN_1722894	ZNRD1	-6.37E-01	1.59E-08	6.01E-07
ILMN_1664833	MRPL50	-6.37E-01	3.78E-11	6.28E-09
ILMN_2334989	CCT3	-6.37E-01	8.24E-05	7.59E-04
ILMN_1691809	PSMA1	-6.38E-01	6.57E-08	1.85E-06
ILMN_2182335	ARID2	-6.38E-01	1.64E-10	1.80E-08
ILMN_1739397	GLMN	-6.39E-01	1.26E-07	3.21E-06
ILMN_2200503	NIT2	-6.39E-01	8.12E-08	2.22E-06
ILMN_1761981	FAM96A	-6.39E-01	1.96E-05	2.23E-04
ILMN_1668411	FHL2	-6.39E-01	9.45E-03	4.44E-02
ILMN_3211935	LOC100132715	-6.39E-01	8.11E-07	1.50E-05
ILMN_1804834	C6orf130	-6.39E-01	1.62E-08	6.07E-07

ILMN_2194649	TADA1L	-6.40E-01	1.68E-06	2.75E-05
ILMN_1764549	UBE3A	-6.40E-01	2.00E-08	7.25E-07
ILMN_3206827	LOC100131737	-6.41E-01	5.51E-08	1.62E-06
ILMN_1683817	UBE2Q2	-6.41E-01	1.11E-06	1.94E-05
ILMN_1685661	RRP15	-6.41E-01	3.44E-08	1.11E-06
ILMN_2211800	HMGB1L1	-6.41E-01	2.99E-03	1.67E-02
ILMN_1719158	CTBP1	-6.41E-01	1.29E-05	1.56E-04
ILMN_2168564	KLHL14	-6.41E-01	1.11E-04	9.77E-04
ILMN_1796099	LOC644380	-6.42E-01	3.82E-04	2.87E-03
ILMN_1797684	PDCD2	-6.42E-01	2.46E-11	4.62E-09
ILMN_2310909	ATP2A3	-6.42E-01	8.33E-09	3.58E-07
ILMN_1774196	URM1	-6.43E-01	1.46E-09	9.19E-08
ILMN_2360415	PRNP	-6.43E-01	2.24E-04	1.80E-03
ILMN_1658883	ARAF	-6.43E-01	4.24E-08	1.31E-06
ILMN_3226505	MSL3	-6.43E-01	5.74E-03	2.91E-02
ILMN_1752927	KIAA1600	-6.43E-01	1.13E-07	2.92E-06
ILMN_1803775	HSPE1	-6.44E-01	2.04E-04	1.66E-03
ILMN_2092693	LSM12	-6.44E-01	1.72E-07	4.16E-06
ILMN_1706149	PDCD2L	-6.44E-01	1.86E-07	4.42E-06
ILMN_1800451	MED16	-6.44E-01	2.59E-09	1.43E-07
ILMN_2223010	VBP1	-6.45E-01	5.05E-08	1.51E-06
ILMN_1664602	LOC143543	-6.45E-01	1.51E-07	3.72E-06
ILMN_1735908	UTP15	-6.45E-01	1.41E-07	3.51E-06
ILMN_2402168	EXOSC10	-6.46E-01	6.90E-07	1.31E-05
ILMN_1711414	MRPS27	-6.46E-01	1.22E-04	1.07E-03
ILMN_1695271	RPP25	-6.46E-01	2.96E-04	2.31E-03
ILMN_1694466	ZBED1	-6.46E-01	5.29E-06	7.36E-05
ILMN_1694327	LOC285176	-6.49E-01	6.72E-06	9.00E-05
ILMN_1683933	SERPINA9	-6.49E-01	2.32E-04	1.86E-03
ILMN_1672717	C10orf57	-6.49E-01	3.79E-10	3.34E-08
ILMN_1678362	INO80	-6.49E-01	2.15E-08	7.63E-07
ILMN_1719975	HOXC4	-6.49E-01	1.37E-07	3.44E-06
ILMN_1762666	DHRS4	-6.49E-01	1.62E-08	6.08E-07
ILMN_1853824	MGAT3	-6.50E-01	1.27E-08	5.01E-07
ILMN_3248966	MMADHC	-6.50E-01	1.21E-08	4.83E-07
ILMN_1734602	SRRM2	-6.50E-01	3.23E-07	6.98E-06
ILMN_1738263	PIGU	-6.51E-01	8.06E-08	2.21E-06
ILMN_1693766	CEP135	-6.51E-01	2.28E-09	1.29E-07
ILMN_3268165	LOC100128353	-6.51E-01	1.80E-06	2.92E-05
ILMN_1727402	HCLS1	-6.52E-01	7.97E-10	5.83E-08
ILMN_1675669	IBTK	-6.53E-01	1.05E-06	1.87E-05
ILMN_1749838	MZF1	-6.54E-01	3.16E-10	2.90E-08
ILMN_1763641	ZNF614	-6.55E-01	1.14E-09	7.60E-08
ILMN_1699887	ST14	-6.55E-01	4.83E-05	4.79E-04
ILMN_1664177	ATXN7L2	-6.56E-01	3.82E-04	2.87E-03
ILMN_1661595	C1orf53	-6.56E-01	5.42E-09	2.54E-07

ILMN_1843198		-6.56E-01	3.85E-05	3.96E-04
ILMN_3181420	HMGXB4	-6.57E-01	2.78E-09	1.51E-07
ILMN_3207605	LOC399804	-6.57E-01	1.16E-10	1.40E-08
ILMN_1739345	C11orf48	-6.58E-01	2.89E-07	6.33E-06
ILMN_1678799	RAPGEF1	-6.58E-01	1.76E-03	1.06E-02
ILMN_3284447	LOC647150	-6.59E-01	9.52E-07	1.71E-05
ILMN_1694305	SMS	-6.59E-01	1.16E-04	1.02E-03
ILMN_1663002	STOML2	-6.59E-01	2.21E-07	5.10E-06
ILMN_3298829	LOC729505	-6.59E-01	4.19E-06	6.05E-05
ILMN_2053829	CBLN3	-6.59E-01	8.65E-04	5.78E-03
ILMN_2275248	ECE2	-6.60E-01	3.12E-04	2.41E-03
ILMN_1667222	MTX1	-6.60E-01	1.69E-08	6.31E-07
ILMN_1654639	HERC6	-6.60E-01	1.96E-07	4.62E-06
ILMN_1685112	TACO1	-6.60E-01	8.46E-09	3.63E-07
ILMN_1661197	CLCF1	-6.60E-01	2.57E-03	1.47E-02
ILMN_1731891	UBXN8	-6.60E-01	1.47E-08	5.64E-07
ILMN_1789240	MLST8	-6.60E-01	1.24E-05	1.51E-04
ILMN_1807455	DHRS7	-6.61E-01	3.82E-04	2.87E-03
ILMN_1665738	FLI1	-6.61E-01	7.49E-09	3.30E-07
ILMN_1735930	KLF2	-6.61E-01	3.38E-03	1.85E-02
ILMN_2323491	NUP62	-6.61E-01	3.12E-10	2.87E-08
ILMN_2064898	CCDC56	-6.61E-01	3.48E-07	7.39E-06
ILMN_1818149		-6.61E-01	7.25E-05	6.81E-04
ILMN_2311537	HMGA1	-6.62E-01	1.89E-04	1.56E-03
ILMN_1799367	TXNDC14	-6.62E-01	4.19E-07	8.62E-06
ILMN_1696601	VARS	-6.62E-01	1.03E-07	2.72E-06
ILMN_1660749	ASPSCR1	-6.62E-01	5.99E-04	4.22E-03
ILMN_1796235	CIRH1A	-6.62E-01	1.20E-08	4.79E-07
ILMN_1696870	TGFBRAP1	-6.63E-01	7.28E-11	1.01E-08
ILMN_3247835	CXorf64	-6.63E-01	1.96E-05	2.23E-04
ILMN_3249406	URB2	-6.64E-01	5.33E-07	1.06E-05
ILMN_1728512	YWHAH	-6.64E-01	7.16E-04	4.91E-03
ILMN_2055477	EXOSC7	-6.64E-01	3.19E-10	2.91E-08
ILMN_1678165	LSM7	-6.65E-01	1.83E-08	6.72E-07
ILMN_1726064	PAK1IP1	-6.66E-01	6.81E-08	1.91E-06
ILMN_1679405	DDX56	-6.67E-01	4.87E-08	1.47E-06
ILMN_1757317	LARS	-6.67E-01	2.51E-06	3.89E-05
ILMN_1696127	KIAA0240	-6.68E-01	3.99E-09	2.01E-07
ILMN_1774334	HIGD2A	-6.68E-01	2.28E-08	7.98E-07
ILMN_2351298	WIPF1	-6.68E-01	4.15E-04	3.09E-03
ILMN_2369018	EVI2A	-6.68E-01	1.24E-06	2.13E-05
ILMN_1748916	C18orf55	-6.68E-01	8.59E-11	1.14E-08
ILMN_1669497	OSBPL10	-6.68E-01	1.50E-03	9.22E-03
ILMN_1805271	ZNF721	-6.70E-01	1.64E-07	3.99E-06
ILMN_3248343	INO80	-6.70E-01	4.02E-07	8.34E-06
ILMN_2351548	FAIM	-6.70E-01	7.09E-08	1.97E-06

ILMN_1700168	LARS2	-6.70E-01	5.61E-11	8.26E-09
ILMN_1795922	CCDC16	-6.70E-01	1.01E-08	4.18E-07
ILMN_1764362	LYAR	-6.71E-01	1.22E-03	7.72E-03
ILMN_1814230	MTCP1	-6.71E-01	1.69E-08	6.30E-07
ILMN_1795341	SFRS1	-6.71E-01	1.02E-06	1.81E-05
ILMN_3244348	SNORA18	-6.71E-01	1.77E-04	1.47E-03
ILMN_1672662	SLC20A1	-6.72E-01	3.01E-08	9.92E-07
ILMN_2414399	NME1	-6.72E-01	3.94E-06	5.75E-05
ILMN_3299558	SFRS18	-6.72E-01	1.23E-10	1.45E-08
ILMN_1696485	HNRNPAB	-6.73E-01	2.01E-03	1.19E-02
ILMN_1659524	C6orf66	-6.73E-01	1.31E-06	2.22E-05
ILMN_1707503	C1orf144	-6.73E-01	5.54E-10	4.41E-08
ILMN_1761479	ZC3HC1	-6.73E-01	2.77E-11	5.01E-09
ILMN_1814657	TFAP4	-6.74E-01	2.13E-05	2.39E-04
ILMN_3196019	FAM60A	-6.74E-01	7.48E-07	1.40E-05
ILMN_3251404	NUCKS1	-6.75E-01	2.97E-07	6.49E-06
ILMN_1743635	ALG14	-6.75E-01	1.53E-07	3.78E-06
ILMN_1686401	LOC728739	-6.76E-01	7.99E-09	3.46E-07
ILMN_1680390	GCNT2	-6.76E-01	5.77E-03	2.92E-02
ILMN_2331163	CUL4A	-6.77E-01	1.12E-09	7.48E-08
ILMN_1672496	DNAJA1	-6.77E-01	5.61E-04	3.99E-03
ILMN_1804812	ANAPC1	-6.77E-01	1.67E-10	1.81E-08
ILMN_1676745	ZNF142	-6.77E-01	1.63E-07	3.96E-06
ILMN_2373266	SFRS12	-6.77E-01	1.57E-08	5.96E-07
ILMN_3241756	FAM136B	-6.78E-01	1.19E-10	1.43E-08
ILMN_1710923	SLAMF7	-6.79E-01	1.82E-08	6.71E-07
ILMN_1730294	INO80C	-6.79E-01	9.33E-10	6.53E-08
ILMN_1772702	SFRS2B	-6.79E-01	1.96E-07	4.62E-06
ILMN_2233878	SERF1B	-6.80E-01	2.47E-08	8.48E-07
ILMN_3179371	HNRNPK	-6.80E-01	1.70E-06	2.78E-05
ILMN_1751368	HNRNPD	-6.80E-01	2.12E-08	7.53E-07
ILMN_1671494	USP5	-6.80E-01	2.35E-08	8.15E-07
ILMN_1723962	LXN	-6.80E-01	5.63E-05	5.48E-04
ILMN_2082130	C1orf123	-6.81E-01	3.15E-08	1.03E-06
ILMN_3251137	FAM119A	-6.81E-01	1.48E-06	2.47E-05
ILMN_2387285	MSL3	-6.82E-01	2.29E-03	1.33E-02
ILMN_1803939	YIPF6	-6.82E-01	2.86E-08	9.53E-07
ILMN_1745497	C12orf26	-6.82E-01	1.78E-07	4.26E-06
ILMN_1810488	NFYC	-6.83E-01	4.42E-08	1.35E-06
ILMN_1657893	TXNRD2	-6.83E-01	1.07E-08	4.37E-07
ILMN_2125374	CMAS	-6.83E-01	1.64E-05	1.92E-04
ILMN_1710209	MFSD6	-6.84E-01	6.47E-04	4.51E-03
ILMN_1734742	ARHGDIA	-6.84E-01	1.30E-03	8.15E-03
ILMN_1746257	DAZAP1	-6.84E-01	6.74E-09	3.01E-07
ILMN_1707339	BTG3	-6.85E-01	7.49E-08	2.07E-06
ILMN_2164164	AICDA	-6.86E-01	4.93E-04	3.57E-03

ILMN_1701749	UQCRFS1	-6.87E-01	1.59E-08	6.01E-07
ILMN_1717366	MDFIC	-6.87E-01	3.96E-04	2.96E-03
ILMN_2347234	PRMT1	-6.87E-01	1.73E-05	2.01E-04
ILMN_2112402	PHF5A	-6.87E-01	1.19E-06	2.07E-05
ILMN_1706502	EIF2AK2	-6.87E-01	2.57E-03	1.47E-02
ILMN_3211857	LOC648822	-6.88E-01	6.84E-06	9.12E-05
ILMN_1749006	RCSD1	-6.88E-01	4.94E-05	4.89E-04
ILMN_1704571	FAM53B	-6.88E-01	5.36E-07	1.06E-05
ILMN_1680738	C5orf13	-6.88E-01	8.68E-10	6.15E-08
ILMN_1742935	ZNF33B	-6.88E-01	8.44E-10	6.06E-08
ILMN_1723729	RSL1D1	-6.88E-01	3.32E-07	7.13E-06
ILMN_1705908	RPL7L1	-6.89E-01	1.33E-05	1.60E-04
ILMN_1767219	POLR1C	-6.89E-01	5.20E-08	1.54E-06
ILMN_2088612	XPO4	-6.89E-01	5.30E-07	1.05E-05
ILMN_1684594	USP24	-6.89E-01	5.55E-05	5.41E-04
ILMN_1707763	ST7	-6.90E-01	2.20E-08	7.77E-07
ILMN_2374076	C14orf102	-6.90E-01	9.10E-07	1.65E-05
ILMN_2344971	FOXM1	-6.90E-01	1.59E-08	6.01E-07
ILMN_1701374	NUP35	-6.90E-01	5.16E-10	4.19E-08
ILMN_1662843	CD53	-6.90E-01	1.37E-06	2.31E-05
ILMN_1703430	FLJ10374	-6.90E-01	5.15E-08	1.53E-06
ILMN_1812795	RUNX1T1	-6.90E-01	1.75E-10	1.86E-08
ILMN_1702806	PDCL3	-6.91E-01	1.99E-05	2.26E-04
ILMN_1786105	PCBD1	-6.92E-01	1.38E-08	5.36E-07
ILMN_1679929	KLF13	-6.92E-01	3.15E-04	2.44E-03
ILMN_1750805	ARHGAP30	-6.92E-01	3.52E-07	7.46E-06
ILMN_1660938	TOE1	-6.92E-01	5.64E-12	1.60E-09
ILMN_1787511	THUMPD2	-6.93E-01	1.71E-08	6.37E-07
ILMN_2382657	ARHGAP9	-6.93E-01	3.99E-09	2.01E-07
ILMN_2151368	NOL12	-6.93E-01	2.82E-08	9.43E-07
ILMN_2401769	PHF14	-6.93E-01	3.13E-08	1.02E-06
ILMN_2388272	MED24	-6.94E-01	2.64E-10	2.57E-08
ILMN_1727043	GLT25D1	-6.94E-01	1.70E-05	1.98E-04
ILMN_1803676	ENOSF1	-6.95E-01	1.85E-06	3.00E-05
ILMN_2163306	FAM120A	-6.95E-01	9.62E-11	1.24E-08
ILMN_1765520	MTIF2	-6.96E-01	7.65E-09	3.34E-07
ILMN_1697268	EMILIN2	-6.96E-01	1.72E-06	2.80E-05
ILMN_1670901	COX10	-6.96E-01	2.03E-08	7.32E-07
ILMN_1798172	IPO4	-6.97E-01	3.73E-06	5.51E-05
ILMN_1690386	CSRP2BP	-6.97E-01	1.01E-08	4.17E-07
ILMN_1728845	SMARCD1	-6.97E-01	1.92E-11	3.91E-09
ILMN_1797693	BRI3BP	-6.97E-01	6.27E-08	1.79E-06
ILMN_1698463	ILF3	-6.97E-01	4.65E-09	2.26E-07
ILMN_1784467	NUP210	-6.97E-01	5.00E-08	1.50E-06
ILMN_1712634	TIA1	-6.97E-01	2.01E-08	7.28E-07
ILMN_2219712	HMGB2	-6.98E-01	4.45E-06	6.39E-05

ILMN_1809583	CREBBP	-6.99E-01	1.26E-05	1.53E-04
ILMN_1812580	YDJC	-7.00E-01	2.92E-08	9.68E-07
ILMN_1708672	ACAT2	-7.00E-01	1.11E-07	2.90E-06
ILMN_1763605	DIDO1	-7.00E-01	9.17E-09	3.85E-07
ILMN_1711189	EXOSC10	-7.00E-01	2.04E-10	2.08E-08
ILMN_1730260	N6AMT2	-7.00E-01	6.43E-09	2.92E-07
ILMN_2398039	TCERG1	-7.01E-01	3.52E-09	1.83E-07
ILMN_1805658	LTV1	-7.01E-01	2.18E-09	1.25E-07
ILMN_1740395	RAVER1	-7.02E-01	8.02E-09	3.47E-07
ILMN_1778796	ADSS	-7.02E-01	2.38E-06	3.71E-05
ILMN_1698803	ZAK	-7.02E-01	1.34E-08	5.22E-07
ILMN_2375319	RASGRP2	-7.03E-01	1.24E-03	7.87E-03
ILMN_1740861	DTWD1	-7.03E-01	4.58E-10	3.82E-08
ILMN_3220718	LOC729086	-7.03E-01	4.62E-08	1.40E-06
ILMN_2072541	RAB11FIP2	-7.03E-01	1.90E-06	3.05E-05
ILMN_1682567	CCDC106	-7.03E-01	1.04E-03	6.78E-03
ILMN_1703263	SP140	-7.04E-01	5.19E-08	1.54E-06
ILMN_2067709	TFB2M	-7.04E-01	4.31E-07	8.78E-06
ILMN_3244065	C9orf69	-7.04E-01	6.84E-05	6.49E-04
ILMN_1788416	FAM108C1	-7.04E-01	8.81E-07	1.61E-05
ILMN_1711450	TH1L	-7.04E-01	2.07E-09	1.21E-07
ILMN_1789944	PGAM5	-7.04E-01	6.60E-09	2.98E-07
ILMN_1752046	SH2B3	-7.05E-01	8.60E-05	7.87E-04
ILMN_2316918	PANK1	-7.05E-01	2.68E-08	9.04E-07
ILMN_3177285	HNRNPR	-7.05E-01	1.24E-06	2.13E-05
ILMN_1745271	EXOSC4	-7.05E-01	1.85E-08	6.80E-07
ILMN_1741200	RFX5	-7.05E-01	7.23E-08	2.01E-06
ILMN_3278995	LOC643167	-7.05E-01	1.98E-04	1.62E-03
ILMN_1654268	HMGB2	-7.05E-01	9.83E-08	2.62E-06
ILMN_1737988	PRNP	-7.07E-01	1.08E-07	2.83E-06
ILMN_1666409	PSMB6	-7.07E-01	1.57E-11	3.43E-09
ILMN_1800619	BRI3BP	-7.07E-01	5.29E-09	2.50E-07
ILMN_2321451	HNRNPD	-7.08E-01	1.64E-12	7.68E-10
ILMN_1680703	MRPS15	-7.08E-01	5.20E-11	7.90E-09
ILMN_1837935	TNPO1	-7.08E-01	1.51E-10	1.69E-08
ILMN_2069593	SFRS2IP	-7.08E-01	3.32E-07	7.13E-06
ILMN_2053567	FASTKD2	-7.09E-01	4.62E-12	1.42E-09
ILMN_1653180	TPM4	-7.10E-01	1.93E-05	2.20E-04
ILMN_1735093	TIMELESS	-7.11E-01	2.64E-08	8.93E-07
ILMN_1782551	E2F5	-7.11E-01	1.02E-07	2.70E-06
ILMN_2210601	RNASEL	-7.11E-01	6.96E-05	6.59E-04
ILMN_2099528	BTLA	-7.11E-01	8.72E-04	5.82E-03
ILMN_1784655	TLCD1	-7.11E-01	3.87E-04	2.90E-03
ILMN_1670638	PITPNC1	-7.11E-01	5.90E-03	2.98E-02
ILMN_1779584	UTP18	-7.12E-01	5.26E-10	4.25E-08
ILMN_1777811	URG4	-7.12E-01	6.67E-09	3.00E-07

ILMN_1775761	TSR1	-7.12E-01	1.92E-05	2.19E-04
ILMN_2378257	SDF4	-7.12E-01	6.82E-08	1.91E-06
ILMN_1660063	POLE4	-7.13E-01	2.42E-11	4.59E-09
ILMN_1800750	ZNF692	-7.14E-01	8.30E-08	2.26E-06
ILMN_2326512	CASP1	-7.14E-01	1.87E-06	3.02E-05
ILMN_1695945	MEIS2	-7.14E-01	1.46E-08	5.59E-07
ILMN_1721605	SMYD2	-7.15E-01	1.08E-10	1.35E-08
ILMN_2090558	C2orf25	-7.15E-01	2.70E-08	9.08E-07
ILMN_2333865	DNAJB12	-7.15E-01	1.02E-08	4.22E-07
ILMN_1760256	RBM22	-7.16E-01	5.60E-13	3.86E-10
ILMN_1772946	STRN3	-7.16E-01	3.33E-05	3.50E-04
ILMN_2126706	LMNB1	-7.16E-01	3.15E-04	2.43E-03
ILMN_1652379	SUCLG2	-7.16E-01	3.12E-06	4.71E-05
ILMN_3304898	LOC92755	-7.18E-01	2.69E-06	4.12E-05
ILMN_1700822	DPP3	-7.18E-01	6.52E-07	1.25E-05
ILMN_1786852	ZCCHC3	-7.18E-01	3.64E-08	1.16E-06
ILMN_2055330	KIF26B	-7.19E-01	3.53E-04	2.68E-03
ILMN_1675124	DDX17	-7.19E-01	3.19E-10	2.91E-08
ILMN_1773716	MRPL9	-7.19E-01	2.71E-10	2.61E-08
ILMN_1696065	SDF4	-7.19E-01	1.47E-07	3.65E-06
ILMN_1704702	MCM7	-7.20E-01	9.17E-06	1.17E-04
ILMN_1659273	LOC441408	-7.21E-01	3.66E-10	3.24E-08
ILMN_1697735	EWSR1	-7.22E-01	3.70E-11	6.18E-09
ILMN_2349129	DPP3	-7.22E-01	6.20E-08	1.78E-06
ILMN_2373831	BTN3A3	-7.22E-01	7.37E-06	9.72E-05
ILMN_1814971	TCF25	-7.22E-01	1.08E-10	1.35E-08
ILMN_1713156	MSL3L1	-7.22E-01	4.38E-03	2.31E-02
ILMN_2413808	CD53	-7.23E-01	2.43E-06	3.78E-05
ILMN_1778681	EBF1	-7.24E-01	1.14E-04	1.01E-03
ILMN_1660027	FCGR2B	-7.24E-01	4.79E-09	2.31E-07
ILMN_1800612	VBP1	-7.25E-01	8.01E-08	2.20E-06
ILMN_1662848	TXNDC15	-7.25E-01	9.35E-09	3.92E-07
ILMN_1688971	NOL11	-7.25E-01	2.57E-07	5.75E-06
ILMN_1789349	UBQLN4	-7.25E-01	5.12E-09	2.43E-07
ILMN_1711853	MED24	-7.25E-01	5.60E-10	4.43E-08
ILMN_1856861		-7.27E-01	4.64E-11	7.30E-09
ILMN_1811327	MRPL27	-7.27E-01	2.90E-10	2.73E-08
ILMN_1738529	BCS1L	-7.27E-01	2.48E-11	4.63E-09
ILMN_1769319	CNBP	-7.27E-01	5.74E-05	5.57E-04
ILMN_1729142	CENPV	-7.28E-01	5.22E-03	2.69E-02
ILMN_1659257	TNFRSF8	-7.28E-01	9.85E-03	4.59E-02
ILMN_2109156	RANBP1	-7.29E-01	3.50E-04	2.66E-03
ILMN_1793410	SNTB1	-7.29E-01	2.38E-09	1.34E-07
ILMN_1802553	MRPS24	-7.29E-01	5.44E-09	2.55E-07
ILMN_1776552	FUBP1	-7.29E-01	3.80E-09	1.92E-07
ILMN_1746686	POLR1C	-7.30E-01	7.36E-10	5.55E-08

ILMN_1790461	C6orf125	-7.30E-01	7.78E-07	1.45E-05
ILMN_1753190	C9orf102	-7.30E-01	8.84E-05	8.07E-04
ILMN_1761083	HNRNPA3	-7.30E-01	6.03E-08	1.73E-06
ILMN_2414325	TNFAIP8	-7.31E-01	8.59E-03	4.09E-02
ILMN_3285198	LOC389168	-7.33E-01	7.62E-05	7.11E-04
ILMN_1721457	RANBP1	-7.33E-01	4.20E-04	3.11E-03
ILMN_1737184	CDCA7	-7.33E-01	2.14E-05	2.40E-04
ILMN_1805474	C1orf131	-7.34E-01	7.02E-07	1.32E-05
ILMN_2065606	TOMM40L	-7.34E-01	1.58E-09	9.68E-08
ILMN_1692398	CNTNAP1	-7.35E-01	2.10E-08	7.51E-07
ILMN_1797005	PGLS	-7.36E-01	1.08E-07	2.84E-06
ILMN_1695792	CUL4A	-7.37E-01	4.92E-09	2.36E-07
ILMN_1701114	GBP1	-7.37E-01	2.85E-09	1.53E-07
ILMN_1679880	THOC6	-7.38E-01	1.69E-10	1.83E-08
ILMN_1777982	USF1	-7.39E-01	1.39E-04	1.19E-03
ILMN_1666632	VPS52	-7.39E-01	3.75E-09	1.91E-07
ILMN_2142284	SLC25A43	-7.40E-01	1.44E-04	1.23E-03
ILMN_3212373	LOC727803	-7.40E-01	1.11E-04	9.80E-04
ILMN_2194009	ABCC4	-7.40E-01	2.71E-10	2.61E-08
ILMN_1685678	EEF1B2	-7.41E-01	1.98E-04	1.62E-03
ILMN_2179837	BANF1	-7.41E-01	1.96E-07	4.63E-06
ILMN_1695422	NCL	-7.42E-01	2.62E-03	1.50E-02
ILMN_2390457	ESR2	-7.42E-01	1.63E-11	3.50E-09
ILMN_2180827	MEPCE	-7.42E-01	2.93E-11	5.22E-09
ILMN_1783681	MRPL34	-7.42E-01	3.43E-09	1.79E-07
ILMN_2375599	RNH1	-7.43E-01	9.91E-06	1.25E-04
ILMN_2397024	SPOP	-7.43E-01	8.58E-10	6.12E-08
ILMN_1765332	TIMM10	-7.43E-01	9.07E-06	1.16E-04
ILMN_2047511	CENTA1	-7.44E-01	1.82E-07	4.33E-06
ILMN_1678235	KIAA1267	-7.44E-01	5.19E-08	1.54E-06
ILMN_1741572	AKAP8	-7.44E-01	2.90E-12	1.09E-09
ILMN_1810759	C2orf25	-7.45E-01	4.71E-09	2.28E-07
ILMN_1775542	FAIM3	-7.46E-01	1.13E-03	7.25E-03
ILMN_1678143	ARHGDIB	-7.47E-01	1.13E-10	1.39E-08
ILMN_1653504	EDG1	-7.47E-01	1.43E-09	9.05E-08
ILMN_1716730	FAM44B	-7.47E-01	2.55E-09	1.42E-07
ILMN_3250243	FAM119A	-7.47E-01	1.34E-08	5.23E-07
ILMN_1718558	PARP12	-7.47E-01	5.19E-06	7.24E-05
ILMN_2279635	EIF4G2	-7.48E-01	2.80E-06	4.27E-05
ILMN_2318643	TGIF1	-7.50E-01	2.33E-03	1.35E-02
ILMN_3231638	FAM160B1	-7.50E-01	2.98E-08	9.87E-07
ILMN_2092536	HSPE1	-7.50E-01	1.60E-03	9.76E-03
ILMN_1706094	HECTD1	-7.51E-01	1.97E-05	2.24E-04
ILMN_2137536	ZZZ3	-7.52E-01	6.24E-06	8.46E-05
ILMN_1772719	GPN1	-7.53E-01	4.21E-08	1.31E-06
ILMN_1673962	NUP205	-7.53E-01	1.45E-10	1.64E-08

ILMN_1814173	SMARCA4	-7.53E-01	7.87E-09	3.42E-07
ILMN_1736555	ZNF280D	-7.55E-01	2.86E-09	1.53E-07
ILMN_1712950	PFN1	-7.55E-01	4.13E-05	4.20E-04
ILMN_1688534	EIF2B5	-7.55E-01	5.32E-10	4.29E-08
ILMN_1761456	ALG13	-7.56E-01	1.57E-09	9.68E-08
ILMN_1815169	MCM5	-7.57E-01	2.16E-07	5.01E-06
ILMN_1750051	FLJ39827	-7.58E-01	9.79E-08	2.62E-06
ILMN_3241257	POLR3E	-7.58E-01	1.05E-09	7.11E-08
ILMN_1812926	ANTXR2	-7.58E-01	8.13E-03	3.91E-02
ILMN_1660462	MCOLN2	-7.59E-01	1.99E-03	1.18E-02
ILMN_1782045	FKBP4	-7.59E-01	8.42E-05	7.73E-04
ILMN_1722838	MRPL46	-7.59E-01	1.47E-10	1.66E-08
ILMN_2398587	ZNRD1	-7.60E-01	8.16E-11	1.09E-08
ILMN_1663954	TH1L	-7.60E-01	1.56E-08	5.92E-07
ILMN_2380946	EIF4G2	-7.60E-01	4.77E-06	6.75E-05
ILMN_3224934	SFRS18	-7.60E-01	4.40E-10	3.72E-08
ILMN_1805481	TRMT11	-7.60E-01	3.54E-06	5.26E-05
ILMN_2121437	NCL	-7.61E-01	4.06E-04	3.03E-03
ILMN_1811181	FLJ20444	-7.61E-01	1.39E-07	3.47E-06
ILMN_1795089	RASAL3	-7.62E-01	2.99E-05	3.20E-04
ILMN_1658437	SFXN4	-7.63E-01	4.24E-04	3.14E-03
ILMN_2359800	MS4A6A	-7.63E-01	1.28E-04	1.11E-03
ILMN_1652787	PIK3AP1	-7.63E-01	1.02E-05	1.27E-04
ILMN_1726245	TGFBR2	-7.64E-01	5.30E-03	2.72E-02
ILMN_1805812	TOR1A	-7.64E-01	2.07E-11	4.10E-09
ILMN_1706886	BCL7A	-7.65E-01	1.47E-09	9.25E-08
ILMN_2151056	C10orf32	-7.65E-01	1.91E-05	2.18E-04
ILMN_1727740	SYNCRIP	-7.66E-01	1.20E-06	2.08E-05
ILMN_1686662	C15orf28	-7.66E-01	1.23E-09	8.06E-08
ILMN_1769633	CTSO	-7.66E-01	1.23E-08	4.88E-07
ILMN_2161357	C6orf111	-7.66E-01	4.78E-09	2.31E-07
ILMN_1716816	TMEM87A	-7.66E-01	2.60E-09	1.44E-07
ILMN_3291709	LOC402175	-7.66E-01	1.49E-09	9.33E-08
ILMN_1773117	BCOR	-7.67E-01	5.42E-06	7.51E-05
ILMN_2070815	MPDU1	-7.67E-01	3.44E-09	1.79E-07
ILMN_2045729	WDR12	-7.68E-01	6.21E-08	1.78E-06
ILMN_1747303	DDX39	-7.68E-01	2.36E-08	8.20E-07
ILMN_1708203	OTUD4	-7.69E-01	2.33E-08	8.10E-07
ILMN_1665797	CSE1L	-7.69E-01	2.03E-07	4.77E-06
ILMN_1656066	TNPO2	-7.69E-01	2.65E-09	1.45E-07
ILMN_1692517	LOC653381	-7.69E-01	2.54E-06	3.92E-05
ILMN_1691798	ZNF26	-7.69E-01	5.68E-10	4.48E-08
ILMN_1791388	ZNF787	-7.70E-01	1.02E-10	1.28E-08
ILMN_1703617	AHSA1	-7.70E-01	4.16E-10	3.57E-08
ILMN_1737298	MAT2A	-7.70E-01	1.37E-06	2.31E-05
ILMN_1706238	CSE1L	-7.71E-01	1.35E-06	2.28E-05

ILMN_1802519	VPS36	-7.71E-01	1.01E-09	6.94E-08
ILMN_1810423	RPP40	-7.71E-01	3.59E-06	5.32E-05
ILMN_1662318	CCDC59	-7.71E-01	1.22E-08	4.85E-07
ILMN_1720819	LOC653566	-7.71E-01	3.82E-08	1.21E-06
ILMN_2372040	MTP18	-7.72E-01	1.36E-10	1.57E-08
ILMN_2385173	U2AF2	-7.73E-01	6.42E-12	1.74E-09
ILMN_2356574	GTF3C2	-7.73E-01	1.13E-10	1.39E-08
ILMN_1706275	C8orf33	-7.73E-01	1.48E-08	5.65E-07
ILMN_1739236	ZNF668	-7.74E-01	6.84E-13	4.05E-10
ILMN_1673138	ZBTB33	-7.74E-01	3.84E-08	1.21E-06
ILMN_2182531	C18orf55	-7.74E-01	1.17E-11	2.68E-09
ILMN_1803254	KIAA2010	-7.75E-01	5.66E-08	1.65E-06
ILMN_2139100	SHISA5	-7.75E-01	5.00E-07	1.00E-05
ILMN_1801119	BCL2	-7.75E-01	6.99E-03	3.44E-02
ILMN_2326509	CASP1	-7.75E-01	1.23E-06	2.12E-05
ILMN_2386818	URG4	-7.76E-01	7.40E-11	1.01E-08
ILMN_1756204	RPS6KA4	-7.76E-01	8.38E-10	6.03E-08
ILMN_2246882	SP140	-7.76E-01	6.24E-08	1.78E-06
ILMN_1759154	PABPN1	-7.77E-01	1.16E-08	4.68E-07
ILMN_1753819	RFFL	-7.77E-01	7.92E-07	1.47E-05
ILMN_1731224	PARP9	-7.77E-01	9.10E-03	4.30E-02
ILMN_1741054	SLC5A6	-7.77E-01	4.85E-06	6.85E-05
ILMN_1698491	MBD3	-7.78E-01	1.02E-11	2.43E-09
ILMN_1699695	TNFRSF21	-7.81E-01	2.85E-09	1.53E-07
ILMN_1666670	RBX1	-7.81E-01	2.50E-08	8.55E-07
ILMN_1713875	NME1	-7.81E-01	2.54E-08	8.65E-07
ILMN_2266005	C21orf51	-7.81E-01	1.08E-09	7.27E-08
ILMN_1692486	ZNRD1	-7.82E-01	5.23E-12	1.54E-09
ILMN_1688698	ZEB2	-7.82E-01	9.61E-05	8.66E-04
ILMN_1689001	CDK4	-7.83E-01	1.51E-09	9.39E-08
ILMN_3282768	LOC644879	-7.83E-01	1.50E-07	3.71E-06
ILMN_3306730	RBM47	-7.83E-01	5.78E-06	7.94E-05
ILMN_2067708	TFB2M	-7.84E-01	7.80E-07	1.45E-05
ILMN_2090059	ZFY	-7.84E-01	4.18E-06	6.04E-05
ILMN_1751571	RAD23A	-7.84E-01	7.45E-10	5.59E-08
ILMN_1785336	PMM2	-7.85E-01	1.70E-09	1.03E-07
ILMN_1714730	UBE2C	-7.85E-01	1.80E-09	1.07E-07
ILMN_1721106	C14orf159	-7.86E-01	1.77E-06	2.88E-05
ILMN_1779751	C7orf55	-7.87E-01	8.83E-07	1.61E-05
ILMN_2312606	IRF5	-7.87E-01	8.49E-04	5.68E-03
ILMN_2175894	HNRPR	-7.87E-01	4.70E-07	9.47E-06
ILMN_3256868	LOC100129585	-7.88E-01	2.42E-09	1.36E-07
ILMN_1657632	ZMYM6	-7.89E-01	6.47E-08	1.83E-06
ILMN_1711862	RNF7	-7.89E-01	1.80E-09	1.07E-07
ILMN_2117330	NDUFB2	-7.89E-01	1.24E-06	2.13E-05
ILMN_1664560	DYRK1A	-7.89E-01	7.42E-12	1.95E-09

ILMN_2357272	BCLAF1	-7.90E-01	3.65E-10	3.24E-08
ILMN_3224926	RBM47	-7.90E-01	2.62E-09	1.44E-07
ILMN_1809439	HMGB1L1	-7.91E-01	2.64E-04	2.08E-03
ILMN_1683859	SLC7A1	-7.91E-01	1.53E-04	1.29E-03
ILMN_1704750	LOC647000	-7.92E-01	1.66E-08	6.20E-07
ILMN_2216582	LYL1	-7.92E-01	1.72E-10	1.84E-08
ILMN_3243457	ANKLE1	-7.93E-01	2.77E-06	4.23E-05
ILMN_1815668	GTF2IP1	-7.93E-01	2.00E-04	1.63E-03
ILMN_1663616	DNAJC7	-7.93E-01	4.72E-09	2.28E-07
ILMN_2406892	C19orf2	-7.95E-01	5.88E-08	1.70E-06
ILMN_2300695	IKZF3	-7.95E-01	4.46E-10	3.76E-08
ILMN_1790577	SLC35F2	-7.95E-01	6.80E-09	3.03E-07
ILMN_1692473	PRMT1	-7.96E-01	1.21E-07	3.11E-06
ILMN_2401714	MS4A1	-7.96E-01	2.03E-10	2.07E-08
ILMN_1757415	C1orf163	-7.97E-01	4.22E-11	6.83E-09
ILMN_2408645	LOC653566	-7.97E-01	1.04E-07	2.75E-06
ILMN_1793220	GART	-7.97E-01	2.98E-09	1.58E-07
ILMN_1789171	EEF2K	-7.98E-01	1.34E-05	1.61E-04
ILMN_1651433	DCK	-7.99E-01	1.16E-08	4.69E-07
ILMN_1735548	HIVEP1	-7.99E-01	5.92E-03	2.99E-02
ILMN_3210917	LOC389168	-8.00E-01	7.59E-06	9.97E-05
ILMN_1812940	TRMT1	-8.00E-01	9.26E-11	1.20E-08
ILMN_1798459	PPAN	-8.00E-01	3.07E-08	1.01E-06
ILMN_1733390	LARP1B	-8.03E-01	1.29E-07	3.27E-06
ILMN_1803005	MMACHC	-8.03E-01	4.43E-13	3.22E-10
ILMN_1682404	SETMAR	-8.05E-01	1.88E-11	3.89E-09
ILMN_1769433	IQGAP2	-8.05E-01	1.60E-08	6.02E-07
ILMN_1788457	ABCC4	-8.06E-01	3.60E-11	6.11E-09
ILMN_1704305	NIP7	-8.06E-01	8.80E-07	1.61E-05
ILMN_2151048	STAG1	-8.07E-01	1.64E-10	1.80E-08
ILMN_2363361	SFXN4	-8.07E-01	1.23E-03	7.81E-03
ILMN_2403458	SMARCB1	-8.09E-01	1.34E-06	2.26E-05
ILMN_1787127	SLC43A2	-8.09E-01	4.73E-05	4.71E-04
ILMN_2364535	SNUPN	-8.11E-01	4.38E-11	7.00E-09
ILMN_1700660	RNF135	-8.11E-01	1.27E-08	5.01E-07
ILMN_3234142	LOC728855	-8.11E-01	5.36E-06	7.44E-05
ILMN_2301083	UBE2C	-8.12E-01	7.84E-11	1.06E-08
ILMN_3245236	FBRS	-8.12E-01	8.33E-09	3.58E-07
ILMN_2374293	DYRK1A	-8.14E-01	1.45E-09	9.12E-08
ILMN_3236551	WDFY4	-8.14E-01	1.48E-10	1.66E-08
ILMN_1662799	GPSM3	-8.14E-01	1.37E-06	2.31E-05
ILMN_3307786	L3MBTL2	-8.14E-01	1.02E-06	1.82E-05
ILMN_1715179	SNRPA1	-8.14E-01	1.35E-08	5.26E-07
ILMN_2105983	XRCC5	-8.14E-01	1.70E-07	4.10E-06
ILMN_1653618	ZZZ3	-8.15E-01	1.23E-05	1.50E-04
ILMN_2371700	UCHL5IP	-8.16E-01	2.03E-09	1.19E-07

ILMN_1788810	C12orf30	-8.17E-01	4.75E-11	7.45E-09
ILMN_1869109		-8.17E-01	6.92E-10	5.26E-08
ILMN_1724907	NUDT3	-8.17E-01	5.14E-10	4.19E-08
ILMN_1678054	TRIM21	-8.18E-01	6.98E-06	9.29E-05
ILMN_2383774	TRAF3	-8.18E-01	2.53E-05	2.77E-04
ILMN_1751086	ATL3	-8.20E-01	1.09E-07	2.84E-06
ILMN_1656129	SLC39A10	-8.20E-01	8.65E-09	3.68E-07
ILMN_1811551	DERA	-8.20E-01	3.26E-05	3.44E-04
ILMN_1725105	EEF1E1	-8.21E-01	7.92E-12	2.03E-09
ILMN_1702783	LOC652595	-8.21E-01	2.23E-09	1.28E-07
ILMN_1739541	NMI	-8.21E-01	5.67E-07	1.11E-05
ILMN_1673450	DDN	-8.21E-01	9.89E-07	1.77E-05
ILMN_1699603	MRPL12	-8.21E-01	6.04E-07	1.17E-05
ILMN_1777139	MAK16	-8.21E-01	1.28E-07	3.24E-06
ILMN_1744059	DCTN6	-8.21E-01	6.07E-10	4.77E-08
ILMN_1665583	TUBB	-8.21E-01	1.36E-07	3.41E-06
ILMN_1671442	WDR43	-8.22E-01	1.27E-07	3.24E-06
ILMN_3247064	SNRNP40	-8.22E-01	8.49E-10	6.08E-08
ILMN_1738681	NUP62	-8.22E-01	2.42E-10	2.40E-08
ILMN_1807423	IGF2BP3	-8.22E-01	6.31E-09	2.89E-07
ILMN_2336109	L3MBTL2	-8.22E-01	4.68E-09	2.28E-07
ILMN_1682054	SRI	-8.22E-01	8.22E-09	3.54E-07
ILMN_1756162	EXOSC8	-8.23E-01	7.04E-06	9.36E-05
ILMN_2342271	BCL11A	-8.26E-01	2.13E-04	1.72E-03
ILMN_2386179	ZMYND8	-8.26E-01	5.52E-09	2.59E-07
ILMN_1725705	CLPP	-8.27E-01	3.78E-07	7.92E-06
ILMN_1772522	ZFP161	-8.27E-01	8.13E-12	2.08E-09
ILMN_3251629	EIF4A1	-8.27E-01	1.38E-06	2.33E-05
ILMN_1756445	PMF1	-8.28E-01	5.42E-14	8.25E-11
ILMN_1750088	VRK2	-8.28E-01	3.34E-08	1.08E-06
ILMN_1770692	WDR12	-8.28E-01	5.60E-07	1.10E-05
ILMN_1815115	CYC1	-8.30E-01	7.08E-07	1.33E-05
ILMN_2138801	TP73L	-8.31E-01	2.51E-05	2.75E-04
ILMN_2098325	C8orf33	-8.31E-01	3.83E-08	1.21E-06
ILMN_3307930	RAN	-8.31E-01	3.82E-07	7.99E-06
ILMN_2384241	TGFBR2	-8.33E-01	3.43E-03	1.88E-02
ILMN_2320250	NOL6	-8.34E-01	2.85E-05	3.07E-04
ILMN_1708009	LANCL2	-8.34E-01	3.60E-06	5.33E-05
ILMN_2316878	PTPRO	-8.34E-01	7.96E-05	7.38E-04
ILMN_3236945	PTPMT1	-8.35E-01	3.46E-08	1.11E-06
ILMN_1669484	WDR6	-8.36E-01	2.96E-08	9.82E-07
ILMN_1730917	KMO	-8.38E-01	5.26E-07	1.05E-05
ILMN_1780382	LOC653566	-8.38E-01	2.96E-09	1.58E-07
ILMN_1712305	CYBRD1	-8.38E-01	2.89E-09	1.55E-07
ILMN_1799725	DOCK2	-8.39E-01	6.67E-11	9.45E-09
ILMN_2075189	SLC35F2	-8.39E-01	1.53E-09	9.50E-08

ILMN_1810996	COL24A1	-8.39E-01	1.05E-02	4.83E-02
ILMN_3263329	GAR1	-8.40E-01	3.29E-07	7.08E-06
ILMN_1747052	ITGA4	-8.41E-01	4.98E-05	4.92E-04
ILMN_1757384	RAN	-8.41E-01	2.99E-06	4.54E-05
ILMN_1799280	BDH1	-8.42E-01	6.54E-08	1.85E-06
ILMN_2246956	BCL2	-8.42E-01	2.84E-03	1.60E-02
ILMN_3236061	ZNF783	-8.42E-01	2.00E-10	2.06E-08
ILMN_1727041	EWSR1	-8.42E-01	2.03E-10	2.07E-08
ILMN_3244893	BAG2	-8.43E-01	3.01E-10	2.80E-08
ILMN_1716445	LOC727761	-8.43E-01	5.61E-08	1.64E-06
ILMN_2333107	AES	-8.43E-01	5.68E-07	1.11E-05
ILMN_1670272	LRP10	-8.44E-01	3.23E-07	6.98E-06
ILMN_3287266	LOC100133328	-8.45E-01	1.39E-05	1.66E-04
ILMN_1676014	LOC728635	-8.45E-01	2.74E-09	1.49E-07
ILMN_1718334	ITPA	-8.46E-01	4.19E-10	3.58E-08
ILMN_1721035	MS4A6A	-8.47E-01	9.47E-05	8.56E-04
ILMN_1660871	NEK6	-8.47E-01	2.05E-09	1.20E-07
ILMN_1736311	POU2F2	-8.52E-01	8.36E-05	7.68E-04
ILMN_2126239	SMG5	-8.52E-01	6.66E-08	1.87E-06
ILMN_1746457	GTF3C2	-8.53E-01	1.05E-10	1.32E-08
ILMN_1670796	EXOSC10	-8.54E-01	1.28E-09	8.30E-08
ILMN_2364062	THOC4	-8.54E-01	3.11E-08	1.02E-06
ILMN_2365686	ALG8	-8.54E-01	7.88E-08	2.17E-06
ILMN_1677793	P2RX5	-8.54E-01	3.98E-05	4.07E-04
ILMN_2189993	MRPS35	-8.54E-01	1.17E-10	1.41E-08
ILMN_3270641	HNRNPH3	-8.55E-01	7.59E-11	1.04E-08
ILMN_1730773	SNORA70	-8.56E-01	1.93E-06	3.09E-05
ILMN_1725121	XPO1	-8.56E-01	5.44E-12	1.58E-09
ILMN_1654920	HNRPH3	-8.56E-01	7.16E-08	1.99E-06
ILMN_1720113	PTPRO	-8.56E-01	2.12E-05	2.39E-04
ILMN_1664424	ZBED5	-8.56E-01	2.81E-10	2.69E-08
ILMN_1765701	LOC399942	-8.57E-01	8.74E-05	7.98E-04
ILMN_3307729	CXXC5	-8.57E-01	2.08E-04	1.69E-03
ILMN_1756086	INTS3	-8.57E-01	6.44E-10	4.99E-08
ILMN_2388466	TIA1	-8.58E-01	9.67E-12	2.36E-09
ILMN_2330267	ABCE1	-8.58E-01	2.38E-07	5.41E-06
ILMN_1781803	FIZ1	-8.59E-01	2.43E-06	3.78E-05
ILMN_1815723	NUP35	-8.59E-01	1.92E-09	1.13E-07
ILMN_1657873	XPO4	-8.60E-01	1.74E-08	6.46E-07
ILMN_1652407	ZMYND8	-8.61E-01	7.56E-08	2.09E-06
ILMN_2192683	DHX37	-8.62E-01	3.26E-12	1.15E-09
ILMN_3305055	TP63	-8.62E-01	3.07E-06	4.64E-05
ILMN_1706839	TCERG1	-8.62E-01	4.46E-09	2.20E-07
ILMN_1671427	FBXW4	-8.63E-01	4.02E-12	1.33E-09
ILMN_1760280	NXT1	-8.63E-01	1.76E-10	1.87E-08
ILMN_1761519	EIF4G2	-8.64E-01	1.03E-06	1.84E-05

ILMN_1693045	TMED1	-8.64E-01	2.36E-12	9.43E-10
ILMN_3205271	LOC100132863	-8.65E-01	1.62E-04	1.36E-03
ILMN_1687484	ZFX	-8.66E-01	5.37E-11	8.00E-09
ILMN_1745256	CXXC5	-8.66E-01	1.23E-05	1.50E-04
ILMN_1668012	SLC25A13	-8.67E-01	1.86E-09	1.10E-07
ILMN_1771966	BCCIP	-8.68E-01	1.44E-10	1.63E-08
ILMN_3277715	LOC389873	-8.68E-01	3.83E-10	3.36E-08
ILMN_1736015	PHF17	-8.68E-01	5.79E-12	1.62E-09
ILMN_3272603	FAM60A	-8.69E-01	8.70E-10	6.15E-08
ILMN_1658327	BAZ1A	-8.70E-01	2.63E-08	8.91E-07
ILMN_1681101	MARCH1	-8.70E-01	1.04E-02	4.81E-02
ILMN_2398995	MRPL24	-8.70E-01	4.61E-09	2.24E-07
ILMN_3238845	FAM165B	-8.70E-01	1.04E-07	2.76E-06
ILMN_1686968	ZNF362	-8.70E-01	2.72E-06	4.17E-05
ILMN_1767006	PSMB8	-8.71E-01	1.59E-08	6.01E-07
ILMN_3235514	GPR183	-8.72E-01	4.99E-09	2.39E-07
ILMN_1710954	LOC283932	-8.72E-01	3.61E-11	6.12E-09
ILMN_1732537	HEATR2	-8.73E-01	2.76E-10	2.65E-08
ILMN_1772686	FGD3	-8.73E-01	5.04E-04	3.64E-03
ILMN_1707084	UBE2D4	-8.74E-01	4.58E-10	3.82E-08
ILMN_1781373	IFIH1	-8.74E-01	2.77E-03	1.57E-02
ILMN_2110281	UFC1	-8.76E-01	6.74E-11	9.47E-09
ILMN_3207933	LOC647150	-8.76E-01	1.92E-08	7.03E-07
ILMN_1704369	LIMA1	-8.77E-01	9.19E-03	4.33E-02
ILMN_1782247	KAT2A	-8.78E-01	6.19E-06	8.40E-05
ILMN_1703441	ZNF593	-8.78E-01	3.16E-12	1.13E-09
ILMN_3283742	LOC646791	-8.79E-01	1.04E-11	2.48E-09
ILMN_2318638	TGIF1	-8.80E-01	4.51E-04	3.31E-03
ILMN_1772521	MTHFD1L	-8.80E-01	1.19E-03	7.58E-03
ILMN_2180371	C12orf24	-8.80E-01	1.02E-03	6.66E-03
ILMN_1772706	C10orf32	-8.81E-01	9.34E-07	1.69E-05
ILMN_2281069	GPATCH4	-8.81E-01	2.32E-10	2.32E-08
ILMN_1712347	LOC644422	-8.82E-01	1.44E-04	1.23E-03
ILMN_2175075	SFRS4	-8.82E-01	3.02E-08	9.96E-07
ILMN_1676846	ABCE1	-8.82E-01	6.00E-09	2.77E-07
ILMN_3246608	CENPV	-8.82E-01	1.19E-04	1.04E-03
ILMN_1714809	RPIA	-8.84E-01	5.72E-13	3.86E-10
ILMN_2143795	MGC4677	-8.84E-01	2.31E-03	1.34E-02
ILMN_1683475	TOMM40	-8.87E-01	6.53E-08	1.84E-06
ILMN_2365111	MAP4K1	-8.87E-01	5.24E-12	1.54E-09
ILMN_1815878	C11orf59	-8.87E-01	7.26E-07	1.36E-05
ILMN_2192281	CARD8	-8.89E-01	4.72E-10	3.91E-08
ILMN_1789775	WDR74	-8.89E-01	2.49E-09	1.39E-07
ILMN_1679185	LEF1	-8.89E-01	2.19E-06	3.44E-05
ILMN_1779404	PUF60	-8.89E-01	6.15E-08	1.76E-06
ILMN_3224204	PSMG4	-8.92E-01	3.40E-10	3.06E-08

ILMN_2189424	MRPL20	-8.92E-01	5.58E-09	2.60E-07
ILMN_1778561	WEE1	-8.92E-01	6.04E-06	8.24E-05
ILMN_2395728	HNRPUL1	-8.93E-01	3.66E-11	6.16E-09
ILMN_1729691	SLC16A6	-8.93E-01	1.38E-07	3.45E-06
ILMN_3265797	LOC100130561	-8.94E-01	6.73E-05	6.39E-04
ILMN_1733932	SNUPN	-8.94E-01	9.93E-11	1.27E-08
ILMN_1663195	MCM7	-8.96E-01	1.18E-07	3.04E-06
ILMN_1742238	SET	-8.97E-01	1.37E-04	1.18E-03
ILMN_1758823	SMARCB1	-8.98E-01	1.32E-08	5.16E-07
ILMN_1712944	AES	-8.98E-01	1.30E-06	2.21E-05
ILMN_1789702	GBE1	-8.98E-01	1.22E-08	4.86E-07
ILMN_1655077	PRDM1	-8.99E-01	8.95E-04	5.95E-03
ILMN_1758915	PDCD2	-8.99E-01	7.19E-11	9.98E-09
ILMN_2061043	CD48	-9.00E-01	6.64E-04	4.61E-03
ILMN_1713143	MRPL3	-9.00E-01	2.15E-09	1.24E-07
ILMN_1775192	BCLAF1	-9.01E-01	9.87E-10	6.78E-08
ILMN_1737514	KYNU	-9.02E-01	2.29E-07	5.26E-06
ILMN_1767658	RRS1	-9.02E-01	2.05E-11	4.08E-09
ILMN_1810334	COMMD7	-9.04E-01	7.87E-11	1.06E-08
ILMN_2398847	ARHGAP17	-9.05E-01	5.17E-03	2.67E-02
ILMN_1812967	CNPY3	-9.05E-01	5.83E-12	1.62E-09
ILMN_1681301	AIM2	-9.07E-01	2.81E-04	2.20E-03
ILMN_2101885	TUBB	-9.08E-01	6.14E-08	1.76E-06
ILMN_1760849	NETO2	-9.08E-01	1.09E-05	1.35E-04
ILMN_2103841	AIP	-9.08E-01	3.11E-12	1.13E-09
ILMN_1740165	C14orf102	-9.10E-01	1.68E-07	4.05E-06
ILMN_1718734	MLLT6	-9.13E-01	3.37E-05	3.54E-04
ILMN_1708414	GNL3L	-9.15E-01	2.32E-10	2.32E-08
ILMN_1679483	INTS10	-9.16E-01	2.57E-08	8.72E-07
ILMN_1718712	C20orf177	-9.16E-01	3.97E-12	1.33E-09
ILMN_2056002	LOC723972	-9.16E-01	9.01E-10	6.35E-08
ILMN_1744308	DHX33	-9.17E-01	7.20E-10	5.44E-08
ILMN_3260070	LOC100128266	-9.17E-01	1.04E-06	1.85E-05
ILMN_3235853	S1PR1	-9.19E-01	6.25E-10	4.87E-08
ILMN_1760027	WAS	-9.19E-01	4.49E-10	3.77E-08
ILMN_1769158	ISOC2	-9.22E-01	1.52E-08	5.80E-07
ILMN_1668417	WASPIP	-9.23E-01	3.04E-07	6.64E-06
ILMN_2185884	DHRS4	-9.24E-01	9.29E-11	1.20E-08
ILMN_1711005	CDC25A	-9.24E-01	4.85E-06	6.85E-05
ILMN_1771051	RPL29	-9.25E-01	4.44E-06	6.38E-05
ILMN_2093500	ZBED5	-9.26E-01	3.25E-11	5.62E-09
ILMN_1681590	LARP1	-9.26E-01	3.54E-06	5.26E-05
ILMN_1802653	EBI3	-9.27E-01	1.77E-10	1.88E-08
ILMN_1751744	ANKRD41	-9.27E-01	3.88E-06	5.68E-05
ILMN_1786015	CTCF	-9.30E-01	2.26E-09	1.29E-07
ILMN_1746393	TSEN2	-9.31E-01	9.10E-11	1.19E-08

ILMN_1706539	KDM3B	-9.32E-01	1.43E-09	9.05E-08
ILMN_1651557	KDELC2	-9.34E-01	2.52E-03	1.45E-02
ILMN_1770206	GEMIN4	-9.36E-01	1.40E-05	1.67E-04
ILMN_2121282	MRPS18B	-9.36E-01	1.43E-09	9.05E-08
ILMN_1737517	RPL29	-9.36E-01	1.80E-05	2.08E-04
ILMN_1736982	PHACTR1	-9.37E-01	1.67E-07	4.04E-06
ILMN_2155516	QTRTD1	-9.38E-01	4.38E-08	1.34E-06
ILMN_3191695	LOC100128266	-9.39E-01	5.49E-07	1.08E-05
ILMN_1742031	IFRD2	-9.40E-01	3.45E-07	7.36E-06
ILMN_1743711	LOC650215	-9.41E-01	6.03E-09	2.78E-07
ILMN_1720476	PHF2	-9.41E-01	1.35E-13	1.48E-10
ILMN_1806946	UBTF	-9.41E-01	1.34E-11	3.03E-09
ILMN_1715214	PTPN7	-9.42E-01	3.49E-06	5.19E-05
ILMN_1806845	ALG3	-9.43E-01	9.09E-09	3.83E-07
ILMN_1760011	GTF2IRD2B	-9.43E-01	1.49E-06	2.49E-05
ILMN_1809866	WDR74	-9.43E-01	2.10E-08	7.50E-07
ILMN_1651346	TICAM2	-9.43E-01	8.11E-04	5.47E-03
ILMN_1742618	XAF1	-9.44E-01	1.37E-06	2.31E-05
ILMN_1655684	SARS2	-9.44E-01	1.20E-08	4.78E-07
ILMN_1685413	ALG8	-9.45E-01	4.01E-10	3.47E-08
ILMN_1751431	WIBG	-9.46E-01	7.09E-08	1.97E-06
ILMN_2264011	GRAP	-9.47E-01	3.39E-06	5.07E-05
ILMN_1744212	INPP5D	-9.47E-01	6.85E-05	6.49E-04
ILMN_1746517	KYNU	-9.48E-01	5.73E-08	1.66E-06
ILMN_2352293	PRDM10	-9.48E-01	5.08E-07	1.02E-05
ILMN_1824362		-9.49E-01	6.18E-04	4.34E-03
ILMN_1756676	PHF19	-9.49E-01	1.40E-09	9.00E-08
ILMN_3251526	USP13	-9.49E-01	9.71E-13	5.31E-10
ILMN_2366334	FERMT3	-9.50E-01	1.59E-04	1.34E-03
ILMN_2355953	LILRB4	-9.53E-01	6.30E-04	4.41E-03
ILMN_1701244	ITFG2	-9.54E-01	1.92E-07	4.54E-06
ILMN_1688959	CD27	-9.55E-01	3.85E-03	2.07E-02
ILMN_1808196	GSTO1	-9.56E-01	1.35E-07	3.40E-06
ILMN_2407482	ITPA	-9.57E-01	1.61E-09	9.85E-08
ILMN_2389582	HNRNPL	-9.58E-01	1.85E-05	2.12E-04
ILMN_1665423	ZFP91	-9.60E-01	4.58E-12	1.42E-09
ILMN_1676026	MRPS26	-9.62E-01	1.36E-07	3.42E-06
ILMN_1811049	POU2AF1	-9.63E-01	2.08E-03	1.23E-02
ILMN_1703949	KPNB1	-9.64E-01	3.21E-09	1.70E-07
ILMN_1696846	LOC541471	-9.66E-01	1.45E-03	8.94E-03
ILMN_3241169	C3orf75	-9.66E-01	2.93E-11	5.22E-09
ILMN_3307926	ADRBK1	-9.69E-01	4.87E-09	2.34E-07
ILMN_1855278		-9.70E-01	1.12E-04	9.86E-04
ILMN_1683120	UNG	-9.73E-01	3.34E-04	2.56E-03
ILMN_1781479	SUV39H1	-9.73E-01	3.53E-09	1.83E-07
ILMN_1773125	ENTPD1	-9.74E-01	9.25E-07	1.67E-05

ILMN_1769412	RAPGEF1	-9.75E-01	1.31E-04	1.13E-03
ILMN_3245066	DENND4B	-9.75E-01	3.91E-05	4.01E-04
ILMN_1713751	ADAM19	-9.76E-01	8.52E-06	1.10E-04
ILMN_2377385	SERPINA9	-9.77E-01	6.05E-04	4.26E-03
ILMN_1788017	HSH2D	-9.78E-01	1.89E-06	3.03E-05
ILMN_1768816	TMPO	-9.80E-01	2.93E-08	9.71E-07
ILMN_1665483	KIAA0020	-9.80E-01	2.51E-10	2.46E-08
ILMN_1735461	DDX21	-9.81E-01	1.41E-09	9.00E-08
ILMN_2376205	LTB	-9.81E-01	9.24E-04	6.12E-03
ILMN_1745471	IRF9	-9.83E-01	8.43E-08	2.29E-06
ILMN_1795285	PHF15	-9.83E-01	1.24E-12	6.21E-10
ILMN_1764577	MFNG	-9.83E-01	1.24E-04	1.08E-03
ILMN_1655444	LOC728492	-9.83E-01	6.85E-10	5.23E-08
ILMN_1747195	PSMB8	-9.84E-01	2.67E-11	4.94E-09
ILMN_1703692	LOC647000	-9.86E-01	2.57E-07	5.75E-06
ILMN_1693242	ZNF296	-9.86E-01	1.97E-10	2.03E-08
ILMN_2213136	LEF1	-9.86E-01	1.41E-07	3.51E-06
ILMN_1713749	CORO1A	-9.91E-01	5.99E-06	8.18E-05
ILMN_1856315		-9.92E-01	6.67E-10	5.12E-08
ILMN_1716736	CD80	-9.92E-01	4.08E-08	1.28E-06
ILMN_1695058	SLC38A5	-9.93E-01	2.88E-06	4.38E-05
ILMN_2148785	GBP1	-9.94E-01	7.79E-11	1.06E-08
ILMN_2400947	CUGBP2	-9.94E-01	2.14E-06	3.38E-05
ILMN_2087692	CYBRD1	-9.95E-01	5.54E-13	3.86E-10
ILMN_1757827	ECOP	-9.96E-01	3.12E-09	1.65E-07
ILMN_1722059	SAFB	-9.96E-01	2.73E-07	6.04E-06
ILMN_1751079	TAP1	-9.97E-01	1.70E-07	4.10E-06
ILMN_1728426	INPPL1	-9.98E-01	3.05E-11	5.35E-09
ILMN_1688034	COIL	-9.99E-01	5.36E-11	8.00E-09
ILMN_1815190	METTL1	-9.99E-01	5.63E-12	1.60E-09
ILMN_2415011	DCTD	-1.00E+00	5.53E-10	4.41E-08
ILMN_3240685	INO80D	-1.00E+00	2.12E-09	1.23E-07
ILMN_3177271	LOC100129585	-1.00E+00	1.36E-09	8.82E-08
ILMN_1720282	NQO1	-1.00E+00	4.34E-05	4.37E-04
ILMN_2092664	ADSS	-1.00E+00	2.73E-13	2.45E-10
ILMN_3240321	AEN	-1.00E+00	2.55E-07	5.71E-06
ILMN_1729123	PPP2R4	-1.01E+00	2.90E-10	2.73E-08
ILMN_1683664	LOC650369	-1.01E+00	6.72E-09	3.00E-07
ILMN_1755862	PFAS	-1.01E+00	2.38E-11	4.55E-09
ILMN_1718610	ARHGAP17	-1.01E+00	2.35E-03	1.36E-02
ILMN_1719694	LOC729446	-1.01E+00	2.49E-12	9.80E-10
ILMN_3238889	RPRD2	-1.01E+00	8.67E-09	3.69E-07
ILMN_1815734	FCHSD2	-1.01E+00	8.18E-08	2.23E-06
ILMN_2148668	RCBTB2	-1.01E+00	7.03E-07	1.32E-05
ILMN_1782635	YARS2	-1.02E+00	9.37E-12	2.30E-09
ILMN_1682699	PBX2	-1.02E+00	6.71E-09	3.00E-07

ILMN_1707493	SNHG3-RCC1	-1.02E+00	1.26E-05	1.52E-04
ILMN_1690268	HNRPUL1	-1.02E+00	1.15E-11	2.65E-09
ILMN_1662878	PACAP	-1.02E+00	2.40E-09	1.35E-07
ILMN_1793894	ANAPC13	-1.02E+00	2.21E-10	2.21E-08
ILMN_1659888	PPP1R14B	-1.02E+00	2.56E-08	8.71E-07
ILMN_1718988	DAZAP2	-1.02E+00	6.30E-11	9.04E-09
ILMN_2125747	LOC606724	-1.02E+00	5.02E-06	7.06E-05
ILMN_1655011	SERF1B	-1.02E+00	3.05E-06	4.61E-05
ILMN_1787345	FKBP11	-1.02E+00	1.40E-10	1.60E-08
ILMN_1712755	LRRC41	-1.02E+00	3.98E-09	2.00E-07
ILMN_1710514	BCL3	-1.03E+00	3.01E-04	2.34E-03
ILMN_2227573	GSTO1	-1.03E+00	1.96E-06	3.13E-05
ILMN_1717490	RPL6	-1.03E+00	2.16E-05	2.42E-04
ILMN_1711514	COCH	-1.03E+00	4.18E-09	2.08E-07
ILMN_1695576	MRPL24	-1.03E+00	1.07E-08	4.37E-07
ILMN_1725642	SUMO3	-1.03E+00	4.13E-04	3.07E-03
ILMN_1657153	ACTR3	-1.03E+00	2.37E-07	5.41E-06
ILMN_1760247	CD70	-1.03E+00	1.14E-09	7.59E-08
ILMN_1770673	AKNA	-1.03E+00	2.80E-07	6.16E-06
ILMN_2226955	VOPP1	-1.03E+00	1.12E-11	2.60E-09
ILMN_1810467	PPP2R1A	-1.03E+00	3.85E-12	1.30E-09
ILMN_2390162	PHF11	-1.04E+00	5.75E-06	7.90E-05
ILMN_2193233	MGC29506	-1.04E+00	6.40E-08	1.82E-06
ILMN_1784227	MCRS1	-1.04E+00	7.32E-12	1.94E-09
ILMN_1673991	ATIC	-1.04E+00	8.20E-10	5.95E-08
ILMN_1886515		-1.04E+00	9.81E-06	1.24E-04
ILMN_1769245	GLIPR1	-1.04E+00	2.44E-04	1.94E-03
ILMN_1689327	LOC730534	-1.04E+00	8.46E-11	1.12E-08
ILMN_1710434	TBC1D10C	-1.04E+00	2.86E-09	1.53E-07
ILMN_1697409	TNFRSF14	-1.04E+00	2.87E-12	1.09E-09
ILMN_1701551	ABCA6	-1.04E+00	5.30E-03	2.73E-02
ILMN_2395204	SLTM	-1.04E+00	3.47E-07	7.38E-06
ILMN_1663447	HNRNPA1	-1.05E+00	6.69E-10	5.13E-08
ILMN_2370882	ACSL5	-1.05E+00	1.78E-15	9.37E-12
ILMN_1689800	MRTO4	-1.05E+00	1.12E-10	1.39E-08
ILMN_1726108	LASS2	-1.05E+00	3.99E-10	3.45E-08
ILMN_3299365	LOC729406	-1.05E+00	2.76E-09	1.50E-07
ILMN_1655557	INTS6	-1.05E+00	1.36E-10	1.57E-08
ILMN_1671257	DKC1	-1.05E+00	9.23E-10	6.48E-08
ILMN_1756669	POGK	-1.05E+00	5.15E-11	7.90E-09
ILMN_1665943	MAP4K1	-1.05E+00	7.88E-11	1.06E-08
ILMN_2412549	GAR1	-1.06E+00	1.27E-13	1.42E-10
ILMN_1708382	C3orf75	-1.06E+00	4.03E-12	1.33E-09
ILMN_1667893	TNS3	-1.06E+00	9.15E-11	1.19E-08
ILMN_1802753	TSSC4	-1.06E+00	2.45E-11	4.62E-09
ILMN_1703891	TBC1D9	-1.06E+00	1.01E-08	4.19E-07

ILMN_1684446	SPAG7	-1.06E+00	6.74E-11	9.47E-09
ILMN_2390299	PSMB8	-1.06E+00	7.25E-11	1.00E-08
ILMN_1744649	PSMB5	-1.06E+00	1.29E-07	3.26E-06
ILMN_1694213	PLEKHO1	-1.06E+00	1.41E-11	3.13E-09
ILMN_1764964	IFNGR2	-1.06E+00	4.19E-07	8.63E-06
ILMN_1768662	UCK2	-1.06E+00	1.39E-10	1.59E-08
ILMN_1788931	DOCK8	-1.06E+00	2.99E-08	9.87E-07
ILMN_1708059	USP13	-1.06E+00	6.03E-08	1.73E-06
ILMN_2167922	TRMT5	-1.07E+00	1.19E-09	7.87E-08
ILMN_1797341	ARID1A	-1.07E+00	6.47E-10	5.00E-08
ILMN_1723486	HK2	-1.07E+00	6.35E-07	1.22E-05
ILMN_1776723	PHF11	-1.07E+00	7.51E-06	9.88E-05
ILMN_1782704	CD19	-1.07E+00	2.80E-05	3.02E-04
ILMN_1743397	PIGW	-1.07E+00	9.27E-07	1.68E-05
ILMN_2392352	CTPS2	-1.07E+00	1.38E-09	8.90E-08
ILMN_2155172	BRIX1	-1.08E+00	1.07E-11	2.49E-09
ILMN_3230435	LOC729086	-1.08E+00	9.67E-09	4.03E-07
ILMN_1797731	MS4A6A	-1.08E+00	2.86E-05	3.07E-04
ILMN_1677765	LRP8	-1.08E+00	1.25E-07	3.20E-06
ILMN_2087575	ZC3H4	-1.08E+00	2.31E-13	2.15E-10
ILMN_1795822	DIS3L	-1.08E+00	5.43E-12	1.58E-09
ILMN_2383306	GPATCH4	-1.08E+00	8.09E-08	2.22E-06
ILMN_1776080	GTPBP6	-1.08E+00	1.54E-09	9.50E-08
ILMN_2355665	MTP18	-1.08E+00	2.83E-11	5.10E-09
ILMN_1730084	COMT	-1.09E+00	1.26E-05	1.52E-04
ILMN_2400500	LASS2	-1.09E+00	1.63E-10	1.80E-08
ILMN_1742230	BAZ1A	-1.09E+00	2.99E-08	9.89E-07
ILMN_2369580	C16orf35	-1.09E+00	1.08E-05	1.34E-04
ILMN_2154115	PSD4	-1.09E+00	1.15E-10	1.40E-08
ILMN_2124951	RBMX	-1.09E+00	3.78E-09	1.92E-07
ILMN_1674390	PRKAR1B	-1.09E+00	4.56E-05	4.57E-04
ILMN_1903914		-1.09E+00	6.17E-06	8.39E-05
ILMN_1657993	ADNP	-1.10E+00	7.23E-12	1.93E-09
ILMN_1690101	FAIM	-1.10E+00	8.60E-10	6.12E-08
ILMN_2372413	BID	-1.10E+00	1.50E-07	3.72E-06
ILMN_1658800	BRPF3	-1.10E+00	1.61E-12	7.59E-10
ILMN_1728984	PA2G4	-1.10E+00	1.07E-09	7.22E-08
ILMN_1804601	LOC649923	-1.10E+00	2.55E-06	3.94E-05
ILMN_1807372	ADORA2A	-1.10E+00	9.32E-06	1.18E-04
ILMN_2262044	PARP10	-1.10E+00	1.88E-10	1.95E-08
ILMN_1753745	HDDC2	-1.11E+00	4.26E-10	3.61E-08
ILMN_1656628	WDR4	-1.11E+00	3.33E-08	1.08E-06
ILMN_1799467	SAMD9L	-1.11E+00	6.99E-05	6.60E-04
ILMN_1814282	ISG20L1	-1.11E+00	1.27E-06	2.18E-05
ILMN_1759008	ZNF689	-1.11E+00	9.31E-12	2.30E-09
ILMN_2058141	HMGN2	-1.12E+00	3.66E-11	6.16E-09

ILMN_1696004	LRRK1	-1.12E+00	2.18E-07	5.04E-06
ILMN_1677085	RGS19	-1.12E+00	4.57E-08	1.39E-06
ILMN_1673369	SEPHS1	-1.12E+00	2.12E-13	2.08E-10
ILMN_1670723	MSL3	-1.12E+00	6.40E-04	4.47E-03
ILMN_1734833	NBN	-1.12E+00	4.60E-12	1.42E-09
ILMN_3234615	LOC728650	-1.12E+00	1.06E-11	2.49E-09
ILMN_3208233	LOC100131735	-1.12E+00	1.97E-10	2.03E-08
ILMN_1811029	TLK1	-1.12E+00	6.89E-09	3.07E-07
ILMN_1657381	RASSF6	-1.12E+00	6.09E-10	4.77E-08
ILMN_1796339	PLEKHA2	-1.12E+00	4.03E-08	1.26E-06
ILMN_1710017	CD79B	-1.12E+00	3.51E-03	1.92E-02
ILMN_1733937	MMD	-1.13E+00	1.10E-05	1.37E-04
ILMN_2366212	CD79B	-1.13E+00	1.95E-04	1.60E-03
ILMN_1663916	ARHGAP9	-1.13E+00	3.63E-09	1.86E-07
ILMN_2054233	SENP6	-1.13E+00	2.86E-10	2.72E-08
ILMN_1785439	CD79B	-1.13E+00	6.12E-04	4.30E-03
ILMN_2193591	UNC93B1	-1.13E+00	1.67E-07	4.03E-06
ILMN_1804448	MSI2	-1.13E+00	6.95E-11	9.71E-09
ILMN_2410771	KEAP1	-1.13E+00	2.19E-08	7.74E-07
ILMN_1741957	RABEPK	-1.14E+00	1.35E-12	6.71E-10
ILMN_1686920	CCDC58	-1.14E+00	4.86E-10	4.00E-08
ILMN_3306997	METTL1	-1.14E+00	1.44E-09	9.09E-08
ILMN_1792681	CCDC86	-1.14E+00	4.63E-12	1.42E-09
ILMN_2091590	ANKRD41	-1.14E+00	3.97E-06	5.80E-05
ILMN_1790962	RINL	-1.14E+00	4.98E-07	9.97E-06
ILMN_1745420	PHF19	-1.15E+00	2.62E-08	8.87E-07
ILMN_1660754	C13orf25	-1.15E+00	5.90E-12	1.62E-09
ILMN_1790891	CKAP4	-1.15E+00	5.08E-06	7.12E-05
ILMN_1742224	SLTM	-1.15E+00	7.50E-09	3.30E-07
ILMN_1659913	ISG20	-1.15E+00	7.37E-05	6.90E-04
ILMN_1800787	RFTN1	-1.15E+00	1.46E-06	2.44E-05
ILMN_2375418	DPH2	-1.15E+00	1.85E-12	8.17E-10
ILMN_1669674	CNPY3	-1.16E+00	1.74E-12	7.85E-10
ILMN_2049536	TRPV2	-1.16E+00	2.68E-08	9.04E-07
ILMN_2242937	ARSB	-1.17E+00	9.44E-13	5.26E-10
ILMN_1658426	WNT10A	-1.17E+00	5.94E-05	5.73E-04
ILMN_1800638	CUGBP2	-1.17E+00	3.76E-06	5.53E-05
ILMN_2352303	RASSF2	-1.17E+00	8.44E-06	1.09E-04
ILMN_1666902	GPR114	-1.17E+00	2.40E-04	1.91E-03
ILMN_1672417	PTPRCAP	-1.18E+00	9.81E-05	8.80E-04
ILMN_1802456	DCTD	-1.18E+00	1.41E-11	3.13E-09
ILMN_1723912	IFI44L	-1.18E+00	3.55E-06	5.26E-05
ILMN_3247723	NOP16	-1.18E+00	3.03E-08	9.98E-07
ILMN_1803652	C9orf91	-1.18E+00	1.40E-05	1.67E-04
ILMN_1655654	MPDU1	-1.19E+00	5.15E-10	4.19E-08
ILMN_1726842	TYW3	-1.19E+00	6.20E-10	4.85E-08

ILMN_3271555	LOC100130458	-1.19E+00	3.63E-03	1.97E-02
ILMN_1679800	BRIX1	-1.19E+00	1.02E-11	2.43E-09
ILMN_1738523	MYD88	-1.19E+00	8.89E-06	1.14E-04
ILMN_1770824	ARHGAP4	-1.19E+00	1.27E-08	5.01E-07
ILMN_2375032	BEND3	-1.20E+00	6.04E-07	1.17E-05
ILMN_1759991	MGC3731	-1.20E+00	4.85E-09	2.33E-07
ILMN_1702585	LOC646817	-1.20E+00	7.67E-06	1.01E-04
ILMN_1785268	CD58	-1.20E+00	2.66E-07	5.92E-06
ILMN_1731518	PLD6	-1.20E+00	2.38E-10	2.37E-08
ILMN_1737110	LOC651957	-1.21E+00	6.53E-06	8.77E-05
ILMN_1689002	DTX1	-1.21E+00	1.26E-03	7.94E-03
ILMN_1664750	TMBIM4	-1.21E+00	3.28E-11	5.65E-09
ILMN_2363106	RBM23	-1.21E+00	2.19E-11	4.29E-09
ILMN_1797074	EMG1	-1.21E+00	1.51E-10	1.69E-08
ILMN_2410772	KEAP1	-1.22E+00	6.48E-08	1.83E-06
ILMN_1803811	TRIB1	-1.22E+00	7.90E-10	5.82E-08
ILMN_1765621	HDGF	-1.23E+00	2.10E-08	7.50E-07
ILMN_2231928	MX2	-1.23E+00	9.18E-05	8.33E-04
ILMN_1776052	LOC148915	-1.23E+00	1.08E-07	2.83E-06
ILMN_1796762	CCDC102A	-1.23E+00	1.34E-07	3.39E-06
ILMN_1735180	NCSTN	-1.24E+00	7.16E-08	1.99E-06
ILMN_1780756	RBM23	-1.24E+00	3.14E-09	1.66E-07
ILMN_2147517	CD58	-1.25E+00	1.09E-06	1.92E-05
ILMN_1757730	TTC27	-1.25E+00	2.84E-12	1.09E-09
ILMN_1691071	FCRLA	-1.25E+00	1.69E-09	1.03E-07
ILMN_1662964	PRMT3	-1.25E+00	8.44E-12	2.13E-09
ILMN_1704055	HSPC111	-1.26E+00	1.91E-11	3.90E-09
ILMN_1673917	GTF2I	-1.26E+00	1.41E-07	3.51E-06
ILMN_1754121	CSK	-1.26E+00	8.90E-10	6.28E-08
ILMN_1661337	SRM	-1.26E+00	3.17E-05	3.36E-04
ILMN_2330307	SLC43A3	-1.27E+00	2.68E-10	2.60E-08
ILMN_1709683	RASSF2	-1.27E+00	1.44E-05	1.71E-04
ILMN_1717313	NFKBIE	-1.27E+00	7.83E-07	1.45E-05
ILMN_1723235	DUS3L	-1.28E+00	7.89E-10	5.82E-08
ILMN_1658486	MRPL54	-1.28E+00	4.49E-08	1.37E-06
ILMN_1749629	CUL1	-1.28E+00	4.63E-12	1.42E-09
ILMN_2326953	LAT2	-1.28E+00	2.82E-06	4.29E-05
ILMN_1754272	GINS3	-1.28E+00	7.80E-10	5.78E-08
ILMN_2388547	EPSTI1	-1.29E+00	4.21E-03	2.24E-02
ILMN_1750518	THOC4	-1.29E+00	2.79E-06	4.25E-05
ILMN_1669113	ATF5	-1.29E+00	1.90E-05	2.18E-04
ILMN_1773388	C13orf18	-1.30E+00	7.09E-08	1.97E-06
ILMN_1702301	DOCK10	-1.31E+00	5.41E-08	1.59E-06
ILMN_1720124	RCC2	-1.31E+00	7.39E-08	2.05E-06
ILMN_1775677	TYSND1	-1.31E+00	3.21E-14	6.21E-11
ILMN_3176090	LOC100130919	-1.31E+00	2.49E-08	8.52E-07

ILMN_1759075	TNFRSF13B	-1.32E+00	2.25E-08	7.88E-07
ILMN_1768110	ZAK	-1.32E+00	2.37E-05	2.62E-04
ILMN_1813938	CHCHD4	-1.33E+00	3.33E-09	1.75E-07
ILMN_1662026	BTK	-1.33E+00	1.58E-08	5.98E-07
ILMN_1668277	BLK	-1.34E+00	1.42E-13	1.52E-10
ILMN_1726030	GPX7	-1.34E+00	1.09E-08	4.45E-07
ILMN_1801710	APBB1IP	-1.34E+00	2.11E-08	7.51E-07
ILMN_1684293	ANP32B	-1.35E+00	3.78E-09	1.92E-07
ILMN_1682799	STAMBPL1	-1.35E+00	2.34E-04	1.88E-03
ILMN_1722948	LOC652495	-1.35E+00	6.09E-06	8.29E-05
ILMN_1697554	SASH3	-1.36E+00	5.88E-07	1.14E-05
ILMN_2340259	PDE4B	-1.36E+00	2.33E-07	5.32E-06
ILMN_2112049	DNLZ	-1.37E+00	7.07E-06	9.39E-05
ILMN_1731358	ZNF532	-1.37E+00	2.53E-07	5.67E-06
ILMN_1652754	ZNF428	-1.37E+00	1.68E-12	7.78E-10
ILMN_1782609	STAG2	-1.37E+00	5.58E-12	1.60E-09
ILMN_2255133	BCL11A	-1.38E+00	3.88E-03	2.09E-02
ILMN_1777233	E2F2	-1.38E+00	4.75E-14	7.74E-11
ILMN_1768176	CXorf26	-1.38E+00	6.79E-12	1.83E-09
ILMN_3238680	C7orf55	-1.39E+00	4.59E-08	1.39E-06
ILMN_1653001	CABLES1	-1.39E+00	1.06E-03	6.86E-03
ILMN_2049184	DNASE1L3	-1.39E+00	2.32E-04	1.86E-03
ILMN_3219806	LOC643384	-1.40E+00	1.28E-07	3.25E-06
ILMN_1727134	KLHDC5	-1.40E+00	6.38E-09	2.91E-07
ILMN_1803560	LAT2	-1.41E+00	8.45E-07	1.55E-05
ILMN_2176768	SEPHS1	-1.41E+00	3.09E-13	2.65E-10
ILMN_1752899	BCL11A	-1.41E+00	1.07E-02	4.91E-02
ILMN_1763386	BID	-1.41E+00	4.96E-10	4.08E-08
ILMN_1745820	RASSF6	-1.41E+00	3.12E-12	1.13E-09
ILMN_1763198	STAT6	-1.41E+00	3.62E-07	7.64E-06
ILMN_1727574	ZNF827	-1.41E+00	5.62E-14	8.29E-11
ILMN_1710216	AVEN	-1.42E+00	1.77E-09	1.06E-07
ILMN_2196550	C13orf18	-1.42E+00	5.70E-07	1.11E-05
ILMN_2248970	OAS2	-1.43E+00	3.40E-10	3.06E-08
ILMN_1748123	KLHL14	-1.43E+00	5.64E-05	5.48E-04
ILMN_1683026	PSMB10	-1.44E+00	9.69E-07	1.74E-05
ILMN_1676575	IKZF1	-1.44E+00	4.28E-04	3.16E-03
ILMN_1777998	ARHGAP25	-1.45E+00	3.47E-03	1.90E-02
ILMN_1738675	PTPN6	-1.45E+00	7.09E-05	6.68E-04
ILMN_1847822	KIAA0368	-1.46E+00	3.22E-07	6.97E-06
ILMN_1745374	IFI35	-1.46E+00	1.43E-04	1.22E-03
ILMN_1658407	SLC43A3	-1.46E+00	2.84E-11	5.10E-09
ILMN_1746864	PSCDBP	-1.46E+00	3.43E-13	2.74E-10
ILMN_1779252	TRIM22	-1.47E+00	4.85E-07	9.73E-06
ILMN_1796210	PPRC1	-1.47E+00	1.54E-13	1.58E-10
ILMN_1808299	IQSEC1	-1.48E+00	1.28E-03	8.05E-03

ILMN_1733579	EVI2A	-1.48E+00	1.27E-06	2.18E-05
ILMN_1806040	TYMS	-1.50E+00	5.14E-06	7.18E-05
ILMN_1655307	FAM136A	-1.51E+00	3.34E-14	6.21E-11
ILMN_2059549	SYK	-1.51E+00	1.97E-04	1.61E-03
ILMN_1754234	ZMYND11	-1.51E+00	7.51E-12	1.95E-09
ILMN_1710937	IFI16	-1.52E+00	2.15E-09	1.24E-07
ILMN_1772302	MTHFS	-1.52E+00	6.50E-09	2.95E-07
ILMN_1783285	CTPS	-1.52E+00	1.08E-07	2.84E-06
ILMN_1769911	SLC38A1	-1.52E+00	1.75E-12	7.85E-10
ILMN_1654118	BCL2L1	-1.52E+00	4.50E-06	6.45E-05
ILMN_1680501	GTF2IRD2B	-1.54E+00	1.33E-07	3.36E-06
ILMN_1714965	NFKB1	-1.54E+00	6.38E-06	8.61E-05
ILMN_1713990	TRIP6	-1.56E+00	1.68E-10	1.82E-08
ILMN_1713249	PHF19	-1.57E+00	1.80E-14	4.46E-11
ILMN_2085862	SLC15A3	-1.59E+00	5.85E-12	1.62E-09
ILMN_1756595	SH3TC1	-1.60E+00	3.75E-04	2.82E-03
ILMN_1768930	U2AF2	-1.60E+00	5.20E-11	7.90E-09
ILMN_1789830	CFLAR	-1.63E+00	9.62E-07	1.73E-05
ILMN_1746704	TRIM8	-1.64E+00	1.58E-14	4.39E-11
ILMN_3248910	MIR155HG	-1.66E+00	5.19E-03	2.68E-02
ILMN_2383305	GPATCH4	-1.66E+00	2.31E-11	4.47E-09
ILMN_1727045	RASGRP3	-1.66E+00	3.21E-13	2.65E-10
ILMN_1705247	ACSL5	-1.67E+00	1.18E-13	1.40E-10
ILMN_1662358	MX1	-1.67E+00	2.42E-03	1.40E-02
ILMN_1810431	LOC642299	-1.68E+00	1.13E-04	9.97E-04
ILMN_1804419	LRMP	-1.71E+00	4.20E-07	8.64E-06
ILMN_2366330	FERMT3	-1.72E+00	3.00E-06	4.54E-05
ILMN_1674386	PITX1	-1.72E+00	2.73E-14	5.86E-11
ILMN_1716596	NSMAF	-1.72E+00	1.02E-10	1.28E-08
ILMN_1691578	GTF3C6	-1.75E+00	5.29E-08	1.57E-06
ILMN_2379130	IRAK1	-1.75E+00	1.70E-10	1.83E-08
ILMN_1711894	MYB	-1.78E+00	1.47E-13	1.54E-10
ILMN_1732705	HCFC1	-1.82E+00	2.82E-09	1.53E-07
ILMN_1795762	PLEK	-1.82E+00	2.45E-05	2.70E-04
ILMN_3242271	GAPT	-1.82E+00	3.35E-09	1.75E-07
ILMN_2110908	MYC	-1.84E+00	1.68E-07	4.05E-06
ILMN_2384181	DHRS9	-1.84E+00	3.49E-05	3.64E-04
ILMN_1763452	EVI2B	-1.87E+00	1.86E-07	4.42E-06
ILMN_1775486	SSPN	-1.92E+00	3.25E-12	1.15E-09
ILMN_1746148	LRRC33	-1.98E+00	2.26E-12	9.16E-10
ILMN_1713759	UBE2J1	-1.99E+00	9.28E-16	6.26E-12
ILMN_1782729	CLECL1	-1.99E+00	1.76E-14	4.46E-11
ILMN_1675191	GAPT	-1.99E+00	1.25E-09	8.17E-08
ILMN_2067656	CCND2	-1.99E+00	9.13E-05	8.29E-04
ILMN_3207122	LOC644563	-2.00E+00	1.77E-09	1.06E-07
ILMN_1768534	BHLHB2	-2.01E+00	2.08E-08	7.48E-07

ILMN_1674063	OAS2	-2.03E+00	1.39E-08	5.39E-07
ILMN_1668822	BATF	-2.03E+00	3.35E-05	3.51E-04
ILMN_1667081	CCND2	-2.09E+00	2.81E-05	3.04E-04
ILMN_3240586	PLD6	-2.12E+00	3.78E-14	6.61E-11
ILMN_1680618	MYC	-2.12E+00	4.53E-07	9.17E-06
ILMN_1733998	DHRS9	-2.27E+00	8.03E-08	2.21E-06
ILMN_2168217	EBI2	-2.40E+00	1.15E-12	5.92E-10
ILMN_1768016	TNFRSF17	-2.50E+00	3.26E-13	2.65E-10
ILMN_1798706	EBI2	-2.58E+00	7.57E-09	3.32E-07
ILMN_1795298	GPER	-2.67E+00	8.10E-17	1.91E-12
ILMN_2384056	GPER	-2.69E+00	3.08E-14	6.21E-11
