# Supplementary material for: Novel HDAC inhibitors exhibit pre-clinical efficacy in lymphoma models and point to the importance of CDKN1A expression levels in mediating their anti-tumor response
Source: Oncotarget. 2014 Dec 30;6(7):5059–71. doi: 10.18632/oncotarget.3239 (PMC4467133; doi:10.18632/oncotarget.3239)
Supplement: Supplementary file 8 [file oncotarget-06-5059-s008.pdf]

Supplementary table 7

NAME	SIZE	ES	NES	NOM p-val	FDR q-val
Gene sets with FDR <0.25 for lTF-B high LC50/GI50 group					
DOUBLE_STRANDED_DNA_BINDING	32	0.7347026	2.0270953	0	0.005075325
STRUCTURE_SPECIFIC_DNA_BINDING	55	0.6731778	1.9978237	0	0.005107932
DNA_METABOLIC_PROCESS	245	0.55016303	2.012955	0	0.006142822
DNA_REPLICATION	94	0.59036887	1.9496695	0	0.008788857
RNA_PROCESSING	153	0.55411273	1.9577105	0	0.009195093
CHROMOSOMAL_PART	95	0.5744869	1.8870713	0	0.026286691
ATP_DEPENDENT_HELICASE_ACTIVITY	26	0.6988458	1.8649483	0	0.03346332
REGULATION_OF_CYTOKINE_SECRETION	16	0.7716953	1.8020217	0	0.039525654
SINGLE_STRANDED_DNA_BINDING	34	0.6678308	1.8181212	0.001855288	0.039799016
NUCLEASE_ACTIVITY	55	0.596678	1.803908	0	0.040882934
DNA_RECOMBINATION	45	0.62508947	1.8205118	0	0.041361578
NUCLEAR_CHROMOSOME	53	0.6173291	1.8340468	0	0.04137234
CHROMOSOME	122	0.54557407	1.8417734	0	0.041764557
DNA_REPAIR	122	0.54217386	1.8248954	0	0.042608682
DNA_DEPENDENT_DNA_REPLICATION	50	0.5996267	1.8050013	0	0.04322557
RIBONUCLEOPROTEIN_COMPLEX_BIOGENESIS_AND_ASSEMBLY	76	0.5470285	1.7617941	0	0.067170195
RESPONSE_TO_DNA_DAMAGE_STIMULUS	158	0.4977731	1.7332816	0	0.08594621
ONE_CARBON_COMPOUND_METABOLIC_PROCESS	26	0.66690725	1.728054	0.005747126	0.08600886
NUCLEAR_CHROMOSOME_PART	33	0.6389842	1.7337164	0.007326007	0.09057619
EARLY_ENDOSOME	18	0.7004526	1.7190182	0.007490637	0.09229749
MITOTIC_CELL_CYCLE	151	0.48972717	1.7136582	0	0.09372239
MEMBRANE_ENCLOSED_LUMEN	446	0.43693948	1.6951588	0	0.09420124
CHROMATIN	35	0.6150674	1.6886688	0.003809524	0.09468754
NUCLEOLUS	124	0.4995908	1.6816031	0	0.09688577
CHROMOSOMEPERICENTRIC_REGION	31	0.6257775	1.6954186	0.011472276	0.097634904
RESPONSE_TO_ENDOGENOUS_STIMULUS	196	0.47001868	1.6888983	0	0.09793029
STRIATED_MUSCLE_DEVELOPMENT	40	0.58483267	1.6823827	0.005514706	0.09918469
G1_S_TRANSITION_OF_MITOTIC_CELL_CYCLE	27	0.64805806	1.6973548	0.005649718	0.09985489
CELL_CYCLE_CHECKPOINT_GO_0000075	47	0.5829912	1.7026699	0.001865672	0.1020795
MITOCHONDRIAL_TRANSPORT	20	0.6757852	1.6730446	0.007692308	0.103056826
ORGANELLE_LUMEN	446	0.43693948	1.6980957	0	0.10325138
AMINO_ACID_METABOLIC_PROCESS	77	0.51623344	1.6495429	0.001848429	0.10826468
INDUCTION_OF_APOPTOSIS_BY_INTRACELLULAR_SIGNALS	23	0.6534315	1.649989	0.003738318	0.11051459
NUCLEAR_LUMEN	376	0.42894205	1.6448649	0	0.11072507
AMINE_BIOSYNTHETIC_PROCESS	15	0.7167386	1.6509827	0.021912351	0.112153776
MITOCHONDRION_ORGANIZATION_AND_BIOGENESIS	47	0.57079583	1.6617279	0.005514706	0.11227867
VITAMIN_METABOLIC_PROCESS	17	0.7055371	1.6584783	0.009451796	0.11308002
RIBONUCLEOPROTEIN_COMPLEX	142	0.48141262	1.6510482	0	0.115268566
SISTER_CHROMATID_SEGREGATION	17	0.6699416	1.6283293	0.023346303	0.116986774
PROTEIN_RNA_COMPLEX_ASSEMBLY	57	0.54494303	1.6512151	0.01171875	0.118474595
CELL_CYCLE_PROCESS	186	0.45833194	1.6344776	0	0.119239174
KINETOCHORE	25	0.6326055	1.6288016	0.011450382	0.11925718
TRNA_METABOLIC_PROCESS	18	0.66758925	1.6296372	0.017142856	0.12126205
RNA_POLYMERASE_ACTIVITY	16	0.6828411	1.6086717	0.019379845	0.13243556
DNA_DAMAGE_RESPONSESIGNAL_TRANSDUCTION	34	0.57068294	1.5870306	0.015473888	0.1326839
REGULATION_OF_TRANSLATIONAL_INITIATION	31	0.5754079	1.5929612	0.021072797	0.1330157
RNA_SPLICING	74	0.5023967	1.5847144	0.005847953	0.13350356
CONDENSED_CHROMOSOME	32	0.59673554	1.5941299	0.011070111	0.13402586
TRANSLATIONAL_INITIATION	39	0.5672492	1.5817118	0.013671875	0.13451777
MITOTIC_SISTER_CHROMATID_SEGREGATION	16	0.67795193	1.6113898	0.017274473	0.13488154
CELL_CYCLE_PHASE	166	0.45230126	1.5871047	0	0.13502118
M_PHASE	110	0.4782915	1.6088139	0.003584229	0.13526289
COFACTOR_METABOLIC_PROCESS	53	0.52726614	1.5991015	0.012433393	0.1360853
SPINDLE_MICROTUBULE	15	0.69930094	1.6036316	0.02	0.13642825
APOPTOSIS_GO	428	0.40805343	1.5965786	0	0.13651146
HELICASE_ACTIVITY	50	0.5400125	1.5941573	0.011450382	0.13661402
PROGRAMMED_CELL_DEATH	429	0.40802822	1.5877836	0	0.13667744
B_CELL_ACTIVATION	20	0.65771514	1.599256	0.018484289	0.1387479
M_PHASE_OF_MITOTIC_CELL_CYCLE	84	0.49465412	1.5749435	0.007272727	0.14061503
SEQUENCE_SPECIFIC_DNA_BINDING	54	0.53169054	1.5708342	0.011090573	0.14391632
ORGANELLE_ORGANIZATION_AND_BIOGENESIS	462	0.40146098	1.5688126	0	0.14408061
CHROMATIN_BINDING	32	0.58086514	1.5645003	0.022944551	0.14714049
CELL_CYCLE_GO_0007049	306	0.41309333	1.5614562	0	0.14856493
INTERPHASE_OF_MITOTIC_CELL_CYCLE	62	0.5017818	1.5485955	0.013133208	0.14997071
RESPONSE_TO_STRESS	499	0.39438713	1.5564121	0	0.15060912

REPLICATION_FORK	18	0.64677256	1.5498136	0.027422303	0.15079993
PIGMENT_METABOLIC_PROCESS	18	0.64125174	1.5510516	0.037807185	0.15124886
SPLICEOSOME	50	0.53658086	1.5572522	0.015594542	0.15165533
UBIQUITIN_LIGASE_COMPLEX	26	0.59388053	1.5533016	0.03468208	0.15254077
CHROMOSOME_ORGANIZATION_AND_BIOGENESIS	118	0.45652723	1.5450376	0.001865672	0.15257762
REGULATION_OF_PROTEIN_SECRETION	22	0.61587507	1.5513453	0.027777778	0.15322976
MITOSIS	81	0.48093566	1.5392519	0.003703704	0.15849005
ACTIVATION_OF_JNK_ACTIVITY	16	0.64451796	1.5234327	0.036036037	0.16054697
INTRINSIC_TO_ENDOPLASMIC_RETICULUM_MEMBRANE	24	0.59298354	1.5263046	0.044145875	0.16068478
MYOBLAST_DIFFERENTIATION	17	0.655071	1.5343491	0.0317757	0.16075912
DNA_HELICASE_ACTIVITY	25	0.59163743	1.5244471	0.039138943	0.16094778
AMINE_METABOLIC_PROCESS	140	0.45175692	1.5356654	0.001766785	0.16104656
CYTOKINESIS	19	0.6204527	1.5321852	0.048732944	0.1614018
DNA_DEPENDENT_ATPASE_ACTIVITY	22	0.60042423	1.5269935	0.037593983	0.16171418
SKELETAL_MUSCLE_DEVELOPMENT	31	0.56303287	1.528261	0.043977056	0.16209742
NITROGEN_COMPOUND_METABOLIC_PROCESS	153	0.43868417	1.5207331	0.001798561	0.16215146
AMINO_ACID_AND_DERIVATIVE_METABOLIC_PROCESS	100	0.4632675	1.5190852	0.001792115	0.16232125
ORGANIC_ACID_METABOLIC_PROCESS	177	0.42766288	1.5161282	0.003552398	0.16250747
INTEGRAL_TO_ENDOPLASMIC_RETICULUM_MEMBRANE	24	0.59298354	1.5288727	0.04411765	0.16346732
TRANSITION_METAL_ION_BINDING	110	0.4625469	1.5164765	0.005747126	0.16393869
COATED_VESICLE_MEMBRANE	16	0.63652444	1.5131648	0.0608365	0.16461235
RESPONSE_TO_OXIDATIVE_STRESS	45	0.5303407	1.5105205	0.035250463	0.16658737
RRNA_METABOLIC_PROCESS	16	0.6406418	1.5078859	0.05147059	0.16816126
CARBOXYLIC_ACID_METABOLIC_PROCESS	175	0.42522413	1.5057168	0.003629764	0.16898394
NUCLEOTIDYLTRANSFERASE_ACTIVITY	47	0.5163623	1.5037594	0.027027028	0.16978605
ENDOMEMBRANE_SYSTEM	217	0.40537408	1.4745731	0	0.16997626
PIGMENT_BIOSYNTHETIC_PROCESS	17	0.6426637	1.4748536	0.06284658	0.17109579
POSITIVE_REGULATION_OF_JNK_ACTIVITY	18	0.6247988	1.4899095	0.04315197	0.17234296
PROTEIN_SECRETION	32	0.53361183	1.4750489	0.0455408	0.17242451
NUCLEOPLASM	270	0.39867193	1.4858909	0	0.1725915
SMALL_NUCLEAR_RIBONUCLEOPROTEIN_COMPLEX	22	0.598186	1.4883114	0.041970804	0.172787
MRNA_PROCESSING_GO_0006397	55	0.4976383	1.487163	0.03460838	0.17283802
CELL_CYCLE_ARREST_GO_0007050	57	0.49160698	1.4905406	0.016917294	0.17325954
TUBULIN_BINDING	46	0.5106246	1.4754866	0.029850746	0.17336324
RIBOSOME	39	0.5339315	1.4924804	0.0227704	0.17409238
MITOCHONDRIAL_LUMEN	46	0.50879836	1.4758189	0.0317757	0.17456688
TRANSCRIPTION_COREPRESSOR_ACTIVITY	89	0.4590313	1.490839	0.017985612	0.17467281
ENDOSOME	66	0.4820446	1.4775469	0.025408348	0.1752333
NUCLEAR_ENVELOPE	71	0.47908732	1.4965032	0.013487476	0.17564651
BASE_EXCISION_REPAIR	16	0.6498335	1.4762158	0.046904314	0.17567416
VESICLE_COAT	15	0.6376046	1.4782321	0.06498195	0.1757659
RNA_HELICASE_ACTIVITY	23	0.5825216	1.4925414	0.048148148	0.1758714
INSOLUBLE_FRACTION	15	0.63462186	1.4947509	0.050092764	0.17640048
RRNA_PROCESSING	15	0.6450406	1.4971359	0.06994329	0.17676736
DAMAGED_DNA_BINDING	20	0.6099996	1.4931827	0.057086613	0.17689608
MITOCHONDRIAL_MATRIX	46	0.50879836	1.4785188	0.03888889	0.17701647
RNA_BINDING	247	0.3986095	1.4675852	0.001706485	0.17760554
MAGNESIUM_ION_BINDING	60	0.4835592	1.466134	0.023593467	0.17802367
RIBOSOME_BIOGENESIS_AND_ASSEMBLY	18	0.6119318	1.4787906	0.061068702	0.1784075
REGULATION_OF_JNK_ACTIVITY	20	0.60329854	1.480192	0.05065666	0.17858712
CELL_DIVISION	21	0.5873972	1.4632698	0.05514706	0.18028069
INTERPHASE	68	0.46598712	1.4518218	0.027777778	0.1913868
ENVELOPE	165	0.4114975	1.4528087	0.007561437	0.19148163
PROTEOLYSIS	188	0.40413597	1.4503274	0.003690037	0.19215819
TRANSCRIPTION_FACTOR_BINDING	299	0.3834482	1.4529533	0.001718213	0.19293213
RESPONSE_TO_ABIOTIC_STIMULUS	89	0.44741562	1.4335114	0.017274473	0.19819732
SULFUR_METABOLIC_PROCESS	37	0.5223308	1.4266143	0.051526718	0.1986143
RIBOSOMAL_SUBUNIT	20	0.56963205	1.4320418	0.09471767	0.19885813
SECONDARY_METABOLIC_PROCESS	26	0.5547487	1.4307259	0.07590133	0.19891776
RNA_POLYMERASE_II_TRANSCRIPTION_FACTOR_ACTIVITY	180	0.4043103	1.4295563	0.005454545	0.19903831
CYTOKINE_SECRETION	18	0.5882834	1.4336301	0.08761905	0.1995452
ESTABLISHMENT_OF_CELLULAR_LOCALIZATION	346	0.37105697	1.4246875	0.003430532	0.19984554
ATP_DEPENDENT_RNA_HELICASE_ACTIVITY	16	0.60728115	1.4266542	0.07663552	0.2000597
RIBONUCLEASE_ACTIVITY	25	0.53665286	1.4340386	0.065298505	0.20055543
RNA_POLYMERASE_COMPLEX	16	0.60243267	1.4268745	0.0729783	0.20122033
MACROMOLECULAR_COMPLEX_ASSEMBLY	269	0.38709733	1.434219	0.003669725	0.20184538
MYELOID_LEUKOCYTE_DIFFERENTIATION	15	0.6201914	1.4346573	0.083636366	0.20287399
INTRINSIC_TO_ORGANELLE_MEMBRANE	51	0.47990772	1.4186221	0.045787547	0.2030627

HYDROLASE_ACTIVITY_ACTING_ON_ESTER_BONDS	266	0.380421	1.4176143	0.001801802	0.20315768
DOUBLE_STRAND_BREAK_REPAIR	23	0.5540229	1.4214114	0.06678383	0.20340522
RNA_DEPENDENT_ATPASE_ACTIVITY	17	0.6057121	1.4201856	0.07561437	0.20384927
INTEGRAL_TO_ORGANELLE_MEMBRANE	49	0.47616753	1.4159075	0.047169812	0.20390706
ORGANELLE_MEMBRANE	294	0.37693542	1.4187137	0.005357143	0.20440683
MUSCLE_DEVELOPMENT	93	0.4376932	1.4346845	0.024347827	0.20444322
NUCLEAR_PORE	31	0.54541826	1.4351789	0.053435113	0.2052819
ORGANELLE_ENVELOPE	165	0.4114975	1.4360602	0.008992806	0.20561118
BIOPOLYMER_CATABOLIC_PROCESS	116	0.43042204	1.4392637	0.013035382	0.20639516
TRANSLATION_INITIATION_FACTOR_ACTIVITY	24	0.566066	1.4361031	0.07442748	0.20719925
NUCLEAR_DNA_DIRECTED_RNA_POLYMERASE_COMPLEX	16	0.60243267	1.4370297	0.07794677	0.20767653
CHROMOSOME_SEGREGATION	31	0.51835597	1.4081134	0.056751467	0.20851165
MICROTUBULE_ORGANIZING_CENTER	63	0.4567808	1.4070909	0.041366905	0.20866977
REGULATION_OF_CELLULAR_COMPONENT_ORGANIZATION_AND_BIOGENESIS	124	0.41717014	1.4110531	0.021937843	0.20889181
REGULATION_OF_PROGRAMMED_CELL_DEATH	340	0.36703455	1.4055814	0.006482982	0.20937587
MICROTUBULE_BINDING	32	0.5218439	1.4082321	0.060998153	0.20976861
CENTROSOME	54	0.47576094	1.4088695	0.053703703	0.21037792
CELLULAR_BIOSYNTHETIC_PROCESS	317	0.3708114	1.4038763	0.005244755	0.21060336
NUCLEOBASENUCLEOSIDENUCLEOTIDE_KINASE_ACTIVITY	25	0.5471401	1.4003767	0.0951417	0.2143522
CELLULAR_COMPONENT_ASSEMBLY	286	0.37529317	1.3978273	0.003472222	0.21676949
MEMBRANE_FUSION	28	0.53505266	1.392763	0.08704062	0.21722041
REGULATION_OF_DEVELOPMENTAL_PROCESS	438	0.35955036	1.3966223	0	0.21726146
TRANSCRIPTION_REPRESSOR_ACTIVITY	145	0.403447	1.3933865	0.023076924	0.21754538
NUCLEAR_MEMBRANE	50	0.4721272	1.3941983	0.05871886	0.21785823
ION_BINDING	267	0.3709369	1.3873031	0.010327023	0.21819216
CATION_BINDING	209	0.38312912	1.3909979	0.007104796	0.21825889
COATED_MEMBRANE	16	0.594315	1.3862225	0.09245283	0.21837151
POSITIVE_REGULATION_OF_CELL_PROLIFERATION	147	0.39856908	1.3844182	0.024390243	0.21842124
ENERGY_DERIVATION_BY_OXIDATION_OF_ORGANIC_COMPOUNDS	37	0.49774095	1.3786653	0.076241136	0.21860434
REGULATION_OF_I_KAPPAB_KINASE_NF_KAPPAB_CASCADE	90	0.4350825	1.3877624	0.037102472	0.21881363
DNA_DIRECTED_RNA_POLYMERASE_COMPLEX	16	0.60243267	1.3944545	0.10019268	0.21894494
SPINDLE_POLE	18	0.5763553	1.3774611	0.09487666	0.21898994
RESPONSE_TO_EXTRACELLULAR_STIMULUS	33	0.49717587	1.3740106	0.09854015	0.21913938
EXTRACELLULAR_SPACE	240	0.37198323	1.3756621	0.011925043	0.21923463
MEMBRANE_ORGANIZATION_AND_BIOGENESIS	133	0.4028322	1.3791856	0.023214286	0.21926086
CELLULAR_PROTEIN_CATABOLIC_PROCESS	58	0.45403567	1.3728802	0.0701107	0.21945141
DEOXYRIBONUCLEASE_ACTIVITY	22	0.5592031	1.3845932	0.09471767	0.21950641
MEMBRANE_COAT	16	0.594315	1.3743671	0.10288066	0.21988344
MITOCHONDRION	333	0.36088082	1.3760875	0.005263158	0.21989727
CELLULAR_LOCALIZATION	364	0.365569	1.388041	0.007017544	0.21990982
ESTABLISHMENT_OF_PROTEIN_LOCALIZATION	187	0.38707	1.3815149	0.015929203	0.22003803
REGULATION_OF_APOPTOSIS	339	0.36706337	1.3887557	0.001814882	0.22023664
MICROTUBULE_CYTOSKELETON_ORGANIZATION_AND_BIOGENESIS	34	0.50519955	1.3792572	0.06666667	0.22045796
ORGANELLAR_RIBOSOME	22	0.5427753	1.3802477	0.07749078	0.22046462
PROTEIN_CATABOLIC_PROCESS	68	0.44794676	1.3815653	0.043402776	0.22126277
MITOCHONDRIAL_RIBOSOME	22	0.5427753	1.3681889	0.091240875	0.22492132
TRANSCRIPTION_COFACTOR_ACTIVITY	223	0.36708304	1.3601531	0.012704174	0.22651556
COATED_VESICLE	46	0.47230852	1.3659687	0.08300395	0.22682688
NEGATIVE_REGULATION_OF_TRANSCRIPTION_FACTOR_ACTIVITY	15	0.57811624	1.360225	0.14583333	0.22765535
MITOCHONDRIAL_PART	140	0.39749578	1.3643752	0.03018868	0.22796294
MACROMOLECULE_LOCALIZATION	231	0.37079376	1.3631206	0.008880994	0.22841755
REGULATION_OF_CELL_PROLIFERATION	304	0.36117315	1.3606074	0.005405406	0.22844857
SPINDLE	38	0.48780012	1.3612522	0.07421875	0.22880894
POSITIVE_REGULATION_OF_SECRETION	20	0.5507547	1.3614603	0.11066398	0.22980347
REGULATION_OF_RNA_METABOLIC_PROCESS	461	0.3448012	1.3535823	0.005084746	0.23525625
MONOCARBOXYLIC_ACID_METABOLIC_PROCESS	86	0.42058134	1.3509461	0.04659498	0.23655356
REGULATION_OF_TRANSCRIPTIONDNA_DEPENDENT	455	0.34534505	1.3479588	0.001709402	0.23708351
PROTEIN_LOCALIZATION	211	0.3731059	1.3496699	0.02169982	0.23708674
EXONUCLEASE_ACTIVITY	19	0.56482637	1.3513999	0.12450593	0.23720872
NEGATIVE_REGULATION_OF_CELL_CYCLE	79	0.42087343	1.3469442	0.055762082	0.23742694
MRNA_BINDING	23	0.5339321	1.3482575	0.115311906	0.23784645
REGULATION_OF_SECRETION	40	0.47020572	1.3450394	0.096834265	0.23804884
SEXUAL_REPRODUCTION	138	0.388716	1.3452853	0.029929578	0.23894602
TRANSLATION_FACTOR_ACTIVITY_NUCLEIC_ACID_BINDING	38	0.48211548	1.3425198	0.1040146	0.24062918
CELLULAR_RESPONSE_TO_STIMULUS	19	0.55862397	1.3406193	0.11394892	0.24243978
POSITIVE_REGULATION_OF_I_KAPPAB_KINASE_NF_KAPPAB_CASCADE	84	0.42065594	1.3379456	0.059130434	0.24513318
NEGATIVE_REGULATION_OF_DEVELOPMENTAL_PROCESS	196	0.3733051	1.3368722	0.022569444	0.24551289
INTRACELLULAR_PROTEIN_TRANSPORT	142	0.384589	1.3352638	0.043809526	0.24655478

Gene sets with FDR <0.25 for ITF-B low LC50/GI50 group					
ISOMERASE_ACTIVITY	35	-0.647987	-1.8120486	0.002074689	0.1203844
ADHERENS_JUNCTION	22	-0.7272807	-1.8359196	0.002118644	0.14387879
CELL_JUNCTION	81	-0.57033104	-1.8644456	0	0.14899072
APICOLATERAL_PLASMA_MEMBRANE	34	-0.64566815	-1.8127947	0.002217295	0.14906
L_AMINO_ACID_TRANSMEMBRANE_TRANSPORTER_ACTIVITY	17	-0.7947959	-1.9020346	0	0.15723027
TIGHT_JUNCTION	31	-0.645349	-1.7577567	0.003898636	0.17274599
CALCIUM_INDEPENDENT_CELL_CELL_ADHESION	22	-0.6694639	-1.7172897	0.003952569	0.18960369
APICAL_JUNCTION_COMPLEX	34	-0.64566815	-1.7591468	0.004504505	0.19703126
INTERCELLULAR_JUNCTION	65	-0.5553819	-1.7201418	0	0.20288719
MESODERM_DEVELOPMENT	22	-0.66943175	-1.6937227	0.012552301	0.20340838
CELL_MIGRATION	94	-0.5077429	-1.701577	0	0.20372346
CYTOKINE_AND_CHEMOKINE_MEDIATED_SIGNALING_PATHWAY	22	-0.66552794	-1.7266408	0.008350731	0.21339622
INTERLEUKIN_RECEPTOR_ACTIVITY	19	-0.6674477	-1.6748273	0.010526316	0.2306989
CARBOHYDRATE_KINASE_ACTIVITY	15	-0.6996769	-1.6640166	0.016632017	0.24113375
