# Supplementary material for: Novel HDAC inhibitors exhibit pre-clinical efficacy in lymphoma models and point to the importance of CDKN1A expression levels in mediating their anti-tumor response
Source: Oncotarget. 2014 Dec 30;6(7):5059–71. doi: 10.18632/oncotarget.3239 (PMC4467133; doi:10.18632/oncotarget.3239)
Supplement: Supplementary file 9 [file oncotarget-06-5059-s009.pdf]

Supplementary table 8.

GENE	IC50 ITF-B	p-value IC50 ITF-B	IC50 ITF-A	p-value IC50 ITF-A	LC50 ITF-B	p-value LC50 ITF-B	LC50 ITF-A	p-value LC50 ITF-A
BCL2L1	0.227	0.219	**0.490**	**0.006**	0.147	0.429	0.219	0.236
SOD2	**0.428**	**0.017**	0.308	0.093	**0.569**	**0.001**	**0.542**	**0.002**
GSR	0.248	0.179	0.223	0.228	**0.444**	**0.013**	**0.419**	**0.020**
TXN	-0.060	0.748	-0.048	0.796	0.352	0.053	**0.384**	**0.034**
CREBBP	0.225	0.224	0.176	0.343	**0.431**	**0.016**	0.347	0.056
EP300	-0.334	0.067	-0.300	0.101	**-0.382**	**0.035**	**-0.485**	**0.006**

The table shows Spearman correlation coefficients for IC50 and LC50 values vs gene expression levels, with corresponding p-values listed in the adjacent column. Values in bold correspond to correlation coefficients with a p-value ≤0.05
